# Uncovering bacterial and functional diversity in macroinvertebrate mitochondrial-metagenomic datasets by differential centrifugation

**DOI:** 10.1038/s41598-019-46717-4

**Published:** 2019-07-16

**Authors:** Jan-Niklas Macher, Arjen Speksnijder, Le Qin Choo, Berry van der Hoorn, Willem Renema

**Affiliations:** Naturalis Biodiversity Center, PO Box 9517, 2300 RA Leiden, Netherlands

**Keywords:** Genetic techniques, Biodiversity, Ecosystem ecology, Bacteria, Molecular biology

## Abstract

PCR-free techniques such as meta-mitogenomics (MMG) can recover taxonomic composition of macroinvertebrate communities, but suffer from low efficiency, as >90% of sequencing data is mostly uninformative due to the great abundance of nuclear DNA that cannot be identified with current reference databases. Current MMG studies do not routinely check data for information on macroinvertebrate-associated bacteria and gene functions. However, this could greatly increase the efficiency of MMG studies by revealing yet overlooked diversity within ecosystems and making currently unused data available for ecological studies. By analysing six ‘mock’ communities, each containing three macroinvertebrate taxa, we tested whether this additional data on bacterial taxa and functional potential of communities can be extracted from MMG datasets. Further, we tested whether differential centrifugation, which is known to greatly increase efficiency of macroinvertebrate MMG studies by enriching for mitochondria, impacts on the inferred bacterial community composition. Our results show that macroinvertebrate MMG datasets contain a high number of mostly endosymbiont bacterial taxa and associated gene functions. Centrifugation reduced both the absolute and relative abundance of highly abundant Gammaproteobacteria, thereby facilitating detection of rare taxa and functions. When analysing both taxa and gene functions, the number of features obtained from the MMG dataset increased 31-fold (‘enriched’) respectively 234-fold (‘not enriched’). We conclude that analysing MMG datasets for bacteria and gene functions greatly increases the amount of information available and facilitates the use of shotgun metagenomic techniques for future studies on biodiversity.

## Introduction

Advances in DNA sequencing technology have led to an enormous increase in information on biodiversity across all levels, from genes to ecosystems, and revolutionized studies on biodiversity^1–3^. Shotgun metagenomics, i.e. sequencing and analysing millions of DNA fragments distributed randomly across whole genomes, and meta-mitogenomics (MMG), i.e. analysing the mitochondrial DNA of metazoa recovered from shotgun metagenomic datasets, are promising approaches to further deepen the understanding of biodiversity on both taxonomic and functional level^3–5^. Unlike amplicon-based techniques (e.g. metabarcoding^6^), shotgun metagenomic approaches do not suffer from PCR and primer bias^3,7^ and allow assessment of taxonomic composition and biomass of both microbial and macroinvertebrate samples^8,9^. To date, however, MMG studies on macroinvertebrates suffer from comparatively low efficiency and commonly require high sequencing depth. While differential centrifugation can significantly increase mitochondrial DNA yield^10^, roughly 90% (and commonly >99% in studies not applying enrichment^3^) of sequences are of non-mitochondrial origin and can mostly not be used for macroinvertebrate species identification due to missing references for nuclear DNA fragments. Usability and cost efficiency of MMG studies will increase if the information in the >90% non-mitochondrial DNA was included in analyses, e.g. by screening for nuclear genes^11^, and taxa and functions of often abundant microbiota like fungi, viruses, protists or bacteria, which are commonly found in association with macroinvertebrates^12–14^. Analysing the functional potential of taxa associated with macroinvertebrates can be useful for the assessment of environmental factors on species and ecosystems. Both ecological studies and potential applications like biomonitoring could benefit from including such data, as it might allow identifying the role of organisms in the studied ecosystem.

Here we test whether information on bacterial taxa and gene functions can be extracted from macroinvertebrate MMG datasets, and whether using differential centrifugation will not only enrich for mitochondria, but also for macroinvertebrate-associated microbiota. Mitochondria retain many characteristics of their bacterial origin^15^, and we therefore hypothesise that a differential centrifugation approach as described in^10^ also enriches for bacteria associated with the studied macroinvertebrates. The approach could increase efficiency of MMG studies by allowing to extract a wealth of additional information from the yet unused data. Second, we hypothesize that differential centrifugation will change the inferred community composition of macroinvertebrate-associated bacteria in MMG datasets when compared to non-enriched samples, as differential centrifugation is known to selectively enrich for specific cell size and weights. Low centrifugal forces pellet out heavy cells and cell fractions, while lightweight cells and cell fractions remain in suspension. If the goal is to analyze the latter, only the light fraction of the samples is retained, thereby losing information on heavier cells and fractions present in the sample^16,17^.

We argue that analysing the >90% of non-mitochondrial reads in MMG datasets for microbiota and gene functions allows better insights into the biodiversity of macroinvertebrate communities. Subsequently, the gained knowledge could be used for studies on biodiversity and ecology, and its potential for applications such as biomonitoring can be investigated.

## Results

We showed that it is possible to obtain information on bacterial taxonomic diversity and functional potential from a macroinvertebrate MMG dataset, and that enrichment via differential centrifugation results in a different community profile from a non-enriched dataset.

Analyses of taxonomic diversity of Bacteria in the MMG dataset were performed using MG-RAST^18^. For taxonomic assignment, we used the NCBI RefSeq^19^ protein database as implemented in MG-RAST. Across all samples, a total of 42 taxa (35 Bacteria, 4 Eukaryota, 2 Virus, one unclassified) were identified on order level (see Supplementary Information 1 for the full table). A total of 713 associated functional categories (SEED level 3) were identified using the SEED protein function database^20^ as implemented in MG-RAST (see Supplementary Information 2 for the full table of functions). Analysing 100 000 high quality reads was sufficient for the reliable recovery of diversity in both the ‘enriched’ and the ‘not enriched’ dataset based on rarefaction curves showing that the number of additional taxa per read reached saturation (Supplementary Fig. 1).

### Taxonomic assignment

**Figure 1:**
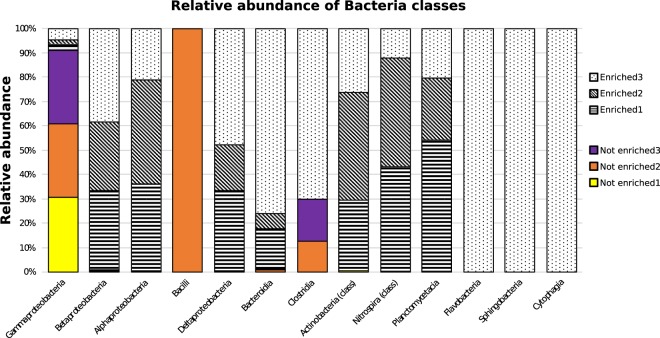
Relative abundance of hits per bacterial class, with coloured bars indicating ‘non-enriched’ and patterned bars indicating ‘enriched’ samples as in the legend.

On average 87,673 hits in the dataset not enriched for mitochondria (further: ‘not enriched’) and an average of 5,153 hits in the dataset enriched for mitochondria (further: ‘enriched’) were annotated to a taxonomic name. Bacteria made up the majority of hits in all samples, with an average abundance of 85.83% in ‘enriched’ samples and average abundance of 92.35% in ‘not enriched’ samples (see Table 1 for abundance per replicate). Gammaproteobacteria, and within those the genera *Vibrio* and *Escherichia*, were the most abundant taxa in the ‘not enriched’ dataset. Alpha- and Betaproteobacteria were the dominant groups in the ‘enriched’ samples (Table 1; Fig. 1).

**Table 1:** Relative abundance of all Bacteria, and classes found at more than 2% relative abundance: Gammaproteobacteria (with focus on genera *Vibrio* and *Escherichia*), Alphaproteobacteria and Betaproteobacteria, per replicate (R1–R3) in the ‘not enriched’ and ‘enriched’ datasets. Mann-Whitney U statistics describing the difference between groups are reported. Significant differences are highlighted in bold.

Treatment	Not enriched			Enriched			Mann Whitney U
Replicates	R1	R2	R3	R1	R2	R3	
Bacteria	99.11	99.22	78.72	83.33	85.53	88.63	U = 6 P = 0.35
Gammaproteobacteria	**98.71**	**97.2**	**78.19**	**6.27**	**5.45**	**12.9**	**U = 9** **P = 0.05**
* Vibrio*	**52.99**	**46.3**	**11.87**	**0.00**	**0.00**	**0.00**	**U = 9** **P = 0.03**
* Escherichia*	**20.44**	**40.55**	**20.89**	**0.34**	**0.00**	**0.57**	**U = 9** **P = 0.05**
Alphaproteobacteria	**0.09**	**0.13**	**0.12**	**36.99**	**44.99**	**23.28**	**U = 0** **P = 0.05**
Betaproteobacteria	**0.28**	**0.34**	**0.25**	**36.05**	**31.85**	**44.73**	**U = 0** **P = 0.05**

All other Bacteria classes were found with a relative abundance of less than 2% in any of the samples. The class Bacilli was exclusively found in the ‘not enriched’ dataset, while the classes of Cytophagia, Flavobacteria, Nitrospira, Planctomycetacia and Sphingobacteria were exclusively detected in the ‘enriched’ dataset. Bacilli were exclusively found in ‘not enriched’ replicate 2, while Cytophagia, Flavobacteria, Sphingobacteria were exclusively found in ‘enriched replicate 3′ (Fig. 1).

**Figure 2:**
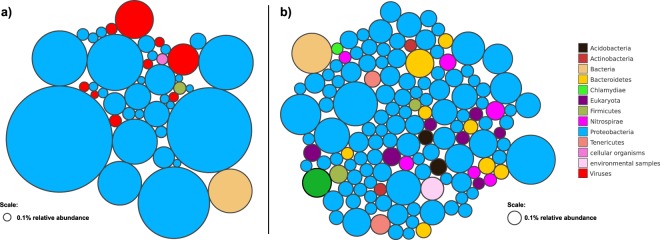
Bubble plots showing the number and abundance of taxa reaching more than 0.1% of relative abundance in ‘not enriched’ (**a**) and ‘enriched’ samples. Each bubble represents one species; the bubble diameter represents the relative abundance of taxa in the dataset.

To confirm the results of taxonomic assignment using MG-RAST, we used the taxonomic classification approach implemented in Kaiju^21^, which uses the NCBI RefSeq non-redundant protein database for taxonomic assignment. For this, we analysed all three replicates per treatment (‘enriched’ and ‘not enriched’) together. Using Kaiju, 278,033 of 300,000 DNA reads across all three ‘not enriched’ samples could be assigned to a taxonomic name, and 4,917 taxa were identified. Across the three ‘enriched’ samples, 153,090 reads could be assigned to a taxonomic name, and 11,557 taxa were identified (see Supplementary Information 3 and 4 for tables containing taxa and abundance). Figure 2 shows taxa that reached more than 0.1% abundance in the ‘not enriched’ (Fig. 2a) respectively the ‘enriched’ samples (Fig. 2b), identified to the taxonomic level of phylum (or higher level in case no lower level taxonomy was available for the identified taxa).

Gammaproteobacteria were the most abundant taxon in the ‘not enriched’ samples (219,158 assigned reads; 73.1% of reads), while they made up 12,175 reads (4.1%) in the ‘enriched’ samples. In the latter, Alpha- and Betaproteobacteria dominated with 46,795 (15.6%) respectively 39,066 reads (13%).

### Function assignment

Analyses of functional potential of Bacteria in the MMG dataset were also performed using MG-RAST^18^. An average of 45,379 (‘not enriched’) respectively 2,834 (‘enriched’) hits were assigned to a function using the SEED database. On average 35,520 (‘not enriched’) respectively 1138 (‘enriched’) of these were assigned to bacterial taxa.

**Figure 3:**
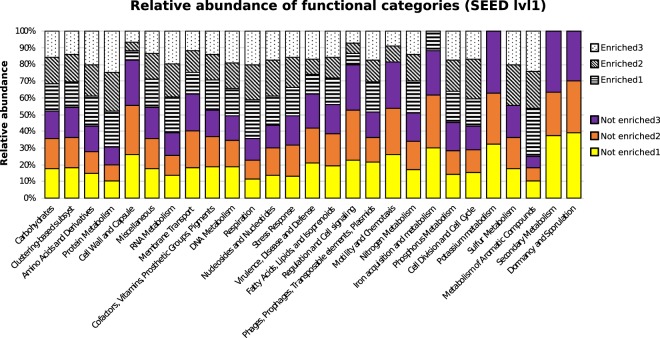
Relative abundance of bacteria-associated functional categories, with coloured bars indicating ‘non-enriched’ and patterned bars indicating ‘enriched’ samples as in the legend.

The largest fractions of functions associated with bacteria were identified as belonging to the ‘Carbohydrate’ metabolism group (average; ‘not enriched’: 15.7%; ‘enriched’: 14.3%) respectively the ‘Protein Metabolism’ group (‘not enriched’: 6.6%, ‘enriched’: 15.1%). See Supplementary Information 2 for the full list of functions. All functional categories had a higher abundance in the ‘not enriched’ samples. Within the ten most abundant functional categories, only one (‘Cell wall and capsul’e) showed a strong difference between ‘not enriched’ and ‘enriched’ samples, with a high relative abundance in ‘not enriched’ samples (Fig. 3).

### Alpha diversity

We here describe the results of taxonomic assignment on order level and SEED functional level 3.

### Bacteria

**Figure 4:**
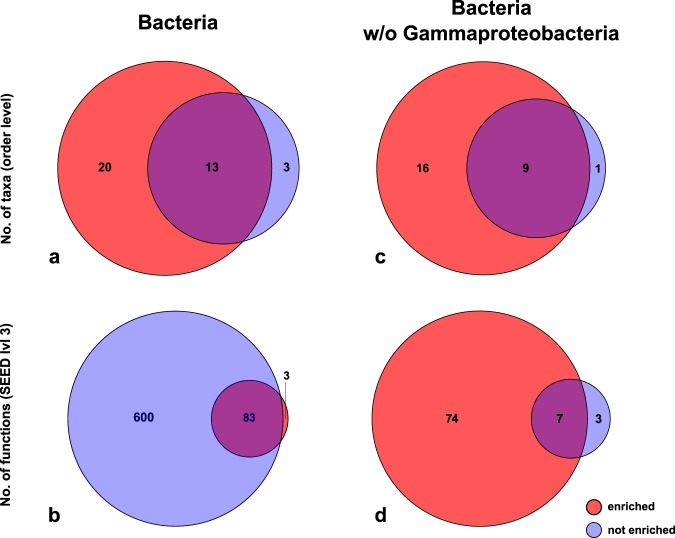
Venn diagrams showing the number of taxa (order level) respectively gene functions (SEED level 3) found exclusively in ‘enriched’ samples, exclusively in ‘not enriched’ samples, and found with both techniques. (**a**) All taxa, orders; (**b**) All taxa, gene functions; (**c**) Bacteria, orders; (**d**) Bacteria, gene functions; (**e**) Bacteria without Gammaproteobacteria, orders; (**f**) Bacteria without Gammaproteobacteria, gene functions.

In total, 36 orders of Bacteria were identified. In the ‘not enriched’ samples, a minimum of 12 and a maximum of 13 orders were found. In the ‘enriched’ samples, a minimum of 25 and a maximum of 28 orders were identified (Table 2). Across all replicates, the majority of taxa (55.6%) were found exclusively in the ‘enriched’ dataset, 36.1% were found in both datasets, and 8.3% were found exclusively in the ‘not enriched’ dataset (Fig. 4a).

**Table 2:** Number of bacterial orders respectively bacteria-associated functions (SEED lvl3) found in three replicates (R1–R3) in ‘not enriched’ respectively ‘enriched’ samples. Mann-Whitney U statistics describing the difference between groups are reported. Significant differences are highlighted in bold.

Treatment	Not enriched			Enriched			Mann-Whitney U
Replicates	R1	R2	R3	R1	R2	R3	
Bacteria, orders	**12**	**13**	**13**	**28**	**25**	**26**	**U = 0** **P = 0.04**
Bacterial w/o Gammaproteobacteria, orders	**6**	**7**	**7**	**20**	**17**	**21**	**U = 0** **P = 0.04**
Bacteria-associated functions, SEED lvl3	**607**	**621**	**552**	**65**	**67**	**47**	**U = 9** **P = 0.05**
*Bacteria-associated* *functions w/o* *Gammaproteobacteria,SEED lvl3*	**0**	**10**	**0**	**63**	**63**	**39**	**U = 0** **P = 0.04**

A total of 686 gene functions were identified. In the ‘not enriched’ samples, a minimum of 552 and a maximum of 621 functions were found. In the ‘enriched’ samples, a minimum of 47 and a maximum of 67 functions were identified (Table 2). 12.1% of these functions were found in both datasets, 0.6% exclusively in the ‘enriched’ dataset, and the majority (87.5%) were exclusively found in the ‘not enriched’ dataset (Fig. 4b).

### Bacteria excluding Gammaproteobacteria

When the highly abundant Gammaproteobacteria were removed from the Bacteria dataset, a total of 14,278 taxonomic hits remained. In total, 26 orders were identified. In the ‘not enriched’ samples, a minimum of 6 and a maximum of 7 orders were found. In the ‘enriched’ samples, a minimum of 17 and a maximum of 21 orders were identified (Table 2). 34.6% of orders were found with both techniques, one order (3.9%) was exclusively found in the ‘not enriched’ dataset, and the majority (61.5%) was found exclusively in the ‘enriched’ dataset (Fig. 4c).

After removing the Gammaproteobacteria from the Bacteria dataset, 3,188 hits against SEED functional categories and a total of 84 out of 686 functions remained. In the ‘not enriched’ samples, a minimum of 0 and a maximum of 10 functions were found. In the ‘enriched’ samples, a minimum of 39 and a maximum of 63 functions were identified (Table 2). Across all replicates, the majority (88.1%) of these functions were exclusively found in the ‘enriched’ dataset, 3.6% were found exclusively in the ‘not enriched’ dataset, and 8.33% were found in both datasets (Fig. 4d).

### Beta diversity

#### Within techniques

Bray-Curtis Dissimilarity of Bacteria on order level was highest between the ‘not enriched’ (0.26; ‘enriched’: 0.21) samples. Bray-Curtis Dissimilarity of SEED functions (level 3) was highest between the ‘enriched’ samples (0.27; ‘not enriched’: 0.16).

#### Between techniques

Beta diversity (as Bray-Curtis dissimilarity) on Bacteria order level was 0.48 between techniques in the Bacteria dataset. NMDS analyses showed that ‘enriched’ and ‘not enriched’ samples form distinct clusters without overlap (Supplementary Fig. 2a).

On functional level (SEED level 3), Bray Curtis Dissimilarity between techniques was 0.54 in the Bacteria dataset. NMDS analyses showed that ‘enriched’ and ‘not enriched’ samples form distinct clusters with a slight overlap (Supplementary Fig. 2b).

#### Increase in number of extracted features

The mock-communities were assembled from the three macroinvertebrate species *Gammarus roeselii*, *Hydropsyche exocellata* and *Corbicula fluminea*. One additional species, the acanthocephalan endoparasite *Pomphorhynchus laevis*, was discovered by enrichment for mitochondria and mitogenome skimming^10^.

By including the macroinvertebrate-associated bacteria found across the three replicates per technique, the number of taxa on order level increased to 19 (6.33 fold) without enrichment, respectively 37 (9.25 fold) with enrichment. When both taxa and functions (as identified using the MG-RAST server with the SEED functional database) were treated as features characterising the studied community, the number of features rose to 702 (234 fold, ‘not enriched’) respectively 123 (30.75 fold, ‘enriched’) (all information: Table 3).

**Table 3:** Increase in number of ecosystem features when including macroinvertebrate taxa, bacterial taxa and bacteria-associated functions on order and SEED level 3.

Treatment	Not enriched	Enriched
Macroinvertebrate taxa	3	4
Bacterial taxa	16	33
**Sum (fold increase)**	**19 (6.33)**	**37 (9.25)**
Bacterial functions	683	86
**Sum (fold increase)**	**702 (234)**	**123 (30.75)**

## Discussion

Mitochondrial metagenomics (MMG) can be used to study the taxonomic composition of macroinvertebrate samples via mitogenome skimming^22,23^. Still, the vast majority of reads in these studies is not used, making the approach less cost-efficient than amplicon-based approaches such as metabarcoding^3^. We here show that MMG datasets targeting macroinvertebrates can also be used to extract information on taxonomic diversity and functional potential of macroinvertebrate-associated microbiota. The gained knowledge can be used for biodiversity studies, and potential future applications such as the use for biomonitoring and ecological studies can be investigated.

We showed that analysing 100 000 high quality reads allowed reliable recovery of bacterial diversity in both the ‘enriched’ and the ‘not enriched’ dataset for the small mock community containing three macroinvertebrate species. However, we acknowledge that sequencing and analysing more reads might allow detecting more very rare bacterial taxa.

We hypothesised that a differential centrifugation approach as described in^10^ will not only enrich for macroinvertebrate mitochondria, but also bacteria associated with the studied macroinvertebrates. We demonstrate that fewer hits against the bacterial references were found in the ‘enriched’ samples, possibly because the differential centrifugation removed highly abundant bacteria for which reference sequences exist in the databases. Rare bacterial taxa and the majority of nuclear DNA fragments of the host macroinvertebrates cannot be assigned to taxonomic names or functions due to missing references. We conclude that the differential centrifugation protocol does not enrich for bacteria, but an increase in available references might change results in future experiments. Second, we hypothesised that the centrifugation approach will lead to a different inferred community composition of macroinvertebrate-associated bacteria, as differential centrifugation is known to enrich for certain sizes and weights. Using Kaiju and MG-RAST for taxonomic assignment, we could show that despite a lower overall yield of bacteria, differential centrifugation leads to a higher number of discovered taxa in enriched samples, and indeed leads to a change in inferred community composition, as also shown by beta diversity analyses. NMDS plots showing the Bray-Curtis dissimilarity between treatments show that ‘not enriched’ respectively ‘enriched’ samples form distinct clusters with little overlap. However, du to the limited number of samples, we refrain from drawing definite conclusions based on this analyses. Our results are in line with those of studies on other ecosystems, which found centrifugation approaches to be effective for detecting rare microbial taxa^24,25^. On the other hand, using the SEED database, more gene functions were found in samples not enriched by differential centrifugation. We point out that the MG-RAST approach does not allow definite linking of taxa and functions, as several taxa can share the same functions. Still, removing the Gammaproteobacteria, including the two most abundant taxa *Escherichia* and *Vibrio*, and subsequently all functions exclusively associated with these taxa, resulted in higher taxonomic and functional alpha diversity in the ‘enriched’ dataset, showing that a large part of functional diversity was contributed by these taxa. *Escherichia* and *Vibrio* are common intestinal bacteria in many metazoan organisms^26–29^. They often attach to the intestines of their host^30–32^ and we assume that due to this, these taxa are pelleted out together with the heavier cell debris during differential centrifugation. This is also supported by the finding that genes coding for proteins in the functional category of ‘Cell wall and capsule’ were found in high relative abundance in ‘not enriched’ samples, but with a low abundance in ‘enriched’ samples, which indicates that bacteria with cell walls like *Escherichia* were efficiently removed by differential centrifugation. This removal of highly abundant taxonomic groups leads to a higher inferred taxonomic diversity in the ‘enriched’ samples, as rare taxa are less likely to be outcompeted during sequencing.

Our results have several implications for future studies on taxonomic and functional diversity of macroinvertebrates and their associated microbiota. We demonstrate that enrichment via differential centrifugation biases abundance of taxa and functional diversity by removing highly abundant bacteria from the dataset. This was confirmed by Bray-Curtis dissimilarity analyses, which showed that community composition was more different between ‘enriched’ and ‘not enriched’ samples than within the respective techniques. Further studies investigating this phenomenon are needed, but our results suggest that enrichment by differential centrifugation should not be used to infer abundance data of macroinvertebrate-associated microbiota. However, by greatly reducing the number of intestinal Gammaproteobacteria, differential centrifugation can be beneficial for studies that aim at assessing a larger part of the macroinvertebrate-associated microbial diversity. The approach allows detection of rare taxa, which can be highly important for reference-library build-up and ecological studies^33,34^. Combining both techniques will give the most accurate results on both abundance and diversity of taxa and functions, and should be considered depending on the research question.

Further, our results suggest that combining the taxonomic information on macroinvertebrates gained through mitogenome skimming with those of the associated microbial taxa and functions could be highly beneficial for studies on biodiversity of macroinvertebrates. By including the macroinvertebrate-associated bacteria and their gene functions, the number of potentially informative features increased 234 (‘not enriched’) respectively 31-fold (‘enriched’).

Further research is needed to test to what extend macroinvertebrate-associated bacteria and gene functions extracted from MMG datasets can be used for applications like biomonitoring and in ecological studies, but studies on gut microbiota show that the bacterial community can be ecologically informative^35–37^, and microbial communities are routinely used to monitor ecosystems^38–40^. We suggest both mock-community experiments in which every species is also shotgun sequenced separately, and case studies in environmental gradients that allow assessing the power of the here-proposed approach for ecological studies. RNA-based analyses will further show to what extent the findings on functional potential present in the community, as described here, correspond to actual gene expression, i.e. active genes. Further, we expect that more complete reference databases comprising whole annotated bacterial and eukaryotic genomes will increase the accuracy of both taxonomic and functional assignment. Currently, the number of sequenced and annotated genomes is much higher for bacteria, and Eukaryotaic protein data is underrepresented in the databases, especially for non-model macroinvertebrates. This will lead to an overestimation of bacterial abundance, as the macroinvertebrate DNA cannot be assigned to taxonomic names and functional categories due to missing references. Reference database gaps are a well-recognized challenge in biodiversity studies even on microbial taxa, and lead to biases in diversity estimates, as e.g. described in^41^. In addition, using strict filtering thresholds for identification of taxa and functions can reduce the number of false-positives and avoid overestimating diversity, but increase the number of false-negatives in the dataset and can lead to underestimation of diversity. A trade off will be imminent in any study. We therefore are dependent on the sequencing and annotation of full eukaryotic genomes and genomes of rare and little studied microbial taxa in order to fill reference databases. This task will require enormous efforts of the scientific community, but will become more affordable due to the decrease in sequencing costs. Future studies should also investigate different classification approaches for taxonomic and functional assignment of metagenomic datasets^42,43^, and techniques such as supervised machine learning, which are promising for the analyses of datasets containing yet unknown diversity^44,45^, should be investigated.

We conclude that the here described approach of extracting information on microbial taxa and gene functions can greatly increase the number of features available for analyses, thereby allowing to optimise the use of MMG datasets and facilitating this technique for future studies on biodiversity.

## Material and Methods

The study is based on samples and DNA sequences described in^10^. In brief, six mock communities, each containing three *Gammarus roeselii*, three *Hydropsyche exocellata* and one *Corbicula fluminea*, were assembled by sampling the specimens from one sampling site (Gillbach, Germany) and pooling them together in the laboratory. Three of these mock-communities were enriched for mitochondria (further: ‘enriched’) by differential centrifugation, and three were not enriched (further: ‘not enriched’). From each mock-community, 100 000 high quality DNA reads (as described in^10^) were further analysed in this study. Taxonomic and functional annotation was conducted using the MG-RAST webserver^18,46,47^, a commonly used resource for analyses of bacterial metabarcoding and metagenomic datasets from a wide range of ecosystems^48–53^. The approach relies on the translation of DNA to protein coding sequences and uses the NCBI RefSeq protein database to assign taxonomy to reads. Databases containing information on protein functions are used for functional annotation and subsequent identification of functional potential of organisms. As analysed reads can cover several proteins, the number of matches (reported as hits) can be higher than the number of analysed reads. Taxa can be linked to functional categories, but the same function can be shared by several taxonomic groups (full manual for MG-RAST with all details available online: (https://github.com/MG-RAST/tech-report). The default and commonly used settings in MG-RAST (e-value e^−5^, minimum 60% identity to reference, 15 bp alignment length and minimum abundance of 1) were changed to e^−20^, minimum 90% identity, 50 bp alignment length and a minimum abundance of 10 in order to get a more conservative, reliable assignment of reads to taxa and functions^54^. The ‘representative hit’ function was used to prevent overinflation of hit counts. Functions were annotated to sequences based on the SEED functional subsystems database^20^. The SEED database contains genomes that are annotated and curated by experts, and protein families with known functions are extracted from this curated dataset. Functions are grouped into functional groups (lvl1, lvl2, lv3, functions, i.e. from broader functional categories to single protein functions). Rarefaction curves showing the increase in number of species per read were extracted from MG-RAST to see whether a sufficient sequencing depth for the following analyses was reached. To confirm results of taxonomic assignment using MG-RAST, we used the taxonomic assignment approach implemented in Kaiju^21^ (available online: http://kaiju.binf.ku.dk) using the NCBI non redundant protein database containing Bacteria, Archaea, Viruses, Fungi and microbial eukaryotes. The minimum match length was set to 15, minimum match score to 75, and 4 mismatches allowed, which is stricter than the default settings (default: minimum match length: 11, minimum match score: 75, 5 mismatches allowed). Bubble plots showing the abundance and number of identified taxa were downloaded from the Kaiju server.

All data obtained from MG-RAST were further analysed in R^55^ using the package vegan^56^. Alpha diversity was calculated across all three replicates per technique on the taxonomic level of order, as this allows reliable identification with the used MG-RAST settings, as well as for SEED functional lvl3. These calculations were performed for Bacteria, for which the MG-RAST approach is optimised due to the high number of sequenced and annotated bacterial genomes in the reference databases. For visualization of taxonomic and functional diversity the levels of class respectively SEED level 1 were chosen to retain readability. Stacked bar plots were calculated for visualisation, with the hit number of bacterial classes sqrt transformed to allow visualisation of rare taxa. Stacked bar plots were also used to show relative abundance of Bacteria classes, and hit number and relative abundance of SEED functional categories on level 1. The R package VennDiagram^57^ was used to visualise the number of taxa and functions found with both treatments, respectively found exclusively with one of the treatments, across all replicates. Further, to assess contribution of Gammaproteobacteria to overall functional diversity, Alpha diversity was calculated for datasets excluding Gammaproteobacteria and functions exclusively associated with these taxa, and Venn diagrams showing the differences between treatments were calculated. Bray-Curtis dissimilarity was calculated within and between techniques, based on hit counts that were transformed into relative abundances. The metaMDS function as implemented in the vegan package was used to calculate NMDS plots based on Bray-Curtis dissimilarity of all taxa (order level) and all functional categories (SEED, level 3). One-sided Mann-Whitney U tests were run to test for significant differences between ‘not enriched’ and ‘enriched’ samples. Further, we calculated the increase in number of taxa and functions (i.e. ecosystem features) that can be extracted from the MMG datasets when adding the macroinvertebrate-associated bacterial taxa and gene functions to the original dataset, which contains solely the taxonomic information on macroinvertebrate taxa.

## Supplementary information

Supplementary_Information.pdf

## Data Availability

All raw data is available from the SRA (SRA number: SAMN07828199 - SAMN07828210).
