# Supplementary material for: Uncovering bacterial and functional diversity in macroinvertebrate mitochondrial-metagenomic datasets by differential centrifugation
Source: Sci Rep. 2019 Jul 16;9:10257. doi: 10.1038/s41598-019-46717-4 (PMC6635389; doi:10.1038/s41598-019-46717-4)
Supplement: Supplementary file 1 — Supplementary_Information.pdf [file 41598_2019_46717_MOESM1_ESM.pdf]

Supplementary Information

Uncovering bacterial and functional diversity in macroinvertebrate mitochondrial-metagenomic datasets by differential centrifugation

Jan-Niklas Macher (JNM)[1], Arjen Speksnijder (AS)[1], Le Qin Choo (LQC)[1], Berry van der Hoorn (BvdH)[1], Willem Renema (WR)[1]*

[1] Naturalis Biodiversity Center, PO Box 9517, 2300 RA Leiden, Netherlands

[*]Corresponding author

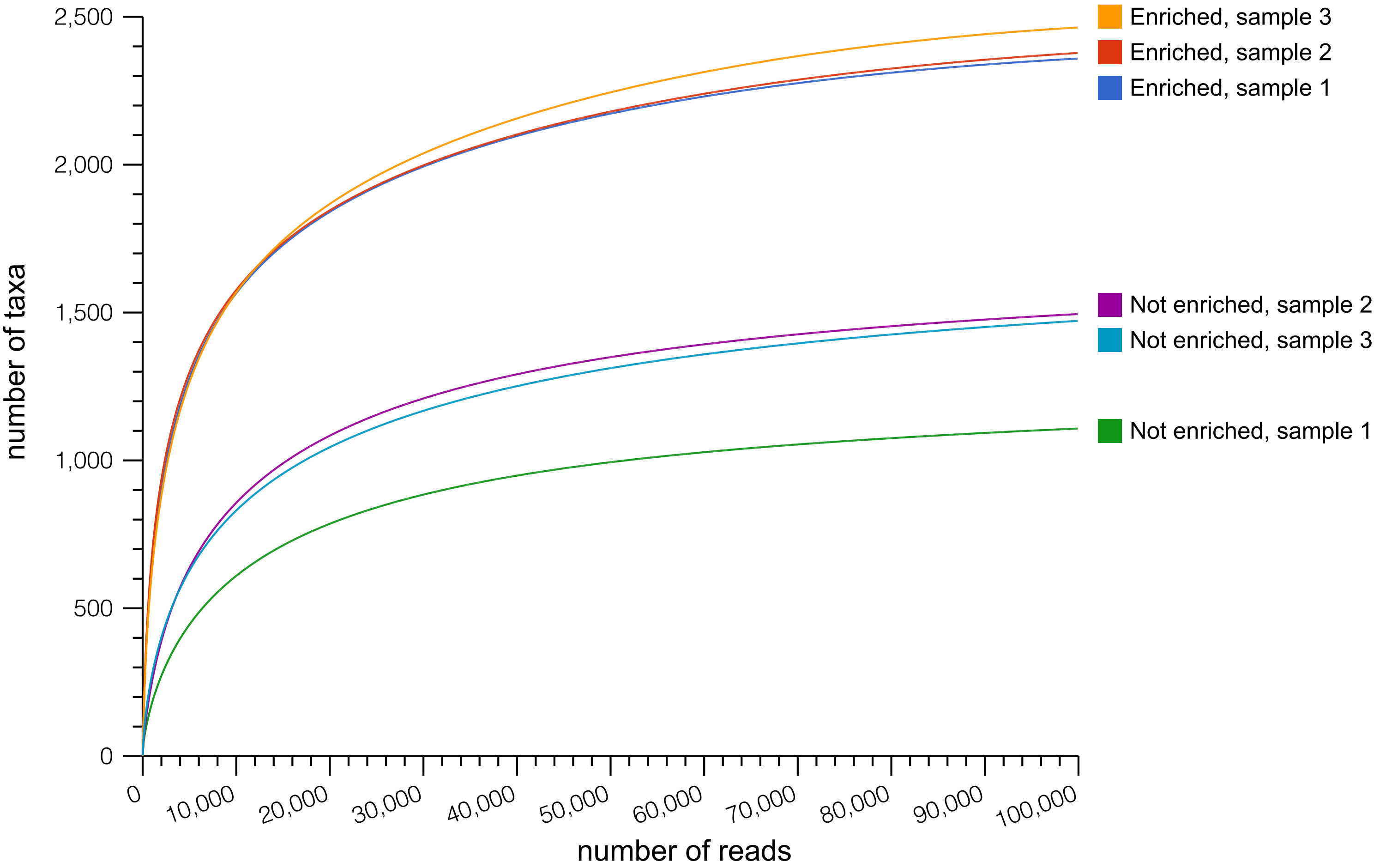

Supplementary Figure 1: Rarefaction curves showing the increase in taxa number per read

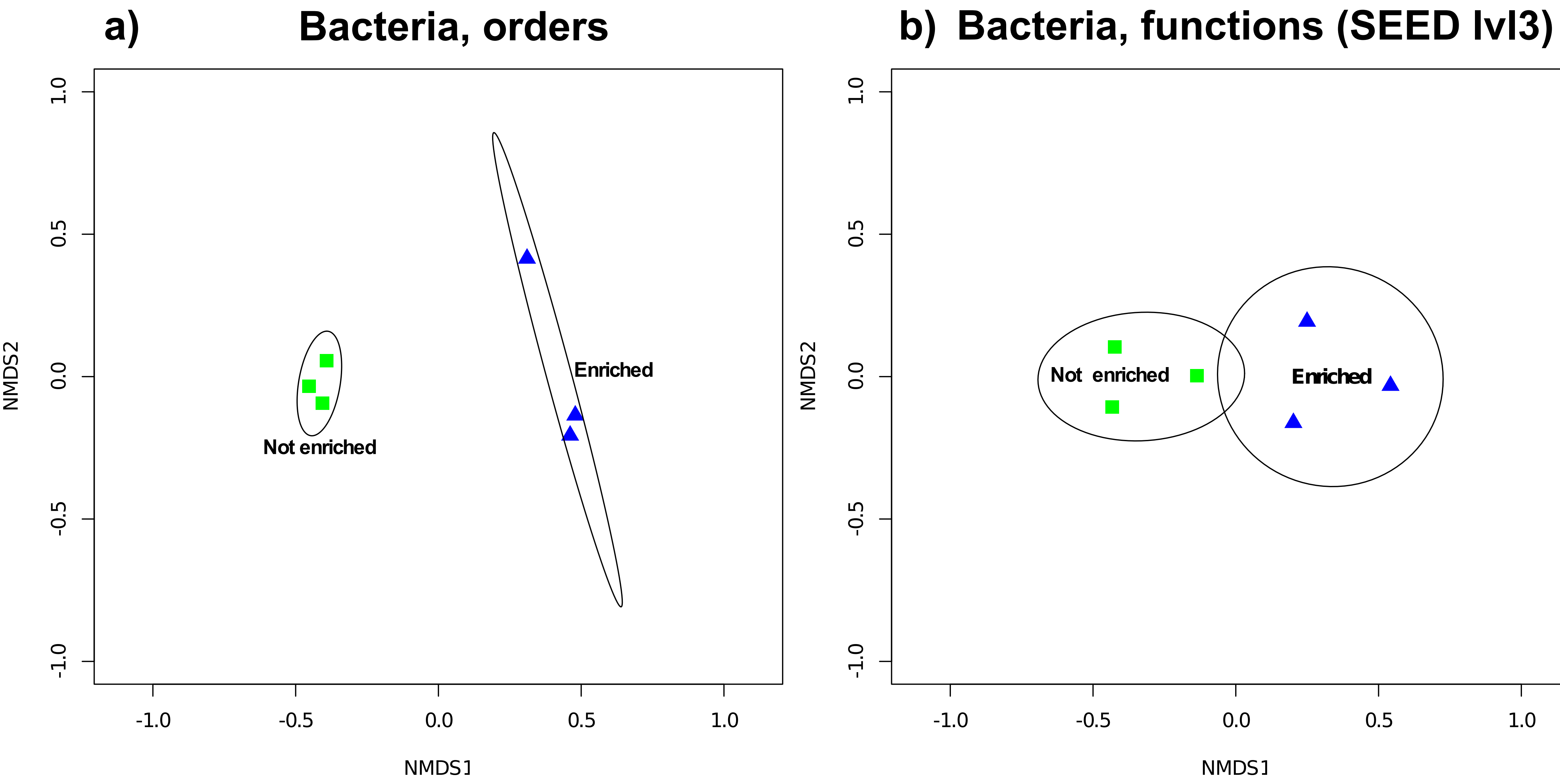

Supplementary Figure 2: NMDS plots based on beta diversity (Bray-Curtis). Green squares: 'Not enriched' samples; blue triangles: 'Enriched' samples.
a) Bacteria, order level taxonomy; b) Bacterial functions, SEED lvl3

Supplementary Information 1

Uncovering bacterial and functional diversity in macroinvertebrate mitochondrial-metagenomic datasets by differential centrifugation

Jan-Niklas Macher (JNM)[1], Arjen Speksnijder (AS)[1], Le Qin Choo (LQC)[1], Berry van der Hoorn (BvdH)[1], Willem Renema (WR)[1]*
[1] *Naturalis Biodiversity Center, PO Box 9517, 2300 RA Leiden, Netherlands*
[*] *Corresponding author*

Supporting Information 1: Taxonomic levels (domain, phylum, class, order, family; hit counts for all samples ('Enriched 1-3'; Not enriched 1-3).

domain	phylum	className	order	family	Enriched 1	Enriched 2	Enriched 3	Not enriched 1	Not enriched 2	Not enriched 3
Bacteria	Proteobacteria	Alphaproteobacteria	Rhodospirillales	Acetobacteraceae	22	13	0	0	0	0
Bacteria	Proteobacteria	Gammaproteobacteria	Aeromonadales	Aeromonadaceae	50	75	125	2995	2269	13057
Bacteria	Proteobacteria	Betaproteobacteria	Burkholderiales	Alcaligenaceae	28	19	36	0	0	0
Bacteria	Proteobacteria	Gammaproteobacteria	Alteromonadales	Alteromonadaceae	0	0	0	23	14	11
Bacteria	Bacteroidetes	Bacteroidia	Bacteroidales	Bacteroidaceae	17	0	59	0	14	0
Bacteria	Proteobacteria	Alphaproteobacteria	Rhizobiales	Beijerinckiaceae	11	0	0	0	0	0
Bacteria	Proteobacteria	Alphaproteobacteria	Rhizobiales	Bradyrhizobiaceae	73	71	38	47	46	25
Bacteria	Proteobacteria	Alphaproteobacteria	Rhizobiales	Brucellaceae	0	14	10	0	0	0
Bacteria	Proteobacteria	Betaproteobacteria	Burkholderiales	Burkholderiaceae	143	141	197	244	249	132
Bacteria	Proteobacteria	Alphaproteobacteria	Caulobacterales	Caulobacteraceae	10	20	14	0	0	0
Bacteria	Firmicutes	Clostridia	Clostridiales	Clostridiaceae	0	0	0	0	84	77
Bacteria	Proteobacteria	Betaproteobacteria	Burkholderiales	Comamonadaceae	1298	1062	1109	12	19	17
Eukaryota	Arthropoda	Insecta	Diptera	Culicidae	0	0	12	0	0	0
Bacteria	Bacteroidetes	Cytophagia	Cytophagales	Cytophagaceae	0	0	12	0	0	0
Bacteria	Proteobacteria	Deltaproteobacteria	Desulfovibrionales	Desulfovibrionaceae	51	28	83	0	0	15
Bacteria	Proteobacteria	Gammaproteobacteria	Chromatiales	Ectothiorhodospiraceae	10	0	0	0	0	0
Bacteria	Proteobacteria	Gammaproteobacteria	Enterobacteriales	Enterobacteriaceae	49	19	63	42723	42026	36386
Bacteria	Firmicutes	Bacilli	Lactobacillales	Enterococcaceae	0	0	0	0	1248	0
Bacteria	Proteobacteria	Alphaproteobacteria	Sphingomonadales	Erythrobacteraceae	168	196	78	0	0	13
Bacteria	Proteobacteria	Gammaproteobacteria	Alteromonadales	Ferrimonadaceae	0	0	0	0	0	16
Bacteria	Bacteroidetes	Flavobacteria	Flavobacteriales	Flavobacteriaceae	0	0	21	0	0	0
Bacteria	Proteobacteria	Betaproteobacteria	Gallionellales	Gallionellaceae	15	0	14	0	0	0
Bacteria	Proteobacteria	Deltaproteobacteria	Desulfuromonadales	Geobacteraceae	10	0	0	0	0	0
Eukaryota	Cnidaria	Hydrozoa	Hydroida	Hydridae	57	39	42	0	0	0
Bacteria	Proteobacteria	Betaproteobacteria	Hydrogenophilales	Hydrogenophilaceae	16	20	0	0	0	0
Bacteria	Proteobacteria	Alphaproteobacteria	Rhizobiales	Hyphomicrobiaceae	29	34	10	0	0	0
Bacteria	Proteobacteria	Alphaproteobacteria	Rhodobacterales	Hyphomonadaceae	13	14	0	0	0	0
Viruses	unclassified (derived from Viruses)	unclassified (derived from Viruses)	unclassified (derived from Viruses)	Inoviridae	0	0	0	11	0	0
Eukaryota	Arthropoda	Malacostraca	Amphipoda	Lysianassidae	21	0	10	0	0	0
Bacteria	Proteobacteria	Alphaproteobacteria	Rhizobiales	Methylobacteriaceae	16	12	0	0	0	0
Bacteria	Proteobacteria	Gammaproteobacteria	Methylococcales	Methylococcaceae	12	11	0	0	0	0
Bacteria	Proteobacteria	Betaproteobacteria	Methylophilales	Methylophilaceae	95	46	370	16	0	0
Viruses	unclassified (derived from Viruses)	unclassified (derived from Viruses)	unclassified (derived from Viruses)	Microviridae	819	695	435	654	492	541
Viruses	unclassified (derived from Viruses)	unclassified (derived from Viruses)	Caudovirales	Myoviridae	0	0	0	41	88	15379
Bacteria	Proteobacteria	Betaproteobacteria	Neisseriales	Neisseriaceae	16	15	22	0	0	0
Bacteria	Proteobacteria	Betaproteobacteria	Nitrosomonadales	Nitrosomonadaceae	11	23	10	0	0	0
Bacteria	Nitrospirae	Nitrospira (class)	Nitrospirales	Nitrospiraceae	48	49	12	0	0	0
Bacteria	Actinobacteria	Actinobacteria (class)	Actinomycetales	Nocardioidaceae	0	10	0	0	0	0
Bacteria	Proteobacteria	Betaproteobacteria	Burkholderiales	Oxalobacteraceae	31	22	23	0	0	0
Bacteria	Proteobacteria	Gammaproteobacteria	Pasteurellales	Pasteurellaceae	0	0	0	22	21	37
Bacteria	Proteobacteria	Alphaproteobacteria	Rhizobiales	Phyllobacteriaceae	56	59	16	0	0	0
Eukaryota	Chordata	Amphibia	Anura	Pipidae	0	10	0	0	0	0
Bacteria	Planctomycetes	Planctomycetacia	Planctomycetales	Planctomycetaceae	30	14	10	0	0	0
Viruses	unclassified (derived from Viruses)	unclassified (derived from Viruses)	Caudovirales	Podoviridae	0	0	0	164	0	14
Bacteria	Proteobacteria	Deltaproteobacteria	Myxococcales	Polyangiaceae	10	0	0	0	0	0
Bacteria	Bacteroidetes	Bacteroidia	Bacteroidales	Porphyromonadaceae	0	0	46	0	0	0
Bacteria	Bacteroidetes	Bacteroidia	Bacteroidales	Prevotellaceae	0	0	13	0	0	0
Bacteria	Proteobacteria	Gammaproteobacteria	Alteromonadales	Pseudoalteromonadaceae	0	0	0	0	0	11
Bacteria	Proteobacteria	Gammaproteobacteria	Pseudomonadales	Pseudomonadaceae	96	43	197	24	17	102
Bacteria	Proteobacteria	Alphaproteobacteria	Rhizobiales	Rhizobiaceae	60	62	39	0	0	0
Bacteria	Proteobacteria	Alphaproteobacteria	Rhodobacterales	Rhodobacteraceae	984	1243	525	18	23	13
Bacteria	Proteobacteria	Betaproteobacteria	Rhodocyclales	Rhodocyclaceae	52	52	68	0	0	0
Bacteria	Proteobacteria	Alphaproteobacteria	Rhodospirillales	Rhodospirillaceae	11	16	0	0	0	0
Bacteria	Proteobacteria	Gammaproteobacteria	Alteromonadales	Shewanellaceae	0	10	126	123	53	224
Viruses	unclassified (derived from Viruses)	unclassified (derived from Viruses)	Caudovirales	Siphoviridae	0	0	0	10	20	14
Bacteria	Proteobacteria	Alphaproteobacteria	Sphingomonadales	Sphingomonadaceae	507	561	280	0	25	17
Bacteria	Proteobacteria	Deltaproteobacteria	Syntrophobacterales	Syntrophaceae	0	0	13	0	0	0
Bacteria	Proteobacteria	Gammaproteobacteria	Vibrionales	Vibrionaceae	0	0	0	53192	40329	8965
Bacteria	Proteobacteria	Alphaproteobacteria	Rhizobiales	Xanthobacteraceae	22	18	0	0	0	0
Bacteria	Proteobacteria	Gammaproteobacteria	Xanthomonadales	Xanthomonadaceae	61	60	30	0	0	0
Bacteria	Proteobacteria	Betaproteobacteria	unclassified (derived from Betaproteobacteria)	unclassified (derived from Betaproteobacteria)	11	0	16	0	0	0
Bacteria	Proteobacteria	Betaproteobacteria	Burkholderiales	unclassified (derived from Burkholderiales)	245	257	167	0	0	0
Bacteria	Proteobacteria	Gammaproteobacteria	unclassified (derived from Gammaproteobacteria)	unclassified (derived from Gammaproteobacteria)	15	14	0	0	0	0
Bacteria	Proteobacteria	Alphaproteobacteria	Rhodobacterales	unclassified (derived from Rhodobacterales)	0	12	0	0	0	0
other sequences	unclassified (derived from other sequences)	unclassified (derived from other sequences)	unclassified (derived from other sequences)	unclassified (derived from other sequences)	0	0	0	14	38	76

Supplementary Information 2

Uncovering bacterial and functional diversity in macroinvertebrate mitochondrial-metagenomic datasets by differential centrifugation

Jan-Niklas Macher (JNM)[1], Arjen Speksnijder (AS)[1], Le Qin Choo (LQC)[1], Berry van der Hoorn (BvdH)[1], Willem Renema (WR)[1]*

[1] Naturalis Biodiversity Center, PO Box 9517, 2300 RA Leiden, Netherlands

[*] Corresponding author

Supporting Information 2: SEED functional categories (level 1, 2, 3, functions; hit counts for all samples ('Enriched 1-3'; Not enriched 1-3).

level1	level2	level3	function	Enriched 1	Enriched 2	Enriched 3	Not enriched 1	Not enriched 2	Not enriched 3
Carbohydrates	Aminosugars	(GlcNAc)2_Catabolic_Operon	(GlcNAc)2 ABC transporter, ATP-binding component 1	0	0	0	13	11	10
Carbohydrates	Aminosugars	(GlcNAc)2_Catabolic_Operon	(GlcNAc)2 ABC transporter, ATP-binding component 2	0	0	0	10	0	0
Carbohydrates	Aminosugars	(GlcNAc)2_Catabolic_Operon	(GlcNAc)2 ABC transporter, periplasmic substrate-binding protein	0	0	0	13	21	11
Carbohydrates	Aminosugars	(GlcNAc)2_Catabolic_Operon	(GlcNAc)2 ABC transporter, permease component 1	0	0	0	12	0	0
Carbohydrates	Aminosugars	(GlcNAc)2_Catabolic_Operon	(GlcNAc)2 ABC transporter, permease component 2	0	0	0	15	0	0
Carbohydrates	Di- and oligosaccharides	Trehalose_Biosynthesis	1,4-alpha-glucan (glycogen) branching enzyme, GH-13-type (EC 2.4.1.18)	0	0	0	77	38	26
Cofactors, Vitamins, Prosthetic Groups, Pigments	Quinone cofactors	Menaquinone_and_Phylloquinone_Biosynthesis	1,4-dihydroxy-2-naphthoate octaprenyltransferase (EC 2.5.1.-)	0	0	0	29	14	11
Fatty Acids, Lipids, and Isoprenoids	Phospholipids	Glycerolipid_and_Glycerophospholipid_Metabolism_in_Bacteria	1-acyl-sn-glycerol-3-phosphate acyltransferase (EC 2.3.1.51)	0	0	0	41	33	16
Clustering-based subsystems	Isoprenoid/cell wall biosynthesis: PREDICTED UNDECAPRENYL DIPHOSPHATE PHOSPHATASE	CBSS-83331.1.peg.3039	1-deoxy-D-xylulose 5-phosphate reductoisomerase (EC 1.1.1.267)	0	0	0	20	28	17
Cofactors, Vitamins, Prosthetic Groups, Pigments	Pyridoxine	Pyridoxin_(Vitamin_B6)_Biosynthesis	1-deoxy-D-xylulose 5-phosphate synthase (EC 2.2.1.7)	0	0	0	44	35	26
Clustering-based subsystems	Isoprenoid/cell wall biosynthesis: PREDICTED UNDECAPRENYL DIPHOSPHATE PHOSPHATASE	CBSS-83331.1.peg.3039	1-hydroxy-2-methyl-2-(E)-butenyl 4-diphosphate synthase (EC 1.17.7.1)	0	0	0	34	27	15
Carbohydrates	Monosaccharides	Fructose_utilization	1-phosphofructokinase (EC 2.7.1.56)	0	0	0	0	0	13
Virulence, Disease and Defense	Adhesion	Mediator_of_hyperadherence_YidE_in_Enterobacteria_and_its_conserved_region	16 kDa heat shock protein A	0	0	0	14	15	14
Nucleosides and Nucleotides	Pyrimidines	pyrimidine_conversions	2',3'-cyclic-nucleotide 2'-phosphodiesterase (EC 3.1.4.16)	0	0	0	45	42	14
Amino Acids and Derivatives	Lysine, threonine, methionine, and cysteine	Lysine_Biosynthesis_DAP_Pathway	2,3,4,5-tetrahydropyridine-2,6-dicarboxylate N-succinyltransferase (EC 2.3.1.117)	0	0	0	28	0	11
Carbohydrates	Central carbohydrate metabolism	Glycolysis_and_Gluconeogenesis	2,3-bisphosphoglycerate-independent phosphoglycerate mutase (EC 5.4.2.1)	0	0	0	29	34	25
Iron acquisition and metabolism	NULL	Iron_acquisition_in_Vibrio	2,3-dihydroxybenzoate-AMP ligase (EC 2.7.7.58)	0	0	0	12	0	0
Fatty Acids, Lipids, and Isoprenoids	Fatty acids	Fatty_acid_degradation_regulons	2,4-dienoyl-CoA reductase [NADPH] (EC 1.3.1.34)	0	0	0	58	44	16
Amino Acids and Derivatives	Aromatic amino acids and derivatives	Aromatic_amino_acid_degradation	2,4-dihydroxyhept-2-ene-1,7-dioic acid aldolase (EC 4.1.2.-)	0	0	0	25	10	0
Carbohydrates	Monosaccharides	D-gluconate_and_ketogluconates_metabolism	2,5-diketo-D-gluconate reductase A (EC 1.1.1.274)	0	0	0	10	0	0
Carbohydrates	Monosaccharides	D-gluconate_and_ketogluconates_metabolism	2,5-diketo-D-gluconic acid reductase B (EC 1.1.1.274)	0	0	0	10	10	0
Fatty Acids, Lipids, and Isoprenoids	Isoprenoids	polyprenyl_synthesis	2-C-methyl-D-erythritol 2,4-cyclodiphosphate synthase (EC 4.6.1.12)	0	0	0	10	12	0
Fatty Acids, Lipids, and Isoprenoids	Isoprenoids	polyprenyl_synthesis	2-C-methyl-D-erythritol 4-phosphate cytidylyltransferase (EC 2.7.7.60)	0	0	0	0	0	10
Cell Wall and Capsule	Gram-Negative cell wall components	KDO2-Lipid_A_biosynthesis	2-Keto-3-deoxy-D-manno-octulosonate-8-phosphate synthase (EC 2.5.1.55)	0	0	0	14	19	0
Amino Acids and Derivatives	Alanine, serine, and glycine	Glycine_Biosynthesis	2-amino-3-ketobutyrate coenzyme A ligase (EC 2.3.1.29)	0	0	0	32	22	16
Miscellaneous	Plant-Prokaryote DOE project	COG1565	2-amino-4-hydroxy-6-hydroxymethyldihydropteridine pyrophosphokinase (EC 2.7.6.3)	0	0	0	20	0	13
Phosphorus Metabolism	NULL	Phosphonate_metabolism	2-aminoethylphosphonate uptake and metabolism regulator	0	0	0	14	0	0
Phosphorus Metabolism	NULL	Phosphoenolpyruvate_phosphomutase	2-aminoethylphosphonate:pyruvate aminotransferase (EC 2.6.1.37)	0	0	0	21	0	0
Carbohydrates	Central carbohydrate metabolism	Entner-Doudoroff_Pathway	2-dehydro-3-deoxygluconate kinase (EC 2.7.1.45)	0	0	0	0	14	13
Carbohydrates	Monosaccharides	D-galactonate_catabolism	2-dehydro-3-deoxyphosphogalactonate aldolase (EC 4.1.2.21)	0	0	0	0	11	0
Carbohydrates	NULL	Sugar_utilization_in_Thermotogales	2-dehydro-3-deoxyphosphogluconate aldolase (EC 4.1.2.14)	0	0	0	26	27	0
Cofactors, Vitamins, Prosthetic Groups, Pigments	Coenzyme A	Coenzyme_A_Biosynthesis	2-dehydropantoate 2-reductase (EC 1.1.1.169)	0	0	0	10	12	11
Clustering-based subsystems	Hypothetical in Lysine biosynthetic cluster	CBSS-323850.3.peg.3269	2-haloalkanoic acid dehalogenase (EC 3.8.1.2)	0	0	0	0	0	12
Carbohydrates	Monosaccharides	D-galactarate,_D-glucarate_and_D-glycerate_catabolism	2-hydroxy-3-oxopropionate reductase (EC 1.1.1.60)	0	0	0	15	24	12
Secondary Metabolism	Aromatic amino acids and derivatives	Cinnamic_Acid_Degradation	2-hydroxy-6-ketonona-2,4-dienedioic acid hydrolase (EC 3.7.1.-)	0	0	0	0	0	12
Amino Acids and Derivatives	Branched-chain amino acids	Branched-Chain_Amino_Acid_Biosynthesis	2-isopropylmalate synthase (EC 2.3.3.13)	0	0	0	43	19	22
Amino Acids and Derivatives	Aromatic amino acids and derivatives	Common_Pathway_For_Synthesis_of_Aromatic_Compounds_(DAHP_synthase_to_chorismate)	2-keto-3-deoxy-D-arabino-heptulosonate-7-phosphate synthase I alpha (EC 2.5.1.54)	0	0	0	85	47	50
Secondary Metabolism	Aromatic amino acids and derivatives	Cinnamic_Acid_Degradation	2-keto-4-pentenoate hydratase (EC 4.2.1.-)	0	0	0	20	13	0
Carbohydrates	Monosaccharides	D-gluconate_and_ketogluconates_metabolism	2-ketoaldonate reductase, broad specificity (EC 1.1.1.215) (EC 1.1.1.-)	0	0	0	0	14	0
Carbohydrates	Organic acids	Methylcitrate_cycle	2-methylcitrate dehydratase (EC 4.2.1.79)	0	0	0	13	11	0
Carbohydrates	Organic acids	Methylcitrate_cycle	2-methylcitrate dehydratase FeS dependent (EC 4.2.1.79)	0	0	0	36	12	10
Carbohydrates	Organic acids	Methylcitrate_cycle	2-methylcitrate synthase (EC 2.3.3.5)	0	0	0	21	21	14
[illegible]	[illegible]	[illegible]	[illegible]	[illegible]	[illegible]	[illegible]	[illegible]	[illegible]	[illegible]

Supplementary Information 3

Uncovering bacterial and functional diversity in macroinvertebrate mitochondrial-metagenomic datasets by differential centrifugation

Jan-Niklas Macher (JNM)[1*], Arjen Speksnijder (AS)[1], Le Qin Choo (LQC)[1], Berry van der Hoorn (BvdH)[1], Willem Renema (WR)[1]
[1] Naturalis Biodiversity Center, PO Box 9517, 2300 RA Leiden, Netherlands
[*] Corresponding author

Supplementary Information 3: Abundance of taxa identified across the 'not enriched' samples using Kaiju

Hits	Domain	Phylum	Class	Order	Family	Genus	Species, strain or clade
59409	Bacteria	Proteobacteria	Gammaproteobacteria	Vibrionales	Vibrionaceae	Vibrio	
37902	Bacteria	Proteobacteria	Gammaproteobacteria	Enterobacterales	Enterobacteriaceae		
26949	Bacteria	Proteobacteria	Gammaproteobacteria	Aeromonadales	Aeromonadaceae	Aeromonas	
16581	Bacteria	Proteobacteria	Gammaproteobacteria	Vibrionales	Vibrionaceae	Vibrio	Vibrio mimicus
15993	Bacteria	Proteobacteria					
15615	Bacteria	Proteobacteria	Gammaproteobacteria	Enterobacterales	Enterobacteriaceae	Escherichia	
10666	Bacteria	Proteobacteria	Gammaproteobacteria	Enterobacterales	Enterobacteriaceae	Escherichia	Escherichia coli
10103	Bacteria						
10017	Bacteria	Proteobacteria	Gammaproteobacteria	Enterobacterales			
7618	dsDNA viruses, no RNA stage	Caudovirales	Myoviridae				
5106	/						
4842	Bacteria	Proteobacteria	Gammaproteobacteria	Vibrionales	Vibrionaceae	Vibrio	Vibrio cholerae
3570	Bacteria	Proteobacteria	Gammaproteobacteria				
3399	Bacteria	Proteobacteria	Gammaproteobacteria	Aeromonadales	Aeromonadaceae	Aeromonas	Aeromonas veronii
2982	/						
2591	Bacteria	Proteobacteria	Gammaproteobacteria	Enterobacterales	Enterobacteriaceae	Citrobacter	
2374	Bacteria	Proteobacteria	Gammaproteobacteria	Enterobacterales	Enterobacteriaceae	Shigella	Shigella sonnei
1479	Bacteria	Proteobacteria	Gammaproteobacteria	Enterobacterales	Enterobacteriaceae	Klebsiella	Klebsiella pneumoniae
1340	Bacteria	Proteobacteria	Gammaproteobacteria	Aeromonadales	Aeromonadaceae	Aeromonas	Aeromonas hydrophila
805	Bacteria	Terrabacteria group	Firmicutes				
796	Bacteria	Proteobacteria	Gammaproteobacteria	Enterobacterales	Enterobacteriaceae	Citrobacter	Citrobacter freundii complex
749	dsDNA viruses, no RNA stage	Caudovirales	Myoviridae	Ounavirinae	Felixo1virus	Salmonella virus FelixO1	Salmonella phage FelixO1
718	Bacteria	Proteobacteria	Gammaproteobacteria	Enterobacterales	Enterobacteriaceae	Klebsiella	
689	Bacteria	Proteobacteria	Gammaproteobacteria	Enterobacterales	Enterobacteriaceae	Citrobacter	Citrobacter freundii complex
667	dsDNA viruses, no RNA stage	Caudovirales	Myoviridae	Ounavirinae	Felixo1virus		
642	/						
547	Bacteria	Proteobacteria	Gammaproteobacteria	Vibrionales	Vibrionaceae	Vibrio	Vibrio metoecus
515	Bacteria	Proteobacteria	Gammaproteobacteria	Vibrionales	Vibrionaceae		
494	Bacteria	Proteobacteria	Gammaproteobacteria	Aeromonadales	Aeromonadaceae	Aeromonas	Aeromonas media
494	dsDNA viruses, no RNA stage	Caudovirales	Myoviridae	unclassified Myoviridae			
489	Bacteria	Proteobacteria	Gammaproteobacteria	Enterobacterales	unclassified Enterobacterales	Plesiomonas	
487	Bacteria	Proteobacteria	Gammaproteobacteria	Aeromonadales	Aeromonadaceae	Aeromonas	Aeromonas allosaccharophila
483	dsDNA viruses, no RNA stage	Caudovirales	Myoviridae	Ounavirinae	Felixo1virus	Escherichia virus HY02	Escherichia phage HY02
478	dsDNA viruses, no RNA stage	Caudovirales	Myoviridae	Ounavirinae	Felixo1virus	Escherichia virus AYO145A	Escherichia phage vB_EcoM_AYO145A
465	dsDNA viruses, no RNA stage	Caudovirales	Myoviridae	Ounavirinae	Felixo1virus	Escherichia virus EC6	Escherichia phage EC6
429	Bacteria	Proteobacteria	Gammaproteobacteria	Enterobacterales	Enterobacteriaceae	Salmonella	Salmonella enterica
424	Bacteria	Proteobacteria	Gammaproteobacteria	Aeromonadales	Aeromonadaceae	Aeromonas	Aeromonas caviae
407	Bacteria	Proteobacteria	Gammaproteobacteria	Vibrionales	Vibrionaceae	Vibrio	Vibrio mimicus
317	Bacteria	Proteobacteria	delta/epsilon subdivisions	Deltaproteobacteria	Desulfovibrionales	Desulfovibrionaceae	Desulfovibrio
306	Bacteria	Proteobacteria	Gammaproteobacteria	Vibrionales	Vibrionaceae	Vibrio	Vibrio harveyi group
296	Bacteria	Proteobacteria	Gammaproteobacteria	Enterobacterales	Enterobacteriaceae	Citrobacter	Citrobacter freundii complex
293	dsDNA viruses, no RNA stage	Caudovirales	Myoviridae	Ounavirinae	Felixo1virus	Escherichia virus JH2	Escherichia phage JH2
285	Bacteria	Proteobacteria	Gammaproteobacteria	Aeromonadales	Aeromonadaceae	Aeromonas	Aeromonas salmonicida
284	Bacteria	Proteobacteria	Gammaproteobacteria	Vibrionales	Vibrionaceae	Vibrio	Vibrio mimicus
269	Bacteria	Proteobacteria	Gammaproteobacteria	Enterobacterales	Enterobacteriaceae	Escherichia	Escherichia coli
268	dsDNA viruses, no RNA stage	Caudovirales	Myoviridae	unclassified Myoviridae	Escherichia phage vB_EcoM_Alf5		
258	dsDNA viruses, no RNA stage	Caudovirales	Myoviridae	Ounavirinae	Felixo1virus	Salmonella virus UAB87	Enterobacteriaphage UAB_Phi87
237	Bacteria	Proteobacteria	Gammaproteobacteria	Enterobacterales	Enterobacteriaceae	Escherichia	Escherichia coli
231	dsDNA viruses, no RNA stage	Caudovirales	Myoviridae	Ounavirinae	Felixo1virus	Salmonella virus HB2014	Salmonella phage HB-2014
229	Bacteria	Proteobacteria	Gammaproteobacteria	Enterobacterales	Enterobacteriaceae	Escherichia	Escherichia sp. KTE159
223	Bacteria	Proteobacteria	Gammaproteobacteria	Vibrionales	Vibrionaceae	Vibrio	Vibrio mimicus
218	Bacteria	Proteobacteria	Gammaproteobacteria	Enterobacterales	Enterobacteriaceae	Klebsiella	Klebsiella pneumoniae
217	Bacteria	Proteobacteria	Gammaproteobacteria	Vibrionales	Vibrionaceae	Vibrio	Vibrio sp. RC586
207	dsDNA viruses, no RNA stage	Caudovirales	Myoviridae	unclassified Myoviridae	Salmonella phage BPS15Q2		
201	Bacteria	Proteobacteria	Gammaproteobacteria	Vibrionales	Vibrionaceae	Vibrio	Vibrio vulnificus
174	Bacteria	Terrabacteria group	Firmicutes	Clostridia	Clostridiales	Clostridiaceae	Clostridium
173	Bacteria	Terrabacteria group	Firmicutes	Bacilli	Lactobacillales	Enterococcaceae	Enterococcus
173	dsDNA viruses, no RNA stage	Caudovirales	Myoviridae	unclassified Myoviridae	Salmonella phage FSL SP-107		
171	Bacteria	Proteobacteria	Gammaproteobacteria	Enterobacterales	Enterobacteriaceae	Escherichia	Escherichia coli
171	dsDNA viruses, no RNA stage	Caudovirales	Myoviridae	Ounavirinae	Suspvirus		
169	Bacteria	Proteobacteria	Gammaproteobacteria	Enterobacterales	Enterobacteriaceae	Escherichia	Escherichia sp. TW09231
167	Bacteria	Proteobacteria	Gammaproteobacteria	Enterobacterales	Enterobacteriaceae	Shigella	Shigella flexneri
162	Bacteria	Proteobacteria	Gammaproteobacteria	Enterobacterales	unclassified Enterobacterales	Plesiomonas	Plesiomonas shigelloides
148	Bacteria	Terrabacteria group	Firmicutes	Bacilli	Bacillales	Bacillaceae	Bacillus
146	dsDNA viruses, no RNA stage	Caudovirales	Myoviridae	Ounavirinae	Felixo1virus	Salmonella virus FelixO1	
145	Bacteria	Proteobacteria	Gammaproteobacteria	Enterobacterales	Enterobacteriaceae	Enterobacter	Enterobacter cloacae complex
138	Bacteria	Proteobacteria	Gammaproteobacteria	Aeromonadales	Aeromonadaceae	Aeromonas	Aeromonas sp. ZOR0001
137	Bacteria	Proteobacteria	Betaproteobacteria	Burkholderiales	Burkholderiaceae	Ralstonia	Ralstonia pickettii
135	Bacteria	Proteobacteria	Gammaproteobacteria	Pseudomonadales	Pseudomonadaceae	Pseudomonas	Pseudomonas aeruginosa group
134	Bacteria	Terrabacteria group	Firmicutes	Bacilli	Lactobacillales	Enterococcaceae	Enterococcus
131	Bacteria	Proteobacteria	Gammaproteobacteria	Aeromonadales	Aeromonadaceae	Aeromonas	Aeromonas veronii
126	Bacteria	environmental samples	uncultured bacterium				
122	Bacteria	Proteobacteria	Gammaproteobacteria	Enterobacterales	Enterobacteriaceae	Citrobacter	Citrobacter freundii complex
121	Bacteria	Proteobacteria	Gammaproteobacteria	Vibrionales			
116	Bacteria	FCB group	Bacteroidetes/Chlorobi group	Bacteroidetes	Bacteroidia	Bacteroidales	Rikenellaceae
115	dsDNA viruses, no RNA stage	Caudovirales	Myoviridae	Ounavirinae	Felixo1virus	Escherichia virus wV8	Escherichia phage wV8
113	Bacteria	Proteobacteria	Betaproteobacteria	Burkholderiales	Burkholderiaceae	Ralstonia	
112	Bacteria	Proteobacteria	Gammaproteobacteria	Enterobacterales	Enterobacteriaceae	Enterobacter	
110	Bacteria	Proteobacteria	Alphaproteobacteria	Rhizobiales	Bradyrhizobiaceae	Bradyrhizobium	Bradyrhizobium sp. BTAi1
106	Bacteria	Terrabacteria group	Firmicutes	Bacilli	Bacillales	Bacillaceae	Bacillus
106	Bacteria	Proteobacteria	Gammaproteobacteria	Enterobacterales	Erwiniaceae	Pantoea	Pantoea ananatis
104	Eukaryota	Opisthokonta	Fungi	Dikarya	Ascomycota	saccharomyceta	Saccharomycotina
100	Bacteria	Proteobacteria	Gammaproteobacteria	Vibrionales	Vibrionaceae	Vibrio	Vibrio mimicus
99	Bacteria	Proteobacteria	Gammaproteobacteria	Enterobacterales	Enterobacteriaceae	Salmonella	Salmonella enterica
99	Bacteria	Proteobacteria	Gammaproteobacteria	Enterobacterales	Enterobacteriaceae	Escherichia	Escherichia coli
97	Bacteria	Proteobacteria	Gammaproteobacteria	Aeromonadales	Aeromonadaceae	Aeromonas	Aeromonas jandaei
97	dsDNA viruses, no RNA stage	Caudovirales	Myoviridae	Ounavirinae	Felixo1virus	Salmonella virus Mushroom	Salmonella phage Mushroom
95	Bacteria	Proteobacteria	Gammaproteobacteria	Enterobacterales	Enterobacteriaceae	Enterobacter	Enterobacter cloacae complex
95	Bacteria	Proteobacteria	Gammaproteobacteria	Aeromonadales	Aeromonadaceae	Aeromonas	Aeromonas sp. DNP9
94	Bacteria	Proteobacteria	Gammaproteobacteria	Aeromonadales	Aeromonadaceae	Aeromonas	Aeromonas sp. YN13HZO-058
89	Bacteria	Proteobacteria	Gammaproteobacteria	Enterobacterales	Enterobacteriaceae	Escherichia	Escherichia coli
89	Bacteria	Proteobacteria	Gammaproteobacteria	Enterobacterales	Enterobacteriaceae	Citrobacter	Citrobacter sp. S-77
87	Bacteria	Terrabacteria group	Cyanobacteria/Melainabacteria group	Cyanobacteria	Nostocales	Aphanizomenonaceae	Aphanizomenon
87	Bacteria	Proteobacteria	Gammaproteobacteria	Vibrionales	Vibrionaceae	Vibrio	Vibrio mimicus
84	Bacteria	Proteobacteria	Gammaproteobacteria	Enterobacterales	Enterobacteriaceae	Citrobacter	Citrobacter freundii complex
83	Bacteria	FCB group	Bacteroidetes/Chlorobi group	Bacteroidetes	Bacteroidia	Bacteroidales	Porphyromonadaceae
82	Bacteria	Proteobacteria	Gammaproteobacteria	Enterobacterales	Enterobacteriaceae	Escherichia	Escherichia albertii
82	Bacteria	Proteobacteria	Betaproteobacteria	Burkholderiales	Burkholderiaceae		
79	Bacteria	Proteobacteria	Gammaproteobacteria	Enterobacterales	Enterobacteriaceae	Citrobacter	Citrobacter freundii complex
78	Eukaryota	Opisthokonta	Fungi	Neocallimastigomycota	Neocallimastigomycetes	Neocallimastigales	Neocallimastigaceae
78	Bacteria	Proteobacteria	Gammaproteobacteria	Enterobacterales	Enterobacteriaceae	Shigella	Shigella dysenteriae
78	Bacteria	Proteobacteria	Gammaproteobacteria	Enterobacterales	Enterobacteriaceae	Escherichia	Escherichia coli
78	Bacteria	Proteobacteria	Gammaproteobacteria	Aeromonadales	Aeromonadaceae	Aeromonas	Aeromonas australiensis
78	dsDNA viruses, no RNA stage	Caudovirales	Myoviridae	Ounavirinae	Felixo1virus	Escherichia virus VpaE1	Escherichia phage vB_EcoM-VpaE1
73	Bacteria	Proteobacteria	Gammaproteobacteria	Enterobacterales	Enterobacteriaceae	Klebsiella	Klebsiella variicola
72	Bacteria	Proteobacteria	Gammaproteobacteria	Pseudomonadales	Pseudomonadaceae	Pseudomonas	
72	Bacteria	Proteobacteria	Gammaproteobacteria	Enterobacterales	Enterobacteriaceae	Shigella	
72	Bacteria	Proteobacteria	Gammaproteobacteria	Aeromonadales	Aeromonadaceae	Aeromonas	Aeromonas finlandiensis
71	Bacteria	Proteobacteria	Gammaproteobacteria	Vibrionales	Vibrionaceae	Vibrio	Vibrio mimicus
71	Bacteria	Proteobacteria	Gammaproteobacteria	Vibrionales	Vibrionaceae	Vibrio	Vibrio mimicus
70	Bacteria	FCB group	Bacteroidetes/Chlorobi group	Bacteroidetes	unclassified Bacteroidetes	unclassified Bacteroidetes (miscellaneous)	
69	Bacteria	Proteobacteria	Gammaproteobacteria	Enterobacterales	Enterobacteriaceae	Citrobacter	Citrobacter koseri
68	Bacteria	Proteobacteria	Gammaproteobacteria	Pasteurellales	Pasteurellaceae	Haemophilus	Haemophilus influenzae
68	Bacteria	Proteobacteria	Gammaproteobacteria	Enterobacterales	Enterobacteriaceae	Escherichia	Escherichia sp. TW09308
68	Bacteria	Proteobacteria	Gammaproteobacteria	Aeromonadales	Aeromonadaceae	Aeromonas	Aeromonas encheleia
67	Bacteria	Proteobacteria	Gammaproteobacteria	Vibrionales	Vibrionaceae	Vibrio	Vibrio harveyi group
67	Bacteria	Proteobacteria	Gammaproteobacteria	Enterobacterales	Enterobacteriaceae	Klebsiella	Klebsiella pneumoniae
67	Bacteria	Proteobacteria	Gammaproteobacteria	Aeromonadales	Aeromonadaceae	Aeromonas	Aeromonas hydrophila
66	Bacteria	Proteobacteria	Gammaproteobacteria	Enterobacterales	Enterobacteriaceae	Escherichia	Escherichia coli
66	Bacteria	Proteobacteria	Gammaproteobacteria	Aeromonadales	Aeromonadaceae	Aeromonas	Aeromonas bivalvium
66	dsDNA viruses, no RNA stage	Caudovirales	Myoviridae	unclassified Myoviridae	Salmonella phage SPT-1		
65	Bacteria	Proteobacteria	Gammaproteobacteria	Alteromonadales	Shewanellaceae	Shewanella	
65	Bacteria	Proteobacteria	Gammaproteobacteria	Aeromonadales	Aeromonadaceae	Aeromonas	Aeromonas enteropelogenes
64	Bacteria	Proteobacteria	Gammaproteobacteria	Enterobacterales	Enterobacteriaceae	Salmonella	
64	Bacteria	Proteobacteria	Gammaproteobacteria	Enterobacterales	Enterobacteriaceae	Escherichia	Escherichia coli
64	Bacteria	Proteobacteria	Gammaproteobacteria	Aeromonadales	Aeromonadaceae	Aeromonas	Aeromonas rivuli
64	dsDNA viruses, no RNA stage	Caudovirales	Myoviridae	Ounavirinae	Suspvirus	Escherichia virus SUSP2	Escherichia phage SUSP2
64	dsDNA viruses, no RNA stage	Caudovirales	Myoviridae	Ounavirinae	Felixo1virus	unclassified FelixO1likevirus	Salmonella phage Si3
63	Bacteria	Proteobacteria	Gammaproteobacteria	Vibrionales	Vibrionaceae	Vibrio	Vibrio xuii
63	Bacteria	Proteobacteria	Gammaproteobacteria	Aeromonadales	Aeromonadaceae	Aeromonas	Aeromonas veronii
62	Bacteria	Proteobacteria	Gammaproteobacteria	Enterobacterales	Enterobacteriaceae	Kluyvera	Kluyvera intermedia
62	Bacteria	Proteobacteria	Gammaproteobacteria	Aeromonadales	Aeromonadaceae	Aeromonas	Aeromonas sobria
60	Bacteria	Proteobacteria	Gammaproteobacteria	Vibrionales	Vibrionaceae	Vibrio	Vibrio mimicus
60	Bacteria	Proteobacteria	Gammaproteobacteria	Enterobacterales	Enterobacteriaceae	Escherichia	Escherichia coli
60	Bacteria	Proteobacteria	Gammaproteobacteria	Enterobacterales	Enterobacteriaceae	Escherichia	Escherichia coli
58	Eukaryota	Opisthokonta	Fungi	Dikarya	Ascomycota	saccharomyceta	Pezizomycotina
58	Bacteria	Proteobacteria	Gammaproteobacteria	Vibrionales	Vibrionaceae	Vibrio	Vibrio sp. RC341
57	Bacteria	Proteobacteria	Gammaproteobacteria	Aeromonadales	Aeromonadaceae	Aeromonas	Aeromonas salmonicida
55	Bacteria	Proteobacteria	Gammaproteobacteria	Vibrionales	Vibrionaceae	Vibrio	Vibrio vulnificus
55	Bacteria	Proteobacteria	Gammaproteobacteria	Enterobacterales	Enterobacteriaceae	Citrobacter	Citrobacter amalonaticus
54	Bacteria	Proteobacteria	Gammaproteobacteria	Aeromonadales	Aeromonadaceae	Aeromonas	Aeromonas schubertii
53	Bacteria	Proteobacteria	Gammaproteobacteria	Enterobacterales	Enterobacteriaceae	Shigella	Shigella dysenteriae
53	Bacteria	Proteobacteria	Gammaproteobacteria	Enterobacterales	Enterobacteriaceae	Escherichia	Escherichia coli
53	dsDNA viruses, no RNA stage	Caudovirales	Myoviridae	unclassified Myoviridae	Staphylococcus phage SA1		
52	Bacteria	Proteobacteria	Gammaproteobacteria	Enterobacterales	Enterobacteriaceae	Escherichia	Escherichia coli
52	Bacteria	Proteobacteria	Alphaproteobacteria				
51	Bacteria	Proteobacteria	Gammaproteobacteria	Enterobacterales	Enterobacteriaceae	Citrobacter	Citrobacter sp. KTE32
51	Bacteria	Proteobacteria	Gammaproteobacteria	Aeromonadales	Aeromonadaceae	Aeromonas	Aeromonas sp. 159
50	Bacteria	FCB group	Bacteroidetes/Chlorobi group	Bacteroidetes	Bacteroidia	Bacteroidales	Bacteroidaceae
48	Bacteria	FCB group	Bacteroidetes/Chlorobi group	Bacteroidetes	Bacteroidia	Bacteroidales	
47	Bacteria	Proteobacteria	Gammaproteobacteria	Enterobacterales	Enterobacteriaceae	Escherichia	Escherichia sp. TW14182
47	Bacteria	Proteobacteria	Gammaproteobacteria	Enterobacterales	Enterobacteriaceae	Escherichia	Escherichia fergusonii
47	Bacteria	Proteobacteria	Gammaproteobacteria	Enterobacterales	Enterobacteriaceae	Escherichia	Escherichia coli
46	Bacteria	Proteobacteria	Gammaproteobacteria	Enterobacterales	Enterobacteriaceae	Citrobacter	Citrobacter koseri
45	Bacteria	Terrabacteria group	Firmicutes	Clostridia	Clostridiales	Peptostreptococcaceae	Romboutsia
45	Bacteria	Terrabacteria group	Actinobacteria	Actinobacteria	Corynebacteriales	Corynebacteriaceae	Corynebacterium
45	Bacteria	Proteobacteria	Gammaproteobacteria	Vibrionales	Vibrionaceae	Vibrio	Vibrio harveyi group
45	Bacteria	Proteobacteria	Gammaproteobacteria	Enterobacterales	Enterobacteriaceae	Salmonella	Salmonella enterica
45	Bacteria	Proteobacteria	Gammaproteobacteria	Aeromonadales	Aeromonadaceae	Aeromonas	Aeromonas fluvialis
44	Bacteria	Proteobacteria	Gammaproteobacteria	Enterobacterales	unclassified Enterobacterales	Plesiomonas	Plesiomonas sp. ZOR0011
44	Bacteria	Proteobacteria	Gammaproteobacteria	Enterobacterales	Enterobacteriaceae	Klebsiella	Klebsiella sp. MS 92-3
44	Bacteria	Proteobacteria	Gammaproteobacteria	Aeromonadales	Aeromonadaceae	Aeromonas	Aeromonas sp. EERV15
44	Bacteria	Proteobacteria	Gammaproteobacteria	Aeromonadales	Aeromonadaceae	Aeromonas	Aeromonas sanarellii
44	Bacteria	Proteobacteria	Gammaproteobacteria	Aeromonadales	Aeromonadaceae		
43	Bacteria	Terrabacteria group	Firmicutes	Clostridia	Clostridiales		
43	Bacteria	Proteobacteria	Gammaproteobacteria	Aeromonadales	Aeromonadaceae	Aeromonas	Aeromonas aquatica
43	Bacteria	Proteobacteria	Alphaproteobacteria	Rhizobiales	Bradyrhizobiaceae	Bradyrhizobium	
42	Bacteria	Proteobacteria	Alphaproteobacteria	Sphingomonadales	Sphingomonadaceae	Sphingomonas	Sphingomonas histidinilytica
42	Bacteria	Proteobacteria	Alphaproteobacteria	Sphingomonadales	Sphingomonadaceae	Novosphingobium	
41	Bacteria	Proteobacteria	Gammaproteobacteria	Enterobacterales	Enterobacteriaceae	Shigella	Shigella flexneri
40	Bacteria	Proteobacteria	Gammaproteobacteria	Vibrionales	Vibrionaceae	Vibrio	Vibrio cholerae
40	Bacteria	Proteobacteria	Gammaproteobacteria	Enterobacterales	Enterobacteriaceae	Escherichia	Escherichia coli
40	Bacteria	Proteobacteria	Gammaproteobacteria	Aeromonadales	Aeromonadaceae	Aeromonas	Aeromonas media
39	Bacteria	Proteobacteria	Gammaproteobacteria	Enterobacterales	Enterobacteriaceae	Escherichia	Escherichia coli
39	Bacteria	PVC group	Chlamydiae	Chlamydiia	Chlamydiales	Chlamydiaceae	Chlamydia/Chlamydophila group
39	unclassified bacterial viruses	Salmonella phage SBA-1781					
39	dsDNA viruses, no RNA stage	Caudovirales	Myoviridae	unclassified Myoviridae	Citrobacter phage Mijalis		
38	Bacteria	Proteobacteria	Gammaproteobacteria	Enterobacterales	Enterobacteriaceae	Klebsiella	Klebsiella aerogenes
38	Bacteria	Proteobacteria	Gammaproteobacteria	Aeromonadales	Aeromonadaceae	Aeromonas	Aeromonas lacus
37	Bacteria	Proteobacteria	Gammaproteobacteria	Enterobacterales	Enterobacteriaceae	Escherichia	Escherichia coli
37	Bacteria	Proteobacteria	Gammaproteobacteria	Enterobacterales	Enterobacteriaceae	Enterobacter	Enterobacter cloacae complex
37	Bacteria	Proteobacteria	Gammaproteobacteria	Aeromonadales	Aeromonadaceae	Aeromonas	Aeromonas piscicola
36	Bacteria	Proteobacteria	Gammaproteobacteria	Enterobacterales	Enterobacteriaceae	Salmonella	Salmonella enterica
36	Bacteria	Proteobacteria	Gammaproteobacteria	Enterobacterales	Enterobacteriaceae	Escherichia	Escherichia coli
36	Bacteria	Proteobacteria	Gammaproteobacteria	Enterobacterales	Enterobacteriaceae	Citrobacter	Citrobacter freundii complex
36	Bacteria	Proteobacteria	Gammaproteobacteria	Enterobacterales	Enterobacteriaceae	Citrobacter	Citrobacter freundii complex
36	Bacteria	Proteobacteria	Gammaproteobacteria	Aeromonadales	Aeromonadaceae	Aeromonas	Aeromonas taiwanensis
36	Bacteria	Proteobacteria	Betaproteobacteria	Burkholderiales			
36	Bacteria	FCB group	Bacteroidetes/Chlorobi group	Bacteroidetes			
34	Bacteria	Proteobacteria	Gammaproteobacteria	Vibrionales	Vibrionaceae	Vibrio	Vibrio cholerae
34	Bacteria	Proteobacteria	Gammaproteobacteria	Aeromonadales	Aeromonadaceae	Aeromonas	Aeromonas sp. L_1B5_3
34	Bacteria	FCB group	Bacteroidetes/Chlorobi group	Bacteroidetes	Sphingobacteriia	Sphingobacteriales	Sphingobacteriaceae
34	Bacteria	FCB group	Bacteroidetes/Chlorobi group	Bacteroidetes	Bacteroidia	Bacteroidales	Porphyromonadaceae
34	Bacteria	FCB group	Bacteroidetes/Chlorobi group	Bacteroidetes	Bacteroidia	Bacteroidales	Porphyromonadaceae
33	Bacteria	Proteobacteria	Gammaproteobacteria	Vibrionales	Vibrionaceae	Vibrio	Vibrio cholerae
33	Bacteria	Proteobacteria	Gammaproteobacteria	Enterobacterales	Enterobacteriaceae	Salmonella	Salmonella enterica
33	Bacteria	Proteobacteria	Gammaproteobacteria	Enterobacterales	Enterobacteriaceae	Klebsiella	Klebsiella pneumoniae
32	Bacteria	Proteobacteria	delta/epsilon subdivisions	Deltaproteobacteria	Desulfovibrionales	Desulfovibrionaceae	Desulfovibrio
32	Bacteria	Proteobacteria	Gammaproteobacteria	Enterobacterales	Enterobacteriaceae	Shigella	Shigella boydii
32	Bacteria	Proteobacteria	Gammaproteobacteria	Enterobacterales	Enterobacteriaceae	Klebsiella	Klebsiella oxytoca
32	Bacteria	Proteobacteria	Gammaproteobacteria	Enterobacterales	Enterobacteriaceae	Escherichia	Escherichia sp. B1147
32	Bacteria	Proteobacteria	Gammaproteobacteria	Enterobacterales	Enterobacteriaceae	Escherichia	Escherichia albertii
32	Bacteria	Proteobacteria	Gammaproteobacteria	Aeromonadales	Aeromonadaceae	Aeromonas	Aeromonas sp. SCS5
31	Bacteria	Proteobacteria	Gammaproteobacteria	Vibrionales	Vibrionaceae	Vibrio	Vibrio cholerae
31	Bacteria	Proteobacteria	Gammaproteobacteria	Pseudomonadales	Moraxellaceae	Psychrobacter	Psychrobacter sp. AntiMn-1
30	Bacteria	FCB group	Bacteroidetes/Chlorobi group	Bacteroidetes	Bacteroidia	Bacteroidales	Porphyromonadaceae
29	Bacteria	Proteobacteria	delta/epsilon subdivisions	Deltaproteobacteria	Desulfovibrionales	Desulfovibrionaceae	Desulfovibrio
29	Bacteria	Proteobacteria	Gammaproteobacteria	Enterobacterales	Enterobacteriaceae	Klebsiella	Klebsiella pneumoniae
29	Bacteria	Proteobacteria	Gammaproteobacteria	Enterobacterales	Enterobacteriaceae	Klebsiella	Klebsiella pneumoniae
29	Bacteria	Proteobacteria	Gammaproteobacteria	Enterobacterales	Enterobacteriaceae	Citrobacter	Citrobacter pasteurii
29	Bacteria	Proteobacteria	Gammaproteobacteria	Enterobacterales	Enterobacteriaceae	Citrobacter	Citrobacter freundii complex
29	Bacteria	Proteobacteria	Gammaproteobacteria	Enterobacterales	Enterobacteriaceae	Citrobacter	Citrobacter europaeus
29	Bacteria	Proteobacteria	Gammaproteobacteria	Aeromonadales	Aeromonadaceae	Aeromonas	Aeromonas veronii
29	Bacteria	Proteobacteria	Gammaproteobacteria	Aeromonadales	Aeromonadaceae	Aeromonas	Aeromonas popoffii
29	Bacteria	Proteobacteria	Gammaproteobacteria	Aeromonadales	Aeromonadaceae	Aeromonas	Aeromonas dhakensis
28	Bacteria	Terrabacteria group	Tenericutes	Mollicutes	Acholeplasmatales	Acholeplasmataceae	Candidatus Phytoplasma
28	Bacteria	Proteobacteria	Gammaproteobacteria	Enterobacterales	Enterobacteriaceae	Escherichia	Escherichia coli
28	Bacteria	Proteobacteria	Gammaproteobacteria	Enterobacterales	Enterobacteriaceae	Enterobacter	Enterobacter cloacae complex
28	Bacteria	Proteobacteria	Gammaproteobacteria	Enterobacterales	Enterobacteriaceae	Enterobacter	Enterobacter cloacae complex
28	Bacteria	Proteobacteria	Gammaproteobacteria	Enterobacterales	Enterobacteriaceae	Citrobacter	Citrobacter freundii complex
28	Bacteria	Proteobacteria	Gammaproteobacteria	Aeromonadales	Aeromonadaceae	Aeromonas	Aeromonas tecta
28	Bacteria	Proteobacteria	Alphaproteobacteria	Rhizobiales			
27	Bacteria	Proteobacteria	Gammaproteobacteria	Vibrionales	Vibrionaceae	Vibrio	Vibrio harveyi group
27	Bacteria	Proteobacteria	Gammaproteobacteria	Vibrionales	Vibrionaceae	Vibrio	Vibrio harveyi group
27	Bacteria	Proteobacteria	Gammaproteobacteria	Enterobacterales	Enterobacteriaceae	Escherichia	Escherichia coli
27	Bacteria	Proteobacteria	Gammaproteobacteria	Enterobacterales	Enterobacteriaceae	Citrobacter	Citrobacter freundii complex
27	Bacteria	Proteobacteria	Gammaproteobacteria	Aeromonadales	Aeromonadaceae	Aeromonas	Aeromonas veronii
27	Bacteria	Proteobacteria	Gammaproteobacteria	Aeromonadales	Aeromonadaceae	Aeromonas	Aeromonas sp. RU39B
27	Bacteria	Proteobacteria	Gammaproteobacteria	Aeromonadales	Aeromonadaceae	Aeromonas	Aeromonas sp. 62-46
27	Bacteria	Proteobacteria	Alphaproteobacteria	Rhodobacterales	Rhodobacteraceae		
27	Bacteria	FCB group	Bacteroidetes/Chlorobi group	Bacteroidetes	Bacteroidia	Bacteroidales	Prevotellaceae
26	Bacteria	Terrabacteria group	Firmicutes	Bacilli			
26	Bacteria	Proteobacteria	Gammaproteobacteria	Vibrionales	Vibrionaceae	Vibrio	Vibrio anguillarum
26	Bacteria	Proteobacteria	Gammaproteobacteria	Enterobacterales	Enterobacteriaceae	Klebsiella	Klebsiella pneumoniae
26	Bacteria	Proteobacteria	Gammaproteobacteria	Enterobacterales	Enterobacteriaceae	Escherichia	Escherichia coli
26	Bacteria	Proteobacteria	Gammaproteobacteria	Enterobacterales	Enterobacteriaceae	Citrobacter	Citrobacter sp. 30_2
26	Bacteria	Proteobacteria	Gammaproteobacteria	Aeromonadales	Aeromonadaceae	Aeromonas	Aeromonas veronii
26	Bacteria	Proteobacteria	Gammaproteobacteria	Aeromonadales	Aeromonadaceae	Aeromonas	Aeromonas molluscorum
26	Bacteria	Proteobacteria	Alphaproteobacteria	Sphingomonadales	Sphingomonadaceae	Sphingopyxis	
26	Bacteria	Nitrospirae	Nitrospira	Nitrospirales	Nitrospiraceae	Nitrospira	Candidatus Nitrospira nitrosa
25	Bacteria	Proteobacteria	Gammaproteobacteria	Enterobacterales	Enterobacteriaceae	Klebsiella	Klebsiella pneumoniae
25	Bacteria	Proteobacteria	Gammaproteobacteria	Enterobacterales	Enterobacteriaceae	Escherichia	Escherichia coli
25	Bacteria	Proteobacteria	Gammaproteobacteria	Enterobacterales	Enterobacteriaceae	Enterobacter	Enterobacter mori
25	Bacteria	Proteobacteria	Gammaproteobacteria	Aeromonadales	Aeromonadaceae	Aeromonas	Aeromonas sp. ZOR0002
24	Bacteria	Proteobacteria	Gammaproteobacteria	Vibrionales	Vibrionaceae	Vibrio	Vibrio harveyi group
24	Bacteria	Proteobacteria	Gammaproteobacteria	Vibrionales	Vibrionaceae	Vibrio	Vibrio cholerae
24	Bacteria	Proteobacteria	Gammaproteobacteria	Enterobacterales	Enterobacteriaceae	Escherichia	Escherichia coli
24	Bacteria	Proteobacteria	Gammaproteobacteria	Enterobacterales	Enterobacteriaceae	Enterobacter	Enterobacter sp. FY-07
23	Bacteria	Proteobacteria	Gammaproteobacteria	Enterobacterales	Enterobacteriaceae	Leclercia	Leclercia adecarboxylata
23	Bacteria	Proteobacteria	Gammaproteobacteria	Enterobacterales	Enterobacteriaceae	Citrobacter	Citrobacter rodentium
23	Bacteria	Proteobacteria	Gammaproteobacteria	Aeromonadales	Aeromonadaceae	Aeromonas	Aeromonas hydrophila
23	Bacteria	Proteobacteria	Betaproteobacteria				
23	Bacteria	Nitrospirae	Nitrospira	Nitrospirales	Nitrospiraceae	Nitrospira	Candidatus Nitrospira nitrificans
23	Bacteria	FCB group	Bacteroidetes/Chlorobi group	Bacteroidetes	Bacteroidia	Bacteroidales	Rikenellaceae
22	Bacteria	Terrabacteria group	Actinobacteria	Coriobacteriia	Coriobacteriales	Coriobacteriaceae	Collinsella
22	Bacteria	Proteobacteria	Gammaproteobacteria	Vibrionales	Vibrionaceae	Vibrio	Vibrio cholerae
22	Bacteria	Proteobacteria	Gammaproteobacteria	Enterobacterales	Enterobacteriaceae	Klebsiella	Klebsiella pneumoniae
22	Bacteria	Proteobacteria	Gammaproteobacteria	Enterobacterales	Enterobacteriaceae	Klebsiella	Klebsiella pneumoniae
22	Bacteria	Proteobacteria	Betaproteobacteria	unclassified Betaproteobacteria	unclassified Betaproteobacteria (miscellaneous)	Betaproteobacteria bacterium SG8_40	
22	Bacteria	Proteobacteria	Betaproteobacteria	Rhodocyclales	Rhodocyclaceae	Azovibrio	Azovibrio restrictus
22	dsDNA viruses, no RNA stage	Caudovirales	Myoviridae	unclassified Myoviridae	Escherichia coli O157 typing phage 15		
21	Bacteria	Proteobacteria	Gammaproteobacteria	Vibrionales	Vibrionaceae	Vibrio	Vibrio harveyi group
21	Bacteria	Proteobacteria	Gammaproteobacteria	Vibrionales	Vibrionaceae	Aliivibrio	Aliivibrio fischeri
21	Bacteria	Proteobacteria	Gammaproteobacteria	Enterobacterales	Enterobacteriaceae	unclassified Enterobacteriaceae	unclassified Enterobacteriaceae (miscellaneous)
21	Bacteria	Proteobacteria	Gammaproteobacteria	Enterobacterales	Enterobacteriaceae	Salmonella	Salmonella enterica
21	Bacteria	Proteobacteria	Gammaproteobacteria	Enterobacterales	Enterobacteriaceae	Raoultella	Raoultella planticola
21	Bacteria	Proteobacteria	Gammaproteobacteria	Enterobacterales	Enterobacteriaceae	Klebsiella	Klebsiella pneumoniae
21	Bacteria	Proteobacteria	Gammaproteobacteria	Enterobacterales	Enterobacteriaceae	Escherichia	Escherichia sp. KTE52
21	Bacteria	Proteobacteria	Gammaproteobacteria	Enterobacterales	Enterobacteriaceae	Escherichia	Escherichia coli
21	Bacteria	Proteobacteria	Gammaproteobacteria	Enterobacterales	Enterobacteriaceae	Enterobacter	Enterobacter cloacae complex
21	Bacteria	Proteobacteria	Alphaproteobacteria	Rhizobiales	Hyphomicrobiaceae	Hyphomicrobium	
20	Eukaryota	Opisthokonta	Fungi	Microsporidia	Apansporoblastina	Enterocytozoonidae	Enterospora
20	Bacteria	Terrabacteria group	Cyanobacteria/Melainabacteria group	Cyanobacteria	Nostocales	Nostocaceae	Anabaena
20	Bacteria	Proteobacteria	Gammaproteobacteria	Vibrionales	Vibrionaceae	Vibrio	Vibrio furnissii
20	Bacteria	Proteobacteria	Gammaproteobacteria	Vibrionales	Vibrionaceae	Vibrio	Vibrio albensis
20	Bacteria	Proteobacteria	Gammaproteobacteria	Oceanospirillales	Halomonadaceae	Halomonas	Halomonas titanicae
20	Bacteria	Proteobacteria	Gammaproteobacteria	Enterobacterales	Enterobacteriaceae	Salmonella	Salmonella enterica
20	Bacteria	Proteobacteria	Gammaproteobacteria	Enterobacterales	Enterobacteriaceae	Klebsiella	Klebsiella pneumoniae
20	Bacteria	Proteobacteria	Gammaproteobacteria	Enterobacterales	Enterobacteriaceae	Klebsiella	Klebsiella pneumoniae
20	Bacteria	Proteobacteria	Gammaproteobacteria	Enterobacterales	Enterobacteriaceae	Escherichia	Escherichia fergusonii
20	Bacteria	Proteobacteria	Gammaproteobacteria	Enterobacterales	Enterobacteriaceae	Citrobacter	Citrobacter sp. KTE151
20	Bacteria	Proteobacteria	Gammaproteobacteria	Aeromonadales	Aeromonadaceae	Aeromonas	Aeromonas salmonicida
20	Bacteria	Proteobacteria	Gammaproteobacteria	Aeromonadales	Aeromonadaceae	Aeromonas	Aeromonas eucrenophila
20	Bacteria	Proteobacteria	Alphaproteobacteria	Sphingomonadales	Sphingomonadaceae	Sphingomonas	
20	Bacteria	FCB group	Bacteroidetes/Chlorobi group	Bacteroidetes	Bacteroidia	Bacteroidales	Porphyromonadaceae
20	Bacteria	FCB group	Bacteroidetes/Chlorobi group	Bacteroidetes	Bacteroidia	Bacteroidales	Porphyromonadaceae
19	Eukaryota	Opisthokonta	Fungi	Dikarya	Ascomycota	saccharomyceta	Pezizomycotina
19	Bacteria	Terrabacteria group	Firmicutes	Clostridia	Clostridiales	Peptostreptococcaceae	Clostridioides
19	Bacteria	Proteobacteria	Gammaproteobacteria	Vibrionales	Vibrionaceae	Vibrio	Vibrio cholerae
19	Bacteria	Proteobacteria	Gammaproteobacteria	Enterobacterales	Enterobacteriaceae	Kluyvera	Kluyvera cryocrescens
19	Bacteria	Proteobacteria	Gammaproteobacteria	Enterobacterales	Enterobacteriaceae	Escherichia	Escherichia coli
18	Bacteria	Proteobacteria	Gammaproteobacteria	Vibrionales	Vibrionaceae	Vibrio	Vibrio sp. 5234-5
18	Bacteria	Proteobacteria	Gammaproteobacteria	Vibrionales	Vibrionaceae	Vibrio	Vibrio cholerae
18	Bacteria	Proteobacteria	Gammaproteobacteria	Pseudomonadales	Pseudomonadaceae	Pseudomonas	Pseudomonas aeruginosa group
18	Bacteria	Proteobacteria	Gammaproteobacteria	Enterobacterales	Enterobacteriaceae	Salmonella	Salmonella bongori
18	Bacteria	Proteobacteria	Gammaproteobacteria	Enterobacterales	Enterobacteriaceae	Klebsiella	Klebsiella sp. RIT-PI-d
18	Bacteria	Proteobacteria	Gammaproteobacteria	Enterobacterales	Enterobacteriaceae	Klebsiella	Klebsiella pneumoniae
18	Bacteria	Proteobacteria	Gammaproteobacteria	Enterobacterales	Enterobacteriaceae	Escherichia	Escherichia albertii
18	Bacteria	Proteobacteria	Alphaproteobacteria	Caulobacterales	Caulobacteraceae	unclassified Caulobacteraceae	Caulobacteraceae bacterium OTSz_A_272
18	Bacteria	FCB group	Bacteroidetes/Chlorobi group	Bacteroidetes	Bacteroidia	Bacteroidales	Rikenellaceae
18	Bacteria	FCB group	Bacteroidetes/Chlorobi group	Bacteroidetes	Bacteroidia	Bacteroidales	Rikenellaceae
18	Bacteria	FCB group	Bacteroidetes/Chlorobi group	Bacteroidetes	Bacteroidia	Bacteroidales	Porphyromonadaceae
18	Bacteria	FCB group	Bacteroidetes/Chlorobi group	Bacteroidetes	Bacteroidia	Bacteroidales	Porphyromonadaceae
17	Bacteria	Proteobacteria	Gammaproteobacteria	Vibrionales	Vibrionaceae	Vibrio	Vibrio ordalii
17	Bacteria	Proteobacteria	Gammaproteobacteria	Vibrionales	Vibrionaceae	Vibrio	Vibrio cholerae
17	Bacteria	Proteobacteria	Gammaproteobacteria	Vibrionales	Vibrionaceae	Vibrio	Vibrio albensis
17	Bacteria	Proteobacteria	Gammaproteobacteria	Vibrionales	Vibrionaceae	Photobacterium	Photobacterium damselae
17	Bacteria	Proteobacteria	Gammaproteobacteria	Enterobacterales	Enterobacteriaceae	Klebsiella	Klebsiella pneumoniae
17	Bacteria	Proteobacteria	Gammaproteobacteria	Enterobacterales	Enterobacteriaceae	Escherichia	Escherichia coli
17	Bacteria	Proteobacteria	Gammaproteobacteria	Enterobacterales	Enterobacteriaceae	Escherichia	Escherichia coli
17	Bacteria	Proteobacteria	Gammaproteobacteria	Enterobacterales	Enterobacteriaceae	Escherichia	Escherichia coli
17	Bacteria	Proteobacteria	Gammaproteobacteria	Enterobacterales	Enterobacteriaceae	Citrobacter	Citrobacter sp. FDAARGOS_156
17	Bacteria	Proteobacteria	Gammaproteobacteria	Enterobacterales	Enterobacteriaceae	Citrobacter	Citrobacter freundii complex
17	Bacteria	PVC group	Verrucomicrobia	Verrucomicrobiae	Verrucomicrobiales	Verrucomicrobiaceae	Haloferula
17	Bacteria	PVC group	Planctomycetes	Planctomycetia	Planctomycetales	Planctomycetaceae	Fuerstia
17	Bacteria	Nitrospirae	Nitrospira	Nitrospirales	Nitrospiraceae	Nitrospira	Nitrospira sp. SG-bin1
17	Bacteria	FCB group	Bacteroidetes/Chlorobi group	Bacteroidetes	Bacteroidia	Bacteroidales	Rikenellaceae
17	dsDNA viruses, no RNA stage	Caudovirales	Myoviridae	unclassified Myoviridae	Yersinia phage PY100		
16	Bacteria	Proteobacteria	delta/epsilon subdivisions	Deltaproteobacteria	Desulfovibrionales	Desulfovibrionaceae	Bilophila
16	Bacteria	Proteobacteria	Gammaproteobacteria	Xanthomonadales	Xanthomonadaceae	Xanthomonas	Xanthomonas citri group
16	Bacteria	Proteobacteria	Gammaproteobacteria	Vibrionales	Vibrionaceae	Vibrio	Vibrio cholerae
16	Bacteria	Proteobacteria	Gammaproteobacteria	Oceanospirillales	Oceanospirillaceae	Nitrincola	Nitrincola nitratireducens
16	Bacteria	Proteobacteria	Gammaproteobacteria	Enterobacterales	Yersiniaceae	Yersinia	
16	Bacteria	Proteobacteria	Gammaproteobacteria	Enterobacterales	Enterobacteriaceae	Escherichia	Escherichia coli
16	Bacteria	Proteobacteria	Gammaproteobacteria	Enterobacterales	Enterobacteriaceae	Escherichia	Escherichia coli
16	Bacteria	Proteobacteria	Gammaproteobacteria	Enterobacterales	Enterobacteriaceae	Escherichia	Escherichia coli
16	Bacteria	Proteobacteria	Gammaproteobacteria	Enterobacterales	Enterobacteriaceae	Escherichia	Escherichia coli
16	Bacteria	Proteobacteria	Gammaproteobacteria	Enterobacterales	Enterobacteriaceae	Escherichia	Escherichia coli
16	Bacteria	Proteobacteria	Gammaproteobacteria	Enterobacterales	Enterobacteriaceae	Escherichia	Escherichia coli
16	Bacteria	Proteobacteria	Gammaproteobacteria	Enterobacterales	Enterobacteriaceae	Escherichia	Escherichia coli
16	Bacteria	Proteobacteria	Gammaproteobacteria	Enterobacterales	Enterobacteriaceae	Escherichia	Escherichia coli
16	Bacteria	Proteobacteria	Gammaproteobacteria	Enterobacterales	Enterobacteriaceae	Cronobacter	
16	Bacteria	Proteobacteria	Gammaproteobacteria	Enterobacterales	Enterobacteriaceae	Citrobacter	Citrobacter farmeri
16	Bacteria	Proteobacteria	Gammaproteobacteria	Aeromonadales	Aeromonadaceae	Aeromonas	Aeromonas bestiarum
16	Bacteria	Proteobacteria	Betaproteobacteria	Burkholderiales	Comamonadaceae	Curvibacter	Curvibacter putative symbiont of Hydra magnipapillata
16	Bacteria	PVC group	Verrucomicrobia	Verrucomicrobiae	Verrucomicrobiales	Akkermansiaceae	Akkermansia
16	Bacteria	FCB group	Bacteroidetes/Chlorobi group	Bacteroidetes	Bacteroidia	Bacteroidales	Rikenellaceae
15	Eukaryota						
15	Bacteria	Terrabacteria group	Actinobacteria				
15	Bacteria	Proteobacteria	delta/epsilon subdivisions	Deltaproteobacteria	Desulfovibrionales	Desulfovibrionaceae	Desulfovibrio
15	Bacteria	Proteobacteria	Gammaproteobacteria	Vibrionales	Vibrionaceae	Vibrio	Vibrio harveyi group
15	Bacteria	Proteobacteria	Gammaproteobacteria	Enterobacterales	Morganellaceae	Xenorhabdus	Xenorhabdus innexi
15	Bacteria	Proteobacteria	Gammaproteobacteria	Enterobacterales	Enterobacteriaceae	Yokenella	Yokenella regensburgei
15	Bacteria	Proteobacteria	Gammaproteobacteria	Enterobacterales	Enterobacteriaceae	Shigella	Shigella flexneri
15	Bacteria	Proteobacteria	Gammaproteobacteria	Enterobacterales	Enterobacteriaceae	Shigella	Shigella flexneri
15	Bacteria	Proteobacteria	Gammaproteobacteria	Enterobacterales	Enterobacteriaceae	Pluralibacter	Pluralibacter gergoviae
15	Bacteria	Proteobacteria	Gammaproteobacteria	Enterobacterales	Enterobacteriaceae	Klebsiella	Klebsiella quasipneumoniae
15	Bacteria	Proteobacteria	Gammaproteobacteria	Enterobacterales	Enterobacteriaceae	Klebsiella	Klebsiella pneumoniae
15	Bacteria	Proteobacteria	Gammaproteobacteria	Enterobacterales	Enterobacteriaceae	Escherichia	Escherichia coli
15	Bacteria	Proteobacteria	Gammaproteobacteria	Enterobacterales	Enterobacteriaceae	Escherichia	Escherichia coli
15	Bacteria	Proteobacteria	Gammaproteobacteria	Enterobacterales	Enterobacteriaceae	Escherichia	Escherichia coli
15	Bacteria	Proteobacteria	Gammaproteobacteria	Aeromonadales	Aeromonadaceae	Aeromonas	Aeromonas sp. HZM
15	Bacteria	Proteobacteria	Alphaproteobacteria	Sphingomonadales	Sphingomonadaceae		
15	Bacteria	Proteobacteria	Alphaproteobacteria	Rickettsiales	Anaplasmataceae	Wolbachieae	Wolbachia
15	Bacteria	Proteobacteria	Alphaproteobacteria	Holosporales	unclassified Holosporales	Caedibacter	Caedibacter varicaedens
15	Bacteria	PVC group	Planctomycetes	Planctomycetia	Planctomycetales	Planctomycetaceae	Pirellula
15	Bacteria	Nitrospirae	Nitrospira	Nitrospirales	Nitrospiraceae	Nitrospira	Nitrospira sp. HN-bin3
15	Bacteria	FCB group	Bacteroidetes/Chlorobi group	Bacteroidetes	Bacteroidia	Bacteroidales	Rikenellaceae
15	Bacteria	FCB group	Bacteroidetes/Chlorobi group	Bacteroidetes	Bacteroidia	Bacteroidales	Porphyromonadaceae
15	environmental samples	uncultured Mediterranean phage uvMED					
14	Bacteria	Terrabacteria group	Firmicutes	Clostridia	Clostridiales	Ruminococcaceae	Faecalibacterium
14	Bacteria	Terrabacteria group	Actinobacteria	Actinobacteria	Corynebacteriales	Mycobacteriaceae	Mycobacterium
14	Bacteria	Proteobacteria	Gammaproteobacteria	Vibrionales	Vibrionaceae	Vibrio	Vibrio harveyi group
14	Bacteria	Proteobacteria	Gammaproteobacteria	Vibrionales	Vibrionaceae	Vibrio	Vibrio cholerae
14	Bacteria	Proteobacteria	Gammaproteobacteria	Vibrionales	Vibrionaceae	Photobacterium	Photobacterium phosphoreum
14	Bacteria	Proteobacteria	Gammaproteobacteria	Enterobacterales	Enterobacteriaceae	Escherichia	Escherichia coli
14	Bacteria	Proteobacteria	Gammaproteobacteria	Enterobacterales	Enterobacteriaceae	Escherichia	Escherichia coli
14	Bacteria	Proteobacteria	Gammaproteobacteria	Enterobacterales	Enterobacteriaceae	Escherichia	Escherichia coli
14	Bacteria	Proteobacteria	Gammaproteobacteria	Enterobacterales	Enterobacteriaceae	Enterobacter	Enterobacter cloacae complex
14	Bacteria	Proteobacteria	Gammaproteobacteria	Aeromonadales	Aeromonadaceae	Aeromonas	Aeromonas salmonicida
14	Bacteria	PVC group	Planctomycetes	Planctomycetia	Planctomycetales	Gemmataceae	Gemmata
14	Bacteria	FCB group	Bacteroidetes/Chlorobi group	Bacteroidetes	Bacteroidia	Bacteroidales	Porphyromonadaceae
13	Eukaryota	Opisthokonta	Fungi	Dikarya	Ascomycota	saccharomyceta	Pezizomycotina
13	Bacteria	Terrabacteria group	Firmicutes	Bacilli	Lactobacillales	Streptococcaceae	Streptococcus
13	Bacteria	Proteobacteria	Gammaproteobacteria	Vibrionales	Vibrionaceae	Vibrio	Vibrio navarrensis
13	Bacteria	Proteobacteria	Gammaproteobacteria	Vibrionales	Vibrionaceae	Vibrio	Vibrio harveyi group
13	Bacteria	Proteobacteria	Gammaproteobacteria	Vibrionales	Vibrionaceae	Salinivibrio	Salinivibrio sp. IB282
13	Bacteria	Proteobacteria	Gammaproteobacteria	Oceanospirillales	Oceanospirillaceae	Oleibacter	Oleibacter marinus
13	Bacteria	Proteobacteria	Gammaproteobacteria	Oceanospirillales	Hahellaceae	Endozoicomonas	Endozoicomonas numazuensis
13	Bacteria	Proteobacteria	Gammaproteobacteria	Methylococcales	Methylococcaceae	Methylococcus	Methylococcus capsulatus
13	Bacteria	Proteobacteria	Gammaproteobacteria	Enterobacterales	Yersiniaceae	Yersinia	Yersinia pseudotuberculosis complex
13	Bacteria	Proteobacteria	Gammaproteobacteria	Enterobacterales	Enterobacteriaceae	Shigella	Shigella dysenteriae
13	Bacteria	Proteobacteria	Gammaproteobacteria	Enterobacterales	Enterobacteriaceae	Kluyvera	
13	Bacteria	Proteobacteria	Gammaproteobacteria	Enterobacterales	Enterobacteriaceae	Klebsiella	Klebsiella quasipneumoniae
13	Bacteria	Proteobacteria	Gammaproteobacteria	Enterobacterales	Enterobacteriaceae	Escherichia	Escherichia sp. KTE114
13	Bacteria	Proteobacteria	Gammaproteobacteria	Enterobacterales	Enterobacteriaceae	Escherichia	Escherichia coli
13	Bacteria	Proteobacteria	Gammaproteobacteria	Enterobacterales	Enterobacteriaceae	Escherichia	Escherichia coli
13	Bacteria	Proteobacteria	Gammaproteobacteria	Enterobacterales	Enterobacteriaceae	Escherichia	Escherichia coli
13	Bacteria	Proteobacteria	Gammaproteobacteria	Enterobacterales	Enterobacteriaceae	Escherichia	Escherichia albertii
13	Bacteria	Proteobacteria	Gammaproteobacteria	Enterobacterales	Enterobacteriaceae	Citrobacter	Citrobacter rodentium
13	Bacteria	Proteobacteria	Gammaproteobacteria	Aeromonadales	Aeromonadaceae	Aeromonas	Aeromonas veronii
13	Bacteria	Proteobacteria	Betaproteobacteria	unclassified Betaproteobacteria	unclassified Betaproteobacteria (miscellaneous)		
13	Bacteria	Proteobacteria	Alphaproteobacteria	Sphingomonadales	Sphingomonadaceae	Sphingopyxis	Sphingopyxis sp. LPB0140
13	Bacteria	Proteobacteria	Alphaproteobacteria	Sphingomonadales			
13	Bacteria	Proteobacteria	Alphaproteobacteria	Rhodobacterales	unclassified Rhodobacterales		
13	Bacteria	PVC group	Planctomycetes	Planctomycetia	Planctomycetales	Planctomycetaceae	Planctomyces
13	Bacteria	PVC group	Planctomycetes	Planctomycetia	Planctomycetales	Planctomycetaceae	Blastopirellula
13	Bacteria	PVC group	Chlamydiae	Chlamydiia	Chlamydiales	Chlamydiaceae	Chlamydia/Chlamydophila group
13	Bacteria	FCB group	Bacteroidetes/Chlorobi group	Bacteroidetes	Flavobacteriia	Flavobacteriales	Flavobacteriaceae
13	Bacteria	FCB group	Bacteroidetes/Chlorobi group	Bacteroidetes	Bacteroidia	Bacteroidales	Bacteroidaceae
13	Bacteria	FCB group	Bacteroidetes/Chlorobi group	Bacteroidetes	Bacteroidia		
12	Bacteria	Proteobacteria	Gammaproteobacteria	Vibrionales	Vibrionaceae	Vibrio	Vibrio tritonius
12	Bacteria	Proteobacteria	Gammaproteobacteria	Vibrionales	Vibrionaceae	Vibrio	Vibrio crassostreae
12	Bacteria	Proteobacteria	Gammaproteobacteria	Vibrionales	Vibrionaceae	Vibrio	Vibrio coralliilyticus
12	Bacteria	Proteobacteria	Gammaproteobacteria	Vibrionales	Vibrionaceae	Vibrio	Vibrio cholerae
12	Bacteria	Proteobacteria	Gammaproteobacteria	Vibrionales	Vibrionaceae	Photobacterium	Photobacterium leiognathi
12	Bacteria	Proteobacteria	Gammaproteobacteria	Pasteurellales	Pasteurellaceae	Mannheimia	Mannheimia haemolytica
12	Bacteria	Proteobacteria	Gammaproteobacteria	Pasteurellales	Pasteurellaceae	Haemophilus	
12	Bacteria	Proteobacteria	Gammaproteobacteria	Enterobacterales	Yersiniaceae	Serratia	
12	Bacteria	Proteobacteria	Gammaproteobacteria	Enterobacterales	Yersiniaceae	Rahnella	
12	Bacteria	Proteobacteria	Gammaproteobacteria	Enterobacterales	Pectobacteriaceae	Pectobacterium	Pectobacterium carotovorum
12	Bacteria	Proteobacteria	Gammaproteobacteria	Enterobacterales	Enterobacteriaceae	Raoultella	Raoultella ornithinolytica
12	Bacteria	Proteobacteria	Gammaproteobacteria	Enterobacterales	Enterobacteriaceae	Escherichia	Escherichia marmotae
12	Bacteria	Proteobacteria	Gammaproteobacteria	Enterobacterales	Enterobacteriaceae	Escherichia	Escherichia fergusonii
12	Bacteria	Proteobacteria	Gammaproteobacteria	Enterobacterales	Enterobacteriaceae	Escherichia	Escherichia coli
12	Bacteria	Proteobacteria	Gammaproteobacteria	Enterobacterales	Enterobacteriaceae	Escherichia	Escherichia coli
12	Bacteria	Proteobacteria	Gammaproteobacteria	Enterobacterales	Enterobacteriaceae	Cronobacter	Cronobacter sakazakii
12	Bacteria	Proteobacteria	Gammaproteobacteria	Enterobacterales	Enterobacteriaceae	Cronobacter	Cronobacter sakazakii
12	Bacteria	Proteobacteria	Gammaproteobacteria	Enterobacterales	Enterobacteriaceae	Cedecea	Cedecea davisae
12	Bacteria	Proteobacteria	Alphaproteobacteria	Rickettsiales	Anaplasmataceae	Anaplasma	phagocytophilum group
12	Bacteria	Proteobacteria	Alphaproteobacteria	Rhizobiales	Rhizobiaceae	Rhizobium/Agrobacterium group	Rhizobium
12	Bacteria	PVC group	Planctomycetes	Planctomycetia	Planctomycetales	Planctomycetaceae	unclassified Planctomycetaceae
12	Bacteria	Nitrospirae	Nitrospira	Nitrospirales	Nitrospiraceae	Nitrospira	
12	Bacteria	FCB group	Bacteroidetes/Chlorobi group	Bacteroidetes	Flavobacteriia	Flavobacteriales	Flavobacteriaceae
12	Bacteria	FCB group	Bacteroidetes/Chlorobi group	Bacteroidetes	Bacteroidia	Bacteroidales	Prevotellaceae
12	Bacteria	Acidobacteria	unclassified Acidobacteria	unclassified Acidobacteria (miscellaneous)			
12	dsDNA viruses, no RNA stage	Caudovirales	Podoviridae	unclassified Podoviridae	Enterobacteria phage IME10		
11	Eukaryota	Amoebozoa	Tubulinea	Euamoebida	Tubulinida	Hartmannellidae	Vermamoeba
11	Bacteria	Terrabacteria group	Actinobacteria	Actinobacteria	Streptomycetales	Streptomycetaceae	Streptomyces
11	Bacteria	Synergistetes	Synergistia	Synergistales	Synergistaceae	Fretibacterium	Fretibacterium fastidiosum
11	Bacteria	Proteobacteria	Gammaproteobacteria	environmental samples	uncultured gamma proteobacterium HF0010_01E20		
11	Bacteria	Proteobacteria	Gammaproteobacteria	Vibrionales	Vibrionaceae	Vibrio	Vibrio tasmaniensis
11	Bacteria	Proteobacteria	Gammaproteobacteria	Vibrionales	Vibrionaceae	Vibrio	Vibrio neptunius
11	Bacteria	Proteobacteria	Gammaproteobacteria	Vibrionales	Vibrionaceae	Vibrio	Vibrio ichthyoenteri
11	Bacteria	Proteobacteria	Gammaproteobacteria	Vibrionales	Vibrionaceae	Vibrio	Vibrio cyclitrophicus
11	Bacteria	Proteobacteria	Gammaproteobacteria	Vibrionales	Vibrionaceae	Vibrio	Vibrio cholerae
11	Bacteria	Proteobacteria	Gammaproteobacteria	Vibrionales	Vibrionaceae	Salinivibrio	
11	Bacteria	Proteobacteria	Gammaproteobacteria	Pasteurellales	Pasteurellaceae	Histophilus	Histophilus somni
11	Bacteria	Proteobacteria	Gammaproteobacteria	Enterobacterales	Enterobacteriaceae	Escherichia	Escherichia coli
11	Bacteria	Proteobacteria	Gammaproteobacteria	Enterobacterales	Enterobacteriaceae	Escherichia	Escherichia coli
11	Bacteria	Proteobacteria	Gammaproteobacteria	Enterobacterales	Enterobacteriaceae	Escherichia	Escherichia coli
11	Bacteria	Proteobacteria	Gammaproteobacteria	Enterobacterales	Enterobacteriaceae	Escherichia	Escherichia coli
11	Bacteria	Proteobacteria	Gammaproteobacteria	Enterobacterales	Enterobacteriaceae	Escherichia	Escherichia coli
11	Bacteria	Proteobacteria	Gammaproteobacteria	Enterobacterales	Enterobacteriaceae	Escherichia	Escherichia coli
11	Bacteria	Proteobacteria	Gammaproteobacteria	Enterobacterales	Enterobacteriaceae	Escherichia	Escherichia coli
11	Bacteria	Proteobacteria	Gammaproteobacteria	Enterobacterales	Enterobacteriaceae	Escherichia	Escherichia coli
11	Bacteria	Proteobacteria	Gammaproteobacteria	Aeromonadales	Aeromonadaceae	Aeromonas	Aeromonas salmonicida
11	Bacteria	Proteobacteria	Betaproteobacteria	Burkholderiales	Burkholderiaceae	Burkholderia	
11	Bacteria	Proteobacteria	Alphaproteobacteria	Sphingomonadales	unclassified Sphingomonadales	Sphingomonadales bacterium 63-6	
11	Bacteria	Proteobacteria	Alphaproteobacteria	Rhodobacterales			
11	Bacteria	PVC group	Planctomycetes	Planctomycetia	Planctomycetales	Planctomycetaceae	Roseimaritima
11	Bacteria	PVC group	Planctomycetes	Planctomycetia	Planctomycetales	Planctomycetaceae	Pirellula
11	Bacteria	FCB group	Bacteroidetes/Chlorobi group	Bacteroidetes	Bacteroidia	Bacteroidales	Porphyromonadaceae
11	Bacteria	FCB group	Bacteroidetes/Chlorobi group	Bacteroidetes	Bacteroidia	Bacteroidales	Porphyromonadaceae
10	Eukaryota	Opisthokonta	Choanoflagellida	Craspedida	Salpingoecidae	Monosiga	Monosiga brevicollis
10	Bacteria	Terrabacteria group	Firmicutes	Clostridia	Clostridiales	Clostridiaceae	Clostridium
10	Bacteria	Terrabacteria group	Firmicutes	Clostridia	Clostridiales	Clostridiaceae	Clostridium
10	Bacteria	Terrabacteria group	Firmicutes	Bacilli	Lactobacillales		
10	Bacteria	Terrabacteria group	Actinobacteria	Actinobacteria			
10	Bacteria	Proteobacteria	delta/epsilon subdivisions	Deltaproteobacteria	unclassified Deltaproteobacteria	Candidatus Adiutrix	Candidatus Adiutrix intracellularis
10	Bacteria	Proteobacteria	Gammaproteobacteria	Vibrionales	Vibrionaceae	Vibrio	Vibrio sp. ZOR0018
10	Bacteria	Proteobacteria	Gammaproteobacteria	Vibrionales	Vibrionaceae	Vibrio	Vibrio oreintalis group
10	Bacteria	Proteobacteria	Gammaproteobacteria	Vibrionales	Vibrionaceae	Vibrio	Vibrio harveyi group
10	Bacteria	Proteobacteria	Gammaproteobacteria	Vibrionales	Vibrionaceae	Vibrio	Vibrio harveyi group
10	Bacteria	Proteobacteria	Gammaproteobacteria	Vibrionales	Vibrionaceae	Vibrio	Vibrio cidicii
10	Bacteria	Proteobacteria	Gammaproteobacteria	Vibrionales	Vibrionaceae	Vibrio	Vibrio cholerae
10	Bacteria	Proteobacteria	Gammaproteobacteria	Orbales	Orbaceae	Gilliamella	Gilliamella apicola
10	Bacteria	Proteobacteria	Gammaproteobacteria	Oceanospirillales	Oceanospirillaceae	Neptuniibacter	Neptuniibacter pectenicola
10	Bacteria	Proteobacteria	Gammaproteobacteria	Enterobacterales	Yersiniaceae	Serratia	Serratia symbiotica
10	Bacteria	Proteobacteria	Gammaproteobacteria	Enterobacterales	Enterobacteriaceae	Trabulsiella	Trabulsiella odontotermitis
10	Bacteria	Proteobacteria	Gammaproteobacteria	Enterobacterales	Enterobacteriaceae	Shigella	Shigella sp. SF-2015
10	Bacteria	Proteobacteria	Gammaproteobacteria	Enterobacterales	Enterobacteriaceae	Salmonella	Salmonella enterica
10	Bacteria	Proteobacteria	Gammaproteobacteria	Enterobacterales	Enterobacteriaceae	Salmonella	Salmonella enterica
10	Bacteria	Proteobacteria	Gammaproteobacteria	Enterobacterales	Enterobacteriaceae	Lelliottia	Lelliottia amnigena
10	Bacteria	Proteobacteria	Gammaproteobacteria	Enterobacterales	Enterobacteriaceae	Klebsiella	Klebsiella michiganensis
10	Bacteria	Proteobacteria	Gammaproteobacteria	Enterobacterales	Enterobacteriaceae	Escherichia	Escherichia coli
10	Bacteria	Proteobacteria	Gammaproteobacteria	Enterobacterales	Enterobacteriaceae	Escherichia	Escherichia coli

10	Bacteria	Proteobacteria	Gammaproteobacteria	Enterobacterales	Enterobacteriaceae	Escherichia	Escherichia coli
10	Bacteria	Proteobacteria	Gammaproteobacteria	Enterobacterales	Enterobacteriaceae	Escherichia	Escherichia coli
10	Bacteria	Proteobacteria	Gammaproteobacteria	Enterobacterales	Enterobacteriaceae	Escherichia	Escherichia coli
10	Bacteria	Proteobacteria	Gammaproteobacteria	Enterobacterales	Enterobacteriaceae	Escherichia	Escherichia coli
10	Bacteria	Proteobacteria	Gammaproteobacteria	Enterobacterales	Enterobacteriaceae	Escherichia	Escherichia coli
10	Bacteria	Proteobacteria	Gammaproteobacteria	Enterobacterales	Enterobacteriaceae	Escherichia	Escherichia coli
10	Bacteria	Proteobacteria	Gammaproteobacteria	Enterobacterales	Enterobacteriaceae	Escherichia	Escherichia coli
10	Bacteria	Proteobacteria	Gammaproteobacteria	Enterobacterales	Enterobacteriaceae	Cronobacter	Cronobacter turicensis
10	Bacteria	Proteobacteria	Gammaproteobacteria	Enterobacterales	Enterobacteriaceae	Citrobacter	Citrobacter freundii complex
10	Bacteria	Proteobacteria	Gammaproteobacteria	Enterobacterales	Enterobacteriaceae	Citrobacter	Citrobacter freundii complex
10	Bacteria	Proteobacteria	Gammaproteobacteria	Alteromonadales	Shewanellaceae	Shewanella	Shewanella xiamenensis
10	Bacteria	Proteobacteria	Gammaproteobacteria	Aeromonadales	Aeromonadaceae	Aeromonas	Aeromonas simiae
10	Bacteria	Proteobacteria	Gammaproteobacteria	Aeromonadales	Aeromonadaceae	Aeromonas	Aeromonas hydrophila
10	Bacteria	Proteobacteria	Alphaproteobacteria	Rhodospirillales	Rhodospirillaceae	Pararhodospirillum	Pararhodospirillum photometricum
10	Bacteria	Proteobacteria	Alphaproteobacteria	Rhodobacterales	Rhodobacteraceae	Gemmobacter	Gemmobacter aquatilis
10	Bacteria	Proteobacteria	Alphaproteobacteria	Rhizobiales	Rhizobiaceae	Rhizobium/Agrobacterium group	
10	Bacteria	Proteobacteria	Alphaproteobacteria	Rhizobiales	Hyphomicrobiaceae	Devosia	
10	Bacteria	PVC group	Verrucomicrobia	Verrucomicrobiae	Verrucomicrobiales	Verrucomicrobiaceae	Verrucomicrobium
10	Bacteria	PVC group	Planctomycetes	Planctomycetia	Planctomycetales	Planctomycetaceae	Rhodopirellula
10	Bacteria	PVC group	Planctomycetes	Planctomycetia	Planctomycetales	Planctomycetaceae	Planctomicrobium
10	Bacteria	PVC group	Planctomycetes	Planctomycetia	Planctomycetales	Gemmataceae	Gemmata
10	Bacteria	FCB group	Bacteroidetes/Chlorobi group	Bacteroidetes	Bacteroidia	Bacteroidales	unclassified Bacteroidales
10	Bacteria	FCB group	Bacteroidetes/Chlorobi group	Bacteroidetes	Bacteroidia	Bacteroidales	unclassified Bacteroidales
10	Bacteria	FCB group	Bacteroidetes/Chlorobi group	Bacteroidetes	Bacteroidia	Bacteroidales	Porphyromonadaceae
10	dsDNA viruses, no RNA stage	Caudovirales	Myoviridae	Ounavirinae	Felixo1virus	Escherichia virus TP1	Escherichia coli O157 typing phage 1
9	Bacteria	unclassified Bacteria	Bacteria candidate phyla	Candidatus Rokubacteria			
9	Bacteria	Terrabacteria group	Firmicutes	Bacilli	Lactobacillales	Enterococcaceae	Enterococcus
9	Bacteria	Synergistetes	Synergistia	Synergistales	Synergistaceae	Aminobacterium	Aminobacterium mobile
9	Bacteria	Proteobacteria	delta/epsilon subdivisions	Deltaproteobacteria	Desulfovibrionales	Desulfovibrionaceae	Desulfovibrio
9	Bacteria	Proteobacteria	delta/epsilon subdivisions	Deltaproteobacteria	Desulfovibrionales	Desulfovibrionaceae	Bilophila
9	Bacteria	Proteobacteria	Gammaproteobacteria	Vibrionales	Vibrionaceae	Vibrio	Vibrio tasmaniensis
9	Bacteria	Proteobacteria	Gammaproteobacteria	Vibrionales	Vibrionaceae	Vibrio	Vibrio sp. JCM 18905
9	Bacteria	Proteobacteria	Gammaproteobacteria	Vibrionales	Vibrionaceae	Vibrio	Vibrio proteolyticus
9	Bacteria	Proteobacteria	Gammaproteobacteria	Vibrionales	Vibrionaceae	Photobacterium	Photobacterium gaetbulicola
9	Bacteria	Proteobacteria	Gammaproteobacteria	Enterobacterales	Yersiniaceae	Yersinia	Yersinia pseudotuberculosis complex
9	Bacteria	Proteobacteria	Gammaproteobacteria	Enterobacterales	Yersiniaceae	Serratia	Serratia marcescens
9	Bacteria	Proteobacteria	Gammaproteobacteria	Enterobacterales	Morganellaceae	Xenorhabdus	Xenorhabdus szentirmaii
9	Bacteria	Proteobacteria	Gammaproteobacteria	Enterobacterales	Erwiniaceae	Pantoea	
9	Bacteria	Proteobacteria	Gammaproteobacteria	Enterobacterales	Enterobacteriaceae	Shigella	Shigella dysenteriae
9	Bacteria	Proteobacteria	Gammaproteobacteria	Enterobacterales	Enterobacteriaceae	Salmonella	Salmonella enterica
9	Bacteria	Proteobacteria	Gammaproteobacteria	Enterobacterales	Enterobacteriaceae	Escherichia	Escherichia sp. KTE96
9	Bacteria	Proteobacteria	Gammaproteobacteria	Enterobacterales	Enterobacteriaceae	Escherichia	Escherichia coli
9	Bacteria	Proteobacteria	Gammaproteobacteria	Enterobacterales	Enterobacteriaceae	Escherichia	Escherichia coli
9	Bacteria	Proteobacteria	Gammaproteobacteria	Enterobacterales	Enterobacteriaceae	Escherichia	Escherichia coli
9	Bacteria	Proteobacteria	Gammaproteobacteria	Enterobacterales	Enterobacteriaceae	Escherichia	Escherichia coli
9	Bacteria	Proteobacteria	Gammaproteobacteria	Enterobacterales	Enterobacteriaceae	Escherichia	Escherichia coli
9	Bacteria	Proteobacteria	Gammaproteobacteria	Enterobacterales	Enterobacteriaceae	Escherichia	Escherichia coli
9	Bacteria	Proteobacteria	Gammaproteobacteria	Enterobacterales	Enterobacteriaceae	Escherichia	Escherichia coli
9	Bacteria	Proteobacteria	Gammaproteobacteria	Enterobacterales	Enterobacteriaceae	Escherichia	Escherichia coli
9	Bacteria	Proteobacteria	Gammaproteobacteria	Enterobacterales	Enterobacteriaceae	Enterobacter	Enterobacter massiliensis
9	Bacteria	Proteobacteria	Gammaproteobacteria	Aeromonadales	Aeromonadaceae	Aeromonas	Aeromonas salmonicida
9	Bacteria	Proteobacteria	Betaproteobacteria	Rhodocyclales	Rhodocyclaceae	Azonexus	Azonexus hydrophilus
9	Bacteria	Proteobacteria	Alphaproteobacteria	Rhizobiales	Hyphomicrobiaceae	Hyphomicrobium	Hyphomicrobium sp. NDB2Meth4
9	Bacteria	PVC group	Planctomycetes	unclassified Planctomycetes	Planctomycetes bacterium RBG_16_64_12		
9	Bacteria	PVC group	Planctomycetes	Planctomycetia	Planctomycetales	Planctomycetaceae	Schlesneria
9	Bacteria	FCB group	Bacteroidetes/Chlorobi group	Bacteroidetes	Bacteroidia	Bacteroidales	Porphyromonadaceae
9	ssDNA viruses	Inoviridae	Inovirus	Vibrio phage fs1			
9	dsDNA viruses, no RNA stage	Caudovirales	Myoviridae	Ounavirinae			
9	Retro-transcribing viruses	Retroviridae	Orthoretrovirinae	Alpharetrovirus	Avian leukosis virus		
8	Eukaryota	Opisthokonta	Fungi	Dikarya	Ascomycota	saccharomyceta	Pezizomycotina
8	Bacteria	unclassified Bacteria	unclassified Bacteria (miscellaneous)	bacteria symbiont BFo1 of Frankliniella occidentalis			
8	Bacteria	Proteobacteria	unclassified Proteobacteria	unclassified Proteobacteria (miscellaneous)	Proteobacteria bacterium HN_bin10		
8	Bacteria	Proteobacteria	delta/epsilon subdivisions	Deltaproteobacteria	Desulfovibrionales	Desulfovibrionaceae	Halodesulfovibrio
8	Bacteria	Proteobacteria	delta/epsilon subdivisions	Deltaproteobacteria	Desulfovibrionales	Desulfovibrionaceae	Desulfovibrio
8	Bacteria	Proteobacteria	delta/epsilon subdivisions	Deltaproteobacteria	Desulfovibrionales	Desulfovibrionaceae	
8	Bacteria	Proteobacteria	Gammaproteobacteria	unclassified Gammaproteobacteria	sulfur-oxidizing symbionts	Solemya velum gill symbiont	
8	Bacteria	Proteobacteria	Gammaproteobacteria	Vibrionales	Vibrionaceae	Vibrio	Vibrio harveyi group
8	Bacteria	Proteobacteria	Gammaproteobacteria	Vibrionales	Vibrionaceae	Vibrio	Vibrio cholerae
[illegible]	[illegible]	[illegible]	[illegible]	[illegible]	[illegible]	[illegible]	[illegible]

4	Bacteria	Proteobacteria	Gammaproteobacteria	Enterobacterales	Enterobacteriaceae	Salmonella	Salmonella enterica
4	Bacteria	Proteobacteria	Gammaproteobacteria	Enterobacterales	Enterobacteriaceae	Salmonella	Salmonella enterica
4	Bacteria	Proteobacteria	Gammaproteobacteria	Enterobacterales	Enterobacteriaceae	Salmonella	Salmonella enterica
4	Bacteria	Proteobacteria	Gammaproteobacteria	Enterobacterales	Enterobacteriaceae	Salmonella	Salmonella enterica
4	Bacteria	Proteobacteria	Gammaproteobacteria	Enterobacterales	Enterobacteriaceae	Salmonella	Salmonella enterica
4	Bacteria	Proteobacteria	Gammaproteobacteria	Enterobacterales	Enterobacteriaceae	Salmonella	Salmonella bongori
4	Bacteria	Proteobacteria	Gammaproteobacteria	Enterobacterales	Enterobacteriaceae	Mangrovibacter	Mangrovibacter sp. MFB070
4	Bacteria	Proteobacteria	Gammaproteobacteria	Enterobacterales	Enterobacteriaceae	Leclercia	
4	Bacteria	Proteobacteria	Gammaproteobacteria	Enterobacterales	Enterobacteriaceae	Kosakonia	Kosakonia oryzendophytica
4	Bacteria	Proteobacteria	Gammaproteobacteria	Enterobacterales	Enterobacteriaceae	Klebsiella	Klebsiella sp. 1_1_55
4	Bacteria	Proteobacteria	Gammaproteobacteria	Enterobacterales	Enterobacteriaceae	Klebsiella	Klebsiella pneumoniae
4	Bacteria	Proteobacteria	Gammaproteobacteria	Enterobacterales	Enterobacteriaceae	Klebsiella	Klebsiella pneumoniae
4	Bacteria	Proteobacteria	Gammaproteobacteria	Enterobacterales	Enterobacteriaceae	Klebsiella	Klebsiella pneumoniae
4	Bacteria	Proteobacteria	Gammaproteobacteria	Enterobacterales	Enterobacteriaceae	Klebsiella	Klebsiella pneumoniae
4	Bacteria	Proteobacteria	Gammaproteobacteria	Enterobacterales	Enterobacteriaceae	Escherichia	Escherichia coli
4	Bacteria	Proteobacteria	Gammaproteobacteria	Enterobacterales	Enterobacteriaceae	Escherichia	Escherichia coli
4	Bacteria	Proteobacteria	Gammaproteobacteria	Enterobacterales	Enterobacteriaceae	Escherichia	Escherichia coli
4	Bacteria	Proteobacteria	Gammaproteobacteria	Enterobacterales	Enterobacteriaceae	Escherichia	Escherichia coli
4	Bacteria	Proteobacteria	Gammaproteobacteria	Enterobacterales	Enterobacteriaceae	Escherichia	Escherichia coli
4	Bacteria	Proteobacteria	Gammaproteobacteria	Enterobacterales	Enterobacteriaceae	Escherichia	Escherichia coli
4	Bacteria	Proteobacteria	Gammaproteobacteria	Enterobacterales	Enterobacteriaceae	Escherichia	Escherichia coli
4	Bacteria	Proteobacteria	Gammaproteobacteria	Enterobacterales	Enterobacteriaceae	Escherichia	Escherichia coli
4	Bacteria	Proteobacteria	Gammaproteobacteria	Enterobacterales	Enterobacteriaceae	Escherichia	Escherichia coli
4	Bacteria	Proteobacteria	Gammaproteobacteria	Enterobacterales	Enterobacteriaceae	Escherichia	Escherichia coli
4	Bacteria	Proteobacteria	Gammaproteobacteria	Enterobacterales	Enterobacteriaceae	Escherichia	Escherichia coli
4	Bacteria	Proteobacteria	Gammaproteobacteria	Enterobacterales	Enterobacteriaceae	Escherichia	Escherichia coli
4	Bacteria	Proteobacteria	Gammaproteobacteria	Enterobacterales	Enterobacteriaceae	Escherichia	Escherichia coli
4	Bacteria	Proteobacteria	Gammaproteobacteria	Enterobacterales	Enterobacteriaceae	Escherichia	Escherichia coli
4	Bacteria	Proteobacteria	Gammaproteobacteria	Enterobacterales	Enterobacteriaceae	Escherichia	Escherichia coli
4	Bacteria	Proteobacteria	Gammaproteobacteria	Enterobacterales	Enterobacteriaceae	Escherichia	Escherichia coli
4	Bacteria	Proteobacteria	Gammaproteobacteria	Enterobacterales	Enterobacteriaceae	Escherichia	Escherichia coli
4	Bacteria	Proteobacteria	Gammaproteobacteria	Enterobacterales	Enterobacteriaceae	Escherichia	Escherichia coli
4	Bacteria	Proteobacteria	Gammaproteobacteria	Enterobacterales	Enterobacteriaceae	Escherichia	Escherichia coli
4	Bacteria	Proteobacteria	Gammaproteobacteria	Enterobacterales	Enterobacteriaceae	Escherichia	Escherichia coli
4	Bacteria	Proteobacteria	Gammaproteobacteria	Enterobacterales	Enterobacteriaceae	Escherichia	Escherichia coli
4	Bacteria	Proteobacteria	Gammaproteobacteria	Enterobacterales	Enterobacteriaceae	Escherichia	Escherichia coli
4	Bacteria	Proteobacteria	Gammaproteobacteria	Enterobacterales	Enterobacteriaceae	Escherichia	Escherichia coli
4	Bacteria	Proteobacteria	Gammaproteobacteria	Enterobacterales	Enterobacteriaceae	Escherichia	Escherichia coli
4	Bacteria	Proteobacteria	Gammaproteobacteria	Enterobacterales	Enterobacteriaceae	Escherichia	Escherichia coli
4	Bacteria	Proteobacteria	Gammaproteobacteria	Enterobacterales	Enterobacteriaceae	Escherichia	Escherichia coli
4	Bacteria	Proteobacteria	Gammaproteobacteria	Enterobacterales	Enterobacteriaceae	Escherichia	Escherichia coli
4	Bacteria	Proteobacteria	Gammaproteobacteria	Enterobacterales	Enterobacteriaceae	Escherichia	Escherichia coli
4	Bacteria	Proteobacteria	Gammaproteobacteria	Enterobacterales	Enterobacteriaceae	Escherichia	Escherichia coli
4	Bacteria	Proteobacteria	Gammaproteobacteria	Enterobacterales	Enterobacteriaceae	Escherichia	Escherichia coli
4	Bacteria	Proteobacteria	Gammaproteobacteria	Enterobacterales	Enterobacteriaceae	Escherichia	Escherichia coli
4	Bacteria	Proteobacteria	Gammaproteobacteria	Enterobacterales	Enterobacteriaceae	Escherichia	Escherichia coli
4	Bacteria	Proteobacteria	Gammaproteobacteria	Enterobacterales	Enterobacteriaceae	Escherichia	Escherichia coli
4	Bacteria	Proteobacteria	Gammaproteobacteria	Enterobacterales	Enterobacteriaceae	Escherichia	Escherichia coli
4	Bacteria	Proteobacteria	Gammaproteobacteria	Enterobacterales	Enterobacteriaceae	Escherichia	Escherichia coli
4	Bacteria	Proteobacteria	Gammaproteobacteria	Enterobacterales	Enterobacteriaceae	Escherichia	Escherichia coli
4	Bacteria	Proteobacteria	Gammaproteobacteria	Enterobacterales	Enterobacteriaceae	Escherichia	Escherichia coli
4	Bacteria	Proteobacteria	Gammaproteobacteria	Enterobacterales	Enterobacteriaceae	Escherichia	Escherichia coli
4	Bacteria	Proteobacteria	Gammaproteobacteria	Enterobacterales	Enterobacteriaceae	Escherichia	Escherichia coli
4	Bacteria	Proteobacteria	Gammaproteobacteria	Enterobacterales	Enterobacteriaceae	Escherichia	Escherichia coli
4	Bacteria	Proteobacteria	Gammaproteobacteria	Enterobacterales	Enterobacteriaceae	Escherichia	Escherichia coli
4	Bacteria	Proteobacteria	Gammaproteobacteria	Enterobacterales	Enterobacteriaceae	Escherichia	Escherichia coli
4	Bacteria	Proteobacteria	Gammaproteobacteria	Enterobacterales	Enterobacteriaceae	Escherichia	Escherichia coli
4	Bacteria	Proteobacteria	Gammaproteobacteria	Enterobacterales	Enterobacteriaceae	Escherichia	Escherichia coli
4	Bacteria	Proteobacteria	Gammaproteobacteria	Enterobacterales	Enterobacteriaceae	Enterobacter	Enterobacter sp. Bisph1
4	Bacteria	Proteobacteria	Gammaproteobacteria	Enterobacterales	Enterobacteriaceae	Enterobacter	Enterobacter cloacae complex
4	Bacteria	Proteobacteria	Gammaproteobacteria	Enterobacterales	Enterobacteriaceae	Cronobacter	Cronobacter dublinensis
4	Bacteria	Proteobacteria	Gammaproteobacteria	Enterobacterales	Enterobacteriaceae	Citrobacter	Citrobacter freundii complex
4	Bacteria	Proteobacteria	Gammaproteobacteria	Enterobacterales	Enterobacteriaceae	Citrobacter	Citrobacter freundii complex
4	Bacteria	Proteobacteria	Gammaproteobacteria	Enterobacterales	Enterobacteriaceae	Citrobacter	Citrobacter freundii complex
4	Bacteria	Proteobacteria	Gammaproteobacteria	Enterobacterales	Enterobacteriaceae	Cedecea	Cedecea davisae
4	Bacteria	Proteobacteria	Gammaproteobacteria	Chromatiales	Chromatiaceae	Thiorhodovibrio	Thiorhodovibrio sp. 970
4	Bacteria	Proteobacteria	Gammaproteobacteria	Chromatiales	Chromatiaceae	Rheinheimera	Rheinheimera nanhaiensis
4	Bacteria	Proteobacteria	Gammaproteobacteria	Aeromonadales	Aeromonadaceae	Aeromonas	Aeromonas hydrophila
4	Bacteria	Proteobacteria	Betaproteobacteria	unclassified Betaproteobacteria	unclassified Betaproteobacteria (miscellaneous)	Betaproteobacteria bacterium RIFCSPLOWO2_12_FULL_65_14	
4	Bacteria	Proteobacteria	Betaproteobacteria	unclassified Betaproteobacteria	unclassified Betaproteobacteria (miscellaneous)	Betaproteobacteria bacterium RIFCSPLOWO2_02_FULL_62_17	
4	Bacteria	Proteobacteria	Betaproteobacteria	unclassified Betaproteobacteria	Candidatus Accumulibacter	Candidatus Accumulibacter aalborgensis	
4	Bacteria	Proteobacteria	Betaproteobacteria	Rhodocyclales	Rhodocyclaceae	unclassified Rhodocyclaceae	Rhodocyclaceae bacterium Paddy-1
4	Bacteria	Proteobacteria	Betaproteobacteria	Rhodocyclales	Rhodocyclaceae	Dechloromonas	Dechloromonas aromatica
4	Bacteria	Proteobacteria	Betaproteobacteria	Rhodocyclales	Rhodocyclaceae	Azospira	Azospira oryzae
4	Bacteria	Proteobacteria	Betaproteobacteria	Nitrosomonadales	Nitrosomonadaceae	Nitrosomonas	Nitrosomonas halophila
4	Bacteria	Proteobacteria	Betaproteobacteria	Neisseriales	Chromobacteriaceae		
4	Bacteria	Proteobacteria	Betaproteobacteria	Methylophilales	Methylophilaceae	Methylotenera	Methylotenera mobilis
4	Bacteria	Proteobacteria	Betaproteobacteria	Methylophilales	Methylophilaceae	Methylotenera	
4	Bacteria	Proteobacteria	Betaproteobacteria	Hydrogenophilales	Hydrogenophilaceae	Thiobacillus	Thiobacillus denitrificans
4	Bacteria	Proteobacteria	Betaproteobacteria	Hydrogenophilales	Hydrogenophilaceae	Thiobacillus	
4	Bacteria	Proteobacteria	Betaproteobacteria	Burkholderiales	unclassified Burkholderiales	Burkholderiales Genera incertae sedis	Thiomonas
4	Bacteria	Proteobacteria	Betaproteobacteria	Burkholderiales	unclassified Burkholderiales	Burkholderiales Genera incertae sedis	Methylibium
4	Bacteria	Proteobacteria	Betaproteobacteria	Burkholderiales	Oxalobacteraceae	Massilia	
4	Bacteria	Proteobacteria	Betaproteobacteria	Burkholderiales	Comamonadaceae	unclassified Comamonadaceae	Comamonadaceae bacterium CG1_02_60_18
4	Bacteria	Proteobacteria	Betaproteobacteria	Burkholderiales	Comamonadaceae	Variovorax	Variovorax sp. OV329
4	Bacteria	Proteobacteria	Betaproteobacteria	Burkholderiales	Comamonadaceae	Polaromonas	
4	Bacteria	Proteobacteria	Betaproteobacteria	Burkholderiales	Comamonadaceae	Pelomonas	
4	Bacteria	Proteobacteria	Betaproteobacteria	Burkholderiales	Comamonadaceae	Hydrogenophaga	Hydrogenophaga sp. LPB0072
4	Bacteria	Proteobacteria	Betaproteobacteria	Burkholderiales	Comamonadaceae	Hydrogenophaga	
4	Bacteria	Proteobacteria	Betaproteobacteria	Burkholderiales	Comamonadaceae	Comamonas	Comamonas testosteroni
4	Bacteria	Proteobacteria	Betaproteobacteria	Burkholderiales	Burkholderiaceae	Ralstonia	Ralstonia sp. AU12-08
4	Bacteria	Proteobacteria	Betaproteobacteria	Burkholderiales	Burkholderiaceae	Ralstonia	Ralstonia pickettii
4	Bacteria	Proteobacteria	Betaproteobacteria	Burkholderiales	Burkholderiaceae	Pandoraea	Pandoraea pnomenusa
4	Bacteria	Proteobacteria	Betaproteobacteria	Burkholderiales	Burkholderiaceae	Burkholderia	Burkholderia ubonensis
4	Bacteria	Proteobacteria	Betaproteobacteria	Burkholderiales	Burkholderiaceae	Burkholderia	Burkholderia cepacia complex
4	Bacteria	Proteobacteria	Betaproteobacteria	Burkholderiales	Alcaligenaceae	Bordetella	Bordetella trematum
4	Bacteria	Proteobacteria	Betaproteobacteria	Burkholderiales	Alcaligenaceae	Achromobacter	
4	Bacteria	Proteobacteria	Alphaproteobacteria	unclassified Alphaproteobacteria	unclassified Alphaproteobacteria (miscellaneous)	alpha proteobacterium Mf 1.05b.01	
4	Bacteria	Proteobacteria	Alphaproteobacteria	Sphingomonadales	Sphingomonadaceae	Sphingomonas	Sphingomonas changbaiensis
4	Bacteria	Proteobacteria	Alphaproteobacteria	Sphingomonadales	Sphingomonadaceae	Sphingobium	Sphingobium ummariense
4	Bacteria	Proteobacteria	Alphaproteobacteria	Sphingomonadales	Sphingomonadaceae	Sphingobium	Sphingobium sp. SYK-6
4	Bacteria	Proteobacteria	Alphaproteobacteria	Sphingomonadales	Sphingomonadaceae	Sphingobium	Sphingobium sp. 66-54
4	Bacteria	Proteobacteria	Alphaproteobacteria	Sphingomonadales	Sphingomonadaceae	Sphingobium	Sphingobium baderi
4	Bacteria	Proteobacteria	Alphaproteobacteria	Sphingomonadales	Sphingomonadaceae	Sphingobium	
4	Bacteria	Proteobacteria	Alphaproteobacteria	Sphingomonadales	Sphingomonadaceae	Novosphingobium	Novosphingobium sp. CF614
4	Bacteria	Proteobacteria	Alphaproteobacteria	Sphingomonadales	Sphingomonadaceae	Novosphingobium	Novosphingobium nitrogenifigens
4	Bacteria	Proteobacteria	Alphaproteobacteria	Sphingomonadales	Sphingomonadaceae	Novosphingobium	Novosphingobium naphthalenivorans
4	Bacteria	Proteobacteria	Alphaproteobacteria	Sphingomonadales	Erythrobacteraceae	Erythrobacter	Erythrobacter sp. RIFCSPHIGHO2_12_FULL_63_10
4	Bacteria	Proteobacteria	Alphaproteobacteria	Sphingomonadales	Erythrobacteraceae	Erythrobacter	Erythrobacter nanhaisediminis
4	Bacteria	Proteobacteria	Alphaproteobacteria	Sphingomonadales	Erythrobacteraceae	Erythrobacter	Erythrobacter luteus
4	Bacteria	Proteobacteria	Alphaproteobacteria	Sphingomonadales	Erythrobacteraceae	Erythrobacter	Erythrobacter litoralis
4	Bacteria	Proteobacteria	Alphaproteobacteria	Sphingomonadales	Erythrobacteraceae	Altererythrobacter	Altererythrobacter epoxidivorans
4	Bacteria	Proteobacteria	Alphaproteobacteria	Rickettsiales	unclassified Rickettsiales	unclassified Rickettsiales (miscellaneous)	Rickettsiales bacterium Ac37b
4	Bacteria	Proteobacteria	Alphaproteobacteria	Rickettsiales	Rickettsiaceae	Rickettsia	
4	Bacteria	Proteobacteria	Alphaproteobacteria	Rickettsiales	Anaplasmataceae	Candidatus Neoehrlichia	Candidatus Neoehrlichia lotoris
4	Bacteria	Proteobacteria	Alphaproteobacteria	Rhodospirillales	Rhodospirillaceae	Dongia	Dongia sp. URHE0060
4	Bacteria	Proteobacteria	Alphaproteobacteria	Rhodospirillales	Rhodospirillaceae	Azospirillum	
4	Bacteria	Proteobacteria	Alphaproteobacteria	Rhodobacterales	unclassified Rhodobacterales	Rhodobacterales bacterium 65-51	
4	Bacteria	Proteobacteria	Alphaproteobacteria	Rhodobacterales	Rhodobacteraceae	unclassified Rhodobacteraceae	
4	Bacteria	Proteobacteria	Alphaproteobacteria	Rhodobacterales	Rhodobacteraceae	Wenxinia	Wenxinia marina
4	Bacteria	Proteobacteria	Alphaproteobacteria	Rhodobacterales	Rhodobacteraceae	Ruegeria	
4	Bacteria	Proteobacteria	Alphaproteobacteria	Rhodobacterales	Rhodobacteraceae	Rhodobacter	Rhodobacter sphaeroides
4	Bacteria	Proteobacteria	Alphaproteobacteria	Rhodobacterales	Rhodobacteraceae	Rhodobacter	
4	Bacteria	Proteobacteria	Alphaproteobacteria	Rhodobacterales	Rhodobacteraceae	Nesiotobacter	Nesiotobacter exalbescens
4	Bacteria	Proteobacteria	Alphaproteobacteria	Rhizobiales	unclassified Rhizobiales	unclassified Rhizobiales (miscellaneous)	Rhizobiales bacterium YIM 77505
4	Bacteria	Proteobacteria	Alphaproteobacteria	Rhizobiales	unclassified Rhizobiales	Pseudorhodoplanes	Pseudorhodoplanes sinuspersici
4	Bacteria	Proteobacteria	Alphaproteobacteria	Rhizobiales	Xanthobacteraceae	Pseudolabrys	Pseudolabrys sp. Root1462
4	Bacteria	Proteobacteria	Alphaproteobacteria	Rhizobiales	Xanthobacteraceae	Azorhizobium	Azorhizobium doebereinerae
4	Bacteria	Proteobacteria	Alphaproteobacteria	Rhizobiales	Phyllobacteriaceae	Phyllobacterium	
4	Bacteria	Proteobacteria	Alphaproteobacteria	Rhizobiales	Phyllobacteriaceae	Mesorhizobium	Mesorhizobium metallidurans
4	Bacteria	Proteobacteria	Alphaproteobacteria	Rhizobiales	Phyllobacteriaceae	Aminobacter	Aminobacter sp. J41
4	Bacteria	Proteobacteria	Alphaproteobacteria	Rhizobiales	Methylocystaceae	Methylosinus	
4	Bacteria	Proteobacteria	Alphaproteobacteria	Rhizobiales	Aurantimonadaceae	Aureimonas	
4	Bacteria	PVC group	Planctomycetes	Planctomycetia	Planctomycetales	Planctomycetaceae	Rubripirellula
4	Bacteria	PVC group	Planctomycetes	Planctomycetia	Planctomycetales	Planctomycetaceae	Rhodopirellula
4	Bacteria	Nitrospirae	unclassified Nitrospirae				
4	Bacteria	Nitrospirae	Nitrospira	Nitrospirales	Nitrospiraceae	Nitrospira	Candidatus Nitrospira inopinata
4	Bacteria	FCB group	Bacteroidetes/Chlorobi group	Ignavibacteriae	Ignavibacteria	unclassified Ignavibacteria	
4	Bacteria	FCB group	Bacteroidetes/Chlorobi group	Ignavibacteriae	Ignavibacteria	Ignavibacteriales	Melioribacteraceae
4	Bacteria	FCB group	Bacteroidetes/Chlorobi group	Chlorobi	unclassified Chlorobi	Chlorobi bacterium OLB5	
4	Bacteria	FCB group	Bacteroidetes/Chlorobi group	Bacteroidetes	unclassified Bacteroidetes	unclassified Bacteroidetes (miscellaneous)	Bacteroidetes bacterium GWF2_41_31
4	Bacteria	FCB group	Bacteroidetes/Chlorobi group	Bacteroidetes	unclassified Bacteroidetes	unclassified Bacteroidetes (miscellaneous)	Bacteroidetes bacterium GWB2_41_8
4	Bacteria	FCB group	Bacteroidetes/Chlorobi group	Bacteroidetes	unclassified Bacteroidetes	unclassified Bacteroidetes (miscellaneous)	Bacteroidetes bacterium CG2_30_32_10
4	Bacteria	FCB group	Bacteroidetes/Chlorobi group	Bacteroidetes	Sphingobacteriia	Sphingobacteriales	unclassified Sphingobacteriales
4	Bacteria	FCB group	Bacteroidetes/Chlorobi group	Bacteroidetes	Sphingobacteriia	Sphingobacteriales	Sphingobacteriaceae
4	Bacteria	FCB group	Bacteroidetes/Chlorobi group	Bacteroidetes	Sphingobacteriia	Sphingobacteriales	Sphingobacteriaceae
4	Bacteria	FCB group	Bacteroidetes/Chlorobi group	Bacteroidetes	Saprospiria	Saprospirales	Haliscomenobacteraceae
4	Bacteria	FCB group	Bacteroidetes/Chlorobi group	Bacteroidetes	Flavobacteriia	Flavobacteriales	Flavobacteriaceae
4	Bacteria	FCB group	Bacteroidetes/Chlorobi group	Bacteroidetes	Cytophagia	Cytophagales	Thermonemataceae
4	Bacteria	FCB group	Bacteroidetes/Chlorobi group	Bacteroidetes	Cytophagia	Cytophagales	Hymenobacteraceae
4	Bacteria	FCB group	Bacteroidetes/Chlorobi group	Bacteroidetes	Cytophagia	Cytophagales	Cytophagaceae
4	Bacteria	FCB group	Bacteroidetes/Chlorobi group	Bacteroidetes	Cytophagia	Cytophagales	Cytophagaceae
4	Bacteria	FCB group	Bacteroidetes/Chlorobi group	Bacteroidetes	Chitinophagia	Chitinophagales	Chitinophagaceae
4	Bacteria	FCB group	Bacteroidetes/Chlorobi group	Bacteroidetes	Bacteroidia	Marinilabiliales	Prolixibacteraceae
4	Bacteria	FCB group	Bacteroidetes/Chlorobi group	Bacteroidetes	Bacteroidia	Bacteroidales	Rikenellaceae
4	Bacteria	FCB group	Bacteroidetes/Chlorobi group	Bacteroidetes	Bacteroidia	Bacteroidales	Rikenellaceae
4	Bacteria	FCB group	Bacteroidetes/Chlorobi group	Bacteroidetes	Bacteroidia	Bacteroidales	Rikenellaceae
4	Bacteria	FCB group	Bacteroidetes/Chlorobi group	Bacteroidetes	Bacteroidia	Bacteroidales	Prevotellaceae
4	Bacteria	FCB group	Bacteroidetes/Chlorobi group	Bacteroidetes	Bacteroidia	Bacteroidales	Prevotellaceae
4	Bacteria	FCB group	Bacteroidetes/Chlorobi group	Bacteroidetes	Bacteroidia	Bacteroidales	Porphyromonadaceae
4	Bacteria	FCB group	Bacteroidetes/Chlorobi group	Bacteroidetes	Bacteroidia	Bacteroidales	Porphyromonadaceae
4	Bacteria	FCB group	Bacteroidetes/Chlorobi group	Bacteroidetes	Bacteroidia	Bacteroidales	Bacteroidaceae
4	Bacteria	FCB group	Bacteroidetes/Chlorobi group	Bacteroidetes	Bacteroidia	Bacteroidales	Bacteroidaceae
4	Bacteria	FCB group	Bacteroidetes/Chlorobi group	Bacteroidetes	Bacteroidia	Bacteroidales	Bacteroidaceae
4	Bacteria	Elusimicrobia	unclassified Elusimicrobia				
4	ssDNA viruses	Inoviridae	unassigned Inoviridae	Vibrio phage KSF-1phi			
4	dsDNA viruses, no RNA stage	Phycodnaviridae	unclassified Phycodnaviridae	Yellowstone lake phycodnavirus 1			
4	dsDNA viruses, no RNA stage	Caudovirales	Myoviridae	unclassified Myoviridae	Aeromonas phage vB_AsaM-56		
3	Eukaryota	Viridiplantae	Chlorophyta				
3	Eukaryota	Rhodophyta	Florideophyceae	Gigartinales	Solieriaceae	Solieria	Solieria pacifica
3	Eukaryota	Rhodophyta	Florideophyceae	Gigartinales	Gigartinaceae	Chondrus	Chondrus crispus
3	Eukaryota	Opisthokonta	Fungi	Dikarya	Basidiomycota	Pucciniomycotina	Microbotryomycetes
3	Eukaryota	Opisthokonta	Fungi	Dikarya	Basidiomycota	Agaricomycotina	Agaricomycetes
3	Eukaryota	Opisthokonta	Fungi	Dikarya	Ascomycota	saccharomyceta	Pezizomycotina
3	Eukaryota	Opisthokonta	Choanoflagellida	Craspedida	Salpingoecidae	Salpingoeca	Salpingoeca rosetta
3	Eukaryota	Malawimonadidae	Malawimonas	Malawimonas sp. strain 249			
3	Eukaryota	Alveolata	Dinophyceae	Suessiales	Symbiodiniaceae	Symbiodinium	Symbiodinium microadriaticum
3	Eukaryota	Alveolata	Ciliophora	Intramacronucleata	Spirotrichea	Stichotrichia	Sporadotrichida
3	Eukaryota	Alveolata	Apicomplexa	Conoidasida	Coccidia	Eucoccidiorida	Eimeriorina
3	Bacteria	unclassified Bacteria	Candidatus Vecturithrix	Candidatus Vecturithrix granuli			
3	Bacteria	unclassified Bacteria	Bacteria candidate phyla	candidate division CPR1	candidate division CPR1 bacterium GW2011_GWA2_42_17		
3	Bacteria	unclassified Bacteria	Bacteria candidate phyla	Patescibacteria group	Parcubacteria group		
3	Bacteria	Terrabacteria group	Tenericutes	Mollicutes	Entomoplasmatales	unclassified Entomoplasmatales	Entomoplasmatales bacterium EntAcro10
3	Bacteria	Terrabacteria group	Firmicutes	Negativicutes	Selenomonadales	Sporomusaceae	Propionispora
3	Bacteria	Terrabacteria group	Firmicutes	Clostridia	Thermoanaerobacterales	Thermoanaerobacteraceae	Thermacetogenium
3	Bacteria	Terrabacteria group	Firmicutes	Clostridia	Clostridiales	Syntrophomonadaceae	Syntrophomonas
3	Bacteria	Terrabacteria group	Firmicutes	Clostridia	Clostridiales	Peptostreptococcaceae	
3	Bacteria	Terrabacteria group	Firmicutes	Clostridia	Clostridiales	Lachnospiraceae	Robinsoniella
3	Bacteria	Terrabacteria group	Firmicutes	Clostridia	Clostridiales	Lachnospiraceae	Blautia
3	Bacteria	Terrabacteria group	Firmicutes	Clostridia	Clostridiales	Eubacteriaceae	Eubacterium
3	Bacteria	Terrabacteria group	Firmicutes	Clostridia	Clostridiales	Clostridiaceae	Clostridium
3	Bacteria	Terrabacteria group	Firmicutes	Clostridia	Clostridiales	Clostridiaceae	Clostridium
3	Bacteria	Terrabacteria group	Firmicutes	Clostridia	Clostridiales	Clostridiaceae	Clostridium
3	Bacteria	Terrabacteria group	Firmicutes	Clostridia	Clostridiales	Clostridiaceae	Clostridium
3	Bacteria	Terrabacteria group	Firmicutes	Clostridia	Clostridiales	Clostridiaceae	
3	Bacteria	Terrabacteria group	Firmicutes	Bacilli	Lactobacillales	Enterococcaceae	Enterococcus
3	Bacteria	Terrabacteria group	Firmicutes	Bacilli	Bacillales	Staphylococcaceae	Staphylococcus
3	Bacteria	Terrabacteria group	Firmicutes	Bacilli	Bacillales	Paenibacillaceae	Cohnella
3	Bacteria	Terrabacteria group	Firmicutes	Bacilli	Bacillales	Bacillaceae	Bacillus
3	Bacteria	Terrabacteria group	Chloroflexi	Ardenticatenia	Ardenticatenales	Ardenticatenaceae	Ardenticatena
3	Bacteria	Terrabacteria group	Chloroflexi	Anaerolineae	unclassified Anaerolineae	Anaerolineae bacterium SG8_19	
3	Bacteria	Terrabacteria group	Chloroflexi				
3	Bacteria	Terrabacteria group	Armatimonadetes	Fimbriimonadia	Fimbriimonadales	Fimbriimonadaceae	Fimbriimonas
3	Bacteria	Terrabacteria group	Actinobacteria	Actinobacteria	Propionibacteriales	Propionibacteriaceae	Granulicoccus
3	Bacteria	Terrabacteria group	Actinobacteria	Actinobacteria	Micromonosporales	Micromonosporaceae	Dactylosporangium
3	Bacteria	Terrabacteria group	Actinobacteria	Acidimicrobiia	Acidimicrobiales	Microthrixaceae	Candidatus Microthrix
3	Bacteria	Synergistetes	Synergistia	Synergistales	unclassified Synergistales		
3	Bacteria	Synergistetes	Synergistia	Synergistales	Synergistaceae	Thermovirga	Thermovirga lienii
3	Bacteria	Synergistetes	Synergistia	Synergistales	Synergistaceae	Thermanaerovibrio	Thermanaerovibrio velox
3	Bacteria	Synergistetes	Synergistia	Synergistales	Synergistaceae	Thermanaerovibrio	Thermanaerovibrio acidaminovorans
3	Bacteria	Synergistetes	Synergistia	Synergistales	Synergistaceae	Aminiphilus	Aminiphilus circumscriptus
3	Bacteria	Proteobacteria	delta/epsilon subdivisions	Deltaproteobacteria	unclassified Deltaproteobacteria	unclassified Deltaproteobacteria (miscellaneous)	Deltaproteobacteria bacterium RIFOXYD12_FULL_56_24
3	Bacteria	Proteobacteria	delta/epsilon subdivisions	Deltaproteobacteria	unclassified Deltaproteobacteria	unclassified Deltaproteobacteria (miscellaneous)	Deltaproteobacteria bacterium RBG_13_43_22
3	Bacteria	Proteobacteria	delta/epsilon subdivisions	Deltaproteobacteria	Syntrophobacterales	Syntrophaceae	Desulfomonile
3	Bacteria	Proteobacteria	delta/epsilon subdivisions	Deltaproteobacteria	Myxococcales	Sorangiineae	Polyangiaceae
3	Bacteria	Proteobacteria	delta/epsilon subdivisions	Deltaproteobacteria	Desulfovibrionales	Desulfovibrionaceae	Desulfovibrio
3	Bacteria	Proteobacteria	delta/epsilon subdivisions	Deltaproteobacteria	Desulfovibrionales	Desulfovibrionaceae	Desulfovibrio
3	Bacteria	Proteobacteria	delta/epsilon subdivisions	Deltaproteobacteria	Desulfovibrionales	Desulfovibrionaceae	Desulfovibrio
3	Bacteria	Proteobacteria	delta/epsilon subdivisions	Deltaproteobacteria	Desulfovibrionales	Desulfovibrionaceae	Desulfovibrio
3	Bacteria	Proteobacteria	delta/epsilon subdivisions	Deltaproteobacteria	Desulfovibrionales	Desulfovibrionaceae	Desulfovibrio
3	Bacteria	Proteobacteria	delta/epsilon subdivisions	Deltaproteobacteria	Desulfovibrionales	Desulfonatronaceae	Desulfonatronum
3	Bacteria	Proteobacteria	delta/epsilon subdivisions	Deltaproteobacteria	Desulfovibrionales	Desulfomicrobiaceae	Desulfomicrobium
3	Bacteria	Proteobacteria	delta/epsilon subdivisions	Deltaproteobacteria	Desulfovibrionales	Desulfomicrobiaceae	Desulfomicrobium
3	Bacteria	Proteobacteria	delta/epsilon subdivisions	Deltaproteobacteria	Desulfovibrionales	Desulfomicrobiaceae	Desulfomicrobium
3	Bacteria	Proteobacteria	delta/epsilon subdivisions	Deltaproteobacteria	Desulfobacterales	Desulfobulbaceae	Desulfofustis
3	Bacteria	Proteobacteria	delta/epsilon subdivisions	Deltaproteobacteria	Desulfobacterales	Desulfobacteraceae	Desulfobacterium
3	Bacteria	Proteobacteria	delta/epsilon subdivisions	Deltaproteobacteria	Desulfobacterales	Desulfobacteraceae	Candidatus Magnetoglobus
3	Bacteria	Proteobacteria	delta/epsilon subdivisions	Deltaproteobacteria			
3	Bacteria	Proteobacteria	Gammaproteobacteria	unclassified Gammaproteobacteria	unclassified Gammaproteobacteria (miscellaneous)	Gammaproteobacteria bacterium SG8_31	
3	Bacteria	Proteobacteria	Gammaproteobacteria	unclassified Gammaproteobacteria	unclassified Gammaproteobacteria (miscellaneous)	Gammaproteobacteria bacterium RIFCSPLOWO2_02_FULL_61_13	
3	Bacteria	Proteobacteria	Gammaproteobacteria	unclassified Gammaproteobacteria	sulfur-oxidizing symbionts	Solemya elarraichensis gill symbiont	
3	Bacteria	Proteobacteria	Gammaproteobacteria	unclassified Gammaproteobacteria	sulfur-oxidizing symbionts	Candidatus Thiodiazotropha	Candidatus Thiodiazotropha endolucinida
3	Bacteria	Proteobacteria	Gammaproteobacteria	unclassified Gammaproteobacteria	Competibacteraceae	Candidatus Contendobacter	Candidatus Contendobacter odensis
3	Bacteria	Proteobacteria	Gammaproteobacteria	Xanthomonadales	Rhodanobacteraceae	Dokdonella	Dokdonella koreensis
3	Bacteria	Proteobacteria	Gammaproteobacteria	Vibrionales	Vibrionaceae	Vibrio	Vibrio xiamenensis
3	Bacteria	Proteobacteria	Gammaproteobacteria	Vibrionales	Vibrionaceae	Vibrio	Vibrio toranzoniae
3	Bacteria	Proteobacteria	Gammaproteobacteria	Vibrionales	Vibrionaceae	Vibrio	Vibrio sp. J2-29
3	Bacteria	Proteobacteria	Gammaproteobacteria	Vibrionales	Vibrionaceae	Vibrio	Vibrio sp. HENC-01
3	Bacteria	Proteobacteria	Gammaproteobacteria	Vibrionales	Vibrionaceae	Vibrio	Vibrio sp. ECSMB14106
3	Bacteria	Proteobacteria	Gammaproteobacteria	Vibrionales	Vibrionaceae	Vibrio	Vibrio sp. CECT 9027
3	Bacteria	Proteobacteria	Gammaproteobacteria	Vibrionales	Vibrionaceae	Vibrio	Vibrio sp. 712i1
3	Bacteria	Proteobacteria	Gammaproteobacteria	Vibrionales	Vibrionaceae	Vibrio	Vibrio maritimus
3	Bacteria	Proteobacteria	Gammaproteobacteria	Vibrionales	Vibrionaceae	Vibrio	Vibrio harveyi group
3	Bacteria	Proteobacteria	Gammaproteobacteria	Vibrionales	Vibrionaceae	Vibrio	Vibrio gazogenes
3	Bacteria	Proteobacteria	Gammaproteobacteria	Vibrionales	Vibrionaceae	Vibrio	Vibrio cholerae
3	Bacteria	Proteobacteria	Gammaproteobacteria	Vibrionales	Vibrionaceae	Vibrio	Vibrio cholerae
3	Bacteria	Proteobacteria	Gammaproteobacteria	Vibrionales	Vibrionaceae	Vibrio	Vibrio cholerae
3	Bacteria	Proteobacteria	Gammaproteobacteria	Vibrionales	Vibrionaceae	Vibrio	Vibrio cholerae
3	Bacteria	Proteobacteria	Gammaproteobacteria	Vibrionales	Vibrionaceae	Photobacterium	Photobacterium sp. SKA34
3	Bacteria	Proteobacteria	Gammaproteobacteria	Vibrionales	Vibrionaceae	Photobacterium	Photobacterium gaetbulicola
3	Bacteria	Proteobacteria	Gammaproteobacteria	Pseudomonadales	Pseudomonadaceae	Pseudomonas	Pseudomonas syringae group
3	Bacteria	Proteobacteria	Gammaproteobacteria	Pseudomonadales	Pseudomonadaceae	Pseudomonas	Pseudomonas putida group
3	Bacteria	Proteobacteria	Gammaproteobacteria	Pseudomonadales	Pseudomonadaceae	Pseudomonas	Pseudomonas peli
3	Bacteria	Proteobacteria	Gammaproteobacteria	Pseudomonadales	Pseudomonadaceae	Pseudomonas	Pseudomonas guineae
3	Bacteria	Proteobacteria	Gammaproteobacteria	Pseudomonadales	Pseudomonadaceae	Pseudomonas	Pseudomonas chlororaphis group
3	Bacteria	Proteobacteria	Gammaproteobacteria	Pseudomonadales	Pseudomonadaceae	Azotobacter group	Azotobacter
3	Bacteria	Proteobacteria	Gammaproteobacteria	Pseudomonadales	Moraxellaceae	Acinetobacter	Acinetobacter calcoaceticus/baumannii complex
3	Bacteria	Proteobacteria	Gammaproteobacteria	Pasteurellales	Pasteurellaceae	Haemophilus	Haemophilus parainfluenzae
3	Bacteria	Proteobacteria	Gammaproteobacteria	Pasteurellales	Pasteurellaceae	Gallibacterium	Gallibacterium anatis
3	Bacteria	Proteobacteria	Gammaproteobacteria	Oceanospirillales	unclassified Oceanospirillales	unclassified Oceanospirillales (miscellaneous)	Oceanospirillales bacterium LUC14_002_19_P2
3	Bacteria	Proteobacteria	Gammaproteobacteria	Oceanospirillales	Oceanospirillaceae	Neptunomonas	Neptunomonas phycophila
3	Bacteria	Proteobacteria	Gammaproteobacteria	Oceanospirillales	Oceanospirillaceae	Marinomonas	Marinomonas spartinae
3	Bacteria	Proteobacteria	Gammaproteobacteria	Oceanospirillales	Halomonadaceae	Halomonas	Halomonas sp. R57-5
3	Bacteria	Proteobacteria	Gammaproteobacteria	Oceanospirillales	Alcanivoracaceae	Alcanivorax	Alcanivorax pacificus
3	Bacteria	Proteobacteria	Gammaproteobacteria	Oceanospirillales	Alcanivoracaceae	Alcanivorax	
3	Bacteria	Proteobacteria	Gammaproteobacteria	Methylococcales	Methylococcaceae	Methylomonas	
3	Bacteria	Proteobacteria	Gammaproteobacteria	Methylococcales	Methylococcaceae	Methylomicrobium	
3	Bacteria	Proteobacteria	Gammaproteobacteria	Enterobacterales	Yersiniaceae	Yersinia	Yersinia pseudotuberculosis complex
3	Bacteria	Proteobacteria	Gammaproteobacteria	Enterobacterales	Yersiniaceae	Yersinia	Yersinia frederiksenii
3	Bacteria	Proteobacteria	Gammaproteobacteria	Enterobacterales	Yersiniaceae	Serratia	Serratia marcescens
3	Bacteria	Proteobacteria	Gammaproteobacteria	Enterobacterales	Pectobacteriaceae	Dickeya	Dickeya zeae
3	Bacteria	Proteobacteria	Gammaproteobacteria	Enterobacterales	Morganellaceae	Providencia	Providencia stuartii
3	Bacteria	Proteobacteria	Gammaproteobacteria	Enterobacterales	Morganellaceae	Proteus	Proteus mirabilis
3	Bacteria	Proteobacteria	Gammaproteobacteria	Enterobacterales	Hafniaceae	Edwardsiella	Edwardsiella tarda
3	Bacteria	Proteobacteria	Gammaproteobacteria	Enterobacterales	Erwiniaceae	Erwinia	Erwinia tracheiphila
3	Bacteria	Proteobacteria	Gammaproteobacteria	Enterobacterales	Erwiniaceae	Erwinia	Erwinia teleogrylli
3	Bacteria	Proteobacteria	Gammaproteobacteria	Enterobacterales	Erwiniaceae	Erwinia	
3	Bacteria	Proteobacteria	Gammaproteobacteria	Enterobacterales	Enterobacteriaceae	Shigella	Shigella sp. SF-2015
3	Bacteria	Proteobacteria	Gammaproteobacteria	Enterobacterales	Enterobacteriaceae	Shigella	Shigella flexneri
3	Bacteria	Proteobacteria	Gammaproteobacteria	Enterobacterales	Enterobacteriaceae	Salmonella	Salmonella enterica
3	Bacteria	Proteobacteria	Gammaproteobacteria	Enterobacterales	Enterobacteriaceae	Salmonella	Salmonella enterica
3	Bacteria	Proteobacteria	Gammaproteobacteria	Enterobacterales	Enterobacteriaceae	Salmonella	Salmonella enterica
3	Bacteria	Proteobacteria	Gammaproteobacteria	Enterobacterales	Enterobacteriaceae	Salmonella	Salmonella enterica
3	Bacteria	Proteobacteria	Gammaproteobacteria	Enterobacterales	Enterobacteriaceae	Salmonella	Salmonella enterica
3	Bacteria	Proteobacteria	Gammaproteobacteria	Enterobacterales	Enterobacteriaceae	Salmonella	Salmonella enterica
3	Bacteria	Proteobacteria	Gammaproteobacteria	Enterobacterales	Enterobacteriaceae	Salmonella	Salmonella enterica
3	Bacteria	Proteobacteria	Gammaproteobacteria	Enterobacterales	Enterobacteriaceae	Salmonella	Salmonella enterica
3	Bacteria	Proteobacteria	Gammaproteobacteria	Enterobacterales	Enterobacteriaceae	Salmonella	Salmonella enterica
3	Bacteria	Proteobacteria	Gammaproteobacteria	Enterobacterales	Enterobacteriaceae	Salmonella	Salmonella enterica
3	Bacteria	Proteobacteria	Gammaproteobacteria	Enterobacterales	Enterobacteriaceae	Salmonella	Salmonella enterica
3	Bacteria	Proteobacteria	Gammaproteobacteria	Enterobacterales	Enterobacteriaceae	Kluyvera	Kluyvera georgiana
3	Bacteria	Proteobacteria	Gammaproteobacteria	Enterobacterales	Enterobacteriaceae	Klebsiella	Klebsiella sp. 4_1_44FAA
3	Bacteria	Proteobacteria	Gammaproteobacteria	Enterobacterales	Enterobacteriaceae	Klebsiella	Klebsiella pneumoniae
3	Bacteria	Proteobacteria	Gammaproteobacteria	Enterobacterales	Enterobacteriaceae	Klebsiella	Klebsiella pneumoniae
3	Bacteria	Proteobacteria	Gammaproteobacteria	Enterobacterales	Enterobacteriaceae	Klebsiella	Klebsiella pneumoniae
3	Bacteria	Proteobacteria	Gammaproteobacteria	Enterobacterales	Enterobacteriaceae	Klebsiella	Klebsiella pneumoniae
3	Bacteria	Proteobacteria	Gammaproteobacteria	Enterobacterales	Enterobacteriaceae	Klebsiella	Klebsiella pneumoniae
3	Bacteria	Proteobacteria	Gammaproteobacteria	Enterobacterales	Enterobacteriaceae	Klebsiella	Klebsiella pneumoniae
3	Bacteria	Proteobacteria	Gammaproteobacteria	Enterobacterales	Enterobacteriaceae	Klebsiella	Klebsiella aerogenes
3	Bacteria	Proteobacteria	Gammaproteobacteria	Enterobacterales	Enterobacteriaceae	Franconibacter	Franconibacter helveticus
3	Bacteria	Proteobacteria	Gammaproteobacteria	Enterobacterales	Enterobacteriaceae	Escherichia	Escherichia vulneris
3	Bacteria	Proteobacteria	Gammaproteobacteria	Enterobacterales	Enterobacteriaceae	Escherichia	Escherichia sp. 3_2_53FAA
3	Bacteria	Proteobacteria	Gammaproteobacteria	Enterobacterales	Enterobacteriaceae	Escherichia	Escherichia coli
3	Bacteria	Proteobacteria	Gammaproteobacteria	Enterobacterales	Enterobacteriaceae	Escherichia	Escherichia coli
3	Bacteria	Proteobacteria	Gammaproteobacteria	Enterobacterales	Enterobacteriaceae	Escherichia	Escherichia coli
3	Bacteria	Proteobacteria	Gammaproteobacteria	Enterobacterales	Enterobacteriaceae	Escherichia	Escherichia coli
3	Bacteria	Proteobacteria	Gammaproteobacteria	Enterobacterales	Enterobacteriaceae	Escherichia	Escherichia coli
3	Bacteria	Proteobacteria	Gammaproteobacteria	Enterobacterales	Enterobacteriaceae	Escherichia	Escherichia coli
3	Bacteria	Proteobacteria	Gammaproteobacteria	Enterobacterales	Enterobacteriaceae	Escherichia	Escherichia coli
3	Bacteria	Proteobacteria	Gammaproteobacteria	Enterobacterales	Enterobacteriaceae	Escherichia	Escherichia coli
3	Bacteria	Proteobacteria	Gammaproteobacteria	Enterobacterales	Enterobacteriaceae	Escherichia	Escherichia coli
3	Bacteria	Proteobacteria	Gammaproteobacteria	Enterobacterales	Enterobacteriaceae	Escherichia	Escherichia coli
3	Bacteria	Proteobacteria	Gammaproteobacteria	Enterobacterales	Enterobacteriaceae	Escherichia	Escherichia coli
3	Bacteria	Proteobacteria	Gammaproteobacteria	Enterobacterales	Enterobacteriaceae	Escherichia	Escherichia coli
3	Bacteria	Proteobacteria	Gammaproteobacteria	Enterobacterales	Enterobacteriaceae	Escherichia	Escherichia coli
3	Bacteria	Proteobacteria	Gammaproteobacteria	Enterobacterales	Enterobacteriaceae	Escherichia	Escherichia coli
3	Bacteria	Proteobacteria	Gammaproteobacteria	Enterobacterales	Enterobacteriaceae	Escherichia	Escherichia coli
3	Bacteria	Proteobacteria	Gammaproteobacteria	Enterobacterales	Enterobacteriaceae	Escherichia	Escherichia coli
3	Bacteria	Proteobacteria	Gammaproteobacteria	Enterobacterales	Enterobacteriaceae	Escherichia	Escherichia coli
3	Bacteria	Proteobacteria	Gammaproteobacteria	Enterobacterales	Enterobacteriaceae	Escherichia	Escherichia coli
3	Bacteria	Proteobacteria	Gammaproteobacteria	Enterobacterales	Enterobacteriaceae	Escherichia	Escherichia coli
3	Bacteria	Proteobacteria	Gammaproteobacteria	Enterobacterales	Enterobacteriaceae	Escherichia	Escherichia coli
3	Bacteria	Proteobacteria	Gammaproteobacteria	Enterobacterales	Enterobacteriaceae	Escherichia	Escherichia coli
3	Bacteria	Proteobacteria	Gammaproteobacteria	Enterobacterales	Enterobacteriaceae	Escherichia	Escherichia coli
3	Bacteria	Proteobacteria	Gammaproteobacteria	Enterobacterales	Enterobacteriaceae	Escherichia	Escherichia coli
3	Bacteria	Proteobacteria	Gammaproteobacteria	Enterobacterales	Enterobacteriaceae	Escherichia	Escherichia coli
3	Bacteria	Proteobacteria	Gammaproteobacteria	Enterobacterales	Enterobacteriaceae	Escherichia	Escherichia coli
3	Bacteria	Proteobacteria	Gammaproteobacteria	Enterobacterales	Enterobacteriaceae	Escherichia	Escherichia coli
3	Bacteria	Proteobacteria	Gammaproteobacteria	Enterobacterales	Enterobacteriaceae	Escherichia	Escherichia coli
3	Bacteria	Proteobacteria	Gammaproteobacteria	Enterobacterales	Enterobacteriaceae	Escherichia	Escherichia coli
3	Bacteria	Proteobacteria	Gammaproteobacteria	Enterobacterales	Enterobacteriaceae	Escherichia	Escherichia coli
3	Bacteria	Proteobacteria	Gammaproteobacteria	Enterobacterales	Enterobacteriaceae	Escherichia	Escherichia coli
3	Bacteria	Proteobacteria	Gammaproteobacteria	Enterobacterales	Enterobacteriaceae	Escherichia	Escherichia coli
3	Bacteria	Proteobacteria	Gammaproteobacteria	Enterobacterales	Enterobacteriaceae	Escherichia	Escherichia coli
3	Bacteria	Proteobacteria	Gammaproteobacteria	Enterobacterales	Enterobacteriaceae	Escherichia	Escherichia coli
3	Bacteria	Proteobacteria	Gammaproteobacteria	Enterobacterales	Enterobacteriaceae	Escherichia	Escherichia coli
3	Bacteria	Proteobacteria	Gammaproteobacteria	Enterobacterales	Enterobacteriaceae	Escherichia	Escherichia coli
3	Bacteria	Proteobacteria	Gammaproteobacteria	Enterobacterales	Enterobacteriaceae	Escherichia	Escherichia coli
3	Bacteria	Proteobacteria	Gammaproteobacteria	Enterobacterales	Enterobacteriaceae	Escherichia	Escherichia coli
3	Bacteria	Proteobacteria	Gammaproteobacteria	Enterobacterales	Enterobacteriaceae	Escherichia	Escherichia coli
3	Bacteria	Proteobacteria	Gammaproteobacteria	Enterobacterales	Enterobacteriaceae	Escherichia	Escherichia coli
3	Bacteria	Proteobacteria	Gammaproteobacteria	Enterobacterales	Enterobacteriaceae	Escherichia	Escherichia coli
3	Bacteria	Proteobacteria	Gammaproteobacteria	Enterobacterales	Enterobacteriaceae	Escherichia	Escherichia coli
3	Bacteria	Proteobacteria	Gammaproteobacteria	Enterobacterales	Enterobacteriaceae	Escherichia	Escherichia coli
3	Bacteria	Proteobacteria	Gammaproteobacteria	Enterobacterales	Enterobacteriaceae	Escherichia	Escherichia coli
3	Bacteria	Proteobacteria	Gammaproteobacteria	Enterobacterales	Enterobacteriaceae	Escherichia	Escherichia coli
3	Bacteria	Proteobacteria	Gammaproteobacteria	Enterobacterales	Enterobacteriaceae	Escherichia	Escherichia coli
3	Bacteria	Proteobacteria	Gammaproteobacteria	Enterobacterales	Enterobacteriaceae	Escherichia	Escherichia coli
3	Bacteria	Proteobacteria	Gammaproteobacteria	Enterobacterales	Enterobacteriaceae	Escherichia	Escherichia coli
3	Bacteria	Proteobacteria	Gammaproteobacteria	Enterobacterales	Enterobacteriaceae	Escherichia	Escherichia coli
3	Bacteria	Proteobacteria	Gammaproteobacteria	Enterobacterales	Enterobacteriaceae	Escherichia	Escherichia coli
3	Bacteria	Proteobacteria	Gammaproteobacteria	Enterobacterales	Enterobacteriaceae	Escherichia	Escherichia coli
3	Bacteria	Proteobacteria	Gammaproteobacteria	Enterobacterales	Enterobacteriaceae	Escherichia	Escherichia coli
3	Bacteria	Proteobacteria	Gammaproteobacteria	Enterobacterales	Enterobacteriaceae	Escherichia	Escherichia coli
3	Bacteria	Proteobacteria	Gammaproteobacteria	Enterobacterales	Enterobacteriaceae	Escherichia	Escherichia coli
3	Bacteria	Proteobacteria	Gammaproteobacteria	Enterobacterales	Enterobacteriaceae	Escherichia	Escherichia coli
3	Bacteria	Proteobacteria	Gammaproteobacteria	Enterobacterales	Enterobacteriaceae	Escherichia	Escherichia coli
3	Bacteria	Proteobacteria	Gammaproteobacteria	Enterobacterales	Enterobacteriaceae	Escherichia	Escherichia coli
3	Bacteria	Proteobacteria	Gammaproteobacteria	Enterobacterales	Enterobacteriaceae	Escherichia	Escherichia coli
3	Bacteria	Proteobacteria	Gammaproteobacteria	Enterobacterales	Enterobacteriaceae	Escherichia	Escherichia coli
3	Bacteria	Proteobacteria	Gammaproteobacteria	Enterobacterales	Enterobacteriaceae	Escherichia	Escherichia coli
3	Bacteria	Proteobacteria	Gammaproteobacteria	Enterobacterales	Enterobacteriaceae	Escherichia	Escherichia coli
3	Bacteria	Proteobacteria	Gammaproteobacteria	Enterobacterales	Enterobacteriaceae	Escherichia	Escherichia coli
3	Bacteria	Proteobacteria	Gammaproteobacteria	Enterobacterales	Enterobacteriaceae	Escherichia	Escherichia coli
3	Bacteria	Proteobacteria	Gammaproteobacteria	Enterobacterales	Enterobacteriaceae	Escherichia	Escherichia coli
3	Bacteria	Proteobacteria	Gammaproteobacteria	Enterobacterales	Enterobacteriaceae	Escherichia	Escherichia coli
3	Bacteria	Proteobacteria	Gammaproteobacteria	Enterobacterales	Enterobacteriaceae	Escherichia	Escherichia coli
3	Bacteria	Proteobacteria	Gammaproteobacteria	Enterobacterales	Enterobacteriaceae	Escherichia	Escherichia coli
3	Bacteria	Proteobacteria	Gammaproteobacteria	Enterobacterales	Enterobacteriaceae	Escherichia	Escherichia coli
3	Bacteria	Proteobacteria	Gammaproteobacteria	Enterobacterales	Enterobacteriaceae	Escherichia	Escherichia coli
3	Bacteria	Proteobacteria	Gammaproteobacteria	Enterobacterales	Enterobacteriaceae	Escherichia	Escherichia coli
3	Bacteria	Proteobacteria	Gammaproteobacteria	Enterobacterales	Enterobacteriaceae	Escherichia	Escherichia coli
3	Bacteria	Proteobacteria	Gammaproteobacteria	Enterobacterales	Enterobacteriaceae	Escherichia	Escherichia coli
3	Bacteria	Proteobacteria	Gammaproteobacteria	Enterobacterales	Enterobacteriaceae	Enterobacter	Enterobacter sp. Sa187
3	Bacteria	Proteobacteria	Gammaproteobacteria	Enterobacterales	Enterobacteriaceae	Enterobacter	Enterobacter sp. NFR05
3	Bacteria	Proteobacteria	Gammaproteobacteria	Enterobacterales	Enterobacteriaceae	Enterobacter	Enterobacter cloacae complex
3	Bacteria	Proteobacteria	Gammaproteobacteria	Enterobacterales	Enterobacteriaceae	Enterobacter	Enterobacter cloacae complex
3	Bacteria	Proteobacteria	Gammaproteobacteria	Enterobacterales	Enterobacteriaceae	Enterobacter	Enterobacter cloacae complex
3	Bacteria	Proteobacteria	Gammaproteobacteria	Enterobacterales	Enterobacteriaceae	Cronobacter	Cronobacter condimenti
3	Bacteria	Proteobacteria	Gammaproteobacteria	Enterobacterales	Enterobacteriaceae	Citrobacter	Citrobacter freundii complex
3	Bacteria	Proteobacteria	Gammaproteobacteria	Enterobacterales	Enterobacteriaceae	Buttiauxella	Buttiauxella gaviniae
3	Bacteria	Proteobacteria	Gammaproteobacteria	Enterobacterales	Budviciaceae	Leminorella	Leminorella grimontii
3	Bacteria	Proteobacteria	Gammaproteobacteria	Enterobacterales	Budviciaceae	Budvicia	Budvicia aquatica
3	Bacteria	Proteobacteria	Gammaproteobacteria	Chromatiales	Ectothiorhodospiraceae	Acidihalobacter	Acidihalobacter prosperus
3	Bacteria	Proteobacteria	Gammaproteobacteria	Chromatiales	Chromatiaceae	Nitrosococcus	Nitrosococcus halophilus
3	Bacteria	Proteobacteria	Gammaproteobacteria	Alteromonadales	Shewanellaceae	Shewanella	Shewanella sp. ZOR0012
3	Bacteria	Proteobacteria	Gammaproteobacteria	Alteromonadales	Shewanellaceae	Shewanella	Shewanella oneidensis
3	Bacteria	Proteobacteria	Gammaproteobacteria	Alteromonadales	Pseudoalteromonadaceae	Pseudoalteromonas	Pseudoalteromonas luteoviolacea
3	Bacteria	Proteobacteria	Gammaproteobacteria	Alteromonadales	Pseudoalteromonadaceae	Pseudoalteromonas	
3	Bacteria	Proteobacteria	Gammaproteobacteria	Alteromonadales	Colwelliaceae	Colwellia	Colwellia mytili
3	Bacteria	Proteobacteria	Gammaproteobacteria	Alteromonadales	Alteromonadaceae	Gayadomonas	Gayadomonas joobiniege
3	Bacteria	Proteobacteria	Gammaproteobacteria	Aeromonadales	Aeromonadaceae	Aeromonas	Aeromonas veronii
3	Bacteria	Proteobacteria	Gammaproteobacteria	Aeromonadales	Aeromonadaceae	Aeromonas	Aeromonas veronii
3	Bacteria	Proteobacteria	Gammaproteobacteria	Aeromonadales	Aeromonadaceae	Aeromonas	Aeromonas sp. ANP5
3	Bacteria	Proteobacteria	Candidatus Lambdaproteobacteria				
3	Bacteria	Proteobacteria	Betaproteobacteria	unclassified Betaproteobacteria	unclassified Betaproteobacteria (miscellaneous)	Betaproteobacteria bacterium RIFCSPLOWO2_12_FULL_64_23	
3	Bacteria	Proteobacteria	Betaproteobacteria	unclassified Betaproteobacteria	unclassified Betaproteobacteria (miscellaneous)	Betaproteobacteria bacterium RIFCSPLOWO2_02_FULL_67_19	
3	Bacteria	Proteobacteria	Betaproteobacteria	unclassified Betaproteobacteria	unclassified Betaproteobacteria (miscellaneous)	Betaproteobacteria bacterium RBG_16_66_20	
3	Bacteria	Proteobacteria	Betaproteobacteria	unclassified Betaproteobacteria	unclassified Betaproteobacteria (miscellaneous)	Betaproteobacteria bacterium MOLA814	
3	Bacteria	Proteobacteria	Betaproteobacteria	unclassified Betaproteobacteria	Candidatus Accumulibacter	Candidatus Accumulibacter sp. BA-91	
3	Bacteria	Proteobacteria	Betaproteobacteria	Rhodocyclales	unclassified Rhodocyclales	Rhodocyclales bacterium GWA2_65_20	
3	Bacteria	Proteobacteria	Betaproteobacteria	Rhodocyclales	Rhodocyclaceae	Zoogloea	Zoogloea sp. LCSB751
3	Bacteria	Proteobacteria	Betaproteobacteria	Rhodocyclales	Rhodocyclaceae	Propionivibrio	Propionivibrio dicarboxylicus
3	Bacteria	Proteobacteria	Betaproteobacteria	Rhodocyclales	Rhodocyclaceae	Propionivibrio	Candidatus Propionivibrio aalborgensis
3	Bacteria	Proteobacteria	Betaproteobacteria	Rhodocyclales	Rhodocyclaceae	Azoarcus	
3	Bacteria	Proteobacteria	Betaproteobacteria	Neisseriales	Chromobacteriaceae	Chromobacterium group	Chromobacterium
3	Bacteria	Proteobacteria	Betaproteobacteria	Neisseriales	Chromobacteriaceae	Chitinibacter	
3	Bacteria	Proteobacteria	Betaproteobacteria	Methylophilales	unclassified Methylophilales	Methylophilales bacterium RIFCSPHIGHO2_02_FULL_57_10	
3	Bacteria	Proteobacteria	Betaproteobacteria	Methylophilales	Methylophilaceae	Methylovorus	Methylovorus sp. MM1
3	Bacteria	Proteobacteria	Betaproteobacteria	Methylophilales	Methylophilaceae	Methylotenera	Methylotenera sp. G11
3	Bacteria	Proteobacteria	Betaproteobacteria	Burkholderiales	unclassified Burkholderiales	unclassified Burkholderiales (miscellaneous)	Burkholderiales bacterium RIFOXYC12_FULL_65_23
3	Bacteria	Proteobacteria	Betaproteobacteria	Burkholderiales	unclassified Burkholderiales	Burkholderiales Genera incertae sedis	Thiomonas
3	Bacteria	Proteobacteria	Betaproteobacteria	Burkholderiales	unclassified Burkholderiales	Burkholderiales Genera incertae sedis	Tepidimonas
3	Bacteria	Proteobacteria	Betaproteobacteria	Burkholderiales	unclassified Burkholderiales	Burkholderiales Genera incertae sedis	Rubrivivax
3	Bacteria	Proteobacteria	Betaproteobacteria	Burkholderiales	unclassified Burkholderiales	Burkholderiales Genera incertae sedis	Methylibium
3	Bacteria	Proteobacteria	Betaproteobacteria	Burkholderiales	unclassified Burkholderiales	Burkholderiales Genera incertae sedis	Ideonella
3	Bacteria	Proteobacteria	Betaproteobacteria	Burkholderiales	unclassified Burkholderiales	Burkholderiales Genera incertae sedis	Aquabacterium
3	Bacteria	Proteobacteria	Betaproteobacteria	Burkholderiales	Sutterellaceae	Sutterella	Sutterella wadsworthensis
3	Bacteria	Proteobacteria	Betaproteobacteria	Burkholderiales	Oxalobacteraceae	Noviherbaspirillum	Noviherbaspirillum sp. Root189
3	Bacteria	Proteobacteria	Betaproteobacteria	Burkholderiales	Oxalobacteraceae	Herbaspirillum	Herbaspirillum autotrophicum
3	Bacteria	Proteobacteria	Betaproteobacteria	Burkholderiales	Oxalobacteraceae	Collimonas	
3	Bacteria	Proteobacteria	Betaproteobacteria	Burkholderiales	Oxalobacteraceae		
3	Bacteria	Proteobacteria	Betaproteobacteria	Burkholderiales	Comamonadaceae	Rhodoferax	Rhodoferax saidenbachensis
3	Bacteria	Proteobacteria	Betaproteobacteria	Burkholderiales	Comamonadaceae	Polaromonas	Polaromonas sp. A23
3	Bacteria	Proteobacteria	Betaproteobacteria	Burkholderiales	Comamonadaceae	Polaromonas	Polaromonas glacialis
3	Bacteria	Proteobacteria	Betaproteobacteria	Burkholderiales	Comamonadaceae	Ottowia	Ottowia sp. oral taxon 894
3	Bacteria	Proteobacteria	Betaproteobacteria	Burkholderiales	Comamonadaceae	Limnohabitans	Limnohabitans sp. 103DPR2
3	Bacteria	Proteobacteria	Betaproteobacteria	Burkholderiales	Comamonadaceae	Hylemonella	Hylemonella gracilis
3	Bacteria	Proteobacteria	Betaproteobacteria	Burkholderiales	Comamonadaceae	Delftia	
3	Bacteria	Proteobacteria	Betaproteobacteria	Burkholderiales	Comamonadaceae	Curvibacter	Curvibacter delicatus
3	Bacteria	Proteobacteria	Betaproteobacteria	Burkholderiales	Comamonadaceae		
3	Bacteria	Proteobacteria	Betaproteobacteria	Burkholderiales	Burkholderiaceae	Ralstonia	Ralstonia solanacearum
3	Bacteria	Proteobacteria	Betaproteobacteria	Burkholderiales	Burkholderiaceae	Polynucleobacter	Polynucleobacter asymbioticus
3	Bacteria	Proteobacteria	Betaproteobacteria	Burkholderiales	Burkholderiaceae	Paraburkholderia	Paraburkholderia tropica
3	Bacteria	Proteobacteria	Betaproteobacteria	Burkholderiales	Burkholderiaceae	Cupriavidus	Cupriavidus necator
3	Bacteria	Proteobacteria	Betaproteobacteria	Burkholderiales	Burkholderiaceae	Cupriavidus	
3	Bacteria	Proteobacteria	Betaproteobacteria	Burkholderiales	Burkholderiaceae	Burkholderia	pseudomallei group
3	Bacteria	Proteobacteria	Betaproteobacteria	Burkholderiales	Burkholderiaceae	Burkholderia	Burkholderia cepacia complex
3	Bacteria	Proteobacteria	Betaproteobacteria	Burkholderiales	Burkholderiaceae	Burkholderia	Burkholderia cepacia complex
3	Bacteria	Proteobacteria	Betaproteobacteria	Burkholderiales	Alcaligenaceae		
3	Bacteria	Proteobacteria	Alphaproteobacteria	unclassified Alphaproteobacteria	unclassified Alphaproteobacteria (miscellaneous)	Alphaproteobacteria bacterium RIFCSPHIGHO2_12_FULL_63_12	
3	Bacteria	Proteobacteria	Alphaproteobacteria	unclassified Alphaproteobacteria	Thermopetrobacter	Thermopetrobacter sp. TC1	
3	Bacteria	Proteobacteria	Alphaproteobacteria	unclassified Alphaproteobacteria	Geminicoccus	Geminicoccus roseus	
3	Bacteria	Proteobacteria	Alphaproteobacteria	Sphingomonadales	unclassified Sphingomonadales	Sphingomonadales bacterium EhC05	
3	Bacteria	Proteobacteria	Alphaproteobacteria	Sphingomonadales	Sphingomonadaceae	Sphingopyxis	Sphingopyxis sp. H050
3	Bacteria	Proteobacteria	Alphaproteobacteria	Sphingomonadales	Sphingomonadaceae	Sphingomonas	Sphingomonas sp. MCT13
3	Bacteria	Proteobacteria	Alphaproteobacteria	Sphingomonadales	Sphingomonadaceae	Sphingomonas	Sphingomonas sp. Ag1
3	Bacteria	Proteobacteria	Alphaproteobacteria	Sphingomonadales	Sphingomonadaceae	Sphingomonas	Sphingomonas koreensis
3	Bacteria	Proteobacteria	Alphaproteobacteria	Sphingomonadales	Sphingomonadaceae	Pacificimonas	Pacificimonas flava
3	Bacteria	Proteobacteria	Alphaproteobacteria	Sphingomonadales	Sphingomonadaceae	Novosphingobium	Novosphingobium sp. SCN 66-18
3	Bacteria	Proteobacteria	Alphaproteobacteria	Sphingomonadales	Sphingomonadaceae	Novosphingobium	Novosphingobium sp. Fuku2-ISO-50
3	Bacteria	Proteobacteria	Alphaproteobacteria	Sphingomonadales	Sphingomonadaceae	Novosphingobium	Novosphingobium sp. FSW06-99
3	Bacteria	Proteobacteria	Alphaproteobacteria	Sphingomonadales	Sphingomonadaceae	Novosphingobium	Novosphingobium sp. AP12
3	Bacteria	Proteobacteria	Alphaproteobacteria	Sphingomonadales	Sphingomonadaceae	Novosphingobium	Novosphingobium acidiphilum
3	Bacteria	Proteobacteria	Alphaproteobacteria	Sphingomonadales	Sphingomonadaceae	Citromicrobium	
3	Bacteria	Proteobacteria	Alphaproteobacteria	Sphingomonadales	Sphingomonadaceae	Blastomonas	
3	Bacteria	Proteobacteria	Alphaproteobacteria	Sphingomonadales	Erythrobacteraceae	Erythrobacter	Erythrobacter marinus
3	Bacteria	Proteobacteria	Alphaproteobacteria	Sphingomonadales	Erythrobacteraceae		
3	Bacteria	Proteobacteria	Alphaproteobacteria	Rickettsiales	Anaplasmataceae	Wolbachieae	Wolbachia
3	Bacteria	Proteobacteria	Alphaproteobacteria	Rickettsiales	Anaplasmataceae	Ehrlichia	canis group
3	Bacteria	Proteobacteria	Alphaproteobacteria	Rhodospirillales	unclassified Rhodospirillales	unclassified Rhodospirillales (miscellaneous)	Rhodospirillales bacterium 70-18
3	Bacteria	Proteobacteria	Alphaproteobacteria	Rhodospirillales	unclassified Rhodospirillales	Elioraea	Elioraea tepidiphila
3	Bacteria	Proteobacteria	Alphaproteobacteria	Rhodospirillales	Rhodospirillaceae	Tistlia	Tistlia consotensis
3	Bacteria	Proteobacteria	Alphaproteobacteria	Rhodospirillales	Rhodospirillaceae	Magnetospirillum	Magnetospirillum sp. 64-120
3	Bacteria	Proteobacteria	Alphaproteobacteria	Rhodospirillales	Rhodospirillaceae	Fodinicurvata	Fodinicurvata fenggangensis
3	Bacteria	Proteobacteria	Alphaproteobacteria	Rhodobacterales	Rhodobacteraceae	unclassified Rhodobacteraceae	Rhodobacteraceae bacterium EhC02
3	Bacteria	Proteobacteria	Alphaproteobacteria	Rhodobacterales	Rhodobacteraceae	Roseovarius	Roseovarius albus
3	Bacteria	Proteobacteria	Alphaproteobacteria	Rhodobacterales	Rhodobacteraceae	Pseudooceanicola	Pseudooceanicola batsensis
3	Bacteria	Proteobacteria	Alphaproteobacteria	Rhodobacterales	Rhodobacteraceae	Ponticoccus	Ponticoccus sp. SJ5A-1
3	Bacteria	Proteobacteria	Alphaproteobacteria	Rhodobacterales	Rhodobacteraceae	Loktanella	Loktanella pyoseonensis
3	Bacteria	Proteobacteria	Alphaproteobacteria	Rhodobacterales	Rhodobacteraceae	Labrenzia	Labrenzia alba
3	Bacteria	Proteobacteria	Alphaproteobacteria	Rhodobacterales	Rhodobacteraceae	Labrenzia	
3	Bacteria	Proteobacteria	Alphaproteobacteria	Rhodobacterales	Rhodobacteraceae	Jannaschia	Jannaschia rubra
3	Bacteria	Proteobacteria	Alphaproteobacteria	Rhodobacterales	Rhodobacteraceae	Gemmobacter	Gemmobacter megaterium
3	Bacteria	Proteobacteria	Alphaproteobacteria	Rhodobacterales	Rhodobacteraceae	Defluviimonas	Defluviimonas alba
3	Bacteria	Proteobacteria	Alphaproteobacteria	Rhodobacterales	Hyphomonadaceae		
3	Bacteria	Proteobacteria	Alphaproteobacteria	Rhizobiales	unclassified Rhizobiales	unclassified Rhizobiales (miscellaneous)	
3	Bacteria	Proteobacteria	Alphaproteobacteria	Rhizobiales	Xanthobacteraceae	Labrys	Labrys sp. WJW
3	Bacteria	Proteobacteria	Alphaproteobacteria	Rhizobiales	Xanthobacteraceae	Ancylobacter	Ancylobacter sp. FA202
3	Bacteria	Proteobacteria	Alphaproteobacteria	Rhizobiales	Rhodobiaceae	Amorphus	Amorphus coralli
3	Bacteria	Proteobacteria	Alphaproteobacteria	Rhizobiales	Rhizobiaceae	Sinorhizobium/Ensifer group	
3	Bacteria	Proteobacteria	Alphaproteobacteria	Rhizobiales	Rhizobiaceae	Rhizobium/Agrobacterium group	Agrobacterium
3	Bacteria	Proteobacteria	Alphaproteobacteria	Rhizobiales	Phyllobacteriaceae	Nitratireductor	Nitratireductor basaltis
3	Bacteria	Proteobacteria	Alphaproteobacteria	Rhizobiales	Phyllobacteriaceae	Mesorhizobium	Mesorhizobium qingshengii
3	Bacteria	Proteobacteria	Alphaproteobacteria	Rhizobiales	Phyllobacteriaceae	Aliihoeflea	Aliihoeflea sp. 2WW
3	Bacteria	Proteobacteria	Alphaproteobacteria	Rhizobiales	Methylobacteriaceae	Microvirga	
3	Bacteria	Proteobacteria	Alphaproteobacteria	Rhizobiales	Hyphomicrobiaceae	Rhodomicrobium	
3	Bacteria	Proteobacteria	Alphaproteobacteria	Rhizobiales	Hyphomicrobiaceae	Prosthecomicrobium	Prosthecomicrobium hirschii
3	Bacteria	Proteobacteria	Alphaproteobacteria	Rhizobiales	Hyphomicrobiaceae	Hyphomicrobium	Hyphomicrobium zavarzinii
3	Bacteria	Proteobacteria	Alphaproteobacteria	Rhizobiales	Hyphomicrobiaceae	Hyphomicrobium	Hyphomicrobium sp. CS1BSMeth3
3	Bacteria	Proteobacteria	Alphaproteobacteria	Rhizobiales	Hyphomicrobiaceae	Devosia	Devosia geojensis
3	Bacteria	Proteobacteria	Alphaproteobacteria	Rhizobiales	Bradyrhizobiaceae	Bradyrhizobium	Bradyrhizobium sp. URHD0069
3	Bacteria	Proteobacteria	Alphaproteobacteria	Rhizobiales	Bradyrhizobiaceae	Bradyrhizobium	Bradyrhizobium pachyrhizi

3	Bacteria	Proteobacteria	Alphaproteobacteria	Rhizobiales	Beijerinckiaceae	Methylocapsa	Methylocapsa acidiphila
3	Bacteria	Proteobacteria	Alphaproteobacteria	Rhizobiales	Aurantimonadaceae	Aureimonas	Aureimonas sp. AU12
3	Bacteria	Proteobacteria	Alphaproteobacteria	Rhizobiales	Aurantimonadaceae	Aureimonas	Aureimonas altamirensis
3	Bacteria	Proteobacteria	Alphaproteobacteria	Caulobacterales	Caulobacteraceae	Caulobacter	
3	Bacteria	Proteobacteria	Alphaproteobacteria	Caulobacterales	Caulobacteraceae	Asticcacaulis	Asticcacaulis sp. AC460
3	Bacteria	Proteobacteria	Acidithiobacillia	Acidithiobacillales	unclassified Acidithiobacillales	Acidithiobacillales bacterium SM23_46	
3	Bacteria	Proteobacteria	Acidithiobacillia	Acidithiobacillales	Acidithiobacillaceae	Acidithiobacillus	Acidithiobacillus ferrivorans
3	Bacteria	Proteobacteria	Acidithiobacillia	Acidithiobacillales	Acidithiobacillaceae	Acidithiobacillus	Acidithiobacillus caldus
3	Bacteria	PVC group	Verrucomicrobia	unclassified Verrucomicrobia	unclassified Verrucomicrobia (miscellaneous)	Verrucomicrobia bacterium IMCC26134	
3	Bacteria	PVC group	Verrucomicrobia	Verrucomicrobiae	Verrucomicrobiales	Verrucomicrobiaceae	Verrucomicrobium
3	Bacteria	PVC group	Verrucomicrobia	Opitutae	Opitutales	Opitutaceae	Lacunisphaera
3	Bacteria	PVC group	Planctomycetes	unclassified Planctomycetes	Planctomycetes bacterium UTPLA1		
3	Bacteria	PVC group	Planctomycetes	unclassified Planctomycetes	Planctomycetes bacterium RBG_19FT_COMBO_48_8		
3	Bacteria	PVC group	Planctomycetes	unclassified Planctomycetes	Planctomycetes bacterium RBG_16_64_10		
3	Bacteria	PVC group	Planctomycetes	unclassified Planctomycetes	Planctomycetes bacterium GWF2_42_9		
3	Bacteria	PVC group	Planctomycetes	Planctomycetia	Planctomycetales	Planctomycetaceae	Rhodopirellula
3	Bacteria	PVC group	Planctomycetes	Planctomycetia	Planctomycetales	Isosphaeraceae	Singulisphaera
3	Bacteria	PVC group	Lentisphaerae	unclassified Lentisphaerae	unclassified Lentisphaerae (miscellaneous)	Lentisphaerae bacterium GWF2_57_35	
3	Bacteria	PVC group	Chlamydiae	Chlamydiia	Chlamydiales	Chlamydiaceae	Chlamydia/Chlamydophila group
3	Bacteria	PVC group					
3	Bacteria	Nitrospirae	Nitrospira	Nitrospirales	Nitrospiraceae	Nitrospira	Nitrospira sp. ST-bin4
3	Bacteria	Nitrospirae	Nitrospira	Nitrospirales	Nitrospiraceae	Nitrospira	Nitrospira moscoviensis
3	Bacteria	Nitrospirae	Nitrospira	Nitrospirales	Nitrospiraceae	Nitrospira	Nitrospira japonica
3	Bacteria	FCB group	Gemmatimonadetes	Gemmatimonadetes	Gemmatimonadales	Gemmatimonadaceae	Gemmatimonas
3	Bacteria	FCB group	Gemmatimonadetes				
3	Bacteria	FCB group	Candidatus Kryptonia	Candidatus Kryptonium	Candidatus Kryptonium thompsoni		
3	Bacteria	FCB group	Candidatus Kryptonia	Candidatus Kryptobacter	Candidatus Kryptobacter tengchongensis		
3	Bacteria	FCB group	Bacteroidetes/Chlorobi group	Bacteroidetes	unclassified Bacteroidetes	unclassified Bacteroidetes (miscellaneous)	Bacteroidetes bacterium 4572_117
3	Bacteria	FCB group	Bacteroidetes/Chlorobi group	Bacteroidetes	Sphingobacteriia	Sphingobacteriales	unclassified Sphingobacteriales
3	Bacteria	FCB group	Bacteroidetes/Chlorobi group	Bacteroidetes	Sphingobacteriia	Sphingobacteriales	Sphingobacteriaceae
3	Bacteria	FCB group	Bacteroidetes/Chlorobi group	Bacteroidetes	Sphingobacteriia	Sphingobacteriales	Sphingobacteriaceae
3	Bacteria	FCB group	Bacteroidetes/Chlorobi group	Bacteroidetes	Sphingobacteriia	Sphingobacteriales	Sphingobacteriaceae
3	Bacteria	FCB group	Bacteroidetes/Chlorobi group	Bacteroidetes	Sphingobacteriia	Sphingobacteriales	Sphingobacteriaceae
3	Bacteria	FCB group	Bacteroidetes/Chlorobi group	Bacteroidetes	Flavobacteriia	Flavobacteriales	Flavobacteriaceae
3	Bacteria	FCB group	Bacteroidetes/Chlorobi group	Bacteroidetes	Flavobacteriia	Flavobacteriales	Flavobacteriaceae
3	Bacteria	FCB group	Bacteroidetes/Chlorobi group	Bacteroidetes	Flavobacteriia	Flavobacteriales	Flavobacteriaceae
3	Bacteria	FCB group	Bacteroidetes/Chlorobi group	Bacteroidetes	Flavobacteriia	Flavobacteriales	Flavobacteriaceae
3	Bacteria	FCB group	Bacteroidetes/Chlorobi group	Bacteroidetes	Flavobacteriia	Flavobacteriales	Flavobacteriaceae
3	Bacteria	FCB group	Bacteroidetes/Chlorobi group	Bacteroidetes	Flavobacteriia	Flavobacteriales	Cryomorphaceae
3	Bacteria	FCB group	Bacteroidetes/Chlorobi group	Bacteroidetes	Cytophagia	Cytophagales	unclassified Cytophagales
3	Bacteria	FCB group	Bacteroidetes/Chlorobi group	Bacteroidetes	Cytophagia	Cytophagales	Hymenobacteraceae
3	Bacteria	FCB group	Bacteroidetes/Chlorobi group	Bacteroidetes	Cytophagia	Cytophagales	Cytophagaceae
3	Bacteria	FCB group	Bacteroidetes/Chlorobi group	Bacteroidetes	Cytophagia	Cytophagales	Cytophagaceae
3	Bacteria	FCB group	Bacteroidetes/Chlorobi group	Bacteroidetes	Cytophagia	Cytophagales	Cytophagaceae
3	Bacteria	FCB group	Bacteroidetes/Chlorobi group	Bacteroidetes	Cytophagia	Cytophagales	Cytophagaceae
3	Bacteria	FCB group	Bacteroidetes/Chlorobi group	Bacteroidetes	Chitinophagia	Chitinophagales	Chitinophagaceae
3	Bacteria	FCB group	Bacteroidetes/Chlorobi group	Bacteroidetes	Bacteroidia	Marinilabiliales	Prolixibacteraceae
3	Bacteria	FCB group	Bacteroidetes/Chlorobi group	Bacteroidetes	Bacteroidia	Marinilabiliales	Marinilabiliaceae
3	Bacteria	FCB group	Bacteroidetes/Chlorobi group	Bacteroidetes	Bacteroidia	Marinilabiliales	Marinilabiliaceae
3	Bacteria	FCB group	Bacteroidetes/Chlorobi group	Bacteroidetes	Bacteroidia	Marinilabiliales	Marinilabiliaceae
3	Bacteria	FCB group	Bacteroidetes/Chlorobi group	Bacteroidetes	Bacteroidia	Bacteroidales	unclassified Bacteroidales
3	Bacteria	FCB group	Bacteroidetes/Chlorobi group	Bacteroidetes	Bacteroidia	Bacteroidales	unclassified Bacteroidales
3	Bacteria	FCB group	Bacteroidetes/Chlorobi group	Bacteroidetes	Bacteroidia	Bacteroidales	unclassified Bacteroidales
3	Bacteria	FCB group	Bacteroidetes/Chlorobi group	Bacteroidetes	Bacteroidia	Bacteroidales	Prevotellaceae
3	Bacteria	FCB group	Bacteroidetes/Chlorobi group	Bacteroidetes	Bacteroidia	Bacteroidales	Porphyromonadaceae
[illegible]	[illegible]	[illegible]	[illegible]	[illegible]	[illegible]	[illegible]	[illegible]

2	Bacteria	Proteobacteria	Gammaproteobacteria	Enterobacterales	Enterobacteriaceae	Enterobacter	Enterobacter cloacae complex
2	Bacteria	Proteobacteria	Gammaproteobacteria	Enterobacterales	Enterobacteriaceae	Enterobacter	Enterobacter cloacae complex
2	Bacteria	Proteobacteria	Gammaproteobacteria	Enterobacterales	Enterobacteriaceae	Enterobacter	Enterobacter cloacae complex
2	Bacteria	Proteobacteria	Gammaproteobacteria	Enterobacterales	Enterobacteriaceae	Cronobacter	Cronobacter sakazakii
2	Bacteria	Proteobacteria	Gammaproteobacteria	Enterobacterales	Enterobacteriaceae	Citrobacter	Citrobacter sp. KTE30
2	Bacteria	Proteobacteria	Gammaproteobacteria	Enterobacterales	Enterobacteriaceae	Citrobacter	Citrobacter sp. A1
2	Bacteria	Proteobacteria	Gammaproteobacteria	Enterobacterales	Enterobacteriaceae	Citrobacter	Citrobacter freundii complex
2	Bacteria	Proteobacteria	Gammaproteobacteria	Enterobacterales	Enterobacteriaceae	Citrobacter	Citrobacter freundii complex
2	Bacteria	Proteobacteria	Gammaproteobacteria	Enterobacterales	Enterobacteriaceae	Citrobacter	Citrobacter freundii complex
2	Bacteria	Proteobacteria	Gammaproteobacteria	Enterobacterales	Enterobacteriaceae	Citrobacter	Citrobacter freundii complex
2	Bacteria	Proteobacteria	Gammaproteobacteria	Enterobacterales	Enterobacteriaceae	Citrobacter	Citrobacter freundii complex
2	Bacteria	Proteobacteria	Gammaproteobacteria	Enterobacterales	Enterobacteriaceae	Cedecea	Cedecea neteri
2	Bacteria	Proteobacteria	Gammaproteobacteria	Enterobacterales	Enterobacteriaceae	Buttiauxella	Buttiauxella brennerae
2	Bacteria	Proteobacteria	Gammaproteobacteria	Enterobacterales	Enterobacteriaceae	Buttiauxella	Buttiauxella agrestis
2	Bacteria	Proteobacteria	Gammaproteobacteria	Chromatiales	environmental samples	uncultured Chromatiales bacterium HF0200_41F04	
2	Bacteria	Proteobacteria	Gammaproteobacteria	Chromatiales	Woeseiaceae	Woeseia	Woeseia oceani
2	Bacteria	Proteobacteria	Gammaproteobacteria	Chromatiales	Ectothiorhodospiraceae	Thioalkalivibrio	
2	Bacteria	Proteobacteria	Gammaproteobacteria	Chromatiales	Chromatiaceae	Rheinheimera	Rheinheimera sp. A13L
2	Bacteria	Proteobacteria	Gammaproteobacteria	Chromatiales	Chromatiaceae	Rheinheimera	Rheinheimera nanhaiensis
2	Bacteria	Proteobacteria	Gammaproteobacteria	Chromatiales	Chromatiaceae	Candidatus Tenderia	Candidatus Tenderia electrophaga
2	Bacteria	Proteobacteria	Gammaproteobacteria	Cellvibrionales	Microbulbiferaceae	Microbulbifer	Microbulbifer donghaiensis
2	Bacteria	Proteobacteria	Gammaproteobacteria	Alteromonadales	Shewanellaceae	Shewanella	Shewanella sp. POL2
2	Bacteria	Proteobacteria	Gammaproteobacteria	Alteromonadales	Shewanellaceae	Shewanella	Shewanella sp. MR-7
2	Bacteria	Proteobacteria	Gammaproteobacteria	Alteromonadales	Shewanellaceae	Shewanella	Shewanella sp. MR-4
2	Bacteria	Proteobacteria	Gammaproteobacteria	Alteromonadales	Shewanellaceae	Shewanella	Shewanella sp. ECSMB14102
2	Bacteria	Proteobacteria	Gammaproteobacteria	Alteromonadales	Shewanellaceae	Shewanella	Shewanella piezotolerans
2	Bacteria	Proteobacteria	Gammaproteobacteria	Alteromonadales	Shewanellaceae	Shewanella	Shewanella mangrovi
2	Bacteria	Proteobacteria	Gammaproteobacteria	Alteromonadales	Shewanellaceae	Shewanella	Shewanella baltica
2	Bacteria	Proteobacteria	Gammaproteobacteria	Alteromonadales	Pseudoalteromonadaceae	Pseudoalteromonas	Pseudoalteromonas luteoviolacea
2	Bacteria	Proteobacteria	Gammaproteobacteria	Alteromonadales	Alteromonadaceae	Marinobacter	
2	Bacteria	Proteobacteria	Gammaproteobacteria	Alteromonadales	Alteromonadaceae	Alteromonas	Alteromonas lipolytica
2	Bacteria	Proteobacteria	Gammaproteobacteria	Alteromonadales	Alteromonadaceae	Alishewanella	
2	Bacteria	Proteobacteria	Gammaproteobacteria	Aeromonadales	Succinivibrionaceae	Succinatimonas	Succinatimonas hippei
2	Bacteria	Proteobacteria	Gammaproteobacteria	Aeromonadales	Aeromonadaceae	Aeromonas	Aeromonas salmonicida
2	Bacteria	Proteobacteria	Gammaproteobacteria	Aeromonadales	Aeromonadaceae	Aeromonas	Aeromonas hydrophila
2	Bacteria	Proteobacteria	Betaproteobacteria	unclassified Betaproteobacteria	unclassified Betaproteobacteria (miscellaneous)	beta proteobacterium AAP99	
2	Bacteria	Proteobacteria	Betaproteobacteria	unclassified Betaproteobacteria	unclassified Betaproteobacteria (miscellaneous)	Betaproteobacteria bacterium RIFCSPLOWO2_02_64_14	
2	Bacteria	Proteobacteria	Betaproteobacteria	unclassified Betaproteobacteria	unclassified Betaproteobacteria (miscellaneous)	Betaproteobacteria bacterium RBG_16_56_24	
2	Bacteria	Proteobacteria	Betaproteobacteria	unclassified Betaproteobacteria	unclassified Betaproteobacteria (miscellaneous)	Betaproteobacteria bacterium 13_1_40CM_4_64_4	
2	Bacteria	Proteobacteria	Betaproteobacteria	unclassified Betaproteobacteria	Candidatus Accumulibacter	Candidatus Accumulibacter sp. BA-93	
2	Bacteria	Proteobacteria	Betaproteobacteria	unclassified Betaproteobacteria	Candidatus Accumulibacter	Candidatus Accumulibacter sp. 66-26	
2	Bacteria	Proteobacteria	Betaproteobacteria	Rhodocyclales	Rhodocyclaceae	Uliginosibacterium	Uliginosibacterium gangwonense
2	Bacteria	Proteobacteria	Betaproteobacteria	Rhodocyclales	Rhodocyclaceae	Thauera	Thauera terpenica
2	Bacteria	Proteobacteria	Betaproteobacteria	Rhodocyclales	Rhodocyclaceae	Thauera	Thauera phenylacetica
2	Bacteria	Proteobacteria	Betaproteobacteria	Rhodocyclales	Rhodocyclaceae	Thauera	
2	Bacteria	Proteobacteria	Betaproteobacteria	Rhodocyclales	Rhodocyclaceae	Sulfuritalea	Sulfuritalea hydrogenivorans
2	Bacteria	Proteobacteria	Betaproteobacteria	Rhodocyclales	Rhodocyclaceae	Rugosibacter	Rugosibacter aromaticivorans
2	Bacteria	Proteobacteria	Betaproteobacteria	Rhodocyclales	Rhodocyclaceae		
2	Bacteria	Proteobacteria	Betaproteobacteria	Rhodocyclales			
2	Bacteria	Proteobacteria	Betaproteobacteria	Nitrosomonadales	Nitrosomonadaceae	Nitrosomonas	Nitrosomonas marina
2	Bacteria	Proteobacteria	Betaproteobacteria	Nitrosomonadales	Nitrosomonadaceae	Nitrosomonas	Nitrosomonas cryotolerans
2	Bacteria	Proteobacteria	Betaproteobacteria	Nitrosomonadales	Nitrosomonadaceae	Nitrosomonas	
2	Bacteria	Proteobacteria	Betaproteobacteria	Neisseriales	Neisseriaceae	Stenoxybacter	Stenoxybacter acetivorans
2	Bacteria	Proteobacteria	Betaproteobacteria	Neisseriales	Neisseriaceae	Neisseria	Neisseria meningitidis
2	Bacteria	Proteobacteria	Betaproteobacteria	Neisseriales	Neisseriaceae	Kingella	Kingella sp. Sch538
2	Bacteria	Proteobacteria	Betaproteobacteria	Neisseriales	Neisseriaceae	Crenobacter	Crenobacter luteus
2	Bacteria	Proteobacteria	Betaproteobacteria	Neisseriales	Chromobacteriaceae	Vogesella	Vogesella sp. EB
2	Bacteria	Proteobacteria	Betaproteobacteria	Neisseriales	Chromobacteriaceae	Pseudogulbenkiania	Pseudogulbenkiania sp. MAI-1
2	Bacteria	Proteobacteria	Betaproteobacteria	Neisseriales	Chromobacteriaceae	Jeongeupia	Jeongeupia sp. USM3
2	Bacteria	Proteobacteria	Betaproteobacteria	Neisseriales	Chromobacteriaceae	Gulbenkiania	
2	Bacteria	Proteobacteria	Betaproteobacteria	Neisseriales	Chromobacteriaceae	Formivibrio	Formivibrio citricus
2	Bacteria	Proteobacteria	Betaproteobacteria	Neisseriales	Chromobacteriaceae	Chitiniphilus	Chitiniphilus shinanonensis
2	Bacteria	Proteobacteria	Betaproteobacteria	Neisseriales	Chromobacteriaceae	Chitinibacter	Chitinibacter tainanensis
2	Bacteria	Proteobacteria	Betaproteobacteria	Neisseriales	Chromobacteriaceae	Aquitalea	
2	Bacteria	Proteobacteria	Betaproteobacteria	Neisseriales	Chromobacteriaceae	Andreprevotia	Andreprevotia chitinilytica
2	Bacteria	Proteobacteria	Betaproteobacteria	Methylophilales	Methylophilaceae	Methylotenera	Methylotenera sp. RIFCSPLOWO2_02_FULL_45_14
2	Bacteria	Proteobacteria	Betaproteobacteria	Methylophilales	Methylophilaceae	Methylotenera	Methylotenera sp. N17
2	Bacteria	Proteobacteria	Betaproteobacteria	Methylophilales	Methylophilaceae	Methylophilus	
2	Bacteria	Proteobacteria	Betaproteobacteria	Hydrogenophilales	Hydrogenophilaceae	Thiobacillus	Thiobacillus sp. 65-29
2	Bacteria	Proteobacteria	Betaproteobacteria	Hydrogenophilales			
2	Bacteria	Proteobacteria	Betaproteobacteria	Gallionellales	unclassified Gallionellales	Gallionellales bacterium GWA2_59_43	
2	Bacteria	Proteobacteria	Betaproteobacteria	Ferrovales	Ferrovaceae	Ferrovum	Ferrovum sp. JA12
2	Bacteria	Proteobacteria	Betaproteobacteria	Ferrovales	Ferrovaceae	Ferrovum	
2	Bacteria	Proteobacteria	Betaproteobacteria	Burkholderiales	unclassified Burkholderiales	unclassified Burkholderiales (miscellaneous)	Burkholderiales bacterium RIFCSPLOWO2_12_FULL_61_40
2	Bacteria	Proteobacteria	Betaproteobacteria	Burkholderiales	unclassified Burkholderiales	unclassified Burkholderiales (miscellaneous)	Burkholderiales bacterium 68-10
2	Bacteria	Proteobacteria	Betaproteobacteria	Burkholderiales	unclassified Burkholderiales	unclassified Burkholderiales (miscellaneous)	
2	Bacteria	Proteobacteria	Betaproteobacteria	Burkholderiales	unclassified Burkholderiales	[Polyangium] brachysporum	
2	Bacteria	Proteobacteria	Betaproteobacteria	Burkholderiales	unclassified Burkholderiales	Burkholderiales Genera incertae sedis	Tepidimonas
2	Bacteria	Proteobacteria	Betaproteobacteria	Burkholderiales	unclassified Burkholderiales	Burkholderiales Genera incertae sedis	Rubrivivax
2	Bacteria	Proteobacteria	Betaproteobacteria	Burkholderiales	unclassified Burkholderiales	Burkholderiales Genera incertae sedis	Mitsuaria
2	Bacteria	Proteobacteria	Betaproteobacteria	Burkholderiales	unclassified Burkholderiales	Burkholderiales Genera incertae sedis	Ideonella
2	Bacteria	Proteobacteria	Betaproteobacteria	Burkholderiales	unclassified Burkholderiales	Burkholderiales Genera incertae sedis	Aquincola
2	Bacteria	Proteobacteria	Betaproteobacteria	Burkholderiales	unclassified Burkholderiales	Burkholderiales Genera incertae sedis	Aquabacterium
2	Bacteria	Proteobacteria	Betaproteobacteria	Burkholderiales	unclassified Burkholderiales		
2	Bacteria	Proteobacteria	Betaproteobacteria	Burkholderiales	Sutterellaceae	Sutterella	environmental samples
2	Bacteria	Proteobacteria	Betaproteobacteria	Burkholderiales	Oxalobacteraceae	Massilia	Massilia sp. KIM
2	Bacteria	Proteobacteria	Betaproteobacteria	Burkholderiales	Oxalobacteraceae	Janthinobacterium	Janthinobacterium sp. Marseille
2	Bacteria	Proteobacteria	Betaproteobacteria	Burkholderiales	Oxalobacteraceae	Janthinobacterium	Janthinobacterium sp. B9-8
2	Bacteria	Proteobacteria	Betaproteobacteria	Burkholderiales	Oxalobacteraceae	Janthinobacterium	Janthinobacterium lividum
2	Bacteria	Proteobacteria	Betaproteobacteria	Burkholderiales	Oxalobacteraceae	Janthinobacterium	Janthinobacterium agaricidamnosum
2	Bacteria	Proteobacteria	Betaproteobacteria	Burkholderiales	Oxalobacteraceae	Herbaspirillum	Herbaspirillum sp. TSA66
2	Bacteria	Proteobacteria	Betaproteobacteria	Burkholderiales	Oxalobacteraceae	Duganella	
2	Bacteria	Proteobacteria	Betaproteobacteria	Burkholderiales	Comamonadaceae	unclassified Comamonadaceae	
2	Bacteria	Proteobacteria	Betaproteobacteria	Burkholderiales	Comamonadaceae	Verminephrobacter	Verminephrobacter eiseniae
2	Bacteria	Proteobacteria	Betaproteobacteria	Burkholderiales	Comamonadaceae	Rhodoferax	Albidiferax sp. OV413
2	Bacteria	Proteobacteria	Betaproteobacteria	Burkholderiales	Comamonadaceae	Rhodoferax	
2	Bacteria	Proteobacteria	Betaproteobacteria	Burkholderiales	Comamonadaceae	Pseudorhodoferax	Pseudorhodoferax sp. Leaf267
2	Bacteria	Proteobacteria	Betaproteobacteria	Burkholderiales	Comamonadaceae	Polaromonas	Polaromonas sp. CG9_12
2	Bacteria	Proteobacteria	Betaproteobacteria	Burkholderiales	Comamonadaceae	Pelomonas	Pelomonas sp. Root1444
2	Bacteria	Proteobacteria	Betaproteobacteria	Burkholderiales	Comamonadaceae	Lampropedia	Lampropedia cohaerens
2	Bacteria	Proteobacteria	Betaproteobacteria	Burkholderiales	Comamonadaceae	Hydrogenophaga	Hydrogenophaga sp. Root209
2	Bacteria	Proteobacteria	Betaproteobacteria	Burkholderiales	Comamonadaceae	Hydrogenophaga	Hydrogenophaga sp. PBC
2	Bacteria	Proteobacteria	Betaproteobacteria	Burkholderiales	Comamonadaceae	Hydrogenophaga	Hydrogenophaga sp. H7
2	Bacteria	Proteobacteria	Betaproteobacteria	Burkholderiales	Comamonadaceae	Hydrogenophaga	Hydrogenophaga palleronii
2	Bacteria	Proteobacteria	Betaproteobacteria	Burkholderiales	Comamonadaceae	Hydrogenophaga	Hydrogenophaga flava
2	Bacteria	Proteobacteria	Betaproteobacteria	Burkholderiales	Comamonadaceae	Delftia	Delftia acidovorans
2	Bacteria	Proteobacteria	Betaproteobacteria	Burkholderiales	Comamonadaceae	Comamonas	Comamonas sp. SCN 65-56
2	Bacteria	Proteobacteria	Betaproteobacteria	Burkholderiales	Comamonadaceae	Caldimonas	Caldimonas taiwanensis
2	Bacteria	Proteobacteria	Betaproteobacteria	Burkholderiales	Comamonadaceae	Acidovorax	Acidovorax delafieldii
2	Bacteria	Proteobacteria	Betaproteobacteria	Burkholderiales	Burkholderiaceae	Ralstonia	Ralstonia insidiosa
2	Bacteria	Proteobacteria	Betaproteobacteria	Burkholderiales	Burkholderiaceae	Polynucleobacter	
2	Bacteria	Proteobacteria	Betaproteobacteria	Burkholderiales	Burkholderiaceae	Paraburkholderia	Paraburkholderia kururiensis
2	Bacteria	Proteobacteria	Betaproteobacteria	Burkholderiales	Burkholderiaceae	Paraburkholderia	Paraburkholderia diazotrophica
2	Bacteria	Proteobacteria	Betaproteobacteria	Burkholderiales	Burkholderiaceae	Paraburkholderia	Paraburkholderia caribensis
2	Bacteria	Proteobacteria	Betaproteobacteria	Burkholderiales	Burkholderiaceae	Pandoraea	
2	Bacteria	Proteobacteria	Betaproteobacteria	Burkholderiales	Burkholderiaceae	Lautropia	Lautropia mirabilis
2	Bacteria	Proteobacteria	Betaproteobacteria	Burkholderiales	Burkholderiaceae	Cupriavidus	Cupriavidus sp. WS
2	Bacteria	Proteobacteria	Betaproteobacteria	Burkholderiales	Burkholderiaceae	Cupriavidus	Cupriavidus sp. HPC(L)
2	Bacteria	Proteobacteria	Betaproteobacteria	Burkholderiales	Burkholderiaceae	Cupriavidus	Cupriavidus sp. HMR-1
2	Bacteria	Proteobacteria	Betaproteobacteria	Burkholderiales	Burkholderiaceae	Cupriavidus	Cupriavidus basilensis
2	Bacteria	Proteobacteria	Betaproteobacteria	Burkholderiales	Burkholderiaceae	Caballeronia	Caballeronia sordidicola
2	Bacteria	Proteobacteria	Betaproteobacteria	Burkholderiales	Burkholderiaceae	Burkholderia	Burkholderia cepacia complex
2	Bacteria	Proteobacteria	Betaproteobacteria	Burkholderiales	Burkholderiaceae	Burkholderia	Burkholderia cepacia complex
2	Bacteria	Proteobacteria	Betaproteobacteria	Burkholderiales	Alcaligenaceae	Orrella	Orrella dioscoreae
2	Bacteria	Proteobacteria	Betaproteobacteria	Burkholderiales	Alcaligenaceae	Candidimonas	Candidimonas bauzanensis
2	Bacteria	Proteobacteria	Betaproteobacteria	Burkholderiales	Alcaligenaceae	Bordetella	Bordetella sp. FB-8
2	Bacteria	Proteobacteria	Betaproteobacteria	Burkholderiales	Alcaligenaceae	Bordetella	Bordetella petrii
2	Bacteria	Proteobacteria	Betaproteobacteria	Burkholderiales	Alcaligenaceae	Bordetella	Bordetella ansorpii
2	Bacteria	Proteobacteria	Betaproteobacteria	Burkholderiales	Alcaligenaceae	Bordetella	
2	Bacteria	Proteobacteria	Betaproteobacteria	Burkholderiales	Alcaligenaceae	Azohydromonas	
2	Bacteria	Proteobacteria	Alphaproteobacteria	unclassified Alphaproteobacteria	unclassified Alphaproteobacteria (miscellaneous)	alpha proteobacterium BAL199	
2	Bacteria	Proteobacteria	Alphaproteobacteria	unclassified Alphaproteobacteria	unclassified Alphaproteobacteria (miscellaneous)	alpha proteobacterium AAP81b	
2	Bacteria	Proteobacteria	Alphaproteobacteria	unclassified Alphaproteobacteria	unclassified Alphaproteobacteria (miscellaneous)	Alphaproteobacteria bacterium RIFCSPHIGHO2_12_FULL_66_14	
2	Bacteria	Proteobacteria	Alphaproteobacteria	unclassified Alphaproteobacteria	unclassified Alphaproteobacteria (miscellaneous)	Alphaproteobacteria bacterium CG1_02_46_17	
2	Bacteria	Proteobacteria	Alphaproteobacteria	unclassified Alphaproteobacteria	unclassified Alphaproteobacteria (miscellaneous)	Alphaproteobacteria bacterium BRH_c36	
2	Bacteria	Proteobacteria	Alphaproteobacteria	unclassified Alphaproteobacteria	unclassified Alphaproteobacteria (miscellaneous)	Alphaproteobacteria bacterium AO1-B	
2	Bacteria	Proteobacteria	Alphaproteobacteria	unclassified Alphaproteobacteria	unclassified Alphaproteobacteria (miscellaneous)	Alphaproteobacteria bacterium 41-28	
2	Bacteria	Proteobacteria	Alphaproteobacteria	unclassified Alphaproteobacteria	unclassified Alphaproteobacteria (miscellaneous)		
2	Bacteria	Proteobacteria	Alphaproteobacteria	unclassified Alphaproteobacteria	SAR116 cluster	Candidatus Puniceispirillum	Candidatus Puniceispirillum marinum
2	Bacteria	Proteobacteria	Alphaproteobacteria	unclassified Alphaproteobacteria	Polymorphum	Polymorphum gilvum	
2	Bacteria	Proteobacteria	Alphaproteobacteria	Sphingomonadales	unclassified Sphingomonadales	Sphingomonadales bacterium BRH_c42	
2	Bacteria	Proteobacteria	Alphaproteobacteria	Sphingomonadales	unclassified Sphingomonadales	Sphingomonadales bacterium BRH_c3	
2	Bacteria	Proteobacteria	Alphaproteobacteria	Sphingomonadales	Sphingomonadaceae	Sphingopyxis	Sphingopyxis sp. YR583
2	Bacteria	Proteobacteria	Alphaproteobacteria	Sphingomonadales	Sphingomonadaceae	Sphingopyxis	Sphingopyxis sp. Root1497
2	Bacteria	Proteobacteria	Alphaproteobacteria	Sphingomonadales	Sphingomonadaceae	Sphingopyxis	Sphingopyxis alaskensis
2	Bacteria	Proteobacteria	Alphaproteobacteria	Sphingomonadales	Sphingomonadaceae	Sphingomonas	Sphingomonas sp. Y57
2	Bacteria	Proteobacteria	Alphaproteobacteria	Sphingomonadales	Sphingomonadaceae	Sphingomonas	Sphingomonas sp. URHD0057
2	Bacteria	Proteobacteria	Alphaproteobacteria	Sphingomonadales	Sphingomonadaceae	Sphingomonas	Sphingomonas sp. TZW2008
2	Bacteria	Proteobacteria	Alphaproteobacteria	Sphingomonadales	Sphingomonadaceae	Sphingomonas	Sphingomonas sp. NFR15
2	Bacteria	Proteobacteria	Alphaproteobacteria	Sphingomonadales	Sphingomonadaceae	Sphingomonas	Sphingomonas sp. Leaf17
2	Bacteria	Proteobacteria	Alphaproteobacteria	Sphingomonadales	Sphingomonadaceae	Sphingomonas	Sphingomonas sp. LM7
2	Bacteria	Proteobacteria	Alphaproteobacteria	Sphingomonadales	Sphingomonadaceae	Sphingomonas	Sphingomonas sp. LH128
2	Bacteria	Proteobacteria	Alphaproteobacteria	Sphingomonadales	Sphingomonadaceae	Sphingomonas	Sphingomonas sp. Ant20
2	Bacteria	Proteobacteria	Alphaproteobacteria	Sphingomonadales	Sphingomonadaceae	Sphingomonas	Sphingomonas sp. 66-10
2	Bacteria	Proteobacteria	Alphaproteobacteria	Sphingomonadales	Sphingomonadaceae	Sphingomonas	Sphingomonas sp. 35-24ZXX
2	Bacteria	Proteobacteria	Alphaproteobacteria	Sphingomonadales	Sphingomonadaceae	Sphingomonas	Sphingomonas rubra
2	Bacteria	Proteobacteria	Alphaproteobacteria	Sphingomonadales	Sphingomonadaceae	Sphingobium	Sphingobium xenophagum
2	Bacteria	Proteobacteria	Alphaproteobacteria	Sphingomonadales	Sphingomonadaceae	Sphingobium	Sphingobium sp. MI1205
2	Bacteria	Proteobacteria	Alphaproteobacteria	Sphingomonadales	Sphingomonadaceae	Sphingobium	Sphingobium phenoxybenzoativorans
2	Bacteria	Proteobacteria	Alphaproteobacteria	Sphingomonadales	Sphingomonadaceae	Novosphingobium	Novosphingobium sp. ST904
2	Bacteria	Proteobacteria	Alphaproteobacteria	Sphingomonadales	Sphingomonadaceae	Novosphingobium	Novosphingobium sp. MD-1
2	Bacteria	Proteobacteria	Alphaproteobacteria	Sphingomonadales	Sphingomonadaceae	Novosphingobium	Novosphingobium sp. B1
2	Bacteria	Proteobacteria	Alphaproteobacteria	Sphingomonadales	Sphingomonadaceae	Novosphingobium	Novosphingobium sp. AAP83
2	Bacteria	Proteobacteria	Alphaproteobacteria	Sphingomonadales	Sphingomonadaceae	Novosphingobium	Novosphingobium fuchskuhlense
2	Bacteria	Proteobacteria	Alphaproteobacteria	Sphingomonadales	Sphingomonadaceae	Blastomonas	Blastomonas sp. RAC04
2	Bacteria	Proteobacteria	Alphaproteobacteria	Sphingomonadales	Sphingomonadaceae	Blastomonas	Blastomonas sp. AAP25
2	Bacteria	Proteobacteria	Alphaproteobacteria	Sphingomonadales	Erythrobacteraceae	Porphyrobacter	Porphyrobacter sp. LM 6
2	Bacteria	Proteobacteria	Alphaproteobacteria	Sphingomonadales	Erythrobacteraceae	Porphyrobacter	Porphyrobacter dokdonensis
2	Bacteria	Proteobacteria	Alphaproteobacteria	Sphingomonadales	Erythrobacteraceae	Porphyrobacter	Porphyrobacter cryptus
2	Bacteria	Proteobacteria	Alphaproteobacteria	Sphingomonadales	Erythrobacteraceae	Erythrobacter	Erythrobacter sp. YT30
2	Bacteria	Proteobacteria	Alphaproteobacteria	Sphingomonadales	Erythrobacteraceae	Erythrobacter	Erythrobacter atlanticus
2	Bacteria	Proteobacteria	Alphaproteobacteria	Sphingomonadales	Erythrobacteraceae	Erythrobacter	
2	Bacteria	Proteobacteria	Alphaproteobacteria	Sphingomonadales	Erythrobacteraceae	Croceicoccus	Croceicoccus naphthovorans
2	Bacteria	Proteobacteria	Alphaproteobacteria	Sphingomonadales	Erythrobacteraceae	Croceicoccus	Croceicoccus mobilis
2	Bacteria	Proteobacteria	Alphaproteobacteria	Sphingomonadales	Erythrobacteraceae	Altererythrobacter	Altererythrobacter sp. 66-12
2	Bacteria	Proteobacteria	Alphaproteobacteria	Sphingomonadales	Erythrobacteraceae	Altererythrobacter	Altererythrobacter dongtanensis
2	Bacteria	Proteobacteria	Alphaproteobacteria	Sphingomonadales	Erythrobacteraceae	Altererythrobacter	Altererythrobacter atlanticus
2	Bacteria	Proteobacteria	Alphaproteobacteria	Rickettsiales	Anaplasmataceae		
2	Bacteria	Proteobacteria	Alphaproteobacteria	Rhodospirillales	unclassified Rhodospirillales	unclassified Rhodospirillales (miscellaneous)	Rhodospirillales bacterium URHD0088
2	Bacteria	Proteobacteria	Alphaproteobacteria	Rhodospirillales	unclassified Rhodospirillales	unclassified Rhodospirillales (miscellaneous)	Rhodospirillales bacterium URHD0017
2	Bacteria	Proteobacteria	Alphaproteobacteria	Rhodospirillales	Rhodospirillaceae	Magnetovibrio	Magnetovibrio blakemorei
2	Bacteria	Proteobacteria	Alphaproteobacteria	Rhodospirillales	Rhodospirillaceae	Magnetospirillum	Magnetospirillum gryphiswaldense
2	Bacteria	Proteobacteria	Alphaproteobacteria	Rhodospirillales	Rhodospirillaceae	Magnetospirillum	
2	Bacteria	Proteobacteria	Alphaproteobacteria	Rhodospirillales	Rhodospirillaceae	Azospirillum	Azospirillum oryzae
2	Bacteria	Proteobacteria	Alphaproteobacteria	Rhodospirillales	Acetobacteraceae	unclassified Acetobacteraceae	Acetobacteraceae bacterium AT-5844
2	Bacteria	Proteobacteria	Alphaproteobacteria	Rhodospirillales	Acetobacteraceae	Rubritepida	Rubritepida flocculans
2	Bacteria	Proteobacteria	Alphaproteobacteria	Rhodospirillales	Acetobacteraceae	Roseomonas	Roseomonas sp. M3
2	Bacteria	Proteobacteria	Alphaproteobacteria	Rhodospirillales	Acetobacteraceae	Acidiphilium	Acidiphilium sp. PM
2	Bacteria	Proteobacteria	Alphaproteobacteria	Rhodospirillales	Acetobacteraceae	Acetobacter	environmental samples
2	Bacteria	Proteobacteria	Alphaproteobacteria	Rhodobacterales	Rhodobacteraceae	unclassified Rhodobacteraceae	Rhodobacteraceae bacterium HLUCCA08
2	Bacteria	Proteobacteria	Alphaproteobacteria	Rhodobacterales	Rhodobacteraceae	unclassified Rhodobacteraceae	Rhodobacteraceae bacterium GWE1_64_9
2	Bacteria	Proteobacteria	Alphaproteobacteria	Rhodobacterales	Rhodobacteraceae	environmental samples	uncultured Rhodobacteraceae bacterium
2	Bacteria	Proteobacteria	Alphaproteobacteria	Rhodobacterales	Rhodobacteraceae	Tranquillimonas	Tranquillimonas alkanivorans
2	Bacteria	Proteobacteria	Alphaproteobacteria	Rhodobacterales	Rhodobacteraceae	Thalassobium	Thalassobium sp. R2A62
2	Bacteria	Proteobacteria	Alphaproteobacteria	Rhodobacterales	Rhodobacteraceae	Stappia	Stappia indica
2	Bacteria	Proteobacteria	Alphaproteobacteria	Rhodobacterales	Rhodobacteraceae	Ruegeria	Ruegeria sp. ZGT118
2	Bacteria	Proteobacteria	Alphaproteobacteria	Rhodobacterales	Rhodobacteraceae	Ruegeria	Ruegeria pomeroyi
2	Bacteria	Proteobacteria	Alphaproteobacteria	Rhodobacterales	Rhodobacteraceae	Ruegeria	Ruegeria atlantica
2	Bacteria	Proteobacteria	Alphaproteobacteria	Rhodobacterales	Rhodobacteraceae	Roseovarius	Roseovarius tolerans
2	Bacteria	Proteobacteria	Alphaproteobacteria	Rhodobacterales	Rhodobacteraceae	Roseovarius	Roseovarius pacificus
2	Bacteria	Proteobacteria	Alphaproteobacteria	Rhodobacterales	Rhodobacteraceae	Roseovarius	Roseovarius halotolerans
2	Bacteria	Proteobacteria	Alphaproteobacteria	Rhodobacterales	Rhodobacteraceae	Roseovarius	
2	Bacteria	Proteobacteria	Alphaproteobacteria	Rhodobacterales	Rhodobacteraceae	Roseinatronobacter	Roseinatronobacter thiooxidans
2	Bacteria	Proteobacteria	Alphaproteobacteria	Rhodobacterales	Rhodobacteraceae	Roseibium	Roseibium sp. TrichSKD4
2	Bacteria	Proteobacteria	Alphaproteobacteria	Rhodobacterales	Rhodobacteraceae	Rhodobacter	Rhodobacter capsulatus
2	Bacteria	Proteobacteria	Alphaproteobacteria	Rhodobacterales	Rhodobacteraceae	Rhodobacter	Rhodobacter aestuarii
2	Bacteria	Proteobacteria	Alphaproteobacteria	Rhodobacterales	Rhodobacteraceae	Rhodobacter	Candidatus Rhodobacter lobularis
2	Bacteria	Proteobacteria	Alphaproteobacteria	Rhodobacterales	Rhodobacteraceae	Puniceibacterium	Puniceibacterium sp. IMCC21224
2	Bacteria	Proteobacteria	Alphaproteobacteria	Rhodobacterales	Rhodobacteraceae	Pseudovibrio	Pseudovibrio axinellae
2	Bacteria	Proteobacteria	Alphaproteobacteria	Rhodobacterales	Rhodobacteraceae	Pseudoruegeria	Pseudoruegeria sp. SK021
2	Bacteria	Proteobacteria	Alphaproteobacteria	Rhodobacterales	Rhodobacteraceae	Pseudoruegeria	Pseudoruegeria aquimaris
2	Bacteria	Proteobacteria	Alphaproteobacteria	Rhodobacterales	Rhodobacteraceae	Pseudorhodobacter	Pseudorhodobacter ferrugineus
2	Bacteria	Proteobacteria	Alphaproteobacteria	Rhodobacterales	Rhodobacteraceae	Pseudorhodobacter	Pseudorhodobacter aquimaris
2	Bacteria	Proteobacteria	Alphaproteobacteria	Rhodobacterales	Rhodobacteraceae	Pseudopelagicola	Pseudopelagicola gijangensis
2	Bacteria	Proteobacteria	Alphaproteobacteria	Rhodobacterales	Rhodobacteraceae	Pseudooceanicola	Pseudooceanicola marinus
2	Bacteria	Proteobacteria	Alphaproteobacteria	Rhodobacterales	Rhodobacteraceae	Pleomorphobacterium	Pleomorphobacterium xiamenense
2	Bacteria	Proteobacteria	Alphaproteobacteria	Rhodobacterales	Rhodobacteraceae	Phaeobacter	Phaeobacter gallaeciensis
2	Bacteria	Proteobacteria	Alphaproteobacteria	Rhodobacterales	Rhodobacteraceae	Pelagibaca	Pelagibaca bermudensis
2	Bacteria	Proteobacteria	Alphaproteobacteria	Rhodobacterales	Rhodobacteraceae	Paracoccus	Paracoccus sphaerophysae
2	Bacteria	Proteobacteria	Alphaproteobacteria	Rhodobacterales	Rhodobacteraceae	Paracoccus	Paracoccus sp. SM22M-07
2	Bacteria	Proteobacteria	Alphaproteobacteria	Rhodobacterales	Rhodobacteraceae	Paracoccus	Paracoccus homiensis
2	Bacteria	Proteobacteria	Alphaproteobacteria	Rhodobacterales	Rhodobacteraceae	Paracoccus	Paracoccus chinensis
2	Bacteria	Proteobacteria	Alphaproteobacteria	Rhodobacterales	Rhodobacteraceae	Paracoccus	
2	Bacteria	Proteobacteria	Alphaproteobacteria	Rhodobacterales	Rhodobacteraceae	Pannonibacter	
2	Bacteria	Proteobacteria	Alphaproteobacteria	Rhodobacterales	Rhodobacteraceae	Oceanicola	Oceanicola litoreus
2	Bacteria	Proteobacteria	Alphaproteobacteria	Rhodobacterales	Rhodobacteraceae	Maribius	Maribius sp. MOLA 401
2	Bacteria	Proteobacteria	Alphaproteobacteria	Rhodobacterales	Rhodobacteraceae	Loktanella	Loktanella tamlensis
2	Bacteria	Proteobacteria	Alphaproteobacteria	Rhodobacterales	Rhodobacteraceae	Litoreibacter	Litoreibacter arenae
2	Bacteria	Proteobacteria	Alphaproteobacteria	Rhodobacterales	Rhodobacteraceae	Lentibacter	Lentibacter algarum
2	Bacteria	Proteobacteria	Alphaproteobacteria	Rhodobacterales	Rhodobacteraceae	Leisingera	Leisingera sp. ANG-M1
2	Bacteria	Proteobacteria	Alphaproteobacteria	Rhodobacterales	Rhodobacteraceae	Labrenzia	Labrenzia aggregata
2	Bacteria	Proteobacteria	Alphaproteobacteria	Rhodobacterales	Hyphomonadaceae	Hyphomonas	Hyphomonas jannaschiana
2	Bacteria	Proteobacteria	Alphaproteobacteria	Rhizobiales	unclassified Rhizobiales	unclassified Rhizobiales (miscellaneous)	Rhizobiales bacterium 68-8
2	Bacteria	Proteobacteria	Alphaproteobacteria	Rhizobiales	unclassified Rhizobiales	unclassified Rhizobiales (miscellaneous)	Rhizobiales bacterium 65-9
2	Bacteria	Proteobacteria	Alphaproteobacteria	Rhizobiales	unclassified Rhizobiales	unclassified Rhizobiales (miscellaneous)	Rhizobiales bacterium 65-79
2	Bacteria	Proteobacteria	Alphaproteobacteria	Rhizobiales	unclassified Rhizobiales	unclassified Rhizobiales (miscellaneous)	Rhizobiales bacterium 64-17
2	Bacteria	Proteobacteria	Alphaproteobacteria	Rhizobiales	unclassified Rhizobiales	Bauldia	Bauldia litoralis
2	Bacteria	Proteobacteria	Alphaproteobacteria	Rhizobiales	Xanthobacteraceae	Xanthobacter	
2	Bacteria	Proteobacteria	Alphaproteobacteria	Rhizobiales	Xanthobacteraceae	Azorhizobium	Azorhizobium caulinodans
2	Bacteria	Proteobacteria	Alphaproteobacteria	Rhizobiales	Rhodobiaceae	Tepidicaulis	Tepidicaulis marinus
2	Bacteria	Proteobacteria	Alphaproteobacteria	Rhizobiales	Rhodobiaceae	Afifella	Afifella pfennigii
2	Bacteria	Proteobacteria	Alphaproteobacteria	Rhizobiales	Rhizobiaceae	Sinorhizobium/Ensifer group	Ensifer
2	Bacteria	Proteobacteria	Alphaproteobacteria	Rhizobiales	Rhizobiaceae	Shinella	
2	Bacteria	Proteobacteria	Alphaproteobacteria	Rhizobiales	Rhizobiaceae	Rhizobium/Agrobacterium group	Rhizobium
2	Bacteria	Proteobacteria	Alphaproteobacteria	Rhizobiales	Rhizobiaceae	Rhizobium/Agrobacterium group	Rhizobium
2	Bacteria	Proteobacteria	Alphaproteobacteria	Rhizobiales	Rhizobiaceae	Rhizobium/Agrobacterium group	Rhizobium
2	Bacteria	Proteobacteria	Alphaproteobacteria	Rhizobiales	Rhizobiaceae	Rhizobium/Agrobacterium group	Agrobacterium
2	Bacteria	Proteobacteria	Alphaproteobacteria	Rhizobiales	Rhizobiaceae	Kaistia	Kaistia soli
2	Bacteria	Proteobacteria	Alphaproteobacteria	Rhizobiales	Rhizobiaceae	Kaistia	Kaistia adipata
2	Bacteria	Proteobacteria	Alphaproteobacteria	Rhizobiales	Phyllobacteriaceae	Nitratireductor	Nitratireductor indicus
2	Bacteria	Proteobacteria	Alphaproteobacteria	Rhizobiales	Phyllobacteriaceae	Mesorhizobium	Mesorhizobium sp. LC103
2	Bacteria	Proteobacteria	Alphaproteobacteria	Rhizobiales	Phyllobacteriaceae	Mesorhizobium	Mesorhizobium sp. B7
2	Bacteria	Proteobacteria	Alphaproteobacteria	Rhizobiales	Phyllobacteriaceae	Mesorhizobium	Mesorhizobium sp. 65-26
2	Bacteria	Proteobacteria	Alphaproteobacteria	Rhizobiales	Phyllobacteriaceae	Mesorhizobium	Mesorhizobium sp. 1M-11
2	Bacteria	Proteobacteria	Alphaproteobacteria	Rhizobiales	Phyllobacteriaceae	Mesorhizobium	Mesorhizobium loti
2	Bacteria	Proteobacteria	Alphaproteobacteria	Rhizobiales	Phyllobacteriaceae	Hoeflea	Hoeflea sp. BRH_c9
2	Bacteria	Proteobacteria	Alphaproteobacteria	Rhizobiales	Phyllobacteriaceae	Aminobacter	Aminobacter aminovorans
2	Bacteria	Proteobacteria	Alphaproteobacteria	Rhizobiales	Methylocystaceae	Pleomorphomonas	Pleomorphomonas oryzae
2	Bacteria	Proteobacteria	Alphaproteobacteria	Rhizobiales	Methylocystaceae	Methylocystis	Methylocystis parvus
2	Bacteria	Proteobacteria	Alphaproteobacteria	Rhizobiales	Methylocystaceae	Methylocystis	
2	Bacteria	Proteobacteria	Alphaproteobacteria	Rhizobiales	Methylobacteriaceae	Microvirga	Microvirga sp. BSC39
2	Bacteria	Proteobacteria	Alphaproteobacteria	Rhizobiales	Methylobacteriaceae	Microvirga	Microvirga ossetica
2	Bacteria	Proteobacteria	Alphaproteobacteria	Rhizobiales	Methylobacteriaceae	Microvirga	Microvirga massiliensis
2	Bacteria	Proteobacteria	Alphaproteobacteria	Rhizobiales	Methylobacteriaceae	Methylobacterium	Methylobacterium radiotolerans
2	Bacteria	Proteobacteria	Alphaproteobacteria	Rhizobiales	Hyphomicrobiaceae	Pelagibacterium	Pelagibacterium luteolum
2	Bacteria	Proteobacteria	Alphaproteobacteria	Rhizobiales	Hyphomicrobiaceae	Hyphomicrobium	Hyphomicrobium sp. SCN 65-11
2	Bacteria	Proteobacteria	Alphaproteobacteria	Rhizobiales	Hyphomicrobiaceae	Hyphomicrobium	Hyphomicrobium sp. CS1GBMeth3
2	Bacteria	Proteobacteria	Alphaproteobacteria	Rhizobiales	Hyphomicrobiaceae	Hyphomicrobium	Hyphomicrobium facile
2	Bacteria	Proteobacteria	Alphaproteobacteria	Rhizobiales	Hyphomicrobiaceae	Devosia	Devosia epidermidihirudinis
2	Bacteria	Proteobacteria	Alphaproteobacteria	Rhizobiales	Hyphomicrobiaceae	Devosia	Devosia enhydra
2	Bacteria	Proteobacteria	Alphaproteobacteria	Rhizobiales	Hyphomicrobiaceae		
2	Bacteria	Proteobacteria	Alphaproteobacteria	Rhizobiales	Bradyrhizobiaceae	Salinarimonas	Salinarimonas rosea
2	Bacteria	Proteobacteria	Alphaproteobacteria	Rhizobiales	Bradyrhizobiaceae	Bradyrhizobium	Bradyrhizobium yuanmingense
2	Bacteria	Proteobacteria	Alphaproteobacteria	Rhizobiales	Bradyrhizobiaceae	Bradyrhizobium	Bradyrhizobium lablabi
2	Bacteria	Proteobacteria	Alphaproteobacteria	Rhizobiales	Bradyrhizobiaceae	Bradyrhizobium	Bradyrhizobium erythrophlei
2	Bacteria	Proteobacteria	Alphaproteobacteria	Rhizobiales	Bradyrhizobiaceae	Bradyrhizobium	Bradyrhizobium elkanii
2	Bacteria	Proteobacteria	Alphaproteobacteria	Rhizobiales	Bradyrhizobiaceae	Bradyrhizobium	Bradyrhizobium canariense
2	Bacteria	Proteobacteria	Alphaproteobacteria	Rhizobiales	Bradyrhizobiaceae	Bosea	Bosea sp. Root483D1
2	Bacteria	Proteobacteria	Alphaproteobacteria	Rhizobiales	Bradyrhizobiaceae	Bosea	Bosea sp. Leaf344
2	Bacteria	Proteobacteria	Alphaproteobacteria	Rhizobiales	Bradyrhizobiaceae	Bosea	Bosea sp. AAP35
2	Bacteria	Proteobacteria	Alphaproteobacteria	Rhizobiales	Bradyrhizobiaceae	Bosea	Bosea sp. 117
2	Bacteria	Proteobacteria	Alphaproteobacteria	Rhizobiales	Bradyrhizobiaceae	Bosea	Bosea lupini
2	Bacteria	Proteobacteria	Alphaproteobacteria	Rhizobiales	Bradyrhizobiaceae	Afipia	Afipia felis
2	Bacteria	Proteobacteria	Alphaproteobacteria	Rhizobiales	Bradyrhizobiaceae	Afipia	
2	Bacteria	Proteobacteria	Alphaproteobacteria	Rhizobiales	Beijerinckiaceae	Methylocapsa	Methylocapsa palsarum
2	Bacteria	Proteobacteria	Alphaproteobacteria	Rhizobiales	Beijerinckiaceae	Beijerinckia	Beijerinckia indica
2	Bacteria	Proteobacteria	Alphaproteobacteria	Rhizobiales	Bartonellaceae	Bartonella	Bartonella rattaustraliani
2	Bacteria	Proteobacteria	Alphaproteobacteria	Rhizobiales	Aurantimonadaceae	Martelella	Martelella mediterranea
2	Bacteria	Proteobacteria	Alphaproteobacteria	Rhizobiales	Aurantimonadaceae	Martelella	Martelella endophytica
2	Bacteria	Proteobacteria	Alphaproteobacteria	Rhizobiales	Aurantimonadaceae	Aureimonas	Aureimonas ureilytica
2	Bacteria	Proteobacteria	Alphaproteobacteria	Rhizobiales	Aurantimonadaceae	Aurantimonas	
2	Bacteria	Proteobacteria	Alphaproteobacteria	Pelagibacterales	Pelagibacteraceae		
2	Bacteria	Proteobacteria	Alphaproteobacteria	Magnetococcales	Magnetococcaceae	Magnetofaba	Magnetofaba australis
2	Bacteria	Proteobacteria	Alphaproteobacteria	Kordiimonadales	Kordiimonadaceae	Kordiimonas	Kordiimonas gwangyangensis
2	Bacteria	Proteobacteria	Alphaproteobacteria	Holosporales	unclassified Holosporales	Caedibacter	Caedibacter sp. 37-49
2	Bacteria	Proteobacteria	Alphaproteobacteria	Caulobacterales	Caulobacteraceae	unclassified Caulobacteraceae	Caulobacteraceae bacterium TH1-2
2	Bacteria	Proteobacteria	Alphaproteobacteria	Caulobacterales	Caulobacteraceae	Phenylobacterium	Phenylobacterium sp. SCN 70-31
2	Bacteria	Proteobacteria	Alphaproteobacteria	Caulobacterales	Caulobacteraceae	Asticcacaulis	Asticcacaulis sp. YBE204
2	Bacteria	Proteobacteria	Alphaproteobacteria	Caulobacterales	Caulobacteraceae	Asticcacaulis	Asticcacaulis benevestitus
2	Bacteria	Proteobacteria	Alphaproteobacteria	Caulobacterales	Caulobacteraceae	Asticcacaulis	
2	Bacteria	Proteobacteria	Alphaproteobacteria	Caulobacterales	Caulobacteraceae		
2	Bacteria	PVC group	Verrucomicrobia	unclassified Verrucomicrobia	unclassified Verrucomicrobia (miscellaneous)	Verrucomicrobia bacterium SCGC AAA168-F10	
2	Bacteria	PVC group	Verrucomicrobia	unclassified Verrucomicrobia	unclassified Verrucomicrobia (miscellaneous)	Verrucomicrobia bacterium GWF2_62_7	
2	Bacteria	PVC group	Verrucomicrobia	unclassified Verrucomicrobia	unclassified Verrucomicrobia (miscellaneous)	Verrucomicrobia bacterium 13_1_20CM_4_55_9	
2	Bacteria	PVC group	Verrucomicrobia	Verrucomicrobiae	Verrucomicrobiales	Verrucomicrobiaceae	Verrucomicrobium
2	Bacteria	PVC group	Verrucomicrobia	Verrucomicrobiae	Verrucomicrobiales	Verrucomicrobiaceae	Verrucomicrobium
2	Bacteria	PVC group	Verrucomicrobia	Verrucomicrobiae	Verrucomicrobiales	Akkermansiaceae	Akkermansia
2	Bacteria	PVC group	Verrucomicrobia	Spartobacteria	Terrimicrobium	Terrimicrobium sacchariphilum	
2	Bacteria	PVC group	Verrucomicrobia	Opitutae	Opitutales	Opitutaceae	Opitutus
2	Bacteria	PVC group	Verrucomicrobia				
2	Bacteria	PVC group	Planctomycetes	unclassified Planctomycetes	Planctomycetes bacterium RBG_13_60_9		
2	Bacteria	PVC group	Planctomycetes	unclassified Planctomycetes	Planctomycetes bacterium GWF2_41_51		
2	Bacteria	PVC group	Planctomycetes	Planctomycetia	Planctomycetales	unclassified Planctomycetales	unclassified Planctomycetales (miscellaneous)
2	Bacteria	PVC group	Planctomycetes	Planctomycetia	Planctomycetales	Planctomycetaceae	Rhodopirellula
2	Bacteria	PVC group	Planctomycetes	Planctomycetia	Planctomycetales	Planctomycetaceae	Rhodopirellula
2	Bacteria	PVC group	Planctomycetes	Planctomycetia	Planctomycetales	Planctomycetaceae	Planctopirus
2	Bacteria	PVC group	Planctomycetes	Planctomycetia	Planctomycetales	Planctomycetaceae	Planctopirus
2	Bacteria	PVC group	Planctomycetes	Planctomycetia	Planctomycetales	Planctomycetaceae	Planctomyces
2	Bacteria	PVC group	Planctomycetes	Planctomycetia	Planctomycetales	Isosphaeraceae	Paludisphaera
2	Bacteria	PVC group	Planctomycetes	Planctomycetia	Planctomycetales	Isosphaeraceae	Isosphaera
2	Bacteria	PVC group	Planctomycetes	Planctomycetia	Planctomycetales		
2	Bacteria	PVC group	Planctomycetes	Planctomycetia	Candidatus Brocadiales	Candidatus Brocadiaceae	Candidatus Brocadia
2	Bacteria	PVC group	Chlamydiae	unclassified Chlamydiae	Chlamydiae bacterium SM23_39		
2	Bacteria	PVC group	Candidatus Omnitrophica	unclassified Candidatus Omnitrophica	Omnitrophica bacterium RIFCSPHIGHO2_02_FULL_51_18		
2	Bacteria	Nitrospirae	unclassified Nitrospirae	Nitrospirae bacterium RBG_13_39_12			
2	Bacteria	Nitrospirae	Nitrospira	Nitrospirales	Nitrospiraceae	Nitrospira	Nitrospira sp. ND1
2	Bacteria	Nitrospinae/Tectomicrobia group	Nitrospinae	Nitrospinia	Nitrospinales	Nitrospinaceae	Nitrospina
2	Bacteria	Nitrospinae/Tectomicrobia group	Candidatus Tectomicrobia	Candidatus Entotheonella	Candidatus Entotheonella sp. TSY1		
2	Bacteria	Fusobacteria	Fusobacteriia	Fusobacteriales	Fusobacteriaceae	Fusobacterium	Fusobacterium ulcerans
2	Bacteria	Fusobacteria	Fusobacteriia	Fusobacteriales	Fusobacteriaceae	Fusobacterium	
2	Bacteria	FCB group	Gemmatimonadetes	unclassified Gemmatimonadetes	Gemmatimonadetes bacterium 13_1_40CM_2_60_3		
2	Bacteria	FCB group	Bacteroidetes/Chlorobi group	Ignavibacteriae	Ignavibacteria	Ignavibacteriales	Ignavibacteriaceae
2	Bacteria	FCB group	Bacteroidetes/Chlorobi group	Bacteroidetes	unclassified Bacteroidetes	unclassified Bacteroidetes (miscellaneous)	Bacteroidetes bacterium RBG_13_43_22
2	Bacteria	FCB group	Bacteroidetes/Chlorobi group	Bacteroidetes	unclassified Bacteroidetes	unclassified Bacteroidetes (miscellaneous)	Bacteroidetes bacterium OLB9
2	Bacteria	FCB group	Bacteroidetes/Chlorobi group	Bacteroidetes	unclassified Bacteroidetes	unclassified Bacteroidetes (miscellaneous)	Bacteroidetes bacterium OLB10
2	Bacteria	FCB group	Bacteroidetes/Chlorobi group	Bacteroidetes	unclassified Bacteroidetes	unclassified Bacteroidetes (miscellaneous)	Bacteroidetes bacterium HLUCCA01
2	Bacteria	FCB group	Bacteroidetes/Chlorobi group	Bacteroidetes	unclassified Bacteroidetes	unclassified Bacteroidetes (miscellaneous)	Bacteroidetes bacterium GWF2_49_14
2	Bacteria	FCB group	Bacteroidetes/Chlorobi group	Bacteroidetes	unclassified Bacteroidetes	unclassified Bacteroidetes (miscellaneous)	Bacteroidetes bacterium 4572_128
2	Bacteria	FCB group	Bacteroidetes/Chlorobi group	Bacteroidetes	unclassified Bacteroidetes	unclassified Bacteroidetes (miscellaneous)	Bacteroidetes bacterium 4484_249
2	Bacteria	FCB group	Bacteroidetes/Chlorobi group	Bacteroidetes	unclassified Bacteroidetes	unclassified Bacteroidetes (miscellaneous)	Bacteroidetes bacterium 37-13
2	Bacteria	FCB group	Bacteroidetes/Chlorobi group	Bacteroidetes	Sphingobacteriia	Sphingobacteriales	Sphingobacteriaceae
2	Bacteria	FCB group	Bacteroidetes/Chlorobi group	Bacteroidetes	Sphingobacteriia	Sphingobacteriales	Sphingobacteriaceae
2	Bacteria	FCB group	Bacteroidetes/Chlorobi group	Bacteroidetes	Sphingobacteriia	Sphingobacteriales	Sphingobacteriaceae
2	Bacteria	FCB group	Bacteroidetes/Chlorobi group	Bacteroidetes	Sphingobacteriia	Sphingobacteriales	Sphingobacteriaceae
2	Bacteria	FCB group	Bacteroidetes/Chlorobi group	Bacteroidetes	Sphingobacteriia	Sphingobacteriales	Sphingobacteriaceae
2	Bacteria	FCB group	Bacteroidetes/Chlorobi group	Bacteroidetes	Sphingobacteriia	Sphingobacteriales	Sphingobacteriaceae
2	Bacteria	FCB group	Bacteroidetes/Chlorobi group	Bacteroidetes	Sphingobacteriia	Sphingobacteriales	Sphingobacteriaceae
2	Bacteria	FCB group	Bacteroidetes/Chlorobi group	Bacteroidetes	Sphingobacteriia	Sphingobacteriales	Sphingobacteriaceae
2	Bacteria	FCB group	Bacteroidetes/Chlorobi group	Bacteroidetes	Sphingobacteriia	Sphingobacteriales	Sphingobacteriaceae
2	Bacteria	FCB group	Bacteroidetes/Chlorobi group	Bacteroidetes	Saprospiria	Saprospirales	Lewinellaceae
2	Bacteria	FCB group	Bacteroidetes/Chlorobi group	Bacteroidetes	Flavobacteriia	Flavobacteriales	unclassified Flavobacteriales
2	Bacteria	FCB group	Bacteroidetes/Chlorobi group	Bacteroidetes	Flavobacteriia	Flavobacteriales	Schleiferiaceae
2	Bacteria	FCB group	Bacteroidetes/Chlorobi group	Bacteroidetes	Flavobacteriia	Flavobacteriales	Flavobacteriaceae
2	Bacteria	FCB group	Bacteroidetes/Chlorobi group	Bacteroidetes	Flavobacteriia	Flavobacteriales	Flavobacteriaceae
2	Bacteria	FCB group	Bacteroidetes/Chlorobi group	Bacteroidetes	Flavobacteriia	Flavobacteriales	Flavobacteriaceae
2	Bacteria	FCB group	Bacteroidetes/Chlorobi group	Bacteroidetes	Flavobacteriia	Flavobacteriales	Flavobacteriaceae
2	Bacteria	FCB group	Bacteroidetes/Chlorobi group	Bacteroidetes	Flavobacteriia	Flavobacteriales	Flavobacteriaceae
2	Bacteria	FCB group	Bacteroidetes/Chlorobi group	Bacteroidetes	Flavobacteriia	Flavobacteriales	Flavobacteriaceae
2	Bacteria	FCB group	Bacteroidetes/Chlorobi group	Bacteroidetes	Flavobacteriia	Flavobacteriales	Flavobacteriaceae
2	Bacteria	FCB group	Bacteroidetes/Chlorobi group	Bacteroidetes	Flavobacteriia	Flavobacteriales	Flavobacteriaceae
2	Bacteria	FCB group	Bacteroidetes/Chlorobi group	Bacteroidetes	Flavobacteriia	Flavobacteriales	Flavobacteriaceae
2	Bacteria	FCB group	Bacteroidetes/Chlorobi group	Bacteroidetes	Flavobacteriia	Flavobacteriales	Flavobacteriaceae
2	Bacteria	FCB group	Bacteroidetes/Chlorobi group	Bacteroidetes	Flavobacteriia	Flavobacteriales	Flavobacteriaceae
2	Bacteria	FCB group	Bacteroidetes/Chlorobi group	Bacteroidetes	Flavobacteriia	Flavobacteriales	Flavobacteriaceae
2	Bacteria	FCB group	Bacteroidetes/Chlorobi group	Bacteroidetes	Flavobacteriia	Flavobacteriales	Flavobacteriaceae
2	Bacteria	FCB group	Bacteroidetes/Chlorobi group	Bacteroidetes	Flavobacteriia	Flavobacteriales	Flavobacteriaceae
2	Bacteria	FCB group	Bacteroidetes/Chlorobi group	Bacteroidetes	Flavobacteriia	Flavobacteriales	Flavobacteriaceae
2	Bacteria	FCB group	Bacteroidetes/Chlorobi group	Bacteroidetes	Flavobacteriia	Flavobacteriales	Flavobacteriaceae
2	Bacteria	FCB group	Bacteroidetes/Chlorobi group	Bacteroidetes	Flavobacteriia	Flavobacteriales	Cryomorphaceae
2	Bacteria	FCB group	Bacteroidetes/Chlorobi group	Bacteroidetes	Flavobacteriia		
2	Bacteria	FCB group	Bacteroidetes/Chlorobi group	Bacteroidetes	Cytophagia	Cytophagales	unclassified Cytophagales
2	Bacteria	FCB group	Bacteroidetes/Chlorobi group	Bacteroidetes	Cytophagia	Cytophagales	Hymenobacteraceae
2	Bacteria	FCB group	Bacteroidetes/Chlorobi group	Bacteroidetes	Cytophagia	Cytophagales	Hymenobacteraceae
2	Bacteria	FCB group	Bacteroidetes/Chlorobi group	Bacteroidetes	Cytophagia	Cytophagales	Hymenobacteraceae
2	Bacteria	FCB group	Bacteroidetes/Chlorobi group	Bacteroidetes	Cytophagia	Cytophagales	Hymenobacteraceae
2	Bacteria	FCB group	Bacteroidetes/Chlorobi group	Bacteroidetes	Cytophagia	Cytophagales	Flammeovirgaceae
2	Bacteria	FCB group	Bacteroidetes/Chlorobi group	Bacteroidetes	Cytophagia	Cytophagales	Flammeovirgaceae
2	Bacteria	FCB group	Bacteroidetes/Chlorobi group	Bacteroidetes	Cytophagia	Cytophagales	Flammeovirgaceae
2	Bacteria	FCB group	Bacteroidetes/Chlorobi group	Bacteroidetes	Cytophagia	Cytophagales	Flammeovirgaceae
2	Bacteria	FCB group	Bacteroidetes/Chlorobi group	Bacteroidetes	Cytophagia	Cytophagales	Cytophagaceae
2	Bacteria	FCB group	Bacteroidetes/Chlorobi group	Bacteroidetes	Cytophagia	Cytophagales	Cytophagaceae
2	Bacteria	FCB group	Bacteroidetes/Chlorobi group	Bacteroidetes	Cytophagia	Cytophagales	Cytophagaceae
2	Bacteria	FCB group	Bacteroidetes/Chlorobi group	Bacteroidetes	Cytophagia	Cytophagales	Cytophagaceae
2	Bacteria	FCB group	Bacteroidetes/Chlorobi group	Bacteroidetes	Cytophagia	Cytophagales	Cytophagaceae
2	Bacteria	FCB group	Bacteroidetes/Chlorobi group	Bacteroidetes	Cytophagia	Cytophagales	Cytophagaceae
2	Bacteria	FCB group	Bacteroidetes/Chlorobi group	Bacteroidetes	Cytophagia	Cytophagales	Cytophagaceae
2	Bacteria	FCB group	Bacteroidetes/Chlorobi group	Bacteroidetes	Cytophagia	Cytophagales	Cytophagaceae
2	Bacteria	FCB group	Bacteroidetes/Chlorobi group	Bacteroidetes	Cytophagia	Cytophagales	Cyclobacteriaceae
2	Bacteria	FCB group	Bacteroidetes/Chlorobi group	Bacteroidetes	Cytophagia	Cytophagales	Cyclobacteriaceae
2	Bacteria	FCB group	Bacteroidetes/Chlorobi group	Bacteroidetes	Cytophagia	Cytophagales	Cyclobacteriaceae
2	Bacteria	FCB group	Bacteroidetes/Chlorobi group	Bacteroidetes	Cytophagia	Cytophagales	Cyclobacteriaceae
2	Bacteria	FCB group	Bacteroidetes/Chlorobi group	Bacteroidetes	Cytophagia	Cytophagales	Catalimonadaceae
2	Bacteria	FCB group	Bacteroidetes/Chlorobi group	Bacteroidetes	Cytophagia	Cytophagales	
2	Bacteria	FCB group	Bacteroidetes/Chlorobi group	Bacteroidetes	Chitinophagia	Chitinophagales	Chitinophagaceae
2	Bacteria	FCB group	Bacteroidetes/Chlorobi group	Bacteroidetes	Chitinophagia	Chitinophagales	Chitinophagaceae
2	Bacteria	FCB group	Bacteroidetes/Chlorobi group	Bacteroidetes	Chitinophagia	Chitinophagales	Chitinophagaceae
2	Bacteria	FCB group	Bacteroidetes/Chlorobi group	Bacteroidetes	Chitinophagia	Chitinophagales	Chitinophagaceae
2	Bacteria	FCB group	Bacteroidetes/Chlorobi group	Bacteroidetes	Chitinophagia	Chitinophagales	Chitinophagaceae
2	Bacteria	FCB group	Bacteroidetes/Chlorobi group	Bacteroidetes	Chitinophagia	Chitinophagales	Chitinophagaceae
2	Bacteria	FCB group	Bacteroidetes/Chlorobi group	Bacteroidetes	Chitinophagia	Chitinophagales	Chitinophagaceae
2	Bacteria	FCB group	Bacteroidetes/Chlorobi group	Bacteroidetes	Chitinophagia	Chitinophagales	Chitinophagaceae
2	Bacteria	FCB group	Bacteroidetes/Chlorobi group	Bacteroidetes	Chitinophagia	Chitinophagales	Chitinophagaceae
2	Bacteria	FCB group	Bacteroidetes/Chlorobi group	Bacteroidetes	Chitinophagia	Chitinophagales	Chitinophagaceae
2	Bacteria	FCB group	Bacteroidetes/Chlorobi group	Bacteroidetes	Chitinophagia	Chitinophagales	Chitinophagaceae
2	Bacteria	FCB group	Bacteroidetes/Chlorobi group	Bacteroidetes	Chitinophagia	Chitinophagales	Chitinophagaceae
2	Bacteria	FCB group	Bacteroidetes/Chlorobi group	Bacteroidetes	Chitinophagia	Chitinophagales	Chitinophagaceae
2	Bacteria	FCB group	Bacteroidetes/Chlorobi group	Bacteroidetes	Chitinophagia	Chitinophagales	Chitinophagaceae
2	Bacteria	FCB group	Bacteroidetes/Chlorobi group	Bacteroidetes	Bacteroidia	Marinilabiliales	Prolixibacteraceae
2	Bacteria	FCB group	Bacteroidetes/Chlorobi group	Bacteroidetes	Bacteroidia	Marinilabiliales	Marinilabiliaceae
2	Bacteria	FCB group	Bacteroidetes/Chlorobi group	Bacteroidetes	Bacteroidia	Marinilabiliales	Marinilabiliaceae
2	Bacteria	FCB group	Bacteroidetes/Chlorobi group	Bacteroidetes	Bacteroidia	Bacteroidales	Rikenellaceae
2	Bacteria	FCB group	Bacteroidetes/Chlorobi group	Bacteroidetes	Bacteroidia	Bacteroidales	Rikenellaceae
2	Bacteria	FCB group	Bacteroidetes/Chlorobi group	Bacteroidetes	Bacteroidia	Bacteroidales	Rikenellaceae
2	Bacteria	FCB group	Bacteroidetes/Chlorobi group	Bacteroidetes	Bacteroidia	Bacteroidales	Rikenellaceae
2	Bacteria	FCB group	Bacteroidetes/Chlorobi group	Bacteroidetes	Bacteroidia	Bacteroidales	Prevotellaceae
2	Bacteria	FCB group	Bacteroidetes/Chlorobi group	Bacteroidetes	Bacteroidia	Bacteroidales	Prevotellaceae
2	Bacteria	FCB group	Bacteroidetes/Chlorobi group	Bacteroidetes	Bacteroidia	Bacteroidales	Prevotellaceae
2	Bacteria	FCB group	Bacteroidetes/Chlorobi group	Bacteroidetes	Bacteroidia	Bacteroidales	Prevotellaceae
2	Bacteria	FCB group	Bacteroidetes/Chlorobi group	Bacteroidetes	Bacteroidia	Bacteroidales	Prevotellaceae
2	Bacteria	FCB group	Bacteroidetes/Chlorobi group	Bacteroidetes	Bacteroidia	Bacteroidales	Prevotellaceae
2	Bacteria	FCB group	Bacteroidetes/Chlorobi group	Bacteroidetes	Bacteroidia	Bacteroidales	Prevotellaceae
2	Bacteria	FCB group	Bacteroidetes/Chlorobi group	Bacteroidetes	Bacteroidia	Bacteroidales	Porphyromonadaceae
2	Bacteria	FCB group	Bacteroidetes/Chlorobi group	Bacteroidetes	Bacteroidia	Bacteroidales	Porphyromonadaceae
2	Bacteria	FCB group	Bacteroidetes/Chlorobi group	Bacteroidetes	Bacteroidia	Bacteroidales	Porphyromonadaceae
2	Bacteria	FCB group	Bacteroidetes/Chlorobi group	Bacteroidetes	Bacteroidia	Bacteroidales	Porphyromonadaceae
2	Bacteria	FCB group	Bacteroidetes/Chlorobi group	Bacteroidetes	Bacteroidia	Bacteroidales	Porphyromonadaceae
2	Bacteria	FCB group	Bacteroidetes/Chlorobi group	Bacteroidetes	Bacteroidia	Bacteroidales	Porphyromonadaceae
2	Bacteria	FCB group	Bacteroidetes/Chlorobi group	Bacteroidetes	Bacteroidia	Bacteroidales	Porphyromonadaceae
2	Bacteria	FCB group	Bacteroidetes/Chlorobi group	Bacteroidetes	Bacteroidia	Bacteroidales	Porphyromonadaceae
2	Bacteria	FCB group	Bacteroidetes/Chlorobi group	Bacteroidetes	Bacteroidia	Bacteroidales	Bacteroidaceae
2	Bacteria	FCB group	Bacteroidetes/Chlorobi group	Bacteroidetes	Bacteroidia	Bacteroidales	Bacteroidaceae
2	Bacteria	FCB group	Bacteroidetes/Chlorobi group	Bacteroidetes	Bacteroidia	Bacteroidales	Bacteroidaceae
2	Bacteria	FCB group	Bacteroidetes/Chlorobi group	Bacteroidetes	Bacteroidia	Bacteroidales	Bacteroidaceae
2	Bacteria	FCB group	Bacteroidetes/Chlorobi group	Bacteroidetes	Bacteroidia	Bacteroidales	Bacteroidaceae
2	Bacteria	FCB group	Bacteroidetes/Chlorobi group	Bacteroidetes	Bacteroidia	Bacteroidales	Bacteroidaceae
2	Bacteria	FCB group	Bacteroidetes/Chlorobi group	Bacteroidetes	Bacteroidia	Bacteroidales	Bacteroidaceae
2	Bacteria	FCB group	Bacteroidetes/Chlorobi group	Bacteroidetes	Bacteroidia	Bacteroidales	Bacteroidaceae
2	Bacteria	Deferribacteres	Deferribacteres	Deferribacterales	Deferribacteraceae	Denitrovibrio	Denitrovibrio acetiphilus
2	Bacteria	Deferribacteres	Deferribacteres	Deferribacterales	Deferribacteraceae	Calditerrivibrio	Calditerrivibrio nitroreducens
2	Bacteria	Acidobacteria	unclassified Acidobacteria	unclassified Acidobacteria (miscellaneous)	Acidobacteria bacterium SCN 69-37		
2	Bacteria	Acidobacteria	unclassified Acidobacteria	unclassified Acidobacteria (miscellaneous)	Acidobacteria bacterium RIFCSPLOWO2_02_FULL_65_29		
2	Bacteria	Acidobacteria	unclassified Acidobacteria	unclassified Acidobacteria (miscellaneous)	Acidobacteria bacterium 13_1_40CM_3_55_6		
2	Bacteria	Acidobacteria	unclassified Acidobacteria	unclassified Acidobacteria (miscellaneous)	Acidobacteria bacterium 13_1_20CM_4_56_7		
2	Bacteria	Acidobacteria	Acidobacteriia	Acidobacteriales	Acidobacteriaceae	unclassified Acidobacteriaceae	Acidobacteriaceae bacterium KBS 96
2	Archaea	TACK group	Crenarchaeota	Thermoprotei	Fervidicoccales	Fervidicoccaceae	Fervidicoccus
2	Archaea	Euryarchaeota	Methanomicrobia	Methanosarcinales	Methanosarcinaceae	Methanomethylovorans	Methanomethylovorans hollandica
2	unclassified viruses	Cedratvirus A11					
2	environmental samples						
2	dsDNA viruses, no RNA stage	Caudovirales	Podoviridae	Kf1virus	Edwardsiella virus KF1	Edwardsiella phage KF-1	
2	dsDNA viruses, no RNA stage	Caudovirales	Myoviridae	unclassified Myoviridae	Enterobacteria phage SfI		
2	dsDNA viruses, no RNA stage	Caudovirales	Myoviridae	Ounavirinae	Mooglevirus	unclassified Mooglevirus	Citrobacter phage Michonne
2	dsDNA viruses, no RNA stage	Caudovirales					
1	Eukaryota	Viridiplantae	Chlorophyta	prasinophytes	Mamiellophyceae	Mamiellales	Mamiellaceae
1	Eukaryota	Viridiplantae	Chlorophyta	Ulvophyceae	Ulvales	Ulvales incertae sedis	Pseudendoclonium
1	Eukaryota	Viridiplantae	Chlorophyta	Trebouxiophyceae	Microthamniales	Asterochloris	Asterochloris sp. DA2
1	Eukaryota	Viridiplantae	Chlorophyta	Chlorophyceae	Sphaeropleales	Selenastraceae	Monoraphidium
1	Eukaryota	Stramenopiles	Pelagophyceae	Pelagomonadales	Aureococcus	Aureococcus anophagefferens	
1	Eukaryota	Stramenopiles	PX clade	Phaeophyceae	Ectocarpales	Chordariaceae	Leathesia
1	Eukaryota	Stramenopiles	Bacillariophyta	Coscinodiscophyceae	Thalassiosirophycidae	Thalassiosirales	Thalassiosiraceae
1	Eukaryota	Stramenopiles	Bacillariophyta	Coscinodiscophyceae	Thalassiosirophycidae	Thalassiosirales	Thalassiosiraceae
1	Eukaryota	Stramenopiles	Bacillariophyta	Bacillariophyceae	Bacillariophycidae	Naviculales	Pinnulariaceae
1	Eukaryota	Rhodophyta	Florideophyceae	Rhodymeniales	Lomentariaceae	Semnocarpa	Semnocarpa minuta
1	Eukaryota	Rhodophyta	Florideophyceae	Nemaliales	Liagoraceae	Liagora	Liagora brachyclada
1	Eukaryota	Rhodophyta	Florideophyceae	Gigartinales	unclassified Gigartinales	Gigartinales sp. 1 GWS-2013	
1	Eukaryota	Rhodophyta	Florideophyceae	Ceramiales	Rhodomelaceae	Vertebrata	Vertebrata lanosa
1	Eukaryota	Rhodophyta	Florideophyceae	Ceramiales	Rhodomelaceae	Laurencia	
1	Eukaryota	Rhodophyta	Florideophyceae				
1	Eukaryota	Rhizaria	Foraminifera	Monothalamids	Reticulomyxidae	Reticulomyxa	Reticulomyxa filosa
1	Eukaryota	Rhizaria	Cercozoa	Imbricatea	Plasmodiophorida	Plasmodiophoridae	Plasmodiophora
1	Eukaryota	Parabasalia	Tritrichomonadida	Tritrichomonadidae	Tritrichomonas	Tritrichomonas foetus	
1	Eukaryota	Opisthokonta	Fungi	Zoopagomycota	Kickxellomycotina	Harpellales	Legeriomycetaceae
1	Eukaryota	Opisthokonta	Fungi	Neocallimastigomycota	Neocallimastigomycetes	Neocallimastigales	Neocallimastigaceae
1	Eukaryota	Opisthokonta	Fungi	Mucoromycota	Mucoromycotina	Mucorales	Mucorineae
1	Eukaryota	Opisthokonta	Fungi	Mucoromycota	Mucoromycotina	Mucorales	Cunninghamellaceae
1	Eukaryota	Opisthokonta	Fungi	Mucoromycota	Mortierellomycotina	Mortierellales	Mortierellaceae
1	Eukaryota	Opisthokonta	Fungi	Microsporidia	Microsporidia incertae sedis	Pseudoloma	Pseudoloma neurophilia
1	Eukaryota	Opisthokonta	Fungi	Microsporidia	Apansporoblastina	Nosematidae	Vittaforma
1	Eukaryota	Opisthokonta	Fungi	Dikarya	Basidiomycota	Agaricomycotina	Tremellomycetes
1	Eukaryota	Opisthokonta	Fungi	Dikarya	Basidiomycota	Agaricomycotina	Tremellomycetes
1	Eukaryota	Opisthokonta	Fungi	Dikarya	Basidiomycota	Agaricomycotina	Tremellomycetes
1	Eukaryota	Opisthokonta	Fungi	Dikarya	Basidiomycota	Agaricomycotina	Dacrymycetes
1	Eukaryota	Opisthokonta	Fungi	Dikarya	Basidiomycota	Agaricomycotina	Agaricomycetes
1	Eukaryota	Opisthokonta	Fungi	Dikarya	Basidiomycota	Agaricomycotina	Agaricomycetes
1	Eukaryota	Opisthokonta	Fungi	Dikarya	Basidiomycota	Agaricomycotina	Agaricomycetes
1	Eukaryota	Opisthokonta	Fungi	Dikarya	Basidiomycota	Agaricomycotina	Agaricomycetes
1	Eukaryota	Opisthokonta	Fungi	Dikarya	Basidiomycota	Agaricomycotina	Agaricomycetes
1	Eukaryota	Opisthokonta	Fungi	Dikarya	Basidiomycota	Agaricomycotina	Agaricomycetes
1	Eukaryota	Opisthokonta	Fungi	Dikarya	Basidiomycota	Agaricomycotina	Agaricomycetes
1	Eukaryota	Opisthokonta	Fungi	Dikarya	Basidiomycota	Agaricomycotina	Agaricomycetes

1	Eukaryota	Opisthokonta	Fungi	Dikarya	Basidiomycota	Agaricomycotina	Agaricomycetes
1	Eukaryota	Opisthokonta	Fungi	Dikarya	Basidiomycota	Agaricomycotina	Agaricomycetes
1	Eukaryota	Opisthokonta	Fungi	Dikarya	Basidiomycota	Agaricomycotina	Agaricomycetes
1	Eukaryota	Opisthokonta	Fungi	Dikarya	Basidiomycota	Agaricomycotina	Agaricomycetes
1	Eukaryota	Opisthokonta	Fungi	Dikarya	Ascomycota	saccharomyceta	Saccharomycotina
1	Eukaryota	Opisthokonta	Fungi	Dikarya	Ascomycota	saccharomyceta	Saccharomycotina
1	Eukaryota	Opisthokonta	Fungi	Dikarya	Ascomycota	saccharomyceta	Saccharomycotina
1	Eukaryota	Opisthokonta	Fungi	Dikarya	Ascomycota	saccharomyceta	Pezizomycotina
1	Eukaryota	Opisthokonta	Fungi	Dikarya	Ascomycota	saccharomyceta	Pezizomycotina
1	Eukaryota	Opisthokonta	Fungi	Dikarya	Ascomycota	saccharomyceta	Pezizomycotina
1	Eukaryota	Opisthokonta	Fungi	Dikarya	Ascomycota	saccharomyceta	Pezizomycotina
1	Eukaryota	Opisthokonta	Fungi	Dikarya	Ascomycota	saccharomyceta	Pezizomycotina
1	Eukaryota	Opisthokonta	Fungi	Dikarya	Ascomycota	saccharomyceta	Pezizomycotina
1	Eukaryota	Opisthokonta	Fungi	Dikarya	Ascomycota	saccharomyceta	Pezizomycotina
1	Eukaryota	Opisthokonta	Fungi	Dikarya	Ascomycota	saccharomyceta	Pezizomycotina
1	Eukaryota	Opisthokonta	Fungi	Dikarya	Ascomycota	saccharomyceta	Pezizomycotina
1	Eukaryota	Opisthokonta	Fungi	Dikarya	Ascomycota	saccharomyceta	Pezizomycotina
1	Eukaryota	Opisthokonta	Fungi	Dikarya	Ascomycota	saccharomyceta	Pezizomycotina
1	Eukaryota	Opisthokonta	Fungi	Dikarya	Ascomycota	saccharomyceta	Pezizomycotina
1	Eukaryota	Opisthokonta	Fungi	Dikarya	Ascomycota	saccharomyceta	Pezizomycotina
1	Eukaryota	Opisthokonta	Fungi	Dikarya	Ascomycota	saccharomyceta	Pezizomycotina
1	Eukaryota	Opisthokonta	Fungi	Dikarya	Ascomycota	saccharomyceta	Pezizomycotina
1	Eukaryota	Opisthokonta	Fungi	Dikarya	Ascomycota	saccharomyceta	
1	Eukaryota	Opisthokonta	Fungi	Dikarya	Ascomycota	Taphrinomycotina	Schizosaccharomycetes
1	Eukaryota	Opisthokonta	Fungi	Chytridiomycota	Chytridiomycetes	Chytridiales	Chytriomycetaceae
1	Eukaryota	Opisthokonta	Fungi	Blastocladiomycota	Blastocladiomycetes	Blastocladiales	Blastocladiaceae
1	Eukaryota	Opisthokonta	Fungi	Blastocladiomycota	Blastocladiomycetes	Blastocladiales	
1	Eukaryota	Haptophyceae	Isochrysidales	Noelaerhabdaceae	Emiliania	Emiliania huxleyi	Emiliania huxleyi CCMP1516
1	Eukaryota	Glaucocystophyceae	Cyanophoraceae	Cyanophora	Cyanophora paradoxa		
1	Eukaryota	Glaucocystophyceae	Cyanophoraceae	Cyanophora	Cyanophora biloba		
1	Eukaryota	Euglenozoa	Kinetoplastida	Trypanosomatidae	Trypanosoma	Trypanozoon	Trypanosoma brucei
1	Eukaryota	Cryptophyta	Cryptomonadales	Cryptomonadaceae	Cryptomonas	Cryptomonas ovata	
1	Eukaryota	Apusozoa	Apusomonadidae	Thecamonas	Thecamonas trahens	Thecamonas trahens ATCC 50062	
1	Eukaryota	Amoebozoa	Mycetozoa	Myxogastria	Trichiida	Trichiidae	Arcyria
1	Eukaryota	Amoebozoa	Mycetozoa	Dictyostelida	Dictyostelium	Dictyostelium purpureum	
1	Eukaryota	Amoebozoa	Mycetozoa	Dictyostelida	Acytostelium	Acytostelium subglobosum	Acytostelium subglobosum LB1
1	Eukaryota	Amoebozoa	Mycetozoa	Dictyostelida	Acytostelium	Acytostelium leptosomum	
1	Eukaryota	Amoebozoa	Discosea	Longamoebia	Centramoebida	Acanthamoebidae	Acanthamoeba
1	Eukaryota	Amoebozoa	Discosea	Himatismenida	Cochliopodiidae	Cochliopodium	Cochliopodium minus
1	Eukaryota	Alveolata	Ciliophora	Intramacronucleata	Spirotrichea	Stichotrichia	Sporadotrichida
1	Eukaryota	Alveolata	Ciliophora	Intramacronucleata	Spirotrichea	Stichotrichia	Sporadotrichida
1	Eukaryota	Alveolata	Ciliophora	Intramacronucleata	Oligohymenophorea	Scuticociliatia	Philasterida
1	Eukaryota	Alveolata	Ciliophora	Intramacronucleata	Oligohymenophorea	Hymenostomatida	
1	Eukaryota	Alveolata	Chromerida	Vitrella	Vitrella brassicaformis	Vitrella brassicaformis CCMP3155	
1	Eukaryota	Alveolata	Apicomplexa	Conoidasida	Gregarinasina	Eugregarinorida	Gregarinidae
1	Eukaryota	Alveolata	Apicomplexa	Conoidasida	Coccidia	Eucoccidiorida	Eimeriorina
1	Eukaryota	Alveolata	Apicomplexa	Aconoidasida	Piroplasmida	Theileriidae	Theileria
1	Eukaryota	Alveolata	Apicomplexa	Aconoidasida	Haemosporida	Plasmodiidae	Plasmodium
1	Eukaryota	Alveolata	Apicomplexa	Aconoidasida	Haemosporida	Plasmodiidae	Plasmodium
1	Bacteria	unclassified Bacteria	unclassified Bacteria (miscellaneous)	magneto-ovoid bacterium MO-1			
1	Bacteria	unclassified Bacteria	unclassified Bacteria (miscellaneous)	bacterium SCGC AG-212-C10			
1	Bacteria	unclassified Bacteria	unclassified Bacteria (miscellaneous)	bacterium P3			
1	Bacteria	unclassified Bacteria	unclassified Bacteria (miscellaneous)	bacterium JKG1			
1	Bacteria	unclassified Bacteria	unclassified Bacteria (miscellaneous)	bacterium BRH_c32			
1	Bacteria	unclassified Bacteria	unclassified Bacteria (miscellaneous)	bacterium A52C2			
1	Bacteria	unclassified Bacteria	Candidatus Moduliflexus	Candidatus Moduliflexus flocculans			
1	Bacteria	unclassified Bacteria	Bacteria candidate phyla	candidate division WWE3	candidate division WWE3 bacterium GW2011_GWB1_41_6		
1	Bacteria	unclassified Bacteria	Bacteria candidate phyla	candidate division WWE3			
1	Bacteria	unclassified Bacteria	Bacteria candidate phyla	candidate division WOR-3	candidate division WOR_3 bacterium SM23_60		
1	Bacteria	unclassified Bacteria	Bacteria candidate phyla	candidate division KSB1	unclassified candidate division KSB1	candidate division KSB1 bacterium 4484_219	
1	Bacteria	unclassified Bacteria	Bacteria candidate phyla	candidate division KSB1	unclassified candidate division KSB1	candidate division KSB1 4572_119	
1	Bacteria	unclassified Bacteria	Bacteria candidate phyla	candidate division Hyd24-12			
1	Bacteria	unclassified Bacteria	Bacteria candidate phyla	Patescibacteria group	Parcubacteria group	unclassified Parcubacteria group	Parcubacteria group bacterium GW2011_GWD2_38_11
1	Bacteria	unclassified Bacteria	Bacteria candidate phyla	Patescibacteria group	Parcubacteria group	unclassified Parcubacteria group	Parcubacteria group bacterium GW2011_GWA1_53_13
1	Bacteria	unclassified Bacteria	Bacteria candidate phyla	Patescibacteria group	Parcubacteria group	unclassified Parcubacteria group	Parcubacteria group bacterium CG2_30_44_11
1	Bacteria	unclassified Bacteria	Bacteria candidate phyla	Patescibacteria group	Parcubacteria group	Candidatus Yonathbacteria	Candidatus Yonathbacteria bacterium RIFOXYC2_FULL_47_9
1	Bacteria	unclassified Bacteria	Bacteria candidate phyla	Patescibacteria group	Parcubacteria group	Candidatus Yanofskybacteria	
1	Bacteria	unclassified Bacteria	Bacteria candidate phyla	Patescibacteria group	Parcubacteria group	Candidatus Wolfebacteria	Candidatus Wolfebacteria bacterium RIFOXYB1_FULL_54_12
1	Bacteria	unclassified Bacteria	Bacteria candidate phyla	Patescibacteria group	Parcubacteria group	Candidatus Uhrbacteria	Candidatus Uhrbacteria bacterium RIFCSPLOWO2_02_FULL_49_11
1	Bacteria	unclassified Bacteria	Bacteria candidate phyla	Patescibacteria group	Parcubacteria group	Candidatus Uhrbacteria	Candidatus Uhrbacteria bacterium GW2011_GWF2_39_13
1	Bacteria	unclassified Bacteria	Bacteria candidate phyla	Patescibacteria group	Parcubacteria group	Candidatus Uhrbacteria	
1	Bacteria	unclassified Bacteria	Bacteria candidate phyla	Patescibacteria group	Parcubacteria group	Candidatus Nealsonbacteria	Candidatus Nealsonbacteria bacterium RBG_13_36_15
1	Bacteria	unclassified Bacteria	Bacteria candidate phyla	Patescibacteria group	Parcubacteria group	Candidatus Kaiserbacteria	Candidatus Kaiserbacteria bacterium RIFCSPHIGHO2_01_FULL_53_29
1	Bacteria	unclassified Bacteria	Bacteria candidate phyla	Patescibacteria group	Parcubacteria group	Candidatus Kaiserbacteria	Candidatus Kaiserbacteria bacterium GWA2_50_9
1	Bacteria	unclassified Bacteria	Bacteria candidate phyla	Patescibacteria group	Parcubacteria group	Candidatus Kaiserbacteria	
1	Bacteria	unclassified Bacteria	Bacteria candidate phyla	Patescibacteria group	Parcubacteria group	Candidatus Colwellbacteria	
1	Bacteria	unclassified Bacteria	Bacteria candidate phyla	Patescibacteria group	Microgenomates group	Candidatus Woesebacteria	Candidatus Woesebacteria bacterium RIFCSPHIGHO2_01_FULL_41_10
1	Bacteria	unclassified Bacteria	Bacteria candidate phyla	Patescibacteria group	Microgenomates group	Candidatus Woesebacteria	
1	Bacteria	unclassified Bacteria	Bacteria candidate phyla	Patescibacteria group	Microgenomates group	Candidatus Gottesmanbacteria	Candidatus Gottesmanbacteria bacterium GW2011_GWC2_39_8
1	Bacteria	unclassified Bacteria	Bacteria candidate phyla	Candidatus Saccharibacteria	Candidatus Saccharimonas	Candidatus Saccharimonas aalborgensis	
1	Bacteria	unclassified Bacteria	Bacteria candidate phyla	Candidatus Saccharibacteria			
1	Bacteria	unclassified Bacteria	Bacteria candidate phyla	Candidatus Rokubacteria	Candidatus Rokubacteria bacterium 13_2_20CM_69_15_1		
1	Bacteria	unclassified Bacteria	Bacteria candidate phyla	Candidatus Rokubacteria	Candidatus Rokubacteria bacterium 13_1_40CM_2_68_8		
1	Bacteria	unclassified Bacteria	Bacteria candidate phyla	Candidatus Raymondbacteria			
1	Bacteria	unclassified Bacteria	Bacteria candidate phyla	Candidatus Margulisbacteria	Candidatus Margulisbacteria bacterium GWF2_35_9		
1	Bacteria	unclassified Bacteria	Bacteria candidate phyla	Candidatus Lindowbacteria			
1	Bacteria	unclassified Bacteria	Bacteria candidate phyla	Candidatus Dependentiae	unclassified Candidatus Dependentiae	candidate division TM6 bacterium GW2011_GWF2_28_16	
1	Bacteria	unclassified Bacteria	Bacteria candidate phyla	Candidatus Dependentiae	unclassified Candidatus Dependentiae		
1	Bacteria	unclassified Bacteria	Bacteria candidate phyla	Candidatus Dadabacteria			
1	Bacteria	unclassified Bacteria	Bacteria candidate phyla	Candidatus Aminicenantes	unclassified Aminicenantes	Candidatus Aminicenantes bacterium RBG_16_63_16	
1	Bacteria	environmental samples	uncultured bacterium pDL136				
1	Bacteria	environmental samples	uncultured bacterium WWRS-2005				
1	Bacteria	environmental samples	uncultured bacterium UPO38				
1	Bacteria	environmental samples	uncultured bacterium Contig785_n_1807_cl				
1	Bacteria	environmental samples	uncultured bacterium Contig117				
1	Bacteria	environmental samples	uncultured bacterium Contig116				
1	Bacteria	environmental samples	uncultured bacterium BAC10-10				
1	Bacteria	environmental samples	uncultured bacterium A1Q1_fos_4				
1	Bacteria	environmental samples	uncultured bacterium A1Q1_fos_1870				
1	Bacteria	environmental samples	uncultured bacterium A1Q1_fos_1815				
1	Bacteria	environmental samples	uncultured bacterium 162				
1	Bacteria	environmental samples	uncultured bacterium (gcode 4)				
1	Bacteria	environmental samples					
1	Bacteria	Thermotogae	Thermotogae	Thermotogales	Fervidobacteriaceae	Thermosipho	Thermosipho atlanticus
1	Bacteria	Thermotogae	Thermotogae	Petrotogales	Petrotogaceae	Marinitoga	Marinitoga piezophila
1	Bacteria	Thermotogae	Thermotogae	Petrotogales	Petrotogaceae	Marinitoga	
1	Bacteria	Thermotogae	Thermotogae	Kosmotogales	Kosmotogaceae	Mesotoga	
1	Bacteria	Thermodesulfobacteria	Thermodesulfobacteria	Thermodesulfobacteriales	Thermodesulfobacteriaceae	Thermosulfurimonas	Thermosulfurimonas dismutans
1	Bacteria	Thermodesulfobacteria	Thermodesulfobacteria	Thermodesulfobacteriales	Thermodesulfobacteriaceae	Thermodesulfatator	Thermodesulfatator atlanticus
1	Bacteria	Thermodesulfobacteria	Thermodesulfobacteria	Thermodesulfobacteriales	Thermodesulfobacteriaceae	Thermodesulfatator	
1	Bacteria	Terrabacteria group	Tenericutes	Mollicutes	Entomoplasmatales	Spiroplasmataceae	Spiroplasma
1	Bacteria	Terrabacteria group	Firmicutes	unclassified Firmicutes sensu stricto	unclassified Firmicutes sensu stricto (miscellaneous)	Firmicutes bacterium ML8_F2	
1	Bacteria	Terrabacteria group	Firmicutes	environmental samples	Firmicutes bacterium CAG:94		
1	Bacteria	Terrabacteria group	Firmicutes	environmental samples	Firmicutes bacterium CAG:65		
1	Bacteria	Terrabacteria group	Firmicutes	Tissierellia	unclassified Tissierellia	Sedimentibacter	Sedimentibacter sp. B4
1	Bacteria	Terrabacteria group	Firmicutes	Tissierellia	Tissierellales	Peptoniphilaceae	Peptoniphilus
1	Bacteria	Terrabacteria group	Firmicutes	Negativicutes	Veillonellales	Veillonellaceae	Veillonella
1	Bacteria	Terrabacteria group	Firmicutes	Negativicutes	Veillonellales	Veillonellaceae	Megasphaera
1	Bacteria	Terrabacteria group	Firmicutes	Negativicutes	Veillonellales	Veillonellaceae	
1	Bacteria	Terrabacteria group	Firmicutes	Negativicutes	Selenomonadales	Sporomusaceae	Thermosinus
1	Bacteria	Terrabacteria group	Firmicutes	Negativicutes	Selenomonadales	Sporomusaceae	Sporomusa
1	Bacteria	Terrabacteria group	Firmicutes	Negativicutes	Selenomonadales	Sporomusaceae	Pelosinus
1	Bacteria	Terrabacteria group	Firmicutes	Negativicutes	Selenomonadales	Sporomusaceae	Dendrosporobacter
1	Bacteria	Terrabacteria group	Firmicutes	Negativicutes	Selenomonadales	Sporomusaceae	Anaerosporomusa
1	Bacteria	Terrabacteria group	Firmicutes	Negativicutes	Selenomonadales	Sporomusaceae	Acetonema
1	Bacteria	Terrabacteria group	Firmicutes	Negativicutes	Selenomonadales	Selenomonadaceae	Selenomonas
1	Bacteria	Terrabacteria group	Firmicutes	Negativicutes	Selenomonadales	Selenomonadaceae	Selenomonas
1	Bacteria	Terrabacteria group	Firmicutes	Negativicutes	Selenomonadales	Selenomonadaceae	Selenomonas
1	Bacteria	Terrabacteria group	Firmicutes	Negativicutes	Selenomonadales	Selenomonadaceae	Mitsuokella
1	Bacteria	Terrabacteria group	Firmicutes	Negativicutes	Selenomonadales	Selenomonadaceae	Megamonas
1	Bacteria	Terrabacteria group	Firmicutes	Negativicutes	Acidaminococcales	Acidaminococcaceae	Phascolarctobacterium
1	Bacteria	Terrabacteria group	Firmicutes	Erysipelotrichia	Erysipelotrichales	Erysipelotrichaceae	Traorella
1	Bacteria	Terrabacteria group	Firmicutes	Erysipelotrichia	Erysipelotrichales	Erysipelotrichaceae	Erysipelothrix
1	Bacteria	Terrabacteria group	Firmicutes	Clostridia	unclassified Clostridia	unclassified Clostridia (miscellaneous)	Clostridia bacterium 41_269
1	Bacteria	Terrabacteria group	Firmicutes	Clostridia	Thermoanaerobacterales	Thermoanaerobacteraceae	Carboxydothermus
1	Bacteria	Terrabacteria group	Firmicutes	Clostridia	Thermoanaerobacterales	Thermoanaerobacteraceae	Caldanaerobacter
1	Bacteria	Terrabacteria group	Firmicutes	Clostridia	Thermoanaerobacterales	Thermoanaerobacteraceae	
1	Bacteria	Terrabacteria group	Firmicutes	Clostridia	Halanaerobiales	Halanaerobiaceae	Halanaerobium
[illegible]	[illegible]	[illegible]	[illegible]	[illegible]	[illegible]	[illegible]	[illegible]

1	Bacteria	Terrabacteria group	Actinobacteria	Actinobacteria	Pseudonocardiales	Pseudonocardiaceae	Amycolatopsis
1	Bacteria	Terrabacteria group	Actinobacteria	Actinobacteria	Pseudonocardiales	Pseudonocardiaceae	Amycolatopsis
1	Bacteria	Terrabacteria group	Actinobacteria	Actinobacteria	Pseudonocardiales	Pseudonocardiaceae	Amycolatopsis
1	Bacteria	Terrabacteria group	Actinobacteria	Actinobacteria	Pseudonocardiales	Pseudonocardiaceae	Actinosynnema
1	Bacteria	Terrabacteria group	Actinobacteria	Actinobacteria	Pseudonocardiales	Pseudonocardiaceae	Actinosynnema
1	Bacteria	Terrabacteria group	Actinobacteria	Actinobacteria	Pseudonocardiales	Pseudonocardiaceae	Actinoalloteichus
1	Bacteria	Terrabacteria group	Actinobacteria	Actinobacteria	Propionibacteriales	Propionibacteriaceae	Tessaracoccus
1	Bacteria	Terrabacteria group	Actinobacteria	Actinobacteria	Propionibacteriales	Propionibacteriaceae	Tessaracoccus
1	Bacteria	Terrabacteria group	Actinobacteria	Actinobacteria	Propionibacteriales	Propionibacteriaceae	Microlunatus
1	Bacteria	Terrabacteria group	Actinobacteria	Actinobacteria	Propionibacteriales	Propionibacteriaceae	Cutibacterium
1	Bacteria	Terrabacteria group	Actinobacteria	Actinobacteria	Propionibacteriales	Propionibacteriaceae	Acidipropionibacterium
1	Bacteria	Terrabacteria group	Actinobacteria	Actinobacteria	Propionibacteriales	Propionibacteriaceae	Acidipropionibacterium
1	Bacteria	Terrabacteria group	Actinobacteria	Actinobacteria	Propionibacteriales	Nocardioidaceae	Nocardioides
1	Bacteria	Terrabacteria group	Actinobacteria	Actinobacteria	Propionibacteriales	Nocardioidaceae	Nocardioides
1	Bacteria	Terrabacteria group	Actinobacteria	Actinobacteria	Propionibacteriales	Nocardioidaceae	Nocardioides
1	Bacteria	Terrabacteria group	Actinobacteria	Actinobacteria	Propionibacteriales	Nocardioidaceae	Nocardioides
1	Bacteria	Terrabacteria group	Actinobacteria	Actinobacteria	Propionibacteriales	Nocardioidaceae	Nocardioides
1	Bacteria	Terrabacteria group	Actinobacteria	Actinobacteria	Propionibacteriales	Nocardioidaceae	Nocardioides
1	Bacteria	Terrabacteria group	Actinobacteria	Actinobacteria	Propionibacteriales	Nocardioidaceae	Marmoricola
1	Bacteria	Terrabacteria group	Actinobacteria	Actinobacteria	Propionibacteriales	Nocardioidaceae	Kribbella
1	Bacteria	Terrabacteria group	Actinobacteria	Actinobacteria	Propionibacteriales	Nocardioidaceae	Aeromicrobium
1	Bacteria	Terrabacteria group	Actinobacteria	Actinobacteria	Propionibacteriales	Nocardioidaceae	Aeromicrobium
1	Bacteria	Terrabacteria group	Actinobacteria	Actinobacteria	Propionibacteriales	Nocardioidaceae	Actinopolymorpha
1	Bacteria	Terrabacteria group	Actinobacteria	Actinobacteria	Propionibacteriales	Nocardioidaceae	
1	Bacteria	Terrabacteria group	Actinobacteria	Actinobacteria	Micromonosporales	Micromonosporaceae	Micromonospora
1	Bacteria	Terrabacteria group	Actinobacteria	Actinobacteria	Micromonosporales	Micromonosporaceae	Hamadaea
1	Bacteria	Terrabacteria group	Actinobacteria	Actinobacteria	Micromonosporales	Micromonosporaceae	Couchioplanes
1	Bacteria	Terrabacteria group	Actinobacteria	Actinobacteria	Micromonosporales	Micromonosporaceae	Actinoplanes
1	Bacteria	Terrabacteria group	Actinobacteria	Actinobacteria	Micrococcales	Sanguibacteraceae	Sanguibacter
1	Bacteria	Terrabacteria group	Actinobacteria	Actinobacteria	Micrococcales	Promicromonosporaceae	Isoptericola
1	Bacteria	Terrabacteria group	Actinobacteria	Actinobacteria	Micrococcales	Micrococcaceae	Nesterenkonia
1	Bacteria	Terrabacteria group	Actinobacteria	Actinobacteria	Micrococcales	Micrococcaceae	Kocuria
1	Bacteria	Terrabacteria group	Actinobacteria	Actinobacteria	Micrococcales	Micrococcaceae	Arthrobacter
1	Bacteria	Terrabacteria group	Actinobacteria	Actinobacteria	Micrococcales	Micrococcaceae	Arthrobacter
1	Bacteria	Terrabacteria group	Actinobacteria	Actinobacteria	Micrococcales	Micrococcaceae	Arthrobacter
1	Bacteria	Terrabacteria group	Actinobacteria	Actinobacteria	Micrococcales	Micrococcaceae	Arthrobacter
1	Bacteria	Terrabacteria group	Actinobacteria	Actinobacteria	Micrococcales	Micrococcaceae	Arthrobacter
1	Bacteria	Terrabacteria group	Actinobacteria	Actinobacteria	Micrococcales	Microbacteriaceae	Microbacterium
1	Bacteria	Terrabacteria group	Actinobacteria	Actinobacteria	Micrococcales	Microbacteriaceae	Microbacterium
1	Bacteria	Terrabacteria group	Actinobacteria	Actinobacteria	Micrococcales	Microbacteriaceae	Leucobacter
1	Bacteria	Terrabacteria group	Actinobacteria	Actinobacteria	Micrococcales	Microbacteriaceae	Leifsonia
[illegible]	[illegible]	[illegible]	[illegible]	[illegible]	[illegible]	[illegible]	[illegible]

1	Bacteria	Proteobacteria	Gammaproteobacteria	Enterobacterales	Yersiniaceae	Serratia	Serratia rubidaea
1	Bacteria	Proteobacteria	Gammaproteobacteria	Enterobacterales	Yersiniaceae	Serratia	Serratia marcescens
1	Bacteria	Proteobacteria	Gammaproteobacteria	Enterobacterales	Yersiniaceae	Serratia	Serratia marcescens
1	Bacteria	Proteobacteria	Gammaproteobacteria	Enterobacterales	Yersiniaceae	Serratia	Serratia liquefaciens
1	Bacteria	Proteobacteria	Gammaproteobacteria	Enterobacterales	Yersiniaceae	Serratia	Serratia liquefaciens
1	Bacteria	Proteobacteria	Gammaproteobacteria	Enterobacterales	Yersiniaceae	Rahnella	Rahnella sp. Y9602
1	Bacteria	Proteobacteria	Gammaproteobacteria	Enterobacterales	Yersiniaceae	Rahnella	Rahnella aquatilis
1	Bacteria	Proteobacteria	Gammaproteobacteria	Enterobacterales	Yersiniaceae	Rahnella	Rahnella aquatilis
1	Bacteria	Proteobacteria	Gammaproteobacteria	Enterobacterales	Yersiniaceae	Nissabacter	Nissabacter archeti
1	Bacteria	Proteobacteria	Gammaproteobacteria	Enterobacterales	Pectobacteriaceae	Sodalis	Sodalis glossinidius
1	Bacteria	Proteobacteria	Gammaproteobacteria	Enterobacterales	Pectobacteriaceae	Sodalis	
1	Bacteria	Proteobacteria	Gammaproteobacteria	Enterobacterales	Pectobacteriaceae	Pectobacterium	Pectobacterium wasabiae
1	Bacteria	Proteobacteria	Gammaproteobacteria	Enterobacterales	Pectobacteriaceae	Pectobacterium	Pectobacterium wasabiae
1	Bacteria	Proteobacteria	Gammaproteobacteria	Enterobacterales	Pectobacteriaceae	Pectobacterium	Pectobacterium parmentieri
1	Bacteria	Proteobacteria	Gammaproteobacteria	Enterobacterales	Pectobacteriaceae	Dickeya	Dickeya dadantii
1	Bacteria	Proteobacteria	Gammaproteobacteria	Enterobacterales	Pectobacteriaceae	Brenneria	Brenneria sp. EniD312
1	Bacteria	Proteobacteria	Gammaproteobacteria	Enterobacterales	Pectobacteriaceae		
1	Bacteria	Proteobacteria	Gammaproteobacteria	Enterobacterales	Morganellaceae	Xenorhabdus	Xenorhabdus khoisanae
1	Bacteria	Proteobacteria	Gammaproteobacteria	Enterobacterales	Morganellaceae	Xenorhabdus	Xenorhabdus hominickii
1	Bacteria	Proteobacteria	Gammaproteobacteria	Enterobacterales	Morganellaceae	Xenorhabdus	Xenorhabdus eapokensis
1	Bacteria	Proteobacteria	Gammaproteobacteria	Enterobacterales	Morganellaceae	Xenorhabdus	Xenorhabdus doucetiae
1	Bacteria	Proteobacteria	Gammaproteobacteria	Enterobacterales	Morganellaceae	Xenorhabdus	Xenorhabdus bovienii
1	Bacteria	Proteobacteria	Gammaproteobacteria	Enterobacterales	Morganellaceae	Providencia	Providencia rettgeri
1	Bacteria	Proteobacteria	Gammaproteobacteria	Enterobacterales	Morganellaceae	Providencia	Providencia burhodogranariea
1	Bacteria	Proteobacteria	Gammaproteobacteria	Enterobacterales	Morganellaceae	Proteus	Proteus vulgaris
1	Bacteria	Proteobacteria	Gammaproteobacteria	Enterobacterales	Morganellaceae	Photorhabdus	Photorhabdus luminescens
1	Bacteria	Proteobacteria	Gammaproteobacteria	Enterobacterales	Morganellaceae	Photorhabdus	Photorhabdus heterorhabditis
1	Bacteria	Proteobacteria	Gammaproteobacteria	Enterobacterales	Morganellaceae	Morganella	Morganella morganii
1	Bacteria	Proteobacteria	Gammaproteobacteria	Enterobacterales	Morganellaceae	Moellerella	Moellerella wisconsensis
1	Bacteria	Proteobacteria	Gammaproteobacteria	Enterobacterales	Hafniaceae	Obesumbacterium	Obesumbacterium proteus
1	Bacteria	Proteobacteria	Gammaproteobacteria	Enterobacterales	Hafniaceae	Hafnia	Hafnia sp. HMSC23F03
1	Bacteria	Proteobacteria	Gammaproteobacteria	Enterobacterales	Hafniaceae	Hafnia	Hafnia paralvei
1	Bacteria	Proteobacteria	Gammaproteobacteria	Enterobacterales	Hafniaceae	Hafnia	Hafnia alvei
1	Bacteria	Proteobacteria	Gammaproteobacteria	Enterobacterales	Hafniaceae	Edwardsiella	Edwardsiella tarda
1	Bacteria	Proteobacteria	Gammaproteobacteria	Enterobacterales	Hafniaceae	Edwardsiella	Edwardsiella piscicida
1	Bacteria	Proteobacteria	Gammaproteobacteria	Enterobacterales	Erwiniaceae	Pantoea	Pantoea wallisii
1	Bacteria	Proteobacteria	Gammaproteobacteria	Enterobacterales	Erwiniaceae	Pantoea	Pantoea stewartii
1	Bacteria	Proteobacteria	Gammaproteobacteria	Enterobacterales	Erwiniaceae	Pantoea	Pantoea stewartii
1	Bacteria	Proteobacteria	Gammaproteobacteria	Enterobacterales	Erwiniaceae	Pantoea	Pantoea sp. RIT-PI-b
1	Bacteria	Proteobacteria	Gammaproteobacteria	Enterobacterales	Erwiniaceae	Pantoea	Pantoea sp. PSNIH2
1	Bacteria	Proteobacteria	Gammaproteobacteria	Enterobacterales	Erwiniaceae	Pantoea	Pantoea sp. OV426
1	Bacteria	Proteobacteria	Gammaproteobacteria	Enterobacterales	Erwiniaceae	Pantoea	Pantoea sp. IMH
1	Bacteria	Proteobacteria	Gammaproteobacteria	Enterobacterales	Erwiniaceae	Pantoea	Pantoea septica
1	Bacteria	Proteobacteria	Gammaproteobacteria	Enterobacterales	Erwiniaceae	Pantoea	Pantoea rodasii
1	Bacteria	Proteobacteria	Gammaproteobacteria	Enterobacterales	Erwiniaceae	Erwinia	Erwinia tasmaniensis
1	Bacteria	Proteobacteria	Gammaproteobacteria	Enterobacterales	Erwiniaceae	Erwinia	Erwinia sp. Leaf53
1	Bacteria	Proteobacteria	Gammaproteobacteria	Enterobacterales	Erwiniaceae	Erwinia	Erwinia piriflorinigrans
1	Bacteria	Proteobacteria	Gammaproteobacteria	Enterobacterales	Erwiniaceae	Erwinia	Erwinia mallotivora
1	Bacteria	Proteobacteria	Gammaproteobacteria	Enterobacterales	Erwiniaceae	Erwinia	Erwinia amylovora
1	Bacteria	Proteobacteria	Gammaproteobacteria	Enterobacterales	Enterobacteriaceae	unclassified Enterobacteriaceae	unclassified Enterobacteriaceae (miscellaneous)
1	Bacteria	Proteobacteria	Gammaproteobacteria	Enterobacterales	Enterobacteriaceae	unclassified Enterobacteriaceae	unclassified Enterobacteriaceae (miscellaneous)
1	Bacteria	Proteobacteria	Gammaproteobacteria	Enterobacterales	Enterobacteriaceae	unclassified Enterobacteriaceae	ant, tsetse, mealybug, aphid, etc. endosymbionts
1	Bacteria	Proteobacteria	Gammaproteobacteria	Enterobacterales	Enterobacteriaceae	unclassified Enterobacteriaceae	ant, tsetse, mealybug, aphid, etc. endosymbionts
1	Bacteria	Proteobacteria	Gammaproteobacteria	Enterobacterales	Enterobacteriaceae	unclassified Enterobacteriaceae	ant, tsetse, mealybug, aphid, etc. endosymbionts
1	Bacteria	Proteobacteria	Gammaproteobacteria	Enterobacterales	Enterobacteriaceae	unclassified Enterobacteriaceae	ant, tsetse, mealybug, aphid, etc. endosymbionts
1	Bacteria	Proteobacteria	Gammaproteobacteria	Enterobacterales	Enterobacteriaceae	Shigella	Shigella sp. FC2833
1	Bacteria	Proteobacteria	Gammaproteobacteria	Enterobacterales	Enterobacteriaceae	Shigella	Shigella sp. FC2383
1	Bacteria	Proteobacteria	Gammaproteobacteria	Enterobacterales	Enterobacteriaceae	Shigella	Shigella sonnei
1	Bacteria	Proteobacteria	Gammaproteobacteria	Enterobacterales	Enterobacteriaceae	Shigella	Shigella flexneri
1	Bacteria	Proteobacteria	Gammaproteobacteria	Enterobacterales	Enterobacteriaceae	Shigella	Shigella flexneri
1	Bacteria	Proteobacteria	Gammaproteobacteria	Enterobacterales	Enterobacteriaceae	Shigella	Shigella flexneri
1	Bacteria	Proteobacteria	Gammaproteobacteria	Enterobacterales	Enterobacteriaceae	Shigella	Shigella flexneri
1	Bacteria	Proteobacteria	Gammaproteobacteria	Enterobacterales	Enterobacteriaceae	Shigella	Shigella flexneri
1	Bacteria	Proteobacteria	Gammaproteobacteria	Enterobacterales	Enterobacteriaceae	Shigella	Shigella flexneri
1	Bacteria	Proteobacteria	Gammaproteobacteria	Enterobacterales	Enterobacteriaceae	Shigella	Shigella flexneri
1	Bacteria	Proteobacteria	Gammaproteobacteria	Enterobacterales	Enterobacteriaceae	Shigella	Shigella dysenteriae
1	Bacteria	Proteobacteria	Gammaproteobacteria	Enterobacterales	Enterobacteriaceae	Shigella	Shigella dysenteriae
1	Bacteria	Proteobacteria	Gammaproteobacteria	Enterobacterales	Enterobacteriaceae	Shigella	Shigella boydii
1	Bacteria	Proteobacteria	Gammaproteobacteria	Enterobacterales	Enterobacteriaceae	Salmonella	Salmonella enterica
1	Bacteria	Proteobacteria	Gammaproteobacteria	Enterobacterales	Enterobacteriaceae	Salmonella	Salmonella enterica
1	Bacteria	Proteobacteria	Gammaproteobacteria	Enterobacterales	Enterobacteriaceae	Salmonella	Salmonella enterica
1	Bacteria	Proteobacteria	Gammaproteobacteria	Enterobacterales	Enterobacteriaceae	Salmonella	Salmonella enterica
1	Bacteria	Proteobacteria	Gammaproteobacteria	Enterobacterales	Enterobacteriaceae	Salmonella	Salmonella enterica
1	Bacteria	Proteobacteria	Gammaproteobacteria	Enterobacterales	Enterobacteriaceae	Salmonella	Salmonella enterica
1	Bacteria	Proteobacteria	Gammaproteobacteria	Enterobacterales	Enterobacteriaceae	Salmonella	Salmonella enterica
1	Bacteria	Proteobacteria	Gammaproteobacteria	Enterobacterales	Enterobacteriaceae	Salmonella	Salmonella enterica
1	Bacteria	Proteobacteria	Gammaproteobacteria	Enterobacterales	Enterobacteriaceae	Salmonella	Salmonella enterica
[illegible]	[illegible]	[illegible]	[illegible]	[illegible]	[illegible]	[illegible]	[illegible]
1	Bacteria	Proteobacteria	Gammaproteobacteria	Chromatiales	Ectothiorhodospiraceae	Halorhodospira	
1	Bacteria	Proteobacteria	Gammaproteobacteria	Chromatiales	Chromatiaceae	unclassified Chromatiaceae	Chromatiaceae bacterium 2141T.STBD.0c.01a
1	Bacteria	Proteobacteria	Gammaproteobacteria	Chromatiales	Chromatiaceae	Thiorhodococcus	Thiorhodococcus drewsii
1	Bacteria	Proteobacteria	Gammaproteobacteria	Chromatiales	Chromatiaceae	Thiocystis	Thiocystis violascens
1	Bacteria	Proteobacteria	Gammaproteobacteria	Chromatiales	Chromatiaceae	Thiocapsa	Thiocapsa sp. KS1
1	Bacteria	Proteobacteria	Gammaproteobacteria	Chromatiales	Chromatiaceae	Thiocapsa	Thiocapsa roseopersicina
1	Bacteria	Proteobacteria	Gammaproteobacteria	Chromatiales	Chromatiaceae	Rheinheimera	Rheinheimera sp. SA_1
1	Bacteria	Proteobacteria	Gammaproteobacteria	Chromatiales	Chromatiaceae	Rheinheimera	Rheinheimera sp. F8
1	Bacteria	Proteobacteria	Gammaproteobacteria	Chromatiales	Chromatiaceae	Rheinheimera	
1	Bacteria	Proteobacteria	Gammaproteobacteria	Chromatiales	Chromatiaceae	Lamprocystis	Lamprocystis purpurea
1	Bacteria	Proteobacteria	Gammaproteobacteria	Chromatiales	Chromatiaceae	Arsukibacterium	
1	Bacteria	Proteobacteria	Gammaproteobacteria	Chromatiales	Chromatiaceae	Allochromatium	Allochromatium warmingii
1	Bacteria	Proteobacteria	Gammaproteobacteria	Chromatiales			
1	Bacteria	Proteobacteria	Gammaproteobacteria	Cellvibrionales	Spongiibacteraceae	Spongiibacter	Spongiibacter tropicus
1	Bacteria	Proteobacteria	Gammaproteobacteria	Cellvibrionales	Spongiibacteraceae	Spongiibacter	Spongiibacter sp. IMCC21906
1	Bacteria	Proteobacteria	Gammaproteobacteria	Cellvibrionales	Spongiibacteraceae	Oceanicoccus	Oceanicoccus sagamiensis
1	Bacteria	Proteobacteria	Gammaproteobacteria	Cellvibrionales	Spongiibacteraceae	Dasania	Dasania marina
1	Bacteria	Proteobacteria	Gammaproteobacteria	Cellvibrionales	Spongiibacteraceae		
1	Bacteria	Proteobacteria	Gammaproteobacteria	Cellvibrionales	Microbulbiferaceae	Microbulbifer	Microbulbifer variabilis
1	Bacteria	Proteobacteria	Gammaproteobacteria	Cellvibrionales	Microbulbiferaceae	Microbulbifer	Microbulbifer thermotolerans
1	Bacteria	Proteobacteria	Gammaproteobacteria	Cellvibrionales	Microbulbiferaceae	Microbulbifer	Microbulbifer marinus
1	Bacteria	Proteobacteria	Gammaproteobacteria	Cellvibrionales	Microbulbiferaceae	Microbulbifer	
1	Bacteria	Proteobacteria	Gammaproteobacteria	Cellvibrionales	Halieaceae	unclassified Halieaceae	marine gamma proteobacterium HTCC2148
1	Bacteria	Proteobacteria	Gammaproteobacteria	Cellvibrionales	Halieaceae	Halioglobus	
1	Bacteria	Proteobacteria	Gammaproteobacteria	Cellvibrionales	Halieaceae	Haliea	Haliea salexigens
1	Bacteria	Proteobacteria	Gammaproteobacteria	Cellvibrionales	Cellvibrionaceae	Marinimicrobium	Marinimicrobium sp. LS-A18
1	Bacteria	Proteobacteria	Gammaproteobacteria	Cellvibrionales	Cellvibrionaceae	Gilvimarinus	Gilvimarinus polysaccharolyticus
1	Bacteria	Proteobacteria	Gammaproteobacteria	Cellvibrionales	Cellvibrionaceae	Gilvimarinus	Gilvimarinus chinensis
1	Bacteria	Proteobacteria	Gammaproteobacteria	Cellvibrionales	Cellvibrionaceae	Cellvibrio	Cellvibrio mixtus
1	Bacteria	Proteobacteria	Gammaproteobacteria	Cellvibrionales			
1	Bacteria	Proteobacteria	Gammaproteobacteria	Cardiobacteriales	Cardiobacteriaceae	Cardiobacterium	Cardiobacterium valvarum
1	Bacteria	Proteobacteria	Gammaproteobacteria	Alteromonadales	Shewanellaceae	Shewanella	Shewanella sp. SACH
1	Bacteria	Proteobacteria	Gammaproteobacteria	Alteromonadales	Shewanellaceae	Shewanella	Shewanella sp. ANA-3
1	Bacteria	Proteobacteria	Gammaproteobacteria	Alteromonadales	Shewanellaceae	Shewanella	Shewanella marina
1	Bacteria	Proteobacteria	Gammaproteobacteria	Alteromonadales	Shewanellaceae	Shewanella	Shewanella halifaxensis
1	Bacteria	Proteobacteria	Gammaproteobacteria	Alteromonadales	Shewanellaceae	Shewanella	Shewanella frigidimarina
1	Bacteria	Proteobacteria	Gammaproteobacteria	Alteromonadales	Shewanellaceae	Shewanella	Shewanella decolorationis
1	Bacteria	Proteobacteria	Gammaproteobacteria	Alteromonadales	Shewanellaceae	Shewanella	Shewanella colwelliana
1	Bacteria	Proteobacteria	Gammaproteobacteria	Alteromonadales	Shewanellaceae	Shewanella	Shewanella baltica
1	Bacteria	Proteobacteria	Gammaproteobacteria	Alteromonadales	Shewanellaceae	Shewanella	Shewanella algae
1	Bacteria	Proteobacteria	Gammaproteobacteria	Alteromonadales	Psychromonadaceae	Psychromonas	Psychromonas sp. CNPT3
1	Bacteria	Proteobacteria	Gammaproteobacteria	Alteromonadales	Psychromonadaceae	Psychromonas	Psychromonas aquimarina
1	Bacteria	Proteobacteria	Gammaproteobacteria	Alteromonadales	Pseudoalteromonadaceae	Pseudoalteromonas	Pseudoalteromonas sp. R3
1	Bacteria	Proteobacteria	Gammaproteobacteria	Alteromonadales	Pseudoalteromonadaceae	Pseudoalteromonas	Pseudoalteromonas sp. BSi20311
1	Bacteria	Proteobacteria	Gammaproteobacteria	Alteromonadales	Pseudoalteromonadaceae	Pseudoalteromonas	Pseudoalteromonas elyakovii
1	Bacteria	Proteobacteria	Gammaproteobacteria	Alteromonadales	Pseudoalteromonadaceae	Pseudoalteromonas	Pseudoalteromonas denitrificans
1	Bacteria	Proteobacteria	Gammaproteobacteria	Alteromonadales	Pseudoalteromonadaceae	Pseudoalteromonas	Pseudoalteromonas aliena
1	Bacteria	Proteobacteria	Gammaproteobacteria	Alteromonadales	Moritellaceae	Moritella	Moritella marina
1	Bacteria	Proteobacteria	Gammaproteobacteria	Alteromonadales	Moritellaceae	Moritella	
1	Bacteria	Proteobacteria	Gammaproteobacteria	Alteromonadales	Idiomarinaceae	unclassified Idiomarinaceae	Idiomarinaceae bacterium HL-53
1	Bacteria	Proteobacteria	Gammaproteobacteria	Alteromonadales	Colwelliaceae	Thalassotalea	Thalassotalea agarivorans
1	Bacteria	Proteobacteria	Gammaproteobacteria	Alteromonadales	Colwelliaceae		
1	Bacteria	Proteobacteria	Gammaproteobacteria	Alteromonadales	Alteromonadaceae	Salinimonas	Salinimonas chungwhensis
1	Bacteria	Proteobacteria	Gammaproteobacteria	Alteromonadales	Alteromonadaceae	Paraglaciecola	Paraglaciecola psychrophila
1	Bacteria	Proteobacteria	Gammaproteobacteria	Alteromonadales	Alteromonadaceae	Paraglaciecola	Paraglaciecola arctica
1	Bacteria	Proteobacteria	Gammaproteobacteria	Alteromonadales	Alteromonadaceae	Marinobacter	Marinobacter sp. HL-58
1	Bacteria	Proteobacteria	Gammaproteobacteria	Alteromonadales	Alteromonadaceae	Marinobacter	Marinobacter sp. AC-23
1	Bacteria	Proteobacteria	Gammaproteobacteria	Alteromonadales	Alteromonadaceae	Marinobacter	Marinobacter similis
1	Bacteria	Proteobacteria	Gammaproteobacteria	Alteromonadales	Alteromonadaceae	Marinobacter	Marinobacter salinus
1	Bacteria	Proteobacteria	Gammaproteobacteria	Alteromonadales	Alteromonadaceae	Marinobacter	Marinobacter pelagius
1	Bacteria	Proteobacteria	Gammaproteobacteria	Alteromonadales	Alteromonadaceae	Marinobacter	Marinobacter nanhaiticus
1	Bacteria	Proteobacteria	Gammaproteobacteria	Alteromonadales	Alteromonadaceae	Catenovulum	Catenovulum agarivorans
1	Bacteria	Proteobacteria	Gammaproteobacteria	Alteromonadales	Alteromonadaceae	Alteromonas	Alteromonas mediterranea
1	Bacteria	Proteobacteria	Gammaproteobacteria	Alteromonadales	Alteromonadaceae	Alteromonas	Alteromonas macleodii
1	Bacteria	Proteobacteria	Gammaproteobacteria	Alteromonadales	Alteromonadaceae	Alteromonas	
1	Bacteria	Proteobacteria	Gammaproteobacteria	Alteromonadales	Alteromonadaceae	Alishewanella	Alishewanella agri
1	Bacteria	Proteobacteria	Gammaproteobacteria	Alteromonadales	Alteromonadaceae	Aliiglaciecola	Aliiglaciecola lipolytica
1	Bacteria	Proteobacteria	Gammaproteobacteria	Alteromonadales			
1	Bacteria	Proteobacteria	Gammaproteobacteria	Aeromonadales	Aeromonadaceae	Oceanimonas	Oceanimonas smirnovii
1	Bacteria	Proteobacteria	Gammaproteobacteria	Aeromonadales	Aeromonadaceae	Aeromonas	Aeromonas sp. CD3
1	Bacteria	Proteobacteria	Gammaproteobacteria	Aeromonadales	Aeromonadaceae	Aeromonas	Aeromonas sp. ANNP30
1	Bacteria	Proteobacteria	Gammaproteobacteria	Aeromonadales	Aeromonadaceae	Aeromonas	Aeromonas hydrophila
1	Bacteria	Proteobacteria	Candidatus Muproteobacteria	Candidatus Muproteobacteria bacterium RIFCSPHIGHO2_01_60_12			
1	Bacteria	Proteobacteria	Candidatus Muproteobacteria	Candidatus Muproteobacteria bacterium RBG_16_60_9			
1	Bacteria	Proteobacteria	Betaproteobacteria	unclassified Betaproteobacteria	unclassified Betaproteobacteria (miscellaneous)	beta proteobacterium AAP51	
1	Bacteria	Proteobacteria	Betaproteobacteria	unclassified Betaproteobacteria	unclassified Betaproteobacteria (miscellaneous)	Betaproteobacteria bacterium SCGC AG-212-J23	
1	Bacteria	Proteobacteria	Betaproteobacteria	unclassified Betaproteobacteria	unclassified Betaproteobacteria (miscellaneous)	Betaproteobacteria bacterium RIFCSPLOWO2_12_FULL_68_20	
1	Bacteria	Proteobacteria	Betaproteobacteria	unclassified Betaproteobacteria	unclassified Betaproteobacteria (miscellaneous)	Betaproteobacteria bacterium RIFCSPLOWO2_12_FULL_68_19	
1	Bacteria	Proteobacteria	Betaproteobacteria	unclassified Betaproteobacteria	unclassified Betaproteobacteria (miscellaneous)	Betaproteobacteria bacterium RIFCSPLOWO2_12_FULL_62_58	
1	Bacteria	Proteobacteria	Betaproteobacteria	unclassified Betaproteobacteria	unclassified Betaproteobacteria (miscellaneous)	Betaproteobacteria bacterium RIFCSPLOWO2_02_FULL_67_26	
1	Bacteria	Proteobacteria	Betaproteobacteria	unclassified Betaproteobacteria	unclassified Betaproteobacteria (miscellaneous)	Betaproteobacteria bacterium RBG_19FT_COMBO_58_11	
1	Bacteria	Proteobacteria	Betaproteobacteria	unclassified Betaproteobacteria	unclassified Betaproteobacteria (miscellaneous)	Betaproteobacteria bacterium RBG_16_58_11	
1	Bacteria	Proteobacteria	Betaproteobacteria	unclassified Betaproteobacteria	unclassified Betaproteobacteria (miscellaneous)	Betaproteobacteria bacterium CG2_30_68_42	
1	Bacteria	Proteobacteria	Betaproteobacteria	unclassified Betaproteobacteria	unclassified Betaproteobacteria (miscellaneous)	Betaproteobacteria bacterium CG2_30_59_46	
1	Bacteria	Proteobacteria	Betaproteobacteria	unclassified Betaproteobacteria	Kinetoplastibacterium	Kinetoplastibacterium blastocrithidii	
1	Bacteria	Proteobacteria	Betaproteobacteria	unclassified Betaproteobacteria	Kinetoplastibacterium	Candidatus Kinetoplastibacterium oncopeltii	
1	Bacteria	Proteobacteria	Betaproteobacteria	unclassified Betaproteobacteria	Candidatus Accumulibacter	Candidatus Accumulibacter sp. SK-12	
1	Bacteria	Proteobacteria	Betaproteobacteria	unclassified Betaproteobacteria	Candidatus Accumulibacter	Candidatus Accumulibacter sp. SK-01	
1	Bacteria	Proteobacteria	Betaproteobacteria	unclassified Betaproteobacteria	Candidatus Accumulibacter		
1	Bacteria	Proteobacteria	Betaproteobacteria	environmental samples	uncultured beta proteobacterium CBNPD1 BAC clone 578		
1	Bacteria	Proteobacteria	Betaproteobacteria	Sulfuricellales	Sulfuricellaceae	Sulfuricella	
1	Bacteria	Proteobacteria	Betaproteobacteria	Rhodocyclales	Rhodocyclaceae	Thauera	Thauera sp. 63
1	Bacteria	Proteobacteria	Betaproteobacteria	Rhodocyclales	Rhodocyclaceae	Thauera	Thauera sp. 28
1	Bacteria	Proteobacteria	Betaproteobacteria	Rhodocyclales	Rhodocyclaceae	Thauera	Thauera aminoaromatica
1	Bacteria	Proteobacteria	Betaproteobacteria	Rhodocyclales	Rhodocyclaceae	Sulfuritalea	Sulfuritalea hydrogenivorans
1	Bacteria	Proteobacteria	Betaproteobacteria	Rhodocyclales	Rhodocyclaceae	Sterolibacterium	Sterolibacterium denitrificans
1	Bacteria	Proteobacteria	Betaproteobacteria	Rhodocyclales	Rhodocyclaceae	Propionivibrio	
1	Bacteria	Proteobacteria	Betaproteobacteria	Rhodocyclales	Rhodocyclaceae	Methyloversatilis	Methyloversatilis universalis
1	Bacteria	Proteobacteria	Betaproteobacteria	Rhodocyclales	Rhodocyclaceae	Methyloversatilis	Methyloversatilis thermotolerans
1	Bacteria	Proteobacteria	Betaproteobacteria	Rhodocyclales	Rhodocyclaceae	Methyloversatilis	
1	Bacteria	Proteobacteria	Betaproteobacteria	Rhodocyclales	Rhodocyclaceae	Dechloromonas	Dechloromonas agitata

1	Bacteria	Proteobacteria	Betaproteobacteria	Rhodocyclales	Rhodocyclaceae	Azoarcus	Azoarcus sp. KH32C
1	Bacteria	Proteobacteria	Betaproteobacteria	Nitrosomonadales	unclassified Nitrosomonadales	Nitrosomonadales bacterium SCN 54-20	
1	Bacteria	Proteobacteria	Betaproteobacteria	Nitrosomonadales	Nitrosomonadaceae	Nitrosovibrio	Nitrosovibrio sp. Nv6
1	Bacteria	Proteobacteria	Betaproteobacteria	Nitrosomonadales	Nitrosomonadaceae	Nitrosospira	Nitrosospira multiformis
1	Bacteria	Proteobacteria	Betaproteobacteria	Nitrosomonadales	Nitrosomonadaceae	Nitrosospira	Nitrosospira briensis
1	Bacteria	Proteobacteria	Betaproteobacteria	Nitrosomonadales	Nitrosomonadaceae	Nitrosospira	
1	Bacteria	Proteobacteria	Betaproteobacteria	Nitrosomonadales	Nitrosomonadaceae	Nitrosomonas	Nitrosomonas sp. Nm34
1	Bacteria	Proteobacteria	Betaproteobacteria	Nitrosomonadales	Nitrosomonadaceae	Nitrosomonas	Nitrosomonas sp. AL212
1	Bacteria	Proteobacteria	Betaproteobacteria	Nitrosomonadales	Nitrosomonadaceae	Nitrosomonas	Nitrosomonas mobilis
1	Bacteria	Proteobacteria	Betaproteobacteria	Neisseriales	Neisseriaceae	Simonsiella	Simonsiella muelleri
1	Bacteria	Proteobacteria	Betaproteobacteria	Neisseriales	Neisseriaceae	Neisseria	Neisseria sp. oral taxon 020
1	Bacteria	Proteobacteria	Betaproteobacteria	Neisseriales	Neisseriaceae	Neisseria	Neisseria meningitidis
1	Bacteria	Proteobacteria	Betaproteobacteria	Neisseriales	Neisseriaceae	Neisseria	Neisseria animaloris
1	Bacteria	Proteobacteria	Betaproteobacteria	Neisseriales	Neisseriaceae	Kingella	Kingella kingae
1	Bacteria	Proteobacteria	Betaproteobacteria	Neisseriales	Neisseriaceae	Eikenella	Eikenella sp. NML130454
1	Bacteria	Proteobacteria	Betaproteobacteria	Neisseriales	Neisseriaceae	Eikenella	
1	Bacteria	Proteobacteria	Betaproteobacteria	Neisseriales	Chromobacteriaceae	Pseudogulbenkiania	Pseudogulbenkiania sp. NH8B
1	Bacteria	Proteobacteria	Betaproteobacteria	Neisseriales	Chromobacteriaceae	Microvirgula	Microvirgula aerodenitrificans
1	Bacteria	Proteobacteria	Betaproteobacteria	Neisseriales	Chromobacteriaceae	Gulbenkiania	Gulbenkiania indica
1	Bacteria	Proteobacteria	Betaproteobacteria	Neisseriales	Chromobacteriaceae	Deefgea	Deefgea rivuli
1	Bacteria	Proteobacteria	Betaproteobacteria	Neisseriales	Chromobacteriaceae	Chromobacterium group	Chromobacterium
1	Bacteria	Proteobacteria	Betaproteobacteria	Neisseriales	Chromobacteriaceae	Chromobacterium group	Chromobacterium
1	Bacteria	Proteobacteria	Betaproteobacteria	Neisseriales	Chromobacteriaceae	Aquitalea	Aquitalea pelogenes
1	Bacteria	Proteobacteria	Betaproteobacteria	Neisseriales	Chromobacteriaceae	Andreprevotia	Andreprevotia lacus
1	Bacteria	Proteobacteria	Betaproteobacteria	Methylophilales	unclassified Methylophilales	Methylophilales bacterium LSUCC0135	
1	Bacteria	Proteobacteria	Betaproteobacteria	Methylophilales	Methylophilaceae	unclassified Methylophilaceae	Methylophilaceae bacterium 11
1	Bacteria	Proteobacteria	Betaproteobacteria	Methylophilales	Methylophilaceae	Methylotenera	Methylotenera sp. 1P/1
1	Bacteria	Proteobacteria	Betaproteobacteria	Methylophilales	Methylophilaceae	Methylophilus	Methylophilus sp. 5
1	Bacteria	Proteobacteria	Betaproteobacteria	Methylophilales	Methylophilaceae	Methylophilus	Methylophilus rhizosphaerae
1	Bacteria	Proteobacteria	Betaproteobacteria	Methylophilales	Methylophilaceae	Candidatus Methylopumilus	Candidatus Methylopumilus turicensis
1	Bacteria	Proteobacteria	Betaproteobacteria	Hydrogenophilales	unclassified Hydrogenophilales	Hydrogenophilales bacterium RIFOXYA1_FULL_63_33	
1	Bacteria	Proteobacteria	Betaproteobacteria	Hydrogenophilales	Hydrogenophilaceae	unclassified Hydrogenophilaceae	Hydrogenophilaceae bacterium CG1_02_62_390
1	Bacteria	Proteobacteria	Betaproteobacteria	Hydrogenophilales	Hydrogenophilaceae	Thiobacillus	Thiobacillus sp. SCN 63-374
1	Bacteria	Proteobacteria	Betaproteobacteria	Hydrogenophilales	Hydrogenophilaceae	Tepidiphilus	Tepidiphilus margaritifer
1	Bacteria	Proteobacteria	Betaproteobacteria	Gallionellales	unclassified Gallionellales	Gallionellales bacterium GWA2_55_18	
1	Bacteria	Proteobacteria	Betaproteobacteria	Gallionellales	unclassified Gallionellales		
1	Bacteria	Proteobacteria	Betaproteobacteria	Gallionellales	Gallionellaceae	unclassified Gallionellaceae	Gallionellaceae bacterium CG1_02_56_997
1	Bacteria	Proteobacteria	Betaproteobacteria	Gallionellales	Gallionellaceae	Sideroxydans	Sideroxydans lithotrophicus
1	Bacteria	Proteobacteria	Betaproteobacteria	Gallionellales	Gallionellaceae	Sideroxydans	
1	Bacteria	Proteobacteria	Betaproteobacteria	Gallionellales	Gallionellaceae	Ferriphaselus	
1	Bacteria	Proteobacteria	Betaproteobacteria	Burkholderiales	unclassified Burkholderiales	unclassified Burkholderiales (miscellaneous)	Burkholderiales bacterium YL45
1	Bacteria	Proteobacteria	Betaproteobacteria	Burkholderiales	unclassified Burkholderiales	unclassified Burkholderiales (miscellaneous)	Burkholderiales bacterium RIFCSPLOWO2_02_FULL_66_35
1	Bacteria	Proteobacteria	Betaproteobacteria	Burkholderiales	unclassified Burkholderiales	unclassified Burkholderiales (miscellaneous)	Burkholderiales bacterium GWA2_64_37
1	Bacteria	Proteobacteria	Betaproteobacteria	Burkholderiales	unclassified Burkholderiales	Burkholderiales Genera incertae sedis	Xylophilus
1	Bacteria	Proteobacteria	Betaproteobacteria	Burkholderiales	unclassified Burkholderiales	Burkholderiales Genera incertae sedis	Thiomonas
1	Bacteria	Proteobacteria	Betaproteobacteria	Burkholderiales	unclassified Burkholderiales	Burkholderiales Genera incertae sedis	Rhizobacter
1	Bacteria	Proteobacteria	Betaproteobacteria	Burkholderiales	unclassified Burkholderiales	Burkholderiales Genera incertae sedis	Rhizobacter
1	Bacteria	Proteobacteria	Betaproteobacteria	Burkholderiales	unclassified Burkholderiales	Burkholderiales Genera incertae sedis	Rhizobacter
1	Bacteria	Proteobacteria	Betaproteobacteria	Burkholderiales	unclassified Burkholderiales	Burkholderiales Genera incertae sedis	Mitsuaria
1	Bacteria	Proteobacteria	Betaproteobacteria	Burkholderiales	unclassified Burkholderiales	Burkholderiales Genera incertae sedis	Mitsuaria
1	Bacteria	Proteobacteria	Betaproteobacteria	Burkholderiales	unclassified Burkholderiales	Burkholderiales Genera incertae sedis	Methylibium
1	Bacteria	Proteobacteria	Betaproteobacteria	Burkholderiales	unclassified Burkholderiales	Burkholderiales Genera incertae sedis	Methylibium
1	Bacteria	Proteobacteria	Betaproteobacteria	Burkholderiales	unclassified Burkholderiales	Burkholderiales Genera incertae sedis	Methylibium
1	Bacteria	Proteobacteria	Betaproteobacteria	Burkholderiales	unclassified Burkholderiales	Burkholderiales Genera incertae sedis	Leptothrix
1	Bacteria	Proteobacteria	Betaproteobacteria	Burkholderiales	Sutterellaceae	Sutterella	
1	Bacteria	Proteobacteria	Betaproteobacteria	Burkholderiales	Sutterellaceae	Parasutterella	Parasutterella excrementihominis
1	Bacteria	Proteobacteria	Betaproteobacteria	Burkholderiales	Sutterellaceae	Dakarella	Dakarella massiliensis
1	Bacteria	Proteobacteria	Betaproteobacteria	Burkholderiales	Oxalobacteraceae	unclassified Oxalobacteraceae	Oxalobacteraceae bacterium IMCC9480
1	Bacteria	Proteobacteria	Betaproteobacteria	Burkholderiales	Oxalobacteraceae	Pseudoduganella	Pseudoduganella violaceinigra
1	Bacteria	Proteobacteria	Betaproteobacteria	Burkholderiales	Oxalobacteraceae	Noviherbaspirillum	Noviherbaspirillum massiliense
1	Bacteria	Proteobacteria	Betaproteobacteria	Burkholderiales	Oxalobacteraceae	Noviherbaspirillum	
1	Bacteria	Proteobacteria	Betaproteobacteria	Burkholderiales	Oxalobacteraceae	Massilia	Massilia yuzhufengensis
1	Bacteria	Proteobacteria	Betaproteobacteria	Burkholderiales	Oxalobacteraceae	Massilia	Massilia timonae
1	Bacteria	Proteobacteria	Betaproteobacteria	Burkholderiales	Oxalobacteraceae	Massilia	Massilia sp. LC238
1	Bacteria	Proteobacteria	Betaproteobacteria	Burkholderiales	Oxalobacteraceae	Massilia	Massilia sp. BSC265
1	Bacteria	Proteobacteria	Betaproteobacteria	Burkholderiales	Oxalobacteraceae	Massilia	Massilia sp. 9096
1	Bacteria	Proteobacteria	Betaproteobacteria	Burkholderiales	Oxalobacteraceae	Janthinobacterium	Janthinobacterium sp. RA13
1	Bacteria	Proteobacteria	Betaproteobacteria	Burkholderiales	Oxalobacteraceae	Janthinobacterium	Janthinobacterium sp. CG3
1	Bacteria	Proteobacteria	Betaproteobacteria	Burkholderiales	Oxalobacteraceae	Janthinobacterium	
1	Bacteria	Proteobacteria	Betaproteobacteria	Burkholderiales	Oxalobacteraceae	Herminiimonas	Herminiimonas sp. CN
1	Bacteria	Proteobacteria	Betaproteobacteria	Burkholderiales	Oxalobacteraceae	Herminiimonas	
1	Bacteria	Proteobacteria	Betaproteobacteria	Burkholderiales	Oxalobacteraceae	Herbaspirillum	Herbaspirillum sp. RV1423
1	Bacteria	Proteobacteria	Betaproteobacteria	Burkholderiales	Oxalobacteraceae	Herbaspirillum	Herbaspirillum rhizosphaerae
1	Bacteria	Proteobacteria	Betaproteobacteria	Burkholderiales	Oxalobacteraceae	Herbaspirillum	Herbaspirillum lusitanum
1	Bacteria	Proteobacteria	Betaproteobacteria	Burkholderiales	Oxalobacteraceae	Duganella	Duganella sacchari
1	Bacteria	Proteobacteria	Betaproteobacteria	Burkholderiales	Oxalobacteraceae	Collimonas	Collimonas pratensis
1	Bacteria	Proteobacteria	Betaproteobacteria	Burkholderiales	Comamonadaceae	unclassified Comamonadaceae	Comamonadaceae bacterium BICA1-1
1	Bacteria	Proteobacteria	Betaproteobacteria	Burkholderiales	Comamonadaceae	unclassified Comamonadaceae	Comamonadaceae bacterium A1
1	Bacteria	Proteobacteria	Betaproteobacteria	Burkholderiales	Comamonadaceae	Variovorax	Variovorax sp. YR216
1	Bacteria	Proteobacteria	Betaproteobacteria	Burkholderiales	Comamonadaceae	Variovorax	Variovorax sp. Root473
1	Bacteria	Proteobacteria	Betaproteobacteria	Burkholderiales	Comamonadaceae	Variovorax	Variovorax sp. KK3
1	Bacteria	Proteobacteria	Betaproteobacteria	Burkholderiales	Comamonadaceae	Variovorax	Variovorax soli
1	Bacteria	Proteobacteria	Betaproteobacteria	Burkholderiales	Comamonadaceae	Variovorax	Variovorax boronicumulans
1	Bacteria	Proteobacteria	Betaproteobacteria	Burkholderiales	Comamonadaceae	Simplicispira	Simplicispira psychrophila
1	Bacteria	Proteobacteria	Betaproteobacteria	Burkholderiales	Comamonadaceae	Rhodoferax	Rhodoferax sp. DCY110
1	Bacteria	Proteobacteria	Betaproteobacteria	Burkholderiales	Comamonadaceae	Rhodoferax	Rhodoferax ferrireducens
1	Bacteria	Proteobacteria	Betaproteobacteria	Burkholderiales	Comamonadaceae	Rhodoferax	Rhodoferax fermentans
1	Bacteria	Proteobacteria	Betaproteobacteria	Burkholderiales	Comamonadaceae	Ramlibacter	Ramlibacter sp. Leaf400
1	Bacteria	Proteobacteria	Betaproteobacteria	Burkholderiales	Comamonadaceae	Pseudorhodoferax	Pseudorhodoferax sp. Leaf265
1	Bacteria	Proteobacteria	Betaproteobacteria	Burkholderiales	Comamonadaceae	Pseudorhodoferax	
1	Bacteria	Proteobacteria	Betaproteobacteria	Burkholderiales	Comamonadaceae	Pseudacidovorax	Pseudacidovorax sp. RU35E
1	Bacteria	Proteobacteria	Betaproteobacteria	Burkholderiales	Comamonadaceae	Pseudacidovorax	Pseudacidovorax intermedius
1	Bacteria	Proteobacteria	Betaproteobacteria	Burkholderiales	Comamonadaceae	Polaromonas	Polaromonas sp. YR568
1	Bacteria	Proteobacteria	Betaproteobacteria	Burkholderiales	Comamonadaceae	Polaromonas	Polaromonas sp. OV174
1	Bacteria	Proteobacteria	Betaproteobacteria	Burkholderiales	Comamonadaceae	Polaromonas	Polaromonas sp. EUR3 1.2.1
1	Bacteria	Proteobacteria	Betaproteobacteria	Burkholderiales	Comamonadaceae	Polaromonas	Polaromonas jejuensis
1	Bacteria	Proteobacteria	Betaproteobacteria	Burkholderiales	Comamonadaceae	Pelomonas	Pelomonas sp. KK5
1	Bacteria	Proteobacteria	Betaproteobacteria	Burkholderiales	Comamonadaceae	Ottowia	Ottowia thiooxydans
1	Bacteria	Proteobacteria	Betaproteobacteria	Burkholderiales	Comamonadaceae	Limnohabitans	Limnohabitans sp. Rim28
1	Bacteria	Proteobacteria	Betaproteobacteria	Burkholderiales	Comamonadaceae	Limnohabitans	
1	Bacteria	Proteobacteria	Betaproteobacteria	Burkholderiales	Comamonadaceae	Hydrogenophaga	Hydrogenophaga taeniospiralis
1	Bacteria	Proteobacteria	Betaproteobacteria	Burkholderiales	Comamonadaceae	Hydrogenophaga	Hydrogenophaga sp. IBVHS2
1	Bacteria	Proteobacteria	Betaproteobacteria	Burkholderiales	Comamonadaceae	Hydrogenophaga	Hydrogenophaga pseudoflava
1	Bacteria	Proteobacteria	Betaproteobacteria	Burkholderiales	Comamonadaceae	Diaphorobacter	Diaphorobacter sp. J5-51
1	Bacteria	Proteobacteria	Betaproteobacteria	Burkholderiales	Comamonadaceae	Delftia	Delftia sp. RIT313
1	Bacteria	Proteobacteria	Betaproteobacteria	Burkholderiales	Comamonadaceae	Curvibacter	Curvibacter sp. PAE-UM
1	Bacteria	Proteobacteria	Betaproteobacteria	Burkholderiales	Comamonadaceae	Curvibacter	
1	Bacteria	Proteobacteria	Betaproteobacteria	Burkholderiales	Comamonadaceae	Comamonas	Comamonas kerstersii
1	Bacteria	Proteobacteria	Betaproteobacteria	Burkholderiales	Comamonadaceae	Caldimonas	
1	Bacteria	Proteobacteria	Betaproteobacteria	Burkholderiales	Comamonadaceae	Acidovorax	Acidovorax sp. SCN 65-28
1	Bacteria	Proteobacteria	Betaproteobacteria	Burkholderiales	Comamonadaceae	Acidovorax	Acidovorax sp. Root70
1	Bacteria	Proteobacteria	Betaproteobacteria	Burkholderiales	Comamonadaceae	Acidovorax	Acidovorax sp. GW101-3H11
1	Bacteria	Proteobacteria	Betaproteobacteria	Burkholderiales	Comamonadaceae	Acidovorax	Acidovorax sp. CF316
1	Bacteria	Proteobacteria	Betaproteobacteria	Burkholderiales	Comamonadaceae	Acidovorax	Acidovorax sp. CCH12-A4
1	Bacteria	Proteobacteria	Betaproteobacteria	Burkholderiales	Comamonadaceae	Acidovorax	Acidovorax oryzae
1	Bacteria	Proteobacteria	Betaproteobacteria	Burkholderiales	Comamonadaceae	Acidovorax	Acidovorax konjaci
1	Bacteria	Proteobacteria	Betaproteobacteria	Burkholderiales	Comamonadaceae	Acidovorax	Acidovorax citrulli
1	Bacteria	Proteobacteria	Betaproteobacteria	Burkholderiales	Burkholderiaceae	Ralstonia	Ralstonia sp. PBA
1	Bacteria	Proteobacteria	Betaproteobacteria	Burkholderiales	Burkholderiaceae	Ralstonia	Ralstonia sp. A12
1	Bacteria	Proteobacteria	Betaproteobacteria	Burkholderiales	Burkholderiaceae	Ralstonia	Ralstonia sp. 5_7_47FAA
1	Bacteria	Proteobacteria	Betaproteobacteria	Burkholderiales	Burkholderiaceae	Ralstonia	Ralstonia solanacearum
1	Bacteria	Proteobacteria	Betaproteobacteria	Burkholderiales	Burkholderiaceae	Ralstonia	Ralstonia solanacearum
1	Bacteria	Proteobacteria	Betaproteobacteria	Burkholderiales	Burkholderiaceae	Ralstonia	Ralstonia pickettii
1	Bacteria	Proteobacteria	Betaproteobacteria	Burkholderiales	Burkholderiaceae	Ralstonia	Ralstonia pickettii
1	Bacteria	Proteobacteria	Betaproteobacteria	Burkholderiales	Burkholderiaceae	Polynucleobacter	Polynucleobacter sp. VK13
1	Bacteria	Proteobacteria	Betaproteobacteria	Burkholderiales	Burkholderiaceae	Polynucleobacter	Polynucleobacter sp. GWA2_45_21
1	Bacteria	Proteobacteria	Betaproteobacteria	Burkholderiales	Burkholderiaceae	Polynucleobacter	Polynucleobacter sinensis
1	Bacteria	Proteobacteria	Betaproteobacteria	Burkholderiales	Burkholderiaceae	Paraburkholderia	Paraburkholderia tuberum
1	Bacteria	Proteobacteria	Betaproteobacteria	Burkholderiales	Burkholderiaceae	Paraburkholderia	Paraburkholderia susongensis
1	Bacteria	Proteobacteria	Betaproteobacteria	Burkholderiales	Burkholderiaceae	Paraburkholderia	Paraburkholderia sacchari
1	Bacteria	Proteobacteria	Betaproteobacteria	Burkholderiales	Burkholderiaceae	Paraburkholderia	Paraburkholderia ribeironis
1	Bacteria	Proteobacteria	Betaproteobacteria	Burkholderiales	Burkholderiaceae	Paraburkholderia	Paraburkholderia rhizoxinica
1	Bacteria	Proteobacteria	Betaproteobacteria	Burkholderiales	Burkholderiaceae	Paraburkholderia	Paraburkholderia nodosa
1	Bacteria	Proteobacteria	Betaproteobacteria	Burkholderiales	Burkholderiaceae	Paraburkholderia	Paraburkholderia mimosarum
1	Bacteria	Proteobacteria	Betaproteobacteria	Burkholderiales	Burkholderiaceae	Paraburkholderia	Paraburkholderia ferrariae
1	Bacteria	Proteobacteria	Betaproteobacteria	Burkholderiales	Burkholderiaceae	Paraburkholderia	Paraburkholderia aspalathi
1	Bacteria	Proteobacteria	Betaproteobacteria	Burkholderiales	Burkholderiaceae	Pandoraea	Pandoraea sputorum
1	Bacteria	Proteobacteria	Betaproteobacteria	Burkholderiales	Burkholderiaceae	Limnobacter	Limnobacter sp. MED105
1	Bacteria	Proteobacteria	Betaproteobacteria	Burkholderiales	Burkholderiaceae	Limnobacter	Limnobacter sp. CACIAM 66H1
1	Bacteria	Proteobacteria	Betaproteobacteria	Burkholderiales	Burkholderiaceae	Limnobacter	
1	Bacteria	Proteobacteria	Betaproteobacteria	Burkholderiales	Burkholderiaceae	Cupriavidus	Cupriavidus taiwanensis
1	Bacteria	Proteobacteria	Betaproteobacteria	Burkholderiales	Burkholderiaceae	Cupriavidus	Cupriavidus sp. UYMMa02A
1	Bacteria	Proteobacteria	Betaproteobacteria	Burkholderiales	Burkholderiaceae	Cupriavidus	Cupriavidus sp. IDO
1	Bacteria	Proteobacteria	Betaproteobacteria	Burkholderiales	Burkholderiaceae	Chitinimonas	Chitinimonas taiwanensis
1	Bacteria	Proteobacteria	Betaproteobacteria	Burkholderiales	Burkholderiaceae	Chitinimonas	Chitinimonas koreensis
1	Bacteria	Proteobacteria	Betaproteobacteria	Burkholderiales	Burkholderiaceae	Candidatus Glomeribacter	Candidatus Glomeribacter gigasporarum
1	Bacteria	Proteobacteria	Betaproteobacteria	Burkholderiales	Burkholderiaceae	Caballeronia	
1	Bacteria	Proteobacteria	Betaproteobacteria	Burkholderiales	Burkholderiaceae	Burkholderia	pseudomallei group
1	Bacteria	Proteobacteria	Betaproteobacteria	Burkholderiales	Burkholderiaceae	Burkholderia	pseudomallei group
1	Bacteria	Proteobacteria	Betaproteobacteria	Burkholderiales	Burkholderiaceae	Burkholderia	Burkholderia sp. RPE64
1	Bacteria	Proteobacteria	Betaproteobacteria	Burkholderiales	Burkholderiaceae	Burkholderia	Burkholderia sp. JS23
1	Bacteria	Proteobacteria	Betaproteobacteria	Burkholderiales	Burkholderiaceae	Burkholderia	Burkholderia sp. H160
1	Bacteria	Proteobacteria	Betaproteobacteria	Burkholderiales	Burkholderiaceae	Burkholderia	Burkholderia glebae
1	Bacteria	Proteobacteria	Betaproteobacteria	Burkholderiales	Burkholderiaceae	Burkholderia	Burkholderia gladioli
1	Bacteria	Proteobacteria	Betaproteobacteria	Burkholderiales	Burkholderiaceae	Burkholderia	Burkholderia cepacia complex
1	Bacteria	Proteobacteria	Betaproteobacteria	Burkholderiales	Burkholderiaceae	Burkholderia	Burkholderia cepacia complex
1	Bacteria	Proteobacteria	Betaproteobacteria	Burkholderiales	Burkholderiaceae	Burkholderia	Burkholderia cepacia complex
1	Bacteria	Proteobacteria	Betaproteobacteria	Burkholderiales	Burkholderiaceae	Burkholderia	Burkholderia cepacia complex
1	Bacteria	Proteobacteria	Betaproteobacteria	Burkholderiales	Burkholderiaceae	Burkholderia	Burkholderia cepacia complex
1	Bacteria	Proteobacteria	Betaproteobacteria	Burkholderiales	Alcaligenaceae	Taylorella	Taylorella asinigenitalis
1	Bacteria	Proteobacteria	Betaproteobacteria	Burkholderiales	Alcaligenaceae	Taylorella	Taylorella asinigenitalis
1	Bacteria	Proteobacteria	Betaproteobacteria	Burkholderiales	Alcaligenaceae	Pusillimonas	Pusillimonas sp. T7-7
1	Bacteria	Proteobacteria	Betaproteobacteria	Burkholderiales	Alcaligenaceae	Pusillimonas	Pusillimonas noertemannii
1	Bacteria	Proteobacteria	Betaproteobacteria	Burkholderiales	Alcaligenaceae	Oligella	
1	Bacteria	Proteobacteria	Betaproteobacteria	Burkholderiales	Alcaligenaceae	Castellaniella	Castellaniella caeni
1	Bacteria	Proteobacteria	Betaproteobacteria	Burkholderiales	Alcaligenaceae	Castellaniella	
1	Bacteria	Proteobacteria	Betaproteobacteria	Burkholderiales	Alcaligenaceae	Bordetella	Bordetella sp. SCN 68-11
1	Bacteria	Proteobacteria	Betaproteobacteria	Burkholderiales	Alcaligenaceae	Bordetella	Bordetella sp. N
1	Bacteria	Proteobacteria	Betaproteobacteria	Burkholderiales	Alcaligenaceae	Bordetella	Bordetella genomosp. 13
1	Bacteria	Proteobacteria	Betaproteobacteria	Burkholderiales	Alcaligenaceae	Bordetella	Bordetella flabilis
1	Bacteria	Proteobacteria	Betaproteobacteria	Burkholderiales	Alcaligenaceae	Bordetella	Bordetella bronchiseptica
1	Bacteria	Proteobacteria	Betaproteobacteria	Burkholderiales	Alcaligenaceae	Bordetella	Bordetella avium
1	Bacteria	Proteobacteria	Betaproteobacteria	Burkholderiales	Alcaligenaceae	Basilea	Basilea psittacipulmonis
1	Bacteria	Proteobacteria	Betaproteobacteria	Burkholderiales	Alcaligenaceae	Alcaligenes	Alcaligenes faecalis
1	Bacteria	Proteobacteria	Betaproteobacteria	Burkholderiales	Alcaligenaceae	Advenella	Advenella mimigardefordensis
1	Bacteria	Proteobacteria	Betaproteobacteria	Burkholderiales	Alcaligenaceae	Achromobacter	Achromobacter sp. MFA1 R4
1	Bacteria	Proteobacteria	Betaproteobacteria	Burkholderiales	Alcaligenaceae	Achromobacter	Achromobacter sp. HMSC056C09
1	Bacteria	Proteobacteria	Betaproteobacteria	Burkholderiales	Alcaligenaceae	Achromobacter	Achromobacter sp. ATCC31444
1	Bacteria	Proteobacteria	Betaproteobacteria	Burkholderiales	Alcaligenaceae	Achromobacter	Achromobacter sp. 2789STDY5608625
1	Bacteria	Proteobacteria	Betaproteobacteria	Burkholderiales	Alcaligenaceae	Achromobacter	Achromobacter piechaudii
1	Bacteria	Proteobacteria	Betaproteobacteria	Burkholderiales	Alcaligenaceae	Achromobacter	Achromobacter piechaudii
1	Bacteria	Proteobacteria	Alphaproteobacteria	unclassified Alphaproteobacteria	unclassified Alphaproteobacteria (miscellaneous)	alpha proteobacterium Q-1	
1	Bacteria	Proteobacteria	Alphaproteobacteria	unclassified Alphaproteobacteria	unclassified Alphaproteobacteria (miscellaneous)	Alphaproteobacteria bacterium 62-8	
1	Bacteria	Proteobacteria	Alphaproteobacteria	unclassified Alphaproteobacteria	unclassified Alphaproteobacteria (miscellaneous)	Alphaproteobacteria bacterium 33-17	
1	Bacteria	Proteobacteria	Alphaproteobacteria	unclassified Alphaproteobacteria	unclassified Alphaproteobacteria (miscellaneous)	Alphaproteobacteria bacterium 13_2_20CM_2_64_7	
1	Bacteria	Proteobacteria	Alphaproteobacteria	Sphingomonadales	Sphingomonadaceae	Sphingorhabdus	Sphingorhabdus sp. M41
1	Bacteria	Proteobacteria	Alphaproteobacteria	Sphingomonadales	Sphingomonadaceae	Sphingopyxis	Sphingopyxis terrae
1	Bacteria	Proteobacteria	Alphaproteobacteria	Sphingomonadales	Sphingomonadaceae	Sphingopyxis	Sphingopyxis sp. SCN 67-31
1	Bacteria	Proteobacteria	Alphaproteobacteria	Sphingomonadales	Sphingomonadaceae	Sphingopyxis	Sphingopyxis sp. RIFCSPHIGHO2_01_FULL_65_24
1	Bacteria	Proteobacteria	Alphaproteobacteria	Sphingomonadales	Sphingomonadaceae	Sphingopyxis	Sphingopyxis sp. QXT-31
1	Bacteria	Proteobacteria	Alphaproteobacteria	Sphingomonadales	Sphingomonadaceae	Sphingopyxis	Sphingopyxis sp. LC363
1	Bacteria	Proteobacteria	Alphaproteobacteria	Sphingomonadales	Sphingomonadaceae	Sphingopyxis	Sphingopyxis sp. KK2
1	Bacteria	Proteobacteria	Alphaproteobacteria	Sphingomonadales	Sphingomonadaceae	Sphingopyxis	Sphingopyxis sp. H115
1	Bacteria	Proteobacteria	Alphaproteobacteria	Sphingomonadales	Sphingomonadaceae	Sphingopyxis	Sphingopyxis sp. 113P3
1	Bacteria	Proteobacteria	Alphaproteobacteria	Sphingomonadales	Sphingomonadaceae	Sphingopyxis	Sphingopyxis macrogoltabida
1	Bacteria	Proteobacteria	Alphaproteobacteria	Sphingomonadales	Sphingomonadaceae	Sphingopyxis	Sphingopyxis granuli
1	Bacteria	Proteobacteria	Alphaproteobacteria	Sphingomonadales	Sphingomonadaceae	Sphingopyxis	Sphingopyxis fribergensis
1	Bacteria	Proteobacteria	Alphaproteobacteria	Sphingomonadales	Sphingomonadaceae	Sphingopyxis	Sphingopyxis flava
1	Bacteria	Proteobacteria	Alphaproteobacteria	Sphingomonadales	Sphingomonadaceae	Sphingomonas	Sphingomonas wittichii
1	Bacteria	Proteobacteria	Alphaproteobacteria	Sphingomonadales	Sphingomonadaceae	Sphingomonas	Sphingomonas sp. YR710
1	Bacteria	Proteobacteria	Alphaproteobacteria	Sphingomonadales	Sphingomonadaceae	Sphingomonas	Sphingomonas sp. W16RD
1	Bacteria	Proteobacteria	Alphaproteobacteria	Sphingomonadales	Sphingomonadaceae	Sphingomonas	Sphingomonas sp. SKA58
1	Bacteria	Proteobacteria	Alphaproteobacteria	Sphingomonadales	Sphingomonadaceae	Sphingomonas	Sphingomonas sp. SCN 67-18
1	Bacteria	Proteobacteria	Alphaproteobacteria	Sphingomonadales	Sphingomonadaceae	Sphingomonas	Sphingomonas sp. S6-262
1	Bacteria	Proteobacteria	Alphaproteobacteria	Sphingomonadales	Sphingomonadaceae	Sphingomonas	Sphingomonas sp. S5-249
1	Bacteria	Proteobacteria	Alphaproteobacteria	Sphingomonadales	Sphingomonadaceae	Sphingomonas	Sphingomonas sp. Root710
1	Bacteria	Proteobacteria	Alphaproteobacteria	Sphingomonadales	Sphingomonadaceae	Sphingomonas	Sphingomonas sp. PR090111-T3T-6A
1	Bacteria	Proteobacteria	Alphaproteobacteria	Sphingomonadales	Sphingomonadaceae	Sphingomonas	Sphingomonas sp. PAMC 26617
1	Bacteria	Proteobacteria	Alphaproteobacteria	Sphingomonadales	Sphingomonadaceae	Sphingomonas	Sphingomonas sp. PAMC 26605
1	Bacteria	Proteobacteria	Alphaproteobacteria	Sphingomonadales	Sphingomonadaceae	Sphingomonas	Sphingomonas sp. Ndbn-20
1	Bacteria	Proteobacteria	Alphaproteobacteria	Sphingomonadales	Sphingomonadaceae	Sphingomonas	Sphingomonas sp. NIC1
1	Bacteria	Proteobacteria	Alphaproteobacteria	Sphingomonadales	Sphingomonadaceae	Sphingomonas	Sphingomonas sp. NFR04
1	Bacteria	Proteobacteria	Alphaproteobacteria	Sphingomonadales	Sphingomonadaceae	Sphingomonas	Sphingomonas sp. MEA3-1
1	Bacteria	Proteobacteria	Alphaproteobacteria	Sphingomonadales	Sphingomonadaceae	Sphingomonas	Sphingomonas sp. Leaf412
1	Bacteria	Proteobacteria	Alphaproteobacteria	Sphingomonadales	Sphingomonadaceae	Sphingomonas	Sphingomonas sp. Leaf357
1	Bacteria	Proteobacteria	Alphaproteobacteria	Sphingomonadales	Sphingomonadaceae	Sphingomonas	Sphingomonas sp. Leaf33
1	Bacteria	Proteobacteria	Alphaproteobacteria	Sphingomonadales	Sphingomonadaceae	Sphingomonas	Sphingomonas sp. Leaf198
1	Bacteria	Proteobacteria	Alphaproteobacteria	Sphingomonadales	Sphingomonadaceae	Sphingomonas	Sphingomonas sp. JS21-1
1	Bacteria	Proteobacteria	Alphaproteobacteria	Sphingomonadales	Sphingomonadaceae	Sphingomonas	Sphingomonas sp. JJ-A5
1	Bacteria	Proteobacteria	Alphaproteobacteria	Sphingomonadales	Sphingomonadaceae	Sphingomonas	Sphingomonas sp. ERG5
1	Bacteria	Proteobacteria	Alphaproteobacteria	Sphingomonadales	Sphingomonadaceae	Sphingomonas	Sphingomonas sp. CCH9-E2
1	Bacteria	Proteobacteria	Alphaproteobacteria	Sphingomonadales	Sphingomonadaceae	Sphingomonas	Sphingomonas sp. CCH10-B3
1	Bacteria	Proteobacteria	Alphaproteobacteria	Sphingomonadales	Sphingomonadaceae	Sphingomonas	Sphingomonas sp. BHC-A
1	Bacteria	Proteobacteria	Alphaproteobacteria	Sphingomonadales	Sphingomonadaceae	Sphingomonas	Sphingomonas sp. 67-36
1	Bacteria	Proteobacteria	Alphaproteobacteria	Sphingomonadales	Sphingomonadaceae	Sphingomonas	Sphingomonas sp. 37zxx
1	Bacteria	Proteobacteria	Alphaproteobacteria	Sphingomonadales	Sphingomonadaceae	Sphingomonas	Sphingomonas sanxanigenens
1	Bacteria	Proteobacteria	Alphaproteobacteria	Sphingomonadales	Sphingomonadaceae	Sphingomonas	Sphingomonas pruni
1	Bacteria	Proteobacteria	Alphaproteobacteria	Sphingomonadales	Sphingomonadaceae	Sphingomonas	Sphingomonas panacis
1	Bacteria	Proteobacteria	Alphaproteobacteria	Sphingomonadales	Sphingomonadaceae	Sphingomonas	Sphingomonas jaspsi
1	Bacteria	Proteobacteria	Alphaproteobacteria	Sphingomonadales	Sphingomonadaceae	Sphingomonas	Sphingomonas hengshuiensis
1	Bacteria	Proteobacteria	Alphaproteobacteria	Sphingomonadales	Sphingomonadaceae	Sphingomonas	Sphingomonas haloaromaticamans
1	Bacteria	Proteobacteria	Alphaproteobacteria	Sphingomonadales	Sphingomonadaceae	Sphingomonas	Sphingomonas endophytica
1	Bacteria	Proteobacteria	Alphaproteobacteria	Sphingomonadales	Sphingomonadaceae	Sphingomonas	Sphingomonas azotifigens
1	Bacteria	Proteobacteria	Alphaproteobacteria	Sphingomonadales	Sphingomonadaceae	Sphingomonas	Sphingomonas asaccharolytica
1	Bacteria	Proteobacteria	Alphaproteobacteria	Sphingomonadales	Sphingomonadaceae	Sphingobium	Sphingobium sp. C100
1	Bacteria	Proteobacteria	Alphaproteobacteria	Sphingomonadales	Sphingomonadaceae	Sphingobium	Sphingobium sp. Ant17
1	Bacteria	Proteobacteria	Alphaproteobacteria	Sphingomonadales	Sphingomonadaceae	Sphingobium	Sphingobium sp. AP49
1	Bacteria	Proteobacteria	Alphaproteobacteria	Sphingomonadales	Sphingomonadaceae	Sphingobium	Sphingobium quisquiliarum
1	Bacteria	Proteobacteria	Alphaproteobacteria	Sphingomonadales	Sphingomonadaceae	Sphingobium	Sphingobium faniae
1	Bacteria	Proteobacteria	Alphaproteobacteria	Sphingomonadales	Sphingomonadaceae	Sphingobium	Sphingobium chlorophenolicum
1	Bacteria	Proteobacteria	Alphaproteobacteria	Sphingomonadales	Sphingomonadaceae	Novosphingobium	Novosphingobium sp. P6W
1	Bacteria	Proteobacteria	Alphaproteobacteria	Sphingomonadales	Sphingomonadaceae	Novosphingobium	Novosphingobium sp. NDB2Meth1
1	Bacteria	Proteobacteria	Alphaproteobacteria	Sphingomonadales	Sphingomonadaceae	Novosphingobium	Novosphingobium sp. MBES04
1	Bacteria	Proteobacteria	Alphaproteobacteria	Sphingomonadales	Sphingomonadaceae	Novosphingobium	Novosphingobium sp. KN65.2
1	Bacteria	Proteobacteria	Alphaproteobacteria	Sphingomonadales	Sphingomonadaceae	Novosphingobium	Novosphingobium sp. AAP93
1	Bacteria	Proteobacteria	Alphaproteobacteria	Sphingomonadales	Sphingomonadaceae	Novosphingobium	Novosphingobium sp. AAP1
1	Bacteria	Proteobacteria	Alphaproteobacteria	Sphingomonadales	Sphingomonadaceae	Novosphingobium	Novosphingobium sp. 63-713
1	Bacteria	Proteobacteria	Alphaproteobacteria	Sphingomonadales	Sphingomonadaceae	Novosphingobium	Novosphingobium rosa
1	Bacteria	Proteobacteria	Alphaproteobacteria	Sphingomonadales	Sphingomonadaceae	Novosphingobium	Novosphingobium resinovorum
1	Bacteria	Proteobacteria	Alphaproteobacteria	Sphingomonadales	Sphingomonadaceae	Novosphingobium	Novosphingobium lindaniclasticum
1	Bacteria	Proteobacteria	Alphaproteobacteria	Sphingomonadales	Sphingomonadaceae	Citromicrobium	Citromicrobium sp. JLT1363
1	Bacteria	Proteobacteria	Alphaproteobacteria	Sphingomonadales	Sphingomonadaceae	Blastomonas	Blastomonas sp. CACIA14H2
1	Bacteria	Proteobacteria	Alphaproteobacteria	Sphingomonadales	Sphingomonadaceae	Blastomonas	Blastomonas sp. AAP53
1	Bacteria	Proteobacteria	Alphaproteobacteria	Sphingomonadales	Erythrobacteraceae	Porphyrobacter	Porphyrobacter sp. AAP82
1	Bacteria	Proteobacteria	Alphaproteobacteria	Sphingomonadales	Erythrobacteraceae	Porphyrobacter	Porphyrobacter sp. AAP60
1	Bacteria	Proteobacteria	Alphaproteobacteria	Sphingomonadales	Erythrobacteraceae	Porphyrobacter	Porphyrobacter sanguineus
1	Bacteria	Proteobacteria	Alphaproteobacteria	Sphingomonadales	Erythrobacteraceae	Porphyrobacter	Porphyrobacter mercurialis
1	Bacteria	Proteobacteria	Alphaproteobacteria	Sphingomonadales	Erythrobacteraceae	Erythrobacter	Erythrobacter vulgaris
1	Bacteria	Proteobacteria	Alphaproteobacteria	Sphingomonadales	Erythrobacteraceae	Erythrobacter	Erythrobacter sp. SG61-1L
1	Bacteria	Proteobacteria	Alphaproteobacteria	Sphingomonadales	Erythrobacteraceae	Erythrobacter	Erythrobacter sp. QSSC1-22B
1	Bacteria	Proteobacteria	Alphaproteobacteria	Sphingomonadales	Erythrobacteraceae	Erythrobacter	Erythrobacter sp. NAP1
1	Bacteria	Proteobacteria	Alphaproteobacteria	Sphingomonadales	Erythrobacteraceae	Erythrobacter	Erythrobacter sp. HL-111
1	Bacteria	Proteobacteria	Alphaproteobacteria	Sphingomonadales	Erythrobacteraceae	Erythrobacter	Erythrobacter sp. AP23
1	Bacteria	Proteobacteria	Alphaproteobacteria	Sphingomonadales	Erythrobacteraceae	Erythrobacter	Erythrobacter longus
1	Bacteria	Proteobacteria	Alphaproteobacteria	Sphingomonadales	Erythrobacteraceae	Altererythrobacter	Altererythrobacter sp. MH-B5
1	Bacteria	Proteobacteria	Alphaproteobacteria	Sphingomonadales	Erythrobacteraceae	Altererythrobacter	Altererythrobacter marensis
1	Bacteria	Proteobacteria	Alphaproteobacteria	Sneathiellales	Sneathiellaceae	Oceanibacterium	Oceanibacterium hippocampi
1	Bacteria	Proteobacteria	Alphaproteobacteria	Rickettsiales	Rickettsiaceae	Rickettsieae	Orientia
1	Bacteria	Proteobacteria	Alphaproteobacteria	Rickettsiales	Rickettsiaceae	Rickettsieae	Occidentia
1	Bacteria	Proteobacteria	Alphaproteobacteria	Rickettsiales	Candidatus Midichloriaceae	Candidatus Midichloria	Candidatus Midichloria mitochondrii
1	Bacteria	Proteobacteria	Alphaproteobacteria	Rickettsiales	Anaplasmataceae	Ehrlichia	canis group
1	Bacteria	Proteobacteria	Alphaproteobacteria	Rhodospirillales	unclassified Rhodospirillales	Reyranella	Reyranella massiliensis
1	Bacteria	Proteobacteria	Alphaproteobacteria	Rhodospirillales	unclassified Rhodospirillales		
1	Bacteria	Proteobacteria	Alphaproteobacteria	Rhodospirillales	Rhodospirillaceae	unclassified Rhodospirillaceae	Rhodospirillaceae bacterium CCH5-H10
1	Bacteria	Proteobacteria	Alphaproteobacteria	Rhodospirillales	Rhodospirillaceae	Thalassospira	Thalassospira xiamenensis
1	Bacteria	Proteobacteria	Alphaproteobacteria	Rhodospirillales	Rhodospirillaceae	Thalassobaculum	
1	Bacteria	Proteobacteria	Alphaproteobacteria	Rhodospirillales	Rhodospirillaceae	Rhodospirillum	Rhodospirillum centenum
1	Bacteria	Proteobacteria	Alphaproteobacteria	Rhodospirillales	Rhodospirillaceae	Phaeospirillum	
1	Bacteria	Proteobacteria	Alphaproteobacteria	Rhodospirillales	Rhodospirillaceae	Novispirillum	Novispirillum itersonii
1	Bacteria	Proteobacteria	Alphaproteobacteria	Rhodospirillales	Rhodospirillaceae	Magnetospirillum	Magnetospirillum sp. ME-1
1	Bacteria	Proteobacteria	Alphaproteobacteria	Rhodospirillales	Rhodospirillaceae	Magnetospirillum	Magnetospirillum marisnigri
1	Bacteria	Proteobacteria	Alphaproteobacteria	Rhodospirillales	Rhodospirillaceae	Magnetospirillum	Magnetospirillum magnetotacticum
1	Bacteria	Proteobacteria	Alphaproteobacteria	Rhodospirillales	Rhodospirillaceae	Limimonas	Limimonas halophila
1	Bacteria	Proteobacteria	Alphaproteobacteria	Rhodospirillales	Rhodospirillaceae	Insolitispirillum	Insolitispirillum peregrinum
1	Bacteria	Proteobacteria	Alphaproteobacteria	Rhodospirillales	Rhodospirillaceae	Haematospirillum	Haematospirillum jordaniae
1	Bacteria	Proteobacteria	Alphaproteobacteria	Rhodospirillales	Rhodospirillaceae	Fodinicurvata	
1	Bacteria	Proteobacteria	Alphaproteobacteria	Rhodospirillales	Rhodospirillaceae	Elstera	Elstera litoralis
1	Bacteria	Proteobacteria	Alphaproteobacteria	Rhodospirillales	Rhodospirillaceae	Azospirillum	environmental samples
1	Bacteria	Proteobacteria	Alphaproteobacteria	Rhodospirillales	Rhodospirillaceae	Azospirillum	Azospirillum brasilense
1	Bacteria	Proteobacteria	Alphaproteobacteria	Rhodospirillales	Rhodospirillaceae		
1	Bacteria	Proteobacteria	Alphaproteobacteria	Rhodospirillales	Acetobacteraceae	Roseomonas	Roseomonas stagni
1	Bacteria	Proteobacteria	Alphaproteobacteria	Rhodospirillales	Acetobacteraceae	Roseomonas	Roseomonas rosea
1	Bacteria	Proteobacteria	Alphaproteobacteria	Rhodospirillales	Acetobacteraceae	Roseomonas	Roseomonas cervicalis
1	Bacteria	Proteobacteria	Alphaproteobacteria	Rhodospirillales	Acetobacteraceae	Roseomonas	Roseomonas aerilata
1	Bacteria	Proteobacteria	Alphaproteobacteria	Rhodospirillales	Acetobacteraceae	Komagataeibacter	Komagataeibacter hansenii
1	Bacteria	Proteobacteria	Alphaproteobacteria	Rhodospirillales	Acetobacteraceae	Gluconobacter	Gluconobacter oxydans
1	Bacteria	Proteobacteria	Alphaproteobacteria	Rhodospirillales	Acetobacteraceae	Commensalibacter	Commensalibacter intestini
1	Bacteria	Proteobacteria	Alphaproteobacteria	Rhodospirillales	Acetobacteraceae	Asaia	
1	Bacteria	Proteobacteria	Alphaproteobacteria	Rhodospirillales	Acetobacteraceae	Acidocella	Acidocella aminolytica
1	Bacteria	Proteobacteria	Alphaproteobacteria	Rhodospirillales	Acetobacteraceae	Acidiphilium	
1	Bacteria	Proteobacteria	Alphaproteobacteria	Rhodospirillales	Acetobacteraceae	Acetobacter	Acetobacter syzygii
1	Bacteria	Proteobacteria	Alphaproteobacteria	Rhodospirillales	Acetobacteraceae	Acetobacter	Acetobacter pasteurianus
1	Bacteria	Proteobacteria	Alphaproteobacteria	Rhodospirillales	Acetobacteraceae	Acetobacter	Acetobacter indonesiensis
1	Bacteria	Proteobacteria	Alphaproteobacteria	Rhodospirillales	Acetobacteraceae	Acetobacter	
1	Bacteria	Proteobacteria	Alphaproteobacteria	Rhodospirillales	Acetobacteraceae		
1	Bacteria	Proteobacteria	Alphaproteobacteria	Rhodobacterales	unclassified Rhodobacterales	Rhodobacterales bacterium CG2_30_65_12	
1	Bacteria	Proteobacteria	Alphaproteobacteria	Rhodobacterales	Rhodobacteraceae	unclassified Rhodobacteraceae	Rhodobacteraceae bacterium SB2
1	Bacteria	Proteobacteria	Alphaproteobacteria	Rhodobacterales	Rhodobacteraceae	unclassified Rhodobacteraceae	Rhodobacteraceae bacterium KLH11
1	Bacteria	Proteobacteria	Alphaproteobacteria	Rhodobacterales	Rhodobacteraceae	unclassified Rhodobacteraceae	Rhodobacteraceae bacterium HTCC2150
1	Bacteria	Proteobacteria	Alphaproteobacteria	Rhodobacterales	Rhodobacteraceae	unclassified Rhodobacteraceae	Rhodobacteraceae bacterium HLUCCO18
1	Bacteria	Proteobacteria	Alphaproteobacteria	Rhodobacterales	Rhodobacteraceae	unclassified Rhodobacteraceae	Rhodobacteraceae bacterium HLUCCO07
1	Bacteria	Proteobacteria	Alphaproteobacteria	Rhodobacterales	Rhodobacteraceae	unclassified Rhodobacteraceae	Rhodobacteraceae bacterium HLUCCA12
1	Bacteria	Proteobacteria	Alphaproteobacteria	Rhodobacterales	Rhodobacteraceae	unclassified Rhodobacteraceae	Rhodobacteraceae bacterium CY02
1	Bacteria	Proteobacteria	Alphaproteobacteria	Rhodobacterales	Rhodobacteraceae	unclassified Rhodobacteraceae	Rhodobacteraceae bacterium CG2_30_10_405
1	Bacteria	Proteobacteria	Alphaproteobacteria	Rhodobacterales	Rhodobacteraceae	Yangia	Yangia pacifica
1	Bacteria	Proteobacteria	Alphaproteobacteria	Rhodobacterales	Rhodobacteraceae	Wenxinia	Wenxinia saemankumensis
1	Bacteria	Proteobacteria	Alphaproteobacteria	Rhodobacterales	Rhodobacteraceae	Wenxinia	Wenxinia marina
1	Bacteria	Proteobacteria	Alphaproteobacteria	Rhodobacterales	Rhodobacteraceae	Tropicimonas	Tropicimonas isoalkanivorans
1	Bacteria	Proteobacteria	Alphaproteobacteria	Rhodobacterales	Rhodobacteraceae	Tropicibacter	Tropicibacter multivorans
1	Bacteria	Proteobacteria	Alphaproteobacteria	Rhodobacterales	Rhodobacteraceae	Thioclava	Thioclava sp. SK-1
1	Bacteria	Proteobacteria	Alphaproteobacteria	Rhodobacterales	Rhodobacteraceae	Thioclava	Thioclava dalianensis
1	Bacteria	Proteobacteria	Alphaproteobacteria	Rhodobacterales	Rhodobacteraceae	Thioclava	Thioclava atlantica
1	Bacteria	Proteobacteria	Alphaproteobacteria	Rhodobacterales	Rhodobacteraceae	Thioclava	
1	Bacteria	Proteobacteria	Alphaproteobacteria	Rhodobacterales	Rhodobacteraceae	Thalassobius	Thalassobius mediterraneus
1	Bacteria	Proteobacteria	Alphaproteobacteria	Rhodobacterales	Rhodobacteraceae	Thalassobius	Thalassobius maritimus
1	Bacteria	Proteobacteria	Alphaproteobacteria	Rhodobacterales	Rhodobacteraceae	Thalassobius	Thalassobius gelatinovorus
1	Bacteria	Proteobacteria	Alphaproteobacteria	Rhodobacterales	Rhodobacteraceae	Tateyamaria	Tateyamaria sp. ANG-S1
1	Bacteria	Proteobacteria	Alphaproteobacteria	Rhodobacterales	Rhodobacteraceae	Tateyamaria	
1	Bacteria	Proteobacteria	Alphaproteobacteria	Rhodobacterales	Rhodobacteraceae	Sulfitobacter	Sulfitobacter sp. EhC04
1	Bacteria	Proteobacteria	Alphaproteobacteria	Rhodobacterales	Rhodobacteraceae	Sulfitobacter	Sulfitobacter mediterraneus
1	Bacteria	Proteobacteria	Alphaproteobacteria	Rhodobacterales	Rhodobacteraceae	Sulfitobacter	Sulfitobacter delicatus
1	Bacteria	Proteobacteria	Alphaproteobacteria	Rhodobacterales	Rhodobacteraceae	Shimia	Shimia marina
1	Bacteria	Proteobacteria	Alphaproteobacteria	Rhodobacterales	Rhodobacteraceae	Shimia	Shimia haliotis
1	Bacteria	Proteobacteria	Alphaproteobacteria	Rhodobacterales	Rhodobacteraceae	Sediminimonas	Sediminimonas qiaohouensis
1	Bacteria	Proteobacteria	Alphaproteobacteria	Rhodobacterales	Rhodobacteraceae	Sedimentitalea	Sedimentitalea nanhaiensis
1	Bacteria	Proteobacteria	Alphaproteobacteria	Rhodobacterales	Rhodobacteraceae	Ruegeria	Ruegeria sp. TM1040
1	Bacteria	Proteobacteria	Alphaproteobacteria	Rhodobacterales	Rhodobacteraceae	Ruegeria	Ruegeria halocynthiae
1	Bacteria	Proteobacteria	Alphaproteobacteria	Rhodobacterales	Rhodobacteraceae	Rubrimonas	Rubrimonas cliftonensis
1	Bacteria	Proteobacteria	Alphaproteobacteria	Rhodobacterales	Rhodobacteraceae	Rubellimicrobium	Rubellimicrobium thermophilum
1	Bacteria	Proteobacteria	Alphaproteobacteria	Rhodobacterales	Rhodobacteraceae	Rubellimicrobium	Rubellimicrobium mesophilum
1	Bacteria	Proteobacteria	Alphaproteobacteria	Rhodobacterales	Rhodobacteraceae	Rubellimicrobium	
1	Bacteria	Proteobacteria	Alphaproteobacteria	Rhodobacterales	Rhodobacteraceae	Roseovarius	Roseovarius sp. HI0049
1	Bacteria	Proteobacteria	Alphaproteobacteria	Rhodobacterales	Rhodobacteraceae	Roseovarius	Roseovarius azorensis
1	Bacteria	Proteobacteria	Alphaproteobacteria	Rhodobacterales	Rhodobacteraceae	Roseobacter	Roseobacter sp. SK209-2-6
1	Bacteria	Proteobacteria	Alphaproteobacteria	Rhodobacterales	Rhodobacteraceae	Roseobacter	Roseobacter sp. AzwK-3b
1	Bacteria	Proteobacteria	Alphaproteobacteria	Rhodobacterales	Rhodobacteraceae	Roseobacter	Roseobacter litoralis
1	Bacteria	Proteobacteria	Alphaproteobacteria	Rhodobacterales	Rhodobacteraceae	Roseivivax	Roseivivax marinus
1	Bacteria	Proteobacteria	Alphaproteobacteria	Rhodobacterales	Rhodobacteraceae	Roseivivax	Roseivivax lentus
1	Bacteria	Proteobacteria	Alphaproteobacteria	Rhodobacterales	Rhodobacteraceae	Roseivivax	Roseivivax jejudonensis
1	Bacteria	Proteobacteria	Alphaproteobacteria	Rhodobacterales	Rhodobacteraceae	Roseibacterium	Roseibacterium elongatum
1	Bacteria	Proteobacteria	Alphaproteobacteria	Rhodobacterales	Rhodobacteraceae	Roseibaca	Roseibaca calidilacus
1	Bacteria	Proteobacteria	Alphaproteobacteria	Rhodobacterales	Rhodobacteraceae	Rhodovulum	Rhodovulum sulfidophilum
1	Bacteria	Proteobacteria	Alphaproteobacteria	Rhodobacterales	Rhodobacteraceae	Rhodovulum	Rhodovulum sp. PH10
1	Bacteria	Proteobacteria	Alphaproteobacteria	Rhodobacterales	Rhodobacteraceae	Rhodovulum	Rhodovulum sp. NI22
1	Bacteria	Proteobacteria	Alphaproteobacteria	Rhodobacterales	Rhodobacteraceae	Rhodobacter	Rhodobacter vinaykumarii
1	Bacteria	Proteobacteria	Alphaproteobacteria	Rhodobacterales	Rhodobacteraceae	Rhodobacter	Rhodobacter sp. CCB-MM2
1	Bacteria	Proteobacteria	Alphaproteobacteria	Rhodobacterales	Rhodobacteraceae	Pseudovibrio	Pseudovibrio sp. Tun.PHSC04-5.I4
1	Bacteria	Proteobacteria	Alphaproteobacteria	Rhodobacterales	Rhodobacteraceae	Pseudovibrio	Pseudovibrio sp. FO-BEG1
1	Bacteria	Proteobacteria	Alphaproteobacteria	Rhodobacterales	Rhodobacteraceae	Pseudovibrio	Pseudovibrio sp. Ad13
1	Bacteria	Proteobacteria	Alphaproteobacteria	Rhodobacterales	Rhodobacteraceae	Pseudoruegeria	Pseudoruegeria sabulilitoris
1	Bacteria	Proteobacteria	Alphaproteobacteria	Rhodobacterales	Rhodobacteraceae	Pseudoruegeria	Pseudoruegeria marinistellae
1	Bacteria	Proteobacteria	Alphaproteobacteria	Rhodobacterales	Rhodobacteraceae	Pseudoruegeria	Pseudoruegeria lutimaris
1	Bacteria	Proteobacteria	Alphaproteobacteria	Rhodobacterales	Rhodobacteraceae	Pseudooceanicola	Pseudooceanicola flagellatus
1	Bacteria	Proteobacteria	Alphaproteobacteria	Rhodobacterales	Rhodobacteraceae	Pseudooceanicola	Pseudooceanicola atlanticus
1	Bacteria	Proteobacteria	Alphaproteobacteria	Rhodobacterales	Rhodobacteraceae	Phaeobacter	Phaeobacter sp. CECT 7735
1	Bacteria	Proteobacteria	Alphaproteobacteria	Rhodobacterales	Rhodobacteraceae	Paracoccus	Paracoccus yeei
1	Bacteria	Proteobacteria	Alphaproteobacteria	Rhodobacterales	Rhodobacteraceae	Paracoccus	Paracoccus sp. MKU1
1	Bacteria	Proteobacteria	Alphaproteobacteria	Rhodobacterales	Rhodobacteraceae	Paracoccus	Paracoccus sanguinis
1	Bacteria	Proteobacteria	Alphaproteobacteria	Rhodobacterales	Rhodobacteraceae	Paracoccus	Paracoccus pantotrophus
1	Bacteria	Proteobacteria	Alphaproteobacteria	Rhodobacterales	Rhodobacteraceae	Paracoccus	Paracoccus halophilus
1	Bacteria	Proteobacteria	Alphaproteobacteria	Rhodobacterales	Rhodobacteraceae	Paracoccus	Paracoccus aminovorans
1	Bacteria	Proteobacteria	Alphaproteobacteria	Rhodobacterales	Rhodobacteraceae	Paracoccus	Paracoccus aminophilus
1	Bacteria	Proteobacteria	Alphaproteobacteria	Rhodobacterales	Rhodobacteraceae	Paenirhodobacter	Paenirhodobacter enshiensis
1	Bacteria	Proteobacteria	Alphaproteobacteria	Rhodobacterales	Rhodobacteraceae	Octadecabacter	Octadecabacter antarcticus
1	Bacteria	Proteobacteria	Alphaproteobacteria	Rhodobacterales	Rhodobacteraceae	Oceaniovalibus	Oceaniovalibus guishaninsula
1	Bacteria	Proteobacteria	Alphaproteobacteria	Rhodobacterales	Rhodobacteraceae	Oceanicola	Oceanicola sp. HL-35
1	Bacteria	Proteobacteria	Alphaproteobacteria	Rhodobacterales	Rhodobacteraceae	Oceanicola	Oceanicola granulosus
1	Bacteria	Proteobacteria	Alphaproteobacteria	Rhodobacterales	Rhodobacteraceae	Nioella	Nioella sediminis
1	Bacteria	Proteobacteria	Alphaproteobacteria	Rhodobacterales	Rhodobacteraceae	Natronohydrobacter	Natronohydrobacter thiooxidans
1	Bacteria	Proteobacteria	Alphaproteobacteria	Rhodobacterales	Rhodobacteraceae	Marivita	Marivita cryptomonadis
1	Bacteria	Proteobacteria	Alphaproteobacteria	Rhodobacterales	Rhodobacteraceae	Maritimibacter	Maritimibacter sp. HL-12
1	Bacteria	Proteobacteria	Alphaproteobacteria	Rhodobacterales	Rhodobacteraceae	Marinovum	Marinovum algicola
1	Bacteria	Proteobacteria	Alphaproteobacteria	Rhodobacterales	Rhodobacteraceae	Mameliella	Mameliella alba
1	Bacteria	Proteobacteria	Alphaproteobacteria	Rhodobacterales	Rhodobacteraceae	Loktanella	Loktanella vestfoldensis
1	Bacteria	Proteobacteria	Alphaproteobacteria	Rhodobacterales	Rhodobacteraceae	Loktanella	Loktanella sp. DSM 29012
1	Bacteria	Proteobacteria	Alphaproteobacteria	Rhodobacterales	Rhodobacteraceae	Loktanella	Loktanella koreensis
1	Bacteria	Proteobacteria	Alphaproteobacteria	Rhodobacterales	Rhodobacteraceae	Loktanella	Loktanella cinnabarina
1	Bacteria	Proteobacteria	Alphaproteobacteria	Rhodobacterales	Rhodobacteraceae	Loktanella	
1	Bacteria	Proteobacteria	Alphaproteobacteria	Rhodobacterales	Rhodobacteraceae	Leisingera	Leisingera methylohalidivorans
1	Bacteria	Proteobacteria	Alphaproteobacteria	Rhodobacterales	Rhodobacteraceae	Leisingera	Leisingera aquimarina
1	Bacteria	Proteobacteria	Alphaproteobacteria	Rhodobacterales	Rhodobacteraceae	Leisingera	Leisingera aquaemixtae
1	Bacteria	Proteobacteria	Alphaproteobacteria	Rhodobacterales	Rhodobacteraceae	Leisingera	
1	Bacteria	Proteobacteria	Alphaproteobacteria	Rhodobacterales	Rhodobacteraceae	Labrenzia	Labrenzia alexandrii
1	Bacteria	Proteobacteria	Alphaproteobacteria	Rhodobacterales	Rhodobacteraceae	Labrenzia	Labrenzia aggregata
1	Bacteria	Proteobacteria	Alphaproteobacteria	Rhodobacterales	Rhodobacteraceae	Jannaschia	Jannaschia sp. CCS1
1	Bacteria	Proteobacteria	Alphaproteobacteria	Rhodobacterales	Rhodobacteraceae	Haematobacter	Haematobacter missouriensis
1	Bacteria	Proteobacteria	Alphaproteobacteria	Rhodobacterales	Rhodobacteraceae	Falsirhodobacter	Falsirhodobacter sp. alg1
1	Bacteria	Proteobacteria	Alphaproteobacteria	Rhodobacterales	Rhodobacteraceae	Defluviimonas	Defluviimonas indica
1	Bacteria	Proteobacteria	Alphaproteobacteria	Rhodobacterales	Rhodobacteraceae	Defluviimonas	
1	Bacteria	Proteobacteria	Alphaproteobacteria	Rhodobacterales	Rhodobacteraceae	Cribrihabitans	Cribrihabitans marinus
1	Bacteria	Proteobacteria	Alphaproteobacteria	Rhodobacterales	Rhodobacteraceae	Citreimonas	Citreimonas salinaria
1	Bacteria	Proteobacteria	Alphaproteobacteria	Rhodobacterales	Rhodobacteraceae	Citreicella	Citreicella sp. 357
1	Bacteria	Proteobacteria	Alphaproteobacteria	Rhodobacterales	Rhodobacteraceae	Celeribacter	Celeribacter baekdonensis
1	Bacteria	Proteobacteria	Alphaproteobacteria	Rhodobacterales	Rhodobacteraceae	Aquimixticola	Aquimixticola soesokkakensis
1	Bacteria	Proteobacteria	Alphaproteobacteria	Rhodobacterales	Rhodobacteraceae	Aliiroseovarius	Aliiroseovarius crassostreae
1	Bacteria	Proteobacteria	Alphaproteobacteria	Rhodobacterales	Rhodobacteraceae	Actibacterium	Actibacterium mucosum
1	Bacteria	Proteobacteria	Alphaproteobacteria	Rhodobacterales	Hyphomonadaceae	unclassified Hyphomonadaceae	Hyphomonadaceae bacterium UKL13-1
1	Bacteria	Proteobacteria	Alphaproteobacteria	Rhodobacterales	Hyphomonadaceae	unclassified Hyphomonadaceae	Hyphomonadaceae bacterium BRH_c29
1	Bacteria	Proteobacteria	Alphaproteobacteria	Rhodobacterales	Hyphomonadaceae	Oceanicaulis	Oceanicaulis sp. HLUCCA04
1	Bacteria	Proteobacteria	Alphaproteobacteria	Rhodobacterales	Hyphomonadaceae	Oceanicaulis	
1	Bacteria	Proteobacteria	Alphaproteobacteria	Rhodobacterales	Hyphomonadaceae	Maricaulis	Maricaulis salignorans
1	Bacteria	Proteobacteria	Alphaproteobacteria	Rhodobacterales	Hyphomonadaceae	Hyphomonas	Hyphomonas polymorpha
1	Bacteria	Proteobacteria	Alphaproteobacteria	Rhodobacterales	Hyphomonadaceae	Hyphomonas	Hyphomonas johnsonii
1	Bacteria	Proteobacteria	Alphaproteobacteria	Rhodobacterales	Hyphomonadaceae	Hyphomonas	Hyphomonas adhaerens
1	Bacteria	Proteobacteria	Alphaproteobacteria	Rhodobacterales	Hyphomonadaceae	Henriciella	Henriciella sp. LA220
1	Bacteria	Proteobacteria	Alphaproteobacteria	Rhodobacterales	Hyphomonadaceae	Henriciella	Henriciella marina
1	Bacteria	Proteobacteria	Alphaproteobacteria	Rhodobacterales	Hyphomonadaceae	Henriciella	Henriciella aquimarina
1	Bacteria	Proteobacteria	Alphaproteobacteria	Rhodobacterales	Hyphomonadaceae	Hellea	Hellea balneolensis
1	Bacteria	Proteobacteria	Alphaproteobacteria	Rhizobiales	unclassified Rhizobiales	unclassified Rhizobiales (miscellaneous)	Rhizobiales bacterium MIMtkB18
1	Bacteria	Proteobacteria	Alphaproteobacteria	Rhizobiales	unclassified Rhizobiales	unclassified Rhizobiales (miscellaneous)	Rhizobiales bacterium HL-109
1	Bacteria	Proteobacteria	Alphaproteobacteria	Rhizobiales	unclassified Rhizobiales	unclassified Rhizobiales (miscellaneous)	Rhizobiales bacterium CCH3-A5
1	Bacteria	Proteobacteria	Alphaproteobacteria	Rhizobiales	unclassified Rhizobiales	unclassified Rhizobiales (miscellaneous)	Rhizobiales bacterium CCH10-E5
1	Bacteria	Proteobacteria	Alphaproteobacteria	Rhizobiales	unclassified Rhizobiales	unclassified Rhizobiales (miscellaneous)	Rhizobiales bacterium 63-7
1	Bacteria	Proteobacteria	Alphaproteobacteria	Rhizobiales	unclassified Rhizobiales	unclassified Rhizobiales (miscellaneous)	Rhizobiales bacterium 63-22
1	Bacteria	Proteobacteria	Alphaproteobacteria	Rhizobiales	unclassified Rhizobiales	unclassified Rhizobiales (miscellaneous)	Rhizobiales bacterium 62-47
1	Bacteria	Proteobacteria	Alphaproteobacteria	Rhizobiales	unclassified Rhizobiales	Methyloligella	Methyloligella halotolerans
1	Bacteria	Proteobacteria	Alphaproteobacteria	Rhizobiales	unclassified Rhizobiales	Methyloceanibacter	Methyloceanibacter stevinii
1	Bacteria	Proteobacteria	Alphaproteobacteria	Rhizobiales	unclassified Rhizobiales	Methyloceanibacter	
1	Bacteria	Proteobacteria	Alphaproteobacteria	Rhizobiales	unclassified Rhizobiales		
1	Bacteria	Proteobacteria	Alphaproteobacteria	Rhizobiales	Xanthobacteraceae	Xanthobacter	Xanthobacter sp. 126
1	Bacteria	Proteobacteria	Alphaproteobacteria	Rhizobiales	Xanthobacteraceae	Xanthobacter	Xanthobacter autotrophicus
1	Bacteria	Proteobacteria	Alphaproteobacteria	Rhizobiales	Xanthobacteraceae	Pseudoxanthobacter	Pseudoxanthobacter soli
1	Bacteria	Proteobacteria	Alphaproteobacteria	Rhizobiales	Xanthobacteraceae	Ancylobacter	
1	Bacteria	Proteobacteria	Alphaproteobacteria	Rhizobiales	Xanthobacteraceae		
1	Bacteria	Proteobacteria	Alphaproteobacteria	Rhizobiales	Rhodobiaceae	Parvibaculum	Parvibaculum lavamentivorans
1	Bacteria	Proteobacteria	Alphaproteobacteria	Rhizobiales	Rhodobiaceae	Lutibaculum	Lutibaculum baratangense
1	Bacteria	Proteobacteria	Alphaproteobacteria	Rhizobiales	Rhizobiaceae	Sinorhizobium/Ensifer group	Sinorhizobium
1	Bacteria	Proteobacteria	Alphaproteobacteria	Rhizobiales	Rhizobiaceae	Sinorhizobium/Ensifer group	Ensifer
1	Bacteria	Proteobacteria	Alphaproteobacteria	Rhizobiales	Rhizobiaceae	Sinorhizobium/Ensifer group	Ensifer
1	Bacteria	Proteobacteria	Alphaproteobacteria	Rhizobiales	Rhizobiaceae	Rhizobium/Agrobacterium group	Rhizobium
1	Bacteria	Proteobacteria	Alphaproteobacteria	Rhizobiales	Rhizobiaceae	Rhizobium/Agrobacterium group	Rhizobium
1	Bacteria	Proteobacteria	Alphaproteobacteria	Rhizobiales	Rhizobiaceae	Rhizobium/Agrobacterium group	Rhizobium
1	Bacteria	Proteobacteria	Alphaproteobacteria	Rhizobiales	Rhizobiaceae	Rhizobium/Agrobacterium group	Rhizobium
1	Bacteria	Proteobacteria	Alphaproteobacteria	Rhizobiales	Rhizobiaceae	Rhizobium/Agrobacterium group	Rhizobium
1	Bacteria	Proteobacteria	Alphaproteobacteria	Rhizobiales	Rhizobiaceae	Rhizobium/Agrobacterium group	Rhizobium
1	Bacteria	Proteobacteria	Alphaproteobacteria	Rhizobiales	Rhizobiaceae	Rhizobium/Agrobacterium group	Rhizobium
1	Bacteria	Proteobacteria	Alphaproteobacteria	Rhizobiales	Rhizobiaceae	Rhizobium/Agrobacterium group	Rhizobium
1	Bacteria	Proteobacteria	Alphaproteobacteria	Rhizobiales	Rhizobiaceae	Rhizobium/Agrobacterium group	Rhizobium
1	Bacteria	Proteobacteria	Alphaproteobacteria	Rhizobiales	Rhizobiaceae	Rhizobium/Agrobacterium group	Rhizobium
1	Bacteria	Proteobacteria	Alphaproteobacteria	Rhizobiales	Rhizobiaceae	Rhizobium/Agrobacterium group	Rhizobium
1	Bacteria	Proteobacteria	Alphaproteobacteria	Rhizobiales	Rhizobiaceae	Rhizobium/Agrobacterium group	Pararhizobium
1	Bacteria	Proteobacteria	Alphaproteobacteria	Rhizobiales	Rhizobiaceae	Rhizobium/Agrobacterium group	Neorhizobium
1	Bacteria	Proteobacteria	Alphaproteobacteria	Rhizobiales	Rhizobiaceae	Rhizobium/Agrobacterium group	Agrobacterium
1	Bacteria	Proteobacteria	Alphaproteobacteria	Rhizobiales	Rhizobiaceae	Rhizobium/Agrobacterium group	Agrobacterium
1	Bacteria	Proteobacteria	Alphaproteobacteria	Rhizobiales	Rhizobiaceae	Rhizobium/Agrobacterium group	Agrobacterium
1	Bacteria	Proteobacteria	Alphaproteobacteria	Rhizobiales	Rhizobiaceae	Liberibacter	Liberibacter crescens
1	Bacteria	Proteobacteria	Alphaproteobacteria	Rhizobiales	Rhizobiaceae	Kaistia	Kaistia sp. SCN 65-12
1	Bacteria	Proteobacteria	Alphaproteobacteria	Rhizobiales	Rhizobiaceae	Kaistia	Kaistia granuli
1	Bacteria	Proteobacteria	Alphaproteobacteria	Rhizobiales	Rhizobiaceae	Kaistia	
1	Bacteria	Proteobacteria	Alphaproteobacteria	Rhizobiales	Phyllobacteriaceae	Phyllobacterium	Phyllobacterium sp. YR531
1	Bacteria	Proteobacteria	Alphaproteobacteria	Rhizobiales	Phyllobacteriaceae	Phyllobacterium	Phyllobacterium sp. OV277
1	Bacteria	Proteobacteria	Alphaproteobacteria	Rhizobiales	Phyllobacteriaceae	Nitratireductor	Nitratireductor pacificus
1	Bacteria	Proteobacteria	Alphaproteobacteria	Rhizobiales	Phyllobacteriaceae	Nitratireductor	
1	Bacteria	Proteobacteria	Alphaproteobacteria	Rhizobiales	Phyllobacteriaceae	Mesorhizobium	Mesorhizobium sp. URHA0056
1	Bacteria	Proteobacteria	Alphaproteobacteria	Rhizobiales	Phyllobacteriaceae	Mesorhizobium	Mesorhizobium sp. SCN 65-20
1	Bacteria	Proteobacteria	Alphaproteobacteria	Rhizobiales	Phyllobacteriaceae	Mesorhizobium	Mesorhizobium sp. Root552
1	Bacteria	Proteobacteria	Alphaproteobacteria	Rhizobiales	Phyllobacteriaceae	Mesorhizobium	Mesorhizobium sp. Root157
1	Bacteria	Proteobacteria	Alphaproteobacteria	Rhizobiales	Phyllobacteriaceae	Mesorhizobium	Mesorhizobium sp. LSHC420B00
1	Bacteria	Proteobacteria	Alphaproteobacteria	Rhizobiales	Phyllobacteriaceae	Mesorhizobium	Mesorhizobium sp. F7
1	Bacteria	Proteobacteria	Alphaproteobacteria	Rhizobiales	Phyllobacteriaceae	Mesorhizobium	Mesorhizobium sp. AA22
1	Bacteria	Proteobacteria	Alphaproteobacteria	Rhizobiales	Phyllobacteriaceae	Mesorhizobium	Mesorhizobium plurifarium
1	Bacteria	Proteobacteria	Alphaproteobacteria	Rhizobiales	Phyllobacteriaceae	Mesorhizobium	Mesorhizobium ciceri
1	Bacteria	Proteobacteria	Alphaproteobacteria	Rhizobiales	Phyllobacteriaceae	Hoeflea	Hoeflea olei
1	Bacteria	Proteobacteria	Alphaproteobacteria	Rhizobiales	Phyllobacteriaceae		
1	Bacteria	Proteobacteria	Alphaproteobacteria	Rhizobiales	Methylocystaceae	Terasakiella	Terasakiella pusilla
1	Bacteria	Proteobacteria	Alphaproteobacteria	Rhizobiales	Methylocystaceae	Pleomorphomonas	
1	Bacteria	Proteobacteria	Alphaproteobacteria	Rhizobiales	Methylocystaceae	Methylopila	Methylopila sp. 73B
1	Bacteria	Proteobacteria	Alphaproteobacteria	Rhizobiales	Methylobacteriaceae	Microvirga	Microvirga vignae
1	Bacteria	Proteobacteria	Alphaproteobacteria	Rhizobiales	Methylobacteriaceae	Microvirga	Microvirga guangxiensis
1	Bacteria	Proteobacteria	Alphaproteobacteria	Rhizobiales	Methylobacteriaceae	Methylobacterium	Methylobacterium sp. UNC378MF
1	Bacteria	Proteobacteria	Alphaproteobacteria	Rhizobiales	Methylobacteriaceae	Methylobacterium	Methylobacterium sp. Leaf91
1	Bacteria	Proteobacteria	Alphaproteobacteria	Rhizobiales	Methylobacteriaceae	Methylobacterium	Methylobacterium sp. Leaf89
1	Bacteria	Proteobacteria	Alphaproteobacteria	Rhizobiales	Methylobacteriaceae	Methylobacterium	Methylobacterium sp. Leaf125
1	Bacteria	Proteobacteria	Alphaproteobacteria	Rhizobiales	Methylobacteriaceae	Methylobacterium	Methylobacterium sp. Leaf122
1	Bacteria	Proteobacteria	Alphaproteobacteria	Rhizobiales	Methylobacteriaceae	Methylobacterium	Methylobacterium sp. Leaf108
1	Bacteria	Proteobacteria	Alphaproteobacteria	Rhizobiales	Methylobacteriaceae	Methylobacterium	Methylobacterium sp. 77
1	Bacteria	Proteobacteria	Alphaproteobacteria	Rhizobiales	Methylobacteriaceae	Methylobacterium	Methylobacterium pseudosasicola
1	Bacteria	Proteobacteria	Alphaproteobacteria	Rhizobiales	Methylobacteriaceae	Methylobacterium	Methylobacterium populi
1	Bacteria	Proteobacteria	Alphaproteobacteria	Rhizobiales	Methylobacteriaceae	Methylobacterium	Methylobacterium platani

1	Bacteria	Proteobacteria	Alphaproteobacteria	Rhizobiales	Methylobacteriaceae	Methylobacterium	Methylobacterium nodulans
1	Bacteria	Proteobacteria	Alphaproteobacteria	Rhizobiales	Methylobacteriaceae	Methylobacterium	Methylobacterium extorquens group
1	Bacteria	Proteobacteria	Alphaproteobacteria	Rhizobiales	Methylobacteriaceae	Methylobacterium	Methylobacterium aquaticum
1	Bacteria	Proteobacteria	Alphaproteobacteria	Rhizobiales	Hyphomicrobiaceae	Rhodoplanes	Rhodoplanes sp. Z2-YC6860
1	Bacteria	Proteobacteria	Alphaproteobacteria	Rhizobiales	Hyphomicrobiaceae	Pelagibacterium	Pelagibacterium sp. SCN 64-44
1	Bacteria	Proteobacteria	Alphaproteobacteria	Rhizobiales	Hyphomicrobiaceae	Pelagibacterium	Pelagibacterium sp. SCN 63-23
1	Bacteria	Proteobacteria	Alphaproteobacteria	Rhizobiales	Hyphomicrobiaceae	Hyphomicrobium	Hyphomicrobium sp. 99
1	Bacteria	Proteobacteria	Alphaproteobacteria	Rhizobiales	Hyphomicrobiaceae	Filomicrobium	
1	Bacteria	Proteobacteria	Alphaproteobacteria	Rhizobiales	Hyphomicrobiaceae	Devosia	Devosia sp. Root685
1	Bacteria	Proteobacteria	Alphaproteobacteria	Rhizobiales	Hyphomicrobiaceae	Devosia	Devosia sp. Leaf420
1	Bacteria	Proteobacteria	Alphaproteobacteria	Rhizobiales	Hyphomicrobiaceae	Devosia	Devosia sp. H5989
1	Bacteria	Proteobacteria	Alphaproteobacteria	Rhizobiales	Hyphomicrobiaceae	Devosia	Devosia sp. A16
1	Bacteria	Proteobacteria	Alphaproteobacteria	Rhizobiales	Hyphomicrobiaceae	Devosia	Devosia sp. 67-54
1	Bacteria	Proteobacteria	Alphaproteobacteria	Rhizobiales	Hyphomicrobiaceae	Devosia	Devosia sp. 66-22
1	Bacteria	Proteobacteria	Alphaproteobacteria	Rhizobiales	Hyphomicrobiaceae	Devosia	Devosia soli
1	Bacteria	Proteobacteria	Alphaproteobacteria	Rhizobiales	Hyphomicrobiaceae	Devosia	Devosia psychrophila
1	Bacteria	Proteobacteria	Alphaproteobacteria	Rhizobiales	Hyphomicrobiaceae	Cucumibacter	Cucumibacter marinus
1	Bacteria	Proteobacteria	Alphaproteobacteria	Rhizobiales	Hyphomicrobiaceae	Blastochloris	Blastochloris viridis
1	Bacteria	Proteobacteria	Alphaproteobacteria	Rhizobiales	Cohaesibacteraceae	Cohaesibacter	Cohaesibacter marisflavi
1	Bacteria	Proteobacteria	Alphaproteobacteria	Rhizobiales	Brucellaceae	Pseudochrobactrum	
1	Bacteria	Proteobacteria	Alphaproteobacteria	Rhizobiales	Brucellaceae	Ochrobactrum	Ochrobactrum sp. P6BS-III
1	Bacteria	Proteobacteria	Alphaproteobacteria	Rhizobiales	Brucellaceae	Ochrobactrum	Ochrobactrum cytisi
1	Bacteria	Proteobacteria	Alphaproteobacteria	Rhizobiales	Brucellaceae	Brucella	Brucella ovis
1	Bacteria	Proteobacteria	Alphaproteobacteria	Rhizobiales	Brucellaceae	Brucella	Brucella melitensis
1	Bacteria	Proteobacteria	Alphaproteobacteria	Rhizobiales	Brucellaceae	Brucella	
1	Bacteria	Proteobacteria	Alphaproteobacteria	Rhizobiales	Bradyrhizobiaceae	Tardiphaga	Tardiphaga robiniae
1	Bacteria	Proteobacteria	Alphaproteobacteria	Rhizobiales	Bradyrhizobiaceae	Rhodopseudomonas	Rhodopseudomonas sp. B29
1	Bacteria	Proteobacteria	Alphaproteobacteria	Rhizobiales	Bradyrhizobiaceae	Nitrobacter	Nitrobacter winogradskyi
1	Bacteria	Proteobacteria	Alphaproteobacteria	Rhizobiales	Bradyrhizobiaceae	Nitrobacter	Nitrobacter vulgaris
1	Bacteria	Proteobacteria	Alphaproteobacteria	Rhizobiales	Bradyrhizobiaceae	Nitrobacter	Nitrobacter hamburgensis
1	Bacteria	Proteobacteria	Alphaproteobacteria	Rhizobiales	Bradyrhizobiaceae	Bradyrhizobium	Bradyrhizobium valentinum
1	Bacteria	Proteobacteria	Alphaproteobacteria	Rhizobiales	Bradyrhizobiaceae	Bradyrhizobium	Bradyrhizobium sp. URHA0002
1	Bacteria	Proteobacteria	Alphaproteobacteria	Rhizobiales	Bradyrhizobiaceae	Bradyrhizobium	Bradyrhizobium sp. Tv2a-2
1	Bacteria	Proteobacteria	Alphaproteobacteria	Rhizobiales	Bradyrhizobiaceae	Bradyrhizobium	Bradyrhizobium sp. STM 3843
1	Bacteria	Proteobacteria	Alphaproteobacteria	Rhizobiales	Bradyrhizobiaceae	Bradyrhizobium	Bradyrhizobium sp. STM 3809
1	Bacteria	Proteobacteria	Alphaproteobacteria	Rhizobiales	Bradyrhizobiaceae	Bradyrhizobium	Bradyrhizobium sp. LTSP849
1	Bacteria	Proteobacteria	Alphaproteobacteria	Rhizobiales	Bradyrhizobiaceae	Bradyrhizobium	Bradyrhizobium sp. LMTR 3
1	Bacteria	Proteobacteria	Alphaproteobacteria	Rhizobiales	Bradyrhizobiaceae	Bradyrhizobium	Bradyrhizobium sp. Gha
1	Bacteria	Proteobacteria	Alphaproteobacteria	Rhizobiales	Bradyrhizobiaceae	Bradyrhizobium	Bradyrhizobium sp. DOA1
1	Bacteria	Proteobacteria	Alphaproteobacteria	Rhizobiales	Bradyrhizobiaceae	Bradyrhizobium	Bradyrhizobium sp. BR 10245
1	Bacteria	Proteobacteria	Alphaproteobacteria	Rhizobiales	Bradyrhizobiaceae	Bradyrhizobium	Bradyrhizobium sp. Ai1a-2
[illegible]	[illegible]	[illegible]	[illegible]	[illegible]	[illegible]	[illegible]	[illegible]

1	Archaea	TACK group	Candidatus Korarchaeota	Candidatus Korarchaeum	Candidatus Korarchaeum cryptofilum		
1	Archaea	TACK group	Candidatus Bathyarchaeota	unclassified Candidatus Bathyarchaeota	miscellaneous Crenarchaeota group archaeon SMTZ-80		
1	Archaea	TACK group	Candidatus Bathyarchaeota	MCG-6	miscellaneous Crenarchaeota group-6 archaeon AD8-1		
1	Archaea	Euryarchaeota	environmental samples	uncultured marine group II/III euryarchaeote KM3_169_H09			
1	Archaea	Euryarchaeota	Thermoplasmata	Thermoplasmatales	Ferroplasmaceae	Acidiplasma	Acidiplasma aeolicum
1	Archaea	Euryarchaeota	Thermoplasmata	Methanomassiliicoccales	Methanomassiliicoccaceae	Candidatus Methanoplasma	Candidatus Methanoplasma termitum
1	Archaea	Euryarchaeota	Thermococci	Thermococcales	Thermococcaceae	Thermococcus	Thermococcus zilligii
1	Archaea	Euryarchaeota	Methanomicrobia	Methanosarcinales	unclassified Methanosarcinales	ANME-2 cluster	Candidatus Syntrophoarchaeum
1	Archaea	Euryarchaeota	Methanomicrobia	Methanosarcinales	Methanosarcinaceae	Methanosarcina	Methanosarcina sp. 2.H.A.1B.4
1	Archaea	Euryarchaeota	Methanomicrobia	Methanosarcinales	Methanosarcinaceae	Methanosarcina	
1	Archaea	Euryarchaeota	Methanomicrobia	Methanosarcinales	Methanosarcinaceae	Methanolobus	Methanolobus vulcani
1	Archaea	Euryarchaeota	Methanomicrobia	Methanosarcinales	Methanosaetaceae	Methanosaeta	Methanosaeta harundinacea
1	Archaea	Euryarchaeota	Methanomicrobia	Methanomicrobiales	Methanomicrobiaceae	Methanoculleus	Methanoculleus bourgensis
1	Archaea	Euryarchaeota	Methanomicrobia	Methanocellales	Methanocellaceae	Methanocella	Methanocella conradii
1	Archaea	Euryarchaeota	Methanococci	Methanococcales	Methanococcaceae	Methanococcus	Methanococcus maripaludis
1	Archaea	Euryarchaeota	Methanococci	Methanococcales	Methanocaldococcaceae	Methanotorris	Methanotorris igneus
1	Archaea	Euryarchaeota	Halobacteria	Natrialbales	Natrialbaceae	Haloterrigena	Haloterrigena limicola
1	Archaea	Euryarchaeota	Halobacteria	Haloferacales	Haloferacaceae	Haloferax	Haloferax elongans
1	Archaea	Euryarchaeota	Halobacteria	Halobacteriales	Haloarculaceae	Natronomonas	Natronomonas pharaonis
1	Archaea	Euryarchaeota	Halobacteria	Halobacteriales	Haloarculaceae	Halomicrobium	Halomicrobium mukohataei
1	unclassified bacterial viruses	Aeromonas phage phiARM81ld					
1	dsDNA viruses, no RNA stage	unclassified dsDNA viruses	Klosneuvirinae	Klosneuvirus	Klosneuvirus KNV1		
1	dsDNA viruses, no RNA stage	unclassified dsDNA phages	Synechococcus phage S-CBP2				
1	dsDNA viruses, no RNA stage	Poxviridae	Chordopoxvirinae	Orthopoxvirus	Vaccinia virus	Vaccinia virus GLV-1h68	
1	dsDNA viruses, no RNA stage	Phycodnaviridae	Chlorovirus	unclassified Chlorovirus	Acanthocystis turfacea Chlorella virus GM0701.1		
1	dsDNA viruses, no RNA stage	Caudovirales	Siphoviridae	Lambdavirus	unclassified Lambda-like viruses	Escherichia phage HK639	
1	dsDNA viruses, no RNA stage	Caudovirales	Siphoviridae	Lambdavirus	Escherichia virus Lambda		
1	dsDNA viruses, no RNA stage	Caudovirales	Siphoviridae	Hk578virus	Escherichia virus EK99P1	Escherichia phage EK99P-1	
1	dsDNA viruses, no RNA stage	Caudovirales	Podoviridae	unclassified Podoviridae	Salmonella phage 7-11		
1	dsDNA viruses, no RNA stage	Caudovirales	Podoviridae	unclassified Podoviridae	Rhodoferax phage P26218		
1	dsDNA viruses, no RNA stage	Caudovirales	Podoviridae	P22virus	Shigella phage Sf6		
1	dsDNA viruses, no RNA stage	Caudovirales	Myoviridae	unclassified Myoviridae	Vibrio phage vB_VaiP_IME234		
1	dsDNA viruses, no RNA stage	Caudovirales	Myoviridae	unclassified Myoviridae	Shigella phage SfIV		
1	dsDNA viruses, no RNA stage	Caudovirales	Myoviridae	unclassified Myoviridae	Pseudomonas phage vB_PsyM_KIL1		
1	dsDNA viruses, no RNA stage	Caudovirales	Myoviridae	unclassified Myoviridae	Enterobacteria phage phiP27		
1	dsDNA viruses, no RNA stage	Caudovirales	Myoviridae	Peduovirinae	Hp1virus	Aeromonas virus phiO18P	
1	dsDNA viruses, no RNA stage	Caudovirales	Myoviridae	Msw3virus	Edwardsiella virus MSW3	Edwardsiella phage MSW-3	
1	dsDNA viruses, no RNA stage	Caudovirales	Myoviridae	Msw3virus			
1	Retro-transcribing viruses	Retroviridae	Orthoretrovirinae	Gammaretrovirus	Mammalian gammaretrovirus group	Replication competent viruses	Feline leukemia virus

21967

Supplementary Information 4

Uncovering bacterial and functional diversity in macroinvertebrate mitochondrial-metagenomic datasets by differential centrifugation

Jan-Niklas Macher (JNM)[1], Arjen Speksnijder (AS)[1], Le Qin Choo (LQC)[1], Berry van der Hoorn (BvdH)[1], Willem Renema (WR)[1]*
[1] Naturalis Biodiversity Center, PO Box 9517, 2300 RA Leiden, Netherlands
[*] Corresponding author

Supplementary Information 4: Abundance of taxa identified across the 'enriched' samples using Kaiju

Hits	Domain	Phylum	Class	Order	Family	Genus	Species, strain or clade
2287	Bacteria	Proteobacteria	Betaproteobacteria	Burkholderiales			
1912	Bacteria	Proteobacteria	Betaproteobacteria	Burkholderiales	Comamonadaceae	Hydrogenophaga	
1902	Bacteria						
1590	/						
1473	Bacteria	Proteobacteria	Betaproteobacteria	Burkholderiales	Comamonadaceae	Hydrogenophaga	Hydrogenophaga sp. A37
1408	Bacteria	Proteobacteria					
1280	Bacteria	Proteobacteria	Alphaproteobacteria	Sphingomonadales	Sphingomonadaceae	Novosphingobium	
1149	Bacteria	Proteobacteria	Alphaproteobacteria	Rhodobacterales	Rhodobacteraceae		
1044	Bacteria	Proteobacteria	Alphaproteobacteria	Sphingomonadales	Sphingomonadaceae	Sphingomonas	
977	/						
955	Bacteria	Proteobacteria	Betaproteobacteria	Burkholderiales	unclassified Burkholderiales	unclassified Burkholderiales (miscellaneous)	
938	Bacteria	FCB group	Bacteroidetes/Chlorobi group	Bacteroidetes	Bacteroidia	Mucinivorans	
928	Bacteria	Proteobacteria	Betaproteobacteria	Burkholderiales	Comamonadaceae		
923	Bacteria	Proteobacteria	Betaproteobacteria	Burkholderiales	Comamonadaceae	Hydrogenophaga	Hydrogenophaga flava
762	Bacteria	Proteobacteria	Alphaproteobacteria				
760	Bacteria	Proteobacteria	Alphaproteobacteria	Rhizobiales	unclassified Rhizobiales	Candidatus Hodgkinia	Candidatus Hodgkinia cicadicola
728	Bacteria	Proteobacteria	Alphaproteobacteria	Rhodobacterales	Rhodobacteraceae	Rhodobacter	Rhodobacter sp. CACIA14H1
721	Bacteria	Proteobacteria	Alphaproteobacteria	Rhodobacterales	unclassified Rhodobacterales		
713	Bacteria	Proteobacteria	Alphaproteobacteria	Sphingomonadales	Sphingomonadaceae	Sphingopyxis	
642	Bacteria	Proteobacteria	Alphaproteobacteria	Sphingomonadales			
627	Bacteria	Proteobacteria	Alphaproteobacteria	Sphingomonadales	Sphingomonadaceae		
624	Bacteria	environmental samples	uncultured bacterium				
572	Bacteria	Proteobacteria	Alphaproteobacteria	Rickettsiales	Anaplasmataceae	Wolbachia	
500	Bacteria	Proteobacteria	Gammaproteobacteria	Alteromonadales	Shewanellaceae	Shewanella	Shewanella decolorationis
495	Bacteria	Proteobacteria	Alphaproteobacteria	Rhodobacterales	Rhodobacteraceae	Pseudorhodobacter	Pseudorhodobacter psychrotolerans
487	Bacteria	Proteobacteria	Alphaproteobacteria	Rhodobacterales			
477	Bacteria	Proteobacteria	Betaproteobacteria	Methylophilales	Methylophilaceae	Methylotenera	Methylotenera versatilis
476	Bacteria	Proteobacteria	Gammaproteobacteria	Pasteurellales	Pasteurellaceae	Histophilus	Histophilus somni
470	Bacteria	Proteobacteria	Betaproteobacteria	Burkholderiales	Comamonadaceae	Variovorax	
469	Bacteria	Proteobacteria	Gammaproteobacteria	Pseudomonadales	Pseudomonadaceae	Pseudomonas	
457	Bacteria	Proteobacteria	Alphaproteobacteria	Rickettsiales	Anaplasmataceae	Wolbachia	
453	Bacteria	Proteobacteria	Betaproteobacteria	unclassified Betaproteobacteria	unclassified Betaproteobacteria (miscellaneous)		
451	Bacteria	Terrabacteria group	Tenericutes	Mollicutes	Entomoplasmatales	unclassified Entomoplasmatales	Entomoplasmatales bacterium EntAcro10
445	Bacteria	Proteobacteria	Betaproteobacteria				
417	Bacteria	Proteobacteria	Alphaproteobacteria	Sphingomonadales	Sphingomonadaceae	Sphingopyxis	Sphingopyxis sp. LPB0140
417	Bacteria	Proteobacteria	Alphaproteobacteria	Sphingomonadales	Sphingomonadaceae	Novosphingobium	Novosphingobium barchaimii
411	Bacteria	Proteobacteria	Betaproteobacteria	Burkholderiales	Comamonadaceae	Acidovorax	
410	Bacteria	Proteobacteria	Betaproteobacteria	Burkholderiales	Comamonadaceae	Curvibacter	
406	Bacteria	Nitrospirae	Nitrospira	Nitrospirales	Nitrospiraceae	Nitrospira	Candidatus Nitrospira nitrosa
400	Bacteria	Proteobacteria	Gammaproteobacteria	Pseudomonadales	Pseudomonadaceae	Pseudomonas	Pseudomonas aeruginosa group
388	Eukaryota	Opisthokonta	Fungi	Dikarya	Ascomycota	saccharomyceta	Pezizomycotina
383	Bacteria	Terrabacteria group	Firmicutes	Clostridia	Clostridiales		
373	Bacteria	Proteobacteria	Betaproteobacteria	Burkholderiales	Comamonadaceae	Hydrogenophaga	Hydrogenophaga sp. LPB0072
366	Bacteria	Proteobacteria	Betaproteobacteria	Burkholderiales	Comamonadaceae	Ramlibacter	Ramlibacter tataouinensis
342	Eukaryota	Alveolata	Apicomplexa	Conoidasida	Coccidia	Eucoccidiorida	Eimeriorina
342	Bacteria	Proteobacteria	Betaproteobacteria	Burkholderiales	Comamonadaceae	Polaromonas	
332	Bacteria	Acidobacteria	unclassified Acidobacteria	unclassified Acidobacteria (miscellaneous)			
331	Bacteria	Proteobacteria	delta/epsilon subdivisions	Deltaproteobacteria	Desulfovibrionales	Desulfovibrionaceae	Desulfovibrio
326	Bacteria	FCB group	Bacteroidetes/Chlorobi group	Bacteroidetes	unclassified Bacteroidetes	unclassified Bacteroidetes (miscellaneous)	
319	Bacteria	Proteobacteria	Gammaproteobacteria				
317	Bacteria	Proteobacteria	Alphaproteobacteria	Rhizobiales			
317	Bacteria	Nitrospirae	Nitrospira	Nitrospirales	Nitrospiraceae	Nitrospira	Candidatus Nitrospira nitrificans
316	Bacteria	Proteobacteria	Alphaproteobacteria	Sphingomonadales	Sphingomonadaceae	Novosphingobium	Novosphingobium malaysiense
309	Bacteria	Terrabacteria group	Tenericutes	Mollicutes	Acholeplasmatales	Acholeplasmataceae	Candidatus Phytoplasma
301	Bacteria	Proteobacteria	Betaproteobacteria	Burkholderiales	Burkholderiaceae		
292	Bacteria	Proteobacteria	Gammaproteobacteria	Aeromonadales	Aeromonadaceae	Aeromonas	
290	Bacteria	Proteobacteria	Alphaproteobacteria	Rhodobacterales	Rhodobacteraceae	Rhodobacter	Rhodobacter sp. 24-YEA-8
283	Bacteria	Proteobacteria	Betaproteobacteria	Burkholderiales	unclassified Burkholderiales	unclassified Burkholderiales (miscellaneous)	Burkholderiales bacterium JOSHI_001
276	Bacteria	Proteobacteria	Alphaproteobacteria	Rhodobacterales	Rhodobacteraceae	Gemmobacter	Gemmobacter aquatilis
267	Bacteria	FCB group	Bacteroidetes/Chlorobi group	Bacteroidetes	Bacteroidia	Bacteroidales	Rikenellaceae
263	Bacteria	Acidobacteria	unclassified Acidobacteria	unclassified Acidobacteria (miscellaneous)	Acidobacteria bacterium RIFCSPLOWO2_12_FULL_67_14b		
260	Bacteria	Proteobacteria	Alphaproteobacteria	Sphingomonadales	Sphingomonadaceae	Novosphingobium	Novosphingobium lentum
257	Bacteria	FCB group	Bacteroidetes/Chlorobi group	Bacteroidetes	Bacteroidia	Bacteroidales	
256	Bacteria	Proteobacteria	Betaproteobacteria	Methylophilales	Methylophilaceae	Methylotenera	
253	Bacteria	FCB group	Bacteroidetes/Chlorobi group	Bacteroidetes	Bacteroidia	Bacteroidales	Bacteroidaceae
252	Bacteria	Proteobacteria	Betaproteobacteria	Burkholderiales	Comamonadaceae	Polaromonas	Polaromonas sp. A23
251	Bacteria	Proteobacteria	Betaproteobacteria	Burkholderiales	Comamonadaceae	Caenimonas	Caenimonas sp. SL110
249	Bacteria	Proteobacteria	Betaproteobacteria	Methylophilales	Methylophilaceae		
247	Bacteria	Proteobacteria	Alphaproteobacteria	Rhizobiales	Hyphomicrobiaceae	Hyphomicrobium	
247	Bacteria	Nitrospirae	Nitrospira	Nitrospirales	Nitrospiraceae	Nitrospira	Nitrospira sp. SG-bin1
246	Bacteria	Proteobacteria	Betaproteobacteria	Burkholderiales	Comamonadaceae	Hydrogenophaga	Hydrogenophaga sp. H7
244	Bacteria	Proteobacteria	Alphaproteobacteria	Rhizobiales	Rhizobiaceae	Rhizobium/Agrobacterium group	Rhizobium
242	Bacteria	FCB group	Bacteroidetes/Chlorobi group	Bacteroidetes	Bacteroidia	Bacteroidales	Rikenellaceae
241	Bacteria	Proteobacteria	Betaproteobacteria	Burkholderiales	unclassified Burkholderiales	Burkholderiales Genera incertae sedis	Methylibium
239	Bacteria	Proteobacteria	Betaproteobacteria	Burkholderiales	Comamonadaceae	Rhodoferax	Albidiferax sp. OV413
230	Bacteria	Proteobacteria	Betaproteobacteria	Burkholderiales	Comamonadaceae	Polaromonas	Polaromonas sp. JS666
228	Bacteria	Proteobacteria	Alphaproteobacteria	Rhizobiales	Bradyrhizobiaceae	Bradyrhizobium	
225	Bacteria	Proteobacteria	Betaproteobacteria	Burkholderiales	Comamonadaceae	Hydrogenophaga	Hydrogenophaga taeniospiralis
223	Bacteria	Proteobacteria	Alphaproteobacteria	Rhodobacterales	Rhodobacteraceae	Rhodobacter	Rhodobacter sp. SW2
222	Bacteria	Proteobacteria	Betaproteobacteria	Burkholderiales	unclassified Burkholderiales	unclassified Burkholderiales (miscellaneous)	Burkholderiales bacterium RIFCSPLOWO2_12_FULL_61_40
221	Bacteria	Proteobacteria	Betaproteobacteria	Burkholderiales	Comamonadaceae	Polaromonas	Polaromonas sp. C04
220	Bacteria	Proteobacteria	unclassified Proteobacteria	unclassified Proteobacteria (miscellaneous)	Proteobacteria bacterium ST_bin12		
218	Bacteria	Proteobacteria	Betaproteobacteria	Burkholderiales	Comamonadaceae	Rhodoferax	Rhodoferax saidenbachensis
216	Bacteria	Proteobacteria	Alphaproteobacteria	Sphingomonadales	Sphingomonadaceae	Novosphingobium	Novosphingobium sp. AAP83
214	Bacteria	FCB group	Bacteroidetes/Chlorobi group	Bacteroidetes			
211	Bacteria	Proteobacteria	Betaproteobacteria	Burkholderiales	Comamonadaceae	Hydrogenophaga	Hydrogenophaga pseudoflava
209	Bacteria	Proteobacteria	Betaproteobacteria	Burkholderiales	Comamonadaceae	Curvibacter	Curvibacter putative symbiont of Hydra magnipapillata
208	Eukaryota						
208	Bacteria	Proteobacteria	Betaproteobacteria	Rhodocyclales	Rhodocyclaceae	Azovibrio	Azovibrio restrictus
208	Bacteria	Proteobacteria	Alphaproteobacteria	Sphingomonadales	Sphingomonadaceae	Sphingobium	
206	Bacteria	Proteobacteria	Alphaproteobacteria	Rhizobiales	Phyllobacteriaceae	Mesorhizobium	
205	Eukaryota	Alveolata	Dinophyceae	Gonyaulacales	Lingulodinium	Lingulodinium polyedrum	
204	Bacteria	Proteobacteria	Alphaproteobacteria	Rickettsiales	Rickettsiaceae	Rickettsieae	Occidentia
202	Bacteria	Proteobacteria	Alphaproteobacteria	Rickettsiales	Anaplasmataceae	Wolbachieae	Wolbachia
198	Bacteria	Proteobacteria	Betaproteobacteria	Burkholderiales	unclassified Burkholderiales	unclassified Burkholderiales (miscellaneous)	Burkholderiales bacterium RIFCSPHIGHO2_12_FULL_69_20
195	Bacteria	Proteobacteria	Betaproteobacteria	Burkholderiales	unclassified Burkholderiales	Burkholderiales Genera incertae sedis	Methylibium
193	Bacteria	Proteobacteria	delta/epsilon subdivisions	Deltaproteobacteria	Desulfovibrionales	Desulfovibrionaceae	Desulfovibrio
192	Bacteria	Proteobacteria	Betaproteobacteria	Burkholderiales	unclassified Burkholderiales	Burkholderiales Genera incertae sedis	
190	Bacteria	Proteobacteria	Alphaproteobacteria	Rhodobacterales	Rhodobacteraceae	Rhodobacter	
189	Bacteria	Nitrospirae	Nitrospira	Nitrospirales	Nitrospiraceae	Nitrospira	Nitrospira sp. HN-bin3
188	Bacteria	Proteobacteria	Betaproteobacteria	Burkholderiales	Comamonadaceae	Hydrogenophaga	Hydrogenophaga sp. Root209
188	Bacteria	Proteobacteria	Betaproteobacteria	Burkholderiales	Comamonadaceae	Hydrogenophaga	Hydrogenophaga sp. IBVHS1
188	Bacteria	Proteobacteria	Alphaproteobacteria	Sphingomonadales	Sphingomonadaceae	Sphingorhabdus	Sphingorhabdus sp. M41
187	Bacteria	Proteobacteria	Betaproteobacteria	Burkholderiales	Comamonadaceae	Rhodoferax	Rhodoferax sp. DCY110
186	Bacteria	Proteobacteria	Betaproteobacteria	Burkholderiales	Comamonadaceae	Hydrogenophaga	Hydrogenophaga palleronii
184	Eukaryota	Opisthokonta	Choanoflagellida	Craspedida	Salpingoecidae	Monosiga	Monosiga brevicollis
184	Bacteria	Proteobacteria	Alphaproteobacteria	Rhodospirillales	unclassified Rhodospirillales	unclassified Rhodospirillales (miscellaneous)	Rhodospirillales bacterium 69-11
181	Bacteria	Proteobacteria	Betaproteobacteria	Burkholderiales	Comamonadaceae	Hylemonella	Hylemonella gracilis
181	Bacteria	Proteobacteria	Alphaproteobacteria	Sphingomonadales	Sphingomonadaceae	Sphingorhabdus	Sphingorhabdus marina
180	Bacteria	Proteobacteria	Alphaproteobacteria	Sphingomonadales	Sphingomonadaceae	Novosphingobium	Novosphingobium sp. CF614
180	Bacteria	Nitrospirae	Nitrospira	Nitrospirales	Nitrospiraceae	Nitrospira	
179	Bacteria	Proteobacteria	Betaproteobacteria	Burkholderiales	Comamonadaceae	Limnohabitans	Limnohabitans sp. 103DPR2
179	Bacteria	Proteobacteria	Betaproteobacteria	Burkholderiales	Comamonadaceae	Curvibacter	Curvibacter sp. PAE-UM
176	Bacteria	Proteobacteria	Alphaproteobacteria	Sphingomonadales	unclassified Sphingomonadales	Sphingomonadales bacterium EhC05	
175	Bacteria	Terrabacteria group	Actinobacteria	Acidimicrobiia	Acidimicrobiales	Acidimicrobiaceae	Ilumatobacter
173	Bacteria	Proteobacteria	Betaproteobacteria	Burkholderiales	unclassified Burkholderiales	Burkholderiales Genera incertae sedis	Ideonella
173	Bacteria	FCB group	Bacteroidetes/Chlorobi group	Bacteroidetes	Bacteroidia	Bacteroidales	Porphyromonadaceae
171	Bacteria	Proteobacteria	Betaproteobacteria	Burkholderiales	Comamonadaceae	Rhodoferax	Rhodoferax ferrireducens
171	Bacteria	Proteobacteria	Betaproteobacteria	Burkholderiales	Comamonadaceae	Limnohabitans	Limnohabitans planktonicus
170	Bacteria	Terrabacteria group	Firmicutes				
170	Bacteria	Terrabacteria group	Actinobacteria	Actinobacteria	Streptomycetales	Streptomycetaceae	Streptomyces
170	Bacteria	Proteobacteria	Betaproteobacteria	Burkholderiales	Comamonadaceae	Hydrogenophaga	Hydrogenophaga sp. RAC07
170	Bacteria	Nitrospirae	Nitrospira	Nitrospirales	Nitrospiraceae	Nitrospira	Nitrospira sp. ST-bin5
169	Bacteria	Proteobacteria	Gammaproteobacteria	unclassified Gammaproteobacteria	sulfur-oxidizing symbionts	endosymbiont of Tevnia jerichonana	
164	Bacteria	Proteobacteria	Alphaproteobacteria	Parvularculales	Parvularculaceae	Parvularcula	Parvularcula oceani
163	Bacteria	Proteobacteria	Betaproteobacteria	Burkholderiales	Comamonadaceae	Limnohabitans	
161	Bacteria	Proteobacteria	Alphaproteobacteria	Sphingomonadales	Sphingomonadaceae	Sphingopyxis	Sphingopyxis baekryungensis
160	Bacteria	Proteobacteria	Betaproteobacteria	Burkholderiales	Comamonadaceae	Variovorax	Variovorax paradoxus
160	Bacteria	Proteobacteria	Betaproteobacteria	Burkholderiales	Comamonadaceae	Ramlibacter	Ramlibacter sp. Leaf400
159	Bacteria	Proteobacteria	Alphaproteobacteria	Rhodobacterales	Rhodobacteraceae	Defluviimonas	Defluviimonas alba
158	Eukaryota	Alveolata	Colponemidia	Colponema	Colponema vietnamica		
158	Bacteria	Proteobacteria	Alphaproteobacteria	Rhodobacterales	Rhodobacteraceae	unclassified Rhodobacteraceae	
157	Bacteria	Proteobacteria	Alphaproteobacteria	Rhodobacterales	Rhodobacteraceae	Paracoccus	
156	Bacteria	PVC group	Chlamydiae	Chlamydiia	Chlamydiales	Chlamydiaceae	Chlamydia/Chlamydophila group
155	Bacteria	Proteobacteria	Betaproteobacteria	Burkholderiales	unclassified Burkholderiales	Burkholderiales Genera incertae sedis	Leptothrix
154	Bacteria	Proteobacteria	Betaproteobacteria	Burkholderiales	Comamonadaceae	Limnohabitans	Limnohabitans sp. 63ED37-2
154	Bacteria	Proteobacteria	Betaproteobacteria	Burkholderiales	Comamonadaceae	Hydrogenophaga	Hydrogenophaga sp. IBVHS2
152	Eukaryota	Rhodophyta	Florideophyceae	Ceramiales	Rhodomelaceae	Vertebrata	Vertebrata lanosa
152	Bacteria	Proteobacteria	Betaproteobacteria	Burkholderiales	unclassified Burkholderiales	Burkholderiales Genera incertae sedis	Aquincola
151	Eukaryota	Amoebozoa	Mycetozoa	Myxogastria	Trichiida	Trichiidae	Arcyria
150	Eukaryota	Rhizaria	Cercozoa	Imbricatea	Silicofilosea	Euglyphida	Cyphoderiidae
148	Bacteria	Terrabacteria group	Actinobacteria	Actinobacteria	Corynebacteriales	Corynebacteriaceae	Corynebacterium
147	Bacteria	Proteobacteria	delta/epsilon subdivisions	Deltaproteobacteria	Desulfovibrionales	Desulfovibrionaceae	Desulfovibrio
146	Eukaryota	Opisthokonta	Fungi	Dikarya	Ascomycota	saccharomyceta	Pezizomycotina
146	Bacteria	Proteobacteria	Alphaproteobacteria	Sphingomonadales	Erythrobacteraceae		
145	Bacteria	Proteobacteria	delta/epsilon subdivisions	Deltaproteobacteria	Myxococcales	Sorangiineae	Sandaracinaceae
145	Bacteria	FCB group	Bacteroidetes/Chlorobi group	Bacteroidetes	Bacteroidia	Bacteroidales	Porphyromonadaceae
142	Bacteria	Proteobacteria	Alphaproteobacteria	Sphingomonadales	Erythrobacteraceae	Altererythrobacter	Altererythrobacter sp. Root672
142	Bacteria	Proteobacteria	Alphaproteobacteria	Sphingomonadales	Erythrobacteraceae	Altererythrobacter	Altererythrobacter dongtanensis
140	Bacteria	Proteobacteria	Betaproteobacteria	Burkholderiales	unclassified Burkholderiales	Burkholderiales Genera incertae sedis	Rhizobacter
140	Bacteria	Proteobacteria	Betaproteobacteria	Burkholderiales	Comamonadaceae	Ottowia	Ottowia thiooxydans
139	Bacteria	Terrabacteria group	Actinobacteria	Acidimicrobiia	Acidimicrobiales	Acidimicrobiaceae	Ilumatobacter
139	Bacteria	Proteobacteria	delta/epsilon subdivisions	Deltaproteobacteria	Desulfovibrionales	Desulfovibrionaceae	Desulfovibrio
138	Bacteria	Proteobacteria	Betaproteobacteria	unclassified Betaproteobacteria	unclassified Betaproteobacteria (miscellaneous)	Betaproteobacteria bacterium RIFCSPLOWO2_02_64_14	
138	Bacteria	Proteobacteria	Alphaproteobacteria	Sphingomonadales	Erythrobacteraceae	Erythrobacter	
137	Bacteria	Proteobacteria	Betaproteobacteria	Burkholderiales	Comamonadaceae	Polaromonas	Polaromonas sp. OV174
136	Eukaryota	Viridiplantae	Chlorophyta				
136	Bacteria	Proteobacteria	Gammaproteobacteria	unclassified Gammaproteobacteria	unclassified Gammaproteobacteria (miscellaneous)	Gammaproteobacteria bacterium 13_2_20CM_66_19	
136	Bacteria	Proteobacteria	Alphaproteobacteria	Sphingomonadales	Sphingomonadaceae	Novosphingobium	Novosphingobium tardaugens
136	Bacteria	Proteobacteria	Alphaproteobacteria	Rhodobacterales	Rhodobacteraceae	Gemmobacter	Gemmobacter nectariphilus
136	Bacteria	FCB group	Bacteroidetes/Chlorobi group	Bacteroidetes	Bacteroidia	Bacteroidales	Rikenellaceae
135	Eukaryota	Amoebozoa	Tubulinea	Euamoebida	Tubulinida	Hartmannellidae	Vermamoeba
135	Bacteria	Proteobacteria	Betaproteobacteria	Burkholderiales	Comamonadaceae	unclassified Comamonadaceae	Comamonadaceae bacterium URHA0028
133	Bacteria	Proteobacteria	Betaproteobacteria	Burkholderiales	Comamonadaceae	Pseudorhodoferax	Pseudorhodoferax sp. Leaf267
133	Bacteria	Acidobacteria	unclassified Acidobacteria	unclassified Acidobacteria (miscellaneous)	Acidobacteria bacterium SCN 69-37		
132	Bacteria	Proteobacteria	Betaproteobacteria	Burkholderiales	unclassified Burkholderiales	Burkholderiales Genera incertae sedis	Methylibium
132	Bacteria	Proteobacteria	Betaproteobacteria	Burkholderiales	Comamonadaceae	Curvibacter	Curvibacter delicatus
130	Bacteria	Proteobacteria	Alphaproteobacteria	Rhodobacterales	Rhodobacteraceae	Pseudorhodobacter	Pseudorhodobacter wandonensis
129	Eukaryota	Opisthokonta	Fungi	Dikarya	Ascomycota	saccharomyceta	Saccharomycotina
129	Bacteria	Proteobacteria	delta/epsilon subdivisions	Deltaproteobacteria	Desulfovibrionales	Desulfovibrionaceae	
129	Bacteria	Proteobacteria	Betaproteobacteria	Burkholderiales	Comamonadaceae	Pelomonas	
128	Bacteria	Terrabacteria group	Firmicutes	Clostridia	Clostridiales	Ruminococcaceae	Faecalibacterium
127	Bacteria	Terrabacteria group	Chloroflexi	unclassified Chloroflexi	unclassified Chloroflexi (miscellaneous)	Chloroflexi bacterium RBG_13_50_21	
127	Bacteria	Proteobacteria	Betaproteobacteria	Burkholderiales	Comamonadaceae	Polaromonas	Polaromonas jejuensis
127	Bacteria	Proteobacteria	Betaproteobacteria	Burkholderiales	Comamonadaceae	Caldimonas	
127	Bacteria	FCB group	Bacteroidetes/Chlorobi group	Bacteroidetes	Bacteroidia	Bacteroidales	Rikenellaceae
125	Bacteria	Proteobacteria	delta/epsilon subdivisions	Deltaproteobacteria	Myxococcales	Sorangiineae	Polyangiaceae
124	Bacteria	Proteobacteria	delta/epsilon subdivisions	Deltaproteobacteria	Desulfovibrionales	Desulfovibrionaceae	Bilophila
124	Bacteria	Proteobacteria	Alphaproteobacteria	Sphingomonadales	unclassified Sphingomonadales	Sphingomonadales bacterium BRH_c42	
124	Bacteria	Proteobacteria	Alphaproteobacteria	Sphingomonadales	Sphingomonadaceae	Sphingopyxis	Sphingopyxis macrogoltabida
123	Bacteria	Proteobacteria	Betaproteobacteria	Burkholderiales	Comamonadaceae	Polaromonas	Polaromonas naphthalenivorans
123	Bacteria	Proteobacteria	Betaproteobacteria	Burkholderiales	Comamonadaceae	Hydrogenophaga	Hydrogenophaga sp. T4
123	Bacteria	Proteobacteria	Alphaproteobacteria	Rhodobacterales	Rhodobacteraceae	Rhodobacter	Rhodobacter sphaeroides
122	Bacteria	Terrabacteria group	Actinobacteria	Actinobacteria	Streptomycetales	Streptomycetaceae	Streptomyces
122	Bacteria	Proteobacteria	Betaproteobacteria	unclassified Betaproteobacteria	unclassified Betaproteobacteria (miscellaneous)	beta proteobacterium AAP51	
122	Bacteria	Proteobacteria	Betaproteobacteria	Burkholderiales	Burkholderiaceae	Burkholderia	
121	Bacteria	Proteobacteria	Gammaproteobacteria	Enterobacterales	Morganellaceae	Providencia	Candidatus Providencia siddallii
121	Bacteria	Proteobacteria	Betaproteobacteria	unclassified Betaproteobacteria	unclassified Betaproteobacteria (miscellaneous)	Betaproteobacteria bacterium RIFCSPLOWO2_12_FULL_62_58	
121	Bacteria	Proteobacteria	Betaproteobacteria	Burkholderiales	unclassified Burkholderiales	[Polyangium] brachysporum	
121	Bacteria	Proteobacteria	Betaproteobacteria	Burkholderiales	unclassified Burkholderiales	Burkholderiales Genera incertae sedis	Sphaerotilus
121	Bacteria	FCB group	Bacteroidetes/Chlorobi group	Bacteroidetes	Bacteroidia	Bacteroidales	Rikenellaceae
120	Bacteria	PVC group	Planctomycetes	Planctomycetia	Planctomycetales	Planctomycetaceae	Fuerstia
119	Bacteria	FCB group	Bacteroidetes/Chlorobi group	Bacteroidetes	Bacteroidia	Bacteroidales	Porphyromonadaceae
118	Bacteria	Proteobacteria	Betaproteobacteria	Burkholderiales	Comamonadaceae	Rhodoferax	Rhodoferax fermentans
117	Bacteria	Proteobacteria	Betaproteobacteria	Burkholderiales	unclassified Burkholderiales	Burkholderiales Genera incertae sedis	Ideonella
116	Bacteria	Proteobacteria	Gammaproteobacteria	Alteromonadales	Shewanellaceae	Shewanella	
116	Bacteria	Proteobacteria	Betaproteobacteria	Burkholderiales	unclassified Burkholderiales	Burkholderiales Genera incertae sedis	Rubrivivax
116	Bacteria	Proteobacteria	Betaproteobacteria	Burkholderiales	unclassified Burkholderiales		
115	Bacteria	Proteobacteria	Betaproteobacteria	Burkholderiales	unclassified Burkholderiales	unclassified Burkholderiales (miscellaneous)	Burkholderiales bacterium RIFOXYC12_FULL_65_23
115	Bacteria	Proteobacteria	Betaproteobacteria	Burkholderiales	unclassified Burkholderiales	Burkholderiales Genera incertae sedis	Xylophilus
114	Bacteria	Proteobacteria	Betaproteobacteria	Burkholderiales	Comamonadaceae	Limnohabitans	Limnohabitans sp. Rim47
113	Bacteria	Proteobacteria	Betaproteobacteria	Burkholderiales	Burkholderiaceae	Cupriavidus	
113	Bacteria	Proteobacteria	Alphaproteobacteria	Sphingomonadales	Erythrobacteraceae	Altererythrobacter	Altererythrobacter sp. MH-B5
112	Bacteria	Proteobacteria	delta/epsilon subdivisions	Deltaproteobacteria	unclassified Deltaproteobacteria	Candidatus Adiutrix	Candidatus Adiutrix intracellularis
112	Bacteria	Proteobacteria	Alphaproteobacteria	Rhizobiales	Rhizobiaceae	Rhizobium/Agrobacterium group	
112	Bacteria	Acidobacteria	Holophagae	Holophagales	Holophagaceae	Geothrix	Geothrix fermentans
111	Bacteria	Proteobacteria	Betaproteobacteria	Burkholderiales	Comamonadaceae	Curvibacter	Curvibacter sp. GWA2_64_110
110	Eukaryota	Stramenopiles	Oomycetes	Peronosporales	Peronosporaceae	Plasmopara	Plasmopara euphrasiae
110	Eukaryota	Malawimonadidae	Malawimonas	Malawimonas sp. strain 249			
110	Bacteria	Proteobacteria	Betaproteobacteria	Burkholderiales	unclassified Burkholderiales	Burkholderiales Genera incertae sedis	Aquabacterium
109	Bacteria	Proteobacteria	delta/epsilon subdivisions	Deltaproteobacteria	Desulfovibrionales	Desulfovibrionaceae	Desulfovibrio
109	Bacteria	Proteobacteria	Alphaproteobacteria	Sphingomonadales	Sphingomonadaceae	Novosphingobium	Novosphingobium sp. ST904
109	Bacteria	Proteobacteria	Alphaproteobacteria	Sphingomonadales	Erythrobacteraceae	Altererythrobacter	Altererythrobacter atlanticus
109	Bacteria	Proteobacteria	Alphaproteobacteria	Rhodobacterales	Rhodobacteraceae	Pseudorhodobacter	Pseudorhodobacter antarcticus
108	Bacteria	Proteobacteria	Alphaproteobacteria	Sphingomonadales	Sphingomonadaceae	Sphingomonas	Sphingomonas changbaiensis
108	Bacteria	Proteobacteria	Alphaproteobacteria	Sphingomonadales	Erythrobacteraceae	Erythrobacter	Erythrobacter sp. SG61-1L
108	Bacteria	Proteobacteria	Alphaproteobacteria	Rhizobiales	Methylobacteriaceae	Methylobacterium	
107	Eukaryota	Viridiplantae	Chlorophyta	Trebouxiophyceae	Microthamniales	Asterochloris	Asterochloris sp. DA2
107	Bacteria	Proteobacteria	Betaproteobacteria	Hydrogenophilales	Hydrogenophilaceae	Thiobacillus	
106	Bacteria	Terrabacteria group	Firmicutes	Bacilli	Lactobacillales	Enterococcaceae	Enterococcus
106	Bacteria	Proteobacteria	Alphaproteobacteria	Sphingomonadales	Sphingomonadaceae	Novosphingobium	Novosphingobium rosa
106	Bacteria	Proteobacteria	Alphaproteobacteria	Sphingomonadales	Erythrobacteraceae	Erythrobacter	Erythrobacter luteus
105	Bacteria	Terrabacteria group	Actinobacteria	Actinobacteria	Corynebacteriales	Mycobacteriaceae	Mycobacterium
105	Bacteria	Proteobacteria	Gammaproteobacteria	Xanthomonadales	Xanthomonadaceae	Arenimonas	Arenimonas malthae
105	Bacteria	Proteobacteria	Betaproteobacteria	unclassified Betaproteobacteria	unclassified Betaproteobacteria (miscellaneous)	Betaproteobacteria bacterium RIFCSPLOWO2_12_FULL_62_13	
105	Bacteria	Proteobacteria	Alphaproteobacteria	Rickettsiales	Candidatus Midichloriaceae	Candidatus Midichloria	Candidatus Midichloria mitochondrii
105	Bacteria	PVC group	Planctomycetes	Planctomycetia	Planctomycetales	Gemmataceae	Gemmata
104	Bacteria	Terrabacteria group	Actinobacteria	Actinobacteria			
104	Bacteria	Proteobacteria	Betaproteobacteria	Burkholderiales	Comamonadaceae	Polaromonas	Polaromonas sp. YR568
104	Bacteria	Proteobacteria	Alphaproteobacteria	Sphingomonadales	Sphingomonadaceae	Sphingomonas	Sphingomonas sanxanigenens
104	Bacteria	Proteobacteria	Alphaproteobacteria	Sphingomonadales	Erythrobacteraceae	Erythrobacter	Erythrobacter litoralis
104	Bacteria	Proteobacteria	Alphaproteobacteria	Rhodobacterales	Rhodobacteraceae	Pseudorhodobacter	Pseudorhodobacter ferrugineus
102	Bacteria	Proteobacteria	Betaproteobacteria	Burkholderiales	unclassified Burkholderiales	unclassified Burkholderiales (miscellaneous)	Burkholderiales bacterium 68-10
102	Bacteria	Proteobacteria	Betaproteobacteria	Burkholderiales	Comamonadaceae	Polaromonas	Polaromonas glacialis
102	Bacteria	Proteobacteria	Betaproteobacteria	Burkholderiales	Comamonadaceae	Limnohabitans	Limnohabitans sp. Rim28
101	Bacteria	Proteobacteria	Betaproteobacteria	Burkholderiales	unclassified Burkholderiales	Burkholderiales Genera incertae sedis	Paucibacter
100	Bacteria	Proteobacteria	Betaproteobacteria	unclassified Betaproteobacteria	unclassified Betaproteobacteria (miscellaneous)	Betaproteobacteria bacterium RIFCSPLOWO2_02_FULL_67_26	
100	Bacteria	Proteobacteria	Betaproteobacteria	Burkholderiales	Comamonadaceae	Rhodoferax	Rhodoferax antarcticus
100	Bacteria	Proteobacteria	Betaproteobacteria	Burkholderiales	Comamonadaceae	Pelomonas	Pelomonas sp. KK5
100	Bacteria	Proteobacteria	Alphaproteobacteria	Rhodobacterales	Rhodobacteraceae	Rhodobacter	Rhodobacter capsulatus
100	Bacteria	Deferribacteres	Deferribacteres	Deferribacterales	Deferribacteraceae	Mucispirillum	Mucispirillum schaedleri
99	Bacteria	Proteobacteria	Gammaproteobacteria	unclassified Gammaproteobacteria	unclassified Gammaproteobacteria (miscellaneous)	Gammaproteobacteria bacterium SG8_30	
99	Bacteria	Proteobacteria	Alphaproteobacteria	Sphingomonadales	Sphingomonadaceae	Novosphingobium	Novosphingobium sp. AP12
98	Bacteria	Proteobacteria	Betaproteobacteria	unclassified Betaproteobacteria	unclassified Betaproteobacteria (miscellaneous)	Betaproteobacteria bacterium GR16-43	
98	Bacteria	Proteobacteria	Betaproteobacteria	Burkholderiales	unclassified Burkholderiales	Burkholderiales Genera incertae sedis	Aquabacterium
97	Bacteria	Proteobacteria	Alphaproteobacteria	Sphingomonadales	Sphingomonadaceae	Novosphingobium	Novosphingobium fuchskuhlense
97	Bacteria	FCB group	Bacteroidetes/Chlorobi group	Bacteroidetes	Bacteroidia	Bacteroidales	Rikenellaceae
96	Eukaryota	Opisthokonta	Fungi	Dikarya	Ascomycota	saccharomyceta	Pezizomycotina
94	Bacteria	Proteobacteria	Alphaproteobacteria	Sphingomonadales	Sphingomonadaceae	Sphingobium	Sphingobium sp. SYK-6
93	Bacteria	FCB group	Bacteroidetes/Chlorobi group	Bacteroidetes	Bacteroidia	Bacteroidales	Porphyromonadaceae
92	Eukaryota	Rhodophyta	Florideophyceae	Halymeniales	Halymeniaceae	Pachymeniopsis	Pachymeniopsis gargiuli
92	Bacteria	Acidobacteria	unclassified Acidobacteria	unclassified Acidobacteria (miscellaneous)	Acidobacteria bacterium RIFCSPLOWO2_02_FULL_65_29		
91	Bacteria	Proteobacteria	Betaproteobacteria	Burkholderiales	Comamonadaceae	Variovorax	Variovorax soli
91	Bacteria	Proteobacteria	Alphaproteobacteria	Rhodobacterales	Rhodobacteraceae	Thioclava	
90	Bacteria	Proteobacteria	Betaproteobacteria	Burkholderiales	Comamonadaceae	Polaromonas	Polaromonas sp. EUR3 1.2.1
90	Bacteria	Proteobacteria	Alphaproteobacteria	Sphingomonadales	unclassified Sphingomonadales	Sphingomonadales bacterium 63-6	
90	Bacteria	Proteobacteria	Alphaproteobacteria	Sphingomonadales	Erythrobacteraceae	Erythrobacter	Erythrobacter marinus
89	Bacteria	Proteobacteria	Betaproteobacteria	Burkholderiales	Alcaligenaceae	Azohydromonas	Azohydromonas australica
89	Bacteria	PVC group	Planctomycetes	Planctomycetia	Planctomycetales	Gemmataceae	Gemmata
88	Bacteria	Proteobacteria	Betaproteobacteria	Rhodocyclales	Rhodocyclaceae	Sulfuritalea	Sulfuritalea hydrogenivorans
88	Bacteria	Proteobacteria	Betaproteobacteria	Burkholderiales	Comamonadaceae	unclassified Comamonadaceae	Comamonadaceae bacterium CG2_30_59_20
88	Bacteria	Proteobacteria	Betaproteobacteria	Burkholderiales	Comamonadaceae	Rhodoferax	
88	Bacteria	Proteobacteria	Alphaproteobacteria	Sphingomonadales	Sphingomonadaceae	Sphingomonas	Sphingomonas sp. SCN 67-18
87	Bacteria	Proteobacteria	delta/epsilon subdivisions	Deltaproteobacteria	unclassified Deltaproteobacteria	unclassified Deltaproteobacteria (miscellaneous)	
87	Bacteria	Proteobacteria	Gammaproteobacteria	Nevskiales	Sinobacteraceae	Steroidobacter	Steroidobacter denitrificans
87	Bacteria	Proteobacteria	Betaproteobacteria	Burkholderiales	unclassified Burkholderiales	Burkholderiales Genera incertae sedis	Rubrivivax
87	Bacteria	Proteobacteria	Alphaproteobacteria	Sphingomonadales	Erythrobacteraceae	Altererythrobacter	Altererythrobacter sp. 66-12
87	Bacteria	Acidobacteria	unclassified Acidobacteria	Acidobacteria subdivision 6	Acidobacteria bacterium DSM 100886		
86	Bacteria	Proteobacteria	Alphaproteobacteria	Sphingomonadales	Sphingomonadaceae	Novosphingobium	Novosphingobium sp. MBES04
86	Bacteria	Proteobacteria	Alphaproteobacteria	Sphingomonadales	Sphingomonadaceae	Novosphingobium	Novosphingobium naphthalenivorans
85	Bacteria	Terrabacteria group	Firmicutes	Clostridia	Clostridiales	Clostridiales incertae sedis	Clostridiales Family XVII. Incertae Sedis
85	Bacteria	Proteobacteria	Betaproteobacteria	unclassified Betaproteobacteria	unclassified Betaproteobacteria (miscellaneous)	Betaproteobacteria bacterium MOLA814	
85	Bacteria	Proteobacteria	Alphaproteobacteria	Rhizobiales	Hyphomicrobiaceae	Hyphomicrobium	Hyphomicrobium sulfonivorans
84	Bacteria	Proteobacteria	Alphaproteobacteria	Sphingomonadales	Sphingomonadaceae	Novosphingobium	Novosphingobium sp. Fuku2-ISO-50
84	Bacteria	Proteobacteria	Alphaproteobacteria	Rhodobacterales	Rhodobacteraceae	Gemmobacter	Gemmobacter megaterium
84	Bacteria	PVC group	Verrucomicrobia	Opitutae	Opitutales	Opitutaceae	Opitutus
83	Bacteria	Proteobacteria	Alphaproteobacteria	Sphingomonadales	Sphingomonadaceae	Sphingomonas	Sphingomonas sp. KC8
82	Bacteria	Proteobacteria	Betaproteobacteria	Methylophilales	Methylophilaceae	Methylotenera	Methylotenera mobilis
82	Bacteria	Proteobacteria	Betaproteobacteria	Methylophilales	Methylophilaceae	Methylophilus	
82	Bacteria	Proteobacteria	Betaproteobacteria	Burkholderiales	Oxalobacteraceae		
82	Bacteria	Proteobacteria	Alphaproteobacteria	Sphingomonadales	Sphingomonadaceae	Sphingomonas	Sphingomonas sp. 35-24ZXX
82	Bacteria	Proteobacteria	Alphaproteobacteria	Sphingomonadales	Sphingomonadaceae	Sphingobium	Sphingobium phenoxybenzoativorans
82	Bacteria	FCB group	Bacteroidetes/Chlorobi group	Bacteroidetes	Bacteroidia	Bacteroidales	Prevotellaceae
80	Eukaryota	Opisthokonta	Fungi	Blastocladiomycota	Blastocladiomycetes	Blastocladiales	Blastocladiaceae
80	Bacteria	unclassified Bacteria	Bacteria candidate phyla	Candidatus Rokubacteria			
80	Bacteria	Proteobacteria	Gammaproteobacteria	Xanthomonadales	Xanthomonadaceae		
80	Bacteria	Proteobacteria	Alphaproteobacteria	Sphingomonadales	Sphingomonadaceae	Sphingomonas	Sphingomonas sp. 67-36
80	Bacteria	Proteobacteria	Alphaproteobacteria	Sphingomonadales	Sphingomonadaceae	Blastomonas	
80	Bacteria	Proteobacteria	Alphaproteobacteria	Rhizobiales	Bradyrhizobiaceae		
80	Bacteria	FCB group	Bacteroidetes/Chlorobi group	Bacteroidetes	Bacteroidia	Bacteroidales	Prevotellaceae
79	Bacteria	Proteobacteria	Betaproteobacteria	unclassified Betaproteobacteria	unclassified Betaproteobacteria (miscellaneous)	Betaproteobacteria bacterium SG8_40	
79	Bacteria	Proteobacteria	Betaproteobacteria	Burkholderiales	Comamonadaceae	unclassified Comamonadaceae	Comamonadaceae bacterium CG1_02_60_18
79	Bacteria	Proteobacteria	Betaproteobacteria	Burkholderiales	Alcaligenaceae	Azohydromonas	Azohydromonas lata
79	Bacteria	Proteobacteria	Alphaproteobacteria	Sphingomonadales	Erythrobacteraceae	Altererythrobacter	Altererythrobacter marensis
79	Bacteria	Proteobacteria	Alphaproteobacteria	Rhodospirillales	unclassified Rhodospirillales	unclassified Rhodospirillales (miscellaneous)	Rhodospirillales bacterium URHD0017
79	Bacteria	Proteobacteria	Alphaproteobacteria	Rhodospirillales	Rhodospirillaceae	Dongia	Dongia sp. URHE0060
79	Bacteria	PVC group	Verrucomicrobia	Opitutae	Opitutales	Opitutaceae	Lacunisphaera
79	Bacteria	PVC group	Lentisphaerae	unclassified Lentisphaerae	unclassified Lentisphaerae (miscellaneous)		
78	Bacteria	Proteobacteria	delta/epsilon subdivisions	Deltaproteobacteria	unclassified Deltaproteobacteria	unclassified Deltaproteobacteria (miscellaneous)	Deltaproteobacteria bacterium GWA2_55_10
78	Bacteria	Proteobacteria	Betaproteobacteria	unclassified Betaproteobacteria	unclassified Betaproteobacteria (miscellaneous)	beta proteobacterium AAP99	
78	Bacteria	Proteobacteria	Betaproteobacteria	Burkholderiales	Comamonadaceae	Variovorax	Variovorax sp. OV329
78	Bacteria	Proteobacteria	Betaproteobacteria	Burkholderiales	Comamonadaceae	Comamonas	
78	Bacteria	Proteobacteria	Alphaproteobacteria	Sphingomonadales	Sphingomonadaceae	Novosphingobium	Novosphingobium sp. Rr 2-17
78	Bacteria	Proteobacteria	Alphaproteobacteria	Sphingomonadales	Sphingomonadaceae	Novosphingobium	Novosphingobium sp. B1
78	Bacteria	Proteobacteria	Alphaproteobacteria	Sphingomonadales	Sphingomonadaceae	Blastomonas	Blastomonas sp. RAC04
78	Bacteria	Proteobacteria	Alphaproteobacteria	Rhodobacterales	Rhodobacteraceae	Rhodobacter	Rhodobacter sp. CCB-MM2
77	Eukaryota	Stramenopiles	Bacillariophyta	Bacillariophyceae	Bacillariophycidae	Naviculales	Naviculaceae
77	Eukaryota	Opisthokonta	Fungi	Dikarya	Basidiomycota	Pucciniomycotina	Microbotryomycetes
77	Bacteria	Terrabacteria group	Firmicutes	Bacilli	Bacillales	Paenibacillaceae	Paenibacillus
77	Bacteria	Proteobacteria	delta/epsilon subdivisions	Deltaproteobacteria	Desulfovibrionales	Desulfovibrionaceae	Bilophila
77	Bacteria	Proteobacteria	Gammaproteobacteria	Xanthomonadales			
77	Bacteria	Proteobacteria	Betaproteobacteria	Burkholderiales	Comamonadaceae	unclassified Comamonadaceae	Comamonadaceae bacterium CG2_30_57_122
77	Bacteria	Proteobacteria	Betaproteobacteria	Burkholderiales	Comamonadaceae	Polaromonas	Polaromonas sp. CG9_12
76	Eukaryota	Haptophyceae	Phaeocystales	Phaeocystaceae	Phaeocystis		
76	Bacteria	Proteobacteria	Betaproteobacteria	Burkholderiales	unclassified Burkholderiales	unclassified Burkholderiales (miscellaneous)	Burkholderiales bacterium RIFCSPHIGHO2_12_FULL_61_11
76	Bacteria	Proteobacteria	Betaproteobacteria	Burkholderiales	Comamonadaceae	Variovorax	Variovorax sp. CF079
76	Bacteria	Proteobacteria	Alphaproteobacteria	Rhodobacterales	Rhodobacteraceae	unclassified Rhodobacteraceae	Rhodobacteraceae bacterium EhC02
76	Bacteria	Proteobacteria	Alphaproteobacteria	Rhodobacterales	Rhodobacteraceae	unclassified Rhodobacteraceae	Rhodobacteraceae bacterium CG2_30_10_405
75	Eukaryota	Opisthokonta	Fungi	Dikarya	Basidiomycota	Agaricomycotina	Agaricomycetes
75	Bacteria	Proteobacteria	Betaproteobacteria	Methylophilales	Methylophilaceae	Methylotenera	Methylotenera sp. RIFCSPLOWO2_02_FULL_45_14
75	Bacteria	Proteobacteria	Betaproteobacteria	Burkholderiales	unclassified Burkholderiales	Burkholderiales Genera incertae sedis	Tepidimonas
75	Bacteria	Proteobacteria	Alphaproteobacteria	Sphingomonadales	Sphingomonadaceae	Sphingopyxis	Sphingopyxis sp. H050
75	Bacteria	Proteobacteria	Alphaproteobacteria	Sphingomonadales	Sphingomonadaceae	Novosphingobium	Novosphingobium acidiphilum
74	Bacteria	Proteobacteria	Alphaproteobacteria	Sphingomonadales	Erythrobacteraceae	Altererythrobacter	Altererythrobacter troitsensis
74	Bacteria	Proteobacteria	Alphaproteobacteria	Rhodobacterales	Rhodobacteraceae	Rubellimicrobium	Rubellimicrobium mesophilum
74	Bacteria	Proteobacteria	Alphaproteobacteria	Rhodobacterales	Rhodobacteraceae	Defluviimonas	
74	Bacteria	FCB group	Gemmatimonadetes	unclassified Gemmatimonadetes	Gemmatimonadetes bacterium SCN 70-22		
74	Bacteria	FCB group	Bacteroidetes/Chlorobi group	Bacteroidetes	Bacteroidia	Bacteroidales	Porphyromonadaceae
73	Bacteria	Proteobacteria	delta/epsilon subdivisions	Deltaproteobacteria	Myxococcales	unclassified Myxococcales	unclassified Myxococcales (miscellaneous)
73	Bacteria	Proteobacteria	Betaproteobacteria	Methylophilales	Methylophilaceae	Methylotenera	Methylotenera sp. G11
73	Bacteria	Proteobacteria	Betaproteobacteria	Burkholderiales	unclassified Burkholderiales	Burkholderiales Genera incertae sedis	Rhizobacter
73	Bacteria	Proteobacteria	Betaproteobacteria	Burkholderiales	Comamonadaceae	Variovorax	Variovorax sp. PAMC 28711
73	Bacteria	Proteobacteria	Alphaproteobacteria	Sphingomonadales	Erythrobacteraceae	Altererythrobacter	Altererythrobacter namhicola
72	Bacteria	Proteobacteria	Betaproteobacteria	Burkholderiales	Alcaligenaceae	Achromobacter	
72	Bacteria	PVC group	Planctomycetes	Planctomycetia	Planctomycetales	Planctomycetaceae	Schlesneria
72	Bacteria	Acidobacteria	unclassified Acidobacteria	unclassified Acidobacteria (miscellaneous)	Acidobacteria bacterium OLB17		
71	Bacteria	Proteobacteria	Alphaproteobacteria	Rhodobacterales	Rhodobacteraceae	Wenxinia	Wenxinia marina
71	Bacteria	PVC group	Planctomycetes	Planctomycetia	Planctomycetales	Planctomycetaceae	Planctomyces
70	Bacteria	Proteobacteria	Betaproteobacteria	Burkholderiales	Comamonadaceae	Variovorax	Variovorax sp. KK3
70	Bacteria	Proteobacteria	Alphaproteobacteria	Sphingomonadales	Sphingomonadaceae	Sphingomonas	Sphingomonas jaspsi
70	Bacteria	Proteobacteria	Alphaproteobacteria	Sphingomonadales	Sphingomonadaceae	Novosphingobium	Novosphingobium sp. FSW06-99
70	Bacteria	Proteobacteria	Alphaproteobacteria	Rickettsiales	Anaplasmataceae	Wolbachieae	Wolbachia
70	Bacteria	Proteobacteria	Alphaproteobacteria	Rhodobacterales	Hyphomonadaceae		
70	Bacteria	FCB group	Bacteroidetes/Chlorobi group	Bacteroidetes	Bacteroidia	Bacteroidales	Porphyromonadaceae
69	Bacteria	Terrabacteria group	Actinobacteria	Actinobacteria	unclassified Actinobacteria (class)	unclassified Actinobacteria (class) (miscellaneous)	actinobacterium acAcidi
69	Bacteria	Proteobacteria	Betaproteobacteria	Burkholderiales	Comamonadaceae	Xenophilus	Xenophilus azovorans
69	Bacteria	Proteobacteria	Alphaproteobacteria	Sphingomonadales	Sphingomonadaceae	Novosphingobium	Novosphingobium nitrogenifigens
69	Bacteria	Proteobacteria	Alphaproteobacteria	Rhodobacterales	Rhodobacteraceae	Sulfitobacter	
69	Bacteria	Proteobacteria	Alphaproteobacteria	Rhodobacterales	Rhodobacteraceae	Rubellimicrobium	Rubellimicrobium thermophilum
69	Bacteria	Proteobacteria	Alphaproteobacteria	Rhizobiales	Xanthobacteraceae	Labrys	Labrys sp. WJW
69	Bacteria	PVC group	Planctomycetes	Planctomycetia	Planctomycetales	Gemmataceae	Zavarzinella
68	Bacteria	Proteobacteria	Betaproteobacteria	Burkholderiales	Comamonadaceae	Pseudorhodoferax	Pseudorhodoferax sp. Leaf265
68	Bacteria	Proteobacteria	Alphaproteobacteria	Sphingomonadales	Sphingomonadaceae	Novosphingobium	Novosphingobium lindaniclasticum
68	Bacteria	Proteobacteria	Alphaproteobacteria	Sphingomonadales	Erythrobacteraceae	Erythrobacter	Erythrobacter sp. JL475
68	Bacteria	Proteobacteria	Alphaproteobacteria	Rhizobiales	Bradyrhizobiaceae	Bradyrhizobium	Bradyrhizobium erythrophlei
68	Bacteria	PVC group	Verrucomicrobia	Verrucomicrobiae	Verrucomicrobiales	Verrucomicrobia subdivision 3	Pedosphaera
67	Eukaryota	Opisthokonta	Fungi	Dikarya	Basidiomycota	Ustilaginomycotina	Ustilaginomycetes
67	Bacteria	Proteobacteria	Gammaproteobacteria	Enterobacterales	Enterobacteriaceae		
67	Bacteria	Proteobacteria	Betaproteobacteria	Burkholderiales	Comamonadaceae	Verminephrobacter	Verminephrobacter eiseniae
67	Bacteria	Proteobacteria	Alphaproteobacteria	Rhodobacterales	Rhodobacteraceae	Loktanella	
66	Bacteria	Proteobacteria	delta/epsilon subdivisions	Deltaproteobacteria	Desulfovibrionales	Desulfovibrionaceae	Mailhella
66	Bacteria	Proteobacteria	Betaproteobacteria	unclassified Betaproteobacteria	unclassified Betaproteobacteria (miscellaneous)	Betaproteobacteria bacterium SG8_41	
66	Bacteria	Proteobacteria	Betaproteobacteria	Burkholderiales	unclassified Burkholderiales	Burkholderiales Genera incertae sedis	Rhizobacter
66	Bacteria	Proteobacteria	Alphaproteobacteria	Sphingomonadales	unclassified Sphingomonadales	Sphingomonadales bacterium BRH_c3	
66	Bacteria	Proteobacteria	Alphaproteobacteria	Sphingomonadales	Sphingomonadaceae	Sphingobium	Sphingobium sp. 66-54
66	Bacteria	Proteobacteria	Alphaproteobacteria	Rhodobacterales	Rhodobacteraceae	Roseovarius	
65	Bacteria	Terrabacteria group					
65	Bacteria	Proteobacteria	Gammaproteobacteria	Pseudomonadales	Pseudomonadaceae	Pseudomonas	Pseudomonas aeruginosa group
65	Bacteria	Proteobacteria	Betaproteobacteria	Burkholderiales	Comamonadaceae	Variovorax	Variovorax sp. WDL1
65	Bacteria	Proteobacteria	Betaproteobacteria	Burkholderiales	Comamonadaceae	Giesbergeria	Giesbergeria anulus
65	Bacteria	Proteobacteria	Alphaproteobacteria	Sphingomonadales	Sphingomonadaceae	Sphingomonas	Sphingomonas sp. JJ-A5
65	Bacteria	Proteobacteria	Alphaproteobacteria	Sphingomonadales	Erythrobacteraceae	Croceicoccus	Croceicoccus naphthovorans
65	Bacteria	Proteobacteria	Alphaproteobacteria	Sphingomonadales	Erythrobacteraceae	Altererythrobacter	Altererythrobacter epoxidivorans
65	Bacteria	Proteobacteria	Alphaproteobacteria	Rhodobacterales	Rhodobacteraceae	unclassified Rhodobacteraceae	Rhodobacteraceae bacterium HLUCCA12
64	Bacteria	Terrabacteria group	Firmicutes	Bacilli	Bacillales	Bacillaceae	Bacillus
64	Bacteria	Proteobacteria	Gammaproteobacteria	Enterobacterales			
64	Bacteria	Proteobacteria	Betaproteobacteria	Burkholderiales	Comamonadaceae	Simplicispira	Simplicispira psychrophila
64	Bacteria	Proteobacteria	Betaproteobacteria	Burkholderiales	Comamonadaceae	Delftia	
64	Bacteria	Proteobacteria	Alphaproteobacteria	Sphingomonadales	Sphingomonadaceae	Sphingopyxis	Sphingopyxis sp. MWB1
64	Bacteria	Proteobacteria	Alphaproteobacteria	Sphingomonadales	Sphingomonadaceae	Sphingobium	Sphingobium xenophagum
64	Bacteria	PVC group	Planctomycetes	Planctomycetia	Planctomycetales	Planctomycetaceae	Pirellula
63	Bacteria	Terrabacteria group	Firmicutes	Clostridia	Clostridiales	Clostridiaceae	Clostridium
63	Bacteria	Proteobacteria	delta/epsilon subdivisions	Deltaproteobacteria	Myxococcales	Sorangiineae	Labilitrichaceae
63	Bacteria	Proteobacteria	Gammaproteobacteria	Pseudomonadales	Moraxellaceae	Acinetobacter	
63	Bacteria	Proteobacteria	Betaproteobacteria	Burkholderiales	Comamonadaceae	Pseudorhodoferax	
63	Bacteria	Proteobacteria	Alphaproteobacteria	unclassified Alphaproteobacteria	Thermopetrobacter	Thermopetrobacter sp. TC1	
63	Bacteria	Proteobacteria	Alphaproteobacteria	Sphingomonadales	Sphingomonadaceae	Novosphingobium	Novosphingobium sp. Leaf2
63	Bacteria	Proteobacteria	Alphaproteobacteria	Sphingomonadales	Sphingomonadaceae	Blastomonas	Blastomonas sp. AAP53
63	Bacteria	Proteobacteria	Alphaproteobacteria	Sphingomonadales	Erythrobacteraceae	Erythrobacter	Erythrobacter gangjinensis
63	Bacteria	Proteobacteria	Alphaproteobacteria	Rhodobacterales	Rhodobacteraceae	Loktanella	Loktanella vestfoldensis
63	Bacteria	Proteobacteria	Alphaproteobacteria	Rhizobiales	Hyphomicrobiaceae	Hyphomicrobium	Hyphomicrobium sp. NDB2Meth4
63	Bacteria	Proteobacteria	Alphaproteobacteria	Rhizobiales	Bradyrhizobiaceae	Rhodopseudomonas	Rhodopseudomonas palustris
63	Bacteria	FCB group	Bacteroidetes/Chlorobi group	Bacteroidetes	Bacteroidia	Bacteroidales	Porphyromonadaceae
62	Bacteria	Proteobacteria	delta/epsilon subdivisions	Deltaproteobacteria	Desulfovibrionales	Desulfovibrionaceae	Bilophila
62	Bacteria	Proteobacteria	Betaproteobacteria	Rhodocyclales	Rhodocyclaceae	Thauera	
62	Bacteria	Proteobacteria	Betaproteobacteria	Burkholderiales	unclassified Burkholderiales	unclassified Burkholderiales (miscellaneous)	Burkholderiales bacterium RIFCSPHIGHO2_01_FULL_63_240
62	Bacteria	Proteobacteria	Betaproteobacteria	Burkholderiales	Comamonadaceae	Alicycliphilus	Alicycliphilus sp. EPL6
62	Bacteria	Proteobacteria	Alphaproteobacteria	Sphingomonadales	Erythrobacteraceae	Erythrobacter	Erythrobacter atlanticus
62	Bacteria	Proteobacteria	Alphaproteobacteria	Sphingomonadales	Erythrobacteraceae	Croceicoccus	Croceicoccus marinus
61	Bacteria	Proteobacteria	delta/epsilon subdivisions	Deltaproteobacteria	Desulfovibrionales	Desulfovibrionaceae	Desulfovibrio
61	Bacteria	Proteobacteria	Gammaproteobacteria	Vibrionales	Vibrionaceae	Vibrio	
61	Bacteria	Proteobacteria	Betaproteobacteria	Rhodocyclales	Rhodocyclaceae	Dechloromonas	Dechloromonas denitrificans
61	Bacteria	Proteobacteria	Betaproteobacteria	Burkholderiales	unclassified Burkholderiales	Burkholderiales Genera incertae sedis	Xylophilus
61	Bacteria	Proteobacteria	Betaproteobacteria	Burkholderiales	unclassified Burkholderiales	Burkholderiales Genera incertae sedis	Rubrivivax
61	Bacteria	Proteobacteria	Betaproteobacteria	Burkholderiales	Oxalobacteraceae	Massilia	
61	Bacteria	Proteobacteria	Betaproteobacteria	Burkholderiales	Comamonadaceae	Variovorax	Variovorax sp. URHB0020
61	Bacteria	Proteobacteria	Alphaproteobacteria	Sphingomonadales	Sphingomonadaceae	Sphingopyxis	Sphingopyxis alaskensis
61	Bacteria	Proteobacteria	Alphaproteobacteria	Sphingomonadales	Sphingomonadaceae	Sphingomonas	Sphingomonas indica
61	Bacteria	Proteobacteria	Alphaproteobacteria	Sphingomonadales	Erythrobacteraceae	Croceicoccus	Croceicoccus sp. H4
61	Bacteria	PVC group	Verrucomicrobia	Verrucomicrobiae	Verrucomicrobiales	Verrucomicrobiaceae	Haloferula
60	Bacteria	Proteobacteria	Gammaproteobacteria	Enterobacterales	Enterobacteriaceae	Salmonella	Salmonella enterica
60	Bacteria	Proteobacteria	Gammaproteobacteria	Chromatiales	unclassified Chromatiales	Chromatiales bacterium (ex Bugula neritina AB1)	
60	Bacteria	Proteobacteria	Betaproteobacteria	Burkholderiales	Comamonadaceae	Verminephrobacter	Verminephrobacter aporrectodeae
60	Bacteria	Proteobacteria	Betaproteobacteria	Burkholderiales	Comamonadaceae	Pseudacidovorax	Pseudacidovorax intermedius
60	Bacteria	Proteobacteria	Betaproteobacteria	Burkholderiales	Comamonadaceae	Comamonas	Comamonas testosteroni
60	Bacteria	Proteobacteria	Betaproteobacteria	Burkholderiales	Comamonadaceae	Alicycliphilus	
60	Bacteria	Proteobacteria	Alphaproteobacteria	Sphingomonadales	Sphingomonadaceae	Sphingomonas	Sphingomonas wittichii
60	Bacteria	Proteobacteria	Alphaproteobacteria	Rhodobacterales	Rhodobacteraceae	Rhodobacter	Rhodobacter vinaykumarii
59	Eukaryota	Opisthokonta	Fungi	Dikarya	Ascomycota	saccharomyceta	Pezizomycotina
59	Bacteria	Proteobacteria	delta/epsilon subdivisions	Deltaproteobacteria	Myxococcales	Sorangiineae	unclassified Sorangiineae
59	Bacteria	Proteobacteria	Zetaproteobacteria	Mariprofundales	Mariprofundaceae	Mariprofundus	Mariprofundus ferrooxydans
59	Bacteria	Proteobacteria	Betaproteobacteria	Hydrogenophilales	Hydrogenophilaceae	Thiobacillus	Thiobacillus denitrificans
59	Bacteria	Proteobacteria	Betaproteobacteria	Burkholderiales	Oxalobacteraceae	Oxalobacter	Oxalobacter formigenes
59	Bacteria	Proteobacteria	Betaproteobacteria	Burkholderiales	Comamonadaceae	unclassified Comamonadaceae	Comamonadaceae bacterium CG2_30_60_41
59	Bacteria	Proteobacteria	Betaproteobacteria	Burkholderiales	Comamonadaceae	unclassified Comamonadaceae	
59	Bacteria	Proteobacteria	Alphaproteobacteria	Sphingomonadales	Erythrobacteraceae	Croceicoccus	Croceicoccus pelagius
59	Bacteria	Proteobacteria	Alphaproteobacteria	Rhizobiales	Hyphomicrobiaceae	Filomicrobium	
59	Bacteria	Proteobacteria	Alphaproteobacteria	Rhizobiales	Bradyrhizobiaceae	Bosea	
59	Bacteria	Nitrospirae	Nitrospira	Nitrospirales	Nitrospiraceae	Nitrospira	Nitrospira moscoviensis
58	Bacteria	Proteobacteria	delta/epsilon subdivisions	Deltaproteobacteria	Desulfovibrionales	Desulfovibrionaceae	Desulfovibrio
58	Bacteria	Proteobacteria	Betaproteobacteria	Methylophilales	Methylophilaceae	Methylovorus	Methylovorus sp. MM1
58	Bacteria	Proteobacteria	Betaproteobacteria	Burkholderiales	unclassified Burkholderiales	unclassified Burkholderiales (miscellaneous)	Burkholderiales bacterium 70-64
58	Bacteria	Proteobacteria	Betaproteobacteria	Burkholderiales	unclassified Burkholderiales	Burkholderiales Genera incertae sedis	Tepidimonas
58	Bacteria	Proteobacteria	Betaproteobacteria	Burkholderiales	unclassified Burkholderiales	Burkholderiales Genera incertae sedis	Rubrivivax
58	Bacteria	Proteobacteria	Betaproteobacteria	Burkholderiales	Comamonadaceae	Brachymonas	Brachymonas denitrificans
58	Bacteria	Proteobacteria	Alphaproteobacteria	unclassified Alphaproteobacteria	unclassified Alphaproteobacteria (miscellaneous)	Alphaproteobacteria bacterium BRH_c36	
58	Bacteria	Proteobacteria	Alphaproteobacteria	Sphingomonadales	Sphingomonadaceae	Sphingomonas	Sphingomonas sp. SS-249
58	Bacteria	Proteobacteria	Alphaproteobacteria	Rhodobacterales	Rhodobacteraceae	Pseudorhodobacter	Pseudorhodobacter aquimaris
58	Bacteria	Proteobacteria	Alphaproteobacteria	Rhizobiales	Hyphomicrobiaceae	Devosia	
57	Bacteria	Proteobacteria	delta/epsilon subdivisions	Deltaproteobacteria	Myxococcales	Nannocystineae	Kofleriaceae
57	Bacteria	Proteobacteria	Alphaproteobacteria	Sphingomonadales	Sphingomonadaceae	Novosphingobium	Novosphingobium pentaromativorans
57	Bacteria	Proteobacteria	Alphaproteobacteria	Sphingomonadales	Erythrobacteraceae	Altererythrobacter	Altererythrobacter ishigakiensis
57	Bacteria	Proteobacteria	Alphaproteobacteria	Rhodobacterales	Rhodobacteraceae	Natronohydrobacter	Natronohydrobacter thiooxidans
57	Bacteria	Proteobacteria	Alphaproteobacteria	Rhodobacterales	Hyphomonadaceae	Hyphomonas	
57	Bacteria	Proteobacteria	Alphaproteobacteria	Rhizobiales	Hyphomicrobiaceae	Hyphomicrobium	Hyphomicrobium zavarzinii
57	Bacteria	PVC group	Planctomycetes	Planctomycetia	Planctomycetales	Planctomycetaceae	Pirellula
56	Bacteria	unclassified Bacteria	unclassified Bacteria (miscellaneous)	bacterium A7P-90m			
56	Bacteria	Terrabacteria group	Firmicutes	Clostridia	Clostridiales	Clostridiaceae	Clostridium
56	Bacteria	Proteobacteria	Alphaproteobacteria	Sphingomonadales	Erythrobacteraceae	Porphyrobacter	Porphyrobacter mercurialis
56	Bacteria	Proteobacteria	Alphaproteobacteria	Rhodobacterales	Rhodobacteraceae	Roseicitreum	Roseicitreum antarcticum
56	Bacteria	Proteobacteria	Alphaproteobacteria	Rhizobiales	Hyphomicrobiaceae	Hyphomicrobium	Hyphomicrobium sp. CS1BSMeth3
56	Bacteria	PVC group	Verrucomicrobia	Spartobacteria	Chthoniobacterales	Chthoniobacteraceae	Chthoniobacter
55	Bacteria	Proteobacteria	Betaproteobacteria	unclassified Betaproteobacteria	unclassified Betaproteobacteria (miscellaneous)	Betaproteobacteria bacterium RIFCSPLOWO2_12_FULL_65_14	

55	Bacteria	Proteobacteria	Betaproteobacteria	Burkholderiales	unclassified Burkholderiales	unclassified Burkholderiales (miscellaneous)	Burkholderiales bacterium RIFCSPLOWO2_12_FULL_65_40
55	Bacteria	Proteobacteria	Betaproteobacteria	Burkholderiales	Comamonadaceae	Pseudacidovorax	
55	Bacteria	Proteobacteria	Betaproteobacteria	Burkholderiales	Comamonadaceae	Lampropedia	Lampropedia hyalina
55	Bacteria	Proteobacteria	Betaproteobacteria	Burkholderiales	Comamonadaceae	Acidovorax	Acidovorax sp. Leaf160
55	Bacteria	Proteobacteria	Alphaproteobacteria	Sphingomonadales	Sphingomonadaceae	Sphingomonas	Sphingomonas sp. CCH10-B3
55	Bacteria	Proteobacteria	Alphaproteobacteria	Sphingomonadales	Erythrobacteraceae	Porphyrobacter	Porphyrobacter sp. LM 6
54	Bacteria	Terrabacteria group	Actinobacteria	Actinobacteria	Propionibacteriales	Nocardioidaceae	Nocardioides
54	Bacteria	Proteobacteria	Gammaproteobacteria	Xanthomonadales	Xanthomonadaceae	Lysobacter	
54	Bacteria	Proteobacteria	Betaproteobacteria	Rhodocyclales	Rhodocyclaceae	Azonexus	Azonexus hydrophilus
54	Bacteria	Proteobacteria	Alphaproteobacteria	Sphingomonadales	Sphingomonadaceae	Blastomonas	Blastomonas sp. CACIA14H2
54	Bacteria	Proteobacteria	Alphaproteobacteria	Sphingomonadales	Erythrobacteraceae	Porphyrobacter	Porphyrobacter cryptus
54	Bacteria	Proteobacteria	Alphaproteobacteria	Sphingomonadales	Erythrobacteraceae	Erythrobacter	Erythrobacter sp. AP23
54	Bacteria	Proteobacteria	Alphaproteobacteria	Rhodospirillales	Rhodospirillaceae	Inquilinus	Inquilinus limosus
54	Bacteria	Proteobacteria	Alphaproteobacteria	Rhizobiales	Hyphomicrobiaceae	Rhodoplanes	Rhodoplanes sp. Z2-YC6860
54	Bacteria	PVC group	Planctomycetes	Planctomycetia	Planctomycetales	Planctomycetaceae	Planctomicrobium
54	Bacteria	FCB group	Bacteroidetes/Chlorobi group	Bacteroidetes	Flavobacteriia	Flavobacteriales	Flavobacteriaceae
54	Bacteria	Deferribacteres	Deferribacteres	Deferribacterales	Deferribacteraceae	Geovibrio	Geovibrio sp. L21-Ace-BES
53	Bacteria	Proteobacteria	Gammaproteobacteria	Xanthomonadales	Rhodanobacteraceae	Rhodanobacter	
53	Bacteria	Proteobacteria	Betaproteobacteria	Burkholderiales	unclassified Burkholderiales	Burkholderiales Genera incertae sedis	Thiomonas
53	Bacteria	Proteobacteria	Betaproteobacteria	Burkholderiales	unclassified Burkholderiales	Burkholderiales Genera incertae sedis	Rhizobacter
53	Bacteria	Proteobacteria	Alphaproteobacteria	Sphingomonadales	Erythrobacteraceae	Erythrobacter	Erythrobacter sp. QSSC1-22B
53	Bacteria	Proteobacteria	Alphaproteobacteria	Rhodospirillales	Rhodospirillaceae	unclassified Rhodospirillaceae	Rhodospirillaceae bacterium CCH5-H10
53	Bacteria	Proteobacteria	Alphaproteobacteria	Rhodobacterales	Rhodobacteraceae	Oceanicola	Oceanicola litoreus
53	Bacteria	PVC group	Planctomycetes	unclassified Planctomycetes			
53	Bacteria	FCB group	Bacteroidetes/Chlorobi group	Bacteroidetes	Bacteroidia	Bacteroidales	Porphyromonadaceae
52	Bacteria	Proteobacteria	delta/epsilon subdivisions	Deltaproteobacteria			
52	Bacteria	Proteobacteria	Betaproteobacteria	Burkholderiales	Oxalobacteraceae	Herbaspirillum	
52	Bacteria	Proteobacteria	Alphaproteobacteria	Sphingomonadales	Sphingomonadaceae	Novosphingobium	Novosphingobium sp. PP1Y
52	Bacteria	Proteobacteria	Alphaproteobacteria	Sphingomonadales	Sphingomonadaceae	Novosphingobium	Novosphingobium sp. MD-1
52	Bacteria	Proteobacteria	Alphaproteobacteria	Sphingomonadales	Erythrobacteraceae	Erythrobacter	Erythrobacter nanhaisediminis
52	Bacteria	Proteobacteria	Alphaproteobacteria	Sphingomonadales	Erythrobacteraceae	Erythrobacter	Erythrobacter longus
52	Bacteria	Proteobacteria	Alphaproteobacteria	Rhodobacterales	Rhodobacteraceae	unclassified Rhodobacteraceae	Rhodobacteraceae bacterium HLUCCA08
52	Bacteria	Nitrospirae	Nitrospira	Nitrospirales	Nitrospiraceae	Nitrospira	Nitrospira sp. SG-bin2
52	Bacteria	FCB group	Bacteroidetes/Chlorobi group	Bacteroidetes	Bacteroidia		
51	Eukaryota	Amoebozoa	Discosea	Longamoebia	Centramoebida	Acanthamoebidae	Acanthamoeba
51	Bacteria	Proteobacteria	Betaproteobacteria	unclassified Betaproteobacteria	unclassified Betaproteobacteria (miscellaneous)	Betaproteobacteria bacterium UKL13-2	
51	Bacteria	Proteobacteria	Betaproteobacteria	Burkholderiales	Comamonadaceae	Variovorax	Variovorax sp. YR216
51	Bacteria	Proteobacteria	Betaproteobacteria	Burkholderiales	Comamonadaceae	Polaromonas	Polaromonas sp. CF318
51	Bacteria	Proteobacteria	Betaproteobacteria	Burkholderiales	Comamonadaceae	Acidovorax	Acidovorax valerianellae
51	Bacteria	Proteobacteria	Alphaproteobacteria	Sphingomonadales	Sphingomonadaceae	Sphingomonas	Sphingomonas sp. MCT13
51	Bacteria	Proteobacteria	Alphaproteobacteria	Rhodobacterales	Rhodobacteraceae	Rhodobacter	Candidatus Rhodobacter lobularis
51	Bacteria	Proteobacteria	Alphaproteobacteria	Rhizobiales	Hyphomicrobiaceae	Hyphomicrobium	Hyphomicrobium nitrativorans
51	Bacteria	Proteobacteria	Alphaproteobacteria	Caulobacterales	Caulobacteraceae	Phenylobacterium	
51	Bacteria	FCB group	Bacteroidetes/Chlorobi group	Bacteroidetes	Bacteroidia	Bacteroidales	Porphyromonadaceae
51	Bacteria	Deferribacteres	Deferribacteres	Deferribacterales	Deferribacteraceae	Deferribacter	Deferribacter desulfuricans
50	Bacteria	Proteobacteria	Betaproteobacteria	Burkholderiales	unclassified Burkholderiales	Burkholderiales Genera incertae sedis	Methylibium
50	Bacteria	Proteobacteria	Betaproteobacteria	Burkholderiales	Comamonadaceae	Comamonas	Comamonas terrigena
50	Bacteria	Proteobacteria	Betaproteobacteria	Burkholderiales	Comamonadaceae	Acidovorax	Acidovorax sp. RAC01
50	Bacteria	Proteobacteria	Alphaproteobacteria	Sphingomonadales	Sphingomonadaceae	Novosphingobium	Novosphingobium sp. AAP93
50	Bacteria	Proteobacteria	Alphaproteobacteria	Sphingomonadales	Sphingomonadaceae	Citromicrobium	
50	Bacteria	Proteobacteria	Alphaproteobacteria	Rhodobacterales	Rhodobacteraceae	unclassified Rhodobacteraceae	Rhodobacteraceae bacterium GWF1_65_7
50	Bacteria	Proteobacteria	Alphaproteobacteria	Rhizobiales	Phyllobacteriaceae	Mesorhizobium	Mesorhizobium sp. F7
50	Bacteria	Proteobacteria	Alphaproteobacteria	Rhizobiales	Hyphomicrobiaceae	Hyphomicrobium	Hyphomicrobium sp. CS1GBMeth3
50	Bacteria	FCB group	Bacteroidetes/Chlorobi group	Bacteroidetes	Bacteroidia	Bacteroidales	Rikenellaceae
49	Bacteria	Proteobacteria	Betaproteobacteria	Burkholderiales	Comamonadaceae	Acidovorax	Acidovorax konjaci
49	Bacteria	Proteobacteria	Alphaproteobacteria	Sphingomonadales	Sphingomonadaceae	Sphingomonas	Sphingomonas sp. Ndbn-20
49	Bacteria	Proteobacteria	Alphaproteobacteria	Sphingomonadales	Sphingomonadaceae	Sphingomonas	Sphingomonas sp. ERG5
49	Bacteria	Proteobacteria	Alphaproteobacteria	Sphingomonadales	Sphingomonadaceae	Sphingomonas	Sphingomonas astaxanthinifaciens
49	Bacteria	Proteobacteria	Alphaproteobacteria	Sphingomonadales	Erythrobacteraceae	Croceicoccus	Croceicoccus mobilis
49	Bacteria	Proteobacteria	Alphaproteobacteria	Rhodobacterales	Rhodobacteraceae	Rhodobacter	Rhodobacter aestuarii
49	Bacteria	Proteobacteria	Alphaproteobacteria	Rhizobiales	Rhizobiaceae	Rhizobium/Agrobacterium group	Rhizobium
49	Bacteria	PVC group	Planctomycetes	Planctomycetia	Planctomycetales	Planctomycetaceae	Gimesia
48	Bacteria	Proteobacteria	Betaproteobacteria	Burkholderiales	unclassified Burkholderiales	Burkholderiales Genera incertae sedis	Roseateles
48	Bacteria	Proteobacteria	Betaproteobacteria	Burkholderiales	Comamonadaceae	Comamonas	Comamonas sp. SCN 65-56
48	Bacteria	Proteobacteria	Betaproteobacteria	Burkholderiales	Comamonadaceae	Brachymonas	Brachymonas chironomi
48	Bacteria	Proteobacteria	Alphaproteobacteria	Rhodobacterales	Rhodobacteraceae	Pseudoruegeria	Pseudoruegeria aquimaris
48	Bacteria	Proteobacteria	Alphaproteobacteria	Rhodobacterales	Rhodobacteraceae	Actibacterium	Actibacterium mucosum
48	Bacteria	Proteobacteria	Alphaproteobacteria	Rhizobiales	Hyphomicrobiaceae		
48	Bacteria	FCB group	Bacteroidetes/Chlorobi group	Bacteroidetes	Bacteroidia	Bacteroidales	Bacteroidaceae
47	Bacteria	Synergistetes	Synergistia	Synergistales	Synergistaceae	Aminomonas	Aminomonas paucivorans
47	Bacteria	Proteobacteria	unclassified Proteobacteria	unclassified Proteobacteria (miscellaneous)	Proteobacteria bacterium ST_bin13		
47	Bacteria	Proteobacteria	delta/epsilon subdivisions	Deltaproteobacteria	Desulfovibrionales	Desulfovibrionaceae	Desulfocurvus
47	Bacteria	Proteobacteria	Gammaproteobacteria	unclassified Gammaproteobacteria	unclassified Gammaproteobacteria (miscellaneous)	Gammaproteobacteria bacterium SG8_31	
47	Bacteria	Proteobacteria	Betaproteobacteria	Burkholderiales	unclassified Burkholderiales	Burkholderiales Genera incertae sedis	Rubrivivax
47	Bacteria	Proteobacteria	Betaproteobacteria	Burkholderiales	Comamonadaceae	unclassified Comamonadaceae	Comamonadaceae bacterium BICA1-1
47	Bacteria	Proteobacteria	Alphaproteobacteria	Sphingomonadales	Sphingomonadaceae	Sphingomonas	Sphingomonas sp. PR090111-T3T-6A
47	Bacteria	Proteobacteria	Alphaproteobacteria	Rhodobacterales	Rhodobacteraceae	Roseisalinus	Roseisalinus antarcticus
47	Bacteria	Proteobacteria	Alphaproteobacteria	Rhodobacterales	Rhodobacteraceae	Rhodovulum	Rhodovulum sulfidophilum
47	Bacteria	Proteobacteria	Alphaproteobacteria	Rhizobiales	unclassified Rhizobiales	Pseudorhodoplanes	Pseudorhodoplanes sinuspersici
47	Bacteria	Proteobacteria	Alphaproteobacteria	Rhizobiales	Rhizobiaceae	Sinorhizobium/Ensifer group	
47	Bacteria	Proteobacteria	Alphaproteobacteria	Caulobacterales	Caulobacteraceae	Caulobacter	
47	Bacteria	FCB group	Bacteroidetes/Chlorobi group	Bacteroidetes	Bacteroidia	Bacteroidales	Porphyromonadaceae
46	Bacteria	Proteobacteria	Betaproteobacteria	Rhodocyclales	Rhodocyclaceae		
46	Bacteria	Proteobacteria	Betaproteobacteria	Methylophilales	Methylophilaceae	Candidatus Methylopumilus	Candidatus Methylopumilus turicensis
46	Bacteria	Proteobacteria	Betaproteobacteria	Burkholderiales	Oxalobacteraceae	Duganella	
46	Bacteria	Proteobacteria	Betaproteobacteria	Burkholderiales	Comamonadaceae	Variovorax	Variovorax sp. HW608
46	Bacteria	Proteobacteria	Alphaproteobacteria	Rhodobacterales	unclassified Rhodobacterales	Rhodobacterales bacterium 65-51	
46	Bacteria	Proteobacteria	Alphaproteobacteria	Rhodobacterales	Rhodobacteraceae	Oceanicola	Oceanicola granulosus
46	Bacteria	Proteobacteria	Alphaproteobacteria	Rhodobacterales	Rhodobacteraceae	Labrenzia	
46	Bacteria	Nitrospirae	Nitrospira	Nitrospirales	Nitrospiraceae	Nitrospira	Nitrospira sp. ST-bin4
46	Bacteria	FCB group	Gemmatimonadetes	Gemmatimonadetes	Gemmatimonadales	Gemmatimonadaceae	Gemmatirosa
46	Bacteria	FCB group	Bacteroidetes/Chlorobi group	Bacteroidetes	Flavobacteriia	Flavobacteriales	Flavobacteriaceae
46	Bacteria	FCB group	Bacteroidetes/Chlorobi group	Bacteroidetes	Bacteroidia	Bacteroidales	Porphyromonadaceae
46	Bacteria	Deferribacteres	Deferribacteres	Deferribacterales	Deferribacteraceae	Denitrovibrio	Denitrovibrio acetiphilus
45	Bacteria	unclassified Bacteria	unclassified Bacteria (miscellaneous)	bacterium YEK0313			
45	Bacteria	Proteobacteria	delta/epsilon subdivisions	Deltaproteobacteria	Desulfovibrionales	Desulfovibrionaceae	Desulfovibrio
45	Bacteria	Proteobacteria	Betaproteobacteria	Rhodocyclales	Rhodocyclaceae	Azoarcus	
45	Bacteria	Proteobacteria	Betaproteobacteria	Burkholderiales	Comamonadaceae	Pseudorhodoferax	Pseudorhodoferax sp. Leaf274
45	Bacteria	Proteobacteria	Betaproteobacteria	Burkholderiales	Comamonadaceae	Ottowia	Ottowia sp. oral taxon 894
45	Bacteria	Proteobacteria	Alphaproteobacteria	Sphingomonadales	Sphingomonadaceae	Sphingomonas	Sphingomonas koreensis
45	Bacteria	Proteobacteria	Alphaproteobacteria	Sphingomonadales	Sphingomonadaceae	Blastomonas	Blastomonas sp. CCH5-A3
45	Bacteria	Proteobacteria	Alphaproteobacteria	Rickettsiales	Anaplasmataceae	Candidatus Neoehrlichia	Candidatus Neoehrlichia lotoris
45	Bacteria	Proteobacteria	Alphaproteobacteria	Rhodobacterales	Rhodobacteraceae	Oceanicola	Oceanicola sp. MCTG156(1a)
45	Bacteria	Proteobacteria	Alphaproteobacteria	Rhizobiales	unclassified Rhizobiales	Bauldia	Bauldia litoralis
45	Bacteria	Proteobacteria	Alphaproteobacteria	Rhizobiales	Rhizobiaceae	Sinorhizobium/Ensifer group	Sinorhizobium
45	Bacteria	FCB group	Bacteroidetes/Chlorobi group	Bacteroidetes	Flavobacteriia	Flavobacteriales	Flavobacteriaceae
44	Eukaryota	Stramenopiles	PX clade	Phaeophyceae	Ectocarpales	Chordariaceae	Leathesia
44	Eukaryota	Opisthokonta	Fungi	Neocallimastigomycota	Neocallimastigomycetes	Neocallimastigales	Neocallimastigaceae
44	Bacteria	Terrabacteria group	Actinobacteria	Actinobacteria	unclassified Actinobacteria (class)	unclassified Actinobacteria (class) (miscellaneous)	Actinobacteria bacterium IMCC26207
44	Bacteria	Proteobacteria	Gammaproteobacteria	Oceanospirillales	Halomonadaceae	Halomonas	
44	Bacteria	Proteobacteria	Betaproteobacteria	Rhodocyclales	Rhodocyclaceae	Propionivibrio	Candidatus Propionivibrio aalborgensis
44	Bacteria	Proteobacteria	Betaproteobacteria	Methylophilales	unclassified Methylophilales	Methylophilales bacterium LSUCC0135	
44	Bacteria	Proteobacteria	Betaproteobacteria	Burkholderiales	Comamonadaceae	Acidovorax	Acidovorax sp. SCN 68-22
44	Bacteria	Proteobacteria	Betaproteobacteria	Burkholderiales	Comamonadaceae	Acidovorax	Acidovorax sp. NO-1
44	Bacteria	Proteobacteria	Betaproteobacteria	Burkholderiales	Burkholderiaceae	Burkholderia	Burkholderia ubonensis
44	Bacteria	Proteobacteria	Betaproteobacteria	Burkholderiales	Alcaligenaceae		
44	Bacteria	Proteobacteria	Alphaproteobacteria	unclassified Alphaproteobacteria	unclassified Alphaproteobacteria (miscellaneous)	Alphaproteobacteria bacterium 13_2_20CM_2_64_7	
44	Bacteria	Proteobacteria	Alphaproteobacteria	Sphingomonadales	Sphingomonadaceae	Sphingomonas	Sphingomonas sp. Root710
44	Bacteria	Proteobacteria	Alphaproteobacteria	Sphingomonadales	Sphingomonadaceae	Sphingomonas	Sphingomonas sp. Leaf357
44	Bacteria	Proteobacteria	Alphaproteobacteria	Sphingomonadales	Sphingomonadaceae	Novosphingobium	Novosphingobium sp. CCH12-A3
44	Bacteria	Proteobacteria	Alphaproteobacteria	Rhizobiales	unclassified Rhizobiales	unclassified Rhizobiales (miscellaneous)	
44	Bacteria	PVC group	Planctomycetes	Planctomycetia	Planctomycetales	Planctomycetaceae	Roseimaritima
44	Bacteria	FCB group	Bacteroidetes/Chlorobi group	Bacteroidetes	Bacteroidia	Bacteroidales	Porphyromonadaceae
44	Bacteria	Deferribacteres	Deferribacteres	Deferribacterales	Deferribacteraceae	Calditerrivibrio	Calditerrivibrio nitroreducens
43	Bacteria	Proteobacteria	Betaproteobacteria	unclassified Betaproteobacteria	unclassified Betaproteobacteria (miscellaneous)	Betaproteobacteria bacterium RBG_16_66_20	
43	Bacteria	Proteobacteria	Betaproteobacteria	Rhodocyclales	Rhodocyclaceae	Dechloromonas	Dechloromonas aromatica
43	Bacteria	Proteobacteria	Betaproteobacteria	Burkholderiales	Comamonadaceae	Acidovorax	Acidovorax delafieldii
43	Bacteria	Proteobacteria	Betaproteobacteria	Burkholderiales	Burkholderiaceae	Cupriavidus	Cupriavidus basilensis
43	Bacteria	Proteobacteria	Alphaproteobacteria	Sphingomonadales	Sphingomonadaceae	Sphingopyxis	Sphingopyxis sp. KK2
43	Bacteria	Proteobacteria	Alphaproteobacteria	Sphingomonadales	Sphingomonadaceae	Sphingomonas	Sphingomonas soli
43	Bacteria	Proteobacteria	Alphaproteobacteria	Sphingomonadales	Erythrobacteraceae	Erythrobacter	Erythrobacter sp. YT30
43	Bacteria	Proteobacteria	Alphaproteobacteria	Rhodospirillales	unclassified Rhodospirillales	Enhydrobacter	Enhydrobacter aerosaccus
43	Bacteria	Proteobacteria	Alphaproteobacteria	Rhodobacterales	Rhodobacteraceae	Paracoccus	Paracoccus sanguinis
43	Bacteria	Proteobacteria	Alphaproteobacteria	Rhodobacterales	Rhodobacteraceae	Loktanella	Loktanella koreensis
43	Bacteria	Proteobacteria	Alphaproteobacteria	Rhodobacterales	Rhodobacteraceae	Aestuariivita	Aestuariivita atlantica
43	Bacteria	Proteobacteria	Alphaproteobacteria	Rhizobiales	Phyllobacteriaceae	Nitratireductor	Nitratireductor basaltis
43	Bacteria	Elusimicrobia	unclassified Elusimicrobia				
42	Bacteria	Proteobacteria	delta/epsilon subdivisions	Deltaproteobacteria	Desulfovibrionales	Desulfovibrionaceae	Desulfovibrio
42	Bacteria	Proteobacteria	Gammaproteobacteria	unclassified Gammaproteobacteria	sulfur-oxidizing symbionts	Bathymodiolus thermophilus thioautotrophic gill symbiont	
42	Bacteria	Proteobacteria	Gammaproteobacteria	Pseudomonadales			
42	Bacteria	Proteobacteria	Gammaproteobacteria	Alteromonadales	Pseudoalteromonadaceae	Pseudoalteromonas	
42	Bacteria	Proteobacteria	Betaproteobacteria	Rhodocyclales	Rhodocyclaceae	Uliginosibacterium	Uliginosibacterium gangwonense
42	Bacteria	Proteobacteria	Betaproteobacteria	Rhodocyclales	Rhodocyclaceae	Propionivibrio	Propionivibrio dicarboxylicus
42	Bacteria	Proteobacteria	Betaproteobacteria	Burkholderiales	unclassified Burkholderiales	unclassified Burkholderiales (miscellaneous)	Burkholderiales bacterium RIFCSPLOWO2_02_FULL_57_36
42	Bacteria	Proteobacteria	Betaproteobacteria	Burkholderiales	Comamonadaceae	Acidovorax	Acidovorax wautersii
42	Bacteria	Proteobacteria	Betaproteobacteria	Burkholderiales	Burkholderiaceae	Pandoraea	
42	Bacteria	Proteobacteria	Alphaproteobacteria	Sphingomonadales	Sphingomonadaceae	Sphingobium	Sphingobium sp. TCM1
42	Bacteria	Proteobacteria	Alphaproteobacteria	Sphingomonadales	Sphingomonadaceae	Sphingobium	Sphingobium sp. EP60837
42	Bacteria	Proteobacteria	Alphaproteobacteria	Sphingomonadales	Sphingomonadaceae	Sphingobium	Sphingobium japonicum
42	Bacteria	Proteobacteria	Alphaproteobacteria	Sphingomonadales	Sphingomonadaceae	Novosphingobium	Novosphingobium sp. ND82Meth1
42	Bacteria	Proteobacteria	Alphaproteobacteria	Rhodobacterales	Rhodobacteraceae	Falsirhodobacter	Falsirhodobacter sp. alg1
42	Bacteria	Proteobacteria	Alphaproteobacteria	Rhizobiales	Rhizobiaceae		
42	Archaea	Euryarchaeota	Halobacteria	Halobacteriales	Haloarculaceae	Natronomonas	Natronomonas pharaonis
41	Bacteria	Proteobacteria	Gammaproteobacteria	Xanthomonadales	Xanthomonadaceae	Lysobacter	Lysobacter antibioticus
41	Bacteria	Proteobacteria	Gammaproteobacteria	Enterobacterales	Enterobacteriaceae	Escherichia	Escherichia coli
41	Bacteria	Proteobacteria	Betaproteobacteria	unclassified Betaproteobacteria	unclassified Betaproteobacteria (miscellaneous)	Betaproteobacteria bacterium RIFCSPLOWO2_12_FULL_64_23	
41	Bacteria	Proteobacteria	Betaproteobacteria	Rhodocyclales	Rhodocyclaceae	unclassified Rhodocyclaceae	Rhodocyclaceae bacterium Paddy-1
41	Bacteria	Proteobacteria	Betaproteobacteria	Burkholderiales	unclassified Burkholderiales	Burkholderiales Genera incertae sedis	Mitsuaria
41	Bacteria	Proteobacteria	Betaproteobacteria	Burkholderiales	Oxalobacteraceae	Noviherbaspirillum	Noviherbaspirillum sp. Root189
41	Bacteria	Proteobacteria	Betaproteobacteria	Burkholderiales	Comamonadaceae	Comamonas	Comamonas sp. B-9
41	Bacteria	Proteobacteria	Betaproteobacteria	Burkholderiales	Comamonadaceae	Comamonas	Comamonas composti
41	Bacteria	Proteobacteria	Betaproteobacteria	Burkholderiales	Comamonadaceae	Acidovorax	Acidovorax sp. MR-S7
41	Bacteria	Proteobacteria	Betaproteobacteria	Burkholderiales	Burkholderiaceae	Lautropia	Lautropia sp. SCN 69-89
41	Bacteria	Proteobacteria	Alphaproteobacteria	unclassified Alphaproteobacteria	unclassified Alphaproteobacteria (miscellaneous)	Alphaproteobacteria bacterium RIFCSPHIGHO2_12_FULL_66_14	
41	Bacteria	Proteobacteria	Alphaproteobacteria	Sphingomonadales	Sphingomonadaceae	Sphingomonas	Sphingomonas sp. 66-10
41	Bacteria	Proteobacteria	Alphaproteobacteria	Sphingomonadales	Erythrobacteraceae	Erythrobacter	Erythrobacter vulgaris
41	Bacteria	Proteobacteria	Alphaproteobacteria	Rhodobacterales	Rhodobacteraceae	Thalassobius	Thalassobius mediterraneus
41	Bacteria	Proteobacteria	Alphaproteobacteria	Rhodobacterales	Rhodobacteraceae	Paenirhodobacter	Paenirhodobacter enshiensis
41	Bacteria	Proteobacteria	Alphaproteobacteria	Rhodobacterales	Rhodobacteraceae	Jhaorihella	Jhaorihella thermophila
41	Bacteria	PVC group	Planctomycetes	Planctomycetia	Planctomycetales	Planctomycetaceae	Blastopirellula
41	Bacteria	FCB group	Bacteroidetes/Chlorobi group	Bacteroidetes	Bacteroidia	Bacteroidales	unclassified Bacteroidales
40	Bacteria	Terrabacteria group	Actinobacteria	Acidimicrobiia	Acidimicrobiales	Microthrixaceae	Candidatus Microthrix
40	Bacteria	Spirochaetes	Spirochaetia	Spirochaetales	Spirochaetaceae	Treponema	
40	Bacteria	Proteobacteria	unclassified Proteobacteria	unclassified Proteobacteria (miscellaneous)			
40	Bacteria	Proteobacteria	delta/epsilon subdivisions	Deltaproteobacteria	Desulfovibrionales	Desulfovibrionaceae	Desulfovibrio
40	Bacteria	Proteobacteria	Gammaproteobacteria	Pseudomonadales	Moraxellaceae	Acinetobacter	Acinetobacter calcoaceticus/baumannii complex
40	Bacteria	Proteobacteria	Gammaproteobacteria	Methylococcales	Crenotrichaceae	Crenothrix	Crenothrix polyspora
40	Bacteria	Proteobacteria	Betaproteobacteria	unclassified Betaproteobacteria	Candidatus Accumulibacter	Candidatus Accumulibacter sp. 66-26	
40	Bacteria	Proteobacteria	Alphaproteobacteria	Sphingomonadales	Sphingomonadaceae	Sphingomonas	Sphingomonas sp. 37zxx
40	Bacteria	Proteobacteria	Alphaproteobacteria	Sphingomonadales	Sphingomonadaceae	Sphingobium	Sphingobium baderi
40	Bacteria	Proteobacteria	Alphaproteobacteria	Sphingomonadales	Erythrobacteraceae	Porphyrobacter	Porphyrobacter donghaensis
40	Bacteria	Proteobacteria	Alphaproteobacteria	Sphingomonadales	Erythrobacteraceae	Erythrobacter	Erythrobacter sp. SCN 62-14
40	Bacteria	Proteobacteria	Alphaproteobacteria	Sphingomonadales	Erythrobacteraceae	Erythrobacter	Erythrobacter sp. HI0063
40	Bacteria	Proteobacteria	Alphaproteobacteria	Rhodospirillales	Rhodospirillaceae	Thalassospira	
40	Bacteria	Proteobacteria	Alphaproteobacteria	Rhodobacterales	Rhodobacteraceae	unclassified Rhodobacteraceae	Rhodobacteraceae bacterium HLUCCO18
40	Bacteria	Proteobacteria	Alphaproteobacteria	Rhodobacterales	Rhodobacteraceae	Thioclava	Thioclava indica
40	Bacteria	Proteobacteria	Alphaproteobacteria	Rhodobacterales	Rhodobacteraceae	Pseudoruegeria	Pseudoruegeria marinistellae
40	Bacteria	Proteobacteria	Alphaproteobacteria	Caulobacterales	Caulobacteraceae	unclassified Caulobacteraceae	Caulobacteraceae bacterium OTSz_A_272
40	Bacteria	PVC group	Planctomycetes	Planctomycetia	Planctomycetales	Planctomycetaceae	Rubinisphaera
40	Bacteria	PVC group	Candidatus Omnitrophica	unclassified Candidatus Omnitrophica			
39	Bacteria	unclassified Bacteria	Candidatus Vecturithrix	Candidatus Vecturithrix granuli			
39	Bacteria	Terrabacteria group	Armatimonadetes	unclassified Armatimonadetes	Armatimonadetes bacterium 55-13		
39	Bacteria	Terrabacteria group	Actinobacteria	Actinobacteria	Frankiales	Frankiaceae	Frankia
39	Bacteria	Proteobacteria	unclassified Proteobacteria	unclassified Proteobacteria (miscellaneous)	Proteobacteria bacterium ST_bin14		
39	Bacteria	Proteobacteria	delta/epsilon subdivisions	Deltaproteobacteria	Desulfovibrionales	Desulfovibrionaceae	Lawsonia
39	Bacteria	Proteobacteria	delta/epsilon subdivisions	Deltaproteobacteria	Desulfovibrionales	Desulfovibrionaceae	Desulfovibrio
39	Bacteria	Proteobacteria	Gammaproteobacteria	unclassified Gammaproteobacteria	unclassified Gammaproteobacteria (miscellaneous)		
39	Bacteria	Proteobacteria	Gammaproteobacteria	Xanthomonadales	Xanthomonadaceae	Xanthomonas	
39	Bacteria	Proteobacteria	Gammaproteobacteria	Cellvibrionales	Halieaceae	Halioglobus	Halioglobus pacificus
39	Bacteria	Proteobacteria	Betaproteobacteria	unclassified Betaproteobacteria	unclassified Betaproteobacteria (miscellaneous)	Betaproteobacteria bacterium RIFCSPLOWO2_02_FULL_62_17	
39	Bacteria	Proteobacteria	Betaproteobacteria	Rhodocyclales	unclassified Rhodocyclales		
39	Bacteria	Proteobacteria	Betaproteobacteria	Burkholderiales	Comamonadaceae	Pelomonas	Pelomonas sp. Root1444
39	Bacteria	Proteobacteria	Betaproteobacteria	Burkholderiales	Comamonadaceae	Curvibacter	Curvibacter lanceolatus
39	Bacteria	Proteobacteria	Betaproteobacteria	Burkholderiales	Comamonadaceae	Comamonas	Comamonas aquatica
39	Bacteria	Proteobacteria	Alphaproteobacteria	Sphingomonadales	Erythrobacteraceae	Erythrobacter	Erythrobacter sp. RIFCSPHIGHO2_12_FULL_63_10
39	Bacteria	Proteobacteria	Alphaproteobacteria	Rhizobiales	Phyllobacteriaceae	Mesorhizobium	Mesorhizobium sp. B7
39	Bacteria	PVC group	Lentisphaerae	unclassified Lentisphaerae	unclassified Lentisphaerae (miscellaneous)	Lentisphaerae bacterium GWF2_45_14	
39	Bacteria	PVC group	Chlamydiae	Chlamydiia	Chlamydiales	Chlamydiaceae	Chlamydia/Chlamydophila group
39	Bacteria	FCB group	Bacteroidetes/Chlorobi group	Bacteroidetes	Flavobacteriia	Flavobacteriales	Flavobacteriaceae
38	Bacteria	Terrabacteria group	Cyanobacteria/Melainabacteria group	Cyanobacteria	Nostocales	Aphanizomenonaceae	Aphanizomenon
38	Bacteria	Proteobacteria	delta/epsilon subdivisions	Deltaproteobacteria	Desulfovibrionales	Desulfovibrionaceae	Desulfovibrio
38	Bacteria	Proteobacteria	Gammaproteobacteria	Xanthomonadales	Xanthomonadaceae	Pseudoxanthomonas	
38	Bacteria	Proteobacteria	Gammaproteobacteria	Chromatiales	Woeseiaceae	Woeseia	Woeseia oceani
38	Bacteria	Proteobacteria	Gammaproteobacteria	Alteromonadales	Alteromonadaceae	Marinobacter	
38	Bacteria	Proteobacteria	Betaproteobacteria	unclassified Betaproteobacteria	unclassified Betaproteobacteria (miscellaneous)	Betaproteobacteria bacterium SG8_39	
38	Bacteria	Proteobacteria	Betaproteobacteria	unclassified Betaproteobacteria	Candidatus Accumulibacter		
38	Bacteria	Proteobacteria	Betaproteobacteria	Burkholderiales	Comamonadaceae	unclassified Comamonadaceae	Comamonadaceae bacterium B1
38	Bacteria	Proteobacteria	Betaproteobacteria	Burkholderiales	Burkholderiaceae	Ralstonia	Ralstonia solanacearum
38	Bacteria	Proteobacteria	Alphaproteobacteria	unclassified Alphaproteobacteria	unclassified Alphaproteobacteria (miscellaneous)	alpha proteobacterium U9-1i	
38	Bacteria	Proteobacteria	Alphaproteobacteria	unclassified Alphaproteobacteria	unclassified Alphaproteobacteria (miscellaneous)	Alphaproteobacteria bacterium 65-37	
38	Bacteria	Proteobacteria	Alphaproteobacteria	Sphingomonadales	Sphingomonadaceae	Sphingomonas	Sphingomonas sp. LM7
38	Bacteria	Proteobacteria	Alphaproteobacteria	Sphingomonadales	Sphingomonadaceae	Sphingomonas	Sphingomonas hengshuiensis
38	Bacteria	Proteobacteria	Alphaproteobacteria	Sphingomonadales	Erythrobacteraceae	Erythrobacter	Erythrobacter sp. NAP1
38	Bacteria	Proteobacteria	Alphaproteobacteria	Rickettsiales	Anaplasmataceae	Anaplasma	phagocytophilum group
38	Bacteria	Proteobacteria	Alphaproteobacteria	Rhodospirillales	Acetobacteraceae	Roseomonas	Roseomonas stagni
38	Bacteria	Proteobacteria	Alphaproteobacteria	Rhodobacterales	Rhodobacteraceae	Rhodovulum	Rhodovulum sp. P5
38	Bacteria	Proteobacteria	Alphaproteobacteria	Rhizobiales	Rhizobiaceae	Kaistia	
38	Bacteria	Proteobacteria	Alphaproteobacteria	Rhizobiales	Hyphomicrobiaceae	Hyphomicrobium	Hyphomicrobium facile
38	Bacteria	Proteobacteria	Alphaproteobacteria	Caulobacterales	Caulobacteraceae	Caulobacter	Caulobacter henricii
38	Bacteria	PVC group	Planctomycetes	unclassified Planctomycetes	Planctomycetes bacterium RBG_16_64_12		
38	Bacteria	Nitrospirae	Nitrospira	Nitrospirales	Nitrospiraceae	Nitrospira	Candidatus Nitrospira inopinata
38	Bacteria	FCB group	Bacteroidetes/Chlorobi group	Bacteroidetes	Chitinophagia	Chitinophagales	Chitinophagaceae
37	Bacteria	Terrabacteria group	Firmicutes	Clostridia	Clostridiales	Ruminococcaceae	
37	Bacteria	Terrabacteria group	Firmicutes	Bacilli	Bacillales		
37	Bacteria	Proteobacteria	delta/epsilon subdivisions	Deltaproteobacteria	Desulfovibrionales	Desulfovibrionaceae	Desulfovibrio
37	Bacteria	Proteobacteria	Betaproteobacteria	unclassified Betaproteobacteria	unclassified Betaproteobacteria (miscellaneous)	beta proteobacterium CB	
37	Bacteria	Proteobacteria	Betaproteobacteria	Rhodocyclales	unclassified Rhodocyclales	Rhodocyclales bacterium GWA2_65_20	
37	Bacteria	Proteobacteria	Betaproteobacteria	Rhodocyclales	Rhodocyclaceae	Azospira	Azospira oryzae
37	Bacteria	Proteobacteria	Betaproteobacteria	Methylophilales	Methylophilaceae	Methylobacillus	Methylobacillus sp. MM2
37	Bacteria	Proteobacteria	Betaproteobacteria	Gallionellales	unclassified Gallionellales		
37	Bacteria	Proteobacteria	Betaproteobacteria	Burkholderiales	Comamonadaceae	Melaminivora	Melaminivora alkalimesophila
37	Bacteria	Proteobacteria	Betaproteobacteria	Burkholderiales	Comamonadaceae	Acidovorax	Acidovorax sp. JHL-3
37	Bacteria	Proteobacteria	Betaproteobacteria	Burkholderiales	Comamonadaceae	Acidovorax	Acidovorax sp. CF316
37	Bacteria	Proteobacteria	Betaproteobacteria	Burkholderiales	Burkholderiaceae	Ralstonia	
37	Bacteria	Proteobacteria	Alphaproteobacteria	Sphingomonadales	Sphingomonadaceae	Sphingopyxis	Sphingopyxis sp. H115
37	Bacteria	Proteobacteria	Alphaproteobacteria	Sphingomonadales	Sphingomonadaceae	Sphingomonas	Sphingomonas sp. URHD0057
37	Bacteria	Proteobacteria	Alphaproteobacteria	Sphingomonadales	Sphingomonadaceae	Sphingobium	Sphingobium sp. Leaf26
37	Bacteria	Proteobacteria	Alphaproteobacteria	Sphingomonadales	Sphingomonadaceae	Novosphingobium	Novosphingobium sp. SCN 66-18
37	Bacteria	Proteobacteria	Alphaproteobacteria	Rhodobacterales	Rhodobacteraceae	Nioella	Nioella sediminis
37	Bacteria	Proteobacteria	Alphaproteobacteria	Rhodobacterales	Rhodobacteraceae	Jannaschia	Jannaschia sp. EhC01
37	Bacteria	Proteobacteria	Alphaproteobacteria	Rhodobacterales	Rhodobacteraceae	Haematobacter	Haematobacter massiliensis
37	Bacteria	Proteobacteria	Alphaproteobacteria	Rhizobiales	unclassified Rhizobiales	unclassified Rhizobiales (miscellaneous)	Rhizobiales bacterium 62-17
37	Bacteria	Proteobacteria	Alphaproteobacteria	Rhizobiales	Aurantimonadaceae	Aureimonas	
37	Bacteria	FCB group	Bacteroidetes/Chlorobi group	Bacteroidetes	Bacteroidia	Bacteroidales	Rikenellaceae
36	Eukaryota	Opisthokonta	Fungi	Microsporidia	Apansporoblastina	Enterocytozoonidae	Enterospora
36	Bacteria	Terrabacteria group	Actinobacteria	Actinobacteria	unclassified Actinobacteria (class)	unclassified Actinobacteria (class) (miscellaneous)	marine actinobacterium MedAcidi-G3
36	Bacteria	Proteobacteria	unclassified Proteobacteria	unclassified Proteobacteria (miscellaneous)	Proteobacteria bacterium HN_bin10		
36	Bacteria	Proteobacteria	Betaproteobacteria	Burkholderiales	unclassified Burkholderiales	Burkholderiales Genera incertae sedis	Thiomonas
36	Bacteria	Proteobacteria	Betaproteobacteria	Burkholderiales	Oxalobacteraceae	Janthinobacterium	
36	Bacteria	Proteobacteria	Betaproteobacteria	Burkholderiales	Burkholderiaceae	Lautropia	Lautropia sp. SCN 70-15
36	Bacteria	Proteobacteria	Betaproteobacteria	Burkholderiales	Alcaligenaceae	Bordetella	Bordetella petrii
36	Bacteria	Proteobacteria	Alphaproteobacteria	unclassified Alphaproteobacteria	Geminicoccus	Geminicoccus roseus	
36	Bacteria	Proteobacteria	Alphaproteobacteria	Sphingomonadales	Sphingomonadaceae	Sphingomonas	Sphingomonas sp. PAMC 26605
36	Bacteria	Proteobacteria	Alphaproteobacteria	Sphingomonadales	Erythrobacteraceae	unclassified Erythrobacteraceae	Erythrobacteraceae bacterium CCH12-C2
36	Bacteria	Proteobacteria	Alphaproteobacteria	Rhodobacterales	Rhodobacteraceae	Puniceibacterium	Puniceibacterium sp. IMCC21224
36	Bacteria	Proteobacteria	Alphaproteobacteria	Rhodobacterales	Rhodobacteraceae	Pseudooceanicola	Pseudooceanicola flagellatus
36	Bacteria	Proteobacteria	Alphaproteobacteria	Rhodobacterales	Rhodobacteraceae	Maritimibacter	Maritimibacter sp. HL-12
36	Bacteria	Proteobacteria	Alphaproteobacteria	Rhizobiales	Rhodobiaceae	Tepidicaulis	Tepidicaulis marinus
36	Bacteria	Proteobacteria	Alphaproteobacteria	Rhizobiales	Hyphomicrobiaceae	Prosthecomicrobium	Prosthecomicrobium hirschii
36	Bacteria	Proteobacteria	Alphaproteobacteria	Rhizobiales	Beijerinckiaceae	Beijerinckia	Beijerinckia sp. 28-YEA-48
36	Bacteria	PVC group	Verrucomicrobia	Opitutae	Opitutales	Opitutaceae	Opitutus
36	Bacteria	PVC group	Lentisphaerae	unclassified Lentisphaerae	unclassified Lentisphaerae (miscellaneous)	Lentisphaerae bacterium GWF2_44_16	
36	Bacteria	FCB group	Bacteroidetes/Chlorobi group	Bacteroidetes	Bacteroidia	Bacteroidales	Rikenellaceae
35	Bacteria	Terrabacteria group	Firmicutes	Clostridia	Clostridiales	Lachnospiraceae	
35	Bacteria	Terrabacteria group	Actinobacteria	Actinobacteria	Corynebacteriales	Nocardiaceae	Rhodococcus
35	Bacteria	Synergistetes	Synergistia	Synergistales	Synergistaceae		
35	Bacteria	Proteobacteria	delta/epsilon subdivisions	Deltaproteobacteria	Desulfovibrionales	Desulfovibrionaceae	Desulfovibrio
35	Bacteria	Proteobacteria	Gammaproteobacteria	Oceanospirillales	Alcanivoracaceae	Alcanivorax	Alcanivorax pacificus
35	Bacteria	Proteobacteria	Betaproteobacteria	unclassified Betaproteobacteria	Candidatus Accumulibacter	Candidatus Accumulibacter phosphatis	
35	Bacteria	Proteobacteria	Betaproteobacteria	Rhodocyclales	Rhodocyclaceae	Zoogloea	Zoogloea sp. LCSB751
35	Bacteria	Proteobacteria	Betaproteobacteria	Rhodocyclales	Rhodocyclaceae	Azoarcus	Azoarcus sp. KH32C
35	Bacteria	Proteobacteria	Betaproteobacteria	Burkholderiales	unclassified Burkholderiales	Burkholderiales Genera incertae sedis	Roseateles
35	Bacteria	Proteobacteria	Betaproteobacteria	Burkholderiales	Comamonadaceae	Variovorax	Variovorax boronicumulans
35	Bacteria	Proteobacteria	Betaproteobacteria	Burkholderiales	Comamonadaceae	Rhodoferax	Rhodoferax sp. RIFCSPLOWO2_12_FULL_60_11
35	Bacteria	Proteobacteria	Betaproteobacteria	Burkholderiales	Comamonadaceae	Lampropedia	Lampropedia cohaerens
35	Bacteria	Proteobacteria	Betaproteobacteria	Burkholderiales	Burkholderiaceae	Burkholderia	Burkholderia cepacia complex
35	Bacteria	Proteobacteria	Betaproteobacteria	Burkholderiales	Alcaligenaceae	Advenella	Advenella kashmirensis
35	Bacteria	Proteobacteria	Alphaproteobacteria	Sphingomonadales	Sphingomonadaceae	unclassified Sphingomonadaceae	unclassified Sphingomonadaceae (miscellaneous)
35	Bacteria	Proteobacteria	Alphaproteobacteria	Sphingomonadales	Sphingomonadaceae	Sphingopyxis	Sphingopyxis sp. LC363
35	Bacteria	Proteobacteria	Alphaproteobacteria	Sphingomonadales	Sphingomonadaceae	Sphingomonas	Sphingomonas sp. W16RD
35	Bacteria	Proteobacteria	Alphaproteobacteria	Sphingomonadales	Sphingomonadaceae	Sphingomonas	Sphingomonas sp. TZW2008
35	Bacteria	Proteobacteria	Alphaproteobacteria	Sphingomonadales	Sphingomonadaceae	Sphingomonas	Sphingomonas sp. Leaf33
35	Bacteria	Proteobacteria	Alphaproteobacteria	Sphingomonadales	Sphingomonadaceae	Sphingobium	Sphingobium yanoikuyae
35	Bacteria	Proteobacteria	Alphaproteobacteria	Sphingomonadales	Sphingomonadaceae	Sphingobium	Sphingobium sp. C100
35	Bacteria	Proteobacteria	Alphaproteobacteria	Sphingomonadales	Sphingomonadaceae	Novosphingobium	Novosphingobium sp. P6W
35	Bacteria	Proteobacteria	Alphaproteobacteria	Sphingomonadales	Erythrobacteraceae	Porphyrobacter	Porphyrobacter neustonensis
35	Bacteria	Proteobacteria	Alphaproteobacteria	Rhodospirillales	Rhodospirillaceae	Azospirillum	Azospirillum lipoferum
35	Bacteria	Proteobacteria	Alphaproteobacteria	Rhodobacterales	Rhodobacteraceae	Wenxinia	Wenxinia saemankumensis
35	Bacteria	Proteobacteria	Alphaproteobacteria	Rhodobacterales	Rhodobacteraceae	Ruegeria	
35	Bacteria	Proteobacteria	Alphaproteobacteria	Rhodobacterales	Rhodobacteraceae	Rubrimonas	Rubrimonas cliftonensis
35	Bacteria	Proteobacteria	Alphaproteobacteria	Rhodobacterales	Rhodobacteraceae	Pseudorhodobacter	
35	Bacteria	Proteobacteria	Alphaproteobacteria	Rhodobacterales	Rhodobacteraceae	Donghicola	
35	Bacteria	Proteobacteria	Alphaproteobacteria	Rhizobiales	unclassified Rhizobiales	unclassified Rhizobiales (miscellaneous)	Rhizobiales bacterium NRL2
35	Bacteria	Proteobacteria	Alphaproteobacteria	Rhizobiales	Rhodobiaceae	Lutibaculum	Lutibaculum baratangense
35	Bacteria	PVC group	Verrucomicrobia	Verrucomicrobiae	Verrucomicrobiales	Verrucomicrobiaceae	Verrucomicrobium
35	Bacteria	FCB group	Bacteroidetes/Chlorobi group	Bacteroidetes	Bacteroidia	Bacteroidales	Rikenellaceae
35	ssDNA viruses	Microviridae	Gokushovirinae	unclassified Gokushovirinae	Gokushovirus WZ-2015a		
34	Bacteria	Proteobacteria	delta/epsilon subdivisions	Deltaproteobacteria	Desulfovibrionales	Desulfovibrionaceae	Desulfovibrio
34	Bacteria	Proteobacteria	Gammaproteobacteria	Orbales	Orbaceae	Gilliamella	Gilliamella apicola
34	Bacteria	Proteobacteria	Gammaproteobacteria	Chromatiales	Ectothiorhodospiraceae	Thioalkalivibrio	
34	Bacteria	Proteobacteria	Betaproteobacteria	Rhodocyclales	Rhodocyclaceae	unclassified Rhodocyclaceae	Rhodocyclaceae bacterium UTPRO2
34	Bacteria	Proteobacteria	Betaproteobacteria	Nitrosomonadales	Nitrosomonadaceae	Nitrosomonas	
34	Bacteria	Proteobacteria	Betaproteobacteria	Methylophilales	Methylophilaceae	Methylovorus	
34	Bacteria	Proteobacteria	Betaproteobacteria	Burkholderiales	Comamonadaceae	Comamonas	Comamonas terrae
34	Bacteria	Proteobacteria	Alphaproteobacteria	unclassified Alphaproteobacteria	unclassified Alphaproteobacteria (miscellaneous)	alpha proteobacterium AAP81b	
34	Bacteria	Proteobacteria	Alphaproteobacteria	Sphingomonadales	Sphingomonadaceae	Sphingomonas	Sphingomonas sp. YR710
34	Bacteria	Proteobacteria	Alphaproteobacteria	Sphingomonadales	Sphingomonadaceae	Sandarakinorhabdus	Sandarakinorhabdus sp. AAP62
34	Bacteria	Proteobacteria	Alphaproteobacteria	Rhodobacterales	Rhodobacteraceae	Paracoccus	Paracoccus chinensis
34	Bacteria	Proteobacteria	Alphaproteobacteria	Rhodobacterales	Rhodobacteraceae	Loktanella	Loktanella fryxellensis
34	Bacteria	Proteobacteria	Alphaproteobacteria	Rhodobacterales	Rhodobacteraceae	Albidovulum	Albidovulum xiamenense
34	Bacteria	Proteobacteria	Alphaproteobacteria	Rhizobiales	unclassified Rhizobiales	unclassified Rhizobiales (miscellaneous)	Rhizobiales bacterium 64-17
34	Bacteria	Proteobacteria	Alphaproteobacteria	Rhizobiales	Hyphomicrobiaceae	Hyphomicrobium	Hyphomicrobium denitrificans
34	Bacteria	Proteobacteria	Alphaproteobacteria	Rhizobiales	Bradyrhizobiaceae	Afipia	Afipia sp. P52-10
34	Bacteria	Nitrospirae	unclassified Nitrospirae				
34	Bacteria	Acidobacteria	Solibacteres	Solibacterales	Solibacteraceae	Candidatus Solibacter	Candidatus Solibacter usitatus
33	Bacteria	Proteobacteria	delta/epsilon subdivisions	Deltaproteobacteria	Myxococcales	Sorangiineae	Polyangiaceae
33	Bacteria	Proteobacteria	delta/epsilon subdivisions	Deltaproteobacteria	Desulfovibrionales	Desulfovibrionaceae	Desulfovibrio
33	Bacteria	Proteobacteria	delta/epsilon subdivisions	Deltaproteobacteria	Desulfovibrionales	Desulfomicrobiaceae	Desulfomicrobium
33	Bacteria	Proteobacteria	delta/epsilon subdivisions	Deltaproteobacteria	Desulfovibrionales	Desulfomicrobiaceae	Desulfomicrobium
33	Bacteria	Proteobacteria	Gammaproteobacteria	unclassified Gammaproteobacteria	unclassified Gammaproteobacteria (miscellaneous)	Gammaproteobacteria bacterium RIFCSPLOWO2_02_FULL_61_13	
33	Bacteria	Proteobacteria	Gammaproteobacteria	unclassified Gammaproteobacteria	unclassified Gammaproteobacteria (miscellaneous)	Gammaproteobacteria bacterium RIFCSPHIGHO2_12_FULL_63_22	
33	Bacteria	Proteobacteria	Gammaproteobacteria	Xanthomonadales	Xanthomonadaceae	Stenotrophomonas	Stenotrophomonas maltophilia group
33	Bacteria	Proteobacteria	Gammaproteobacteria	Pseudomonadales	Pseudomonadaceae	Pseudomonas	Pseudomonas fluorescens group
33	Bacteria	Proteobacteria	Gammaproteobacteria	Alteromonadales	Shewanellaceae	Shewanella	Shewanella putrefaciens
33	Bacteria	Proteobacteria	Betaproteobacteria	Rhodocyclales	unclassified Rhodocyclales	Rhodocyclales bacterium RIFCSPLOWO2_02_FULL_63_24	
33	Bacteria	Proteobacteria	Betaproteobacteria	Rhodocyclales	Rhodocyclaceae	Dechloromonas	Dechloromonas agitata
33	Bacteria	Proteobacteria	Betaproteobacteria	Burkholderiales	unclassified Burkholderiales	unclassified Burkholderiales (miscellaneous)	Burkholderiales bacterium RIFCSPLOWO2_02_FULL_66_35
33	Bacteria	Proteobacteria	Betaproteobacteria	Burkholderiales	Burkholderiaceae	Polynucleobacter	
33	Bacteria	Proteobacteria	Alphaproteobacteria	unclassified Alphaproteobacteria	unclassified Alphaproteobacteria (miscellaneous)	alpha proteobacterium BAL199	
33	Bacteria	Proteobacteria	Alphaproteobacteria	Sphingomonadales	Sphingomonadaceae	Sphingobium	Sphingobium chlorophenolicum
33	Bacteria	Proteobacteria	Alphaproteobacteria	Sphingomonadales	Erythrobacteraceae	Croceicoccus	
33	Bacteria	Proteobacteria	Alphaproteobacteria	Rhodobacterales	Rhodobacteraceae	Ruegeria	Ruegeria pomeroyi
33	Bacteria	Proteobacteria	Alphaproteobacteria	Rhodobacterales	Rhodobacteraceae	Roseovarius	Roseovarius azorensis
33	Bacteria	Proteobacteria	Alphaproteobacteria	Rhodobacterales	Rhodobacteraceae	Roseibaca	Roseibaca calidilacus
33	Bacteria	Proteobacteria	Alphaproteobacteria	Rhodobacterales	Rhodobacteraceae	Paracoccus	Paracoccus halophilus
33	Bacteria	Proteobacteria	Alphaproteobacteria	Rhodobacterales	Rhodobacteraceae	Marivita	Marivita cryptomonadis
33	Bacteria	Proteobacteria	Alphaproteobacteria	Rhodobacterales	Rhodobacteraceae	Marinovum	Marinovum algicola
33	Bacteria	Proteobacteria	Alphaproteobacteria	Rhodobacterales	Rhodobacteraceae	Aestuariivita	Aestuariivita boseongensis
33	Bacteria	Nitrospirae	Nitrospira	Nitrospirales	Nitrospiraceae	Nitrospira	Nitrospira sp. ND1
33	Bacteria	Nitrospirae	Nitrospira	Nitrospirales	Nitrospiraceae	Nitrospira	Nitrospira japonica
33	Bacteria	FCB group	Gemmatimonadetes	unclassified Gemmatimonadetes			
32	Bacteria	Proteobacteria	delta/epsilon subdivisions	Deltaproteobacteria	Myxococcales	Sorangiineae	Polyangiaceae
32	Bacteria	Proteobacteria	Gammaproteobacteria	unclassified Gammaproteobacteria	sulfur-oxidizing symbionts	Solemya velum gill symbiont	
32	Bacteria	Proteobacteria	Betaproteobacteria	Nitrosomonadales	Nitrosomonadaceae	Nitrosospira	
32	Bacteria	Proteobacteria	Betaproteobacteria	Burkholderiales	unclassified Burkholderiales	Burkholderiales Genera incertae sedis	Rhizobacter
32	Bacteria	Proteobacteria	Betaproteobacteria	Burkholderiales	Comamonadaceae	Acidovorax	Acidovorax sp. GW101-3H11
32	Bacteria	Proteobacteria	Alphaproteobacteria	Sphingomonadales	Sphingomonadaceae	Sphingopyxis	Sphingopyxis fribergensis
32	Bacteria	Proteobacteria	Alphaproteobacteria	Sphingomonadales	Sphingomonadaceae	Sphingopyxis	Sphingopyxis flava
32	Bacteria	Proteobacteria	Alphaproteobacteria	Sphingomonadales	Sphingomonadaceae	Sphingomonas	Sphingomonas sp. SRS2
32	Bacteria	Proteobacteria	Alphaproteobacteria	Sphingomonadales	Sphingomonadaceae	Sphingomonas	Sphingomonas panacis
32	Bacteria	Proteobacteria	Alphaproteobacteria	Sphingomonadales	Sphingomonadaceae	Citromicrobium	Citromicrobium sp. JLT1363
32	Bacteria	Proteobacteria	Alphaproteobacteria	Rhodospirillales	unclassified Rhodospirillales	unclassified Rhodospirillales (miscellaneous)	Rhodospirillales bacterium URHD0088
32	Bacteria	Proteobacteria	Alphaproteobacteria	Rhodobacterales	Rhodobacteraceae	Tropicimonas	Tropicimonas isoalkanivorans
32	Bacteria	Proteobacteria	Alphaproteobacteria	Rhodobacterales	Rhodobacteraceae	Ruegeria	Ruegeria marina
32	Bacteria	Proteobacteria	Alphaproteobacteria	Rhodobacterales	Rhodobacteraceae	Paracoccus	Paracoccus yeei
32	Bacteria	Proteobacteria	Alphaproteobacteria	Rhodobacterales	Rhodobacteraceae	Paracoccus	Paracoccus tibetensis
32	Bacteria	Proteobacteria	Alphaproteobacteria	Rhodobacterales	Rhodobacteraceae	Paracoccus	Paracoccus aminovorans
32	Bacteria	Proteobacteria	Alphaproteobacteria	Rhodobacterales	Rhodobacteraceae	Maritimibacter	Maritimibacter alkaliphilus
32	Bacteria	Proteobacteria	Alphaproteobacteria	Rhodobacterales	Rhodobacteraceae	Loktanella	Loktanella litorea
32	Bacteria	Proteobacteria	Alphaproteobacteria	Rhodobacterales	Rhodobacteraceae	Citreicella	Citreicella marina
32	Bacteria	Proteobacteria	Alphaproteobacteria	Rhizobiales	Rhodobiaceae	Parvibaculum	Parvibaculum lavamentivorans
32	Bacteria	Proteobacteria	Alphaproteobacteria	Rhizobiales	Phyllobacteriaceae	Mesorhizobium	Mesorhizobium loti
32	Bacteria	Proteobacteria	Alphaproteobacteria	Rhizobiales	Bradyrhizobiaceae	Salinarimonas	Salinarimonas rosea
32	Bacteria	Proteobacteria	Alphaproteobacteria	Rhizobiales	Beijerinckiaceae	Chelatococcus	
32	Bacteria	PVC group	Verrucomicrobia	Opitutae	Opitutales	Opitutaceae	
32	Bacteria	FCB group	Bacteroidetes/Chlorobi group	Bacteroidetes	unclassified Bacteroidetes	unclassified Bacteroidetes (miscellaneous)	Bacteroidetes bacterium GWF2_40_14
32	Bacteria	FCB group	Bacteroidetes/Chlorobi group	Bacteroidetes	Bacteroidia	Bacteroidales	unclassified Bacteroidales
32	ssDNA viruses	Microviridae	environmental samples	uncultured Gokushovirinae			
31	Bacteria	Proteobacteria	delta/epsilon subdivisions	Deltaproteobacteria	unclassified Deltaproteobacteria	unclassified Deltaproteobacteria (miscellaneous)	Deltaproteobacteria bacterium RBG_16_71_12
31	Bacteria	Proteobacteria	Gammaproteobacteria	Xanthomonadales	Xanthomonadaceae	Stenotrophomonas	
31	Bacteria	Proteobacteria	Gammaproteobacteria	Xanthomonadales	Xanthomonadaceae	Arenimonas	Arenimonas metalli
31	Bacteria	Proteobacteria	Betaproteobacteria	unclassified Betaproteobacteria	unclassified Betaproteobacteria (miscellaneous)	Betaproteobacteria bacterium CG2_30_68_42	
31	Bacteria	Proteobacteria	Betaproteobacteria	Burkholderiales	unclassified Burkholderiales	Burkholderiales Genera incertae sedis	Thiomonas
31	Bacteria	Proteobacteria	Betaproteobacteria	Burkholderiales	unclassified Burkholderiales	Burkholderiales Genera incertae sedis	Thiomonas
31	Bacteria	Proteobacteria	Betaproteobacteria	Burkholderiales	Alcaligenaceae	Azohydromonas	
31	Bacteria	Proteobacteria	Alphaproteobacteria	Sphingomonadales	Sphingomonadaceae	Sphingopyxis	Sphingopyxis sp. RIFCSPHIGHO2_12_FULL_65_19
31	Bacteria	Proteobacteria	Alphaproteobacteria	Sphingomonadales	Sphingomonadaceae	Sphingopyxis	Sphingopyxis granuli
31	Bacteria	Proteobacteria	Alphaproteobacteria	Sphingomonadales	Erythrobacteraceae	Porphyrobacter	Porphyrobacter sp. HL-46
31	Bacteria	Proteobacteria	Alphaproteobacteria	Rhodobacterales	Rhodobacteraceae	unclassified Rhodobacteraceae	Rhodobacteraceae bacterium HTCC2150
31	Bacteria	Proteobacteria	Alphaproteobacteria	Rhodobacterales	Rhodobacteraceae	Tranquillimonas	Tranquillimonas alkanivorans
31	Bacteria	Proteobacteria	Alphaproteobacteria	Rhodobacterales	Rhodobacteraceae	Roseovarius	Roseovarius aestuarii
31	Bacteria	Proteobacteria	Alphaproteobacteria	Rhodobacterales	Rhodobacteraceae	Pseudooceanicola	Pseudooceanicola nanhaiensis
31	Bacteria	Proteobacteria	Alphaproteobacteria	Rhodobacterales	Rhodobacteraceae	Loktanella	Loktanella sediminum
31	Bacteria	Proteobacteria	Alphaproteobacteria	Rhodobacterales	Rhodobacteraceae	Dinoroseobacter	Dinoroseobacter shibae
31	Bacteria	Proteobacteria	Alphaproteobacteria	Rhodobacterales	Rhodobacteraceae	Actibacterium	Actibacterium atlanticum
31	Bacteria	Proteobacteria	Alphaproteobacteria	Rhizobiales	Phyllobacteriaceae		
31	Bacteria	Proteobacteria	Alphaproteobacteria	Rhizobiales	Brucellaceae	Ochrobactrum	
31	Bacteria	PVC group	Planctomycetes	Planctomycetia	Planctomycetales	Planctomycetaceae	Rhodopirellula
31	Bacteria	FCB group	Gemmatimonadetes	Gemmatimonadetes	Gemmatimonadales	Gemmatimonadaceae	Gemmatimonas
31	Bacteria	FCB group	Bacteroidetes/Chlorobi group	Bacteroidetes	Bacteroidia	Bacteroidales	Porphyromonadaceae
31	Bacteria	FCB group	Bacteroidetes/Chlorobi group	Bacteroidetes	Bacteroidia	Bacteroidales	Bacteroidaceae
30	Bacteria	Proteobacteria	delta/epsilon subdivisions	Deltaproteobacteria	Bdellovibrionales	unclassified Bdellovibrionales	
30	Bacteria	Proteobacteria	Gammaproteobacteria	unclassified Gammaproteobacteria	Competibacteraceae	Candidatus Competibacter	Candidatus Competibacter denitrificans
30	Bacteria	Proteobacteria	Gammaproteobacteria	Pseudomonadales	Pseudomonadaceae	Pseudomonas	Pseudomonas syringae group
30	Bacteria	Proteobacteria	Gammaproteobacteria	Oceanospirillales	Alcanivoracaceae	Alcanivorax	
30	Bacteria	Proteobacteria	Betaproteobacteria	Burkholderiales	unclassified Burkholderiales	Burkholderiales Genera incertae sedis	Methylibium
30	Bacteria	Proteobacteria	Betaproteobacteria	Burkholderiales	Oxalobacteraceae	Noviherbaspirillum	Noviherbaspirillum massiliense
30	Bacteria	Proteobacteria	Betaproteobacteria	Burkholderiales	Oxalobacteraceae	Collimonas	
30	Bacteria	Proteobacteria	Betaproteobacteria	Burkholderiales	Comamonadaceae	Candidatus Symbiobacter	Candidatus Symbiobacter mobilis
30	Bacteria	Proteobacteria	Alphaproteobacteria	unclassified Alphaproteobacteria	unclassified Alphaproteobacteria (miscellaneous)	Alphaproteobacteria bacterium RIFCSPHIGHO2_12_FULL_63_12	
30	Bacteria	Proteobacteria	Alphaproteobacteria	Sphingomonadales	Sphingomonadaceae	Sphingopyxis	Sphingopyxis sp. QXT-31
30	Bacteria	Proteobacteria	Alphaproteobacteria	Sphingomonadales	Sphingomonadaceae	Novosphingobium	Novosphingobium resinovorum
30	Bacteria	Proteobacteria	Alphaproteobacteria	Sphingomonadales	Erythrobacteraceae	Porphyrobacter	Porphyrobacter sp. AAP82
30	Bacteria	Proteobacteria	Alphaproteobacteria	Sphingomonadales	Erythrobacteraceae	Porphyrobacter	Porphyrobacter sanguineus
30	Bacteria	Proteobacteria	Alphaproteobacteria	Sphingomonadales	Erythrobacteraceae	Porphyrobacter	
30	Bacteria	Proteobacteria	Alphaproteobacteria	Rhodospirillales	unclassified Rhodospirillales	unclassified Rhodospirillales (miscellaneous)	Rhodospirillales bacterium 70-18
30	Bacteria	Proteobacteria	Alphaproteobacteria	Rhodobacterales	Rhodobacteraceae	Tropicibacter	Tropicibacter naphthalenivorans
30	Bacteria	Proteobacteria	Alphaproteobacteria	Rhodobacterales	Rhodobacteraceae	Thioclava	Thioclava atlantica
30	Bacteria	Proteobacteria	Alphaproteobacteria	Rhodobacterales	Rhodobacteraceae	Sulfitobacter	Sulfitobacter brevis
30	Bacteria	Proteobacteria	Alphaproteobacteria	Rhodobacterales	Rhodobacteraceae	Sagittula	Sagittula stellata
30	Bacteria	Proteobacteria	Alphaproteobacteria	Rhodobacterales	Rhodobacteraceae	Pseudoruegeria	Pseudoruegeria sp. SK021
30	Bacteria	Proteobacteria	Alphaproteobacteria	Rhodobacterales	Rhodobacteraceae	Paracoccus	Paracoccus aminophilus
30	Bacteria	Proteobacteria	Alphaproteobacteria	Rhodobacterales	Rhodobacteraceae	Nioella	Nioella nitratireducens
30	Bacteria	Proteobacteria	Alphaproteobacteria	Rhodobacterales	Rhodobacteraceae	Loktanella	Loktanella atrilutea
30	Bacteria	Proteobacteria	Alphaproteobacteria	Rhodobacterales	Rhodobacteraceae	Albimonas	Albimonas pacifica
30	Bacteria	Proteobacteria	Alphaproteobacteria	Rhizobiales	Beijerinckiaceae	Methyloferula	Methyloferula stellata
30	Bacteria	PVC group	Planctomycetes				
30	Bacteria	FCB group	Bacteroidetes/Chlorobi group	Bacteroidetes	Saprospiria	Saprospirales	Haliscomenobacteraceae
30	Bacteria	FCB group	Bacteroidetes/Chlorobi group	Bacteroidetes	Bacteroidia	Bacteroidales	Porphyromonadaceae
30	Bacteria	FCB group	Bacteroidetes/Chlorobi group	Bacteroidetes	Bacteroidia	Bacteroidales	Porphyromonadaceae
30	Bacteria	Deferribacteres	Deferribacteres	Deferribacterales	Deferribacteraceae	Flexistipes	Flexistipes sinusarabici
29	Bacteria	Terrabacteria group	Actinobacteria	Acidimicrobiia	unclassified Acidimicrobiia		
29	Bacteria	Spirochaetes	unclassified Spirochaetes				
29	Bacteria	Proteobacteria	delta/epsilon subdivisions	Deltaproteobacteria	Myxococcales	Nannocystineae	Nannocystaceae
29	Bacteria	Proteobacteria	delta/epsilon subdivisions	Deltaproteobacteria	Desulfovibrionales	Desulfovibrionaceae	Desulfovibrio
29	Bacteria	Proteobacteria	delta/epsilon subdivisions	Deltaproteobacteria	Desulfovibrionales	Desulfovibrionaceae	Desulfovibrio
29	Bacteria	Proteobacteria	delta/epsilon subdivisions	Deltaproteobacteria	Desulfovibrionales		
29	Bacteria	Proteobacteria	Gammaproteobacteria	Xanthomonadales	Xanthomonadaceae	Arenimonas	Arenimonas oryziterrae
29	Bacteria	Proteobacteria	Gammaproteobacteria	Thiotrichales	Thiotrichaceae	Thiothrix	Thiothrix lacustris
29	Bacteria	Proteobacteria	Gammaproteobacteria	Alteromonadales	Shewanellaceae	Shewanella	Shewanella baltica
29	Bacteria	Proteobacteria	Gammaproteobacteria	Aeromonadales	Aeromonadaceae	Aeromonas	Aeromonas hydrophila
29	Bacteria	Proteobacteria	Betaproteobacteria	Methylophilales	Methylophilaceae	Methylophilus	Methylophilus rhizosphaerae
29	Bacteria	Proteobacteria	Betaproteobacteria	Ferrovales	Ferrovaceae	Ferrovum	
29	Bacteria	Proteobacteria	Betaproteobacteria	Burkholderiales	Oxalobacteraceae	Herbaspirillum	Herbaspirillum sp. TSA66
29	Bacteria	Proteobacteria	Betaproteobacteria	Burkholderiales	Burkholderiaceae	Polynucleobacter	Polynucleobacter sp. VK13
29	Bacteria	Proteobacteria	Betaproteobacteria	Burkholderiales	Burkholderiaceae	Lautropia	Lautropia mirabilis
29	Bacteria	Proteobacteria	Betaproteobacteria	Burkholderiales	Burkholderiaceae	Burkholderia	pseudomallei group
29	Bacteria	Proteobacteria	Betaproteobacteria	Burkholderiales	Alcaligenaceae	Derxia	Derxia gummosa
29	Bacteria	Proteobacteria	Alphaproteobacteria	Sphingomonadales	Sphingomonadaceae	Sphingomonas	Sphingomonas sp. Leaf412
29	Bacteria	Proteobacteria	Alphaproteobacteria	Sphingomonadales	Sphingomonadaceae	Sphingomonas	Sphingomonas sp. Leaf17
29	Bacteria	Proteobacteria	Alphaproteobacteria	Sphingomonadales	Erythrobacteraceae	Altererythrobacter	
29	Bacteria	Proteobacteria	Alphaproteobacteria	Rhodospirillales	Rhodospirillaceae	Magnetospirillum	
29	Bacteria	Proteobacteria	Alphaproteobacteria	Rhodobacterales	Rhodobacteraceae	Roseovarius	Roseovarius pacificus
29	Bacteria	Proteobacteria	Alphaproteobacteria	Rhodobacterales	Rhodobacteraceae	Roseovarius	Roseovarius atlanticus
29	Bacteria	Proteobacteria	Alphaproteobacteria	Rhodobacterales	Rhodobacteraceae	Ponticoccus	Ponticoccus sp. SJ5A-1
29	Bacteria	Proteobacteria	Alphaproteobacteria	Rhodobacterales	Rhodobacteraceae	Pannonibacter	Pannonibacter phragmitetus
29	Bacteria	Proteobacteria	Alphaproteobacteria	Rhodobacterales	Rhodobacteraceae	Octadecabacter	Octadecabacter temperatus
29	Bacteria	Proteobacteria	Alphaproteobacteria	Rhodobacterales	Rhodobacteraceae	Oceanicola	Oceanicola sp. HL-35
29	Bacteria	Proteobacteria	Alphaproteobacteria	Rhodobacterales	Rhodobacteraceae	Celeribacter	Celeribacter ethanolicus
29	Bacteria	Proteobacteria	Alphaproteobacteria	Rhodobacterales	Rhodobacteraceae	Aliiroseovarius	Aliiroseovarius sediminilitoris
29	Bacteria	Proteobacteria	Alphaproteobacteria	Rhizobiales	Xanthobacteraceae	Pseudolabrys	Pseudolabrys sp. Root1462
29	Bacteria	Proteobacteria	Alphaproteobacteria	Rhizobiales	Rhizobiaceae	Kaistia	Kaistia soli
29	Bacteria	Proteobacteria	Alphaproteobacteria	Rhizobiales	Methylocystaceae	Pleomorphomonas	Pleomorphomonas oryzae
29	Bacteria	PVC group	Verrucomicrobia	Verrucomicrobiae	Verrucomicrobiales	Akkermansiaceae	Akkermansia
29	Bacteria	PVC group	Planctomycetes	Planctomycetia	Planctomycetales	Planctomycetaceae	unclassified Planctomycetaceae

29	Bacteria	PVC group	Planctomycetes	Planctomycetia	Planctomycetales	Planctomycetaceae	unclassified Planctomycetaceae
29	Bacteria	PVC group	Planctomycetes	Planctomycetia	Planctomycetales	Planctomycetaceae	Planctopirus
29	Bacteria	PVC group	Planctomycetes	Planctomycetia	Planctomycetales	Isosphaeraceae	Singulisphaera
29	Bacteria	PVC group	Lentisphaerae	unclassified Lentisphaerae	unclassified Lentisphaerae (miscellaneous)	Lentisphaerae bacterium GWF2_52_8	
29	Bacteria	FCB group	Bacteroidetes/Chlorobi group	Bacteroidetes	Sphingobacteriia	Sphingobacteriales	Sphingobacteriaceae
28	Bacteria	unclassified Bacteria	Bacteria candidate phyla	Patescibacteria group	Parcubacteria group		
28	Bacteria	environmental samples	uncultured bacterium pDL136				
28	Bacteria	Terrabacteria group	Firmicutes	Clostridia	Clostridiales	Clostridiaceae	
28	Bacteria	Proteobacteria	delta/epsilon subdivisions	Deltaproteobacteria	Desulfovibrionales	Desulfovibrionaceae	Desulfovibrio
28	Bacteria	Proteobacteria	Gammaproteobacteria	Xanthomonadales	Xanthomonadaceae	Luteimonas	Luteimonas mephitis
28	Bacteria	Proteobacteria	Gammaproteobacteria	Legionellales	Legionellaceae	Legionella	
28	Bacteria	Proteobacteria	Gammaproteobacteria	Aeromonadales	Aeromonadaceae	Aeromonas	Aeromonas veronii
28	Bacteria	Proteobacteria	Betaproteobacteria	unclassified Betaproteobacteria	Candidatus Accumulibacter	Candidatus Accumulibacter aalborgensis	
28	Bacteria	Proteobacteria	Betaproteobacteria	Rhodocyclales	Rhodocyclaceae	Rugosibacter	Rugosibacter aromaticivorans
28	Bacteria	Proteobacteria	Betaproteobacteria	Burkholderiales	unclassified Burkholderiales	unclassified Burkholderiales (miscellaneous)	Burkholderiales bacterium RIFOXYD12_FULL_59_19
28	Bacteria	Proteobacteria	Betaproteobacteria	Burkholderiales	Oxalobacteraceae	unclassified Oxalobacteraceae	Oxalobacteraceae bacterium IMCC9480
28	Bacteria	Proteobacteria	Betaproteobacteria	Burkholderiales	Oxalobacteraceae	Collimonas	Collimonas arenae
28	Bacteria	Proteobacteria	Betaproteobacteria	Burkholderiales	Comamonadaceae	Comamonas	Comamonas granuli
28	Bacteria	Proteobacteria	Betaproteobacteria	Burkholderiales	Burkholderiaceae	Chitinimonas	Chitinimonas taiwanensis
28	Bacteria	Proteobacteria	Betaproteobacteria	Burkholderiales	Alcaligenaceae	Bordetella	
28	Bacteria	Proteobacteria	Alphaproteobacteria	Sphingomonadales	Sphingomonadaceae	Sphingobium	Sphingobium amiense
28	Bacteria	Proteobacteria	Alphaproteobacteria	Rhodobacterales	Rhodobacteraceae	unclassified Rhodobacteraceae	Rhodobacteraceae bacterium HLUCCO07
28	Bacteria	Proteobacteria	Alphaproteobacteria	Rhodobacterales	Rhodobacteraceae	unclassified Rhodobacteraceae	Rhodobacteraceae bacterium GWE1_64_9
28	Bacteria	Proteobacteria	Alphaproteobacteria	Rhodobacterales	Rhodobacteraceae	Poseidonocella	Poseidonocella sedimentorum
28	Bacteria	Proteobacteria	Alphaproteobacteria	Rhodobacterales	Rhodobacteraceae	Celeribacter	Celeribacter baekdonensis
28	Bacteria	Proteobacteria	Alphaproteobacteria	Rhodobacterales	Rhodobacteraceae	Aliiroseovarius	Aliiroseovarius crassostreae
28	Bacteria	Proteobacteria	Alphaproteobacteria	Rhizobiales	unclassified Rhizobiales	unclassified Rhizobiales (miscellaneous)	Rhizobiales bacterium MIMtkB18
28	Bacteria	Proteobacteria	Alphaproteobacteria	Rhizobiales	Rhizobiaceae	Rhizobium/Agrobacterium group	Rhizobium
28	Bacteria	Proteobacteria	Alphaproteobacteria	Rhizobiales	Rhizobiaceae	Kaistia	Kaistia granuli
28	Bacteria	Proteobacteria	Alphaproteobacteria	Rhizobiales	Phyllobacteriaceae	Mesorhizobium	Mesorhizobium alhagi
28	Bacteria	Proteobacteria	Alphaproteobacteria	Rhizobiales	Methylobacteriaceae	Microvirga	Microvirga massiliensis
28	Bacteria	PVC group	Planctomycetes	Planctomycetia	Planctomycetales	Planctomycetaceae	Rhodopirellula
28	Bacteria	FCB group	Bacteroidetes/Chlorobi group	Bacteroidetes	unclassified Bacteroidetes	unclassified Bacteroidetes (miscellaneous)	Bacteroidetes bacterium 41-46
28	Bacteria	FCB group	Bacteroidetes/Chlorobi group	Bacteroidetes	Chitinophagia	Chitinophagales	Chitinophagaceae
28	Bacteria	FCB group	Bacteroidetes/Chlorobi group	Bacteroidetes	Bacteroidia	Bacteroidales	Porphyromonadaceae
28	Bacteria	FCB group	Bacteroidetes/Chlorobi group	Bacteroidetes	Bacteroidia	Bacteroidales	Odoribacteraceae
28	Bacteria	FCB group	Bacteroidetes/Chlorobi group	Bacteroidetes	Bacteroidia	Bacteroidales	Bacteroidaceae
27	Eukaryota	Glaucocystophyceae	Cyanophoraceae	Cyanophora	Cyanophora paradoxa		
27	Bacteria	Spirochaetes	Spirochaetia	Spirochaetales	Spirochaetaceae	Treponema	Treponema primitia
27	Bacteria	Proteobacteria	delta/epsilon subdivisions	Deltaproteobacteria	Desulfovibrionales	Desulfovibrionaceae	Desulfovibrio
27	Bacteria	Proteobacteria	delta/epsilon subdivisions	Deltaproteobacteria	Desulfobacterales	Desulfobacteraceae	unclassified Desulfobacteraceae
27	Bacteria	Proteobacteria	Gammaproteobacteria	Pseudomonadales	Pseudomonadaceae	Pseudomonas	Pseudomonas stutzeri group
27	Bacteria	Proteobacteria	Gammaproteobacteria	Nevskiales	Sinobacteraceae	Solimonas	Solimonas aquatica
27	Bacteria	Proteobacteria	Gammaproteobacteria	Chromatiales	unclassified Chromatiales	Chromatiales bacterium USCg_Taylor	
[illegible]	[illegible]	[illegible]	[illegible]	[illegible]	[illegible]	[illegible]	[illegible]

19	Bacteria	Proteobacteria	Alphaproteobacteria	Rhizobiales	Hyphomicrobiaceae	Devosia	Devosia geojensis
19	Bacteria	Proteobacteria	Alphaproteobacteria	Rhizobiales	Aurantimonadaceae	Martelella	Martelella mediterranea
19	Bacteria	Proteobacteria	Alphaproteobacteria	Rhizobiales	Aurantimonadaceae	Aurantimonas	
19	Bacteria	Proteobacteria	Alphaproteobacteria	Caulobacterales	Caulobacteraceae	Caulobacter	Caulobacter vibrioides
19	Bacteria	PVC group	Planctomycetes	unclassified Planctomycetes	Planctomycetes bacterium RBG_13_63_9		
19	Bacteria	PVC group	Planctomycetes	Planctomycetia	Planctomycetales	Planctomycetaceae	Rhodopirellula
19	Bacteria	Nitrospirae	Nitrospira	Nitrospirales	Nitrospiraceae	Nitrospira	Nitrospira sp. SCGC AG-212-E16
19	Bacteria	FCB group	Bacteroidetes/Chlorobi group	Bacteroidetes	Bacteroidia	Marinilabiliales	Prolixibacteraceae
19	Bacteria	FCB group	Bacteroidetes/Chlorobi group	Bacteroidetes	Bacteroidia	Bacteroidales	Rikenellaceae
19	Bacteria	FCB group	Bacteroidetes/Chlorobi group	Bacteroidetes	Bacteroidia	Bacteroidales	Porphyromonadaceae
19	Bacteria	Acidobacteria	Acidobacteriia	Acidobacteriales	Acidobacteriaceae	unclassified Acidobacteriaceae	Acidobacteriaceae bacterium KBS 96
18	Eukaryota	Heterolobosea	Schizopyrenida	Vahlkampfiidae	Naegleria		
18	Bacteria	unclassified Bacteria	unclassified Bacteria (miscellaneous)	bacterium A52C2			
18	Bacteria	unclassified Bacteria	unclassified Bacteria (miscellaneous)				
18	Bacteria	Terrabacteria group	Firmicutes	Clostridia	Clostridiales	Ruminococcaceae	unclassified Ruminococcaceae
18	Bacteria	Terrabacteria group	Firmicutes	Clostridia	Clostridiales	Ruminococcaceae	Acetanaerobacterium
18	Bacteria	Terrabacteria group	Firmicutes	Clostridia	Clostridiales	Lachnospiraceae	Butyrivibrio
18	Bacteria	Terrabacteria group	Cyanobacteria/Melainabacteria group	Cyanobacteria	Synechococcales	Leptolyngbyaceae	Leptolyngbya
18	Bacteria	Terrabacteria group	Chloroflexi	unclassified Chloroflexi	unclassified Chloroflexi (miscellaneous)	Chloroflexi bacterium RBG_16_72_14	
18	Bacteria	Terrabacteria group	Chloroflexi	unclassified Chloroflexi	Kouleothrix	Kouleothrix aurantiaca	
18	Bacteria	Terrabacteria group	Chloroflexi	Anaerolineae	unclassified Anaerolineae	Anaerolineae bacterium SG8_19	
18	Bacteria	Terrabacteria group	Actinobacteria	Thermoleophilia	Solirubrobacterales	Conexibacteraceae	Conexibacter
18	Bacteria	Terrabacteria group	Actinobacteria	Actinobacteria	Corynebacteriales		
18	Bacteria	Terrabacteria group	Actinobacteria	Acidimicrobiia	Acidimicrobiales	Acidimicrobiaceae	Ilumatobacter
18	Bacteria	Synergistetes	Synergistia	Synergistales	Synergistaceae	Dethiosulfovibrio	Dethiosulfovibrio salsuginis
18	Bacteria	Spirochaetes	unclassified Spirochaetes	Spirochaetes bacterium RBG_13_68_11			
18	Bacteria	Proteobacteria	delta/epsilon subdivisions	Deltaproteobacteria	unclassified Deltaproteobacteria	unclassified Deltaproteobacteria (miscellaneous)	delta proteobacterium MLS_D
18	Bacteria	Proteobacteria	delta/epsilon subdivisions	Deltaproteobacteria	Desulfovibrionales	Desulfovibrionaceae	unclassified Desulfovibrionaceae
18	Bacteria	Proteobacteria	delta/epsilon subdivisions	Deltaproteobacteria	Desulfovibrionales	Desulfovibrionaceae	Desulfovibrio
18	Bacteria	Proteobacteria	delta/epsilon subdivisions	Deltaproteobacteria	Desulfobacterales	Desulfobulbaceae	Desulfobulbus
18	Bacteria	Proteobacteria	delta/epsilon subdivisions	Deltaproteobacteria	Desulfobacterales	Desulfobacteraceae	Desulfatirhabdium
18	Bacteria	Proteobacteria	Gammaproteobacteria	Xanthomonadales	unclassified Xanthomonadales	Xanthomonadales bacterium 63-13	
18	Bacteria	Proteobacteria	Gammaproteobacteria	Xanthomonadales	Xanthomonadaceae	Xanthomonas	Xanthomonas sp. Mitacek01
18	Bacteria	Proteobacteria	Gammaproteobacteria	Xanthomonadales	Xanthomonadaceae	Lysobacter	Lysobacter enzymogenes
18	Bacteria	Proteobacteria	Gammaproteobacteria	Thiotrichales	unclassified Thiotrichales	unclassified Thiotrichales (miscellaneous)	Thiotrichales bacterium HS_08
18	Bacteria	Proteobacteria	Gammaproteobacteria	Enterobacterales	Yersiniaceae	Rahnella	
18	Bacteria	Proteobacteria	Gammaproteobacteria	Enterobacterales	Thorselliaceae	Thorsellia	Thorsellia anophelis
18	Bacteria	Proteobacteria	Gammaproteobacteria	Enterobacterales	Enterobacteriaceae	Klebsiella	Klebsiella pneumoniae
18	Bacteria	Proteobacteria	Betaproteobacteria	Rhodocyclales	Rhodocyclaceae	Azoarcus	Azoarcus toluclasticus
18	Bacteria	Proteobacteria	Betaproteobacteria	Nitrosomonadales	Nitrosomonadaceae	Nitrosospira	Nitrosospira briensis
18	Bacteria	Proteobacteria	Betaproteobacteria	Neisseriales	Neisseriaceae	Vitreoscilla	Vitreoscilla sp. SN6
18	Bacteria	Proteobacteria	Betaproteobacteria	Neisseriales	Chromobacteriaceae	Gulbenkiania	
18	Bacteria	Proteobacteria	Betaproteobacteria	Hydrogenophilales	unclassified Hydrogenophilales	Hydrogenophilales bacterium RIFOXYD1_FULL_62_11	
18	Bacteria	Proteobacteria	Betaproteobacteria	Burkholderiales	Oxalobacteraceae	Herbaspirillum	Herbaspirillum sp. YR522
18	Bacteria	Proteobacteria	Betaproteobacteria	Burkholderiales	Comamonadaceae	Comamonas	Comamonas badia
18	Bacteria	Proteobacteria	Betaproteobacteria	Burkholderiales	Comamonadaceae	Acidovorax	Acidovorax temperans
18	Bacteria	Proteobacteria	Betaproteobacteria	Burkholderiales	Burkholderiaceae	Paraburkholderia	
18	Bacteria	Proteobacteria	Betaproteobacteria	Burkholderiales	Burkholderiaceae	Burkholderia	Burkholderia sp. JPY347
18	Bacteria	Proteobacteria	Alphaproteobacteria	Sphingomonadales	Sphingomonadaceae	Sphingomonas	Sphingomonas mali
18	Bacteria	Proteobacteria	Alphaproteobacteria	Rhodospirillales	Acetobacteraceae	Roseomonas	Roseomonas rosea
18	Bacteria	Proteobacteria	Alphaproteobacteria	Rhodospirillales	Acetobacteraceae	Roseomonas	
18	Bacteria	Proteobacteria	Alphaproteobacteria	Rhodospirillales			
18	Bacteria	Proteobacteria	Alphaproteobacteria	Rhodobacterales	Rhodobacteraceae	unclassified Rhodobacteraceae	Rhodobacteraceae bacterium PD-2
18	Bacteria	Proteobacteria	Alphaproteobacteria	Rhodobacterales	Rhodobacteraceae	Tropicibacter	Tropicibacter multivorans
18	Bacteria	Proteobacteria	Alphaproteobacteria	Rhodobacterales	Rhodobacteraceae	Thioclava	Thioclava pacifica
18	Bacteria	Proteobacteria	Alphaproteobacteria	Rhodobacterales	Rhodobacteraceae	Thalassobacter	
18	Bacteria	Proteobacteria	Alphaproteobacteria	Rhodobacterales	Rhodobacteraceae	Roseovarius	Roseovarius tolerans
18	Bacteria	Proteobacteria	Alphaproteobacteria	Rhodobacterales	Rhodobacteraceae	Roseovarius	Roseovarius sp. MCTG156(2b)
18	Bacteria	Proteobacteria	Alphaproteobacteria	Rhodobacterales	Rhodobacteraceae	Roseovarius	Roseovarius nubinhibens
18	Bacteria	Proteobacteria	Alphaproteobacteria	Rhodobacterales	Rhodobacteraceae	Pseudooceanicola	Pseudooceanicola atlanticus
18	Bacteria	Proteobacteria	Alphaproteobacteria	Rhodobacterales	Rhodobacteraceae	Planktomarina	Planktomarina temperata
18	Bacteria	Proteobacteria	Alphaproteobacteria	Rhodobacterales	Rhodobacteraceae	Oceanicola	Oceanicola sp. S124
18	Bacteria	Proteobacteria	Alphaproteobacteria	Rhodobacterales	Rhodobacteraceae	Nereida	Nereida ignava
18	Bacteria	Proteobacteria	Alphaproteobacteria	Rhodobacterales	Rhodobacteraceae	Marivita	Marivita geojedonensis
18	Bacteria	Proteobacteria	Alphaproteobacteria	Rhodobacterales	Rhodobacteraceae	Loktanella	Loktanella pyoseonensis
18	Bacteria	Proteobacteria	Alphaproteobacteria	Rhodobacterales	Rhodobacteraceae	Citreicella	
18	Bacteria	Proteobacteria	Alphaproteobacteria	Rhizobiales	Xanthobacteraceae	Pseudoxanthobacter	Pseudoxanthobacter soli
18	Bacteria	Proteobacteria	Alphaproteobacteria	Rhizobiales	Phyllobacteriaceae	Mesorhizobium	Mesorhizobium sp. YR577
18	Bacteria	Proteobacteria	Alphaproteobacteria	Rhizobiales	Phyllobacteriaceae	Aliihoeflea	Aliihoeflea sp. 2WW
18	Bacteria	Proteobacteria	Alphaproteobacteria	Rhizobiales	Methylocystaceae	Methylocystis	
18	Bacteria	Proteobacteria	Alphaproteobacteria	Rhizobiales	Hyphomicrobiaceae	Blastochloris	Blastochloris viridis
18	Bacteria	Proteobacteria	Alphaproteobacteria	Rhizobiales	Bradyrhizobiaceae	Bradyrhizobium	Bradyrhizobium japonicum
18	Bacteria	Proteobacteria	Alphaproteobacteria	Rhizobiales	Bradyrhizobiaceae	Afipia	
18	Bacteria	Proteobacteria	Alphaproteobacteria	Magnetococcales	Magnetococcaceae	Magnetofaba	Magnetofaba australis
18	Bacteria	Proteobacteria	Alphaproteobacteria	Caulobacterales			
18	Bacteria	PVC group	Verrucomicrobia	unclassified Verrucomicrobia	unclassified Verrucomicrobia (miscellaneous)		
18	Bacteria	PVC group	Verrucomicrobia	Opitutae	Opitutales	Opitutaceae	unclassified Opitutaceae
18	Bacteria	PVC group	Planctomycetes	Phycisphaerae	unclassified Phycisphaerae	Phycisphaerae bacterium ST-NAGAB-D1	
18	Bacteria	PVC group	Lentisphaerae	unclassified Lentisphaerae	unclassified Lentisphaerae (miscellaneous)	Lentisphaerae bacterium GWF2_57_35	
18	Bacteria	Nitrospirae	Nitrospira	Nitrospirales	Nitrospiraceae	Nitrospira	Nitrospira defluvii
18	Bacteria	FCB group	Fibrobacteres	Chitinispirillia	Chitinispirillales	Chitinispirillaceae	Chitinispirillum
18	Bacteria	FCB group	Bacteroidetes/Chlorobi group	Bacteroidetes	unclassified Bacteroidetes	unclassified Bacteroidetes (miscellaneous)	Bacteroidetes bacterium OLB9
18	Bacteria	FCB group	Bacteroidetes/Chlorobi group	Bacteroidetes	Sphingobacteriia	Sphingobacteriales	Sphingobacteriaceae
18	Bacteria	FCB group	Bacteroidetes/Chlorobi group	Bacteroidetes	Sphingobacteriia	Sphingobacteriales	Sphingobacteriaceae
18	Bacteria	FCB group	Bacteroidetes/Chlorobi group	Bacteroidetes	Bacteroidia	Marinilabiliales	Salinivirgaceae
18	Bacteria	FCB group	Bacteroidetes/Chlorobi group	Bacteroidetes	Bacteroidia	Bacteroidales	unclassified Bacteroidales
18	Bacteria	FCB group	Bacteroidetes/Chlorobi group	Bacteroidetes	Bacteroidia	Bacteroidales	Rikenellaceae
18	Bacteria	FCB group	Bacteroidetes/Chlorobi group	Bacteroidetes	Bacteroidia	Bacteroidales	Porphyromonadaceae
18	Bacteria	FCB group	Bacteroidetes/Chlorobi group	Bacteroidetes	Bacteroidia	Bacteroidales	Bacteroidaceae
18	Bacteria	Acidobacteria	unclassified Acidobacteria	unclassified Acidobacteria (miscellaneous)	Acidobacteria bacterium 13_2_20CM_2_66_4		
18	Bacteria	Acidobacteria	unclassified Acidobacteria	unclassified Acidobacteria (miscellaneous)	Acidobacteria bacterium 13_1_20CM_3_53_8		
17	Eukaryota	Alveolata	Ciliophora	Intramacronucleata	Oligohymenophorea	Hymenostomatida	Tetrahymenina
17	Bacteria	unclassified Bacteria	Bacteria candidate phyla	Candidatus Raymondbacteria			
17	Bacteria	unclassified Bacteria	Bacteria candidate phyla	Candidatus Handelsmanbacteria	Candidatus Handelsmanbacteria bacterium RIFCSPLOWO2_12_FULL_64_10		
17	Bacteria	Terrabacteria group	Firmicutes	Clostridia	Clostridiales	Ruminococcaceae	Ruminococcus
17	Bacteria	Terrabacteria group	Firmicutes	Clostridia	Clostridiales	Ruminococcaceae	Anaerotruncus
17	Bacteria	Terrabacteria group	Chloroflexi	Anaerolineae	Anaerolineales	Anaerolineaceae	Thermanaerothrix
17	Bacteria	Terrabacteria group	Armatimonadetes	unclassified Armatimonadetes	Armatimonadetes bacterium CG2_30_66_41		
17	Bacteria	Terrabacteria group	Actinobacteria	Actinobacteria	Micrococcales		
17	Bacteria	Terrabacteria group	Actinobacteria	Actinobacteria	Corynebacteriales	Corynebacteriaceae	Corynebacterium
17	Bacteria	Proteobacteria	unclassified Proteobacteria	unclassified Proteobacteria (miscellaneous)	Proteobacteria bacterium SG_bin4		
17	Bacteria	Proteobacteria	delta/epsilon subdivisions	Deltaproteobacteria	Myxococcales	unclassified Myxococcales	Enhygromyxa
17	Bacteria	Proteobacteria	delta/epsilon subdivisions	Deltaproteobacteria	Myxococcales	Sorangiineae	Polyangiaceae
17	Bacteria	Proteobacteria	delta/epsilon subdivisions	Deltaproteobacteria	Myxococcales	Cystobacterineae	Archangiaceae
17	Bacteria	Proteobacteria	delta/epsilon subdivisions	Deltaproteobacteria	Desulfuromonadales	Desulfuromonadaceae	Desulfuromonas
17	Bacteria	Proteobacteria	delta/epsilon subdivisions	Deltaproteobacteria	Desulfobacterales	Desulfobulbaceae	unclassified Desulfobulbaceae
17	Bacteria	Proteobacteria	delta/epsilon subdivisions	Deltaproteobacteria	Desulfarculales	Desulfarculaceae	Dethiosulfatarculus
17	Bacteria	Proteobacteria	Gammaproteobacteria	Xanthomonadales	Xanthomonadaceae	Luteimonas	Luteimonas tolerans
17	Bacteria	Proteobacteria	Gammaproteobacteria	Xanthomonadales	Xanthomonadaceae	Luteimonas	Luteimonas sp. J29
17	Bacteria	Proteobacteria	Gammaproteobacteria	Xanthomonadales	Rhodanobacteraceae	Mizugakiibacter	Mizugakiibacter sediminis
17	Bacteria	Proteobacteria	Gammaproteobacteria	Xanthomonadales	Rhodanobacteraceae	Aquimonas	Aquimonas voraii
17	Bacteria	Proteobacteria	Gammaproteobacteria	Thiotrichales	Thiotrichaceae	Leucothrix	Leucothrix mucor
17	Bacteria	Proteobacteria	Gammaproteobacteria	Pseudomonadales	Pseudomonadaceae	Pseudomonas	Pseudomonas pohangensis
17	Bacteria	Proteobacteria	Gammaproteobacteria	Pseudomonadales	Pseudomonadaceae	Pseudomonas	Pseudomonas aeruginosa group
17	Bacteria	Proteobacteria	Gammaproteobacteria	Nevskiales	Sinobacteraceae	Hydrocarboniphaga	Hydrocarboniphaga effusa
17	Bacteria	Proteobacteria	Gammaproteobacteria	Chromatiales	Ectothiorhodospiraceae	Acidihalobacter	Acidihalobacter prosperus
17	Bacteria	Proteobacteria	Gammaproteobacteria	Cellvibrionales	Halieaceae	Luminiphilus	Luminiphilus syltensis
17	Bacteria	Proteobacteria	Gammaproteobacteria	Alteromonadales	Colwelliaceae	Thalassotalea	Thalassotalea agarivorans
17	Bacteria	Proteobacteria	Gammaproteobacteria	Aeromonadales	Aeromonadaceae	Aeromonas	Aeromonas encheleia
17	Bacteria	Proteobacteria	Candidatus Muproteobacteria	Candidatus Muproteobacteria bacterium RBG_16_60_9			
17	Bacteria	Proteobacteria	Betaproteobacteria	unclassified Betaproteobacteria	unclassified Betaproteobacteria (miscellaneous)	Betaproteobacteria bacterium RIFCSPLOWO2_02_FULL_67_19	
17	Bacteria	Proteobacteria	Betaproteobacteria	Rhodocyclales	Rhodocyclaceae	Dechloromonas	
17	Bacteria	Proteobacteria	Betaproteobacteria	Rhodocyclales	Rhodocyclaceae	Azoarcus	Azoarcus sp. CIB
17	Bacteria	Proteobacteria	Betaproteobacteria	Nitrosomonadales	Nitrosomonadaceae	Nitrosomonas	Nitrosomonas eutropha
17	Bacteria	Proteobacteria	Betaproteobacteria	Neisseriales	Chromobacteriaceae	Aquaspirillum	Aquaspirillum sp. LM1
17	Bacteria	Proteobacteria	Betaproteobacteria	Burkholderiales	Oxalobacteraceae	Massilia	Massilia sp. Leaf139
17	Bacteria	Proteobacteria	Betaproteobacteria	Burkholderiales	Comamonadaceae	Variovorax	Variovorax sp. Root411
17	Bacteria	Proteobacteria	Betaproteobacteria	Burkholderiales	Comamonadaceae	Variovorax	Variovorax sp. EL159
17	Bacteria	Proteobacteria	Betaproteobacteria	Burkholderiales	Comamonadaceae	Acidovorax	Acidovorax sp. SD340
17	Bacteria	Proteobacteria	Betaproteobacteria	Burkholderiales	Comamonadaceae	Acidovorax	Acidovorax sp. JS42
17	Bacteria	Proteobacteria	Betaproteobacteria	Burkholderiales	Comamonadaceae	Acidovorax	Acidovorax citrulli
17	Bacteria	Proteobacteria	Betaproteobacteria	Burkholderiales	Alcaligenaceae	Bordetella	Bordetella bronchialis
17	Bacteria	Proteobacteria	Alphaproteobacteria	Sphingomonadales	Sphingomonadaceae	Sphingomonas	Sphingomonas taxi
17	Bacteria	Proteobacteria	Alphaproteobacteria	Sphingomonadales	Sphingomonadaceae	Sphingobium	Sphingobium sp. AP49
17	Bacteria	Proteobacteria	Alphaproteobacteria	Sphingomonadales	Sphingomonadaceae	Sphingobium	Sphingobium chungbukense
17	Bacteria	Proteobacteria	Alphaproteobacteria	Rhodospirillales	unclassified Rhodospirillales	Elioraea	Elioraea tepidiphila
17	Bacteria	Proteobacteria	Alphaproteobacteria	Rhodospirillales	Rhodospirillaceae		
17	Bacteria	Proteobacteria	Alphaproteobacteria	Rhodospirillales	Acetobacteraceae	Acidisphaera	Acidisphaera rubrifaciens
17	Bacteria	Proteobacteria	Alphaproteobacteria	Rhodobacterales	Rhodobacteraceae	Yangia	Yangia sp. CCB-MM3
17	Bacteria	Proteobacteria	Alphaproteobacteria	Rhodobacterales	Rhodobacteraceae	Shimia	Shimia marina
17	Bacteria	Proteobacteria	Alphaproteobacteria	Rhodobacterales	Rhodobacteraceae	Sedimentitalea	Sedimentitalea nanhaiensis
17	Bacteria	Proteobacteria	Alphaproteobacteria	Rhodobacterales	Rhodobacteraceae	Roseobacter	
17	Bacteria	Proteobacteria	Alphaproteobacteria	Rhodobacterales	Rhodobacteraceae	Roseivivax	Roseivivax jejudonensis
17	Bacteria	Proteobacteria	Alphaproteobacteria	Rhodobacterales	Rhodobacteraceae	Planktotalea	Planktotalea frisia
17	Bacteria	Proteobacteria	Alphaproteobacteria	Rhodobacterales	Rhodobacteraceae	Litoreibacter	Litoreibacter ascidiaceicola
17	Bacteria	Proteobacteria	Alphaproteobacteria	Rhodobacterales	Rhodobacteraceae	Leisingera	
17	Bacteria	Proteobacteria	Alphaproteobacteria	Rhodobacterales	Rhodobacteraceae	Labrenzia	Labrenzia alba
17	Bacteria	Proteobacteria	Alphaproteobacteria	Rhizobiales	Rhodobiaceae	Candidatus Phaeomarinobacter	Candidatus Phaeomarinobacter ectocarpi
17	Bacteria	Proteobacteria	Alphaproteobacteria	Rhizobiales	Rhizobiaceae	Rhizobium/Agrobacterium group	Rhizobium
17	Bacteria	Proteobacteria	Alphaproteobacteria	Rhizobiales	Rhizobiaceae	Rhizobium/Agrobacterium group	Agrobacterium
17	Bacteria	Proteobacteria	Alphaproteobacteria	Rhizobiales	Phyllobacteriaceae	Phyllobacterium	Phyllobacterium sp. OV277
17	Bacteria	Proteobacteria	Alphaproteobacteria	Rhizobiales	Phyllobacteriaceae	Aminobacter	Aminobacter aminovorans
17	Bacteria	Proteobacteria	Alphaproteobacteria	Rhizobiales	Methylocystaceae	Pleomorphomonas	Pleomorphomonas koreensis
17	Bacteria	Proteobacteria	Alphaproteobacteria	Rhizobiales	Methylocystaceae	Methylosinus	
17	Bacteria	Proteobacteria	Alphaproteobacteria	Rhizobiales	Methylocystaceae	Methylopila	Methylopila sp. 73B
17	Bacteria	PVC group	Planctomycetes	Planctomycetia	Planctomycetales	Isosphaeraceae	Isosphaera
17	Bacteria	PVC group	Lentisphaerae	unclassified Lentisphaerae	unclassified Lentisphaerae (miscellaneous)	Lentisphaerae bacterium GWF2_50_93	
17	Bacteria	FCB group	Bacteroidetes/Chlorobi group	Bacteroidetes	unclassified Bacteroidetes	unclassified Bacteroidetes (miscellaneous)	Bacteroidetes bacterium RIFCSPLOWO2_12_FULL_35_15
17	Bacteria	FCB group	Bacteroidetes/Chlorobi group	Bacteroidetes	unclassified Bacteroidetes	unclassified Bacteroidetes (miscellaneous)	Bacteroidetes bacterium GWF2_49_14
17	Bacteria	FCB group	Bacteroidetes/Chlorobi group	Bacteroidetes	Bacteroidia	unclassified Bacteroidia	Bacteroidia bacterium 43-41
17	dsDNA viruses, no RNA stage	Phycodnaviridae	unclassified Phycodnaviridae	Yellowstone lake phycodnavirus 1			
16	Bacteria	Terrabacteria group	Firmicutes	Clostridia	Clostridiales	Ruminococcaceae	Ruminococcus
16	Bacteria	Terrabacteria group	Firmicutes	Clostridia	Clostridiales	Ruminococcaceae	Candidatus Soleaferrea
16	Bacteria	Terrabacteria group	Firmicutes	Clostridia	Clostridiales	Peptococcaceae	
16	Bacteria	Terrabacteria group	Firmicutes	Clostridia	Clostridiales	Lachnospiraceae	Cellulosilyticum
16	Bacteria	Terrabacteria group	Chloroflexi	unclassified Chloroflexi	unclassified Chloroflexi (miscellaneous)	Chloroflexi bacterium 54-19	
16	Bacteria	Terrabacteria group	Actinobacteria	Nitriliruptoria	Nitriliruptorales	Nitriliruptoraceae	Nitriliruptor
16	Bacteria	Synergistetes	Synergistia	Synergistales	Synergistaceae	Aminobacterium	Aminobacterium colombiense
16	Bacteria	Spirochaetes	Spirochaetia	Leptospirales	Leptospiraceae	Leptospira	
16	Bacteria	Proteobacteria	delta/epsilon subdivisions	Deltaproteobacteria	Myxococcales	Nannocystineae	Nannocystaceae
16	Bacteria	Proteobacteria	delta/epsilon subdivisions	Deltaproteobacteria	Myxococcales	Cystobacterineae	Anaeromyxobacteraceae
16	Bacteria	Proteobacteria	delta/epsilon subdivisions	Deltaproteobacteria	Myxococcales		
16	Bacteria	Proteobacteria	delta/epsilon subdivisions	Deltaproteobacteria	Desulfuromonadales	Desulfuromonadaceae	Desulfuromonas
16	Bacteria	Proteobacteria	delta/epsilon subdivisions	Deltaproteobacteria	Desulfovibrionales	unclassified Desulfovibrionales	Desulfovibrionales bacterium GWA2_65_9
16	Bacteria	Proteobacteria	delta/epsilon subdivisions	Deltaproteobacteria	Desulfovibrionales	Desulfovibrionaceae	Desulfovibrio
16	Bacteria	Proteobacteria	delta/epsilon subdivisions	Deltaproteobacteria	Desulfovibrionales	Desulfonatronaceae	Desulfonatronum
16	Bacteria	Proteobacteria	delta/epsilon subdivisions	Deltaproteobacteria	Desulfobacterales	Desulfobulbaceae	Desulfofustis
16	Bacteria	Proteobacteria	delta/epsilon subdivisions	Deltaproteobacteria	Desulfobacterales	Desulfobacteraceae	Desulfatitalea
16	Bacteria	Proteobacteria	delta/epsilon subdivisions	Deltaproteobacteria	Desulfarculales	Desulfarculaceae	Desulfocarbo
16	Bacteria	Proteobacteria	Gammaproteobacteria	Xanthomonadales	Xanthomonadaceae	Arenimonas	Arenimonas sp. SCN 70-307
16	Bacteria	Proteobacteria	Gammaproteobacteria	Pseudomonadales	Moraxellaceae	Psychrobacter	
16	Bacteria	Proteobacteria	Gammaproteobacteria	Methylococcales	Methylococcaceae	Methylocaldum	Methylocaldum sp. 175
16	Bacteria	Proteobacteria	Gammaproteobacteria	Chromatiales	Chromatiaceae	Thiohalocapsa	environmental samples
16	Bacteria	Proteobacteria	Gammaproteobacteria	Aeromonadales	Aeromonadaceae	Aeromonas	Aeromonas media
16	Bacteria	Proteobacteria	Betaproteobacteria	Rhodocyclales	Rhodocyclaceae	Thauera	Thauera sp. ZV-1-C
16	Bacteria	Proteobacteria	Betaproteobacteria	Nitrosomonadales			
16	Bacteria	Proteobacteria	Betaproteobacteria	Gallionellales	Gallionellaceae	Ferriphaselus	Ferriphaselus amnicola
16	Bacteria	Proteobacteria	Betaproteobacteria	Ferrovales	Ferrovaceae	Ferrovum	Ferrovum sp. PN-J185
16	Bacteria	Proteobacteria	Betaproteobacteria	Burkholderiales	unclassified Burkholderiales	Burkholderiales Genera incertae sedis	Thiomonas
16	Bacteria	Proteobacteria	Betaproteobacteria	Burkholderiales	unclassified Burkholderiales	Burkholderiales Genera incertae sedis	Roseateles
16	Bacteria	Proteobacteria	Betaproteobacteria	Burkholderiales	Oxalobacteraceae	Janthinobacterium	Janthinobacterium agaricidamnosum
16	Bacteria	Proteobacteria	Betaproteobacteria	Burkholderiales	Comamonadaceae	unclassified Comamonadaceae	Comamonadaceae bacterium A1
16	Bacteria	Proteobacteria	Betaproteobacteria	Burkholderiales	Comamonadaceae	Pelomonas	Pelomonas sp. Root1237
16	Bacteria	Proteobacteria	Betaproteobacteria	Burkholderiales	Burkholderiaceae	Ralstonia	Ralstonia pickettii
16	Bacteria	Proteobacteria	Betaproteobacteria	Burkholderiales	Burkholderiaceae	Paraburkholderia	Paraburkholderia phenazinium
16	Bacteria	Proteobacteria	Betaproteobacteria	Burkholderiales	Burkholderiaceae	Lautropia	Lautropia sp. SCN 66-9
16	Bacteria	Proteobacteria	Betaproteobacteria	Burkholderiales	Burkholderiaceae	Candidatus Glomeribacter	Candidatus Glomeribacter gigasporarum
16	Bacteria	Proteobacteria	Betaproteobacteria	Burkholderiales	Alcaligenaceae	Candidimonas	Candidimonas bauzanensis
16	Bacteria	Proteobacteria	Alphaproteobacteria	Sphingomonadales	Sphingomonadaceae	Sphingopyxis	Sphingopyxis sp. RIFCSPHIGHO2_01_FULL_65_24
16	Bacteria	Proteobacteria	Alphaproteobacteria	Sphingomonadales	Sphingomonadaceae	Sphingomonas	Sphingomonas sp. Y57
16	Bacteria	Proteobacteria	Alphaproteobacteria	Sphingomonadales	Sphingomonadaceae	Sphingomonas	Sphingomonas sp. OK281
16	Bacteria	Proteobacteria	Alphaproteobacteria	Sphingomonadales	Sphingomonadaceae	Sphingomonas	Sphingomonas sp. LH128
16	Bacteria	Proteobacteria	Alphaproteobacteria	Sphingomonadales	Sphingomonadaceae	Sphingomonas	Sphingomonas hankookensis
16	Bacteria	Proteobacteria	Alphaproteobacteria	Sphingomonadales	Sphingomonadaceae	Sphingomonas	Sphingomonas adhaesiva
16	Bacteria	Proteobacteria	Alphaproteobacteria	Rickettsiales	Anaplasmataceae	Neorickettsia	Neorickettsia helminthoeca
16	Bacteria	Proteobacteria	Alphaproteobacteria	Rickettsiales	Anaplasmataceae	Ehrlichia	canis group
16	Bacteria	Proteobacteria	Alphaproteobacteria	Rhodospirillales	Rhodospirillaceae	Skermanella	Skermanella stibiiresistens
16	Bacteria	Proteobacteria	Alphaproteobacteria	Rhodospirillales	Acetobacteraceae	Roseomonas	Roseomonas gilardii
16	Bacteria	Proteobacteria	Alphaproteobacteria	Rhodospirillales	Acetobacteraceae	Acidiphilium	
16	Bacteria	Proteobacteria	Alphaproteobacteria	Rhodobacterales	Rhodobacteraceae	unclassified Rhodobacteraceae	Rhodobacteraceae bacterium KLH11
16	Bacteria	Proteobacteria	Alphaproteobacteria	Rhodobacterales	Rhodobacteraceae	Sulfitobacter	Sulfitobacter sp. EhC04
16	Bacteria	Proteobacteria	Alphaproteobacteria	Rhodobacterales	Rhodobacteraceae	Rubellimicrobium	Rubellimicrobium thermophilum
16	Bacteria	Proteobacteria	Alphaproteobacteria	Rhodobacterales	Rhodobacteraceae	Roseovarius	Roseovarius sp. HI0049
16	Bacteria	Proteobacteria	Alphaproteobacteria	Rhodobacterales	Rhodobacteraceae	Roseovarius	Roseovarius nanhaiticus
16	Bacteria	Proteobacteria	Alphaproteobacteria	Rhodobacterales	Rhodobacteraceae	Rhodovulum	
16	Bacteria	Proteobacteria	Alphaproteobacteria	Rhodobacterales	Rhodobacteraceae	Labrenzia	Labrenzia suaedae
16	Bacteria	Proteobacteria	Alphaproteobacteria	Rhodobacterales	Rhodobacteraceae	Celeribacter	Celeribacter neptunius
16	Bacteria	Proteobacteria	Alphaproteobacteria	Rhizobiales	unclassified Rhizobiales	unclassified Rhizobiales (miscellaneous)	Rhizobiales bacterium CCH3-A5
16	Bacteria	Proteobacteria	Alphaproteobacteria	Rhizobiales	Xanthobacteraceae	Starkeya	Starkeya novella
16	Bacteria	Proteobacteria	Alphaproteobacteria	Rhizobiales	Xanthobacteraceae	Azorhizobium	Azorhizobium caulinodans
16	Bacteria	Proteobacteria	Alphaproteobacteria	Rhizobiales	Rhodobiaceae	Roseospirillum	Roseospirillum parvum
16	Bacteria	Proteobacteria	Alphaproteobacteria	Rhizobiales	Rhodobiaceae	Afifella	Afifella pfennigii
16	Bacteria	Proteobacteria	Alphaproteobacteria	Rhizobiales	Rhizobiaceae	Rhizobium/Agrobacterium group	Rhizobium
16	Bacteria	Proteobacteria	Alphaproteobacteria	Rhizobiales	Hyphomicrobiaceae	Pelagibacterium	Pelagibacterium luteolum
16	Bacteria	Proteobacteria	Alphaproteobacteria	Rhizobiales	Aurantimonadaceae	Aurantimonas	Aurantimonas sp. 22II-16-19i
16	Bacteria	Proteobacteria	Alphaproteobacteria	Caulobacterales	Caulobacteraceae	Phenylobacterium	Phenylobacterium sp. Root700
16	Bacteria	Proteobacteria	Alphaproteobacteria	Caulobacterales	Caulobacteraceae	Asticcacaulis	
16	Bacteria	PVC group	Verrucomicrobia	Verrucomicrobiae	Verrucomicrobiales	Akkermansiaceae	Akkermansia
16	Bacteria	PVC group	Planctomycetes	Planctomycetia	Planctomycetales	unclassified Planctomycetales	unclassified Planctomycetales (miscellaneous)
16	Bacteria	PVC group	Planctomycetes	Planctomycetia	Planctomycetales	Planctomycetaceae	Rhodopirellula
16	Bacteria	PVC group	Planctomycetes	Planctomycetia	Planctomycetales	Planctomycetaceae	Rhodopirellula
16	Bacteria	PVC group	Planctomycetes	Planctomycetia	Candidatus Brocadiales	Candidatus Brocadiaceae	Candidatus Brocadia
16	Bacteria	Fusobacteria	Fusobacteriia	Fusobacteriales	Fusobacteriaceae	Fusobacterium	
16	Bacteria	FCB group	Fibrobacteres	Fibrobacteria	Fibrobacterales	Fibrobacteraceae	Fibrobacter
16	Bacteria	FCB group	Bacteroidetes/Chlorobi group	Bacteroidetes	unclassified Bacteroidetes	unclassified Bacteroidetes (miscellaneous)	Bacteroidetes bacterium RBG_13_42_15
16	Bacteria	FCB group	Bacteroidetes/Chlorobi group	Bacteroidetes	unclassified Bacteroidetes	unclassified Bacteroidetes (miscellaneous)	Bacteroidetes bacterium GWF2_41_31
16	Bacteria	FCB group	Bacteroidetes/Chlorobi group	Bacteroidetes	Saprospiria	Saprospirales	Lewinellaceae
16	Bacteria	FCB group	Bacteroidetes/Chlorobi group	Bacteroidetes	Cytophagia	Cytophagales	Cytophagaceae
16	Bacteria	FCB group	Bacteroidetes/Chlorobi group	Bacteroidetes	Cytophagia	Cytophagales	Cytophagaceae
16	Bacteria	FCB group	Bacteroidetes/Chlorobi group	Bacteroidetes	Cytophagia	Cytophagales	Cytophagaceae
16	Bacteria	FCB group	Bacteroidetes/Chlorobi group	Bacteroidetes	Bacteroidia	Marinilabiliales	Prolixibacteraceae
16	Bacteria	FCB group	Bacteroidetes/Chlorobi group	Bacteroidetes	Bacteroidia	Bacteroidales	Rikenellaceae
16	Bacteria	FCB group	Bacteroidetes/Chlorobi group	Bacteroidetes	Bacteroidia	Bacteroidales	Porphyromonadaceae
16	Bacteria	FCB group	Bacteroidetes/Chlorobi group	Bacteroidetes	Bacteroidia	Bacteroidales	Bacteroidaceae
16	Bacteria	Acidobacteria	unclassified Acidobacteria	unclassified Acidobacteria (miscellaneous)	Acidobacteria bacterium RBG_16_70_10		
16	Bacteria	Acidobacteria	Acidobacteriia	Acidobacteriales	Acidobacteriaceae	Candidatus Koribacter	Candidatus Koribacter versatilis
15	Eukaryota	Alveolata	Apicomplexa	Aconoidasida	Haemosporida	Plasmodiidae	Plasmodium
15	Bacteria	environmental samples					
15	Bacteria	Terrabacteria group	Firmicutes	Clostridia	Thermoanaerobacterales	Thermoanaerobacteraceae	Tepidanaerobacter
15	Bacteria	Terrabacteria group	Firmicutes	Clostridia	Clostridiales	Clostridiaceae	Hungatella
15	Bacteria	Terrabacteria group	Deinococcus-Thermus	Deinococci	Deinococcales	Deinococcaceae	Deinococcus
15	Bacteria	Terrabacteria group	Cyanobacteria/Melainabacteria group	Cyanobacteria	Synechococcales	Synechococcaceae	Synechococcus
15	Bacteria	Terrabacteria group	Actinobacteria	Rubrobacteria	Rubrobacterales	Rubrobacteraceae	Rubrobacter
15	Bacteria	Terrabacteria group	Actinobacteria	Actinobacteria	Micromonosporales	Micromonosporaceae	Micromonospora
15	Bacteria	Terrabacteria group	Actinobacteria	Actinobacteria	Frankiales	Frankiaceae	Frankia
15	Bacteria	Proteobacteria	delta/epsilon subdivisions	Deltaproteobacteria	Desulfuromonadales	unclassified Desulfuromonadales	
15	Bacteria	Proteobacteria	delta/epsilon subdivisions	Deltaproteobacteria	Desulfovibrionales	Desulfovibrionaceae	Desulfovibrio
15	Bacteria	Proteobacteria	delta/epsilon subdivisions	Deltaproteobacteria	Desulfovibrionales	Desulfovibrionaceae	Desulfovibrio
15	Bacteria	Proteobacteria	delta/epsilon subdivisions	Deltaproteobacteria	Desulfovibrionales	Desulfovibrionaceae	Desulfovibrio
15	Bacteria	Proteobacteria	delta/epsilon subdivisions	Deltaproteobacteria	Desulfovibrionales	Desulfohalobiaceae	Desulfovermiculus
15	Bacteria	Proteobacteria	delta/epsilon subdivisions	Deltaproteobacteria	Desulfobacterales	Desulfobulbaceae	unclassified Desulfobulbaceae
15	Bacteria	Proteobacteria	delta/epsilon subdivisions	Deltaproteobacteria	Desulfobacterales	Desulfobulbaceae	Desulfofustis
15	Bacteria	Proteobacteria	delta/epsilon subdivisions	Deltaproteobacteria	Bdellovibrionales	Bdellovibrionaceae	Bdellovibrio
15	Bacteria	Proteobacteria	Gammaproteobacteria	Xanthomonadales	Xanthomonadaceae	Pseudoxanthomonas	Pseudoxanthomonas indica
15	Bacteria	Proteobacteria	Gammaproteobacteria	Xanthomonadales	Xanthomonadaceae	Arenimonas	Arenimonas donghaensis
15	Bacteria	Proteobacteria	Gammaproteobacteria	Thiotrichales	Thiotrichaceae	Thiomargarita	Candidatus Thiomargarita nelsonii
15	Bacteria	Proteobacteria	Gammaproteobacteria	Pseudomonadales	Pseudomonadaceae	Pseudomonas	Pseudomonas putida group
15	Bacteria	Proteobacteria	Gammaproteobacteria	Oceanospirillales	Oleiphilaceae	Oleiphilus	
15	Bacteria	Proteobacteria	Gammaproteobacteria	Nevskiales	Sinobacteraceae	Polycyclovorans	Polycyclovorans algicola
15	Bacteria	Proteobacteria	Gammaproteobacteria	Methylococcales	Methylococcaceae	Methylosarcina	Methylosarcina fibrata
15	Bacteria	Proteobacteria	Gammaproteobacteria	Chromatiales	Chromatiaceae	Thiohalocapsa	Thiohalocapsa sp. ML1
15	Bacteria	Proteobacteria	Gammaproteobacteria	Acidiferrobacterales	Acidiferrobacteraceae	Sulfurifustis	Sulfurifustis variabilis
15	Bacteria	Proteobacteria	Betaproteobacteria	unclassified Betaproteobacteria	Candidatus Accumulibacter	Candidatus Accumulibacter sp. BA-91	
15	Bacteria	Proteobacteria	Betaproteobacteria	Rhodocyclales	Rhodocyclaceae	Thauera	Thauera chlorobenzoica
15	Bacteria	Proteobacteria	Betaproteobacteria	Nitrosomonadales	Nitrosomonadaceae	Nitrosomonas	Nitrosomonas halophila
15	Bacteria	Proteobacteria	Betaproteobacteria	Neisseriales	Neisseriaceae	Crenobacter	Crenobacter luteus
15	Bacteria	Proteobacteria	Betaproteobacteria	Neisseriales	Chromobacteriaceae	Formivibrio	Formivibrio citricus
15	Bacteria	Proteobacteria	Betaproteobacteria	Methylophilales	Methylophilaceae	Candidatus Methylopumilus	Candidatus Methylopumilus planktonicus
15	Bacteria	Proteobacteria	Betaproteobacteria	Hydrogenophilales	Hydrogenophilaceae	Thiobacillus	Thiobacillus sp. GWE1_62_9
15	Bacteria	Proteobacteria	Betaproteobacteria	Burkholderiales	unclassified Burkholderiales	Burkholderiales Genera incertae sedis	Tepidimonas
15	Bacteria	Proteobacteria	Betaproteobacteria	Burkholderiales	Oxalobacteraceae	Massilia	Massilia timonae
15	Bacteria	Proteobacteria	Betaproteobacteria	Burkholderiales	Oxalobacteraceae	Massilia	Massilia sp. NR 4-1
15	Bacteria	Proteobacteria	Betaproteobacteria	Burkholderiales	Comamonadaceae	unclassified Comamonadaceae	Comamonadaceae bacterium H1
15	Bacteria	Proteobacteria	Betaproteobacteria	Burkholderiales	Comamonadaceae	Variovorax	Variovorax sp. PDC80
15	Bacteria	Proteobacteria	Betaproteobacteria	Burkholderiales	Comamonadaceae	Alicycliphilus	Alicycliphilus sp. 69-12
15	Bacteria	Proteobacteria	Betaproteobacteria	Burkholderiales	Burkholderiaceae	Limnobacter	
15	Bacteria	Proteobacteria	Betaproteobacteria	Burkholderiales	Burkholderiaceae	Burkholderia	pseudomallei group
15	Bacteria	Proteobacteria	Betaproteobacteria	Burkholderiales	Burkholderiaceae	Burkholderia	Burkholderia cepacia complex
15	Bacteria	Proteobacteria	Betaproteobacteria	Burkholderiales	Alcaligenaceae	Bordetella	Bordetella pertussis
15	Bacteria	Proteobacteria	Betaproteobacteria	Burkholderiales	Alcaligenaceae	Bordetella	Bordetella flabilis
15	Bacteria	Proteobacteria	Betaproteobacteria	Burkholderiales	Alcaligenaceae	Bordetella	Bordetella ansorpii
15	Bacteria	Proteobacteria	Alphaproteobacteria	unclassified Alphaproteobacteria	unclassified Alphaproteobacteria (miscellaneous)	alpha proteobacterium Q-1	
15	Bacteria	Proteobacteria	Alphaproteobacteria	Sphingomonadales	Sphingomonadaceae	Sphingomonas	Sphingomonas sp. TDK1
15	Bacteria	Proteobacteria	Alphaproteobacteria	Sphingomonadales	Sphingomonadaceae	Sphingomonas	Sphingomonas sp. SKA58
15	Bacteria	Proteobacteria	Alphaproteobacteria	Sphingomonadales	Sphingomonadaceae	Sphingobium	Sphingobium sp. YL23
15	Bacteria	Proteobacteria	Alphaproteobacteria	Sphingomonadales	Sphingomonadaceae	Sphingobium	Sphingobium sp. AP50
15	Bacteria	Proteobacteria	Alphaproteobacteria	Rickettsiales	Anaplasmataceae	Anaplasma	
15	Bacteria	Proteobacteria	Alphaproteobacteria	Rhodospirillales	unclassified Rhodospirillales	unclassified Rhodospirillales (miscellaneous)	Rhodospirillales bacterium RIFCSPLOWO2_12_FULL_67_15
15	Bacteria	Proteobacteria	Alphaproteobacteria	Rhodospirillales	Rhodospirillaceae	Rhodospirillum	Rhodospirillum rubrum
15	Bacteria	Proteobacteria	Alphaproteobacteria	Rhodospirillales	Rhodospirillaceae	Oceanibaculum	Oceanibaculum indicum
15	Bacteria	Proteobacteria	Alphaproteobacteria	Rhodospirillales	Rhodospirillaceae	Insolitispirillum	Insolitispirillum peregrinum
15	Bacteria	Proteobacteria	Alphaproteobacteria	Rhodobacterales	Rhodobacteraceae	Thalassobius	
15	Bacteria	Proteobacteria	Alphaproteobacteria	Rhodobacterales	Rhodobacteraceae	Sulfitobacter	Sulfitobacter noctilucicola
15	Bacteria	Proteobacteria	Alphaproteobacteria	Rhodobacterales	Rhodobacteraceae	Ruegeria	Ruegeria sp. ANG-S4
15	Bacteria	Proteobacteria	Alphaproteobacteria	Rhodobacterales	Rhodobacteraceae	Ruegeria	Ruegeria atlantica
15	Bacteria	Proteobacteria	Alphaproteobacteria	Rhodobacterales	Rhodobacteraceae	Roseovarius	Roseovarius halotolerans
15	Bacteria	Proteobacteria	Alphaproteobacteria	Rhodobacterales	Rhodobacteraceae	Roseivivax	
15	Bacteria	Proteobacteria	Alphaproteobacteria	Rhodobacterales	Rhodobacteraceae	Pseudooceanicola	Pseudooceanicola batsensis
15	Bacteria	Proteobacteria	Alphaproteobacteria	Rhodobacterales	Rhodobacteraceae	Planktotalea	Planktotalea sp. IMCC9565
15	Bacteria	Proteobacteria	Alphaproteobacteria	Rhodobacterales	Rhodobacteraceae	Phaeobacter	Phaeobacter inhibens
15	Bacteria	Proteobacteria	Alphaproteobacteria	Rhodobacterales	Rhodobacteraceae	Phaeobacter	Phaeobacter gallaeciensis
15	Bacteria	Proteobacteria	Alphaproteobacteria	Rhodobacterales	Rhodobacteraceae	Paracoccus	Paracoccus denitrificans
15	Bacteria	Proteobacteria	Alphaproteobacteria	Rhodobacterales	Rhodobacteraceae	Marinosulfonomonas	Marinosulfonomonas sp. PRT-SC04
15	Bacteria	Proteobacteria	Alphaproteobacteria	Rhodobacterales	Rhodobacteraceae	Jannaschia	Jannaschia helgolandensis
15	Bacteria	Proteobacteria	Alphaproteobacteria	Rhodobacterales	Rhodobacteraceae	Jannaschia	Jannaschia donghaensis
15	Bacteria	Proteobacteria	Alphaproteobacteria	Rhodobacterales	Rhodobacteraceae	Epibacterium	Epibacterium ulvae
15	Bacteria	Proteobacteria	Alphaproteobacteria	Rhizobiales	Rhizobiaceae	Rhizobium/Agrobacterium group	Agrobacterium
15	Bacteria	Proteobacteria	Alphaproteobacteria	Rhizobiales	Phyllobacteriaceae	Hoeflea	Hoeflea olei
15	Bacteria	Proteobacteria	Alphaproteobacteria	Rhizobiales	Methylocystaceae		
15	Bacteria	Proteobacteria	Alphaproteobacteria	Rhizobiales	Methylobacteriaceae	Microvirga	Microvirga ossetica
15	Bacteria	Proteobacteria	Alphaproteobacteria	Rhizobiales	Hyphomicrobiaceae	Pelagibacterium	Pelagibacterium sp. SCN 64-44
15	Bacteria	Proteobacteria	Alphaproteobacteria	Rhizobiales	Hyphomicrobiaceae	Devosia	Devosia sp. Root685
15	Bacteria	Proteobacteria	Alphaproteobacteria	Rhizobiales	Bradyrhizobiaceae	Bradyrhizobium	Bradyrhizobium sp. Ec3.3
15	Bacteria	Proteobacteria	Alphaproteobacteria	Caulobacterales	Caulobacteraceae	Phenylobacterium	Phenylobacterium zucineum
15	Bacteria	PVC group	Verrucomicrobia	Verrucomicrobiae	Verrucomicrobiales	Rubritaleaceae	Rubritalea
15	Bacteria	PVC group	Planctomycetes	unclassified Planctomycetes	Planctomycetes bacterium GWF2_42_9		
15	Bacteria	PVC group	Planctomycetes	Planctomycetia	Planctomycetales	Planctomycetaceae	Planctomyces
15	Bacteria	PVC group	Planctomycetes	Planctomycetia	Planctomycetales	Planctomycetaceae	
15	Bacteria	PVC group	Lentisphaerae	unclassified Lentisphaerae	unclassified Lentisphaerae (miscellaneous)	Lentisphaerae bacterium RIFOXYA12_FULL_48_11	
15	Bacteria	PVC group	Chlamydiae	Chlamydiia	Chlamydiales	Chlamydiaceae	Chlamydia/Chlamydophila group
15	Bacteria	Nitrospirae	Nitrospira	Nitrospirales	Nitrospiraceae	Leptospirillum	
15	Bacteria	FCB group	Bacteroidetes/Chlorobi group	Bacteroidetes	unclassified Bacteroidetes	unclassified Bacteroidetes (miscellaneous)	Bacteroidetes bacterium GWA2_31_9
15	Bacteria	FCB group	Bacteroidetes/Chlorobi group	Bacteroidetes	unclassified Bacteroidetes	unclassified Bacteroidetes (miscellaneous)	Bacteroidetes bacterium 38_7
15	Bacteria	FCB group	Bacteroidetes/Chlorobi group	Bacteroidetes	Sphingobacteriia	Sphingobacteriales	unclassified Sphingobacteriales
15	Bacteria	FCB group	Bacteroidetes/Chlorobi group	Bacteroidetes	Saprospiria	Saprospirales	Lewinellaceae
15	Bacteria	FCB group	Bacteroidetes/Chlorobi group	Bacteroidetes	Saprospiria	Saprospirales	Haliscomenobacteraceae
15	Bacteria	FCB group	Bacteroidetes/Chlorobi group	Bacteroidetes	Flavobacteriia	Flavobacteriales	Flavobacteriaceae
15	Bacteria	FCB group	Bacteroidetes/Chlorobi group	Bacteroidetes	Cytophagia	Cytophagales	Cytophagaceae
15	Bacteria	FCB group	Bacteroidetes/Chlorobi group	Bacteroidetes	Bacteroidia	Marinilabiliales	Marinilabiliaceae
15	Bacteria	FCB group	Bacteroidetes/Chlorobi group	Bacteroidetes	Bacteroidia	Marinilabiliales	Marinilabiliaceae
15	Bacteria	FCB group	Bacteroidetes/Chlorobi group	Bacteroidetes	Bacteroidia	Marinilabiliales	Marinilabiliaceae
15	Bacteria	FCB group	Bacteroidetes/Chlorobi group	Bacteroidetes	Bacteroidia	Bacteroidales	Porphyromonadaceae
15	Bacteria	FCB group	Bacteroidetes/Chlorobi group	Bacteroidetes	Bacteroidia	Bacteroidales	Porphyromonadaceae
15	Bacteria	FCB group	Bacteroidetes/Chlorobi group	Bacteroidetes	Bacteroidia	Bacteroidales	Bacteroidaceae
15	Bacteria	FCB group	Bacteroidetes/Chlorobi group	Bacteroidetes	Bacteroidia	Bacteroidales	Bacteroidaceae
15	Bacteria	Acidobacteria	unclassified Acidobacteria	unclassified Acidobacteria (miscellaneous)	Acidobacteria bacterium 13_1_40CM_3_55_6		
14	Eukaryota	Rhodophyta	Florideophyceae	Gigartinales	Gigartinaceae	Chondrus	Chondrus crispus
14	Bacteria	unclassified Bacteria	unclassified Bacteria (miscellaneous)	bacterium MS4			
14	Bacteria	unclassified Bacteria	Bacteria candidate phyla	Candidatus Saccharibacteria	unclassified Saccharibacteria		
14	Bacteria	environmental samples	uncultured bacterium A1Q1_fos_1266				
14	Bacteria	Terrabacteria group	Firmicutes	Clostridia	unclassified Clostridia	unclassified Clostridia (miscellaneous)	Clostridia bacterium BRH_c25
14	Bacteria	Terrabacteria group	Firmicutes	Clostridia	Clostridiales	unclassified Clostridiales	unclassified Clostridiales (miscellaneous)
14	Bacteria	Terrabacteria group	Firmicutes	Clostridia	Clostridiales	Ruminococcaceae	Fournierella
14	Bacteria	Terrabacteria group	Firmicutes	Clostridia	Clostridiales	Ruminococcaceae	Angelakisella
14	Bacteria	Terrabacteria group	Firmicutes	Clostridia	Clostridiales	Lachnospiraceae	unclassified Lachnospiraceae
14	Bacteria	Terrabacteria group	Firmicutes	Clostridia	Clostridiales	Defluviitaleaceae	Defluviitalea
14	Bacteria	Terrabacteria group	Firmicutes	Clostridia	Clostridiales	Clostridiaceae	Maledivibacter
14	Bacteria	Terrabacteria group	Firmicutes	Bacilli	Lactobacillales	Enterococcaceae	Enterococcus
14	Bacteria	Terrabacteria group	Chloroflexi	Chloroflexia	Chloroflexales	Chloroflexineae	Oscillochloridaceae
14	Bacteria	Terrabacteria group	Armatimonadetes	Fimbriimonadia	Fimbriimonadales	Fimbriimonadaceae	Fimbriimonas
14	Bacteria	Terrabacteria group	Actinobacteria	Actinobacteria	unclassified Actinobacteria (class)	unclassified Actinobacteria (class) (miscellaneous)	marine actinobacterium MedAcidi-G2A
14	Bacteria	Terrabacteria group	Actinobacteria	Actinobacteria	Corynebacteriales	Nocardiaceae	Nocardia
14	Bacteria	Synergistetes	Synergistia	Synergistales	Synergistaceae	Thermanaerovibrio	Thermanaerovibrio acidaminovorans
14	Bacteria	Synergistetes	Synergistia	Synergistales	Synergistaceae	Synergistes	Synergistes jonesii
14	Bacteria	Synergistetes	Synergistia	Synergistales	Synergistaceae	Dethiosulfovibrio	Dethiosulfovibrio peptidovorans
14	Bacteria	Proteobacteria	unclassified Proteobacteria	unclassified Proteobacteria (miscellaneous)	Proteobacteria bacterium SG_bin9		
14	Bacteria	Proteobacteria	delta/epsilon subdivisions	Deltaproteobacteria	unclassified Deltaproteobacteria	unclassified Deltaproteobacteria (miscellaneous)	Deltaproteobacteria bacterium CG2_30_63_29
14	Bacteria	Proteobacteria	delta/epsilon subdivisions	Deltaproteobacteria	Syntrophobacterales	Syntrophaceae	Syntrophus
14	Bacteria	Proteobacteria	delta/epsilon subdivisions	Deltaproteobacteria	Desulfovibrionales	Desulfovibrionaceae	Desulfovibrio
14	Bacteria	Proteobacteria	delta/epsilon subdivisions	Deltaproteobacteria	Desulfovibrionales	Desulfomicrobiaceae	Desulfomicrobium
14	Bacteria	Proteobacteria	Gammaproteobacteria	unclassified Gammaproteobacteria	OMG group	OM182 clade	
14	Bacteria	Proteobacteria	Gammaproteobacteria	Xanthomonadales	unclassified Xanthomonadales		
14	Bacteria	Proteobacteria	Gammaproteobacteria	Xanthomonadales	Xanthomonadaceae	Xylella	Xylella fastidiosa
14	Bacteria	Proteobacteria	Gammaproteobacteria	Xanthomonadales	Xanthomonadaceae	Lysobacter	Lysobacter sp. URHA0019
14	Bacteria	Proteobacteria	Gammaproteobacteria	Xanthomonadales	Xanthomonadaceae	Lysobacter	Lysobacter concretionis
14	Bacteria	Proteobacteria	Gammaproteobacteria	Xanthomonadales	Rhodanobacteraceae	Dyella	Dyella jiangningensis
14	Bacteria	Proteobacteria	Gammaproteobacteria	Pseudomonadales	Pseudomonadaceae	Pseudomonas	Pseudomonas hussainii
14	Bacteria	Proteobacteria	Gammaproteobacteria	Pseudomonadales	Moraxellaceae	Perlucidibaca	Perlucidibaca sp. BK296
14	Bacteria	Proteobacteria	Gammaproteobacteria	Nevskiales	Sinobacteraceae	Solimonas	Solimonas soli
14	Bacteria	Proteobacteria	Gammaproteobacteria	Methylococcales	Methylococcaceae	unclassified Methylococcaceae	Methylococcaceae bacterium Sn10-6
14	Bacteria	Proteobacteria	Gammaproteobacteria	Methylococcales	Methylococcaceae	Methylomicrobium	
14	Bacteria	Proteobacteria	Gammaproteobacteria	Chromatiales	Ectothiorhodospiraceae	Thioalkalivibrio	Thioalkalivibrio sulfidiphilus
14	Bacteria	Proteobacteria	Gammaproteobacteria	Chromatiales	Chromatiaceae	Thiocystis	Thiocystis violascens
14	Bacteria	Proteobacteria	Betaproteobacteria	unclassified Betaproteobacteria	unclassified Betaproteobacteria (miscellaneous)	beta proteobacterium L13	
14	Bacteria	Proteobacteria	Betaproteobacteria	unclassified Betaproteobacteria	Candidatus Accumulibacter	Candidatus Accumulibacter sp. SK-11	
14	Bacteria	Proteobacteria	Betaproteobacteria	unclassified Betaproteobacteria	Candidatus Accumulibacter	Candidatus Accumulibacter sp. BA-92	
14	Bacteria	Proteobacteria	Betaproteobacteria	Rhodocyclales	Rhodocyclaceae	Thauera	Thauera linaloolentis
14	Bacteria	Proteobacteria	Betaproteobacteria	Rhodocyclales	Rhodocyclaceae	Methyloversatilis	Methyloversatilis discipulorum
14	Bacteria	Proteobacteria	Betaproteobacteria	Neisseriales	Neisseriaceae		
14	Bacteria	Proteobacteria	Betaproteobacteria	Neisseriales	Chromobacteriaceae	Chitinibacter	
14	Bacteria	Proteobacteria	Betaproteobacteria	Methylophilales	Methylophilaceae	Methylophilus	Methylophilus methylotrophus
14	Bacteria	Proteobacteria	Betaproteobacteria	Hydrogenophilales	Hydrogenophilaceae	Thiobacillus	Thiobacillus sp. SCN 63-374
14	Bacteria	Proteobacteria	Betaproteobacteria	Gallionellales	Gallionellaceae	unclassified Gallionellaceae	Gallionellaceae bacterium CG1_02_56_997
14	Bacteria	Proteobacteria	Betaproteobacteria	Burkholderiales	unclassified Burkholderiales	unclassified Burkholderiales (miscellaneous)	Burkholderiales bacterium RIFOXYC12_FULL_60_6
14	Bacteria	Proteobacteria	Betaproteobacteria	Burkholderiales	Sutterellaceae		
14	Bacteria	Proteobacteria	Betaproteobacteria	Burkholderiales	Oxalobacteraceae	Massilia	Massilia yuzhufengensis
14	Bacteria	Proteobacteria	Betaproteobacteria	Burkholderiales	Oxalobacteraceae	Massilia	Massilia niastensis
14	Bacteria	Proteobacteria	Betaproteobacteria	Burkholderiales	Oxalobacteraceae	Duganella	Duganella sp. CF402
14	Bacteria	Proteobacteria	Betaproteobacteria	Burkholderiales	Comamonadaceae	Acidovorax	Acidovorax sp. CCH12-A4
14	Bacteria	Proteobacteria	Betaproteobacteria	Burkholderiales	Burkholderiaceae	Paraburkholderia	Paraburkholderia oxyphila
14	Bacteria	Proteobacteria	Betaproteobacteria	Burkholderiales	Burkholderiaceae	Burkholderia	Burkholderia sp. JS23
14	Bacteria	Proteobacteria	Betaproteobacteria	Burkholderiales	Alcaligenaceae	Orrella	Orrella dioscoreae
14	Bacteria	Proteobacteria	Betaproteobacteria	Burkholderiales	Alcaligenaceae	Basilea	Basilea psittacipulmonis
14	Bacteria	Proteobacteria	Betaproteobacteria	Burkholderiales	Alcaligenaceae	Achromobacter	Achromobacter sp. DMS1
14	Bacteria	Proteobacteria	Betaproteobacteria	Burkholderiales	Alcaligenaceae	Achromobacter	Achromobacter piechaudii
14	Bacteria	Proteobacteria	Alphaproteobacteria	unclassified Alphaproteobacteria	unclassified Alphaproteobacteria (miscellaneous)	Alphaproteobacteria bacterium MedPE-SWcel	
14	Bacteria	Proteobacteria	Alphaproteobacteria	unclassified Alphaproteobacteria	unclassified Alphaproteobacteria (miscellaneous)		
14	Bacteria	Proteobacteria	Alphaproteobacteria	Sphingomonadales	Sphingomonadaceae	Sphingomonas	Sphingomonas sp. MM-1
14	Bacteria	Proteobacteria	Alphaproteobacteria	Sphingomonadales	Sphingomonadaceae	Sphingobium	Sphingobium sp. SCN 64-10
14	Bacteria	Proteobacteria	Alphaproteobacteria	Sphingomonadales	Sphingomonadaceae	Sphingobium	Sphingobium lactosutens
14	Bacteria	Proteobacteria	Alphaproteobacteria	Rickettsiales	Anaplasmataceae	Ehrlichia	canis group
14	Bacteria	Proteobacteria	Alphaproteobacteria	Rhodospirillales	Rhodospirillaceae	Skermanella	Skermanella aerolata
14	Bacteria	Proteobacteria	Alphaproteobacteria	Rhodospirillales	Rhodospirillaceae	Caenispirillum	Caenispirillum salinarum
14	Bacteria	Proteobacteria	Alphaproteobacteria	Rhodobacterales	Rhodobacteraceae	Shimia	Shimia haliotis
14	Bacteria	Proteobacteria	Alphaproteobacteria	Rhodobacterales	Rhodobacteraceae	Roseovarius	Roseovarius mucosus
14	Bacteria	Proteobacteria	Alphaproteobacteria	Rhodobacterales	Rhodobacteraceae	Rhodobacter	Rhodobacter sp. LPB0142
14	Bacteria	Proteobacteria	Alphaproteobacteria	Rhodobacterales	Rhodobacteraceae	Maribius	Maribius sp. MOLA 401
14	Bacteria	Proteobacteria	Alphaproteobacteria	Rhodobacterales	Rhodobacteraceae	Litoreibacter	Litoreibacter albidus
14	Bacteria	Proteobacteria	Alphaproteobacteria	Rhodobacterales	Hyphomonadaceae	unclassified Hyphomonadaceae	Hyphomonadaceae bacterium BRH_c29
14	Bacteria	Proteobacteria	Alphaproteobacteria	Rhodobacterales	Hyphomonadaceae	Hyphomonas	Hyphomonas sp. Mor2
14	Bacteria	Proteobacteria	Alphaproteobacteria	Rhodobacterales	Hyphomonadaceae	Henriciella	Henriciella litoralis
14	Bacteria	Proteobacteria	Alphaproteobacteria	Rhizobiales	unclassified Rhizobiales	Methyloceanibacter	
14	Bacteria	Proteobacteria	Alphaproteobacteria	Rhizobiales	Rhizobiaceae	Rhizobium/Agrobacterium group	Rhizobium
14	Bacteria	Proteobacteria	Alphaproteobacteria	Rhizobiales	Phyllobacteriaceae	Phyllobacterium	Phyllobacterium sp. YR531
14	Bacteria	Proteobacteria	Alphaproteobacteria	Rhizobiales	Phyllobacteriaceae	Mesorhizobium	Mesorhizobium ciceri
14	Bacteria	Proteobacteria	Alphaproteobacteria	Rhizobiales	Phyllobacteriaceae	Hoeflea	Hoeflea sp. 108
14	Bacteria	Proteobacteria	Alphaproteobacteria	Rhizobiales	Hyphomicrobiaceae	Devosia	Devosia sp. 67-54
14	Bacteria	Proteobacteria	Alphaproteobacteria	Rhizobiales	Hyphomicrobiaceae	Devosia	Devosia limi
14	Bacteria	Proteobacteria	Alphaproteobacteria	Rhizobiales	Brucellaceae	Pseudochrobactrum	
14	Bacteria	Proteobacteria	Alphaproteobacteria	Rhizobiales	Bradyrhizobiaceae	Bradyrhizobium	Bradyrhizobium sp. Tv2a-2
14	Bacteria	Proteobacteria	Alphaproteobacteria	Rhizobiales	Bradyrhizobiaceae	Bradyrhizobium	Bradyrhizobium sp. BTAi1
14	Bacteria	Proteobacteria	Alphaproteobacteria	Rhizobiales	Bradyrhizobiaceae	Bosea	Bosea sp. WAO
14	Bacteria	Proteobacteria	Alphaproteobacteria	Rhizobiales	Bradyrhizobiaceae	Bosea	Bosea sp. Root381
14	Bacteria	Proteobacteria	Alphaproteobacteria	Rhizobiales	Aurantimonadaceae	Aureimonas	Aureimonas sp. AU22
14	Bacteria	Proteobacteria	Alphaproteobacteria	Kordiimonadales	Kordiimonadaceae	Kordiimonas	Kordiimonas gwangyangensis
14	Bacteria	Proteobacteria	Alphaproteobacteria	Caulobacterales	unclassified Caulobacterales	Caulobacterales bacterium 68-7	
14	Bacteria	Proteobacteria	Alphaproteobacteria	Caulobacterales	Caulobacteraceae	unclassified Caulobacteraceae	Caulobacteraceae bacterium PMMR1
14	Bacteria	PVC group	Planctomycetes	unclassified Planctomycetes	Planctomycetes bacterium SM23_25		
14	Bacteria	PVC group	Planctomycetes	unclassified Planctomycetes	Planctomycetes bacterium GWF2_41_51		
14	Bacteria	PVC group	Planctomycetes	Planctomycetia	Planctomycetales	Gemmataceae	Gemmata
14	Bacteria	PVC group	Planctomycetes	Phycisphaerae	Phycisphaerales	Phycisphaeraceae	Phycisphaera
14	Bacteria	Nitrospirae	Nitrospira	Nitrospirales	Nitrospiraceae	Nitrospira	Nitrospira sp. OLB3
14	Bacteria	Nitrospirae	Nitrospira	Nitrospirales	Nitrospiraceae	Leptospirillum	Leptospirillum sp. Group I
14	Bacteria	FCB group	Gemmatimonadetes	Gemmatimonadetes	Gemmatimonadales	Gemmatimonadaceae	Gemmatimonas
14	Bacteria	FCB group	Bacteroidetes/Chlorobi group	Bacteroidetes	unclassified Bacteroidetes	unclassified Bacteroidetes (miscellaneous)	Bacteroidetes oral taxon 274
14	Bacteria	FCB group	Bacteroidetes/Chlorobi group	Bacteroidetes	Sphingobacteriia	Sphingobacteriales	unclassified Sphingobacteriales
14	Bacteria	FCB group	Bacteroidetes/Chlorobi group	Bacteroidetes	Cytophagia	Cytophagales	unclassified Cytophagales
14	Bacteria	FCB group	Bacteroidetes/Chlorobi group	Bacteroidetes	Cytophagia	Cytophagales	unclassified Cytophagales
14	Bacteria	FCB group	Bacteroidetes/Chlorobi group	Bacteroidetes	Cytophagia	Cytophagales	Cytophagaceae
14	Bacteria	FCB group	Bacteroidetes/Chlorobi group	Bacteroidetes	Cytophagia	Cytophagales	Cytophagaceae
14	Bacteria	FCB group	Bacteroidetes/Chlorobi group	Bacteroidetes	Cytophagia	Cytophagales	Cytophagaceae
14	Bacteria	FCB group	Bacteroidetes/Chlorobi group	Bacteroidetes	Cytophagia	Cytophagales	Cytophagaceae
14	Bacteria	FCB group	Bacteroidetes/Chlorobi group	Bacteroidetes	Chitinophagia	Chitinophagales	Chitinophagaceae
14	Bacteria	FCB group	Bacteroidetes/Chlorobi group	Bacteroidetes	Bacteroidia	Marinilabiliales	Marinilabiliaceae
14	Bacteria	FCB group	Bacteroidetes/Chlorobi group	Bacteroidetes	Bacteroidia	Bacteroidales	Bacteroidaceae
14	Bacteria	FCB group	Bacteroidetes/Chlorobi group	Bacteroidetes	Bacteroidia	Bacteroidales	Bacteroidaceae
14	Bacteria	Elusimicrobia	unclassified Elusimicrobia	Elusimicrobia bacterium CG1_02_63_36			
13	Eukaryota	Cryptophyta	Pyrenomonadales	Geminigeraceae	Guillardia	Guillardia theta	Guillardia theta CCMP2712
13	Bacteria	unclassified Bacteria	unclassified Bacteria (miscellaneous)	magneto-ovoid bacterium MO-1			
13	Bacteria	unclassified Bacteria	Bacteria candidate phyla	Candidatus Eisenbacteria	Candidatus Eisenbacteria bacterium RBG_16_71_46		
13	Bacteria	unclassified Bacteria	Bacteria candidate phyla	Candidatus Dadabacteria	Candidatus Dadabacteria bacterium RIFCSPHIGHO2_12_FULL_53_21		
13	Bacteria	unclassified Bacteria	Bacteria candidate phyla	Candidatus Aminicenantes	unclassified Aminicenantes	Candidatus Aminicenantes bacterium RBG_16_63_16	
13	Bacteria	Terrabacteria group	Firmicutes	Clostridia	Clostridiales	Ruminococcaceae	Faecalibacterium
13	Bacteria	Terrabacteria group	Firmicutes	Clostridia	Clostridiales	Lachnospiraceae	Robinsoniella

13	Bacteria	Terrabacteria group	Firmicutes	Clostridia	Clostridiales	Lachnospiraceae	Anaerocolumna
13	Bacteria	Terrabacteria group	Firmicutes	Clostridia	Clostridiales	Clostridiaceae	Clostridium
13	Bacteria	Terrabacteria group	Cyanobacteria/Melainabacteria group	Cyanobacteria	Nostocales	Hapalosiphonaceae	Mastigocladus
13	Bacteria	Terrabacteria group	Cyanobacteria/Melainabacteria group	Cyanobacteria	Nostocales		
13	Bacteria	Terrabacteria group	Chloroflexi	unclassified Chloroflexi	unclassified Chloroflexi (miscellaneous)		
13	Bacteria	Terrabacteria group	Armatimonadetes	unclassified Armatimonadetes	Armatimonadetes bacterium OLB18		
13	Bacteria	Terrabacteria group	Actinobacteria	Thermoleophilia	Solirubrobacterales	Solirubrobacteraceae	Solirubrobacter
13	Bacteria	Terrabacteria group	Actinobacteria	Actinobacteria	unclassified Actinobacteria (class)	unclassified Actinobacteria (class) (miscellaneous)	marine actinobacterium MedAcidi-G2B
13	Bacteria	Terrabacteria group	Actinobacteria	Actinobacteria	unclassified Actinobacteria (class)	unclassified Actinobacteria (class) (miscellaneous)	marine actinobacterium MedAcidi-G1
13	Bacteria	Terrabacteria group	Actinobacteria	Actinobacteria	Pseudonocardiales	Pseudonocardiaceae	Pseudonocardia
13	Bacteria	Terrabacteria group	Actinobacteria	Actinobacteria	Pseudonocardiales	Pseudonocardiaceae	
13	Bacteria	Synergistetes	Synergistia	Synergistales	Synergistaceae	Acetomicrobium	
13	Bacteria	Proteobacteria	delta/epsilon subdivisions	Deltaproteobacteria	Myxococcales	Cystobacterineae	Archangiaceae
13	Bacteria	Proteobacteria	delta/epsilon subdivisions	Deltaproteobacteria	Myxococcales	Cystobacterineae	Anaeromyxobacteraceae
13	Bacteria	Proteobacteria	delta/epsilon subdivisions	Deltaproteobacteria	Myxococcales	Cystobacterineae	Anaeromyxobacteraceae
13	Bacteria	Proteobacteria	delta/epsilon subdivisions	Deltaproteobacteria	Desulfuromonadales	Desulfuromonadaceae	Pelobacter
13	Bacteria	Proteobacteria	delta/epsilon subdivisions	Deltaproteobacteria	Desulfovibrionales	Desulfovibrionaceae	Desulfovibrio
13	Bacteria	Proteobacteria	delta/epsilon subdivisions	Deltaproteobacteria	Desulfovibrionales	Desulfovibrionaceae	Desulfovibrio
13	Bacteria	Proteobacteria	delta/epsilon subdivisions	Deltaproteobacteria	Desulfovibrionales	Desulfonatronaceae	Desulfonatronum
13	Bacteria	Proteobacteria	delta/epsilon subdivisions	Deltaproteobacteria	Desulfovibrionales	Desulfomicrobiaceae	Desulfomicrobium
13	Bacteria	Proteobacteria	delta/epsilon subdivisions	Deltaproteobacteria	Desulfovibrionales	Desulfomicrobiaceae	Desulfomicrobium
13	Bacteria	Proteobacteria	delta/epsilon subdivisions	Deltaproteobacteria	Desulfobacterales	Desulfobulbaceae	unclassified Desulfobulbaceae
13	Bacteria	Proteobacteria	delta/epsilon subdivisions	Deltaproteobacteria	Desulfobacterales	Desulfobulbaceae	Desulfobulbus
13	Bacteria	Proteobacteria	delta/epsilon subdivisions	Deltaproteobacteria	Desulfobacterales	Desulfobacteraceae	unclassified Desulfobacteraceae
13	Bacteria	Proteobacteria	delta/epsilon subdivisions	Deltaproteobacteria	Desulfobacterales	Desulfobacteraceae	Desulfococcus
13	Bacteria	Proteobacteria	delta/epsilon subdivisions	Deltaproteobacteria	Desulfobacterales	Desulfobacteraceae	
13	Bacteria	Proteobacteria	delta/epsilon subdivisions	Deltaproteobacteria	Desulfarculales	Desulfarculaceae	Desulfarculus
13	Bacteria	Proteobacteria	Gammaproteobacteria	unclassified Gammaproteobacteria	Pseudohongiella	Pseudohongiella acticola	
13	Bacteria	Proteobacteria	Gammaproteobacteria	Xanthomonadales	Xanthomonadaceae	Pseudoxanthomonas	Pseudoxanthomonas sp. Root65
13	Bacteria	Proteobacteria	Gammaproteobacteria	Xanthomonadales	Xanthomonadaceae	Luteimonas	Luteimonas abyssi
13	Bacteria	Proteobacteria	Gammaproteobacteria	Vibrionales	Vibrionaceae	Vibrio	Vibrio harveyi group
13	Bacteria	Proteobacteria	Gammaproteobacteria	Pseudomonadales	Pseudomonadaceae	Rugamonas	Rugamonas rubra
13	Bacteria	Proteobacteria	Gammaproteobacteria	Pseudomonadales	Pseudomonadaceae	Pseudomonas	Pseudomonas syringae group
13	Bacteria	Proteobacteria	Gammaproteobacteria	Oceanospirillales	Halomonadaceae	Halotalea	Halotalea alkalilenta
13	Bacteria	Proteobacteria	Gammaproteobacteria	Oceanospirillales	Hahellaceae	Zooshikella	Zooshikella ganghwensis
13	Bacteria	Proteobacteria	Gammaproteobacteria	Oceanospirillales	Hahellaceae	Hahella	Hahella chejuensis
13	Bacteria	Proteobacteria	Gammaproteobacteria	Methylococcales	Methylothermaceae	Methylohalobius	Methylohalobius crimeensis
13	Bacteria	Proteobacteria	Gammaproteobacteria	Methylococcales	Methylococcaceae	Methylomonas	Methylomonas methanica
13	Bacteria	Proteobacteria	Gammaproteobacteria	Methylococcales	Methylococcaceae	Methylococcus	Methylococcus capsulatus
13	Bacteria	Proteobacteria	Gammaproteobacteria	Chromatiales	Ectothiorhodospiraceae	Alkalilimnicola	Alkalilimnicola ehrlichii
13	Bacteria	Proteobacteria	Gammaproteobacteria	Chromatiales	Chromatiaceae	Thiocapsa	Thiocapsa sp. KS1
13	Bacteria	Proteobacteria	Gammaproteobacteria	Chromatiales	Chromatiaceae	Candidatus Tenderia	Candidatus Tenderia electrophaga
13	Bacteria	Proteobacteria	Gammaproteobacteria	Cellvibrionales	Spongiibacteraceae	unclassified Spongiibacteraceae	BD1-7 clade
13	Bacteria	Proteobacteria	Gammaproteobacteria	Cellvibrionales	Microbulbiferaceae	Microbulbifer	
13	Bacteria	Proteobacteria	Gammaproteobacteria	Cellvibrionales	Halieaceae	Halioglobus	
13	Bacteria	Proteobacteria	Betaproteobacteria	unclassified Betaproteobacteria	unclassified Betaproteobacteria (miscellaneous)	Betaproteobacteria bacterium RBG_16_58_11	
13	Bacteria	Proteobacteria	Betaproteobacteria	unclassified Betaproteobacteria	Kinetoplastibacterium		
13	Bacteria	Proteobacteria	Betaproteobacteria	Sulfuricellales	Sulfuricellaceae	Sulfuricella	Sulfuricella denitrificans
13	Bacteria	Proteobacteria	Betaproteobacteria	Rhodocyclales	Rhodocyclaceae	Methyloversatilis	Methyloversatilis universalis
13	Bacteria	Proteobacteria	Betaproteobacteria	Nitrosomonadales	Nitrosomonadaceae	Nitrosospira	Nitrosospira multiformis
13	Bacteria	Proteobacteria	Betaproteobacteria	Nitrosomonadales	Nitrosomonadaceae	Nitrosomonas	Nitrosomonas ureae
13	Bacteria	Proteobacteria	Betaproteobacteria	Nitrosomonadales	Nitrosomonadaceae	Nitrosomonas	Nitrosomonas nitrosa
13	Bacteria	Proteobacteria	Betaproteobacteria	Neisseriales	Chromobacteriaceae	Vogesella	Vogesella sp. LIG4
13	Bacteria	Proteobacteria	Betaproteobacteria	Neisseriales	Chromobacteriaceae	Chromobacterium group	Chromobacterium
13	Bacteria	Proteobacteria	Betaproteobacteria	Gallionellales	unclassified Gallionellales	Gallionellales bacterium GWA2_60_18	
13	Bacteria	Proteobacteria	Betaproteobacteria	Gallionellales	unclassified Gallionellales	Gallionellales bacterium GWA2_60_142	
13	Bacteria	Proteobacteria	Betaproteobacteria	Burkholderiales	Oxalobacteraceae	Janthinobacterium	Janthinobacterium sp. Marseille
13	Bacteria	Proteobacteria	Betaproteobacteria	Burkholderiales	Oxalobacteraceae	Herbaspirillum	Herbaspirillum frisingense
13	Bacteria	Proteobacteria	Betaproteobacteria	Burkholderiales	Comamonadaceae	Variovorax	Variovorax sp. CF313
13	Bacteria	Proteobacteria	Betaproteobacteria	Burkholderiales	Comamonadaceae	Caldimonas	Caldimonas manganoxidans
13	Bacteria	Proteobacteria	Betaproteobacteria	Burkholderiales	Comamonadaceae	Acidovorax	Acidovorax sp. JHL-9
13	Bacteria	Proteobacteria	Betaproteobacteria	Burkholderiales	Burkholderiaceae	Burkholderia	Burkholderia cepacia complex
13	Bacteria	Proteobacteria	Betaproteobacteria	Burkholderiales	Alcaligenaceae	Pusillimonas	Pusillimonas noertemannii
13	Bacteria	Proteobacteria	Betaproteobacteria	Burkholderiales	Alcaligenaceae	Oligella	
13	Bacteria	Proteobacteria	Betaproteobacteria	Burkholderiales	Alcaligenaceae	Derxia	
13	Bacteria	Proteobacteria	Alphaproteobacteria	Sphingomonadales	Sphingomonadaceae	Sphingopyxis	Sphingopyxis sp. Root1497
13	Bacteria	Proteobacteria	Alphaproteobacteria	Sphingomonadales	Sphingomonadaceae	Sphingomonas	Sphingomonas sp. NFR15
13	Bacteria	Proteobacteria	Alphaproteobacteria	Sphingomonadales	Sphingomonadaceae	Sphingomonas	Sphingomonas sp. Ant H11
13	Bacteria	Proteobacteria	Alphaproteobacteria	Sphingomonadales	Sphingomonadaceae	Sphingomonas	Sphingomonas azotifigens
13	Bacteria	Proteobacteria	Alphaproteobacteria	Sphingomonadales	Sphingomonadaceae	Sphingobium	Sphingobium czechense
13	Bacteria	Proteobacteria	Alphaproteobacteria	Sphingomonadales	Sphingomonadaceae	Sandarakinorhabdus	Sandarakinorhabdus limnophila
13	Bacteria	Proteobacteria	Alphaproteobacteria	Rhodospirillales	unclassified Rhodospirillales	unclassified Rhodospirillales (miscellaneous)	
13	Bacteria	Proteobacteria	Alphaproteobacteria	Rhodospirillales	Rhodospirillaceae	Magnetospira	Magnetospira sp. QH-2
13	Bacteria	Proteobacteria	Alphaproteobacteria	Rhodospirillales	Acetobacteraceae	Belnapia	
13	Bacteria	Proteobacteria	Alphaproteobacteria	Rhodobacterales	unclassified Rhodobacterales	Rhodobacterales bacterium HTCC2255	
13	Bacteria	Proteobacteria	Alphaproteobacteria	Rhodobacterales	Rhodobacteraceae	Sulfitobacter	Sulfitobacter guttiformis
13	Bacteria	Proteobacteria	Alphaproteobacteria	Rhodobacterales	Rhodobacteraceae	Ruegeria	Ruegeria sp. 6PALISEP08
13	Bacteria	Proteobacteria	Alphaproteobacteria	Rhodobacterales	Rhodobacteraceae	Roseovarius	Roseovarius lutimaris
13	Bacteria	Proteobacteria	Alphaproteobacteria	Rhodobacterales	Rhodobacteraceae	Roseobacter	Roseobacter litoralis
13	Bacteria	Proteobacteria	Alphaproteobacteria	Rhodobacterales	Rhodobacteraceae	Pseudovibrio	Pseudovibrio stylochi
13	Bacteria	Proteobacteria	Alphaproteobacteria	Rhodobacterales	Rhodobacteraceae	Marivita	
13	Bacteria	Proteobacteria	Alphaproteobacteria	Rhodobacterales	Rhodobacteraceae	Maribius	
13	Bacteria	Proteobacteria	Alphaproteobacteria	Rhodobacterales	Rhodobacteraceae	Jannaschia	Jannaschia seosinensis
13	Bacteria	Proteobacteria	Alphaproteobacteria	Rhodobacterales	Rhodobacteraceae	Haematobacter	Haematobacter missouriensis
13	Bacteria	Proteobacteria	Alphaproteobacteria	Rhodobacterales	Rhodobacteraceae	Celeribacter	Celeribacter halophilus
13	Bacteria	Proteobacteria	Alphaproteobacteria	Rhodobacterales	Hyphomonadaceae	unclassified Hyphomonadaceae	Hyphomonadaceae bacterium UKL13-1
13	Bacteria	Proteobacteria	Alphaproteobacteria	Rhodobacterales	Hyphomonadaceae	Henriciella	Henriciella aquimarina
13	Bacteria	Proteobacteria	Alphaproteobacteria	Rhizobiales	Rhodobiaceae	Afifella	Afifella marina
13	Bacteria	Proteobacteria	Alphaproteobacteria	Rhizobiales	Rhizobiaceae	Sinorhizobium/Ensifer group	Ensifer
13	Bacteria	Proteobacteria	Alphaproteobacteria	Rhizobiales	Phyllobacteriaceae	Nitratireductor	Nitratireductor indicus
13	Bacteria	Proteobacteria	Alphaproteobacteria	Rhizobiales	Phyllobacteriaceae	Mesorhizobium	Mesorhizobium plurifarium
13	Bacteria	Proteobacteria	Alphaproteobacteria	Rhizobiales	Phyllobacteriaceae	Chelativorans	Chelativorans sp. BNC1
13	Bacteria	Proteobacteria	Alphaproteobacteria	Rhizobiales	Methylobacteriaceae	Microvirga	Microvirga lupini
13	Bacteria	Proteobacteria	Alphaproteobacteria	Rhizobiales	Hyphomicrobiaceae	Pelagibacterium	
13	Bacteria	Proteobacteria	Alphaproteobacteria	Rhizobiales	Bradyrhizobiaceae	Rhodopseudomonas	
13	Bacteria	Proteobacteria	Alphaproteobacteria	Rhizobiales	Beijerinckiaceae	Methylocella	Methylocella silvestris
13	Bacteria	Proteobacteria	Alphaproteobacteria	Rhizobiales	Beijerinckiaceae	Methylocapsa	Methylocapsa acidiphila
13	Bacteria	Proteobacteria	Alphaproteobacteria	Rhizobiales	Beijerinckiaceae	Beijerinckia	Beijerinckia mobilis
13	Bacteria	Proteobacteria	Alphaproteobacteria	Rhizobiales	Bartonellaceae	Bartonella	
13	Bacteria	Proteobacteria	Alphaproteobacteria	Kiloniellales	Kiloniellaceae	Kiloniella	Kiloniella laminariae
13	Bacteria	Proteobacteria	Acidithiobacillia	Acidithiobacillales	Thermithiobacillaceae	Thermithiobacillus	Thermithiobacillus tepidarius
13	Bacteria	PVC group	Verrucomicrobia	Opitutae	Puniceicoccales	Puniceicoccaceae	Coraliomargarita
13	Bacteria	PVC group	Planctomycetes	unclassified Planctomycetes	Planctomycetes bacterium RBG_16_64_10		
13	Bacteria	PVC group	Planctomycetes	Planctomycetia	Planctomycetales	Isosphaeraceae	Singulisphaera
13	Bacteria	PVC group	Planctomycetes	Phycisphaerae	unclassified Phycisphaerae	Phycisphaerae bacterium SM23_33	
13	Bacteria	Nitrospirae	unclassified Nitrospirae	Nitrospirae bacterium RBG_16_64_22			
13	Bacteria	FCB group	Bacteroidetes/Chlorobi group	Bacteroidetes	unclassified Bacteroidetes	unclassified Bacteroidetes (miscellaneous)	Bacteroidetes bacterium 4484_249
13	Bacteria	FCB group	Bacteroidetes/Chlorobi group	Bacteroidetes	Chitinophagia	Chitinophagales	Chitinophagaceae
13	Bacteria	FCB group	Bacteroidetes/Chlorobi group	Bacteroidetes	Chitinophagia	Chitinophagales	Chitinophagaceae
13	Bacteria	FCB group	Bacteroidetes/Chlorobi group	Bacteroidetes	Bacteroidia	Marinilabiliales	Prolixibacteraceae
13	Bacteria	FCB group	Bacteroidetes/Chlorobi group	Bacteroidetes	Bacteroidia	Marinilabiliales	Marinilabiliaceae
13	Bacteria	FCB group	Bacteroidetes/Chlorobi group	Bacteroidetes	Bacteroidia	Bacteroidales	Prevotellaceae
13	Bacteria	FCB group	Bacteroidetes/Chlorobi group	Bacteroidetes	Bacteroidia	Bacteroidales	Porphyromonadaceae
13	Bacteria	FCB group	Bacteroidetes/Chlorobi group	Bacteroidetes	Bacteroidia	Bacteroidales	Porphyromonadaceae
13	Bacteria	FCB group	Bacteroidetes/Chlorobi group	Bacteroidetes	Bacteroidia	Bacteroidales	Bacteroidaceae
13	Bacteria	FCB group	Bacteroidetes/Chlorobi group	Bacteroidetes	Bacteroidia	Bacteroidales	Bacteroidaceae
13	Bacteria	Elusimicrobia	unclassified Elusimicrobia	Elusimicrobia bacterium RIFOXYB2_FULL_49_7			
13	Bacteria	Calditrichaeota	Calditrichae	Calditrichales	Calditrichaceae	Caldithrix	Caldithrix abyssi
13	Bacteria	Acidobacteria	Holophagae	Holophagales	Holophagaceae	Holophaga	Holophaga foetida
12	Eukaryota	Rhodophyta	Florideophyceae				
12	Eukaryota	Opisthokonta	Fungi	Dikarya			
12	Bacteria	environmental samples	uncultured bacterium A1Q1_fos_4				
12	Bacteria	environmental samples	uncultured bacterium SH7				
12	Bacteria	Terrabacteria group	Firmicutes	Clostridia	Thermoanaerobacterales	Thermoanaerobacterales Family III. Incertae Sedis	Syntrophaceticus
12	Bacteria	Terrabacteria group	Firmicutes	Clostridia	Clostridiales	Ruminococcaceae	Ruminococcus
12	Bacteria	Terrabacteria group	Firmicutes	Clostridia	Clostridiales	Ruminococcaceae	Ruminococcus
12	Bacteria	Terrabacteria group	Firmicutes	Clostridia	Clostridiales	Ruminococcaceae	Ruminiclostridium
12	Bacteria	Terrabacteria group	Firmicutes	Clostridia	Clostridiales	Ruminococcaceae	Anaerotruncus
12	Bacteria	Terrabacteria group	Firmicutes	Clostridia	Clostridiales	Christensenellaceae	Christensenella
12	Bacteria	Terrabacteria group	Firmicutes	Clostridia	Clostridiales	Christensenellaceae	Christensenella
12	Bacteria	Terrabacteria group	Firmicutes	Bacilli	Bacillales	Bacillaceae	Bacillus
12	Bacteria	Terrabacteria group	Firmicutes	Bacilli			
12	Bacteria	Terrabacteria group	Cyanobacteria/Melainabacteria group	Cyanobacteria	Nostocales	Nostocaceae	Anabaena
12	Bacteria	Terrabacteria group	Actinobacteria	Thermoleophilia	Solirubrobacterales	unclassified Solirubrobacterales	Solirubrobacterales bacterium URHD0059
12	Bacteria	Terrabacteria group	Actinobacteria	Actinobacteria	Propionibacteriales	Nocardioidaceae	Nocardioides
12	Bacteria	Terrabacteria group	Actinobacteria	Actinobacteria	Micrococcales	Micrococcaceae	Arthrobacter
12	Bacteria	Spirochaetes	unclassified Spirochaetes	Spirochaetes bacterium GWB1_36_13			
12	Bacteria	Spirochaetes	Spirochaetia	Spirochaetales	Spirochaetaceae	Spirochaeta	Spirochaeta odontotermitis
12	Bacteria	Spirochaetes	Spirochaetia	Spirochaetales	Spirochaetaceae	Candidatus Marispirochaeta	Candidatus Marispirochaeta associata
12	Bacteria	Spirochaetes	Spirochaetia	Leptospirales	Leptospiraceae	Turneriella	Turneriella parva
12	Bacteria	Proteobacteria	delta/epsilon subdivisions	Epsilonproteobacteria	Campylobacterales	Helicobacteraceae	Helicobacter
12	Bacteria	Proteobacteria	delta/epsilon subdivisions	Deltaproteobacteria	unclassified Deltaproteobacteria	unclassified Deltaproteobacteria (miscellaneous)	delta proteobacterium MLMS-1
12	Bacteria	Proteobacteria	delta/epsilon subdivisions	Deltaproteobacteria	unclassified Deltaproteobacteria	Dissulfuribacter	Dissulfuribacter thermophilus
12	Bacteria	Proteobacteria	delta/epsilon subdivisions	Deltaproteobacteria	Syntrophobacterales	Syntrophobacteraceae	unclassified Syntrophobacteraceae
12	Bacteria	Proteobacteria	delta/epsilon subdivisions	Deltaproteobacteria	Syntrophobacterales	Syntrophobacteraceae	Syntrophobacter
12	Bacteria	Proteobacteria	delta/epsilon subdivisions	Deltaproteobacteria	Syntrophobacterales	Syntrophobacteraceae	Desulfacinum
12	Bacteria	Proteobacteria	delta/epsilon subdivisions	Deltaproteobacteria	Myxococcales	Cystobacterineae	Vulgatibacteraceae
12	Bacteria	Proteobacteria	delta/epsilon subdivisions	Deltaproteobacteria	Myxococcales	Cystobacterineae	
12	Bacteria	Proteobacteria	delta/epsilon subdivisions	Deltaproteobacteria	Desulfovibrionales	Desulfovibrionaceae	Desulfovibrio
12	Bacteria	Proteobacteria	delta/epsilon subdivisions	Deltaproteobacteria	Desulfovibrionales	Desulfovibrionaceae	Desulfovibrio
12	Bacteria	Proteobacteria	delta/epsilon subdivisions	Deltaproteobacteria	Desulfobacterales	Desulfobulbaceae	Desulfobulbus
12	Bacteria	Proteobacteria	delta/epsilon subdivisions	Deltaproteobacteria	Desulfobacterales	Desulfobacteraceae	Desulfoluna
12	Bacteria	Proteobacteria	delta/epsilon subdivisions	Deltaproteobacteria	Desulfobacterales	Desulfobacteraceae	Desulfobacula
12	Bacteria	Proteobacteria	delta/epsilon subdivisions	Deltaproteobacteria	Desulfobacterales	Desulfobacteraceae	Desulfobacterium
12	Bacteria	Proteobacteria	Gammaproteobacteria	unclassified Gammaproteobacteria	unclassified Gammaproteobacteria (miscellaneous)	gamma proteobacterium NOR5-3	
12	Bacteria	Proteobacteria	Gammaproteobacteria	unclassified Gammaproteobacteria	unclassified Gammaproteobacteria (miscellaneous)	Gammaproteobacteria bacterium SG8_11	
12	Bacteria	Proteobacteria	Gammaproteobacteria	unclassified Gammaproteobacteria	unclassified Gammaproteobacteria (miscellaneous)	Gammaproteobacteria bacterium RBG_16_51_14	
12	Bacteria	Proteobacteria	Gammaproteobacteria	Xanthomonadales	Xanthomonadaceae	Silanimonas	Silanimonas lenta
12	Bacteria	Proteobacteria	Gammaproteobacteria	Vibrionales	Vibrionaceae	Vibrio	Vibrio nigripulchritudo
12	Bacteria	Proteobacteria	Gammaproteobacteria	Thiotrichales	Thiotrichaceae	Beggiatoa	Beggiatoa sp. 4572_84
12	Bacteria	Proteobacteria	Gammaproteobacteria	Pseudomonadales	Pseudomonadaceae	Pseudomonas	Pseudomonas sp. BMS12
12	Bacteria	Proteobacteria	Gammaproteobacteria	Pseudomonadales	Pseudomonadaceae	Pseudomonas	Pseudomonas indica
12	Bacteria	Proteobacteria	Gammaproteobacteria	Oceanospirillales	Hahellaceae	Hahella	Hahella ganghwensis
12	Bacteria	Proteobacteria	Gammaproteobacteria	Methylococcales	Methylococcaceae	Methylomonas	Methylomonas lenta
12	Bacteria	Proteobacteria	Gammaproteobacteria	Methylococcales	Methylococcaceae	Methylocaldum	Methylocaldum szegediense
12	Bacteria	Proteobacteria	Gammaproteobacteria	Immundisolibacterales	Immundisolibacteraceae	Immundisolibacter	Immundisolibacter cernigliae
12	Bacteria	Proteobacteria	Gammaproteobacteria	Enterobacterales	Pectobacteriaceae	Dickeya	
12	Bacteria	Proteobacteria	Gammaproteobacteria	Enterobacterales	Budviciaceae	Pragia	Pragia fontium
12	Bacteria	Proteobacteria	Gammaproteobacteria	Enterobacterales	Budviciaceae	Budvicia	Budvicia aquatica
12	Bacteria	Proteobacteria	Gammaproteobacteria	Chromatiales	Wenzhouxiangellaceae	Wenzhouxiangella	Wenzhouxiangella marina
12	Bacteria	Proteobacteria	Gammaproteobacteria	Chromatiales	Ectothiorhodospiraceae	Thiohalomonas	Thiohalomonas denitrificans
12	Bacteria	Proteobacteria	Gammaproteobacteria	Chromatiales	Ectothiorhodospiraceae	Aquisalimonas	Aquisalimonas asiatica
12	Bacteria	Proteobacteria	Gammaproteobacteria	Chromatiales	Chromatiaceae	Thioflavicoccus	Thioflavicoccus mobilis
12	Bacteria	Proteobacteria	Gammaproteobacteria	Cellvibrionales	Spongiibacteraceae	Oceanicoccus	Oceanicoccus sagamiensis
12	Bacteria	Proteobacteria	Gammaproteobacteria	Cellvibrionales	Spongiibacteraceae	Dasania	Dasania marina
12	Bacteria	Proteobacteria	Gammaproteobacteria	Cellvibrionales	Halieaceae	unclassified Halieaceae	marine gamma proteobacterium HTCC2080
12	Bacteria	Proteobacteria	Gammaproteobacteria	Cellvibrionales	Cellvibrionaceae	Cellvibrio	Cellvibrio japonicus
12	Bacteria	Proteobacteria	Gammaproteobacteria	Alteromonadales	Alteromonadaceae	Catenovulum	Catenovulum agarivorans
12	Bacteria	Proteobacteria	Gammaproteobacteria	Aeromonadales	Aeromonadaceae	Aeromonas	Aeromonas sp. RU39B
12	Bacteria	Proteobacteria	Betaproteobacteria	unclassified Betaproteobacteria	unclassified Betaproteobacteria (miscellaneous)	Betaproteobacteria bacterium RIFCSPLOWO2_12_FULL_66_14	
12	Bacteria	Proteobacteria	Betaproteobacteria	unclassified Betaproteobacteria	unclassified Betaproteobacteria (miscellaneous)	Betaproteobacteria bacterium RBG_16_56_24	
12	Bacteria	Proteobacteria	Betaproteobacteria	unclassified Betaproteobacteria	Candidatus Accumulibacter	Candidatus Accumulibacter sp. SK-02	
12	Bacteria	Proteobacteria	Betaproteobacteria	unclassified Betaproteobacteria	Candidatus Accumulibacter	Candidatus Accumulibacter sp. SK-01	
12	Bacteria	Proteobacteria	Betaproteobacteria	unclassified Betaproteobacteria	Candidatus Accumulibacter	Candidatus Accumulibacter sp. BA-93	
12	Bacteria	Proteobacteria	Betaproteobacteria	Nitrosomonadales	Nitrosomonadaceae	Nitrosovibrio	Nitrosovibrio sp. Nv17
12	Bacteria	Proteobacteria	Betaproteobacteria	Nitrosomonadales	Nitrosomonadaceae	Nitrosomonas	Nitrosomonas mobilis
12	Bacteria	Proteobacteria	Betaproteobacteria	Nitrosomonadales	Nitrosomonadaceae	Nitrosomonas	Nitrosomonas communis
12	Bacteria	Proteobacteria	Betaproteobacteria	Neisseriales	Neisseriaceae	Amantichitinum	Amantichitinum ursilacus
12	Bacteria	Proteobacteria	Betaproteobacteria	Neisseriales	Chromobacteriaceae	Pseudogulbenkiania	Pseudogulbenkiania sp. MAI-1
12	Bacteria	Proteobacteria	Betaproteobacteria	Neisseriales	Chromobacteriaceae	Chitinilyticum	Chitinilyticum aquatile
12	Bacteria	Proteobacteria	Betaproteobacteria	Neisseriales	Chromobacteriaceae	Andreprevotia	Andreprevotia lacus
12	Bacteria	Proteobacteria	Betaproteobacteria	Methylophilales	unclassified Methylophilales		
12	Bacteria	Proteobacteria	Betaproteobacteria	Methylophilales	Methylophilaceae	unclassified Methylophilaceae	Methylophilaceae bacterium 11
12	Bacteria	Proteobacteria	Betaproteobacteria	Gallionellales	Gallionellaceae	Ferriphaselus	Ferriphaselus sp. R-1
12	Bacteria	Proteobacteria	Betaproteobacteria	Ferrovales	Ferrovaceae	Ferrovum	Ferrovum sp. JA12
12	Bacteria	Proteobacteria	Betaproteobacteria	Burkholderiales	unclassified Burkholderiales	unclassified Burkholderiales (miscellaneous)	Burkholderiales bacterium 68-12
12	Bacteria	Proteobacteria	Betaproteobacteria	Burkholderiales	Comamonadaceae	Acidovorax	Acidovorax sp. KKS102
12	Bacteria	Proteobacteria	Betaproteobacteria	Burkholderiales	Burkholderiaceae	Polynucleobacter	Polynucleobacter asymbioticus
12	Bacteria	Proteobacteria	Betaproteobacteria	Burkholderiales	Burkholderiaceae	Paraburkholderia	Paraburkholderia kururiensis
12	Bacteria	Proteobacteria	Betaproteobacteria	Burkholderiales	Burkholderiaceae	Limnobacter	Limnobacter sp. MED105
12	Bacteria	Proteobacteria	Betaproteobacteria	Burkholderiales	Burkholderiaceae	Burkholderia	Burkholderia gladioli
12	Bacteria	Proteobacteria	Betaproteobacteria	Burkholderiales	Alcaligenaceae	Kerstersia	Kerstersia gyiorum
12	Bacteria	Proteobacteria	Betaproteobacteria	Burkholderiales	Alcaligenaceae	Castellaniella	Castellaniella defragrans
12	Bacteria	Proteobacteria	Betaproteobacteria	Burkholderiales	Alcaligenaceae	Bordetella	Bordetella genomosp. 9
12	Bacteria	Proteobacteria	Betaproteobacteria	Burkholderiales	Alcaligenaceae	Bordetella	Bordetella genomosp. 8
12	Bacteria	Proteobacteria	Alphaproteobacteria	unclassified Alphaproteobacteria	unclassified Alphaproteobacteria (miscellaneous)	Alphaproteobacteria bacterium 62-8	
12	Bacteria	Proteobacteria	Alphaproteobacteria	Sphingomonadales	Sphingomonadaceae	Sphingomonas	Sphingomonas sp. 67-41
12	Bacteria	Proteobacteria	Alphaproteobacteria	Sphingomonadales	Sphingomonadaceae	Sphingobium	Sphingobium sp. Ant17
12	Bacteria	Proteobacteria	Alphaproteobacteria	Sphingomonadales	Sphingomonadaceae	Blastomonas	Blastomonas sp. AAP25
12	Bacteria	Proteobacteria	Alphaproteobacteria	Rhodospirillales	Rhodospirillaceae	Rhodospirillum	Rhodospirillum centenum
12	Bacteria	Proteobacteria	Alphaproteobacteria	Rhodospirillales	Rhodospirillaceae	Phaeospirillum	Phaeospirillum fulvum
12	Bacteria	Proteobacteria	Alphaproteobacteria	Rhodospirillales	Rhodospirillaceae	Pararhodospirillum	Pararhodospirillum photometricum
12	Bacteria	Proteobacteria	Alphaproteobacteria	Rhodospirillales	Rhodospirillaceae	Nitrospirillum	Nitrospirillum amazonense
12	Bacteria	Proteobacteria	Alphaproteobacteria	Rhodospirillales	Rhodospirillaceae	Magnetospirillum	Magnetospirillum gryphiswaldense
12	Bacteria	Proteobacteria	Alphaproteobacteria	Rhodospirillales	Rhodospirillaceae	Fodinicurvata	
12	Bacteria	Proteobacteria	Alphaproteobacteria	Rhodospirillales	Rhodospirillaceae	Azospirillum	Azospirillum sp. B4
12	Bacteria	Proteobacteria	Alphaproteobacteria	Rhodospirillales	Acetobacteraceae	unclassified Acetobacteraceae	Acetobacteraceae bacterium AT-5844
12	Bacteria	Proteobacteria	Alphaproteobacteria	Rhodospirillales	Acetobacteraceae	Belnapia	Belnapia moabensis
12	Bacteria	Proteobacteria	Alphaproteobacteria	Rhodospirillales	Acetobacteraceae	Acetobacter	environmental samples
12	Bacteria	Proteobacteria	Alphaproteobacteria	Rhodospirillales	Acetobacteraceae	Acetobacter	
12	Bacteria	Proteobacteria	Alphaproteobacteria	Rhodobacterales	Rhodobacteraceae	unclassified Rhodobacteraceae	Rhodobacteraceae bacterium O3.65
12	Bacteria	Proteobacteria	Alphaproteobacteria	Rhodobacterales	Rhodobacteraceae	unclassified Rhodobacteraceae	Rhodobacteraceae bacterium HTCC2083
12	Bacteria	Proteobacteria	Alphaproteobacteria	Rhodobacterales	Rhodobacteraceae	Thalassobius	Thalassobius maritimus
12	Bacteria	Proteobacteria	Alphaproteobacteria	Rhodobacterales	Rhodobacteraceae	Sulfitobacter	Sulfitobacter litoralis
12	Bacteria	Proteobacteria	Alphaproteobacteria	Rhodobacterales	Rhodobacteraceae	Roseovarius	Roseovarius sp. A-2
12	Bacteria	Proteobacteria	Alphaproteobacteria	Rhodobacterales	Rhodobacteraceae	Rhodobacter	Rhodobacter johrii
12	Bacteria	Proteobacteria	Alphaproteobacteria	Rhodobacterales	Rhodobacteraceae	Pseudoruegeria	Pseudoruegeria sabulilitoris
12	Bacteria	Proteobacteria	Alphaproteobacteria	Rhodobacterales	Rhodobacteraceae	Ahrensia	Ahrensia marina
12	Bacteria	Proteobacteria	Alphaproteobacteria	Rhodobacterales	Hyphomonadaceae	Oceanicaulis	
12	Bacteria	Proteobacteria	Alphaproteobacteria	Rhodobacterales	Hyphomonadaceae	Maricaulis	Maricaulis salignorans
12	Bacteria	Proteobacteria	Alphaproteobacteria	Rhizobiales	unclassified Rhizobiales	unclassified Rhizobiales (miscellaneous)	Rhizobiales bacterium 65-79
12	Bacteria	Proteobacteria	Alphaproteobacteria	Rhizobiales	unclassified Rhizobiales	unclassified Rhizobiales (miscellaneous)	Rhizobiales bacterium 63-7
12	Bacteria	Proteobacteria	Alphaproteobacteria	Rhizobiales	unclassified Rhizobiales	Methyloceanibacter	Methyloceanibacter superfactus
12	Bacteria	Proteobacteria	Alphaproteobacteria	Rhizobiales	Rhizobiaceae	Rhizobium/Agrobacterium group	Agrobacterium
12	Bacteria	Proteobacteria	Alphaproteobacteria	Rhizobiales	Phyllobacteriaceae	Nitratireductor	
12	Bacteria	Proteobacteria	Alphaproteobacteria	Rhizobiales	Methylobacteriaceae	Microvirga	Microvirga guangxiensis
12	Bacteria	Proteobacteria	Alphaproteobacteria	Rhizobiales	Methylobacteriaceae	Microvirga	Microvirga flocculans
12	Bacteria	Proteobacteria	Alphaproteobacteria	Rhizobiales	Hyphomicrobiaceae	Hyphomicrobium	Hyphomicrobium sp. GJ21
12	Bacteria	Proteobacteria	Alphaproteobacteria	Rhizobiales	Bradyrhizobiaceae	Bradyrhizobium	Bradyrhizobium sp. ARR65
12	Bacteria	Proteobacteria	Alphaproteobacteria	Rhizobiales	Bradyrhizobiaceae	Bradyrhizobium	Bradyrhizobium lablabi
12	Bacteria	Proteobacteria	Alphaproteobacteria	Rhizobiales	Bradyrhizobiaceae	Bosea	Bosea sp. DSM 18164
12	Bacteria	Proteobacteria	Alphaproteobacteria	Rhizobiales	Beijerinckiaceae	Methylocapsa	Methylocapsa palsarum
12	Bacteria	Proteobacteria	Alphaproteobacteria	Pelagibacterales	Pelagibacteraceae	Candidatus Pelagibacter	Candidatus Pelagibacter ubique
12	Bacteria	PVC group	Verrucomicrobia	unclassified Verrucomicrobia	unclassified Verrucomicrobia (miscellaneous)	Verrucomicrobia bacterium RIFCSPLOWO2_12_FULL_64_8	
12	Bacteria	PVC group	Verrucomicrobia	unclassified Verrucomicrobia	unclassified Verrucomicrobia (miscellaneous)	Verrucomicrobia bacterium RIFCSPHIGHO2_12_FULL_41_10	
12	Bacteria	PVC group	Planctomycetes	Planctomycetia	Planctomycetales	Planctomycetaceae	Planctomyces
12	Bacteria	PVC group	Planctomycetes	Planctomycetia	Planctomycetales		
12	Bacteria	PVC group	Chlamydiae	Chlamydiia	Chlamydiales	unclassified Chlamydiales	unclassified Chlamydiales (miscellaneous)
12	Bacteria	Fusobacteria	Fusobacteriia	Fusobacteriales	Leptotrichiaceae	Sebaldella	Sebaldella termitidis
12	Bacteria	FCB group	Bacteroidetes/Chlorobi group	Chlorobi	Chlorobia	Chlorobiales	Chlorobiaceae
12	Bacteria	FCB group	Bacteroidetes/Chlorobi group	Bacteroidetes	unclassified Bacteroidetes	unclassified Bacteroidetes (miscellaneous)	Bacteroidetes bacterium CG2_30_33_31
12	Bacteria	FCB group	Bacteroidetes/Chlorobi group	Bacteroidetes	unclassified Bacteroidetes	unclassified Bacteroidetes (miscellaneous)	Bacteroidetes bacterium 4484_276
12	Bacteria	FCB group	Bacteroidetes/Chlorobi group	Bacteroidetes	Bacteroidia	Marinilabiliales	Marinilabiliaceae
12	Bacteria	FCB group	Bacteroidetes/Chlorobi group	Bacteroidetes	Bacteroidia	Bacteroidales	unclassified Bacteroidales
12	Bacteria	FCB group	Bacteroidetes/Chlorobi group	Bacteroidetes	Bacteroidia	Bacteroidales	Prevotellaceae
12	Bacteria	FCB group	Bacteroidetes/Chlorobi group	Bacteroidetes	Bacteroidia	Bacteroidales	Porphyromonadaceae
12	Bacteria	FCB group	Bacteroidetes/Chlorobi group	Bacteroidetes	Bacteroidia	Bacteroidales	Porphyromonadaceae
12	Bacteria	FCB group	Bacteroidetes/Chlorobi group	Bacteroidetes	Bacteroidia	Bacteroidales	Porphyromonadaceae
12	Bacteria	FCB group	Bacteroidetes/Chlorobi group	Bacteroidetes	Bacteroidia	Bacteroidales	Bacteroidaceae
12	Bacteria	FCB group	Bacteroidetes/Chlorobi group	Bacteroidetes	Bacteroidia	Bacteroidales	Bacteroidaceae
12	Bacteria	FCB group	Bacteroidetes/Chlorobi group	Bacteroidetes	Bacteroidia	Bacteroidales	Bacteroidaceae
12	Bacteria	Acidobacteria	Blastocatellia	Pyrinomonas	Pyrinomonas methylaliphatogenes		
12	dsDNA viruses, no RNA stage	Phycodnaviridae	Raphidovirus	Heterosigma akashiwo virus 01			
11	Eukaryota	Rhodophyta	Florideophyceae	Plocamiales	Plocamiaceae	Plocamium	Plocamium cf. leptophyllum 1 GWS-2011
11	Eukaryota	Rhodophyta	Florideophyceae	Ceramiales	Ceramiaceae	Ceramium	Ceramium cimbricum
11	Eukaryota	Opisthokonta	Fungi	Dikarya	Ascomycota	saccharomyceta	Pezizomycotina
11	Bacteria	unclassified Bacteria	Bacteria candidate phyla	Patescibacteria group	Parcubacteria group	Candidatus Moranbacteria	
11	Bacteria	environmental samples	uncultured bacterium WWRS-2005				
11	Bacteria	environmental samples	uncultured bacterium A1Q1_fos_1815				
11	Bacteria	Terrabacteria group	Firmicutes	Negativicutes	Selenomonadales	Sporomusaceae	Acetonema
11	Bacteria	Terrabacteria group	Firmicutes	Clostridia	Clostridiales	unclassified Clostridiales	unclassified Clostridiales (miscellaneous)
11	Bacteria	Terrabacteria group	Firmicutes	Clostridia	Clostridiales	unclassified Clostridiales	Epulopiscium
11	Bacteria	Terrabacteria group	Firmicutes	Clostridia	Clostridiales	Peptostreptococcaceae	Clostridioides
11	Bacteria	Terrabacteria group	Firmicutes	Clostridia	Clostridiales	Peptococcaceae	Desulfotomaculum
11	Bacteria	Terrabacteria group	Firmicutes	Clostridia	Clostridiales	Peptococcaceae	Desulfotomaculum
11	Bacteria	Terrabacteria group	Firmicutes	Clostridia	Clostridiales	Peptococcaceae	Desulfosporosinus
11	Bacteria	Terrabacteria group	Firmicutes	Clostridia	Clostridiales	Peptococcaceae	Desulfitibacter
11	Bacteria	Terrabacteria group	Firmicutes	Clostridia	Clostridiales	Clostridiales incertae sedis	Clostridiales Family XII. Incertae Sedis
11	Bacteria	Terrabacteria group	Firmicutes	Clostridia	Clostridiales	Clostridiaceae	Clostridium
11	Bacteria	Terrabacteria group	Firmicutes	Clostridia	Clostridiales	Clostridiaceae	Clostridium
11	Bacteria	Terrabacteria group	Deinococcus-Thermus	Deinococci	Thermales	Thermaceae	Meiothermus
11	Bacteria	Terrabacteria group	Deinococcus-Thermus	Deinococci	Deinococcales	Trueperaceae	Truepera
11	Bacteria	Terrabacteria group	Cyanobacteria/Melainabacteria group	Cyanobacteria	Oscillatoriophycideae	Oscillatoriales	
11	Bacteria	Terrabacteria group	Chloroflexi	Dehalococcoidia	Dehalococcoidales	Dehalococcoidaceae	Dehalococcoides
11	Bacteria	Terrabacteria group	Actinobacteria	Actinobacteria	Pseudonocardiales	Pseudonocardiaceae	Amycolatopsis
11	Bacteria	Terrabacteria group	Actinobacteria	Actinobacteria	Micrococcales	Microbacteriaceae	
11	Bacteria	Spirochaetes	Spirochaetia	Spirochaetales	Spirochaetaceae	Treponema	Treponema caldarium
11	Bacteria	Proteobacteria	unclassified Proteobacteria	unclassified Proteobacteria (miscellaneous)	Proteobacteria bacterium CG1_02_64_396		
11	Bacteria	Proteobacteria	delta/epsilon subdivisions	Deltaproteobacteria	unclassified Deltaproteobacteria	unclassified Deltaproteobacteria (miscellaneous)	delta proteobacterium ML8_D
11	Bacteria	Proteobacteria	delta/epsilon subdivisions	Deltaproteobacteria	unclassified Deltaproteobacteria	unclassified Deltaproteobacteria (miscellaneous)	Deltaproteobacteria bacterium SM23_61
11	Bacteria	Proteobacteria	delta/epsilon subdivisions	Deltaproteobacteria	unclassified Deltaproteobacteria	unclassified Deltaproteobacteria (miscellaneous)	Deltaproteobacteria bacterium RIFOXYD12_FULL_53_23
11	Bacteria	Proteobacteria	delta/epsilon subdivisions	Deltaproteobacteria	unclassified Deltaproteobacteria	Deferrisoma	Deferrisoma camini
11	Bacteria	Proteobacteria	delta/epsilon subdivisions	Deltaproteobacteria	unclassified Deltaproteobacteria	Candidatus Desulfamplus	Candidatus Desulfamplus magnetomortis
11	Bacteria	Proteobacteria	delta/epsilon subdivisions	Deltaproteobacteria	Syntrophobacterales	Syntrophaceae	Syntrophus
11	Bacteria	Proteobacteria	delta/epsilon subdivisions	Deltaproteobacteria	Syntrophobacterales	Syntrophaceae	Desulfobacca
11	Bacteria	Proteobacteria	delta/epsilon subdivisions	Deltaproteobacteria	Myxococcales	unclassified Myxococcales	unclassified Myxococcales (miscellaneous)
11	Bacteria	Proteobacteria	delta/epsilon subdivisions	Deltaproteobacteria	Myxococcales	Cystobacterineae	Myxococcaceae
11	Bacteria	Proteobacteria	delta/epsilon subdivisions	Deltaproteobacteria	Myxococcales	Cystobacterineae	Archangiaceae
11	Bacteria	Proteobacteria	delta/epsilon subdivisions	Deltaproteobacteria	Myxococcales	Cystobacterineae	Archangiaceae
11	Bacteria	Proteobacteria	delta/epsilon subdivisions	Deltaproteobacteria	Desulfovibrionales	Desulfovibrionaceae	Desulfovibrio
11	Bacteria	Proteobacteria	delta/epsilon subdivisions	Deltaproteobacteria	Desulfobacterales	unclassified Desulfobacterales	
11	Bacteria	Proteobacteria	delta/epsilon subdivisions	Deltaproteobacteria	Desulfobacterales	Desulfobulbaceae	Desulforhopalus
11	Bacteria	Proteobacteria	delta/epsilon subdivisions	Deltaproteobacteria	Desulfobacterales	Desulfobacteraceae	Desulfosarcina
11	Bacteria	Proteobacteria	delta/epsilon subdivisions	Deltaproteobacteria	Desulfobacterales	Desulfobacteraceae	Desulfobacter
11	Bacteria	Proteobacteria	delta/epsilon subdivisions	Deltaproteobacteria	Desulfobacterales	Desulfobacteraceae	Desulfatibacillum
11	Bacteria	Proteobacteria	Gammaproteobacteria	unclassified Gammaproteobacteria	unclassified Gammaproteobacteria (miscellaneous)	endosymbiont of unidentified scaly snail isolate Monju	
11	Bacteria	Proteobacteria	Gammaproteobacteria	unclassified Gammaproteobacteria	unclassified Gammaproteobacteria (miscellaneous)	Gammaproteobacteria bacterium SG8_47	
11	Bacteria	Proteobacteria	Gammaproteobacteria	unclassified Gammaproteobacteria	Pseudohongiella	Pseudohongiella spirulinae	
11	Bacteria	Proteobacteria	Gammaproteobacteria	unclassified Gammaproteobacteria	Candidatus Thioglobus	environmental samples	uncultured SUP05 cluster bacterium
11	Bacteria	Proteobacteria	Gammaproteobacteria	Xanthomonadales	Xanthomonadaceae	Pseudoxanthomonas	Pseudoxanthomonas spadix
11	Bacteria	Proteobacteria	Gammaproteobacteria	Xanthomonadales	Xanthomonadaceae	Lysobacter	Lysobacter gummosus
11	Bacteria	Proteobacteria	Gammaproteobacteria	Xanthomonadales	Xanthomonadaceae	Lysobacter	Lysobacter capsici
11	Bacteria	Proteobacteria	Gammaproteobacteria	Xanthomonadales	Xanthomonadaceae	Luteimonas	Luteimonas sp. FCS-9
11	Bacteria	Proteobacteria	Gammaproteobacteria	Xanthomonadales	Xanthomonadaceae	Arenimonas	Arenimonas composti
11	Bacteria	Proteobacteria	Gammaproteobacteria	Thiotrichales	Thiotrichaceae	Beggiatoa	Beggiatoa sp. PS
11	Bacteria	Proteobacteria	Gammaproteobacteria	Pseudomonadales	unclassified Pseudomonadales	unclassified Pseudomonadales (miscellaneous)	Pseudomonadales bacterium RIFCSPLOWO2_12_59_9
11	Bacteria	Proteobacteria	Gammaproteobacteria	Pseudomonadales	Pseudomonadaceae	Pseudomonas	Pseudomonas psychrotolerans
11	Bacteria	Proteobacteria	Gammaproteobacteria	Pseudomonadales	Moraxellaceae	Perlucidibaca	Perlucidibaca piscinae
11	Bacteria	Proteobacteria	Gammaproteobacteria	Pasteurellales	Pasteurellaceae		
11	Bacteria	Proteobacteria	Gammaproteobacteria	Oceanospirillales	Oceanospirillaceae	Balneatrix	Balneatrix alpica
11	Bacteria	Proteobacteria	Gammaproteobacteria	Nevskiales	Sinobacteraceae	Solimonas	
11	Bacteria	Proteobacteria	Gammaproteobacteria	Methylococcales	Methylococcaceae	Methylobacter	Methylobacter luteus
11	Bacteria	Proteobacteria	Gammaproteobacteria	Enterobacterales	Yersiniaceae	Rahnella	Rahnella sp. J11-6
11	Bacteria	Proteobacteria	Gammaproteobacteria	Enterobacterales	Budviciaceae	Leminorella	Leminorella grimontii
11	Bacteria	Proteobacteria	Gammaproteobacteria	Chromatiales	Ectothiorhodospiraceae	Nitrococcus	Nitrococcus mobilis
11	Bacteria	Proteobacteria	Gammaproteobacteria	Chromatiales	Chromatiaceae	unclassified Chromatiaceae	Chromatiaceae bacterium 2141T.STBD.0c.01a
11	Bacteria	Proteobacteria	Gammaproteobacteria	Chromatiales	Chromatiaceae	Thiorhodovibrio	Thiorhodovibrio sp. 970
11	Bacteria	Proteobacteria	Gammaproteobacteria	Cellvibrionales	Spongiibacteraceae		
11	Bacteria	Proteobacteria	Gammaproteobacteria	Cellvibrionales	Halieaceae	Pseudohaliea	Pseudohaliea rubra
11	Bacteria	Proteobacteria	Gammaproteobacteria	Cellvibrionales	Halieaceae	Congregibacter	Congregibacter litoralis
11	Bacteria	Proteobacteria	Gammaproteobacteria	Cellvibrionales	Cellvibrionaceae	Teredinibacter	Teredinibacter sp. 1162T.S.0a.05
11	Bacteria	Proteobacteria	Gammaproteobacteria	Cellvibrionales	Cellvibrionaceae	Saccharophagus	Saccharophagus degradans
11	Bacteria	Proteobacteria	Gammaproteobacteria	Cellvibrionales	Cellvibrionaceae	Candidatus Endobugula	Candidatus Endobugula sertula
11	Bacteria	Proteobacteria	Gammaproteobacteria	Acidiferrobacterales	Acidiferrobacteraceae	Sulfuricaulis	Sulfuricaulis limicola
11	Bacteria	Proteobacteria	Gammaproteobacteria	Acidiferrobacterales	Acidiferrobacteraceae	Acidiferrobacter	Acidiferrobacter thiooxydans
11	Bacteria	Proteobacteria	Betaproteobacteria	unclassified Betaproteobacteria	unclassified Betaproteobacteria (miscellaneous)	Betaproteobacteria bacterium RIFCSPLOWO2_12_FULL_68_20	
11	Bacteria	Proteobacteria	Betaproteobacteria	unclassified Betaproteobacteria	unclassified Betaproteobacteria (miscellaneous)	Betaproteobacteria bacterium RIFCSPLOWO2_12_FULL_67_28	
11	Bacteria	Proteobacteria	Betaproteobacteria	unclassified Betaproteobacteria	unclassified Betaproteobacteria (miscellaneous)	Betaproteobacteria bacterium RIFCSPLOWO2_02_67_12	
11	Bacteria	Proteobacteria	Betaproteobacteria	unclassified Betaproteobacteria	unclassified Betaproteobacteria (miscellaneous)	Betaproteobacteria bacterium CG2_30_59_46	
11	Bacteria	Proteobacteria	Betaproteobacteria	environmental samples	uncultured beta proteobacterium CBNPD1 BAC clone 578		
11	Bacteria	Proteobacteria	Betaproteobacteria	Rhodocyclales	Rhodocyclaceae	Azoarcus	Azoarcus sp. PA01
11	Bacteria	Proteobacteria	Betaproteobacteria	Nitrosomonadales	Nitrosomonadaceae	Nitrosomonas	Nitrosomonas sp. Nm51
11	Bacteria	Proteobacteria	Betaproteobacteria	Neisseriales	Chromobacteriaceae	Pseudogulbenkiania	
11	Bacteria	Proteobacteria	Betaproteobacteria	Neisseriales	Chromobacteriaceae	Chitinilyticum	Chitinilyticum litopenaei
11	Bacteria	Proteobacteria	Betaproteobacteria	Methylophilales	unclassified Methylophilales	Methylophilales bacterium RIFCSPHIGHO2_02_FULL_57_10	
11	Bacteria	Proteobacteria	Betaproteobacteria	Methylophilales	Methylophilaceae	Methylobacillus	Methylobacillus glycogenes
11	Bacteria	Proteobacteria	Betaproteobacteria	Gallionellales	Gallionellaceae	unclassified Gallionellaceae	Gallionellaceae bacterium CG1_02_60_325
11	Bacteria	Proteobacteria	Betaproteobacteria	Burkholderiales	Oxalobacteraceae	Massilia	Massilia sp. Root418
11	Bacteria	Proteobacteria	Betaproteobacteria	Burkholderiales	Oxalobacteraceae	Massilia	Massilia sp. Root335
11	Bacteria	Proteobacteria	Betaproteobacteria	Burkholderiales	Oxalobacteraceae	Massilia	Massilia sp. KIM
11	Bacteria	Proteobacteria	Betaproteobacteria	Burkholderiales	Oxalobacteraceae	Massilia	Massilia sp. 9096
11	Bacteria	Proteobacteria	Betaproteobacteria	Burkholderiales	Comamonadaceae	Diaphorobacter	Diaphorobacter sp. J5-51
11	Bacteria	Proteobacteria	Betaproteobacteria	Burkholderiales	Comamonadaceae	Acidovorax	Acidovorax sp. Root217
11	Bacteria	Proteobacteria	Betaproteobacteria	Burkholderiales	Comamonadaceae	Acidovorax	Acidovorax cattleyae
11	Bacteria	Proteobacteria	Betaproteobacteria	Burkholderiales	Burkholderiaceae	Paraburkholderia	Paraburkholderia bannensis
11	Bacteria	Proteobacteria	Betaproteobacteria	Burkholderiales	Burkholderiaceae	Cupriavidus	Cupriavidus sp. YR651
11	Bacteria	Proteobacteria	Betaproteobacteria	Burkholderiales	Burkholderiaceae	Caballeronia	Caballeronia choica
11	Bacteria	Proteobacteria	Betaproteobacteria	Burkholderiales	Burkholderiaceae	Burkholderia	Burkholderia cepacia complex
11	Bacteria	Proteobacteria	Betaproteobacteria	Burkholderiales	Alcaligenaceae	Pusillimonas	Pusillimonas sp. T7-7
11	Bacteria	Proteobacteria	Betaproteobacteria	Burkholderiales	Alcaligenaceae	Bordetella	Bordetella sp. FB-8
11	Bacteria	Proteobacteria	Betaproteobacteria	Burkholderiales	Alcaligenaceae	Bordetella	Bordetella bronchiseptica
11	Bacteria	Proteobacteria	Alphaproteobacteria	Sphingomonadales	Sphingomonadaceae	Sphingomonas	Sphingomonas sanguinis
11	Bacteria	Proteobacteria	Alphaproteobacteria	Rhodospirillales	Rhodospirillaceae	Thalassobaculum	
11	Bacteria	Proteobacteria	Alphaproteobacteria	Rhodospirillales	Rhodospirillaceae	Oceanibaculum	Oceanibaculum pacificum
11	Bacteria	Proteobacteria	Alphaproteobacteria	Rhodospirillales	Acetobacteraceae	Roseomonas	Roseomonas sp. M3
11	Bacteria	Proteobacteria	Alphaproteobacteria	Rhodospirillales	Acetobacteraceae	Roseomonas	Roseomonas cervicalis
11	Bacteria	Proteobacteria	Alphaproteobacteria	Rhodobacterales	Rhodobacteraceae	Tateyamaria	Tateyamaria sp. ANG-S1
11	Bacteria	Proteobacteria	Alphaproteobacteria	Rhodobacterales	Rhodobacteraceae	Ruegeria	Ruegeria lacuscaerulensis
11	Bacteria	Proteobacteria	Alphaproteobacteria	Rhodobacterales	Rhodobacteraceae	Roseobacter	Roseobacter sp. GAI101
11	Bacteria	Proteobacteria	Alphaproteobacteria	Rhodobacterales	Rhodobacteraceae	Roseivivax	Roseivivax halodurans
11	Bacteria	Proteobacteria	Alphaproteobacteria	Rhodobacterales	Rhodobacteraceae	Rhodovulum	Rhodovulum sp. MB263
11	Bacteria	Proteobacteria	Alphaproteobacteria	Rhodobacterales	Rhodobacteraceae	Pseudopelagicola	Pseudopelagicola gijangensis
11	Bacteria	Proteobacteria	Alphaproteobacteria	Rhodobacterales	Rhodobacteraceae	Paracoccus	Paracoccus sp. PAMC 22219
11	Bacteria	Proteobacteria	Alphaproteobacteria	Rhodobacterales	Rhodobacteraceae	Paracoccus	Paracoccus alkenifer
11	Bacteria	Proteobacteria	Alphaproteobacteria	Rhodobacterales	Rhodobacteraceae	Jannaschia	Jannaschia rubra
11	Bacteria	Proteobacteria	Alphaproteobacteria	Rhodobacterales	Rhodobacteraceae	Gemmobacter	
11	Bacteria	Proteobacteria	Alphaproteobacteria	Rhodobacterales	Rhodobacteraceae	Celeribacter	
11	Bacteria	Proteobacteria	Alphaproteobacteria	Rhodobacterales	Hyphomonadaceae	Ponticaulis	Ponticaulis koreensis
11	Bacteria	Proteobacteria	Alphaproteobacteria	Rhodobacterales	Hyphomonadaceae	Hyphomonas	Hyphomonas sp. BRH_c22
11	Bacteria	Proteobacteria	Alphaproteobacteria	Rhodobacterales	Hyphomonadaceae	Euryhalocaulis	Euryhalocaulis caribicus
11	Bacteria	Proteobacteria	Alphaproteobacteria	Rhizobiales	Xanthobacteraceae	Azorhizobium	Azorhizobium doebereinerae
11	Bacteria	Proteobacteria	Alphaproteobacteria	Rhizobiales	Rhizobiaceae	Shinella	Shinella sp. HZN7
11	Bacteria	Proteobacteria	Alphaproteobacteria	Rhizobiales	Rhizobiaceae	Rhizobium/Agrobacterium group	Rhizobium
11	Bacteria	Proteobacteria	Alphaproteobacteria	Rhizobiales	Rhizobiaceae	Kaistia	Kaistia adipata
11	Bacteria	Proteobacteria	Alphaproteobacteria	Rhizobiales	Phyllobacteriaceae	Mesorhizobium	Mesorhizobium sp. STM 4661
11	Bacteria	Proteobacteria	Alphaproteobacteria	Rhizobiales	Phyllobacteriaceae	Mesorhizobium	Mesorhizobium sp. ORS3324
11	Bacteria	Proteobacteria	Alphaproteobacteria	Rhizobiales	Phyllobacteriaceae	Mesorhizobium	Mesorhizobium sp. 1M-11
11	Bacteria	Proteobacteria	Alphaproteobacteria	Rhizobiales	Phyllobacteriaceae	Mesorhizobium	Mesorhizobium prunaredense
11	Bacteria	Proteobacteria	Alphaproteobacteria	Rhizobiales	Methylocystaceae	Methylocystis	Methylocystis parvus
11	Bacteria	Proteobacteria	Alphaproteobacteria	Rhizobiales	Methylobacteriaceae	Microvirga	Microvirga sp. BSC39
11	Bacteria	Proteobacteria	Alphaproteobacteria	Rhizobiales	Methylobacteriaceae	Methylobacterium	Methylobacterium sp. CCH5-D2
11	Bacteria	Proteobacteria	Alphaproteobacteria	Rhizobiales	Methylobacteriaceae	Methylobacterium	Methylobacterium populi
11	Bacteria	Proteobacteria	Alphaproteobacteria	Rhizobiales	Hyphomicrobiaceae	Devosia	Devosia epidermidihirudinis
11	Bacteria	Proteobacteria	Alphaproteobacteria	Rhizobiales	Bradyrhizobiaceae	Bradyrhizobium	Bradyrhizobium icense
11	Bacteria	Proteobacteria	Alphaproteobacteria	Rhizobiales	Bradyrhizobiaceae	Bosea	Bosea sp. AAP35
11	Bacteria	Proteobacteria	Alphaproteobacteria	Rhizobiales	Beijerinckiaceae	Beijerinckia	Beijerinckia indica
11	Bacteria	Proteobacteria	Alphaproteobacteria	Rhizobiales	Aurantimonadaceae	Aureimonas	Aureimonas sp. AU12
11	Bacteria	Proteobacteria	Alphaproteobacteria	Rhizobiales	Aurantimonadaceae	Aurantimonas	Aurantimonas sp. Leaf443
11	Bacteria	Proteobacteria	Alphaproteobacteria	Rhizobiales	Aurantimonadaceae		
11	Bacteria	Proteobacteria	Alphaproteobacteria	Kordiimonadales	Kordiimonadaceae	Kordiimonas	Kordiimonas lacus
11	Bacteria	Proteobacteria	Alphaproteobacteria	Caulobacterales	Caulobacteraceae	Brevundimonas	Brevundimonas sp. Root1279
11	Bacteria	PVC group	Verrucomicrobia	unclassified Verrucomicrobia	unclassified Verrucomicrobia (miscellaneous)	Verrucomicrobia bacterium A1	
11	Bacteria	PVC group	Verrucomicrobia	Opitutae	Puniceicoccales	Puniceicoccaceae	Coraliomargarita
11	Bacteria	PVC group	Planctomycetes	unclassified Planctomycetes	Planctomycetes bacterium GWC2_45_44		
11	Bacteria	PVC group	Chlamydiae	Chlamydiia	Chlamydiales	Chlamydiaceae	Chlamydia/Chlamydophila group
11	Bacteria	PVC group	Candidatus Omnitrophica	unclassified Candidatus Omnitrophica	Omnitrophica bacterium OLB16		
11	Bacteria	Nitrospinae					
11	Bacteria	Nitrospinae/Tectomicrobia group	Candidatus Tectomicrobia	unclassified Candidatus Tectomicrobia			
11	Bacteria	Nitrospinae/Tectomicrobia group	Candidatus Tectomicrobia	Candidatus Entotheonella			
11	Bacteria	FCB group	Bacteroidetes/Chlorobi group	Bacteroidetes	unclassified Bacteroidetes	unclassified Bacteroidetes (miscellaneous)	Bacteroidetes bacterium OLB10
11	Bacteria	FCB group	Bacteroidetes/Chlorobi group	Bacteroidetes	Sphingobacteriia	Sphingobacteriales	Sphingobacteriaceae
11	Bacteria	FCB group	Bacteroidetes/Chlorobi group	Bacteroidetes	Flavobacteriia	Flavobacteriales	Cryomorphaceae
11	Bacteria	FCB group	Bacteroidetes/Chlorobi group	Bacteroidetes	Cytophagia	Cytophagales	Hymenobacteraceae
11	Bacteria	FCB group	Bacteroidetes/Chlorobi group	Bacteroidetes	Cytophagia	Cytophagales	Cytophagaceae
11	Bacteria	FCB group	Bacteroidetes/Chlorobi group	Bacteroidetes	Cytophagia	Cytophagales	Catalimonadaceae
11	Bacteria	FCB group	Bacteroidetes/Chlorobi group	Bacteroidetes	Chitinophagia	Chitinophagales	Chitinophagaceae
11	Bacteria	FCB group	Bacteroidetes/Chlorobi group	Bacteroidetes	Chitinophagia	Chitinophagales	Chitinophagaceae
11	Bacteria	FCB group	Bacteroidetes/Chlorobi group	Bacteroidetes	Chitinophagia	Chitinophagales	Chitinophagaceae
11	Bacteria	FCB group	Bacteroidetes/Chlorobi group	Bacteroidetes	Chitinophagia	Chitinophagales	Chitinophagaceae
11	Bacteria	FCB group	Bacteroidetes/Chlorobi group	Bacteroidetes	Chitinophagia	Chitinophagales	Chitinophagaceae
11	Bacteria	FCB group	Bacteroidetes/Chlorobi group	Bacteroidetes	Bacteroidia	Marinilabiliales	Marinilabiliaceae
11	Bacteria	FCB group	Bacteroidetes/Chlorobi group	Bacteroidetes	Bacteroidia	Marinilabiliales	Marinilabiliaceae
11	Bacteria	FCB group	Bacteroidetes/Chlorobi group	Bacteroidetes	Bacteroidia	Bacteroidales	unclassified Bacteroidales
11	Bacteria	FCB group	Bacteroidetes/Chlorobi group	Bacteroidetes	Bacteroidia	Bacteroidales	Prevotellaceae
11	Bacteria	FCB group	Bacteroidetes/Chlorobi group	Bacteroidetes	Bacteroidia	Bacteroidales	Porphyromonadaceae
11	Bacteria	FCB group	Bacteroidetes/Chlorobi group	Bacteroidetes	Bacteroidia	Bacteroidales	Bacteroidaceae
11	Bacteria	FCB group	Bacteroidetes/Chlorobi group	Bacteroidetes	Bacteroidetes Order II. Incertae sedis	Rhodothermaceae	unclassified Rhodothermaceae
11	Bacteria	Chrysiogenetes	Chrysiogenetes	Chrysiogenales	Chrysiogenaceae	Chrysiogenes	Chrysiogenes arsenatis
11	Bacteria	Acidobacteria	Acidobacteriia	Acidobacteriales	Acidobacteriaceae	Silvibacterium	Silvibacterium bohemicum
11	environmental samples	uncultured virus					
10	Bacteria	unclassified Bacteria	unclassified Bacteria (miscellaneous)	bacterium JKG1			
10	Bacteria	unclassified Bacteria	Bacteria candidate phyla	candidate division NC10	unclassified candidate division NC10		
10	Bacteria	unclassified Bacteria	Bacteria candidate phyla	Candidatus Edwardsbacteria			
10	Bacteria	Terrabacteria group	Firmicutes	Negativicutes	Veillonellales	Veillonellaceae	Megasphaera
10	Bacteria	Terrabacteria group	Firmicutes	Negativicutes	Selenomonadales	Sporomusaceae	Pelosinus
10	Bacteria	Terrabacteria group	Firmicutes	Clostridia	Thermoanaerobacterales	Thermoanaerobacteraceae	Moorella group
10	Bacteria	Terrabacteria group	Firmicutes	Clostridia	Clostridiales	unclassified Clostridiales	unclassified Clostridiales (miscellaneous)
10	Bacteria	Terrabacteria group	Firmicutes	Clostridia	Clostridiales	unclassified Clostridiales	Flavonifractor
10	Bacteria	Terrabacteria group	Firmicutes	Clostridia	Clostridiales	Ruminococcaceae	Ruminococcus
10	Bacteria	Terrabacteria group	Firmicutes	Clostridia	Clostridiales	Ruminococcaceae	Pseudobacteroides
10	Bacteria	Terrabacteria group	Firmicutes	Clostridia	Clostridiales	Lachnospiraceae	Tyzzerella
10	Bacteria	Terrabacteria group	Firmicutes	Clostridia	Clostridiales	Lachnospiraceae	Lachnoclostridium
10	Bacteria	Terrabacteria group	Firmicutes	Clostridia	Clostridiales	Lachnospiraceae	Anaerobium
10	Bacteria	Terrabacteria group	Firmicutes	Clostridia	Clostridiales	Eubacteriaceae	Eubacterium
10	Bacteria	Terrabacteria group	Firmicutes	Clostridia	Clostridiales	Clostridiaceae	Natronincola
10	Bacteria	Terrabacteria group	Firmicutes	Clostridia	Clostridiales	Clostridiaceae	Clostridium
10	Bacteria	Terrabacteria group	Firmicutes	Bacilli	Bacillales	Alicyclobacillaceae	Alicyclobacillus
10	Bacteria	Terrabacteria group	Cyanobacteria/Melainabacteria group	Cyanobacteria	Synechococcales	Leptolyngbyaceae	Phormidesmis
10	Bacteria	Terrabacteria group	Chloroflexi				
10	Bacteria	Terrabacteria group	Armatimonadetes	unclassified Armatimonadetes	Armatimonadetes bacterium CG2_30_59_28		
10	Bacteria	Terrabacteria group	Actinobacteria	Thermoleophilia	Solirubrobacterales	Patulibacteraceae	Patulibacter
10	Bacteria	Terrabacteria group	Actinobacteria	Actinobacteria	Streptosporangiales	Nocardiopsaceae	Nocardiopsis
10	Bacteria	Terrabacteria group	Actinobacteria	Actinobacteria	Propionibacteriales	Nocardioidaceae	Nocardioides
10	Bacteria	Terrabacteria group	Actinobacteria	Actinobacteria	Micromonosporales	Micromonosporaceae	Micromonospora
10	Bacteria	Synergistetes	Synergistia	Synergistales	Synergistaceae	Cloacibacillus	Cloacibacillus porcorum
10	Bacteria	Spirochaetes	Spirochaetia	Spirochaetales	Spirochaetaceae	Candidatus Marispirochaeta	Candidatus Marispirochaeta sp. JC444
10	Bacteria	Proteobacteria	delta/epsilon subdivisions	Deltaproteobacteria	Syntrophobacterales	Syntrophobacteraceae	Thermodesulforhabdus
10	Bacteria	Proteobacteria	delta/epsilon subdivisions	Deltaproteobacteria	Syntrophobacterales	Syntrophaceae	Syntrophus
10	Bacteria	Proteobacteria	delta/epsilon subdivisions	Deltaproteobacteria	Myxococcales	Sorangiineae	
10	Bacteria	Proteobacteria	delta/epsilon subdivisions	Deltaproteobacteria	Myxococcales	Cystobacterineae	Myxococcaceae
10	Bacteria	Proteobacteria	delta/epsilon subdivisions	Deltaproteobacteria	Desulfuromonadales	Geobacteraceae	Geothermobacter
10	Bacteria	Proteobacteria	delta/epsilon subdivisions	Deltaproteobacteria	Desulfuromonadales	Geobacteraceae	Geobacter
10	Bacteria	Proteobacteria	delta/epsilon subdivisions	Deltaproteobacteria	Desulfuromonadales	Geobacteraceae	Geobacter
10	Bacteria	Proteobacteria	delta/epsilon subdivisions	Deltaproteobacteria	Desulfuromonadales	Desulfuromonadaceae	Desulfuromonas
10	Bacteria	Proteobacteria	delta/epsilon subdivisions	Deltaproteobacteria	Desulfovibrionales	Desulfovibrionaceae	Halodesulfovibrio

10	Bacteria	Proteobacteria	delta/epsilon subdivisions	Deltaproteobacteria	Desulfovibrionales	Desulfohalobiaceae	Desulfonauticus	
10	Bacteria	Proteobacteria	delta/epsilon subdivisions	Deltaproteobacteria	Desulfobacterales	unclassified Desulfobacterales	Desulfobacterales bacterium CG2_30_60_27	
10	Bacteria	Proteobacteria	delta/epsilon subdivisions	Deltaproteobacteria	Desulfobacterales	Desulfobulbaceae	Desulfobulbus	
10	Bacteria	Proteobacteria	delta/epsilon subdivisions	Deltaproteobacteria	Desulfobacterales	Desulfobacteraceae	Desulfobacterium	
10	Bacteria	Proteobacteria	delta/epsilon subdivisions	Deltaproteobacteria	Desulfobacterales	Desulfobacteraceae	Desulfatiglans	
10	Bacteria	Proteobacteria	Gammaproteobacteria	unclassified Gammaproteobacteria	unclassified Gammaproteobacteria (miscellaneous)	Gammaproteobacteria bacterium BRH_c0		
10	Bacteria	Proteobacteria	Gammaproteobacteria	unclassified Gammaproteobacteria	Sedimenticola	Sedimenticola selenatireducens		
10	Bacteria	Proteobacteria	Gammaproteobacteria	Xanthomonadales	Xanthomonadaceae	Xanthomonas	Xanthomonas translucens	
10	Bacteria	Proteobacteria	Gammaproteobacteria	Xanthomonadales	Xanthomonadaceae	Xanthomonas	Xanthomonas campestris	
10	Bacteria	Proteobacteria	Gammaproteobacteria	Xanthomonadales	Xanthomonadaceae	Lysobacter	Lysobacter arseniciresistens	
10	Bacteria	Proteobacteria	Gammaproteobacteria	Xanthomonadales	Xanthomonadaceae	Luteimonas	Luteimonas sp. JM171	
10	Bacteria	Proteobacteria	Gammaproteobacteria	Xanthomonadales	Xanthomonadaceae	Luteimonas	Luteimonas huabeiensis	
10	Bacteria	Proteobacteria	Gammaproteobacteria	Vibrionales	Vibrionaceae	Photobacterium		
10	Bacteria	Proteobacteria	Gammaproteobacteria	Thiotrichales	unclassified Thiotrichales	unclassified Thiotrichales (miscellaneous)	Thiotrichales bacterium SG8_50	
10	Bacteria	Proteobacteria	Gammaproteobacteria	Thiotrichales	Thiotrichaceae	Thiothrix	Thiothrix caldifontis	
10	Bacteria	Proteobacteria	Gammaproteobacteria	Pseudomonadales	Ventosimonadaceae	Ventosimonas	Ventosimonas gracilis	
10	Bacteria	Proteobacteria	Gammaproteobacteria	Pseudomonadales	Moraxellaceae	Moraxella		
10	Bacteria	Proteobacteria	Gammaproteobacteria	Oceanospirillales	Hahellaceae	Endozoicomonas	Endozoicomonas arenosclerae	
10	Bacteria	Proteobacteria	Gammaproteobacteria	Nevskiales	Algiphilaceae	Algiphilus	Algiphilus aromaticivorans	
10	Bacteria	Proteobacteria	Gammaproteobacteria	Methylococcales	Methylococcaceae	Methyloglobulus	Methyloglobulus morosus	
10	Bacteria	Proteobacteria	Gammaproteobacteria	Methylococcales	Methylococcaceae	Methylocaldum	Methylocaldum sp. 14B	
10	Bacteria	Proteobacteria	Gammaproteobacteria	Methylococcales	Methylococcaceae			
10	Bacteria	Proteobacteria	Gammaproteobacteria	Enterobacterales	Yersiniaceae	Serratia	Serratia marcescens	
10	Bacteria	Proteobacteria	Gammaproteobacteria	Enterobacterales	Morganellaceae	Xenorhabdus		
10	Bacteria	Proteobacteria	Gammaproteobacteria	Chromatiales	Halothiobacillaceae	Halothiobacillus	Halothiobacillus sp. LS2	
10	Bacteria	Proteobacteria	Gammaproteobacteria	Chromatiales	Ectothiorhodospiraceae	Arhodomonas	Arhodomonas aquaeolei	
10	Bacteria	Proteobacteria	Gammaproteobacteria	Cellvibrionales	Cellvibrionaceae	Cellvibrio	Cellvibrio mixtus	
10	Bacteria	Proteobacteria	Candidatus Muproteobacteria	Candidatus Muproteobacteria bacterium RBG_16_62_13				
10	Bacteria	Proteobacteria	Betaproteobacteria	Sulfuricellales	Sulfuricellaceae	Sulfuricella		
10	Bacteria	Proteobacteria	Betaproteobacteria	Rhodocyclales	Rhodocyclaceae	Azoarcus	Azoarcus tolulyticus	
10	Bacteria	Proteobacteria	Betaproteobacteria	Rhodocyclales	Rhodocyclaceae	Aromatoleum	Aromatoleum aromaticum	
10	Bacteria	Proteobacteria	Betaproteobacteria	Nitrosomonadales	Nitrosomonadaceae	Nitrosospira	Nitrosospira sp. Nsp14	
10	Bacteria	Proteobacteria	Betaproteobacteria	Nitrosomonadales	Nitrosomonadaceae	Nitrosomonas	Nitrosomonas sp. Nm166	
10	Bacteria	Proteobacteria	Betaproteobacteria	Neisseriales	Chromobacteriaceae	Jeongeupia	Jeongeupia sp. USM3	
10	Bacteria	Proteobacteria	Betaproteobacteria	Neisseriales	Chromobacteriaceae	Chromobacterium group	Chromobacterium	
10	Bacteria	Proteobacteria	Betaproteobacteria	Methylophilales	Methylophilaceae	Methylophilus	Methylophilus sp. 5	
10	Bacteria	Proteobacteria	Betaproteobacteria	Methylophilales				
10	Bacteria	Proteobacteria	Betaproteobacteria	Hydrogenophilales	Hydrogenophilaceae	Thiobacillus	Thiobacillus sp. 0-1251	
10	Bacteria	Proteobacteria	Betaproteobacteria	Gallionellales	unclassified Gallionellales	Gallionellales bacterium RBG_16_57_15		
10	Bacteria	Proteobacteria	Betaproteobacteria	Burkholderiales	unclassified Burkholderiales	unclassified Burkholderiales (miscellaneous)	Burkholderiales bacterium RIFCSPHIGHO2_02_FULL_66_10	
10	Bacteria	Proteobacteria	Betaproteobacteria	Burkholderiales	Oxalobacteraceae	Herminiimonas	Herminiimonas arsenicoxydans	
10	Bacteria	Proteobacteria	Betaproteobacteria	Burkholderiales	Oxalobacteraceae	Collimonas	Collimonas sp. OK607	
10	Bacteria	Proteobacteria	Betaproteobacteria	Burkholderiales	Oxalobacteraceae	Collimonas	Collimonas pratensis	
10	Bacteria	Proteobacteria	Betaproteobacteria	Burkholderiales	Oxalobacteraceae	Collimonas	Collimonas fungivorans	
10	Bacteria	Proteobacteria	Betaproteobacteria	Burkholderiales	Comamonadaceae	Variovorax	Variovorax sp. Root473	
10	Bacteria	Proteobacteria	Betaproteobacteria	Burkholderiales	Comamonadaceae	Acidovorax	Acidovorax sp. Root70	
10	Bacteria	Proteobacteria	Betaproteobacteria	Burkholderiales	Comamonadaceae	Acidovorax	Acidovorax avenae	
10	Bacteria	Proteobacteria	Betaproteobacteria	Burkholderiales	Burkholderiaceae	Polynucleobacter	Polynucleobacter sinensis	
10	Bacteria	Proteobacteria	Betaproteobacteria	Burkholderiales	Alcaligenaceae	Bordetella	Bordetella sp. N	
10	Bacteria	Proteobacteria	Betaproteobacteria	Burkholderiales	Alcaligenaceae	Bordetella	Bordetella sp. H567	
10	Bacteria	Proteobacteria	Betaproteobacteria	Burkholderiales	Alcaligenaceae	Advenella	Advenella mimigardefordensis	
10	Bacteria	Proteobacteria	Alphaproteobacteria	unclassified Alphaproteobacteria	unclassified Alphaproteobacteria (miscellaneous)	Alphaproteobacteria bacterium GWF2_58_20		
10	Bacteria	Proteobacteria	Alphaproteobacteria	unclassified Alphaproteobacteria	unclassified Alphaproteobacteria (miscellaneous)	Alphaproteobacteria bacterium 64-11		
10	Bacteria	Proteobacteria	Alphaproteobacteria	unclassified Alphaproteobacteria	unclassified Alphaproteobacteria (miscellaneous)	Alphaproteobacteria bacterium 33-17		
10	Bacteria	Proteobacteria	Alphaproteobacteria	Sphingomonadales	unclassified Sphingomonadales	Sphingomonadales bacterium GWF1_63_6		
10	Bacteria	Proteobacteria	Alphaproteobacteria	Sphingomonadales	Sphingomonadaceae	Sphingobium	Sphingobium sp. MI1205	
10	Bacteria	Proteobacteria	Alphaproteobacteria	Sphingomonadales	Sphingomonadaceae	Novosphingobium	Novosphingobium sp. 63-713	
10	Bacteria	Proteobacteria	Alphaproteobacteria	Rickettsiales	Anaplasmataceae	Wolbachieae	Wolbachia	
10	Bacteria	Proteobacteria	Alphaproteobacteria	Rickettsiales	Anaplasmataceae	Wolbachieae	Wolbachia	
10	Bacteria	Proteobacteria	Alphaproteobacteria	Rhodospirillales	Rhodospirillaceae	Pararhodospirillum	Pararhodospirillum photometricum	
10	Bacteria	Proteobacteria	Alphaproteobacteria	Rhodospirillales	Acetobacteraceae	Acidocella	Acidocella aminolytica	
10	Bacteria	Proteobacteria	Alphaproteobacteria	Rhodobacterales	Rhodobacteraceae	unclassified Rhodobacteraceae	Rhodobacteraceae bacterium HLUCCA09	
10	Bacteria	Proteobacteria	Alphaproteobacteria	Rhodobacterales	Rhodobacteraceae	Sulfitobacter	Sulfitobacter sp. AM1-D1	
10	Bacteria	Proteobacteria	Alphaproteobacteria	Rhodobacterales	Rhodobacteraceae	Sulfitobacter	Sulfitobacter pseudonitzschiae	
10	Bacteria	Proteobacteria	Alphaproteobacteria	Rhodobacterales	Rhodobacteraceae	Sulfitobacter	Sulfitobacter noctilucae	
10	Bacteria	Proteobacteria	Alphaproteobacteria	Rhodobacterales	Rhodobacteraceae	Sulfitobacter	Sulfitobacter marinus	
10	Bacteria	Proteobacteria	Alphaproteobacteria	Rhodobacterales	Rhodobacteraceae	Sulfitobacter	Sulfitobacter donghicola	
10	Bacteria	Proteobacteria	Alphaproteobacteria	Rhodobacterales	Rhodobacteraceae	Stappia	Stappia sp. ES.058	
10	Bacteria	Proteobacteria	Alphaproteobacteria	Rhodobacterales	Rhodobacteraceae	Ruegeria	Ruegeria sp. CECT 5091	
10	Bacteria	Proteobacteria	Alphaproteobacteria	Rhodobacterales	Rhodobacteraceae	Ruegeria	Ruegeria conchae	
10	Bacteria	Proteobacteria	Alphaproteobacteria	Rhodobacterales	Rhodobacteraceae	Roseovarius	Roseovarius gaetbuli	
10	Bacteria	Proteobacteria	Alphaproteobacteria	Rhodobacterales	Rhodobacteraceae	Pseudovibrio	Pseudovibrio axinellae	
10	Bacteria	Proteobacteria	Alphaproteobacteria	Rhodobacterales	Rhodobacteraceae	Phaeobacter		
10	Bacteria	Proteobacteria	Alphaproteobacteria	Rhodobacterales	Rhodobacteraceae	Paracoccus	Paracoccus sp. SCN 68-21	
10	Bacteria	Proteobacteria	Alphaproteobacteria	Rhodobacterales	Rhodobacteraceae	Paracoccus	Paracoccus sp. N5	
10	Bacteria	Proteobacteria	Alphaproteobacteria	Rhodobacterales	Rhodobacteraceae	Paracoccus	Paracoccus solventivorans	
10	Bacteria	Proteobacteria	Alphaproteobacteria	Rhodobacterales	Rhodobacteraceae	Octadecabacter	Octadecabacter arcticus	
10	Bacteria	Proteobacteria	Alphaproteobacteria	Rhodobacterales	Hyphomonadaceae	Hirschia	Hirschia baltica	
10	Bacteria	Proteobacteria	Alphaproteobacteria	Rhizobiales	unclassified Rhizobiales	unclassified Rhizobiales (miscellaneous)	Rhizobiales bacterium 63-22	
10	Bacteria	Proteobacteria	Alphaproteobacteria	Rhizobiales	Rhizobiaceae	Sinorhizobium/Ensifer group	Sinorhizobium	
10	Bacteria	Proteobacteria	Alphaproteobacteria	Rhizobiales	Rhizobiaceae	Shinella	Shinella sp. 65-6	
10	Bacteria	Proteobacteria	Alphaproteobacteria	Rhizobiales	Rhizobiaceae	Rhizobium/Agrobacterium group	Rhizobium	
10	Bacteria	Proteobacteria	Alphaproteobacteria	Rhizobiales	Methylocystaceae	Methylocystis	Methylocystis sp. ATCC 49242	
10	Bacteria	Proteobacteria	Alphaproteobacteria	Rhizobiales	Methylobacteriaceae	Methylobacterium	Methylobacterium nodulans	
10	Bacteria	Proteobacteria	Alphaproteobacteria	Rhizobiales	Methylobacteriaceae	Methylobacterium	Methylobacterium aquaticum	
10	Bacteria	Proteobacteria	Alphaproteobacteria	Rhizobiales	Bradyrhizobiaceae	Rhodopseudomonas	Rhodopseudomonas sp. AAP120	
10	Bacteria	Proteobacteria	Alphaproteobacteria	Rhizobiales	Bradyrhizobiaceae	Bradyrhizobium	Bradyrhizobium sp. ORS 375	
10	Bacteria	Proteobacteria	Alphaproteobacteria	Rhizobiales	Bradyrhizobiaceae	Bradyrhizobium	Bradyrhizobium sp. DFCI-1	
10	Bacteria	Proteobacteria	Alphaproteobacteria	Rhizobiales	Bradyrhizobiaceae	Bradyrhizobium	Bradyrhizobium canariense	
[illegible]								

8	Bacteria	Proteobacteria	Gammaproteobacteria	Alteromonadales	Alteromonadaceae	Marinobacter	Marinobacter nanhaiticus
8	Bacteria	Proteobacteria	Gammaproteobacteria	Alteromonadales	Alteromonadaceae	Marinobacter	Marinobacter lipolyticus
8	Bacteria	Proteobacteria	Gammaproteobacteria	Aeromonadales	Succinivibrionaceae	Succinivibrio	Succinivibrio dextrinosolvens
8	Bacteria	Proteobacteria	Gammaproteobacteria	Aeromonadales	Aeromonadaceae	Aeromonas	Aeromonas rivuli
8	Bacteria	Proteobacteria	Gammaproteobacteria	Aeromonadales	Aeromonadaceae	Aeromonas	Aeromonas dhakensis
8	Bacteria	Proteobacteria	Betaproteobacteria	unclassified Betaproteobacteria	unclassified Betaproteobacteria (miscellaneous)	Betaproteobacteria bacterium RIFCSPLOWO2_02_FULL_64_12	
8	Bacteria	Proteobacteria	Betaproteobacteria	Rhodocyclales	Rhodocyclaceae	Thauera	Thauera terpenica
8	Bacteria	Proteobacteria	Betaproteobacteria	Rhodocyclales	Rhodocyclaceae	Thauera	Thauera butanivorans
8	Bacteria	Proteobacteria	Betaproteobacteria	Nitrosomonadales	Nitrosomonadaceae	Nitrosovibrio	Nitrosovibrio sp. Nv6
8	Bacteria	Proteobacteria	Betaproteobacteria	Nitrosomonadales	Nitrosomonadaceae	Nitrosospira	Nitrosospira sp. NpAV
8	Bacteria	Proteobacteria	Betaproteobacteria	Nitrosomonadales	Nitrosomonadaceae	Nitrosospira	Nitrosospira sp. 56-18
8	Bacteria	Proteobacteria	Betaproteobacteria	Nitrosomonadales	Nitrosomonadaceae	Nitrosomonas	Nitrosomonas sp. Nm33
8	Bacteria	Proteobacteria	Betaproteobacteria	Nitrosomonadales	Nitrosomonadaceae	Nitrosomonas	Nitrosomonas marina
8	Bacteria	Proteobacteria	Betaproteobacteria	Neisseriales	Neisseriaceae	Simonsiella	Simonsiella muelleri
8	Bacteria	Proteobacteria	Betaproteobacteria	Neisseriales	Neisseriaceae	Neisseria	Neisseria dentiae
8	Bacteria	Proteobacteria	Betaproteobacteria	Neisseriales	Chromobacteriaceae	Vogesella	Vogesella sp. EB
8	Bacteria	Proteobacteria	Betaproteobacteria	Methylophilales	Methylophilaceae	Methylotenera	Methylotenera sp. N17
8	Bacteria	Proteobacteria	Betaproteobacteria	Burkholderiales	unclassified Burkholderiales	Burkholderiales Genera incertae sedis	Aquabacterium
8	Bacteria	Proteobacteria	Betaproteobacteria	Burkholderiales	Oxalobacteraceae	Massilia	Massilia putida
8	Bacteria	Proteobacteria	Betaproteobacteria	Burkholderiales	Comamonadaceae	Variovorax	Variovorax sp. Root318D1
8	Bacteria	Proteobacteria	Betaproteobacteria	Burkholderiales	Comamonadaceae	Hydrogenophaga	Hydrogenophaga intermedia
8	Bacteria	Proteobacteria	Betaproteobacteria	Burkholderiales	Comamonadaceae	Caldimonas	Caldimonas taiwanensis
8	Bacteria	Proteobacteria	Betaproteobacteria	Burkholderiales	Burkholderiaceae	Ralstonia	Ralstonia mannitolilytica
8	Bacteria	Proteobacteria	Betaproteobacteria	Burkholderiales	Burkholderiaceae	Polynucleobacter	Polynucleobacter sp. MWH-Weng1-1
8	Bacteria	Proteobacteria	Betaproteobacteria	Burkholderiales	Burkholderiaceae	Paraburkholderia	Paraburkholderia mimosarum
8	Bacteria	Proteobacteria	Betaproteobacteria	Burkholderiales	Burkholderiaceae	Pandoraea	Pandoraea pnomenusa
8	Bacteria	Proteobacteria	Betaproteobacteria	Burkholderiales	Burkholderiaceae	Cupriavidus	Cupriavidus taiwanensis
8	Bacteria	Proteobacteria	Betaproteobacteria	Burkholderiales	Burkholderiaceae	Cupriavidus	Cupriavidus gilardii
8	Bacteria	Proteobacteria	Betaproteobacteria	Burkholderiales	Burkholderiaceae	Caballeronia	
8	Bacteria	Proteobacteria	Betaproteobacteria	Burkholderiales	Burkholderiaceae	Burkholderia	Burkholderia calidae
8	Bacteria	Proteobacteria	Betaproteobacteria	Burkholderiales	Burkholderiaceae	Burkholderia	Burkholderia arvi
8	Bacteria	Proteobacteria	Betaproteobacteria	Burkholderiales	Alcaligenaceae	Taylorella	Taylorella equigenitalis
8	Bacteria	Proteobacteria	Betaproteobacteria	Burkholderiales	Alcaligenaceae	Castellaniella	Castellaniella caeni
8	Bacteria	Proteobacteria	Betaproteobacteria	Burkholderiales	Alcaligenaceae	Bordetella	Bordetella genomosp. 13
8	Bacteria	Proteobacteria	Betaproteobacteria	Burkholderiales	Alcaligenaceae	Advenella	
8	Bacteria	Proteobacteria	Alphaproteobacteria	unclassified Alphaproteobacteria	unclassified Alphaproteobacteria (miscellaneous)	alpha proteobacterium IMCC14465	
8	Bacteria	Proteobacteria	Alphaproteobacteria	Sphingomonadales	Sphingomonadaceae	Sphingorhabdus	
8	Bacteria	Proteobacteria	Alphaproteobacteria	Sphingomonadales	Sphingomonadaceae	Sphingomonas	Sphingomonas sp. MEA3-1
8	Bacteria	Proteobacteria	Alphaproteobacteria	Sphingomonadales	Sphingomonadaceae	Sphingomonas	Sphingomonas sp. Leaf231
8	Bacteria	Proteobacteria	Alphaproteobacteria	Sphingomonadales	Sphingomonadaceae	Sphingomonas	Sphingomonas sp. CCH9-F2
8	Bacteria	Proteobacteria	Alphaproteobacteria	Sphingomonadales	Sphingomonadaceae	Sphingobium	Sphingobium sp. ba1
8	Bacteria	Proteobacteria	Alphaproteobacteria	Sphingomonadales	Sphingomonadaceae	Novosphingobium	Novosphingobium sp. AAP1
8	Bacteria	Proteobacteria	Alphaproteobacteria	Rickettsiales	Anaplasmataceae	Ehrlichia	canis group
8	Bacteria	Proteobacteria	Alphaproteobacteria	Rickettsiales	Anaplasmataceae	Ehrlichia	canis group
8	Bacteria	Proteobacteria	Alphaproteobacteria	Rhodospirillales	Rhodospirillaceae	Skermanella	
8	Bacteria	Proteobacteria	Alphaproteobacteria	Rhodospirillales	Rhodospirillaceae	Rhodospira	Rhodospira trueperi
8	Bacteria	Proteobacteria	Alphaproteobacteria	Rhodospirillales	Rhodospirillaceae	Magnetospirillum	Magnetospirillum marisnigri
8	Bacteria	Proteobacteria	Alphaproteobacteria	Rhodospirillales	Acetobacteraceae	Acidocella	
8	Bacteria	Proteobacteria	Alphaproteobacteria	Rhodospirillales	Acetobacteraceae	Acidiphilium	Acidiphilium sp. PM
8	Bacteria	Proteobacteria	Alphaproteobacteria	Rhodobacterales	Rhodobacteraceae	Yangia	
8	Bacteria	Proteobacteria	Alphaproteobacteria	Rhodobacterales	Rhodobacteraceae	Sulfitobacter	Sulfitobacter delicatus
8	Bacteria	Proteobacteria	Alphaproteobacteria	Rhodobacterales	Rhodobacteraceae	Shimia	Shimia sp. SK013
8	Bacteria	Proteobacteria	Alphaproteobacteria	Rhodobacterales	Rhodobacteraceae	Ruegeria	Ruegeria mobilis
8	Bacteria	Proteobacteria	Alphaproteobacteria	Rhodobacterales	Rhodobacteraceae	Roseovarius	Roseovarius sp. 217
8	Bacteria	Proteobacteria	Alphaproteobacteria	Rhodobacterales	Rhodobacteraceae	Roseovarius	Roseovarius marisflavi
8	Bacteria	Proteobacteria	Alphaproteobacteria	Rhodobacterales	Rhodobacteraceae	Roseobacter	Roseobacter sp. SK209-2-6
8	Bacteria	Proteobacteria	Alphaproteobacteria	Rhodobacterales	Rhodobacteraceae	Phaeobacter	Phaeobacter sp. P97
8	Bacteria	Proteobacteria	Alphaproteobacteria	Rhodobacterales	Rhodobacteraceae	Paracoccus	Paracoccus sp. S4493
8	Bacteria	Proteobacteria	Alphaproteobacteria	Rhodobacterales	Rhodobacteraceae	Paenirhodobacter	Paenirhodobacter sp. MME-103
8	Bacteria	Proteobacteria	Alphaproteobacteria	Rhodobacterales	Rhodobacteraceae	Loktanella	Loktanella hongkongensis
8	Bacteria	Proteobacteria	Alphaproteobacteria	Rhodobacterales	Hyphomonadaceae	Robiginitomaculum	Robiginitomaculum antarcticum
8	Bacteria	Proteobacteria	Alphaproteobacteria	Rhodobacterales	Hyphomonadaceae	Hyphomonas	Hyphomonas atlantica
8	Bacteria	Proteobacteria	Alphaproteobacteria	Rhizobiales	Xanthobacteraceae	Ancylobacter	Ancylobacter sp. FA202
8	Bacteria	Proteobacteria	Alphaproteobacteria	Rhizobiales	Xanthobacteraceae	Ancylobacter	
8	Bacteria	Proteobacteria	Alphaproteobacteria	Rhizobiales	Rhizobiaceae	Rhizobium/Agrobacterium group	Rhizobium
8	Bacteria	Proteobacteria	Alphaproteobacteria	Rhizobiales	Rhizobiaceae	Rhizobium/Agrobacterium group	Rhizobium
8	Bacteria	Proteobacteria	Alphaproteobacteria	Rhizobiales	Rhizobiaceae	Rhizobium/Agrobacterium group	Rhizobium
8	Bacteria	Proteobacteria	Alphaproteobacteria	Rhizobiales	Rhizobiaceae	Rhizobium/Agrobacterium group	Pararhizobium
8	Bacteria	Proteobacteria	Alphaproteobacteria	Rhizobiales	Phyllobacteriaceae	Mesorhizobium	Mesorhizobium sp. Root157
8	Bacteria	Proteobacteria	Alphaproteobacteria	Rhizobiales	Methylocystaceae	Terasakiella	Terasakiella pusilla
8	Bacteria	Proteobacteria	Alphaproteobacteria	Rhizobiales	Methylocystaceae	Methylocystis	Methylocystis sp. SC2
8	Bacteria	Proteobacteria	Alphaproteobacteria	Rhizobiales	Methylobacteriaceae	Methylobacterium	Methylobacterium sp. ZNC0032
8	Bacteria	Proteobacteria	Alphaproteobacteria	Rhizobiales	Hyphomicrobiaceae	Devosia	Devosia sp. 537
8	Bacteria	Proteobacteria	Alphaproteobacteria	Rhizobiales	Hyphomicrobiaceae	Devosia	Devosia soli
8	Bacteria	Proteobacteria	Alphaproteobacteria	Rhizobiales	Hyphomicrobiaceae	Devosia	Devosia riboflavina
8	Bacteria	Proteobacteria	Alphaproteobacteria	Rhizobiales	Hyphomicrobiaceae	Devosia	Devosia insulae
8	Bacteria	Proteobacteria	Alphaproteobacteria	Rhizobiales	Bradyrhizobiaceae	Oligotropha	Oligotropha carboxidovorans
8	Bacteria	Proteobacteria	Alphaproteobacteria	Rhizobiales	Bradyrhizobiaceae	Nitrobacter	Nitrobacter hamburgensis
8	Bacteria	Proteobacteria	Alphaproteobacteria	Rhizobiales	Bradyrhizobiaceae	Nitrobacter	
8	Bacteria	Proteobacteria	Alphaproteobacteria	Rhizobiales	Bradyrhizobiaceae	Bradyrhizobium	Bradyrhizobium sp. STM 3843
8	Bacteria	Proteobacteria	Alphaproteobacteria	Rhizobiales	Bradyrhizobiaceae	Bradyrhizobium	Bradyrhizobium sp. NAS80.1
8	Bacteria	Proteobacteria	Alphaproteobacteria	Rhizobiales	Bradyrhizobiaceae	Bradyrhizobium	Bradyrhizobium sp. BR 10303
8	Bacteria	Proteobacteria	Alphaproteobacteria	Rhizobiales	Bradyrhizobiaceae	Bosea	Bosea thiooxidans
8	Bacteria	Proteobacteria	Alphaproteobacteria	Rhizobiales	Bradyrhizobiaceae	Bosea	Bosea sp. PAMC 26642
8	Bacteria	Proteobacteria	Alphaproteobacteria	Rhizobiales	Bradyrhizobiaceae	Bosea	Bosea sp. OK403
8	Bacteria	Proteobacteria	Alphaproteobacteria	Rhizobiales	Bradyrhizobiaceae	Bosea	Bosea sp. BIWAKO-01
8	Bacteria	Proteobacteria	Alphaproteobacteria	Rhizobiales	Bradyrhizobiaceae	Bosea	Bosea lupini
8	Bacteria	Proteobacteria	Alphaproteobacteria	Kordiimonadales	Kordiimonadaceae	Kordiimonas	Kordiimonas lipolytica
8	Bacteria	Proteobacteria	Alphaproteobacteria	Holosporales	unclassified Holosporales	Candidatus Nucleicultrix	Candidatus Nucleicultrix amoebiphila
8	Bacteria	Proteobacteria	Alphaproteobacteria	Caulobacterales	Caulobacteraceae	Phenylobacterium	Phenylobacterium sp. SCN 69-14
8	Bacteria	Proteobacteria	Alphaproteobacteria	Caulobacterales	Caulobacteraceae	Brevundimonas	Brevundimonas sp. LM2
8	Bacteria	Proteobacteria	Alphaproteobacteria	Caulobacterales	Caulobacteraceae	Brevundimonas	Brevundimonas sp. AAP58
8	Bacteria	Proteobacteria	Alphaproteobacteria	Caulobacterales	Caulobacteraceae	Brevundimonas	Brevundimonas aveniformis
8	Bacteria	Proteobacteria	Alphaproteobacteria	Caulobacterales	Caulobacteraceae	Asticcacaulis	Asticcacaulis biprosthecium
8	Bacteria	Proteobacteria	Alphaproteobacteria	Caulobacterales	Caulobacteraceae	Asticcacaulis	Asticcacaulis benevestitus
8	Bacteria	PVC group	Verrucomicrobia	Verrucomicrobiae	Verrucomicrobiales	Verrucomicrobiaceae	unclassified Verrucomicrobiaceae
8	Bacteria	PVC group	Verrucomicrobia	Opitutae	Opitutales	Opitutaceae	unclassified Opitutaceae
8	Bacteria	PVC group	Planctomycetes	unclassified Planctomycetes	Planctomycetes bacterium GWF2_50_10		
8	Bacteria	PVC group	Planctomycetes	Planctomycetia	Planctomycetales	Planctomycetaceae	Planctopirus
8	Bacteria	PVC group	Chlamydiae	unclassified Chlamydiae			
8	Bacteria	PVC group	Candidatus Omnitrophica	unclassified Candidatus Omnitrophica	Omnitrophica bacterium GWA2_41_15		
8	Bacteria	Nitrospira	Nitrospira	unclassified Nitrospira	Nitrospira bacterium SG8_3		
8	Bacteria	Fusobacteria	Fusobacteriia	Fusobacteriales	Fusobacteriaceae	Fusobacterium	environmental samples
8	Bacteria	FCB group	candidate division Zixibacteria	candidate division Zixibacteria bacterium SM23_81			
8	Bacteria	FCB group	Candidatus Latescibacteria	unclassified Candidatus Latescibacteria	Candidatus Latescibacteria bacterium 4484_107		
8	Bacteria	FCB group	Candidatus Cloacimonetes	Candidatus Cloacimonas	Candidatus Cloacimonas acidaminovorans		
8	Bacteria	FCB group	Bacteroidetes/Chlorobi group	Ignavibacteriae	Ignavibacteria	unclassified Ignavibacteria	Ignavibacteria bacterium CG2_30_36_16
8	Bacteria	FCB group	Bacteroidetes/Chlorobi group	Ignavibacteriae	Ignavibacteria		
8	Bacteria	FCB group	Bacteroidetes/Chlorobi group	Bacteroidetes	unclassified Bacteroidetes	unclassified Bacteroidetes (miscellaneous)	Bacteroidetes bacterium OLB8
8	Bacteria	FCB group	Bacteroidetes/Chlorobi group	Bacteroidetes	unclassified Bacteroidetes	unclassified Bacteroidetes (miscellaneous)	Bacteroidetes bacterium OLB12
8	Bacteria	FCB group	Bacteroidetes/Chlorobi group	Bacteroidetes	unclassified Bacteroidetes	unclassified Bacteroidetes (miscellaneous)	Bacteroidetes bacterium GWE2_40_15
8	Bacteria	FCB group	Bacteroidetes/Chlorobi group	Bacteroidetes	Sphingobacteriia	Sphingobacteriales	unclassified Sphingobacteriales
8	Bacteria	FCB group	Bacteroidetes/Chlorobi group	Bacteroidetes	Sphingobacteriia	Sphingobacteriales	Sphingobacteriaceae
8	Bacteria	FCB group	Bacteroidetes/Chlorobi group	Bacteroidetes	Flavobacteriia	Flavobacteriales	Flavobacteriaceae
8	Bacteria	FCB group	Bacteroidetes/Chlorobi group	Bacteroidetes	Flavobacteriia	Flavobacteriales	Flavobacteriaceae
8	Bacteria	FCB group	Bacteroidetes/Chlorobi group	Bacteroidetes	Flavobacteriia	Flavobacteriales	Flavobacteriaceae
8	Bacteria	FCB group	Bacteroidetes/Chlorobi group	Bacteroidetes	Flavobacteriia	Flavobacteriales	Flavobacteriaceae
8	Bacteria	FCB group	Bacteroidetes/Chlorobi group	Bacteroidetes	Flavobacteriia	Flavobacteriales	Crocinitomicaceae
8	Bacteria	FCB group	Bacteroidetes/Chlorobi group	Bacteroidetes	Cytophagia	Cytophagales	Flammeovirgaceae
8	Bacteria	FCB group	Bacteroidetes/Chlorobi group	Bacteroidetes	Cytophagia	Cytophagales	Cytophagaceae
8	Bacteria	FCB group	Bacteroidetes/Chlorobi group	Bacteroidetes	Cytophagia	Cytophagales	Cytophagaceae
8	Bacteria	FCB group	Bacteroidetes/Chlorobi group	Bacteroidetes	Cytophagia	Cytophagales	Cytophagaceae
8	Bacteria	FCB group	Bacteroidetes/Chlorobi group	Bacteroidetes	Cytophagia	Cytophagales	Cyclobacteriaceae
8	Bacteria	FCB group	Bacteroidetes/Chlorobi group	Bacteroidetes	Chitinophagia	Chitinophagales	Chitinophagaceae
8	Bacteria	FCB group	Bacteroidetes/Chlorobi group	Bacteroidetes	Chitinophagia	Chitinophagales	Chitinophagaceae
8	Bacteria	FCB group	Bacteroidetes/Chlorobi group	Bacteroidetes	Chitinophagia	Chitinophagales	Chitinophagaceae
8	Bacteria	FCB group	Bacteroidetes/Chlorobi group	Bacteroidetes	Chitinophagia	Chitinophagales	Chitinophagaceae
8	Bacteria	FCB group	Bacteroidetes/Chlorobi group	Bacteroidetes	Bacteroidia	Marinilabiliales	Prolixibacteraceae
8	Bacteria	FCB group	Bacteroidetes/Chlorobi group	Bacteroidetes	Bacteroidia	Bacteroidales	Rikenellaceae
8	Bacteria	FCB group	Bacteroidetes/Chlorobi group	Bacteroidetes	Bacteroidia	Bacteroidales	Prevotellaceae
8	Bacteria	FCB group	Bacteroidetes/Chlorobi group	Bacteroidetes	Bacteroidia	Bacteroidales	Prevotellaceae
8	Bacteria	FCB group	Bacteroidetes/Chlorobi group	Bacteroidetes	Bacteroidia	Bacteroidales	Porphyromonadaceae
8	Bacteria	FCB group	Bacteroidetes/Chlorobi group	Bacteroidetes	Bacteroidia	Bacteroidales	Porphyromonadaceae
8	Bacteria	FCB group	Bacteroidetes/Chlorobi group	Bacteroidetes	Bacteroidia	Bacteroidales	Porphyromonadaceae
8	Bacteria	FCB group	Bacteroidetes/Chlorobi group	Bacteroidetes	Bacteroidia	Bacteroidales	Odoribacteraceae
8	Bacteria	FCB group	Bacteroidetes/Chlorobi group	Bacteroidetes	Bacteroidia	Bacteroidales	Bacteroidaceae
8	Bacteria	FCB group	Bacteroidetes/Chlorobi group	Bacteroidetes	Bacteroidia	Bacteroidales	Bacteroidaceae
8	Bacteria	FCB group	Bacteroidetes/Chlorobi group	Bacteroidetes	Bacteroidia	Bacteroidales	Bacteroidaceae
8	Bacteria	FCB group	Bacteroidetes/Chlorobi group	Bacteroidetes	Bacteroidia	Bacteroidales	Bacteroidaceae
8	Bacteria	FCB group	Bacteroidetes/Chlorobi group	Bacteroidetes	Bacteroidia	Bacteroidales	Bacteroidaceae
8	Bacteria	FCB group	Bacteroidetes/Chlorobi group	Bacteroidetes	Bacteroidia	Bacteroidales	Bacteroidaceae
8	Bacteria	FCB group	Bacteroidetes/Chlorobi group	Bacteroidetes	Bacteroidia	Bacteroidales	Bacteroidaceae
8	Bacteria	FCB group	Bacteroidetes/Chlorobi group	Bacteroidetes	Bacteroidia	Bacteroidales	Bacteroidaceae
8	Bacteria	Balneolaeota	Balneolia	Balneolales	Balneolaceae	Aliifodinibius	Aliifodinibius roseus
8	Bacteria	Acidobacteria	unclassified Acidobacteria	unclassified Acidobacteria (miscellaneous)	Acidobacteria bacterium RIFCSPLOWO2_02_FULL_67_21		
8	Bacteria	Acidobacteria	unclassified Acidobacteria	unclassified Acidobacteria (miscellaneous)	Acidobacteria bacterium RIFCSPLOWO2_02_FULL_60_20		
8	Bacteria	Acidobacteria	unclassified Acidobacteria	unclassified Acidobacteria (miscellaneous)	Acidobacteria bacterium RBG_13_68_16		
8	Bacteria	Acidobacteria	Acidobacteriia	Acidobacteriales	Acidobacteriaceae	unclassified Acidobacteriaceae	Acidobacteriaceae bacterium URHE0068
8	Bacteria	Acidobacteria	Acidobacteriia	Acidobacteriales	Acidobacteriaceae	Terriglobus	
8	Bacteria	Acidobacteria					
8	Archaea	Euryarchaeota	Methanomicrobia	Methanosarcinales	Methanosarcinaceae	Methanosarcina	
7	Eukaryota	Stramenopiles	PX clade	Phaeophyceae	Ectocarpales	Ectocarpaceae	Ectocarpus
7	Eukaryota	Stramenopiles	Oomycetes				
7	Eukaryota	Opisthokonta	Fungi	Mucoromycota	Mucoromycotina	Mucorales	Lichtheimiaceae
7	Eukaryota	Opisthokonta	Fungi	Mucoromycota	Glomeromycotina	Glomeromycetes	Glomerales
7	Eukaryota	Opisthokonta	Fungi	Dikarya	Ascomycota	saccharomyceta	Pezizomycotina
7	Eukaryota	Haptophyceae	Prymnesiales	Chrysochromulinaceae	Chrysochromulina	Chrysochromulina sp. CCMP291	
7	Bacteria	unclassified Bacteria	Bacteria candidate phyla	candidate division NC10	unclassified candidate division NC10	candidate division NC10 bacterium CSP1-5	
7	Bacteria	unclassified Bacteria	Bacteria candidate phyla	candidate division BRC1	unclassified candidate division BRC1	candidate division BRC1 bacterium SM23_51	
7	Bacteria	unclassified Bacteria	Bacteria candidate phyla	Patescibacteria group	Parcubacteria group	Candidatus Uhrbacteria	
7	Bacteria	unclassified Bacteria	Bacteria candidate phyla	Candidatus Lindowbacteria			
7	Bacteria	Terrabacteria group	Firmicutes	Tissierellia	unclassified Tissierellia	Sedimentibacter	Sedimentibacter sp. B4
7	Bacteria	Terrabacteria group	Firmicutes	Tissierellia	unclassified Tissierellia	Dethiosulfatibacter	Dethiosulfatibacter aminovorans
7	Bacteria	Terrabacteria group	Firmicutes	Tissierellia	Tissierellales	Sporanaerobacter	Sporanaerobacter sp. PP17-6a
7	Bacteria	Terrabacteria group	Firmicutes	Tissierellia	Tissierellales	Peptoniphilaceae	Peptoniphilus
7	Bacteria	Terrabacteria group	Firmicutes	Negativicutes	Selenomonadales	Sporomusaceae	Sporomusa
7	Bacteria	Terrabacteria group	Firmicutes	Clostridia	Thermoanaerobacterales	Thermoanaerobacterales Family III. Incertae Sedis	Thermoanaerobacterium
7	Bacteria	Terrabacteria group	Firmicutes	Clostridia	Thermoanaerobacterales	Thermoanaerobacteraceae	Caldanaerobius
7	Bacteria	Terrabacteria group	Firmicutes	Clostridia	Thermoanaerobacterales	Thermoanaerobacteraceae	Caldanaerobacter
7	Bacteria	Terrabacteria group	Firmicutes	Clostridia	Clostridiales	unclassified Clostridiales	Epulopiscium
7	Bacteria	Terrabacteria group	Firmicutes	Clostridia	Clostridiales	unclassified Clostridiales	
7	Bacteria	Terrabacteria group	Firmicutes	Clostridia	Clostridiales	Ruminococcaceae	unclassified Ruminococcaceae
7	Bacteria	Terrabacteria group	Firmicutes	Clostridia	Clostridiales	Ruminococcaceae	unclassified Ruminococcaceae
7	Bacteria	Terrabacteria group	Firmicutes	Clostridia	Clostridiales	Ruminococcaceae	Ruminococcus
7	Bacteria	Terrabacteria group	Firmicutes	Clostridia	Clostridiales	Ruminococcaceae	Papillibacter
7	Bacteria	Terrabacteria group	Firmicutes	Clostridia	Clostridiales	Peptostreptococcaceae	Peptoclostridium
7	Bacteria	Terrabacteria group	Firmicutes	Clostridia	Clostridiales	Peptostreptococcaceae	
7	Bacteria	Terrabacteria group	Firmicutes	Clostridia	Clostridiales	Peptococcaceae	unclassified Peptococcaceae
7	Bacteria	Terrabacteria group	Firmicutes	Clostridia	Clostridiales	Peptococcaceae	unclassified Peptococcaceae
7	Bacteria	Terrabacteria group	Firmicutes	Clostridia	Clostridiales	Peptococcaceae	Desulfotomaculum
7	Bacteria	Terrabacteria group	Firmicutes	Clostridia	Clostridiales	Peptococcaceae	Desulfosporosinus
7	Bacteria	Terrabacteria group	Firmicutes	Clostridia	Clostridiales	Peptococcaceae	Desulfitobacterium
7	Bacteria	Terrabacteria group	Firmicutes	Clostridia	Clostridiales	Peptococcaceae	Dehalobacter
7	Bacteria	Terrabacteria group	Firmicutes	Clostridia	Clostridiales	Lachnospiraceae	Tyzzerella
7	Bacteria	Terrabacteria group	Firmicutes	Clostridia	Clostridiales	Lachnospiraceae	Lachnoclostridium
7	Bacteria	Terrabacteria group	Firmicutes	Clostridia	Clostridiales	Lachnospiraceae	Blautia
7	Bacteria	Terrabacteria group	Firmicutes	Clostridia	Clostridiales	Lachnospiraceae	Anaerosporobacter
7	Bacteria	Terrabacteria group	Firmicutes	Clostridia	Clostridiales	Eubacteriaceae	unclassified Eubacteriaceae
7	Bacteria	Terrabacteria group	Firmicutes	Clostridia	Clostridiales	Eubacteriaceae	Eubacterium
7	Bacteria	Terrabacteria group	Firmicutes	Clostridia	Clostridiales	Eubacteriaceae	Alkalibacter
7	Bacteria	Terrabacteria group	Firmicutes	Clostridia	Clostridiales	Clostridiales incertae sedis	Clostridiales Family XIII. Incertae Sedis
7	Bacteria	Terrabacteria group	Firmicutes	Clostridia	Clostridiales	Clostridiales incertae sedis	Clostridiales Family XII. Incertae Sedis
7	Bacteria	Terrabacteria group	Firmicutes	Clostridia	Clostridiales	Clostridiaceae	Youngiibacter
7	Bacteria	Terrabacteria group	Firmicutes	Clostridia	Clostridiales	Clostridiaceae	Clostridium
7	Bacteria	Terrabacteria group	Firmicutes	Clostridia	Clostridiales	Clostridiaceae	Clostridium
7	Bacteria	Terrabacteria group	Firmicutes	Clostridia	Clostridiales	Clostridiaceae	Clostridium
7	Bacteria	Terrabacteria group	Firmicutes	Bacilli	Lactobacillales	Streptococcaceae	Lactococcus
7	Bacteria	Terrabacteria group	Firmicutes	Bacilli	Bacillales	Bacillaceae	Geobacillus
7	Bacteria	Terrabacteria group	Firmicutes	Bacilli	Bacillales	Bacillaceae	Bacillus
7	Bacteria	Terrabacteria group	Deinococcus-Thermus	Deinococci	Deinococcales	Deinococcaceae	Deinococcus
7	Bacteria	Terrabacteria group	Deinococcus-Thermus	Deinococci	Deinococcales	Deinococcaceae	Deinococcus
7	Bacteria	Terrabacteria group	Cyanobacteria/Melainabacteria group	Cyanobacteria	Synechococcales	Leptolyngbyaceae	Phormidesmis
7	Bacteria	Terrabacteria group	Cyanobacteria/Melainabacteria group	Cyanobacteria	Oscillatoriophycideae	Oscillatoriales	unclassified Oscillatoriales
7	Bacteria	Terrabacteria group	Cyanobacteria/Melainabacteria group	Cyanobacteria	Oscillatoriophycideae	Oscillatoriales	Microcoleaceae
7	Bacteria	Terrabacteria group	Cyanobacteria/Melainabacteria group	Cyanobacteria	Nostocales	Nostocaceae	Anabaena
7	Bacteria	Terrabacteria group	Chloroflexi	unclassified Chloroflexi	unclassified Chloroflexi (miscellaneous)	Chloroflexi bacterium CSP1-4	
7	Bacteria	Terrabacteria group	Chloroflexi	Anaerolineae	Anaerolineales	Anaerolineaceae	unclassified Anaerolineaceae
7	Bacteria	Terrabacteria group	Armatimonadetes	Chthonomonadetes	Chthonomonadales	Chthonomonadaceae	Chthonomonas
7	Bacteria	Terrabacteria group	Actinobacteria	Thermoleophilia	Thermoleophilales	Thermoleophilaceae	Thermoleophilum
7	Bacteria	Terrabacteria group	Actinobacteria	Actinobacteria	unclassified Actinobacteria (class)	unclassified Actinobacteria (class) (miscellaneous)	Actinobacteria bacterium 66_15
7	Bacteria	Terrabacteria group	Actinobacteria	Actinobacteria	Streptosporangiales	Streptosporangiaceae	Nonomuraea
7	Bacteria	Terrabacteria group	Actinobacteria	Actinobacteria	Pseudonocardiales	Pseudonocardiaceae	Pseudonocardia
7	Bacteria	Terrabacteria group	Actinobacteria	Actinobacteria	Pseudonocardiales	Pseudonocardiaceae	Pseudonocardia
7	Bacteria	Terrabacteria group	Actinobacteria	Actinobacteria	Propionibacteriales	Nocardioidaceae	Nocardioides
7	Bacteria	Terrabacteria group	Actinobacteria	Actinobacteria	Propionibacteriales	Nocardioidaceae	Nocardioides
7	Bacteria	Terrabacteria group	Actinobacteria	Actinobacteria	Propionibacteriales	Nocardioidaceae	Nocardioides
7	Bacteria	Terrabacteria group	Actinobacteria	Actinobacteria	Propionibacteriales	Nocardioidaceae	Nocardioides
7	Bacteria	Terrabacteria group	Actinobacteria	Actinobacteria	Propionibacteriales	Nocardioidaceae	Nocardioides
7	Bacteria	Terrabacteria group	Actinobacteria	Actinobacteria	Propionibacteriales	Nocardioidaceae	Nocardioides
7	Bacteria	Terrabacteria group	Actinobacteria	Actinobacteria	Propionibacteriales	Nocardioidaceae	Marmoricola
7	Bacteria	Terrabacteria group	Actinobacteria	Actinobacteria	Micrococcales	Intrasporangiaceae	Phycicoccus
7	Bacteria	Terrabacteria group	Actinobacteria	Actinobacteria	Jiangellales	Jiangellaceae	Jiangella
7	Bacteria	Terrabacteria group	Actinobacteria	Actinobacteria	Geodermatophilales	Geodermatophilaceae	Geodermatophilus
7	Bacteria	Terrabacteria group	Actinobacteria	Actinobacteria	Frankiales	Cryptosporangiaceae	Cryptosporangium
7	Bacteria	Terrabacteria group	Actinobacteria	Actinobacteria	Corynebacteriales	Mycobacteriaceae	Mycobacterium
7	Bacteria	Terrabacteria group	Actinobacteria	Actinobacteria	Corynebacteriales	Gordoniaceae	Gordonia
7	Bacteria	Terrabacteria group	Actinobacteria	Actinobacteria	Actinomycetales	Actinomycetaceae	Varibaculum
7	Bacteria	Terrabacteria group	Actinobacteria	Acidimicrobiia	Acidimicrobiales	Acidimicrobiaceae	Acidithrix
7	Bacteria	Terrabacteria group	Actinobacteria	Acidimicrobiia	Acidimicrobiales	Acidimicrobiaceae	Acidimicrobium
7	Bacteria	Spirochaetes	unclassified Spirochaetes	Spirochaetes bacterium RBG_16_49_21			
7	Bacteria	Spirochaetes	unclassified Spirochaetes	Spirochaetes bacterium GWF1_41_5			
7	Bacteria	Spirochaetes	Spirochaetia	Spirochaetales	Spirochaetaceae	Treponema	Treponema lecithinolyticum
7	Bacteria	Spirochaetes	Spirochaetia	Spirochaetales	Spirochaetaceae	Spirochaeta	Spirochaeta lutea
7	Bacteria	Spirochaetes	Spirochaetia	Brachyspirales	Brachyspiraceae	Brachyspira	
7	Bacteria	Proteobacteria	unclassified Proteobacteria	unclassified Proteobacteria (miscellaneous)	Proteobacteria bacterium ST_bin16		
7	Bacteria	Proteobacteria	unclassified Proteobacteria	unclassified Proteobacteria (miscellaneous)	Proteobacteria bacterium SG_bin7		
7	Bacteria	Proteobacteria	delta/epsilon subdivisions	Epsilonproteobacteria	Campylobacterales	Campylobacteraceae	Campylobacter
7	Bacteria	Proteobacteria	delta/epsilon subdivisions	Epsilonproteobacteria	Campylobacterales	Campylobacteraceae	Arcobacter
7	Bacteria	Proteobacteria	delta/epsilon subdivisions	Deltaproteobacteria	unclassified Deltaproteobacteria	unclassified Deltaproteobacteria (miscellaneous)	delta proteobacterium NaphS2
7	Bacteria	Proteobacteria	delta/epsilon subdivisions	Deltaproteobacteria	unclassified Deltaproteobacteria	unclassified Deltaproteobacteria (miscellaneous)	Deltaproteobacteria bacterium RIFOXYA2_FULL_55_11
7	Bacteria	Proteobacteria	delta/epsilon subdivisions	Deltaproteobacteria	unclassified Deltaproteobacteria	unclassified Deltaproteobacteria (miscellaneous)	Deltaproteobacteria bacterium RIFCSPLOWO2_12_FULL_60_19
7	Bacteria	Proteobacteria	delta/epsilon subdivisions	Deltaproteobacteria	unclassified Deltaproteobacteria	unclassified Deltaproteobacteria (miscellaneous)	Deltaproteobacteria bacterium GWA2_45_12
7	Bacteria	Proteobacteria	delta/epsilon subdivisions	Deltaproteobacteria	Desulfuromonadales	Geobacteraceae	Geopsychrobacter
7	Bacteria	Proteobacteria	delta/epsilon subdivisions	Deltaproteobacteria	Desulfuromonadales	Geobacteraceae	Geobacter
7	Bacteria	Proteobacteria	delta/epsilon subdivisions	Deltaproteobacteria	Desulfuromonadales	Geobacteraceae	Geoalkalibacter
7	Bacteria	Proteobacteria	delta/epsilon subdivisions	Deltaproteobacteria	Desulfovibrionales	Desulfovibrionaceae	Desulfovibrio
7	Bacteria	Proteobacteria	delta/epsilon subdivisions	Deltaproteobacteria	Desulfovibrionales	Desulfovibrionaceae	Desulfovibrio
7	Bacteria	Proteobacteria	delta/epsilon subdivisions	Deltaproteobacteria	Desulfovibrionales	Desulfovibrionaceae	Desulfovibrio
7	Bacteria	Proteobacteria	delta/epsilon subdivisions	Deltaproteobacteria	Desulfovibrionales	Desulfonatronaceae	Desulfonatronum
7	Bacteria	Proteobacteria	delta/epsilon subdivisions	Deltaproteobacteria	Desulfovibrionales	Desulfohalobiaceae	Desulfonatronovibrio
7	Bacteria	Proteobacteria	delta/epsilon subdivisions	Deltaproteobacteria	Desulfobacterales	Desulfobulbaceae	Desulfotalea
7	Bacteria	Proteobacteria	delta/epsilon subdivisions	Deltaproteobacteria	Desulfobacterales	Desulfobulbaceae	Desulfocapsa
7	Bacteria	Proteobacteria	delta/epsilon subdivisions	Deltaproteobacteria	Desulfobacterales	Desulfobacteraceae	unclassified Desulfobacteraceae
7	Bacteria	Proteobacteria	delta/epsilon subdivisions	Deltaproteobacteria	Desulfobacterales	Desulfobacteraceae	Desulfococcus
7	Bacteria	Proteobacteria	delta/epsilon subdivisions	Deltaproteobacteria	Bdellovibrionales	Pseudobacteriovoracaceae	Pseudobacteriovorax
7	Bacteria	Proteobacteria	Gammaproteobacteria	unclassified Gammaproteobacteria	unclassified Gammaproteobacteria (miscellaneous)	Gammaproteobacteria bacterium RIFCSPLOWO2_02_FULL_56_15	
7	Bacteria	Proteobacteria	Gammaproteobacteria	unclassified Gammaproteobacteria	unclassified Gammaproteobacteria (miscellaneous)	Gammaproteobacteria bacterium RBG_16_57_12	
7	Bacteria	Proteobacteria	Gammaproteobacteria	unclassified Gammaproteobacteria	Thiolapillus	Thiolapillus brandeum	
7	Bacteria	Proteobacteria	Gammaproteobacteria	Xanthomonadales	Xanthomonadaceae	Stenotrophomonas	Stenotrophomonas ginsengisoli
7	Bacteria	Proteobacteria	Gammaproteobacteria	Xanthomonadales	Xanthomonadaceae	Pseudoxanthomonas	Pseudoxanthomonas sp. CF385
7	Bacteria	Proteobacteria	Gammaproteobacteria	Xanthomonadales	Xanthomonadaceae	Arenimonas	
7	Bacteria	Proteobacteria	Gammaproteobacteria	Xanthomonadales	Rhodanobacteraceae	unclassified Rhodanobacteraceae	Dyella-like sp. DHo
7	Bacteria	Proteobacteria	Gammaproteobacteria	Xanthomonadales	Rhodanobacteraceae	Frateuria	Frateuria terrea
7	Bacteria	Proteobacteria	Gammaproteobacteria	Vibrionales	Vibrionaceae	Vibrio	Vibrio harveyi group
7	Bacteria	Proteobacteria	Gammaproteobacteria	Vibrionales	Vibrionaceae		
7	Bacteria	Proteobacteria	Gammaproteobacteria	Thiotrichales	Thiotrichaceae	Thiothrix	
7	Bacteria	Proteobacteria	Gammaproteobacteria	Thiotrichales	Thiotrichaceae	Beggiatoa	Beggiatoa sp. PS
7	Bacteria	Proteobacteria	Gammaproteobacteria	Salinisphaerales	Salinisphaeraceae	Salinisphaera	Salinisphaera hydrothermalis
7	Bacteria	Proteobacteria	Gammaproteobacteria	Pseudomonadales	Pseudomonadaceae	Pseudomonas	Pseudomonas stutzeri group
7	Bacteria	Proteobacteria	Gammaproteobacteria	Pseudomonadales	Pseudomonadaceae	Pseudomonas	Pseudomonas sp. NBRC 111119
7	Bacteria	Proteobacteria	Gammaproteobacteria	Pseudomonadales	Pseudomonadaceae	Pseudomonas	Pseudomonas sp. CC6-YY-74
7	Bacteria	Proteobacteria	Gammaproteobacteria	Pseudomonadales	Pseudomonadaceae	Pseudomonas	Pseudomonas putida group
7	Bacteria	Proteobacteria	Gammaproteobacteria	Pseudomonadales	Pseudomonadaceae	Pseudomonas	Pseudomonas oryzae
7	Bacteria	Proteobacteria	Gammaproteobacteria	Pseudomonadales	Pseudomonadaceae	Pseudomonas	Pseudomonas aeruginosa group
7	Bacteria	Proteobacteria	Gammaproteobacteria	Pseudomonadales	Pseudomonadaceae	Pseudomonas	Pseudomonas aeruginosa group
7	Bacteria	Proteobacteria	Gammaproteobacteria	Pseudomonadales	Moraxellaceae	Alkanindiges	
7	Bacteria	Proteobacteria	Gammaproteobacteria	Pasteurellales	Pasteurellaceae	Haemophilus	
7	Bacteria	Proteobacteria	Gammaproteobacteria	Oceanospirillales	Saccharospirillaceae	Saccharospirillum	Saccharospirillum impatiens
7	Bacteria	Proteobacteria	Gammaproteobacteria	Oceanospirillales	Oceanospirillaceae	unclassified Oceanospirillaceae	Osedax symbiont Rs2
7	Bacteria	Proteobacteria	Gammaproteobacteria	Oceanospirillales	Oceanospirillaceae	Motiliproteus	Motiliproteus sp. MSK22-1
7	Bacteria	Proteobacteria	Gammaproteobacteria	Oceanospirillales	Oceanospirillaceae	Marinobacterium	Marinobacterium sp. ST58-10
7	Bacteria	Proteobacteria	Gammaproteobacteria	Oceanospirillales	Halomonadaceae	Halomonas	Halomonas utahensis
7	Bacteria	Proteobacteria	Gammaproteobacteria	Oceanospirillales	Halomonadaceae	Cobetia	
7	Bacteria	Proteobacteria	Gammaproteobacteria	Oceanospirillales	Hahellaceae	Endozoicomonas	Endozoicomonas ascidiicola
7	Bacteria	Proteobacteria	Gammaproteobacteria	Oceanospirillales	Hahellaceae	Endozoicomonas	
7	Bacteria	Proteobacteria	Gammaproteobacteria	Oceanospirillales	Hahellaceae		
7	Bacteria	Proteobacteria	Gammaproteobacteria	Enterobacterales	Yersiniaceae	Serratia	Serratia sp. Leaf51
7	Bacteria	Proteobacteria	Gammaproteobacteria	Enterobacterales	Yersiniaceae	Serratia	Serratia sp. DD3
7	Bacteria	Proteobacteria	Gammaproteobacteria	Enterobacterales	Yersiniaceae	Rahnella	Rahnella aquatilis
7	Bacteria	Proteobacteria	Gammaproteobacteria	Enterobacterales	Morganellaceae	Xenorhabdus	Xenorhabdus bovienii
7	Bacteria	Proteobacteria	Gammaproteobacteria	Enterobacterales	Erwiniaceae	Tatumella	Tatumella morbirosei
7	Bacteria	Proteobacteria	Gammaproteobacteria	Enterobacterales	Enterobacteriaceae	unclassified Enterobacteriaceae	ant, tsetse, mealybug, aphid, etc. endosymbionts
7	Bacteria	Proteobacteria	Gammaproteobacteria	Enterobacterales	Enterobacteriaceae	Klebsiella	Klebsiella pneumoniae
7	Bacteria	Proteobacteria	Gammaproteobacteria	Chromatiales	Halothiobacillaceae	Halothiobacillus	Halothiobacillus neapolitanus
7	Bacteria	Proteobacteria	Gammaproteobacteria	Chromatiales	Ectothiorhodospiraceae	Thiohalospira	Thiohalospira halophila
7	Bacteria	Proteobacteria	Gammaproteobacteria	Chromatiales	Ectothiorhodospiraceae	Acidihalobacter	Acidihalobacter ferrooxidans
7	Bacteria	Proteobacteria	Gammaproteobacteria	Chromatiales	Chromatiaceae	Thiorhodococcus	Thiorhodococcus sp. AK35
7	Bacteria	Proteobacteria	Gammaproteobacteria	Chromatiales	Chromatiaceae	Rheinheimera	Rheinheimera sp. SA_1
7	Bacteria	Proteobacteria	Gammaproteobacteria	Chromatiales	Chromatiaceae	Nitrosococcus	Nitrosococcus halophilus
7	Bacteria	Proteobacteria	Gammaproteobacteria	Chromatiales	Chromatiaceae	Candidatus Nitrosoglobus	Candidatus Nitrosoglobus terrae
7	Bacteria	Proteobacteria	Gammaproteobacteria	Chromatiales	Chromatiaceae	Allochromatium	Allochromatium warmingii
7	Bacteria	Proteobacteria	Gammaproteobacteria	Chromatiales	Chromatiaceae	Allochromatium	Allochromatium vinosum
7	Bacteria	Proteobacteria	Gammaproteobacteria	Chromatiales	Chromatiaceae		
7	Bacteria	Proteobacteria	Gammaproteobacteria	Cellvibrionales	Spongiibacteraceae	Spongiibacter	Spongiibacter sp. IMCC21906
7	Bacteria	Proteobacteria	Gammaproteobacteria	Alteromonadales	unclassified Alteromonadales	Alteromonadales bacterium BS08	
7	Bacteria	Proteobacteria	Gammaproteobacteria	Alteromonadales	Idiomarinaceae	Idiomarina	
7	Bacteria	Proteobacteria	Gammaproteobacteria	Alteromonadales	Alteromonadaceae	Aestuariibacter	Aestuariibacter salexigens
7	Bacteria	Proteobacteria	Candidatus Muproteobacteria	Candidatus Muproteobacteria bacterium RBG_16_64_11			
7	Bacteria	Proteobacteria	Betaproteobacteria	unclassified Betaproteobacteria	unclassified Betaproteobacteria (miscellaneous)	Betaproteobacteria bacterium RIFCSPLOWO2_02_FULL_63_19	
7	Bacteria	Proteobacteria	Betaproteobacteria	Nitrosomonadales	Nitrosomonadaceae	Nitrosovibrio	Nitrosovibrio tenuis
7	Bacteria	Proteobacteria	Betaproteobacteria	Neisseriales	Neisseriaceae	Stenoxybacter	Stenoxybacter acetivorans
7	Bacteria	Proteobacteria	Betaproteobacteria	Neisseriales	Neisseriaceae	Neisseria	Neisseria meningitidis
7	Bacteria	Proteobacteria	Betaproteobacteria	Neisseriales	Neisseriaceae	Eikenella	
7	Bacteria	Proteobacteria	Betaproteobacteria	Neisseriales	Chromobacteriaceae	Paludibacterium	Paludibacterium yongneupense
7	Bacteria	Proteobacteria	Betaproteobacteria	Methylophilales	Methylophilaceae	Methylotenera	Methylotenera sp. 1P/1
7	Bacteria	Proteobacteria	Betaproteobacteria	Hydrogenophilales	Hydrogenophilaceae	Thiobacillus	Thiobacillus thioparus
7	Bacteria	Proteobacteria	Betaproteobacteria	Hydrogenophilales	Hydrogenophilaceae	Tepidiphilus	Tepidiphilus thermophilus
7	Bacteria	Proteobacteria	Betaproteobacteria	Gallionellales	Gallionellaceae	Sideroxydans	Sideroxydans sp. GWF2_59_14
7	Bacteria	Proteobacteria	Betaproteobacteria	Gallionellales	Gallionellaceae	Gallionella	Gallionella capsiferriformans
7	Bacteria	Proteobacteria	Betaproteobacteria	Burkholderiales	unclassified Burkholderiales	unclassified Burkholderiales (miscellaneous)	Burkholderiales bacterium RIFCSPLOWO2_12_FULL_64_99
7	Bacteria	Proteobacteria	Betaproteobacteria	Burkholderiales	unclassified Burkholderiales	unclassified Burkholderiales (miscellaneous)	Burkholderiales bacterium RIFCSPLOWO2_12_67_14
7	Bacteria	Proteobacteria	Betaproteobacteria	Burkholderiales	unclassified Burkholderiales	unclassified Burkholderiales (miscellaneous)	Burkholderiales bacterium GWA2_64_37
7	Bacteria	Proteobacteria	Betaproteobacteria	Burkholderiales	unclassified Burkholderiales	Burkholderiales Genera incertae sedis	Leptothrix
7	Bacteria	Proteobacteria	Betaproteobacteria	Burkholderiales	Sutterellaceae	Sutterella	
7	Bacteria	Proteobacteria	Betaproteobacteria	Burkholderiales	Oxalobacteraceae	unclassified Oxalobacteraceae	Oxalobacteraceae bacterium AB_14
7	Bacteria	Proteobacteria	Betaproteobacteria	Burkholderiales	Oxalobacteraceae	Herbaspirillum	Herbaspirillum sp. WT00C
7	Bacteria	Proteobacteria	Betaproteobacteria	Burkholderiales	Oxalobacteraceae	Herbaspirillum	Herbaspirillum sp. RV1423
7	Bacteria	Proteobacteria	Betaproteobacteria	Burkholderiales	Oxalobacteraceae	Duganella	Duganella sp. CF517
7	Bacteria	Proteobacteria	Betaproteobacteria	Burkholderiales	Oxalobacteraceae	Duganella	Duganella sacchari
7	Bacteria	Proteobacteria	Betaproteobacteria	Burkholderiales	Comamonadaceae	unclassified Comamonadaceae	Comamonadaceae bacterium SCN 68-20
7	Bacteria	Proteobacteria	Betaproteobacteria	Burkholderiales	Comamonadaceae	Comamonas	Comamonas thiooxydans
7	Bacteria	Proteobacteria	Betaproteobacteria	Burkholderiales	Comamonadaceae	Brachymonas	
7	Bacteria	Proteobacteria	Betaproteobacteria	Burkholderiales	Burkholderiaceae	unclassified Burkholderiaceae	Burkholderiaceae bacterium 16
7	Bacteria	Proteobacteria	Betaproteobacteria	Burkholderiales	Burkholderiaceae	Ralstonia	Ralstonia pickettii
7	Bacteria	Proteobacteria	Betaproteobacteria	Burkholderiales	Burkholderiaceae	Paraburkholderia	Paraburkholderia piptadeniae
7	Bacteria	Proteobacteria	Betaproteobacteria	Burkholderiales	Burkholderiaceae	Paraburkholderia	Paraburkholderia fungorum
7	Bacteria	Proteobacteria	Betaproteobacteria	Burkholderiales	Burkholderiaceae	Limnobacter	Limnobacter sp. CACIAM 66H1
7	Bacteria	Proteobacteria	Betaproteobacteria	Burkholderiales	Burkholderiaceae	Cupriavidus	Cupriavidus pinatubonensis
7	Bacteria	Proteobacteria	Betaproteobacteria	Burkholderiales	Burkholderiaceae	Burkholderia	Burkholderia sp. lig30
7	Bacteria	Proteobacteria	Betaproteobacteria	Burkholderiales	Burkholderiaceae	Burkholderia	Burkholderia sp. WSM4176
7	Bacteria	Proteobacteria	Betaproteobacteria	Burkholderiales	Burkholderiaceae	Burkholderia	Burkholderia sp. E7m39
7	Bacteria	Proteobacteria	Betaproteobacteria	Burkholderiales	Burkholderiaceae	Burkholderia	Burkholderia cepacia complex
7	Bacteria	Proteobacteria	Betaproteobacteria	Burkholderiales	Alcaligenaceae	Bordetella	Bordetella hinzii
7	Bacteria	Proteobacteria	Betaproteobacteria	Burkholderiales	Alcaligenaceae	Achromobacter	Achromobacter xylosoxidans
7	Bacteria	Proteobacteria	Alphaproteobacteria	unclassified Alphaproteobacteria	unclassified Alphaproteobacteria (miscellaneous)	Alphaproteobacteria bacterium RIFOXYD12_FULL_60_8	
7	Bacteria	Proteobacteria	Alphaproteobacteria	unclassified Alphaproteobacteria	unclassified Alphaproteobacteria (miscellaneous)	Alphaproteobacteria bacterium CG1_02_46_17	
7	Bacteria	Proteobacteria	Alphaproteobacteria	unclassified Alphaproteobacteria	unclassified Alphaproteobacteria (miscellaneous)	Alphaproteobacteria bacterium 65-7	
7	Bacteria	Proteobacteria	Alphaproteobacteria	Sphingomonadales	Sphingomonadaceae	Sphingopyxis	Sphingopyxis sp. A083
7	Bacteria	Proteobacteria	Alphaproteobacteria	Sphingomonadales	Sphingomonadaceae	Sphingomonas	Sphingomonas sp. Leaf208
7	Bacteria	Proteobacteria	Alphaproteobacteria	Sphingomonadales	Sphingomonadaceae	Sphingomonas	Sphingomonas sp. Leaf198
7	Bacteria	Proteobacteria	Alphaproteobacteria	Sphingomonadales	Sphingomonadaceae	Sphingomonas	Sphingomonas haloaromaticamans
7	Bacteria	Proteobacteria	Alphaproteobacteria	Sphingomonadales	Sphingomonadaceae	Novosphingobium	Novosphingobium capsulatum
7	Bacteria	Proteobacteria	Alphaproteobacteria	Sphingomonadales	Erythrobacteraceae	Erythrobacter	Erythrobacter sp. EhN03
7	Bacteria	Proteobacteria	Alphaproteobacteria	Rhodospirillales	unclassified Rhodospirillales		
7	Bacteria	Proteobacteria	Alphaproteobacteria	Rhodospirillales	Rhodospirillaceae	Thalassospira	Thalassospira mesophila
7	Bacteria	Proteobacteria	Alphaproteobacteria	Rhodospirillales	Rhodospirillaceae	Magnetospirillum	Magnetospirillum moscoviense
7	Bacteria	Proteobacteria	Alphaproteobacteria	Rhodospirillales	Rhodospirillaceae	Magnetospirillum	Magnetospirillum magneticum
7	Bacteria	Proteobacteria	Alphaproteobacteria	Rhodospirillales	Rhodospirillaceae	Magnetospirillum	Magnetospirillum gryphiswaldense
7	Bacteria	Proteobacteria	Alphaproteobacteria	Rhodospirillales	Rhodospirillaceae	Magnetospirillum	Magnetospirillum caucaseum
7	Bacteria	Proteobacteria	Alphaproteobacteria	Rhodospirillales	Rhodospirillaceae	Haematospirillum	Haematospirillum jordaniae
7	Bacteria	Proteobacteria	Alphaproteobacteria	Rhodospirillales	Rhodospirillaceae	Fodinicurvata	Fodinicurvata sediminis
7	Bacteria	Proteobacteria	Alphaproteobacteria	Rhodospirillales	Acetobacteraceae	Belnapia	Belnapia sp. F-4-1
7	Bacteria	Proteobacteria	Alphaproteobacteria	Rhodospirillales	Acetobacteraceae	Acetobacter	Acetobacter subgen. Acetobacter
7	Bacteria	Proteobacteria	Alphaproteobacteria	Rhodobacterales	environmental samples	uncultured Rhodobacterales bacterium HF4000_03E16	
7	Bacteria	Proteobacteria	Alphaproteobacteria	Rhodobacterales	Rhodobacteraceae	Tateyamaria	Tateyamaria omphalii
7	Bacteria	Proteobacteria	Alphaproteobacteria	Rhodobacterales	Rhodobacteraceae	Sulfitobacter	Sulfitobacter sp. 20_GPM-1509m
7	Bacteria	Proteobacteria	Alphaproteobacteria	Rhodobacterales	Rhodobacteraceae	Stappia	
7	Bacteria	Proteobacteria	Alphaproteobacteria	Rhodobacterales	Rhodobacteraceae	Rhodovulum	Rhodovulum sp. NI22
7	Bacteria	Proteobacteria	Alphaproteobacteria	Rhodobacterales	Rhodobacteraceae	Pseudovibrio	Pseudovibrio sp. Tun.PHSC04-5.I4
7	Bacteria	Proteobacteria	Alphaproteobacteria	Rhodobacterales	Rhodobacteraceae	Phaeobacter	Phaeobacter sp. CECT 5382
7	Bacteria	Proteobacteria	Alphaproteobacteria	Rhizobiales	unclassified Rhizobiales	unclassified Rhizobiales (miscellaneous)	Rhizobiales bacterium GAS113
7	Bacteria	Proteobacteria	Alphaproteobacteria	Rhizobiales	unclassified Rhizobiales	Methyloceanibacter	Methyloceanibacter stevinii
7	Bacteria	Proteobacteria	Alphaproteobacteria	Rhizobiales	unclassified Rhizobiales	Methyloceanibacter	Methyloceanibacter methanicus
7	Bacteria	Proteobacteria	Alphaproteobacteria	Rhizobiales	unclassified Rhizobiales	Methyloceanibacter	Methyloceanibacter caenitepidi
7	Bacteria	Proteobacteria	Alphaproteobacteria	Rhizobiales	Rhizobiaceae	Rhizobium/Agrobacterium group	Rhizobium
7	Bacteria	Proteobacteria	Alphaproteobacteria	Rhizobiales	Rhizobiaceae	Rhizobium/Agrobacterium group	Rhizobium
7	Bacteria	Proteobacteria	Alphaproteobacteria	Rhizobiales	Rhizobiaceae	Rhizobium/Agrobacterium group	Rhizobium
7	Bacteria	Proteobacteria	Alphaproteobacteria	Rhizobiales	Rhizobiaceae	Rhizobium/Agrobacterium group	Rhizobium
7	Bacteria	Proteobacteria	Alphaproteobacteria	Rhizobiales	Phyllobacteriaceae	Pseudaminobacter	Pseudaminobacter salicylatoxidans
7	Bacteria	Proteobacteria	Alphaproteobacteria	Rhizobiales	Phyllobacteriaceae	Pseudaminobacter	Pseudaminobacter manganicus
7	Bacteria	Proteobacteria	Alphaproteobacteria	Rhizobiales	Phyllobacteriaceae	Nitratireductor	Nitratireductor aquibiodomus
7	Bacteria	Proteobacteria	Alphaproteobacteria	Rhizobiales	Phyllobacteriaceae	Mesorhizobium	Mesorhizobium sp. LSJC280B00
7	Bacteria	Proteobacteria	Alphaproteobacteria	Rhizobiales	Methylobacteriaceae	Methylobacterium	Methylobacterium mesophilicum
7	Bacteria	Proteobacteria	Alphaproteobacteria	Rhizobiales	Hyphomicrobiaceae	Devosia	Devosia sp. Root635
7	Bacteria	Proteobacteria	Alphaproteobacteria	Rhizobiales	Bradyrhizobiaceae	Bradyrhizobium	Bradyrhizobium yuanmingense
7	Bacteria	Proteobacteria	Alphaproteobacteria	Rhizobiales	Bradyrhizobiaceae	Bradyrhizobium	Bradyrhizobium sp. URHD0069
7	Bacteria	Proteobacteria	Alphaproteobacteria	Rhizobiales	Bradyrhizobiaceae	Bradyrhizobium	Bradyrhizobium sp. S23321
7	Bacteria	Proteobacteria	Alphaproteobacteria	Rhizobiales	Bradyrhizobiaceae	Bradyrhizobium	Bradyrhizobium sp. LTSPM299
7	Bacteria	Proteobacteria	Alphaproteobacteria	Rhizobiales	Bradyrhizobiaceae	Bosea	Bosea sp. UNC402CLCol
7	Bacteria	Proteobacteria	Alphaproteobacteria	Rhizobiales	Bradyrhizobiaceae	Bosea	Bosea sp. Root483D1
7	Bacteria	Proteobacteria	Alphaproteobacteria	Rhizobiales	Bradyrhizobiaceae	Bosea	Bosea sp. LC85
7	Bacteria	Proteobacteria	Alphaproteobacteria	Rhizobiales	Bartonellaceae	Bartonella	Bartonella tamiae
7	Bacteria	Proteobacteria	Alphaproteobacteria	Rhizobiales	Aurantimonadaceae	Aureimonas	Aureimonas sp. Leaf454
7	Bacteria	Proteobacteria	Alphaproteobacteria	Rhizobiales	Aurantimonadaceae	Aureimonas	Aureimonas sp. AU4
7	Bacteria	Proteobacteria	Alphaproteobacteria	Rhizobiales	Aurantimonadaceae	Aurantimonas	Aurantimonas coralicida
7	Bacteria	Proteobacteria	Alphaproteobacteria	Micropepsales	Micropepsaceae	Rhizomicrobium	Rhizomicrobium sp. SCGC AG-212-E05
7	Bacteria	Proteobacteria	Alphaproteobacteria	Caulobacterales	Caulobacteraceae	Caulobacter	Caulobacter sp. AP07
7	Bacteria	Proteobacteria	Alphaproteobacteria	Caulobacterales	Caulobacteraceae	Asticcacaulis	Asticcacaulis taihuensis
7	Bacteria	Proteobacteria	Alphaproteobacteria	Caulobacterales	Caulobacteraceae	Asticcacaulis	Asticcacaulis sp. AC402
7	Bacteria	Proteobacteria	Alphaproteobacteria	Caulobacterales	Caulobacteraceae	Asticcacaulis	Asticcacaulis excentricus
7	Bacteria	Proteobacteria	Alphaproteobacteria	Caulobacterales	Caulobacteraceae		
7	Bacteria	PVC group	Verrucomicrobia	Opitutae	Opitutales	Opitutaceae	Cephaloticoccus
7	Bacteria	PVC group	Planctomycetes	unclassified Planctomycetes	Planctomycetes bacterium SM23_32		
7	Bacteria	PVC group	Planctomycetes	unclassified Planctomycetes	Planctomycetes bacterium RBG_16_55_9		
7	Bacteria	PVC group	Planctomycetes	unclassified Planctomycetes	Planctomycetes bacterium RBG_13_50_24		
7	Bacteria	PVC group	Planctomycetes	Planctomycetia	Planctomycetales	Planctomycetaceae	Planctopirus
7	Bacteria	PVC group	Planctomycetes	Phycisphaerae	unclassified Phycisphaerae	Phycisphaerae bacterium SG8_4	
7	Bacteria	PVC group	Candidatus Omnitrophica	unclassified Candidatus Omnitrophica	Omnitrophica bacterium RIFOXYB12_FULL_50_7		
7	Bacteria	PVC group	Candidatus Omnitrophica	unclassified Candidatus Omnitrophica	Omnitrophica WOR_2 bacterium SM23_29		
7	Bacteria	Nitrospirae	Nitrospira	unclassified Nitrospira	Nitrospira bacterium SM23_35		
7	Bacteria	Nitrospirae	Nitrospira	unclassified Nitrospira	Nitrospira bacterium SG8_35_1		
7	Bacteria	Nitrospirae	Nitrospira	Nitrospirales	Nitrospiraceae	Candidatus Magnetoovum	Candidatus Magnetoovum chiemensis
7	Bacteria	Fusobacteria	Fusobacteriia	Fusobacteriales	Fusobacteriaceae	Cetobacterium	
7	Bacteria	FCB group	Gemmatimonadetes	unclassified Gemmatimonadetes	Gemmatimonadetes bacterium GWC2_71_10		
7	Bacteria	FCB group	Gemmatimonadetes	Gemmatimonadetes	Gemmatimonadales	Gemmatimonadaceae	Gemmatimonas
7	Bacteria	FCB group	Gemmatimonadetes	Gemmatimonadetes	Gemmatimonadales	Gemmatimonadaceae	Gemmatimonas
7	Bacteria	FCB group	Candidatus Marinimicrobia	Candidatus Marinimicrobia bacterium CG1_02_48_14			
7	Bacteria	FCB group	Bacteroidetes/Chlorobi group	unclassified Bacteroidetes/Chlorobi group	bacterium 336/3		
7	Bacteria	FCB group	Bacteroidetes/Chlorobi group	Ignavibacteriae	Ignavibacteria	unclassified Ignavibacteria	Ignavibacteria bacterium RBG_13_36_8
7	Bacteria	FCB group	Bacteroidetes/Chlorobi group	Chlorobi	Chlorobia	Chlorobiales	Chlorobiaceae
7	Bacteria	FCB group	Bacteroidetes/Chlorobi group	Bacteroidetes	unclassified Bacteroidetes	unclassified Bacteroidetes (miscellaneous)	Bacteroidetes bacterium GWA2_31_9b
7	Bacteria	FCB group	Bacteroidetes/Chlorobi group	Bacteroidetes	unclassified Bacteroidetes	unclassified Bacteroidetes (miscellaneous)	Bacteroidetes bacterium CG2_30_32_10
7	Bacteria	FCB group	Bacteroidetes/Chlorobi group	Bacteroidetes	unclassified Bacteroidetes	unclassified Bacteroidetes (miscellaneous)	Bacteroidetes bacterium 4572_128
7	Bacteria	FCB group	Bacteroidetes/Chlorobi group	Bacteroidetes	Sphingobacteriia	unclassified Sphingobacteriia	
7	Bacteria	FCB group	Bacteroidetes/Chlorobi group	Bacteroidetes	Sphingobacteriia	Sphingobacteriales	unclassified Sphingobacteriales
7	Bacteria	FCB group	Bacteroidetes/Chlorobi group	Bacteroidetes	Sphingobacteriia	Sphingobacteriales	Sphingobacteriaceae
7	Bacteria	FCB group	Bacteroidetes/Chlorobi group	Bacteroidetes	Sphingobacteriia	Sphingobacteriales	Sphingobacteriaceae
7	Bacteria	FCB group	Bacteroidetes/Chlorobi group	Bacteroidetes	Sphingobacteriia	Sphingobacteriales	Sphingobacteriaceae
7	Bacteria	FCB group	Bacteroidetes/Chlorobi group	Bacteroidetes	Sphingobacteriia	Sphingobacteriales	Sphingobacteriaceae
7	Bacteria	FCB group	Bacteroidetes/Chlorobi group	Bacteroidetes	Saprospiria	Saprospirales	Saprospiraceae
7	Bacteria	FCB group	Bacteroidetes/Chlorobi group	Bacteroidetes	Flavobacteriia	Flavobacteriales	Flavobacteriaceae
7	Bacteria	FCB group	Bacteroidetes/Chlorobi group	Bacteroidetes	Flavobacteriia	Flavobacteriales	Flavobacteriaceae
7	Bacteria	FCB group	Bacteroidetes/Chlorobi group	Bacteroidetes	Flavobacteriia	Flavobacteriales	Flavobacteriaceae
7	Bacteria	FCB group	Bacteroidetes/Chlorobi group	Bacteroidetes	Flavobacteriia	Flavobacteriales	Flavobacteriaceae
7	Bacteria	FCB group	Bacteroidetes/Chlorobi group	Bacteroidetes	Flavobacteriia	Flavobacteriales	Flavobacteriaceae
7	Bacteria	FCB group	Bacteroidetes/Chlorobi group	Bacteroidetes	Flavobacteriia	Flavobacteriales	Cryomorphaceae
7	Bacteria	FCB group	Bacteroidetes/Chlorobi group	Bacteroidetes	Flavobacteriia	Flavobacteriales	Crocinitomicaceae
7	Bacteria	FCB group	Bacteroidetes/Chlorobi group	Bacteroidetes	Cytophagia	Cytophagales	Flammeovirgaceae
7	Bacteria	FCB group	Bacteroidetes/Chlorobi group	Bacteroidetes	Cytophagia	Cytophagales	Flammeovirgaceae
7	Bacteria	FCB group	Bacteroidetes/Chlorobi group	Bacteroidetes	Cytophagia	Cytophagales	Flammeovirgaceae
7	Bacteria	FCB group	Bacteroidetes/Chlorobi group	Bacteroidetes	Cytophagia	Cytophagales	Cytophagaceae
7	Bacteria	FCB group	Bacteroidetes/Chlorobi group	Bacteroidetes	Cytophagia	Cytophagales	Cytophagaceae
7	Bacteria	FCB group	Bacteroidetes/Chlorobi group	Bacteroidetes	Cytophagia	Cytophagales	Cytophagaceae
7	Bacteria	FCB group	Bacteroidetes/Chlorobi group	Bacteroidetes	Cytophagia	Cytophagales	Cytophagaceae
7	Bacteria	FCB group	Bacteroidetes/Chlorobi group	Bacteroidetes	Chitinophagia	Chitinophagales	Chitinophagaceae
7	Bacteria	FCB group	Bacteroidetes/Chlorobi group	Bacteroidetes	Chitinophagia	Chitinophagales	Chitinophagaceae
7	Bacteria	FCB group	Bacteroidetes/Chlorobi group	Bacteroidetes	Chitinophagia	Chitinophagales	Chitinophagaceae
7	Bacteria	FCB group	Bacteroidetes/Chlorobi group	Bacteroidetes	Chitinophagia	Chitinophagales	Chitinophagaceae
7	Bacteria	FCB group	Bacteroidetes/Chlorobi group	Bacteroidetes	Bacteroidia	Bacteroidales	unclassified Bacteroidales
7	Bacteria	FCB group	Bacteroidetes/Chlorobi group	Bacteroidetes	Bacteroidia	Bacteroidales	unclassified Bacteroidales
7	Bacteria	FCB group	Bacteroidetes/Chlorobi group	Bacteroidetes	Bacteroidia	Bacteroidales	Prevotellaceae
7	Bacteria	FCB group	Bacteroidetes/Chlorobi group	Bacteroidetes	Bacteroidia	Bacteroidales	Prevotellaceae
7	Bacteria	FCB group	Bacteroidetes/Chlorobi group	Bacteroidetes	Bacteroidia	Bacteroidales	Prevotellaceae
7	Bacteria	FCB group	Bacteroidetes/Chlorobi group	Bacteroidetes	Bacteroidia	Bacteroidales	Prevotellaceae
7	Bacteria	FCB group	Bacteroidetes/Chlorobi group	Bacteroidetes	Bacteroidia	Bacteroidales	Porphyromonadaceae
7	Bacteria	FCB group	Bacteroidetes/Chlorobi group	Bacteroidetes	Bacteroidia	Bacteroidales	Porphyromonadaceae
7	Bacteria	FCB group	Bacteroidetes/Chlorobi group	Bacteroidetes	Bacteroidia	Bacteroidales	Bacteroidaceae
7	Bacteria	FCB group	Bacteroidetes/Chlorobi group	Bacteroidetes	Bacteroidia	Bacteroidales	Bacteroidaceae
7	Bacteria	FCB group	Bacteroidetes/Chlorobi group	Bacteroidetes	Bacteroidia	Bacteroidales	Bacteroidaceae
7	Bacteria	Elusimicrobia	Endomicrobia	Endomicrobiales	Endomicrobiaceae	Endomicrobium	Endomicrobium proavitum
7	Bacteria	Elusimicrobia	Elusimicrobia	Elusimicrobiales	Elusimicrobiaceae	Elusimicrobium	Elusimicrobium minutum
7	Bacteria	Acidobacteria	Acidobacteriia	Acidobacteriales	Acidobacteriaceae	unclassified Acidobacteriaceae	Acidobacteriaceae bacterium KBS 83
7	Bacteria	Acidobacteria	Acidobacteriia	Acidobacteriales	Acidobacteriaceae	Terriglobus	Terriglobus roseus
7	Bacteria	Acidobacteria	Acidobacteriia	Acidobacteriales	Acidobacteriaceae	Terracidiphilus	Terracidiphilus gabretensis
7	Bacteria	Acidobacteria	Acidobacteriia	Acidobacteriales	Acidobacteriaceae	Granulicella	Granulicella tundricola

7	Archaea	environmental samples	uncultured archaeon				
7	Archaea	TACK group	Thaumarchaeota	Nitrosopumilales	Nitrosopumilaceae	Nitrosopumilus	Candidatus Nitrosopumilus salaria
6	Eukaryota	Viridiplantae	Chlorophyta	prasinophytes	Mamiellophyceae	Mamiellales	Bathycoccaceae
6	Eukaryota	Viridiplantae	Chlorophyta	Chlorophyceae	Chlamydomonadales	Volvocaceae	
6	Eukaryota	Stramenopiles	Oomycetes	Peronosporales	Phytophthora	Phytophthora sojae	
6	Eukaryota	Rhodophyta	Bangiophyceae	Cyanidiales	Cyanidiaceae	Galdieria	Galdieria sulphuraria
6	Eukaryota	Rhizaria	Cercozoa	Cercomonadida	Cercomonadidae	Paracercomonas	Paracercomonas marina
6	Bacteria	unclassified Bacteria	unclassified Bacteria (miscellaneous)	bacterium SCGC AG-212-C10			
6	Bacteria	unclassified Bacteria	Bacteria candidate phyla	candidate division WOR-1	candidate division WOR_1 bacterium DG_54_3		
6	Bacteria	unclassified Bacteria	Bacteria candidate phyla	Patescibacteria group	Parcubacteria group	Candidatus Magasanikbacteria	
6	Bacteria	unclassified Bacteria	Bacteria candidate phyla	Patescibacteria group	Parcubacteria group	Candidatus Kaiserbacteria	
6	Bacteria	unclassified Bacteria	Bacteria candidate phyla	Patescibacteria group			
6	Bacteria	unclassified Bacteria	Bacteria candidate phyla	Candidatus Schekmanbacteria	Candidatus Schekmanbacteria bacterium RBG_13_48_7		
6	Bacteria	unclassified Bacteria	Bacteria candidate phyla	Candidatus Schekmanbacteria			
6	Bacteria	unclassified Bacteria	Bacteria candidate phyla	Candidatus Rokubacteria	Candidatus Rokubacteria bacterium 13_1_20CM_4_68_9		
6	Bacteria	unclassified Bacteria	Bacteria candidate phyla	Candidatus Doudnabacteria			
6	Bacteria	unclassified Bacteria	Bacteria candidate phyla	Candidatus Aminicenantes	unclassified Aminicenantes		
6	Bacteria	Terrabacteria group	Firmicutes	Tissierellia	unclassified Tissierellia	[Clostridium] ultunense	
6	Bacteria	Terrabacteria group	Firmicutes	Negativicutes	Veillonellales	Veillonellaceae	Megasphaera
6	Bacteria	Terrabacteria group	Firmicutes	Negativicutes	Veillonellales	Veillonellaceae	
6	Bacteria	Terrabacteria group	Firmicutes	Negativicutes	Selenomonadales	Sporomusaceae	Dendrosporobacter
6	Bacteria	Terrabacteria group	Firmicutes	Negativicutes	Acidaminococcales	Acidaminococcaceae	Succinispira
6	Bacteria	Terrabacteria group	Firmicutes	Clostridia	Thermoanaerobacterales	Thermoanaerobacteraceae	Thermanaeromonas
6	Bacteria	Terrabacteria group	Firmicutes	Clostridia	Halanaerobiales	Halobacteroidaceae	Selenihalanaerobacter
6	Bacteria	Terrabacteria group	Firmicutes	Clostridia	Halanaerobiales	Halanaerobiaceae	Halothermothrix
6	Bacteria	Terrabacteria group	Firmicutes	Clostridia	Halanaerobiales	Halanaerobiaceae	Halanaerobium
6	Bacteria	Terrabacteria group	Firmicutes	Clostridia	Clostridiales	unclassified Clostridiales	unclassified Clostridiales (miscellaneous)
6	Bacteria	Terrabacteria group	Firmicutes	Clostridia	Clostridiales	unclassified Clostridiales	Pseudoflavonifractor
6	Bacteria	Terrabacteria group	Firmicutes	Clostridia	Clostridiales	Syntrophomonadaceae	Syntrophothermus
6	Bacteria	Terrabacteria group	Firmicutes	Clostridia	Clostridiales	Syntrophomonadaceae	Syntrophomonas
6	Bacteria	Terrabacteria group	Firmicutes	Clostridia	Clostridiales	Ruminococcaceae	unclassified Ruminococcaceae
6	Bacteria	Terrabacteria group	Firmicutes	Clostridia	Clostridiales	Ruminococcaceae	Ruminococcus
6	Bacteria	Terrabacteria group	Firmicutes	Clostridia	Clostridiales	Ruminococcaceae	Ruminococcus
6	Bacteria	Terrabacteria group	Firmicutes	Clostridia	Clostridiales	Ruminococcaceae	Ruminiclostridium
6	Bacteria	Terrabacteria group	Firmicutes	Clostridia	Clostridiales	Peptococcaceae	unclassified Peptococcaceae
6	Bacteria	Terrabacteria group	Firmicutes	Clostridia	Clostridiales	Peptococcaceae	Desulfotomaculum
6	Bacteria	Terrabacteria group	Firmicutes	Clostridia	Clostridiales	Peptococcaceae	Desulfosporosinus
6	Bacteria	Terrabacteria group	Firmicutes	Clostridia	Clostridiales	Peptococcaceae	Desulfosporosinus
6	Bacteria	Terrabacteria group	Firmicutes	Clostridia	Clostridiales	Lachnospiraceae	Stomatobaculum
6	Bacteria	Terrabacteria group	Firmicutes	Clostridia	Clostridiales	Lachnospiraceae	Roseburia
6	Bacteria	Terrabacteria group	Firmicutes	Clostridia	Clostridiales	Lachnospiraceae	Oribacterium
6	Bacteria	Terrabacteria group	Firmicutes	Clostridia	Clostridiales	Lachnospiraceae	Dorea
6	Bacteria	Terrabacteria group	Firmicutes	Clostridia	Clostridiales	Lachnospiraceae	Butyrivibrio
6	Bacteria	Terrabacteria group	Firmicutes	Clostridia	Clostridiales	Lachnospiraceae	Blautia
6	Bacteria	Terrabacteria group	Firmicutes	Clostridia	Clostridiales	Lachnospiraceae	Blautia
6	Bacteria	Terrabacteria group	Firmicutes	Clostridia	Clostridiales	Lachnospiraceae	Bariatricus
6	Bacteria	Terrabacteria group	Firmicutes	Clostridia	Clostridiales	Eubacteriaceae	Anaerofustis
6	Bacteria	Terrabacteria group	Firmicutes	Clostridia	Clostridiales	Clostridiaceae	Thermotalea
6	Bacteria	Terrabacteria group	Firmicutes	Clostridia	Clostridiales	Clostridiaceae	Clostridium
6	Bacteria	Terrabacteria group	Firmicutes	Clostridia	Clostridiales	Clostridiaceae	Clostridium
6	Bacteria	Terrabacteria group	Firmicutes	Clostridia	Clostridiales	Clostridiaceae	Clostridium
6	Bacteria	Terrabacteria group	Firmicutes	Clostridia	Clostridiales	Clostridiaceae	Clostridium
6	Bacteria	Terrabacteria group	Firmicutes	Clostridia	Clostridiales	Clostridiaceae	Clostridium
6	Bacteria	Terrabacteria group	Firmicutes	Clostridia	Clostridiales	Clostridiaceae	Clostridium
6	Bacteria	Terrabacteria group	Firmicutes	Clostridia	Clostridiales	Clostridiaceae	Clostridium
6	Bacteria	Terrabacteria group	Firmicutes	Clostridia	Clostridiales	Clostridiaceae	Caminicella
6	Bacteria	Terrabacteria group	Firmicutes	Bacilli	Lactobacillales	Streptococcaceae	Lactococcus
6	Bacteria	Terrabacteria group	Firmicutes	Bacilli	Lactobacillales	Enterococcaceae	Enterococcus
6	Bacteria	Terrabacteria group	Firmicutes	Bacilli	Bacillales	Paenibacillaceae	Paenibacillus
6	Bacteria	Terrabacteria group	Firmicutes	Bacilli	Bacillales	Paenibacillaceae	Paenibacillus
6	Bacteria	Terrabacteria group	Firmicutes	Bacilli	Bacillales	Paenibacillaceae	Brevibacillus
6	Bacteria	Terrabacteria group	Firmicutes	Bacilli	Bacillales	Paenibacillaceae	Brevibacillus
6	Bacteria	Terrabacteria group	Firmicutes	Bacilli	Bacillales	Paenibacillaceae	Aneurinibacillus group
6	Bacteria	Terrabacteria group	Firmicutes	Bacilli	Bacillales	Bacillaceae	Bacillus
6	Bacteria	Terrabacteria group	Firmicutes	Bacilli	Bacillales	Alicyclobacillaceae	Alicyclobacillus
6	Bacteria	Terrabacteria group	Deinococcus-Thermus	Deinococci	Thermales	Thermaceae	Thermus
6	Bacteria	Terrabacteria group	Deinococcus-Thermus	Deinococci	Thermales	Thermaceae	Thermus
6	Bacteria	Terrabacteria group	Deinococcus-Thermus	Deinococci	Thermales	Thermaceae	Meiothermus
6	Bacteria	Terrabacteria group	Deinococcus-Thermus	Deinococci	Deinococcales	Deinococcaceae	Deinococcus
6	Bacteria	Terrabacteria group	Cyanobacteria/Melainabacteria group	Cyanobacteria	Oscillatoriophycideae	Chroococcales	Microcystaceae
6	Bacteria	Terrabacteria group	Cyanobacteria/Melainabacteria group	Cyanobacteria	Nostocales	Tolypothrichaceae	Tolypothrix
6	Bacteria	Terrabacteria group	Chloroflexi	unclassified Chloroflexi	unclassified Chloroflexi (miscellaneous)	Chloroflexi bacterium RBG_19FT_COMBO_62_14	
6	Bacteria	Terrabacteria group	Chloroflexi	unclassified Chloroflexi	unclassified Chloroflexi (miscellaneous)	Chloroflexi bacterium RBG_16_69_14	
6	Bacteria	Terrabacteria group	Chloroflexi	Thermomicrobia	Sphaerobacteridae	Sphaerobacterales	Sphaerobacterineae
6	Bacteria	Terrabacteria group	Chloroflexi	Ktedonobacteria	Ktedonobacterales	Ktedonobacteraceae	Ktedonobacter
6	Bacteria	Terrabacteria group	Chloroflexi	Chloroflexia	Chloroflexales	Roseiflexineae	Roseiflexaceae
6	Bacteria	Terrabacteria group	Chloroflexi	Anaerolineae	unclassified Anaerolineae	Anaerolineae bacterium CG2_30_57_67	
6	Bacteria	Terrabacteria group	Actinobacteria	unclassified Actinobacteria	Actinobacteria bacterium RBG_16_70_17		
6	Bacteria	Terrabacteria group	Actinobacteria	unclassified Actinobacteria	Actinobacteria bacterium RBG_16_68_12		
6	Bacteria	Terrabacteria group	Actinobacteria	unclassified Actinobacteria	Actinobacteria bacterium 13_1_20CM_3_71_11		
6	Bacteria	Terrabacteria group	Actinobacteria	Thermoleophilia	Solirubrobacterales	unclassified Solirubrobacterales	Solirubrobacterales bacterium 67-14
6	Bacteria	Terrabacteria group	Actinobacteria	Actinobacteria	Streptosporangiales	Thermomonosporaceae	Thermomonospora
6	Bacteria	Terrabacteria group	Actinobacteria	Actinobacteria	Streptomycetales	Streptomycetaceae	Streptomyces
6	Bacteria	Terrabacteria group	Actinobacteria	Actinobacteria	Streptomycetales	Streptomycetaceae	Streptacidiphilus
6	Bacteria	Terrabacteria group	Actinobacteria	Actinobacteria	Pseudonocardiales	Pseudonocardiaceae	Pseudonocardia
6	Bacteria	Terrabacteria group	Actinobacteria	Actinobacteria	Propionibacteriales	Nocardioidaceae	Nocardioides
6	Bacteria	Terrabacteria group	Actinobacteria	Actinobacteria	Propionibacteriales	Nocardioidaceae	Nocardioides
6	Bacteria	Terrabacteria group	Actinobacteria	Actinobacteria	Propionibacteriales	Nocardioidaceae	Nocardioides
6	Bacteria	Terrabacteria group	Actinobacteria	Actinobacteria	Propionibacteriales	Nocardioidaceae	Nocardioides
6	Bacteria	Terrabacteria group	Actinobacteria	Actinobacteria	Propionibacteriales	Nocardioidaceae	Kribbella
6	Bacteria	Terrabacteria group	Actinobacteria	Actinobacteria	Propionibacteriales	Nocardioidaceae	Aeromicrobium
6	Bacteria	Terrabacteria group	Actinobacteria	Actinobacteria	Micrococcales	Micrococcaceae	Kocuria
6	Bacteria	Terrabacteria group	Actinobacteria	Actinobacteria	Micrococcales	Intrasporangiaceae	
6	Bacteria	Terrabacteria group	Actinobacteria	Actinobacteria	Glycomycetales	Glycomycetaceae	Stackebrandtia
6	Bacteria	Terrabacteria group	Actinobacteria	Actinobacteria	Frankiales	Frankiaceae	Frankia
6	Bacteria	Terrabacteria group	Actinobacteria	Actinobacteria	Corynebacteriales	Nocardiaceae	Smaragdicoccus
6	Bacteria	Terrabacteria group	Actinobacteria	Actinobacteria	Corynebacteriales	Mycobacteriaceae	Mycobacterium
6	Bacteria	Terrabacteria group	Actinobacteria	Actinobacteria	Corynebacteriales	Mycobacteriaceae	Mycobacterium
6	Bacteria	Synergistetes	Synergistia	Synergistales	Synergistaceae	Jonquetella	
6	Bacteria	Spirochaetes	unclassified Spirochaetes	Spirochaetes bacterium RIFOXYC1_FULL_54_7			
6	Bacteria	Spirochaetes	Spirochaetia	Spirochaetales	Spirochaetaceae	Treponema	Treponema socranskii
6	Bacteria	Spirochaetes	Spirochaetia	Spirochaetales	Spirochaetaceae	Sphaerochaeta	Sphaerochaeta coccoides
6	Bacteria	Spirochaetes	Spirochaetia	Spirochaetales	Spirochaetaceae		
6	Bacteria	Proteobacteria	delta/epsilon subdivisions	Epsilonproteobacteria	Campylobacterales	Campylobacteraceae	Arcobacter
6	Bacteria	Proteobacteria	delta/epsilon subdivisions	Epsilonproteobacteria	Campylobacterales	Campylobacteraceae	Arcobacter
6	Bacteria	Proteobacteria	delta/epsilon subdivisions	Deltaproteobacteria	unclassified Deltaproteobacteria	unclassified Deltaproteobacteria (miscellaneous)	delta proteobacterium PSCGC 5451
6	Bacteria	Proteobacteria	delta/epsilon subdivisions	Deltaproteobacteria	unclassified Deltaproteobacteria	unclassified Deltaproteobacteria (miscellaneous)	delta proteobacterium ML8_F1
6	Bacteria	Proteobacteria	delta/epsilon subdivisions	Deltaproteobacteria	unclassified Deltaproteobacteria	unclassified Deltaproteobacteria (miscellaneous)	Deltaproteobacteria bacterium RIFOXYA12_FULL_61_11
6	Bacteria	Proteobacteria	delta/epsilon subdivisions	Deltaproteobacteria	unclassified Deltaproteobacteria	unclassified Deltaproteobacteria (miscellaneous)	Deltaproteobacteria bacterium RIFCSPLOWO2_02_FULL_53_8
6	Bacteria	Proteobacteria	delta/epsilon subdivisions	Deltaproteobacteria	unclassified Deltaproteobacteria	unclassified Deltaproteobacteria (miscellaneous)	Deltaproteobacteria bacterium RIFCSPLOWO2_02_44_9
6	Bacteria	Proteobacteria	delta/epsilon subdivisions	Deltaproteobacteria	unclassified Deltaproteobacteria	unclassified Deltaproteobacteria (miscellaneous)	Deltaproteobacteria bacterium RBG_16_49_23
6	Bacteria	Proteobacteria	delta/epsilon subdivisions	Deltaproteobacteria	unclassified Deltaproteobacteria	unclassified Deltaproteobacteria (miscellaneous)	Deltaproteobacteria bacterium GWA2_54_12
6	Bacteria	Proteobacteria	delta/epsilon subdivisions	Deltaproteobacteria	Syntrophobacterales	Syntrophaceae	unclassified Syntrophaceae
6	Bacteria	Proteobacteria	delta/epsilon subdivisions	Deltaproteobacteria	Myxococcales	Sorangiineae	Polyangiaceae
6	Bacteria	Proteobacteria	delta/epsilon subdivisions	Deltaproteobacteria	Myxococcales	Cystobacterineae	Myxococcaceae
6	Bacteria	Proteobacteria	delta/epsilon subdivisions	Deltaproteobacteria	Myxococcales	Cystobacterineae	Myxococcaceae
6	Bacteria	Proteobacteria	delta/epsilon subdivisions	Deltaproteobacteria	Desulfuromonadales	unclassified Desulfuromonadales	Desulfuromonadales bacterium C00003093
6	Bacteria	Proteobacteria	delta/epsilon subdivisions	Deltaproteobacteria	Desulfuromonadales	Geobacteraceae	unclassified Geobacteraceae
6	Bacteria	Proteobacteria	delta/epsilon subdivisions	Deltaproteobacteria	Desulfuromonadales	Geobacteraceae	Geobacter
6	Bacteria	Proteobacteria	delta/epsilon subdivisions	Deltaproteobacteria	Desulfuromonadales	Geobacteraceae	Geobacter
6	Bacteria	Proteobacteria	delta/epsilon subdivisions	Deltaproteobacteria	Desulfuromonadales	Geobacteraceae	Geobacter
6	Bacteria	Proteobacteria	delta/epsilon subdivisions	Deltaproteobacteria	Desulfuromonadales	Desulfuromonadaceae	unclassified Desulfuromonadaceae
6	Bacteria	Proteobacteria	delta/epsilon subdivisions	Deltaproteobacteria	Desulfuromonadales	Desulfuromonadaceae	Pelobacter
6	Bacteria	Proteobacteria	delta/epsilon subdivisions	Deltaproteobacteria	Desulfuromonadales	Desulfuromonadaceae	Desulfuromonas
6	Bacteria	Proteobacteria	delta/epsilon subdivisions	Deltaproteobacteria	Desulfuromonadales	Desulfuromonadaceae	Desulfuromonas
6	Bacteria	Proteobacteria	delta/epsilon subdivisions	Deltaproteobacteria	Desulfovibrionales	Desulfovibrionaceae	Lawsonia
6	Bacteria	Proteobacteria	delta/epsilon subdivisions	Deltaproteobacteria	Desulfobacterales	unclassified Desulfobacterales	Desulfobacterales bacterium SG8_35_2
6	Bacteria	Proteobacteria	delta/epsilon subdivisions	Deltaproteobacteria	Desulfobacterales	Desulfobulbaceae	Desulfurivibrio
6	Bacteria	Proteobacteria	delta/epsilon subdivisions	Deltaproteobacteria	Desulfobacterales	Desulfobacteraceae	Desulfosarcina
6	Bacteria	Proteobacteria	delta/epsilon subdivisions	Deltaproteobacteria	Desulfobacterales	Desulfobacteraceae	Desulfococcus
6	Bacteria	Proteobacteria	delta/epsilon subdivisions	Deltaproteobacteria	Desulfobacterales	Desulfobacteraceae	Desulfatibacillum
6	Bacteria	Proteobacteria	Gammaproteobacteria	unclassified Gammaproteobacteria	unclassified Gammaproteobacteria (miscellaneous)	gamma proteobacterium HIMB30	
6	Bacteria	Proteobacteria	Gammaproteobacteria	unclassified Gammaproteobacteria	unclassified Gammaproteobacteria (miscellaneous)	Gammaproteobacteria bacterium RIFCSPLOWO2_02_FULL_52_10	
6	Bacteria	Proteobacteria	Gammaproteobacteria	unclassified Gammaproteobacteria	unclassified Gammaproteobacteria (miscellaneous)	Gammaproteobacteria bacterium MFB021	
6	Bacteria	Proteobacteria	Gammaproteobacteria	unclassified Gammaproteobacteria	unclassified Gammaproteobacteria (miscellaneous)	Gammaproteobacteria bacterium 39-13	
6	Bacteria	Proteobacteria	Gammaproteobacteria	unclassified Gammaproteobacteria	Wohlfahrtiimonas	Wohlfahrtiimonas sp. 34C10-3-10	
6	Bacteria	Proteobacteria	Gammaproteobacteria	unclassified Gammaproteobacteria	Wohlfahrtiimonas	Wohlfahrtiimonas larvae	
6	Bacteria	Proteobacteria	Gammaproteobacteria	unclassified Gammaproteobacteria	Thiohalorhabdus	Thiohalorhabdus denitrificans	
6	Bacteria	Proteobacteria	Gammaproteobacteria	unclassified Gammaproteobacteria	SAR86 cluster		
6	Bacteria	Proteobacteria	Gammaproteobacteria	unclassified Gammaproteobacteria	Gallaecimonas	Gallaecimonas pentaromativorans	
6	Bacteria	Proteobacteria	Gammaproteobacteria	unclassified Gammaproteobacteria	Candidatus Thioglobus	Candidatus Thioglobus singularis	
6	Bacteria	Proteobacteria	Gammaproteobacteria	Xanthomonadales	Xanthomonadaceae	Xanthomonas	Xanthomonas phaseoli
6	Bacteria	Proteobacteria	Gammaproteobacteria	Xanthomonadales	Xanthomonadaceae	Stenotrophomonas	Stenotrophomonas panacihumi
6	Bacteria	Proteobacteria	Gammaproteobacteria	Xanthomonadales	Xanthomonadaceae	Pseudoxanthomonas	Pseudoxanthomonas sp. GM95
6	Bacteria	Proteobacteria	Gammaproteobacteria	Xanthomonadales	Xanthomonadaceae	Luteimonas	
6	Bacteria	Proteobacteria	Gammaproteobacteria	Xanthomonadales	Rhodanobacteraceae	Rhodanobacter	Rhodanobacter sp. OK091
6	Bacteria	Proteobacteria	Gammaproteobacteria	Xanthomonadales	Rhodanobacteraceae	Rhodanobacter	Rhodanobacter sp. C06
6	Bacteria	Proteobacteria	Gammaproteobacteria	Xanthomonadales	Rhodanobacteraceae	Rhodanobacter	Rhodanobacter sp. C01
6	Bacteria	Proteobacteria	Gammaproteobacteria	Xanthomonadales	Rhodanobacteraceae	Luteibacter	
6	Bacteria	Proteobacteria	Gammaproteobacteria	Vibrionales	Vibrionaceae	Vibrio	Vibrio anguillarum
6	Bacteria	Proteobacteria	Gammaproteobacteria	Thiotrichales	Thiotrichaceae	Beggiatoa	Beggiatoa alba
6	Bacteria	Proteobacteria	Gammaproteobacteria	Thiotrichales	Piscirickettsiaceae	Thiomicrospira	Thiomicrospira arctica
6	Bacteria	Proteobacteria	Gammaproteobacteria	Thiotrichales	Piscirickettsiaceae	Thioalkalimicrobium	Thioalkalimicrobium microaerophilum
6	Bacteria	Proteobacteria	Gammaproteobacteria	Thiotrichales	Piscirickettsiaceae	Methylophaga	Methylophaga lonarensis
6	Bacteria	Proteobacteria	Gammaproteobacteria	Thiotrichales	Piscirickettsiaceae	Methylophaga	Methylophaga aminisulfidivorans
6	Bacteria	Proteobacteria	Gammaproteobacteria	Pseudomonadales	Pseudomonadaceae	Pseudomonas	Pseudomonas sp. S-6-2
6	Bacteria	Proteobacteria	Gammaproteobacteria	Pseudomonadales	Pseudomonadaceae	Pseudomonas	Pseudomonas sp. CB1
6	Bacteria	Proteobacteria	Gammaproteobacteria	Pseudomonadales	Pseudomonadaceae	Pseudomonas	Pseudomonas salegens
6	Bacteria	Proteobacteria	Gammaproteobacteria	Pseudomonadales	Pseudomonadaceae	Pseudomonas	Pseudomonas pelagia
6	Bacteria	Proteobacteria	Gammaproteobacteria	Pseudomonadales	Pseudomonadaceae	Pseudomonas	Pseudomonas guineae
6	Bacteria	Proteobacteria	Gammaproteobacteria	Pseudomonadales	Pseudomonadaceae	Pseudomonas	Pseudomonas chlororaphis group
6	Bacteria	Proteobacteria	Gammaproteobacteria	Pseudomonadales	Pseudomonadaceae	Pseudomonas	Pseudomonas aeruginosa group
6	Bacteria	Proteobacteria	Gammaproteobacteria	Pseudomonadales	Pseudomonadaceae	Pseudomonas	Pseudomonas aeruginosa group
6	Bacteria	Proteobacteria	Gammaproteobacteria	Pseudomonadales	Pseudomonadaceae	Azotobacter group	Azotobacter
6	Bacteria	Proteobacteria	Gammaproteobacteria	Pseudomonadales	Moraxellaceae	Moraxella	Moraxella boevrei
6	Bacteria	Proteobacteria	Gammaproteobacteria	Pseudomonadales	Moraxellaceae	Acinetobacter	Acinetobacter calcoaceticus/baumannii complex
6	Bacteria	Proteobacteria	Gammaproteobacteria	Oceanospirillales	Oceanospirillaceae	Thalassolituus	Thalassolituus oleivorans
6	Bacteria	Proteobacteria	Gammaproteobacteria	Oceanospirillales	Oceanospirillaceae	Nitrincola	Nitrincola nitratireducens
6	Bacteria	Proteobacteria	Gammaproteobacteria	Oceanospirillales	Oceanospirillaceae	Marinobacterium	Marinobacterium jannaschii
6	Bacteria	Proteobacteria	Gammaproteobacteria	Oceanospirillales	Oceanospirillaceae	Marinobacterium	
6	Bacteria	Proteobacteria	Gammaproteobacteria	Oceanospirillales	Halomonadaceae	Halomonas	Halomonas sp. PR-M31
6	Bacteria	Proteobacteria	Gammaproteobacteria	Oceanospirillales	Halomonadaceae	Halomonas	Halomonas gudaonensis
6	Bacteria	Proteobacteria	Gammaproteobacteria	Oceanospirillales	Halomonadaceae	Halomonas	Halomonas cupida
6	Bacteria	Proteobacteria	Gammaproteobacteria	Oceanospirillales	Alcanivoracaceae	Marinicella	Marinicella sp. F2
6	Bacteria	Proteobacteria	Gammaproteobacteria	Oceanospirillales			
6	Bacteria	Proteobacteria	Gammaproteobacteria	Methylococcales	Methylococcaceae	Methylovulum	Methylovulum miyakonense
6	Bacteria	Proteobacteria	Gammaproteobacteria	Methylococcales	Methylococcaceae	Methylomarinum	Methylomarinum vadi
6	Bacteria	Proteobacteria	Gammaproteobacteria	Methylococcales	Methylococcaceae	Methylogaea	Methylogaea oryzae
6	Bacteria	Proteobacteria	Gammaproteobacteria	Legionellales	Legionellaceae	Legionella	Legionella shakespearei
6	Bacteria	Proteobacteria	Gammaproteobacteria	Legionellales	Legionellaceae	Legionella	Legionella massiliensis
6	Bacteria	Proteobacteria	Gammaproteobacteria	Legionellales	Legionellaceae		
6	Bacteria	Proteobacteria	Gammaproteobacteria	Legionellales	Coxiellaceae	Coxiella	Coxiella burnetii
6	Bacteria	Proteobacteria	Gammaproteobacteria	Enterobacterales	Pectobacteriaceae	Dickeya	Dickeya paradisiaca
6	Bacteria	Proteobacteria	Gammaproteobacteria	Enterobacterales	Enterobacteriaceae	Klebsiella	
6	Bacteria	Proteobacteria	Gammaproteobacteria	Enterobacterales	Enterobacteriaceae	Enterobacter	Enterobacter cloacae complex
6	Bacteria	Proteobacteria	Gammaproteobacteria	Chromatiales	Ectothiorhodospiraceae	Thioalkalivibrio	Thioalkalivibrio paradoxus
6	Bacteria	Proteobacteria	Gammaproteobacteria	Chromatiales	Chromatiaceae	Thiorhodococcus	Thiorhodococcus drewsii
6	Bacteria	Proteobacteria	Gammaproteobacteria	Chromatiales	Chromatiaceae	Rheinheimera	Rheinheimera texasensis
6	Bacteria	Proteobacteria	Gammaproteobacteria	Cellvibrionales	Spongiibacteraceae	Spongiibacter	Spongiibacter tropicus
6	Bacteria	Proteobacteria	Gammaproteobacteria	Cellvibrionales	Microbulbiferaceae	Microbulbifer	Microbulbifer agarilyticus
6	Bacteria	Proteobacteria	Gammaproteobacteria	Cellvibrionales	Cellvibrionaceae	Marinimicrobium	Marinimicrobium sp. LS-A18
6	Bacteria	Proteobacteria	Gammaproteobacteria	Cellvibrionales	Cellvibrionaceae	Marinimicrobium	Marinimicrobium agarilyticum
6	Bacteria	Proteobacteria	Gammaproteobacteria	Cellvibrionales	Cellvibrionaceae	Marinagarivorans	Marinagarivorans algicola
6	Bacteria	Proteobacteria	Gammaproteobacteria	Cellvibrionales	Cellvibrionaceae	Cellvibrio	Cellvibrio sp. PSBB023
6	Bacteria	Proteobacteria	Gammaproteobacteria	Cellvibrionales	Cellvibrionaceae	Cellvibrio	Cellvibrio sp. OA-2007
6	Bacteria	Proteobacteria	Gammaproteobacteria	Alteromonadales	Idiomarinaceae	unclassified Idiomarinaceae	Idiomarinaceae bacterium HL-53
6	Bacteria	Proteobacteria	Gammaproteobacteria	Alteromonadales	Alteromonadaceae	Marinobacter	Marinobacter segnicrescens
6	Bacteria	Proteobacteria	Gammaproteobacteria	Alteromonadales	Alteromonadaceae	Alteromonas	
6	Bacteria	Proteobacteria	Gammaproteobacteria	Alteromonadales	Alteromonadaceae	Aliiglaciecola	Aliiglaciecola lipolytica
6	Bacteria	Proteobacteria	Gammaproteobacteria	Aeromonadales	Succinivibrionaceae	Succinatimonas	Succinatimonas hippei
6	Bacteria	Proteobacteria	Gammaproteobacteria	Aeromonadales	Aeromonadaceae	Tolumonas	Tolumonas auensis
6	Bacteria	Proteobacteria	Candidatus Muproteobacteria	Candidatus Muproteobacteria bacterium RBG_16_65_34			
6	Bacteria	Proteobacteria	Betaproteobacteria	unclassified Betaproteobacteria	unclassified Betaproteobacteria (miscellaneous)	Betaproteobacteria bacterium RIFCSPLOWO2_02_FULL_65_24	
6	Bacteria	Proteobacteria	Betaproteobacteria	unclassified Betaproteobacteria	Candidatus Accumulibacter	Candidatus Accumulibacter sp. BA-94	
6	Bacteria	Proteobacteria	Betaproteobacteria	Rhodocyclales	Rhodocyclaceae	Thauera	Thauera sp. 27
6	Bacteria	Proteobacteria	Betaproteobacteria	Rhodocyclales	Rhodocyclaceae	Thauera	Thauera phenylacetica
6	Bacteria	Proteobacteria	Betaproteobacteria	Nitrosomonadales	Nitrosomonadaceae	Nitrosomonas	Nitrosomonas aestuarii
6	Bacteria	Proteobacteria	Betaproteobacteria	Neisseriales	Chromobacteriaceae	Chitinilyticum	
6	Bacteria	Proteobacteria	Betaproteobacteria	Neisseriales	Chromobacteriaceae	Aquitalea	Aquitalea magnusonii
6	Bacteria	Proteobacteria	Betaproteobacteria	Hydrogenophilales	Hydrogenophilaceae	Tepidiphilus	
6	Bacteria	Proteobacteria	Betaproteobacteria	Burkholderiales	unclassified Burkholderiales	Burkholderiales Genera incertae sedis	Leptothrix
6	Bacteria	Proteobacteria	Betaproteobacteria	Burkholderiales	Oxalobacteraceae	Janthinobacterium	Janthinobacterium sp. LM6
6	Bacteria	Proteobacteria	Betaproteobacteria	Burkholderiales	Oxalobacteraceae	Herbaspirillum	Herbaspirillum sp. GW103
6	Bacteria	Proteobacteria	Betaproteobacteria	Burkholderiales	Oxalobacteraceae	Herbaspirillum	Herbaspirillum lusitanum
6	Bacteria	Proteobacteria	Betaproteobacteria	Burkholderiales	Oxalobacteraceae	Duganella	Duganella sp. Leaf126
6	Bacteria	Proteobacteria	Betaproteobacteria	Burkholderiales	Oxalobacteraceae	Duganella	Duganella sp. CF458
6	Bacteria	Proteobacteria	Betaproteobacteria	Burkholderiales	Oxalobacteraceae	Duganella	Duganella phyllosphaerae
6	Bacteria	Proteobacteria	Betaproteobacteria	Burkholderiales	Comamonadaceae	Verminephrobacter	Verminephrobacter eiseniae
6	Bacteria	Proteobacteria	Betaproteobacteria	Burkholderiales	Comamonadaceae	Delftia	Delftia lacustris
6	Bacteria	Proteobacteria	Betaproteobacteria	Burkholderiales	Comamonadaceae	Alicycliphilus	Alicycliphilus sp. B1
6	Bacteria	Proteobacteria	Betaproteobacteria	Burkholderiales	Comamonadaceae	Acidovorax	Acidovorax sp. Root568
6	Bacteria	Proteobacteria	Betaproteobacteria	Burkholderiales	Comamonadaceae	Acidovorax	Acidovorax sp. Root402
6	Bacteria	Proteobacteria	Betaproteobacteria	Burkholderiales	Comamonadaceae	Acidovorax	Acidovorax ebreus
6	Bacteria	Proteobacteria	Betaproteobacteria	Burkholderiales	Burkholderiaceae	Polynucleobacter	Polynucleobacter wuianus
6	Bacteria	Proteobacteria	Betaproteobacteria	Burkholderiales	Burkholderiaceae	Paraburkholderia	Paraburkholderia tuberum
6	Bacteria	Proteobacteria	Betaproteobacteria	Burkholderiales	Burkholderiaceae	Paraburkholderia	Paraburkholderia susongensis
6	Bacteria	Proteobacteria	Betaproteobacteria	Burkholderiales	Burkholderiaceae	Paraburkholderia	Paraburkholderia sp. SOS3
6	Bacteria	Proteobacteria	Betaproteobacteria	Burkholderiales	Burkholderiaceae	Paraburkholderia	Paraburkholderia nodosa
6	Bacteria	Proteobacteria	Betaproteobacteria	Burkholderiales	Burkholderiaceae	Paraburkholderia	Paraburkholderia monticola
6	Bacteria	Proteobacteria	Betaproteobacteria	Burkholderiales	Burkholderiaceae	Paraburkholderia	Paraburkholderia megapolitana
6	Bacteria	Proteobacteria	Betaproteobacteria	Burkholderiales	Burkholderiaceae	Paraburkholderia	Paraburkholderia caribensis
6	Bacteria	Proteobacteria	Betaproteobacteria	Burkholderiales	Burkholderiaceae	Pandoraea	Pandoraea norimbergensis
6	Bacteria	Proteobacteria	Betaproteobacteria	Burkholderiales	Burkholderiaceae	Cupriavidus	Cupriavidus nantongensis
6	Bacteria	Proteobacteria	Betaproteobacteria	Burkholderiales	Burkholderiaceae	Burkholderia	Burkholderia sp. SRS-W-2-2016
6	Bacteria	Proteobacteria	Betaproteobacteria	Burkholderiales	Burkholderiaceae	Burkholderia	Burkholderia sp. H160
6	Bacteria	Proteobacteria	Betaproteobacteria	Burkholderiales	Burkholderiaceae	Burkholderia	Burkholderia hypogeia
6	Bacteria	Proteobacteria	Betaproteobacteria	Burkholderiales	Burkholderiaceae	Burkholderia	Burkholderia cepacia complex
6	Bacteria	Proteobacteria	Betaproteobacteria	Burkholderiales	Alcaligenaceae	Pelistega	
6	Bacteria	Proteobacteria	Betaproteobacteria	Burkholderiales	Alcaligenaceae	Paenalcaligenes	Paenalcaligenes hominis
6	Bacteria	Proteobacteria	Betaproteobacteria	Burkholderiales	Alcaligenaceae	Bordetella	Bordetella trematum
6	Bacteria	Proteobacteria	Betaproteobacteria	Burkholderiales	Alcaligenaceae	Bordetella	Bordetella bronchiseptica
6	Bacteria	Proteobacteria	Betaproteobacteria	Burkholderiales	Alcaligenaceae	Achromobacter	Achromobacter sp. Root83
6	Bacteria	Proteobacteria	Alphaproteobacteria	unclassified Alphaproteobacteria	unclassified Alphaproteobacteria (miscellaneous)	alpha proteobacterium RS24	
6	Bacteria	Proteobacteria	Alphaproteobacteria	unclassified Alphaproteobacteria	unclassified Alphaproteobacteria (miscellaneous)	Alphaproteobacteria bacterium RBG_16_64_48	
6	Bacteria	Proteobacteria	Alphaproteobacteria	unclassified Alphaproteobacteria	unclassified Alphaproteobacteria (miscellaneous)	Alphaproteobacteria bacterium AO1-B	
6	Bacteria	Proteobacteria	Alphaproteobacteria	unclassified Alphaproteobacteria	SAR116 cluster	unclassified SAR116 cluster	SAR116 cluster alpha proteobacterium HIMB100
6	Bacteria	Proteobacteria	Alphaproteobacteria	Sphingomonadales	Sphingomonadaceae	Sphingopyxis	Sphingopyxis sp. SCN 67-31
6	Bacteria	Proteobacteria	Alphaproteobacteria	Sphingomonadales	Sphingomonadaceae	Sphingomonas	Sphingomonas sp. Leaf10
6	Bacteria	Proteobacteria	Alphaproteobacteria	Sphingomonadales	Sphingomonadaceae	Sphingomonas	Sphingomonas pituitosa
6	Bacteria	Proteobacteria	Alphaproteobacteria	Sphingomonadales	Sphingomonadaceae	Novosphingobium	Novosphingobium sp. SCN 63-17
6	Bacteria	Proteobacteria	Alphaproteobacteria	Sphingomonadales	Erythrobacteraceae	Erythrobacter	Erythrobacter sp. SCN 68-10
6	Bacteria	Proteobacteria	Alphaproteobacteria	Rickettsiales	unclassified Rickettsiales	unclassified Rickettsiales (miscellaneous)	Rickettsiales bacterium Ac37b
6	Bacteria	Proteobacteria	Alphaproteobacteria	Rickettsiales	Rickettsiaceae	Rickettsieae	Rickettsia
6	Bacteria	Proteobacteria	Alphaproteobacteria	Rhodospirillales	Rhodospirillaceae	Thalassospira	Thalassospira sp. TSL5-1
6	Bacteria	Proteobacteria	Alphaproteobacteria	Rhodospirillales	Rhodospirillaceae	Rhodovibrio	Rhodovibrio salinarum
6	Bacteria	Proteobacteria	Alphaproteobacteria	Rhodospirillales	Rhodospirillaceae	Limimonas	Limimonas halophila
6	Bacteria	Proteobacteria	Alphaproteobacteria	Rhodospirillales	Rhodospirillaceae	Fodinicurvata	Fodinicurvata fenggangensis
6	Bacteria	Proteobacteria	Alphaproteobacteria	Rhodospirillales	Rhodospirillaceae	Azospirillum	Azospirillum thiophilum
6	Bacteria	Proteobacteria	Alphaproteobacteria	Rhodospirillales	Rhodospirillaceae	Azospirillum	Azospirillum sp. B506
6	Bacteria	Proteobacteria	Alphaproteobacteria	Rhodospirillales	Rhodospirillaceae	Azospirillum	Azospirillum oryzae
6	Bacteria	Proteobacteria	Alphaproteobacteria	Rhodospirillales	Acetobacteraceae	Kozakia	Kozakia baliensis
6	Bacteria	Proteobacteria	Alphaproteobacteria	Rhodospirillales	Acetobacteraceae	Gluconobacter	
6	Bacteria	Proteobacteria	Alphaproteobacteria	Rhodospirillales	Acetobacteraceae	Acetobacter	Acetobacter nitrogenifigens
6	Bacteria	Proteobacteria	Alphaproteobacteria	Rhodobacterales	Rhodobacteraceae	Xuhuaishuia	Xuhuaishuia manganoxidans
6	Bacteria	Proteobacteria	Alphaproteobacteria	Rhodobacterales	Rhodobacteraceae	Sulfitobacter	Sulfitobacter sp. HI0054
6	Bacteria	Proteobacteria	Alphaproteobacteria	Rhodobacterales	Rhodobacteraceae	Rhodobacter	Rhodobacter sp. BACL10 MAG-120419-bin15
6	Bacteria	Proteobacteria	Alphaproteobacteria	Rhodobacterales	Rhodobacteraceae	Phaeobacter	Phaeobacter sp. 11ANDIMAR09
6	Bacteria	Proteobacteria	Alphaproteobacteria	Rhodobacterales	Rhodobacteraceae	Paracoccus	Paracoccus sp. TRP
6	Bacteria	Proteobacteria	Alphaproteobacteria	Rhodobacterales	Rhodobacteraceae	Pannonibacter	Pannonibacter indicus
6	Bacteria	Proteobacteria	Alphaproteobacteria	Rhodobacterales	Rhodobacteraceae	Oceanibulbus	Oceanibulbus indolifex
6	Bacteria	Proteobacteria	Alphaproteobacteria	Rhodobacterales	Rhodobacteraceae	Leisingera	Leisingera sp. ANG-M1
6	Bacteria	Proteobacteria	Alphaproteobacteria	Rhodobacterales	Rhodobacteraceae	Labrenzia	Labrenzia alexandrii
6	Bacteria	Proteobacteria	Alphaproteobacteria	Rhodobacterales	Rhodobacteraceae	Ketogulonicigenium	
6	Bacteria	Proteobacteria	Alphaproteobacteria	Rhodobacterales	Rhodobacteraceae	Confluentimicrobium	Confluentimicrobium sp. EMB200-NS6
6	Bacteria	Proteobacteria	Alphaproteobacteria	Rhodobacterales	Rhodobacteraceae	Citreicella	Citreicella thiooxidans
6	Bacteria	Proteobacteria	Alphaproteobacteria	Rhodobacterales	Rhodobacteraceae	Citreicella	Citreicella sp. 357
6	Bacteria	Proteobacteria	Alphaproteobacteria	Rhodobacterales	Hyphomonadaceae	Hyphomonas	Hyphomonas adhaerens
6	Bacteria	Proteobacteria	Alphaproteobacteria	Rhodobacterales	Hyphomonadaceae	Henriciella	Henriciella marina
6	Bacteria	Proteobacteria	Alphaproteobacteria	Rhizobiales	unclassified Rhizobiales	unclassified Rhizobiales (miscellaneous)	Rhizobiales bacterium 62-47
6	Bacteria	Proteobacteria	Alphaproteobacteria	Rhizobiales	unclassified Rhizobiales	Candidatus Tokpelaia	Candidatus Tokpelaia hoelldoblerii
6	Bacteria	Proteobacteria	Alphaproteobacteria	Rhizobiales	Xanthobacteraceae		
6	Bacteria	Proteobacteria	Alphaproteobacteria	Rhizobiales	Rhizobiaceae	Sinorhizobium/Ensifer group	Sinorhizobium
6	Bacteria	Proteobacteria	Alphaproteobacteria	Rhizobiales	Rhizobiaceae	Rhizobium/Agrobacterium group	Rhizobium
6	Bacteria	Proteobacteria	Alphaproteobacteria	Rhizobiales	Rhizobiaceae	Rhizobium/Agrobacterium group	Rhizobium
6	Bacteria	Proteobacteria	Alphaproteobacteria	Rhizobiales	Rhizobiaceae	Rhizobium/Agrobacterium group	Rhizobium
6	Bacteria	Proteobacteria	Alphaproteobacteria	Rhizobiales	Rhizobiaceae	Rhizobium/Agrobacterium group	Rhizobium
6	Bacteria	Proteobacteria	Alphaproteobacteria	Rhizobiales	Rhizobiaceae	Rhizobium/Agrobacterium group	Rhizobium
6	Bacteria	Proteobacteria	Alphaproteobacteria	Rhizobiales	Rhizobiaceae	Rhizobium/Agrobacterium group	Rhizobium
6	Bacteria	Proteobacteria	Alphaproteobacteria	Rhizobiales	Rhizobiaceae	Rhizobium/Agrobacterium group	Rhizobium
6	Bacteria	Proteobacteria	Alphaproteobacteria	Rhizobiales	Rhizobiaceae	Rhizobium/Agrobacterium group	Rhizobium
6	Bacteria	Proteobacteria	Alphaproteobacteria	Rhizobiales	Rhizobiaceae	Rhizobium/Agrobacterium group	Rhizobium
6	Bacteria	Proteobacteria	Alphaproteobacteria	Rhizobiales	Rhizobiaceae	Rhizobium/Agrobacterium group	Rhizobium
6	Bacteria	Proteobacteria	Alphaproteobacteria	Rhizobiales	Rhizobiaceae	Rhizobium/Agrobacterium group	Agrobacterium
6	Bacteria	Proteobacteria	Alphaproteobacteria	Rhizobiales	Rhizobiaceae	Rhizobium/Agrobacterium group	Agrobacterium
6	Bacteria	Proteobacteria	Alphaproteobacteria	Rhizobiales	Phyllobacteriaceae	Mesorhizobium	Mesorhizobium sp. WSM3224
6	Bacteria	Proteobacteria	Alphaproteobacteria	Rhizobiales	Phyllobacteriaceae	Mesorhizobium	Mesorhizobium sp. SCN 65-20
6	Bacteria	Proteobacteria	Alphaproteobacteria	Rhizobiales	Phyllobacteriaceae	Mesorhizobium	Mesorhizobium sp. L48C026A00
6	Bacteria	Proteobacteria	Alphaproteobacteria	Rhizobiales	Methylocystaceae	Terasakiella	Terasakiella sp. PR1
6	Bacteria	Proteobacteria	Alphaproteobacteria	Rhizobiales	Methylobacteriaceae	Methylobacterium	Methylobacterium sp. Leaf104
6	Bacteria	Proteobacteria	Alphaproteobacteria	Rhizobiales	Methylobacteriaceae	Methylobacterium	Methylobacterium salsuginis
6	Bacteria	Proteobacteria	Alphaproteobacteria	Rhizobiales	Hyphomicrobiaceae	Devosia	Devosia sp. A16
6	Bacteria	Proteobacteria	Alphaproteobacteria	Rhizobiales	Brucellaceae	Brucella	
6	Bacteria	Proteobacteria	Alphaproteobacteria	Rhizobiales	Bradyrhizobiaceae	Bradyrhizobium	Bradyrhizobium sp. SEMIA 6399
6	Bacteria	Proteobacteria	Alphaproteobacteria	Rhizobiales	Bradyrhizobiaceae	Bradyrhizobium	Bradyrhizobium sp. NAS96.2
6	Bacteria	Proteobacteria	Alphaproteobacteria	Rhizobiales	Aurantimonadaceae	Aureimonas	Aureimonas sp. Leaf324
6	Bacteria	Proteobacteria	Alphaproteobacteria	Caulobacterales	Caulobacteraceae	Caulobacter	Caulobacter sp. UNC358MFTsu5.1
6	Bacteria	Proteobacteria	Alphaproteobacteria	Caulobacterales	Caulobacteraceae	Caulobacter	Caulobacter sp. CCH9-E1
6	Bacteria	Proteobacteria	Alphaproteobacteria	Caulobacterales	Caulobacteraceae	Caulobacter	Caulobacter segnis
6	Bacteria	Proteobacteria	Alphaproteobacteria	Caulobacterales	Caulobacteraceae	Brevundimonas	Brevundimonas viscosa
6	Bacteria	Proteobacteria	Alphaproteobacteria	Caulobacterales	Caulobacteraceae	Brevundimonas	Brevundimonas bacteroides
6	Bacteria	Proteobacteria	Acidithiobacillia	Acidithiobacillales	unclassified Acidithiobacillales	Acidithiobacillales bacterium SG8_45	
6	Bacteria	PVC group	Verrucomicrobia	unclassified Verrucomicrobia	unclassified Verrucomicrobia (miscellaneous)	Verrucomicrobia bacterium GWF2_51_19	
6	Bacteria	PVC group	Verrucomicrobia	Verrucomicrobiae	Verrucomicrobiales	Verrucomicrobiaceae	Verrucomicrobium
6	Bacteria	PVC group	Planctomycetes	unclassified Planctomycetes	Planctomycetes bacterium RBG_19FT_COMBO_48_8		
6	Bacteria	PVC group	Planctomycetes	unclassified Planctomycetes	Planctomycetes bacterium RBG_16_59_8		
6	Bacteria	PVC group	Planctomycetes	unclassified Planctomycetes	Planctomycetes bacterium RBG_13_60_9		
6	Bacteria	PVC group	Planctomycetes	Planctomycetia	Planctomycetales	unclassified Planctomycetales	unclassified Planctomycetales (miscellaneous)
6	Bacteria	PVC group	Planctomycetes	Phycisphaerae	unclassified Phycisphaerae	Phycisphaerae bacterium L21-RPul-D3	
6	Bacteria	PVC group	Chlamydiae	Chlamydiia	Parachlamydiales	Criblamydiaceae	Estrella
6	Bacteria	PVC group	Chlamydiae	Chlamydiia	Parachlamydiales	Criblamydiaceae	Criblamydia
6	Bacteria	Nitrospirae	unclassified Nitrospirae	Nitrospirae bacterium HCH-1			
6	Bacteria	Nitrospirae	Nitrospira	Nitrospirales	Nitrospiraceae	Leptospirillum	Leptospirillum sp. Group III
6	Bacteria	Fusobacteria	Fusobacteriia	Fusobacteriales	Leptotrichiaceae	Leptotrichia	
6	Bacteria	Fusobacteria	Fusobacteriia	Fusobacteriales	Fusobacteriaceae	Ilyobacter	Ilyobacter polytropus
6	Bacteria	FCB group	candidate division Zixibacteria	candidate division Zixibacteria bacterium RBG_16_53_22			
6	Bacteria	FCB group	Gemmatimonadetes	Gemmatimonadetes	Gemmatimonadales	Gemmatimonadaceae	Gemmatimonas
6	Bacteria	FCB group	Fibrobacteres	unclassified Fibrobacteres	Fibrobacteres bacterium CG2_30_45_31		
6	Bacteria	FCB group	Bacteroidetes/Chlorobi group	Ignavibacteriae	Ignavibacteria	Ignavibacteriales	Melioribacteraceae
6	Bacteria	FCB group	Bacteroidetes/Chlorobi group	Ignavibacteriae	Ignavibacteria	Ignavibacteriales	Ignavibacteriaceae
6	Bacteria	FCB group	Bacteroidetes/Chlorobi group	Chlorobi	Chlorobia	Chlorobiales	Chlorobiaceae
6	Bacteria	FCB group	Bacteroidetes/Chlorobi group	Chlorobi	Chlorobia	Chlorobiales	Chlorobiaceae
6	Bacteria	FCB group	Bacteroidetes/Chlorobi group	Chlorobi	Chlorobia	Chlorobiales	Chlorobiaceae
6	Bacteria	FCB group	Bacteroidetes/Chlorobi group	Bacteroidetes	unclassified Bacteroidetes	unclassified Bacteroidetes (miscellaneous)	Bacteroidetes bacterium RIFCSPLOWO2_12_FULL_31_6
6	Bacteria	FCB group	Bacteroidetes/Chlorobi group	Bacteroidetes	unclassified Bacteroidetes	unclassified Bacteroidetes (miscellaneous)	Bacteroidetes bacterium RIFCSPHIGHO2_02_FULL_44_7
6	Bacteria	FCB group	Bacteroidetes/Chlorobi group	Bacteroidetes	unclassified Bacteroidetes	unclassified Bacteroidetes (miscellaneous)	Bacteroidetes bacterium 47-18
6	Bacteria	FCB group	Bacteroidetes/Chlorobi group	Bacteroidetes	unclassified Bacteroidetes	unclassified Bacteroidetes (miscellaneous)	Bacteroidetes bacterium 46-16
6	Bacteria	FCB group	Bacteroidetes/Chlorobi group	Bacteroidetes	Sphingobacteriia	Sphingobacteriales	Sphingobacteriaceae
6	Bacteria	FCB group	Bacteroidetes/Chlorobi group	Bacteroidetes	Sphingobacteriia	Sphingobacteriales	Sphingobacteriaceae
6	Bacteria	FCB group	Bacteroidetes/Chlorobi group	Bacteroidetes	Sphingobacteriia	Sphingobacteriales	Sphingobacteriaceae
6	Bacteria	FCB group	Bacteroidetes/Chlorobi group	Bacteroidetes	Sphingobacteriia	Sphingobacteriales	Sphingobacteriaceae
6	Bacteria	FCB group	Bacteroidetes/Chlorobi group	Bacteroidetes	Sphingobacteriia	Sphingobacteriales	Sphingobacteriaceae
6	Bacteria	FCB group	Bacteroidetes/Chlorobi group	Bacteroidetes	Sphingobacteriia	Sphingobacteriales	Sphingobacteriaceae
6	Bacteria	FCB group	Bacteroidetes/Chlorobi group	Bacteroidetes	Sphingobacteriia	Sphingobacteriales	Sphingobacteriaceae
6	Bacteria	FCB group	Bacteroidetes/Chlorobi group	Bacteroidetes	Saprospiria	Saprospirales	Lewinellaceae
6	Bacteria	FCB group	Bacteroidetes/Chlorobi group	Bacteroidetes	Flavobacteriia	Flavobacteriales	unclassified Flavobacteriales
6	Bacteria	FCB group	Bacteroidetes/Chlorobi group	Bacteroidetes	Flavobacteriia	Flavobacteriales	Schleiferiaceae
6	Bacteria	FCB group	Bacteroidetes/Chlorobi group	Bacteroidetes	Flavobacteriia	Flavobacteriales	Flavobacteriaceae
6	Bacteria	FCB group	Bacteroidetes/Chlorobi group	Bacteroidetes	Flavobacteriia	Flavobacteriales	Flavobacteriaceae
6	Bacteria	FCB group	Bacteroidetes/Chlorobi group	Bacteroidetes	Flavobacteriia	Flavobacteriales	Flavobacteriaceae
6	Bacteria	FCB group	Bacteroidetes/Chlorobi group	Bacteroidetes	Flavobacteriia	Flavobacteriales	Flavobacteriaceae
6	Bacteria	FCB group	Bacteroidetes/Chlorobi group	Bacteroidetes	Flavobacteriia	Flavobacteriales	Flavobacteriaceae
6	Bacteria	FCB group	Bacteroidetes/Chlorobi group	Bacteroidetes	Flavobacteriia	Flavobacteriales	Flavobacteriaceae
6	Bacteria	FCB group	Bacteroidetes/Chlorobi group	Bacteroidetes	Flavobacteriia	Flavobacteriales	Flavobacteriaceae
6	Bacteria	FCB group	Bacteroidetes/Chlorobi group	Bacteroidetes	Flavobacteriia	Flavobacteriales	Flavobacteriaceae
6	Bacteria	FCB group	Bacteroidetes/Chlorobi group	Bacteroidetes	Flavobacteriia	Flavobacteriales	Flavobacteriaceae
6	Bacteria	FCB group	Bacteroidetes/Chlorobi group	Bacteroidetes	Flavobacteriia	Flavobacteriales	Flavobacteriaceae
6	Bacteria	FCB group	Bacteroidetes/Chlorobi group	Bacteroidetes	Flavobacteriia	Flavobacteriales	
6	Bacteria	FCB group	Bacteroidetes/Chlorobi group	Bacteroidetes	Cytophagia	Cytophagales	Hymenobacteraceae
6	Bacteria	FCB group	Bacteroidetes/Chlorobi group	Bacteroidetes	Cytophagia	Cytophagales	Hymenobacteraceae
6	Bacteria	FCB group	Bacteroidetes/Chlorobi group	Bacteroidetes	Cytophagia	Cytophagales	Flammeovirgaceae
6	Bacteria	FCB group	Bacteroidetes/Chlorobi group	Bacteroidetes	Cytophagia	Cytophagales	Cytophagaceae
6	Bacteria	FCB group	Bacteroidetes/Chlorobi group	Bacteroidetes	Cytophagia	Cytophagales	Cytophagaceae
6	Bacteria	FCB group	Bacteroidetes/Chlorobi group	Bacteroidetes	Cytophagia	Cytophagales	Cytophagaceae
6	Bacteria	FCB group	Bacteroidetes/Chlorobi group	Bacteroidetes	Cytophagia	Cytophagales	Cytophagaceae
6	Bacteria	FCB group	Bacteroidetes/Chlorobi group	Bacteroidetes	Cytophagia	Cytophagales	Cyclobacteriaceae
6	Bacteria	FCB group	Bacteroidetes/Chlorobi group	Bacteroidetes	Cytophagia	Cytophagales	Cyclobacteriaceae
6	Bacteria	FCB group	Bacteroidetes/Chlorobi group	Bacteroidetes	Cytophagia	Cytophagales	Cyclobacteriaceae
6	Bacteria	FCB group	Bacteroidetes/Chlorobi group	Bacteroidetes	Cytophagia	Cytophagales	Cyclobacteriaceae
6	Bacteria	FCB group	Bacteroidetes/Chlorobi group	Bacteroidetes	Chitinophagia	Chitinophagales	Chitinophagaceae
6	Bacteria	FCB group	Bacteroidetes/Chlorobi group	Bacteroidetes	Chitinophagia	Chitinophagales	Chitinophagaceae
6	Bacteria	FCB group	Bacteroidetes/Chlorobi group	Bacteroidetes	Chitinophagia	Chitinophagales	Chitinophagaceae
6	Bacteria	FCB group	Bacteroidetes/Chlorobi group	Bacteroidetes	Chitinophagia	Chitinophagales	Chitinophagaceae
6	Bacteria	FCB group	Bacteroidetes/Chlorobi group	Bacteroidetes	Bacteroidia	Bacteroidales	unclassified Bacteroidales
6	Bacteria	FCB group	Bacteroidetes/Chlorobi group	Bacteroidetes	Bacteroidia	Bacteroidales	Rikenellaceae
6	Bacteria	FCB group	Bacteroidetes/Chlorobi group	Bacteroidetes	Bacteroidia	Bacteroidales	Rikenellaceae
6	Bacteria	FCB group	Bacteroidetes/Chlorobi group	Bacteroidetes	Bacteroidia	Bacteroidales	Rikenellaceae
6	Bacteria	FCB group	Bacteroidetes/Chlorobi group	Bacteroidetes	Bacteroidia	Bacteroidales	Prevotellaceae
6	Bacteria	FCB group	Bacteroidetes/Chlorobi group	Bacteroidetes	Bacteroidia	Bacteroidales	Prevotellaceae
6	Bacteria	FCB group	Bacteroidetes/Chlorobi group	Bacteroidetes	Bacteroidia	Bacteroidales	Prevotellaceae
6	Bacteria	FCB group	Bacteroidetes/Chlorobi group	Bacteroidetes	Bacteroidia	Bacteroidales	Porphyromonadaceae
6	Bacteria	FCB group	Bacteroidetes/Chlorobi group	Bacteroidetes	Bacteroidia	Bacteroidales	Porphyromonadaceae
6	Bacteria	FCB group	Bacteroidetes/Chlorobi group	Bacteroidetes	Bacteroidia	Bacteroidales	Porphyromonadaceae
6	Bacteria	FCB group	Bacteroidetes/Chlorobi group	Bacteroidetes	Bacteroidia	Bacteroidales	Porphyromonadaceae
6	Bacteria	FCB group	Bacteroidetes/Chlorobi group	Bacteroidetes	Bacteroidia	Bacteroidales	Porphyromonadaceae
6	Bacteria	FCB group	Bacteroidetes/Chlorobi group	Bacteroidetes	Bacteroidia	Bacteroidales	Porphyromonadaceae
6	Bacteria	FCB group	Bacteroidetes/Chlorobi group	Bacteroidetes	Bacteroidia	Bacteroidales	Odoribacteraceae
6	Bacteria	FCB group	Bacteroidetes/Chlorobi group	Bacteroidetes	Bacteroidia	Bacteroidales	Bacteroidaceae
6	Bacteria	FCB group	Bacteroidetes/Chlorobi group	Bacteroidetes	Bacteroidia	Bacteroidales	Bacteroidaceae
6	Bacteria	FCB group	Bacteroidetes/Chlorobi group	Bacteroidetes	Bacteroidia	Bacteroidales	Bacteroidaceae
6	Bacteria	FCB group	Bacteroidetes/Chlorobi group	Bacteroidetes	Bacteroidetes Order II. Incertae sedis	Rhodothermaceae	Salisaeta
6	Bacteria	Elusimicrobia	unclassified Elusimicrobia	Elusimicrobia bacterium RIFOXYD2_FULL_34_15			
6	Bacteria	Elusimicrobia	unclassified Elusimicrobia	Elusimicrobia bacterium RBG_16_66_12			
6	Bacteria	Chrysiogenetes	Chrysiogenetes	Chrysiogenales	Chrysiogenaceae	Desulfurispirillum	Desulfurispirillum indicum
6	Bacteria	Acidobacteria	unclassified Acidobacteria	unclassified Acidobacteria (miscellaneous)	Acidobacteria bacterium RIFCSPLOWO2_12_FULL_60_22		
6	Bacteria	Acidobacteria	unclassified Acidobacteria	unclassified Acidobacteria (miscellaneous)	Acidobacteria bacterium RIFCSPLOWO2_12_FULL_54_10		
6	Bacteria	Acidobacteria	unclassified Acidobacteria	unclassified Acidobacteria (miscellaneous)	Acidobacteria bacterium RIFCSPLOWO2_02_FULL_64_15		
6	Bacteria	Acidobacteria	unclassified Acidobacteria	unclassified Acidobacteria (miscellaneous)	Acidobacteria bacterium 13_1_20CM_2_65_9		
6	Bacteria	Acidobacteria	Acidobacteriia	Acidobacteriales	unclassified Acidobacteriales	Acidobacteriales bacterium 59-55	
6	Bacteria	Acidobacteria	Acidobacteriia	Acidobacteriales	Acidobacteriaceae		
6	ssDNA viruses	Microviridae	environmental samples	uncultured Microviridae			
5	Eukaryota	Stramenopiles	PX clade	Phaeophyceae	Ectocarpales	Chordariaceae	Elachista
5	Eukaryota	Stramenopiles	Oomycetes	Peronosporales	Peronosporaceae	Plasmopara	Plasmopara halstedii
5	Eukaryota	Opisthokonta	Fungi	Mucoromycota	Mortierellomycotina	Mortierellales	Mortierellaceae
5	Eukaryota	Opisthokonta	Fungi	Dikarya	Basidiomycota	Agaricomycotina	Agaricomycetes
5	Eukaryota	Opisthokonta	Fungi	Dikarya	Basidiomycota	Agaricomycotina	Agaricomycetes
5	Eukaryota	Opisthokonta	Fungi	Dikarya	Ascomycota	saccharomyceta	Pezizomycotina
5	Eukaryota	Opisthokonta	Fungi	Dikarya	Ascomycota	saccharomyceta	Pezizomycotina
5	Eukaryota	Opisthokonta	Fungi				
5	Eukaryota	Opisthokonta	Choanoflagellida	Craspedida	Salpingoecidae	Monosiga	Monosiga brevicollis
5	Eukaryota	Haptophyceae	Isochrysidales	Noelaerhabdaceae	Emiliania	Emiliania huxleyi	Emiliania huxleyi CCMP1516
5	Bacteria	unclassified Bacteria	Bacteria candidate phyla	candidate division NC10	unclassified candidate division NC10	candidate division NC10 bacterium RBG_16_65_8	
5	Bacteria	unclassified Bacteria	Bacteria candidate phyla	Patescibacteria group	Parcubacteria group	unclassified Parcubacteria group	
5	Bacteria	unclassified Bacteria	Bacteria candidate phyla	Patescibacteria group	Microgenomates group	Candidatus Roizmanbacteria	Candidatus Roizmanbacteria bacterium RIFCSPLOWO2_01_FULL_45_11
5	Bacteria	unclassified Bacteria	Bacteria candidate phyla	Patescibacteria group	Microgenomates group	Candidatus Roizmanbacteria	
5	Bacteria	unclassified Bacteria	Bacteria candidate phyla	Candidatus Wallbacteria	Candidatus Wallbacteria bacterium GWC2_49_35		
5	Bacteria	unclassified Bacteria	Bacteria candidate phyla	Candidatus Rokubacteria	Candidatus Rokubacteria bacterium 13_1_40CM_4_67_11		
5	Bacteria	unclassified Bacteria	Bacteria candidate phyla	Candidatus Margulisbacteria			
5	Bacteria	unclassified Bacteria	Bacteria candidate phyla	Candidatus Desantisbacteria	Candidatus Desantisbacteria bacterium CG2_30_40_21		
5	Bacteria	unclassified Bacteria	Bacteria candidate phyla	Candidatus Desantisbacteria	Candidatus Desantisbacteria bacterium CG1_02_49_89		
5	Bacteria	unclassified Bacteria	Bacteria candidate phyla				
5	Bacteria	Thermodesulfobacteria	Thermodesulfobacteria	Thermodesulfobacteriales	Thermodesulfobacteriaceae	Thermodesulfatator	Thermodesulfatator atlanticus
5	Bacteria	Thermodesulfobacteria	Thermodesulfobacteria	Thermodesulfobacteriales	Thermodesulfobacteriaceae	Thermodesulfatator	
5	Bacteria	Thermodesulfobacteria	Thermodesulfobacteria	Thermodesulfobacteriales	Thermodesulfobacteriaceae	Caldimicrobium	Caldimicrobium thiodismutans
5	Bacteria	Terrabacteria group	Tenericutes	Mollicutes	Entomoplasmatales	Spiroplasmataceae	Spiroplasma
5	Bacteria	Terrabacteria group	Firmicutes	unclassified Firmicutes sensu stricto	unclassified Firmicutes sensu stricto (miscellaneous)	Firmicutes bacterium ZOR0006	
5	Bacteria	Terrabacteria group	Firmicutes	Tissierellia	Tissierellales	Tissierellaceae	Tissierella
5	Bacteria	Terrabacteria group	Firmicutes	Negativicutes	Veillonellales	Veillonellaceae	Dialister
5	Bacteria	Terrabacteria group	Firmicutes	Negativicutes	Selenomonadales	Sporomusaceae	Sporomusa
5	Bacteria	Terrabacteria group	Firmicutes	Negativicutes	Selenomonadales	Sporomusaceae	Sporolituus
5	Bacteria	Terrabacteria group	Firmicutes	Negativicutes	Selenomonadales	Selenomonadaceae	Schwartzia
5	Bacteria	Terrabacteria group	Firmicutes	Negativicutes	Acidaminococcales	Acidaminococcaceae	Succiniclasticum
5	Bacteria	Terrabacteria group	Firmicutes	Negativicutes	Acidaminococcales	Acidaminococcaceae	Phascolarctobacterium
5	Bacteria	Terrabacteria group	Firmicutes	Negativicutes	Acidaminococcales	Acidaminococcaceae	Acidaminococcus
5	Bacteria	Terrabacteria group	Firmicutes	Erysipelotrichia	Erysipelotrichales	Erysipelotrichaceae	Candidatus Stoquefichus
5	Bacteria	Terrabacteria group	Firmicutes	Erysipelotrichia	Erysipelotrichales	Erysipelotrichaceae	
5	Bacteria	Terrabacteria group	Firmicutes	Clostridia	Thermoanaerobacterales	unclassified Thermoanaerobacterales	Thermoanaerobacterales bacterium 50_218
5	Bacteria	Terrabacteria group	Firmicutes	Clostridia	Thermoanaerobacterales	Thermoanaerobacteraceae	Thermoanaerobacter
5	Bacteria	Terrabacteria group	Firmicutes	Clostridia	Thermoanaerobacterales	Thermoanaerobacteraceae	Moorella group
5	Bacteria	Terrabacteria group	Firmicutes	Clostridia	Thermoanaerobacterales	Thermoanaerobacteraceae	Desulfovirgula
5	Bacteria	Terrabacteria group	Firmicutes	Clostridia	Clostridiales	unclassified Clostridiales	unclassified Clostridiales (miscellaneous)
5	Bacteria	Terrabacteria group	Firmicutes	Clostridia	Clostridiales	unclassified Clostridiales	unclassified Clostridiales (miscellaneous)
5	Bacteria	Terrabacteria group	Firmicutes	Clostridia	Clostridiales	unclassified Clostridiales	unclassified Clostridiales (miscellaneous)
5	Bacteria	Terrabacteria group	Firmicutes	Clostridia	Clostridiales	unclassified Clostridiales	Proteiniborus
5	Bacteria	Terrabacteria group	Firmicutes	Clostridia	Clostridiales	Symbiobacteriaceae	Symbiobacterium
5	Bacteria	Terrabacteria group	Firmicutes	Clostridia	Clostridiales	Ruminococcaceae	unclassified Ruminococcaceae
5	Bacteria	Terrabacteria group	Firmicutes	Clostridia	Clostridiales	Ruminococcaceae	Ruminococcus
5	Bacteria	Terrabacteria group	Firmicutes	Clostridia	Clostridiales	Ruminococcaceae	Ruminococcus
5	Bacteria	Terrabacteria group	Firmicutes	Clostridia	Clostridiales	Ruminococcaceae	Ruminococcus
5	Bacteria	Terrabacteria group	Firmicutes	Clostridia	Clostridiales	Ruminococcaceae	Ruminiclostridium
5	Bacteria	Terrabacteria group	Firmicutes	Clostridia	Clostridiales	Ruminococcaceae	Ruminiclostridium
5	Bacteria	Terrabacteria group	Firmicutes	Clostridia	Clostridiales	Ruminococcaceae	Acetivibrio
5	Bacteria	Terrabacteria group	Firmicutes	Clostridia	Clostridiales	Proteinivoraceae	Anaerobranca
5	Bacteria	Terrabacteria group	Firmicutes	Clostridia	Clostridiales	Peptostreptococcaceae	Peptoanaerobacter
5	Bacteria	Terrabacteria group	Firmicutes	Clostridia	Clostridiales	Peptostreptococcaceae	Criibacterium
5	Bacteria	Terrabacteria group	Firmicutes	Clostridia	Clostridiales	Peptococcaceae	unclassified Peptococcaceae
5	Bacteria	Terrabacteria group	Firmicutes	Clostridia	Clostridiales	Peptococcaceae	Desulfotomaculum
5	Bacteria	Terrabacteria group	Firmicutes	Clostridia	Clostridiales	Peptococcaceae	Desulfotomaculum
5	Bacteria	Terrabacteria group	Firmicutes	Clostridia	Clostridiales	Peptococcaceae	Desulfotomaculum
5	Bacteria	Terrabacteria group	Firmicutes	Clostridia	Clostridiales	Oscillospiraceae	Oscillibacter
5	Bacteria	Terrabacteria group	Firmicutes	Clostridia	Clostridiales	Lachnospiraceae	unclassified Lachnospiraceae
5	Bacteria	Terrabacteria group	Firmicutes	Clostridia	Clostridiales	Lachnospiraceae	unclassified Lachnospiraceae
5	Bacteria	Terrabacteria group	Firmicutes	Clostridia	Clostridiales	Lachnospiraceae	unclassified Lachnospiraceae
5	Bacteria	Terrabacteria group	Firmicutes	Clostridia	Clostridiales	Lachnospiraceae	Parasporobacterium
5	Bacteria	Terrabacteria group	Firmicutes	Clostridia	Clostridiales	Lachnospiraceae	Niameybacter
5	Bacteria	Terrabacteria group	Firmicutes	Clostridia	Clostridiales	Lachnospiraceae	Lachnoclostridium

5	Bacteria	Terrabacteria group	Firmicutes	Clostridia	Clostridiales	Gracilibacteraceae	Gracilibacter
5	Bacteria	Terrabacteria group	Firmicutes	Clostridia	Clostridiales	Eubacteriaceae	unclassified Eubacteriaceae
5	Bacteria	Terrabacteria group	Firmicutes	Clostridia	Clostridiales	Eubacteriaceae	Eubacterium
5	Bacteria	Terrabacteria group	Firmicutes	Clostridia	Clostridiales	Eubacteriaceae	Eubacterium
5	Bacteria	Terrabacteria group	Firmicutes	Clostridia	Clostridiales	Clostridiales incertae sedis	Clostridiales Family XIII. Incertae Sedis
5	Bacteria	Terrabacteria group	Firmicutes	Clostridia	Clostridiales	Clostridiaceae	unclassified Clostridiaceae
5	Bacteria	Terrabacteria group	Firmicutes	Clostridia	Clostridiales	Clostridiaceae	Tindallia
5	Bacteria	Terrabacteria group	Firmicutes	Clostridia	Clostridiales	Clostridiaceae	Oxobacter
5	Bacteria	Terrabacteria group	Firmicutes	Clostridia	Clostridiales	Clostridiaceae	Clostridium
5	Bacteria	Terrabacteria group	Firmicutes	Clostridia	Clostridiales	Clostridiaceae	Clostridium
5	Bacteria	Terrabacteria group	Firmicutes	Clostridia	Clostridiales	Clostridiaceae	Clostridium
5	Bacteria	Terrabacteria group	Firmicutes	Clostridia	Clostridiales	Clostridiaceae	Clostridium
5	Bacteria	Terrabacteria group	Firmicutes	Clostridia	Clostridiales	Clostridiaceae	Clostridium
5	Bacteria	Terrabacteria group	Firmicutes	Clostridia	Clostridiales	Clostridiaceae	Clostridium
5	Bacteria	Terrabacteria group	Firmicutes	Clostridia	Clostridiales	Clostridiaceae	Clostridium
5	Bacteria	Terrabacteria group	Firmicutes	Clostridia	Clostridiales	Clostridiaceae	Clostridium
5	Bacteria	Terrabacteria group	Firmicutes	Clostridia	Clostridiales	Clostridiaceae	Clostridium
5	Bacteria	Terrabacteria group	Firmicutes	Clostridia	Clostridiales	Clostridiaceae	Clostridium
5	Bacteria	Terrabacteria group	Firmicutes	Clostridia	Clostridiales	Clostridiaceae	Clostridium
5	Bacteria	Terrabacteria group	Firmicutes	Clostridia	Clostridiales	Clostridiaceae	Clostridiisalibacter
5	Bacteria	Terrabacteria group	Firmicutes	Clostridia	Clostridiales	Clostridiaceae	Caloramator
5	Bacteria	Terrabacteria group	Firmicutes	Clostridia	Clostridiales	Clostridiaceae	Alkaliphilus
5	Bacteria	Terrabacteria group	Firmicutes	Clostridia	Clostridiales	Christensenellaceae	Christensenella
5	Bacteria	Terrabacteria group	Firmicutes	Clostridia	Clostridiales	Caldicoprobacteraceae	Caldicoprobacter
5	Bacteria	Terrabacteria group	Firmicutes	Bacilli	Bacillales	unclassified Bacillales	Desulfuribacillus
5	Bacteria	Terrabacteria group	Firmicutes	Bacilli	Bacillales	Thermoactinomycetaceae	Thermoactinomyces
5	Bacteria	Terrabacteria group	Firmicutes	Bacilli	Bacillales	Thermoactinomycetaceae	Novibacillus
5	Bacteria	Terrabacteria group	Firmicutes	Bacilli	Bacillales	Staphylococcaceae	Staphylococcus
5	Bacteria	Terrabacteria group	Firmicutes	Bacilli	Bacillales	Paenibacillaceae	Paenibacillus
5	Bacteria	Terrabacteria group	Firmicutes	Bacilli	Bacillales	Paenibacillaceae	Paenibacillus
5	Bacteria	Terrabacteria group	Firmicutes	Bacilli	Bacillales	Paenibacillaceae	Paenibacillus
5	Bacteria	Terrabacteria group	Firmicutes	Bacilli	Bacillales	Paenibacillaceae	Paenibacillus
5	Bacteria	Terrabacteria group	Firmicutes	Bacilli	Bacillales	Paenibacillaceae	Paenibacillus
5	Bacteria	Terrabacteria group	Firmicutes	Bacilli	Bacillales	Paenibacillaceae	Paenibacillus
5	Bacteria	Terrabacteria group	Firmicutes	Bacilli	Bacillales	Paenibacillaceae	Cohnella
5	Bacteria	Terrabacteria group	Firmicutes	Bacilli	Bacillales	Paenibacillaceae	Cohnella
5	Bacteria	Terrabacteria group	Firmicutes	Bacilli	Bacillales	Paenibacillaceae	Brevibacillus
5	Bacteria	Terrabacteria group	Firmicutes	Bacilli	Bacillales	Listeriaceae	Listeria
5	Bacteria	Terrabacteria group	Firmicutes	Bacilli	Bacillales	Bacillales incertae sedis	Bacillales Family XII. Incertae Sedis
5	Bacteria	Terrabacteria group	Firmicutes	Bacilli	Bacillales	Bacillaceae	Vulcanibacillus
5	Bacteria	Terrabacteria group	Firmicutes	Bacilli	Bacillales	Bacillaceae	Anoxybacillus
5	Bacteria	Terrabacteria group	Firmicutes	Bacilli	Bacillales	Alicyclobacillaceae	Alicyclobacillus
5	Bacteria	Terrabacteria group	Deinococcus-Thermus	Deinococci	Thermales	Thermaceae	Thermus
5	Bacteria	Terrabacteria group	Deinococcus-Thermus	Deinococci	Thermales	Thermaceae	Meiothermus
5	Bacteria	Terrabacteria group	Deinococcus-Thermus	Deinococci	Thermales	Thermaceae	Marinithermus
5	Bacteria	Terrabacteria group	Cyanobacteria/Melainabacteria group	Cyanobacteria	unclassified Cyanobacteria	unclassified Cyanobacteria (miscellaneous)	Cyanobacteria bacterium 13_1_20CM_4_61_6
5	Bacteria	Terrabacteria group	Cyanobacteria/Melainabacteria group	Cyanobacteria	Synechococcales	Merismopediaceae	Synechocystis
5	Bacteria	Terrabacteria group	Cyanobacteria/Melainabacteria group	Cyanobacteria	Synechococcales	Leptolyngbyaceae	Leptolyngbya
5	Bacteria	Terrabacteria group	Cyanobacteria/Melainabacteria group	Cyanobacteria	Spirulinales	Spirulinaceae	Spirulina
5	Bacteria	Terrabacteria group	Cyanobacteria/Melainabacteria group	Cyanobacteria	Pleurocapsales	Xenococcaceae	Xenococcus
5	Bacteria	Terrabacteria group	Cyanobacteria/Melainabacteria group	Cyanobacteria	Pleurocapsales	Hyellaceae	Myxosarcina
5	Bacteria	Terrabacteria group	Cyanobacteria/Melainabacteria group	Cyanobacteria	Oscillatoriophycideae	Oscillatoriales	Oscillatoriaceae
5	Bacteria	Terrabacteria group	Cyanobacteria/Melainabacteria group	Cyanobacteria	Oscillatoriophycideae	Oscillatoriales	Oscillatoriaceae
5	Bacteria	Terrabacteria group	Cyanobacteria/Melainabacteria group	Cyanobacteria	Oscillatoriophycideae	Oscillatoriales	Microcoleaceae
5	Bacteria	Terrabacteria group	Cyanobacteria/Melainabacteria group	Cyanobacteria	Oscillatoriophycideae	Oscillatoriales	Microcoleaceae
5	Bacteria	Terrabacteria group	Cyanobacteria/Melainabacteria group	Cyanobacteria	Oscillatoriophycideae	Oscillatoriales	Microcoleaceae
5	Bacteria	Terrabacteria group	Cyanobacteria/Melainabacteria group	Cyanobacteria	Oscillatoriophycideae	Oscillatoriales	Coleofasciculaceae
5	Bacteria	Terrabacteria group	Cyanobacteria/Melainabacteria group	Cyanobacteria	Oscillatoriophycideae	Chroococcales	Chroococcaceae
5	Bacteria	Terrabacteria group	Cyanobacteria/Melainabacteria group	Cyanobacteria	Nostocales	Tolypothrichaceae	Tolypothrix
5	Bacteria	Terrabacteria group	Cyanobacteria/Melainabacteria group	Cyanobacteria	Nostocales	Nostocaceae	Anabaena
5	Bacteria	Terrabacteria group	Chloroflexi	unclassified Chloroflexi	unclassified Chloroflexi (miscellaneous)	Chloroflexi bacterium RBG_16_54_18	
5	Bacteria	Terrabacteria group	Chloroflexi	unclassified Chloroflexi	unclassified Chloroflexi (miscellaneous)	Chloroflexi bacterium RBG_13_68_17	
5	Bacteria	Terrabacteria group	Chloroflexi	unclassified Chloroflexi	unclassified Chloroflexi (miscellaneous)	Chloroflexi bacterium GWC2_73_18	
5	Bacteria	Terrabacteria group	Chloroflexi	unclassified Chloroflexi	unclassified Chloroflexi (miscellaneous)	Chloroflexi bacterium GWB2_54_36	
5	Bacteria	Terrabacteria group	Chloroflexi	Thermomicrobia	Thermomicrobiales	Thermomicrobiaceae	Thermomicrobium
5	Bacteria	Terrabacteria group	Chloroflexi	Chloroflexia	Herpetosiphonales	Herpetosiphonaceae	Herpetosiphon
5	Bacteria	Terrabacteria group	Chloroflexi	Anaerolineae	unclassified Anaerolineae	Anaerolineae bacterium UTCFX3	
5	Bacteria	Terrabacteria group	Chloroflexi	Anaerolineae	unclassified Anaerolineae	Anaerolineae bacterium UTCFX2	
5	Bacteria	Terrabacteria group	Chloroflexi	Anaerolineae	Anaerolineales	Anaerolineaceae	unclassified Anaerolineaceae
5	Bacteria	Terrabacteria group	Chloroflexi	Anaerolineae	Anaerolineales	Anaerolineaceae	Longilinea
5	Bacteria	Terrabacteria group	Chloroflexi	Anaerolineae	Anaerolineales	Anaerolineaceae	Levilinea
5	Bacteria	Terrabacteria group	Chloroflexi	Anaerolineae	Anaerolineales	Anaerolineaceae	Leptolinea
5	Bacteria	Terrabacteria group	Actinobacteria	unclassified Actinobacteria	Actinobacteria bacterium CG2_30_50_142		
5	Bacteria	Terrabacteria group	Actinobacteria	unclassified Actinobacteria	Actinobacteria bacterium 13_2_20CM_2_66_6		
5	Bacteria	Terrabacteria group	Actinobacteria	Thermoleophilia	Solirubrobacterales	unclassified Solirubrobacterales	Solirubrobacterales bacterium 70-9
5	Bacteria	Terrabacteria group	Actinobacteria	Thermoleophilia	Solirubrobacterales	Solirubrobacteraceae	Solirubrobacter
5	Bacteria	Terrabacteria group	Actinobacteria	Coriobacteriia	Coriobacteriales		
5	Bacteria	Terrabacteria group	Actinobacteria	Actinobacteria	Streptosporangiales	Streptosporangiaceae	Sinosporangium
5	Bacteria	Terrabacteria group	Actinobacteria	Actinobacteria	Streptosporangiales	Streptosporangiaceae	
5	Bacteria	Terrabacteria group	Actinobacteria	Actinobacteria	Streptomycetales	Streptomycetaceae	unclassified Streptomycetaceae
5	Bacteria	Terrabacteria group	Actinobacteria	Actinobacteria	Streptomycetales	Streptomycetaceae	Streptacidiphilus
5	Bacteria	Terrabacteria group	Actinobacteria	Actinobacteria	Streptomycetales	Streptomycetaceae	Streptacidiphilus
5	Bacteria	Terrabacteria group	Actinobacteria	Actinobacteria	Pseudonocardiales	Pseudonocardiaceae	Pseudonocardia
5	Bacteria	Terrabacteria group	Actinobacteria	Actinobacteria	Pseudonocardiales	Pseudonocardiaceae	Pseudonocardia
5	Bacteria	Terrabacteria group	Actinobacteria	Actinobacteria	Pseudonocardiales	Pseudonocardiaceae	Amycolatopsis
5	Bacteria	Terrabacteria group	Actinobacteria	Actinobacteria	Pseudonocardiales	Pseudonocardiaceae	Amycolatopsis
5	Bacteria	Terrabacteria group	Actinobacteria	Actinobacteria	Propionibacteriales	Propionibacteriaceae	
5	Bacteria	Terrabacteria group	Actinobacteria	Actinobacteria	Propionibacteriales	Nocardioidaceae	Nocardioides
5	Bacteria	Terrabacteria group	Actinobacteria	Actinobacteria	Propionibacteriales	Nocardioidaceae	Nocardioides
5	Bacteria	Terrabacteria group	Actinobacteria	Actinobacteria	Propionibacteriales	Nocardioidaceae	Marmoricola
5	Bacteria	Terrabacteria group	Actinobacteria	Actinobacteria	Micromonosporales	Micromonosporaceae	Catenuloplanes
5	Bacteria	Terrabacteria group	Actinobacteria	Actinobacteria	Micromonosporales	Micromonosporaceae	Asanoa
5	Bacteria	Terrabacteria group	Actinobacteria	Actinobacteria	Micromonosporales	Micromonosporaceae	Actinoplanes
5	Bacteria	Terrabacteria group	Actinobacteria	Actinobacteria	Micromonosporales	Micromonosporaceae	Actinocatenispora
5	Bacteria	Terrabacteria group	Actinobacteria	Actinobacteria	Micrococcales	Promicromonosporaceae	Cellulosimicrobium
5	Bacteria	Terrabacteria group	Actinobacteria	Actinobacteria	Micrococcales	Microbacteriaceae	Agreia
5	Bacteria	Terrabacteria group	Actinobacteria	Actinobacteria	Micrococcales	Intrasporangiaceae	Tetrasphaera
5	Bacteria	Terrabacteria group	Actinobacteria	Actinobacteria	Micrococcales	Demequinaceae	Demequina
5	Bacteria	Terrabacteria group	Actinobacteria	Actinobacteria	Frankiales	unclassified Frankiales	unclassified Frankiales (miscellaneous)
5	Bacteria	Terrabacteria group	Actinobacteria	Actinobacteria	Frankiales	Sporichthyaceae	Sporichthya
5	Bacteria	Terrabacteria group	Actinobacteria	Actinobacteria	Corynebacteriales	Nocardiaceae	Rhodococcus
5	Bacteria	Terrabacteria group	Actinobacteria	Actinobacteria	Corynebacteriales	Nocardiaceae	Rhodococcus
5	Bacteria	Terrabacteria group	Actinobacteria	Actinobacteria	Corynebacteriales	Nocardiaceae	Rhodococcus
5	Bacteria	Terrabacteria group	Actinobacteria	Actinobacteria	Corynebacteriales	Nocardiaceae	Rhodococcus
5	Bacteria	Terrabacteria group	Actinobacteria	Actinobacteria	Corynebacteriales	Nocardiaceae	Nocardia
5	Bacteria	Terrabacteria group	Actinobacteria	Actinobacteria	Corynebacteriales	Nocardiaceae	Nocardia
5	Bacteria	Terrabacteria group	Actinobacteria	Actinobacteria	Corynebacteriales	Nocardiaceae	Nocardia
5	Bacteria	Terrabacteria group	Actinobacteria	Actinobacteria	Corynebacteriales	Mycobacteriaceae	Mycobacterium
5	Bacteria	Terrabacteria group	Actinobacteria	Actinobacteria	Corynebacteriales	Gordoniaceae	Gordonia
5	Bacteria	Terrabacteria group	Actinobacteria	Actinobacteria	Bifidobacteriales	Bifidobacteriaceae	Bifidobacterium
5	Bacteria	Terrabacteria group	Actinobacteria	Acidimicrobiia	Acidimicrobiales	Acidimicrobiaceae	Ferrithrix
5	Bacteria	Terrabacteria group	Actinobacteria	Acidimicrobiia	Acidimicrobiales	Acidimicrobiaceae	Acidimicrobium
5	Bacteria	Spirochaetes	unclassified Spirochaetes	Spirochaetes bacterium GWF1_51_8			
5	Bacteria	Spirochaetes	Spirochaetia	Spirochaetales	Spirochaetaceae	Treponema	Treponema brennaborense
5	Bacteria	Spirochaetes	Spirochaetia	Spirochaetales	Spirochaetaceae	Spirochaeta	Spirochaeta sp. LUC14_002_19_P3
5	Bacteria	Spirochaetes	Spirochaetia	Spirochaetales	Spirochaetaceae	Spirochaeta	Spirochaeta cellobiosiphila
5	Bacteria	Spirochaetes	Spirochaetia	Spirochaetales	Spirochaetaceae	Sediminispirochaeta	Sediminispirochaeta bajacaliforniensis
5	Bacteria	Spirochaetes	Spirochaetia	Spirochaetales	Spirochaetaceae	Salinispira	Salinispira pacifica
5	Bacteria	Proteobacteria	unclassified Proteobacteria	unclassified Proteobacteria (miscellaneous)	Proteobacteria bacterium SG_bin8		
5	Bacteria	Proteobacteria	environmental samples	Proteobacteria bacterium CAG:495			
5	Bacteria	Proteobacteria	delta/epsilon subdivisions	Epsilonproteobacteria	Campylobacterales	Campylobacteraceae	Campylobacter
5	Bacteria	Proteobacteria	delta/epsilon subdivisions	Deltaproteobacteria	unclassified Deltaproteobacteria	unclassified Deltaproteobacteria (miscellaneous)	Deltaproteobacteria bacterium SG8_13
5	Bacteria	Proteobacteria	delta/epsilon subdivisions	Deltaproteobacteria	unclassified Deltaproteobacteria	unclassified Deltaproteobacteria (miscellaneous)	Deltaproteobacteria bacterium RIFOXYD12_FULL_57_12
5	Bacteria	Proteobacteria	delta/epsilon subdivisions	Deltaproteobacteria	unclassified Deltaproteobacteria	unclassified Deltaproteobacteria (miscellaneous)	Deltaproteobacteria bacterium RIFOXYD12_FULL_50_9
5	Bacteria	Proteobacteria	delta/epsilon subdivisions	Deltaproteobacteria	unclassified Deltaproteobacteria	unclassified Deltaproteobacteria (miscellaneous)	Deltaproteobacteria bacterium RBG_13_65_10
5	Bacteria	Proteobacteria	delta/epsilon subdivisions	Deltaproteobacteria	unclassified Deltaproteobacteria	unclassified Deltaproteobacteria (miscellaneous)	Deltaproteobacteria bacterium RBG_13_60_28
5	Bacteria	Proteobacteria	delta/epsilon subdivisions	Deltaproteobacteria	unclassified Deltaproteobacteria	unclassified Deltaproteobacteria (miscellaneous)	Deltaproteobacteria bacterium RBG_13_49_15
5	Bacteria	Proteobacteria	delta/epsilon subdivisions	Deltaproteobacteria	unclassified Deltaproteobacteria	unclassified Deltaproteobacteria (miscellaneous)	Deltaproteobacteria bacterium RBG_13_43_22
5	Bacteria	Proteobacteria	delta/epsilon subdivisions	Deltaproteobacteria	Syntrophobacterales	Syntrophaceae	Desulfobacca
5	Bacteria	Proteobacteria	delta/epsilon subdivisions	Deltaproteobacteria	Myxococcales	Cystobacterineae	Archangiaceae
5	Bacteria	Proteobacteria	delta/epsilon subdivisions	Deltaproteobacteria	Desulfuromonadales	Geobacteraceae	unclassified Geobacteraceae
5	Bacteria	Proteobacteria	delta/epsilon subdivisions	Deltaproteobacteria	Desulfuromonadales	Geobacteraceae	Geobacter
5	Bacteria	Proteobacteria	delta/epsilon subdivisions	Deltaproteobacteria	Desulfuromonadales	Desulfuromonadaceae	Desulfuromonas
5	Bacteria	Proteobacteria	delta/epsilon subdivisions	Deltaproteobacteria	Desulfovibrionales	Desulfovibrionaceae	Desulfovibrio
5	Bacteria	Proteobacteria	delta/epsilon subdivisions	Deltaproteobacteria	Desulfovibrionales	Desulfovibrionaceae	Desulfovibrio
5	Bacteria	Proteobacteria	delta/epsilon subdivisions	Deltaproteobacteria	Desulfobacterales	unclassified Desulfobacterales	Desulfobacterales bacterium S5133MH16
5	Bacteria	Proteobacteria	delta/epsilon subdivisions	Deltaproteobacteria	Desulfobacterales	Desulfobacteraceae	unclassified Desulfobacteraceae
5	Bacteria	Proteobacteria	delta/epsilon subdivisions	Deltaproteobacteria	Desulfobacterales	Desulfobacteraceae	unclassified Desulfobacteraceae
5	Bacteria	Proteobacteria	delta/epsilon subdivisions	Deltaproteobacteria	Desulfobacterales	Desulfobacteraceae	unclassified Desulfobacteraceae
5	Bacteria	Proteobacteria	delta/epsilon subdivisions	Deltaproteobacteria	Desulfobacterales	Desulfobacteraceae	unclassified Desulfobacteraceae
5	Bacteria	Proteobacteria	delta/epsilon subdivisions	Deltaproteobacteria	Desulfobacterales	Desulfobacteraceae	Desulfotignum
5	Bacteria	Proteobacteria	delta/epsilon subdivisions	Deltaproteobacteria	Desulfobacterales	Desulfobacteraceae	Desulforegula
5	Bacteria	Proteobacteria	delta/epsilon subdivisions	Deltaproteobacteria	Desulfobacterales	Desulfobacteraceae	Desulfobacterium
5	Bacteria	Proteobacteria	delta/epsilon subdivisions	Deltaproteobacteria	Desulfobacterales	Desulfobacteraceae	Desulfobacter
5	Bacteria	Proteobacteria	delta/epsilon subdivisions	Deltaproteobacteria	Bdellovibrionales	Halobacteriovoraceae	Halobacteriovorax
5	Bacteria	Proteobacteria	delta/epsilon subdivisions	Deltaproteobacteria	Bdellovibrionales	Bacteriovoracaceae	Bacteriovorax
5	Bacteria	Proteobacteria	Zetaproteobacteria	unclassified Zetaproteobacteria	Zetaproteobacteria bacterium CG2_30_59_37		
5	Bacteria	Proteobacteria	Zetaproteobacteria	unclassified Zetaproteobacteria	Zetaproteobacteria bacterium CG2_30_46_52		
5	Bacteria	Proteobacteria	Gammaproteobacteria	unclassified Gammaproteobacteria	unclassified Gammaproteobacteria (miscellaneous)	Gammaproteobacteria bacterium RIFCSPHIGHO2_12_FULL_41_20	
5	Bacteria	Proteobacteria	Gammaproteobacteria	unclassified Gammaproteobacteria	unclassified Gammaproteobacteria (miscellaneous)	Gammaproteobacteria bacterium MedPE	
5	Bacteria	Proteobacteria	Gammaproteobacteria	unclassified Gammaproteobacteria	Sedimenticola	Sedimenticola thiotaurini	
5	Bacteria	Proteobacteria	Gammaproteobacteria	unclassified Gammaproteobacteria	Alkalimonas	Alkalimonas amylolytica	
5	Bacteria	Proteobacteria	Gammaproteobacteria	Xanthomonadales	Xanthomonadaceae	Xylella	Xylella taiwanensis
5	Bacteria	Proteobacteria	Gammaproteobacteria	Xanthomonadales	Xanthomonadaceae	Stenotrophomonas	Stenotrophomonas koreensis
5	Bacteria	Proteobacteria	Gammaproteobacteria	Xanthomonadales	Xanthomonadaceae	Pseudoxanthomonas	Pseudoxanthomonas mexicana
5	Bacteria	Proteobacteria	Gammaproteobacteria	Xanthomonadales	Xanthomonadaceae	Pseudoxanthomonas	Pseudoxanthomonas dokdonensis
5	Bacteria	Proteobacteria	Gammaproteobacteria	Xanthomonadales	Xanthomonadaceae	Lysobacter	Lysobacter sp. A03
5	Bacteria	Proteobacteria	Gammaproteobacteria	Xanthomonadales	Rhodanobacteraceae	Rhodanobacter	Rhodanobacter sp. B05
5	Bacteria	Proteobacteria	Gammaproteobacteria	Xanthomonadales	Rhodanobacteraceae	Luteibacter	Luteibacter sp. 9135
5	Bacteria	Proteobacteria	Gammaproteobacteria	Xanthomonadales	Rhodanobacteraceae	Dyella	Dyella thiooxydans
5	Bacteria	Proteobacteria	Gammaproteobacteria	Xanthomonadales	Rhodanobacteraceae	Dyella	Dyella sp. OK004
5	Bacteria	Proteobacteria	Gammaproteobacteria	Vibrionales	Vibrionaceae	Photobacterium	Photobacterium profundum
5	Bacteria	Proteobacteria	Gammaproteobacteria	Vibrionales	Vibrionaceae	Photobacterium	Photobacterium leiognathi
5	Bacteria	Proteobacteria	Gammaproteobacteria	Thiotrichales	Thiotrichaceae	Thioploca	Thioploca ingrica
5	Bacteria	Proteobacteria	Gammaproteobacteria	Thiotrichales	Piscirickettsiaceae	Methylophaga	Methylophaga frappieri
5	Bacteria	Proteobacteria	Gammaproteobacteria	Thiotrichales	Piscirickettsiaceae	Cycloclasticus	Cycloclasticus sp. symbiont of Poecilosclerida sp. N
5	Bacteria	Proteobacteria	Gammaproteobacteria	Thiotrichales	Piscirickettsiaceae	Cycloclasticus	Cycloclasticus sp. symbiont of Poecilosclerida sp. M
5	Bacteria	Proteobacteria	Gammaproteobacteria	Thiotrichales	Piscirickettsiaceae	Cycloclasticus	
5	Bacteria	Proteobacteria	Gammaproteobacteria	Pseudomonadales	unclassified Pseudomonadales	unclassified Pseudomonadales (miscellaneous)	Pseudomonadales bacterium RIFCSPLOWO2_02_FULL_63_210
5	Bacteria	Proteobacteria	Gammaproteobacteria	Pseudomonadales	Pseudomonadaceae	Pseudomonas	Pseudomonas syringae group
5	Bacteria	Proteobacteria	Gammaproteobacteria	Pseudomonadales	Pseudomonadaceae	Pseudomonas	Pseudomonas syringae group
5	Bacteria	Proteobacteria	Gammaproteobacteria	Pseudomonadales	Pseudomonadaceae	Pseudomonas	Pseudomonas sp. LFM046
5	Bacteria	Proteobacteria	Gammaproteobacteria	Pseudomonadales	Pseudomonadaceae	Pseudomonas	Pseudomonas pertucinogena group
5	Bacteria	Proteobacteria	Gammaproteobacteria	Pseudomonadales	Pseudomonadaceae	Pseudomonas	Pseudomonas pertucinogena group
5	Bacteria	Proteobacteria	Gammaproteobacteria	Pseudomonadales	Pseudomonadaceae	Pseudomonas	Pseudomonas panipatensis
5	Bacteria	Proteobacteria	Gammaproteobacteria	Pseudomonadales	Pseudomonadaceae	Pseudomonas	Pseudomonas graminis
5	Bacteria	Proteobacteria	Gammaproteobacteria	Pseudomonadales	Pseudomonadaceae	Pseudomonas	Pseudomonas bauzanensis
5	Bacteria	Proteobacteria	Gammaproteobacteria	Pseudomonadales	Pseudomonadaceae	Pseudomonas	Pseudomonas argentinensis
5	Bacteria	Proteobacteria	Gammaproteobacteria	Pseudomonadales	Pseudomonadaceae	Oblitimonas	Oblitimonas alkaliphila
5	Bacteria	Proteobacteria	Gammaproteobacteria	Pseudomonadales	Moraxellaceae	Moraxella	Moraxella pluranimalium
5	Bacteria	Proteobacteria	Gammaproteobacteria	Pseudomonadales	Moraxellaceae	Moraxella	Moraxella lincolnii
5	Bacteria	Proteobacteria	Gammaproteobacteria	Pseudomonadales	Moraxellaceae	Acinetobacter	Acinetobacter sp. WCHA34
5	Bacteria	Proteobacteria	Gammaproteobacteria	Pseudomonadales	Moraxellaceae	Acinetobacter	Acinetobacter calcoaceticus/baumannii complex
5	Bacteria	Proteobacteria	Gammaproteobacteria	Pasteurellales	Pasteurellaceae	Haemophilus	[Haemophilus] parasuis
5	Bacteria	Proteobacteria	Gammaproteobacteria	Oceanospirillales	Saccharospirillaceae	Gynuella	Gynuella sunshinyii
5	Bacteria	Proteobacteria	Gammaproteobacteria	Oceanospirillales	Oceanospirillaceae	Oceanospirillum	Oceanospirillum beijerinckii
5	Bacteria	Proteobacteria	Gammaproteobacteria	Oceanospirillales	Oceanospirillaceae	Neptunomonas	Neptunomonas japonica
5	Bacteria	Proteobacteria	Gammaproteobacteria	Oceanospirillales	Oceanospirillaceae	Marinospirillum	Marinospirillum celere
5	Bacteria	Proteobacteria	Gammaproteobacteria	Oceanospirillales	Oceanospirillaceae	Marinobacterium	Marinobacterium profundum
5	Bacteria	Proteobacteria	Gammaproteobacteria	Oceanospirillales	Oceanospirillaceae	Marinobacterium	Marinobacterium litorale
5	Bacteria	Proteobacteria	Gammaproteobacteria	Oceanospirillales	Oceanospirillaceae	Marinobacterium	Marinobacterium georgiense
5	Bacteria	Proteobacteria	Gammaproteobacteria	Oceanospirillales	Oceanospirillaceae	Amphritea	Amphritea atlantica
5	Bacteria	Proteobacteria	Gammaproteobacteria	Oceanospirillales	Halomonadaceae	Halomonas	Halomonas xinjiangensis
5	Bacteria	Proteobacteria	Gammaproteobacteria	Oceanospirillales	Halomonadaceae	Halomonas	Halomonas sp. BC04
5	Bacteria	Proteobacteria	Gammaproteobacteria	Oceanospirillales	Halomonadaceae	Halomonas	Halomonas ilicicola
5	Bacteria	Proteobacteria	Gammaproteobacteria	Oceanospirillales	Hahellaceae	Endozoicomonas	Endozoicomonas sp. S-B4-1U
5	Bacteria	Proteobacteria	Gammaproteobacteria	Oceanospirillales	Hahellaceae	Endozoicomonas	Endozoicomonas numazuensis
5	Bacteria	Proteobacteria	Gammaproteobacteria	Oceanospirillales	Alcanivoracaceae	Alcanivorax	Alcanivorax sp. P2S70
5	Bacteria	Proteobacteria	Gammaproteobacteria	Methylococcales	Methylothermaceae	unclassified Methylothermaceae	Methylothermaceae bacteria B42
5	Bacteria	Proteobacteria	Gammaproteobacteria	Methylococcales	Methylococcaceae	Methylomicrobium	Methylomicrobium alcaliphilum
5	Bacteria	Proteobacteria	Gammaproteobacteria	Legionellales	Legionellaceae	Legionella	Legionella londiniensis
5	Bacteria	Proteobacteria	Gammaproteobacteria	Legionellales	Legionellaceae	Legionella	Legionella geestiana
5	Bacteria	Proteobacteria	Gammaproteobacteria	Legionellales	Legionellaceae	Legionella	Legionella fallonii
5	Bacteria	Proteobacteria	Gammaproteobacteria	Legionellales	Legionellaceae	Legionella	Legionella adelaidensis
5	Bacteria	Proteobacteria	Gammaproteobacteria	Legionellales	Coxiellaceae	Coxiella	Coxiella sp. DG_40
5	Bacteria	Proteobacteria	Gammaproteobacteria	Enterobacterales	Yersiniaceae	Yersinia	Yersinia enterocolitica
5	Bacteria	Proteobacteria	Gammaproteobacteria	Enterobacterales	Yersiniaceae	Serratia	Serratia sp. ATCC 39006
5	Bacteria	Proteobacteria	Gammaproteobacteria	Enterobacterales	Pectobacteriaceae	Dickeya	Dickeya dianthicola
5	Bacteria	Proteobacteria	Gammaproteobacteria	Enterobacterales	Pectobacteriaceae	Brenneria	Brenneria goodwinii
5	Bacteria	Proteobacteria	Gammaproteobacteria	Enterobacterales	Erwiniaceae	Erwinia	Erwinia pyrifoliae
5	Bacteria	Proteobacteria	Gammaproteobacteria	Enterobacterales	Erwiniaceae	Erwinia	
5	Bacteria	Proteobacteria	Gammaproteobacteria	Enterobacterales	Enterobacteriaceae	Escherichia	
5	Bacteria	Proteobacteria	Gammaproteobacteria	Enterobacterales	Enterobacteriaceae	Buttiauxella	Buttiauxella agrestis
5	Bacteria	Proteobacteria	Gammaproteobacteria	Enterobacterales	Enterobacteriaceae	Buttiauxella	
5	Bacteria	Proteobacteria	Gammaproteobacteria	Chromatiales	Ectothiorhodospiraceae	Spiribacter	Spiribacter curvatus
5	Bacteria	Proteobacteria	Gammaproteobacteria	Chromatiales	Ectothiorhodospiraceae	Ectothiorhodosinus	Ectothiorhodosinus mongolicus
5	Bacteria	Proteobacteria	Gammaproteobacteria	Chromatiales	Chromatiaceae	Thiocapsa	
5	Bacteria	Proteobacteria	Gammaproteobacteria	Chromatiales	Chromatiaceae	Nitrosococcus	Nitrosococcus oceani
5	Bacteria	Proteobacteria	Gammaproteobacteria	Cellvibrionales	Spongiibacteraceae	Spongiibacter	Spongiibacter marinus
5	Bacteria	Proteobacteria	Gammaproteobacteria	Cellvibrionales	Porticoccaceae	unclassified Porticoccaceae	Gammaproteobacteria bacterium MOLA455
5	Bacteria	Proteobacteria	Gammaproteobacteria	Cellvibrionales	Porticoccaceae	Porticoccus	Porticoccus hydrocarbonoclasticus
5	Bacteria	Proteobacteria	Gammaproteobacteria	Cellvibrionales	Microbulbiferaceae	Microbulbifer	Microbulbifer sp. CCB-MM1
5	Bacteria	Proteobacteria	Gammaproteobacteria	Cellvibrionales	Microbulbiferaceae	Microbulbifer	Microbulbifer donghaiensis
5	Bacteria	Proteobacteria	Gammaproteobacteria	Cardiobacteriales	Cardiobacteriaceae	Dichelobacter	Dichelobacter nodosus
5	Bacteria	Proteobacteria	Gammaproteobacteria	Alteromonadales	Shewanellaceae	Shewanella	Shewanella xiamenensis
5	Bacteria	Proteobacteria	Gammaproteobacteria	Alteromonadales	Shewanellaceae	Shewanella	Shewanella sp. UCD-KL21
5	Bacteria	Proteobacteria	Gammaproteobacteria	Alteromonadales	Shewanellaceae	Shewanella	Shewanella halifaxensis
5	Bacteria	Proteobacteria	Gammaproteobacteria	Alteromonadales	Shewanellaceae	Shewanella	Shewanella colwelliana
5	Bacteria	Proteobacteria	Gammaproteobacteria	Alteromonadales	Pseudoalteromonadaceae	Pseudoalteromonas	Pseudoalteromonas sp. OCN003
5	Bacteria	Proteobacteria	Gammaproteobacteria	Alteromonadales	Colwelliaceae	Thalassomonas	Thalassomonas actiniarum
5	Bacteria	Proteobacteria	Gammaproteobacteria	Alteromonadales	Alteromonadaceae	Paraglaciecola	Paraglaciecola polaris
5	Bacteria	Proteobacteria	Gammaproteobacteria	Alteromonadales	Alteromonadaceae	Lacimicrobium	Lacimicrobium alkaliphilum
5	Bacteria	Proteobacteria	Gammaproteobacteria	Alteromonadales	Alteromonadaceae	Glaciecola	Glaciecola punicea
5	Bacteria	Proteobacteria	Gammaproteobacteria	Alteromonadales	Alteromonadaceae	Gayadomonas	Gayadomonas joobiniege
5	Bacteria	Proteobacteria	Gammaproteobacteria	Alteromonadales	Alteromonadaceae		
5	Bacteria	Proteobacteria	Gammaproteobacteria	Aeromonadales	Aeromonadaceae	Oceanisphaera	Oceanisphaera psychrotolerans
5	Bacteria	Proteobacteria	Gammaproteobacteria	Aeromonadales	Aeromonadaceae	Oceanimonas	Oceanimonas smirnovii
5	Bacteria	Proteobacteria	Gammaproteobacteria	Aeromonadales	Aeromonadaceae	Aeromonas	Aeromonas finlandiensis
5	Bacteria	Proteobacteria	Candidatus Muproteobacteria	Candidatus Muproteobacteria bacterium RBG_16_65_31			
5	Bacteria	Proteobacteria	Betaproteobacteria	unclassified Betaproteobacteria	unclassified Betaproteobacteria (miscellaneous)	Betaproteobacteria bacterium RBG_19FT_COMBO_58_11	
5	Bacteria	Proteobacteria	Betaproteobacteria	Rhodocyclales	Rhodocyclaceae	Sterolibacterium	Sterolibacterium denitrificans
5	Bacteria	Proteobacteria	Betaproteobacteria	Nitrosomonadales	Nitrosomonadaceae	Nitrosomonas	Nitrosomonas oligotropha
5	Bacteria	Proteobacteria	Betaproteobacteria	Nitrosomonadales	Nitrosomonadaceae	Nitrosomonas	Nitrosomonas cryotolerans
5	Bacteria	Proteobacteria	Betaproteobacteria	Neisseriales	Neisseriaceae	Vitreoscilla	Vitreoscilla stercoraria
5	Bacteria	Proteobacteria	Betaproteobacteria	Neisseriales	Neisseriaceae	Snodgrassella	Snodgrassella sp. CFCC 13594
5	Bacteria	Proteobacteria	Betaproteobacteria	Neisseriales	Neisseriaceae	Kingella	Kingella oralis
5	Bacteria	Proteobacteria	Betaproteobacteria	Neisseriales	Neisseriaceae	Kingella	Kingella kingae
5	Bacteria	Proteobacteria	Betaproteobacteria	Neisseriales	Neisseriaceae	Bergeriella	Bergeriella denitrificans
5	Bacteria	Proteobacteria	Betaproteobacteria	Neisseriales	Chromobacteriaceae	Chromobacterium group	Chromobacterium
5	Bacteria	Proteobacteria	Betaproteobacteria	Hydrogenophilales	Hydrogenophilaceae	Thiobacillus	Thiobacillus sp. 65-1059
5	Bacteria	Proteobacteria	Betaproteobacteria	Gallionellales	unclassified Gallionellales	Gallionellales bacterium RIFCSPHIGHO2_02_FULL_57_16	
5	Bacteria	Proteobacteria	Betaproteobacteria	Burkholderiales	unclassified Burkholderiales	unclassified Burkholderiales (miscellaneous)	Burkholderiales bacterium YL45
5	Bacteria	Proteobacteria	Betaproteobacteria	Burkholderiales	unclassified Burkholderiales	unclassified Burkholderiales (miscellaneous)	Burkholderiales bacterium 66-5
5	Bacteria	Proteobacteria	Betaproteobacteria	Burkholderiales	Sutterellaceae	Sutterella	environmental samples
5	Bacteria	Proteobacteria	Betaproteobacteria	Burkholderiales	Sutterellaceae	Sutterella	Sutterella parvirubra
5	Bacteria	Proteobacteria	Betaproteobacteria	Burkholderiales	Sutterellaceae	Parasutterella	Parasutterella excrementihominis
5	Bacteria	Proteobacteria	Betaproteobacteria	Burkholderiales	Oxalobacteraceae	Pseudoduganella	Pseudoduganella violaceinigra
5	Bacteria	Proteobacteria	Betaproteobacteria	Burkholderiales	Oxalobacteraceae	Massilia	Massilia sp. JS1662
5	Bacteria	Proteobacteria	Betaproteobacteria	Burkholderiales	Oxalobacteraceae	Herbaspirillum	Herbaspirillum rhizosphaerae
5	Bacteria	Proteobacteria	Betaproteobacteria	Burkholderiales	Oxalobacteraceae	Herbaspirillum	Herbaspirillum chlorophenolicum
5	Bacteria	Proteobacteria	Betaproteobacteria	Burkholderiales	Oxalobacteraceae	Duganella	Duganella sp. Leaf61
5	Bacteria	Proteobacteria	Betaproteobacteria	Burkholderiales	Comamonadaceae	Alicycliphilus	Alicycliphilus denitrificans
5	Bacteria	Proteobacteria	Betaproteobacteria	Burkholderiales	Comamonadaceae	Acidovorax	Acidovorax oryzae
5	Bacteria	Proteobacteria	Betaproteobacteria	Burkholderiales	Comamonadaceae	Acidovorax	Acidovorax delafieldii
5	Bacteria	Proteobacteria	Betaproteobacteria	Burkholderiales	Burkholderiaceae	Paraburkholderia	Paraburkholderia tropica
5	Bacteria	Proteobacteria	Betaproteobacteria	Burkholderiales	Burkholderiaceae	Paraburkholderia	Paraburkholderia rhizoxinica
5	Bacteria	Proteobacteria	Betaproteobacteria	Burkholderiales	Burkholderiaceae	Paraburkholderia	Paraburkholderia ferrariae
5	Bacteria	Proteobacteria	Betaproteobacteria	Burkholderiales	Burkholderiaceae	Paraburkholderia	Paraburkholderia acidipaludis
5	Bacteria	Proteobacteria	Betaproteobacteria	Burkholderiales	Burkholderiaceae	Paraburkholderia	Candidatus Paraburkholderia calva
5	Bacteria	Proteobacteria	Betaproteobacteria	Burkholderiales	Burkholderiaceae	Cupriavidus	Cupriavidus sp. UYPR2.512
5	Bacteria	Proteobacteria	Betaproteobacteria	Burkholderiales	Burkholderiaceae	Cupriavidus	Cupriavidus sp. UYMMa02A
5	Bacteria	Proteobacteria	Betaproteobacteria	Burkholderiales	Burkholderiaceae	Caballeronia	Caballeronia udeis
5	Bacteria	Proteobacteria	Betaproteobacteria	Burkholderiales	Burkholderiaceae	Caballeronia	Caballeronia glathei
5	Bacteria	Proteobacteria	Betaproteobacteria	Burkholderiales	Burkholderiaceae	Burkholderia	Candidatus Burkholderia verschuerenii
5	Bacteria	Proteobacteria	Betaproteobacteria	Burkholderiales	Burkholderiaceae	Burkholderia	Burkholderia temeraria
5	Bacteria	Proteobacteria	Betaproteobacteria	Burkholderiales	Burkholderiaceae	Burkholderia	Burkholderia sp. TNe-862
5	Bacteria	Proteobacteria	Betaproteobacteria	Burkholderiales	Burkholderiaceae	Burkholderia	Burkholderia sp. JPY251
5	Bacteria	Proteobacteria	Betaproteobacteria	Burkholderiales	Burkholderiaceae	Burkholderia	Burkholderia pedi
5	Bacteria	Proteobacteria	Betaproteobacteria	Burkholderiales	Burkholderiaceae	Burkholderia	Burkholderia cepacia complex
5	Bacteria	Proteobacteria	Betaproteobacteria	Burkholderiales	Burkholderiaceae	Burkholderia	Burkholderia cepacia complex
5	Bacteria	Proteobacteria	Betaproteobacteria	Burkholderiales	Burkholderiaceae	Burkholderia	Burkholderia cepacia complex
5	Bacteria	Proteobacteria	Betaproteobacteria	Burkholderiales	Burkholderiaceae	Burkholderia	Burkholderia arationis
5	Bacteria	Proteobacteria	Betaproteobacteria	Burkholderiales	Alcaligenaceae	Taylorella	Taylorella asinigenitalis
5	Bacteria	Proteobacteria	Betaproteobacteria	Burkholderiales	Alcaligenaceae	Brackiella	Brackiella oedipodis
5	Bacteria	Proteobacteria	Betaproteobacteria	Burkholderiales	Alcaligenaceae	Achromobacter	Achromobacter sp. LC458
5	Bacteria	Proteobacteria	Betaproteobacteria	Burkholderiales	Alcaligenaceae	Achromobacter	Achromobacter sp. DH1f
5	Bacteria	Proteobacteria	Betaproteobacteria	Burkholderiales	Alcaligenaceae	Achromobacter	Achromobacter sp. 2789STDY5608625
5	Bacteria	Proteobacteria	Alphaproteobacteria	unclassified Alphaproteobacteria	unclassified Alphaproteobacteria (miscellaneous)	Alphaproteobacteria bacterium 64-6	
5	Bacteria	Proteobacteria	Alphaproteobacteria	unclassified Alphaproteobacteria	unclassified Alphaproteobacteria (miscellaneous)	Alphaproteobacteria bacterium 41-28	
5	Bacteria	Proteobacteria	Alphaproteobacteria	Sphingomonadales	Sphingomonadaceae	Sphingopyxis	Sphingopyxis sp. 65-8
5	Bacteria	Proteobacteria	Alphaproteobacteria	Sphingomonadales	Sphingomonadaceae	Sphingomonas	Sphingomonas sp. Leaf67
5	Bacteria	Proteobacteria	Alphaproteobacteria	Sphingomonadales	Sphingomonadaceae	Sphingomonas	Sphingomonas sp. DC-6
5	Bacteria	Proteobacteria	Alphaproteobacteria	Sphingomonadales	Sphingomonadaceae	Sphingomonas	Sphingomonas sp. CCH9-H8
5	Bacteria	Proteobacteria	Alphaproteobacteria	Sphingomonadales	Sphingomonadaceae	Sphingomonas	Sphingomonas paucimobilis
5	Bacteria	Proteobacteria	Alphaproteobacteria	Sphingomonadales	Sphingomonadaceae	Sphingomonas	Sphingomonas melonis
5	Bacteria	Proteobacteria	Alphaproteobacteria	Sphingomonadales	Sphingomonadaceae	Sphingobium	Sphingobium sp. DC-2
5	Bacteria	Proteobacteria	Alphaproteobacteria	Sphingomonadales	Sphingomonadaceae	Sphingobium	Sphingobium abikonense
5	Bacteria	Proteobacteria	Alphaproteobacteria	Sphingomonadales	Sphingomonadaceae	Novosphingobium	Novosphingobium tardaugens
5	Bacteria	Proteobacteria	Alphaproteobacteria	Sphingomonadales	Sphingomonadaceae	Novosphingobium	Novosphingobium lindaniclasticum
5	Bacteria	Proteobacteria	Alphaproteobacteria	Rickettsiales	Anaplasmataceae	Ehrlichia	unclassified Ehrlichia
5	Bacteria	Proteobacteria	Alphaproteobacteria	Rickettsiales	Anaplasmataceae	Ehrlichia	canis group
5	Bacteria	Proteobacteria	Alphaproteobacteria	Rhodospirillales	Rhodospirillaceae	Phaeospirillum	Phaeospirillum molischianum
5	Bacteria	Proteobacteria	Alphaproteobacteria	Rhodospirillales	Rhodospirillaceae	Magnetospirillum	Magnetospirillum sp. ME-1
5	Bacteria	Proteobacteria	Alphaproteobacteria	Rhodospirillales	Rhodospirillaceae	Azospirillum	Azospirillum brasilense
5	Bacteria	Proteobacteria	Alphaproteobacteria	Rhodospirillales	Acetobacteraceae	Tanticharoenia	Tanticharoenia sakaeratensis
5	Bacteria	Proteobacteria	Alphaproteobacteria	Rhodospirillales	Acetobacteraceae	Commensalibacter	Commensalibacter intestini
5	Bacteria	Proteobacteria	Alphaproteobacteria	Rhodospirillales	Acetobacteraceae	Acetobacter	Acetobacter syzygii
5	Bacteria	Proteobacteria	Alphaproteobacteria	Rhodobacterales	Rhodobacteraceae	Sulfitobacter	Sulfitobacter pontiacus
5	Bacteria	Proteobacteria	Alphaproteobacteria	Rhodobacterales	Rhodobacteraceae	Sulfitobacter	Sulfitobacter dubius
5	Bacteria	Proteobacteria	Alphaproteobacteria	Rhodobacterales	Rhodobacteraceae	Ruegeria	Ruegeria sp. ZGT118
5	Bacteria	Proteobacteria	Alphaproteobacteria	Rhodobacterales	Rhodobacteraceae	Ruegeria	Ruegeria sp. TM1040
5	Bacteria	Proteobacteria	Alphaproteobacteria	Rhodobacterales	Rhodobacteraceae	Roseobacter	Roseobacter sp. MED193
5	Bacteria	Proteobacteria	Alphaproteobacteria	Rhodobacterales	Rhodobacteraceae	Pseudophaeobacter	Pseudophaeobacter leonis
5	Bacteria	Proteobacteria	Alphaproteobacteria	Rhodobacterales	Rhodobacteraceae	Nioella	
5	Bacteria	Proteobacteria	Alphaproteobacteria	Rhodobacterales	Rhodobacteraceae	Maribius	Maribius salinus
5	Bacteria	Proteobacteria	Alphaproteobacteria	Rhodobacterales	Rhodobacteraceae	Maribius	Maribius pelagius
5	Bacteria	Proteobacteria	Alphaproteobacteria	Rhodobacterales	Rhodobacteraceae	Mameliella	Mameliella alba
5	Bacteria	Proteobacteria	Alphaproteobacteria	Rhodobacterales	Rhodobacteraceae	Loktanella	Loktanella sp. 3ANDIMAR09
5	Bacteria	Proteobacteria	Alphaproteobacteria	Rhodobacterales	Rhodobacteraceae	Leisingera	Leisingera methylohalidivorans
5	Bacteria	Proteobacteria	Alphaproteobacteria	Rhodobacterales	Hyphomonadaceae	Oceanicaulis	Oceanicaulis alexandrii
5	Bacteria	Proteobacteria	Alphaproteobacteria	Rhodobacterales	Hyphomonadaceae	Maricaulis	Maricaulis sp. W15
5	Bacteria	Proteobacteria	Alphaproteobacteria	Rhodobacterales	Hyphomonadaceae	Hyphomonas	Hyphomonas jannaschiana
5	Bacteria	Proteobacteria	Alphaproteobacteria	Rhodobacterales	Hyphomonadaceae	Hyphomonas	Hyphomonas chukchiensis
5	Bacteria	Proteobacteria	Alphaproteobacteria	Rhizobiales	unclassified Rhizobiales	unclassified Rhizobiales (miscellaneous)	Rhizobiales bacterium GAS188
5	Bacteria	Proteobacteria	Alphaproteobacteria	Rhizobiales	unclassified Rhizobiales	Methyloceanibacter	Methyloceanibacter marginalis
5	Bacteria	Proteobacteria	Alphaproteobacteria	Rhizobiales	Rhizobiaceae	Sinorhizobium/Ensifer group	Sinorhizobium
5	Bacteria	Proteobacteria	Alphaproteobacteria	Rhizobiales	Rhizobiaceae	Rhizobium/Agrobacterium group	Rhizobium
5	Bacteria	Proteobacteria	Alphaproteobacteria	Rhizobiales	Rhizobiaceae	Rhizobium/Agrobacterium group	Rhizobium
5	Bacteria	Proteobacteria	Alphaproteobacteria	Rhizobiales	Rhizobiaceae	Rhizobium/Agrobacterium group	Rhizobium
5	Bacteria	Proteobacteria	Alphaproteobacteria	Rhizobiales	Rhizobiaceae	Rhizobium/Agrobacterium group	Rhizobium
5	Bacteria	Proteobacteria	Alphaproteobacteria	Rhizobiales	Rhizobiaceae	Rhizobium/Agrobacterium group	Rhizobium
5	Bacteria	Proteobacteria	Alphaproteobacteria	Rhizobiales	Rhizobiaceae	Rhizobium/Agrobacterium group	Rhizobium
5	Bacteria	Proteobacteria	Alphaproteobacteria	Rhizobiales	Rhizobiaceae	Rhizobium/Agrobacterium group	Rhizobium
5	Bacteria	Proteobacteria	Alphaproteobacteria	Rhizobiales	Rhizobiaceae	Rhizobium/Agrobacterium group	Rhizobium
5	Bacteria	Proteobacteria	Alphaproteobacteria	Rhizobiales	Rhizobiaceae	Rhizobium/Agrobacterium group	Rhizobium
5	Bacteria	Proteobacteria	Alphaproteobacteria	Rhizobiales	Rhizobiaceae	Rhizobium/Agrobacterium group	Rhizobium
5	Bacteria	Proteobacteria	Alphaproteobacteria	Rhizobiales	Rhizobiaceae	Rhizobium/Agrobacterium group	Agrobacterium
5	Bacteria	Proteobacteria	Alphaproteobacteria	Rhizobiales	Phyllobacteriaceae	Mesorhizobium	Mesorhizobium sp. UASWS1009
5	Bacteria	Proteobacteria	Alphaproteobacteria	Rhizobiales	Phyllobacteriaceae	Mesorhizobium	Mesorhizobium sp. LNHC252B00
5	Bacteria	Proteobacteria	Alphaproteobacteria	Rhizobiales	Phyllobacteriaceae	Mesorhizobium	Mesorhizobium sp. LNHC229A00
5	Bacteria	Proteobacteria	Alphaproteobacteria	Rhizobiales	Phyllobacteriaceae	Mesorhizobium	Mesorhizobium sp. L2C084A000
5	Bacteria	Proteobacteria	Alphaproteobacteria	Rhizobiales	Phyllobacteriaceae	Mesorhizobium	Mesorhizobium sp. L103C119B0
5	Bacteria	Proteobacteria	Alphaproteobacteria	Rhizobiales	Phyllobacteriaceae	Mesorhizobium	Mesorhizobium sp. AA22
5	Bacteria	Proteobacteria	Alphaproteobacteria	Rhizobiales	Phyllobacteriaceae	Mesorhizobium	Mesorhizobium sp. 61-13
5	Bacteria	Proteobacteria	Alphaproteobacteria	Rhizobiales	Phyllobacteriaceae	Mesorhizobium	Mesorhizobium opportunistum
5	Bacteria	Proteobacteria	Alphaproteobacteria	Rhizobiales	Phyllobacteriaceae	Mesorhizobium	Mesorhizobium metallidurans
5	Bacteria	Proteobacteria	Alphaproteobacteria	Rhizobiales	Phyllobacteriaceae	Hoeflea	
5	Bacteria	Proteobacteria	Alphaproteobacteria	Rhizobiales	Methylobacteriaceae	Methylobacterium	Methylobacterium sp. SCN 67-24
5	Bacteria	Proteobacteria	Alphaproteobacteria	Rhizobiales	Methylobacteriaceae	Methylobacterium	Methylobacterium extorquens group
5	Bacteria	Proteobacteria	Alphaproteobacteria	Rhizobiales	Hyphomicrobiaceae	Devosia	Devosia sp. Root436
5	Bacteria	Proteobacteria	Alphaproteobacteria	Rhizobiales	Brucellaceae	Ochrobactrum	Ochrobactrum intermedium
5	Bacteria	Proteobacteria	Alphaproteobacteria	Rhizobiales	Brucellaceae	Ochrobactrum	Ochrobactrum anthropi
5	Bacteria	Proteobacteria	Alphaproteobacteria	Rhizobiales	Bradyrhizobiaceae	Bradyrhizobium	Bradyrhizobium sp. STM 3809
5	Bacteria	Proteobacteria	Alphaproteobacteria	Rhizobiales	Bradyrhizobiaceae	Bradyrhizobium	Bradyrhizobium sp. ORS 285
5	Bacteria	Proteobacteria	Alphaproteobacteria	Rhizobiales	Bradyrhizobiaceae	Bradyrhizobium	Bradyrhizobium sp. NFR13
5	Bacteria	Proteobacteria	Alphaproteobacteria	Rhizobiales	Bradyrhizobiaceae	Bradyrhizobium	Bradyrhizobium sp. Cp5.3
5	Bacteria	Proteobacteria	Alphaproteobacteria	Rhizobiales	Bradyrhizobiaceae	Bradyrhizobium	Bradyrhizobium sp. AS23.2
5	Bacteria	Proteobacteria	Alphaproteobacteria	Rhizobiales	Bradyrhizobiaceae	Bosea	Bosea lathyri
5	Bacteria	Proteobacteria	Alphaproteobacteria	Rhizobiales	Bradyrhizobiaceae	Afipia	Afipia sp. GAS231
5	Bacteria	Proteobacteria	Alphaproteobacteria	Rhizobiales	Bartonellaceae	Bartonella	Bartonella queenslandensis
5	Bacteria	Proteobacteria	Alphaproteobacteria	Rhizobiales	Aurantimonadaceae	Aurantimonas	Aureimonas jatrophae
5	Bacteria	Proteobacteria	Alphaproteobacteria	Kiloniellales	Kiloniellaceae	Kiloniella	Kiloniella litopenaei
5	Bacteria	Proteobacteria	Alphaproteobacteria	Caulobacterales	unclassified Caulobacterales	Caulobacterales bacterium RIFOXYB1_FULL_67_16	
5	Bacteria	Proteobacteria	Alphaproteobacteria	Caulobacterales	Caulobacteraceae	Caulobacter	Caulobacter sp. K31
5	Bacteria	PVC group	Verrucomicrobia	unclassified Verrucomicrobia	unclassified Verrucomicrobia (miscellaneous)	Verrucomicrobia bacterium SCN 57-15	
5	Bacteria	PVC group	Verrucomicrobia	unclassified Verrucomicrobia	unclassified Verrucomicrobia (miscellaneous)	Verrucomicrobia bacterium GWF2_62_7	
5	Bacteria	PVC group	Verrucomicrobia	unclassified Verrucomicrobia	unclassified Verrucomicrobia (miscellaneous)	Verrucomicrobia bacterium GWC2_42_7	
5	Bacteria	PVC group	Verrucomicrobia	Verrucomicrobiae	Verrucomicrobiales	Verrucomicrobiaceae	Verrucomicrobium
5	Bacteria	PVC group	Verrucomicrobia	Verrucomicrobiae	Verrucomicrobiales	Verrucomicrobiaceae	Verrucomicrobium
5	Bacteria	PVC group	Verrucomicrobia	Spartobacteria	Terrimicrobium	Terrimicrobium sacchariphilum	
5	Bacteria	PVC group	Verrucomicrobia	Opitutae	Opitutales	Opitutaceae	unclassified Opitutaceae
5	Bacteria	PVC group	Planctomycetes	unclassified Planctomycetes	Planctomycetes bacterium RBG_16_43_13		
5	Bacteria	PVC group	Planctomycetes	unclassified Planctomycetes	Planctomycetes bacterium RBG_13_46_10		
5	Bacteria	PVC group	Planctomycetes	unclassified Planctomycetes	Planctomycetes bacterium RBG_13_44_8b		
5	Bacteria	PVC group	Planctomycetes	unclassified Planctomycetes	Planctomycetes bacterium DG_20		
5	Bacteria	PVC group	Planctomycetes	Planctomycetia	Candidatus Brocadiales	Candidatus Brocadiaceae	Candidatus Scalindua
5	Bacteria	PVC group	Planctomycetes	Planctomycetia	Candidatus Brocadiales	Candidatus Brocadiaceae	Candidatus Kuenenia
5	Bacteria	PVC group	Planctomycetes	Phycisphaerae	unclassified Phycisphaerae	Phycisphaerae bacterium SM-Chi-D1	
5	Bacteria	PVC group	Lentisphaerae	unclassified Lentisphaerae	unclassified Lentisphaerae (miscellaneous)	Lentisphaerae bacterium RIFOXYC12_FULL_60_16	
5	Bacteria	PVC group	Chlamydiae	Chlamydiia	Parachlamydiales	Parachlamydiaceae	Parachlamydia
5	Bacteria	PVC group	Chlamydiae	Chlamydiia	Chlamydiales	Chlamydiaceae	Chlamydia/Chlamydophila group
5	Bacteria	PVC group	Candidatus Omnitrophica	unclassified Candidatus Omnitrophica	Omnitrophica WOR_2 bacterium SM23_72		
5	Bacteria	PVC group	Candidatus Omnitrophica	unclassified Candidatus Omnitrophica	Omnitrophica WOR_2 bacterium RIFCSPHIGHO2_02_FULL_68_15		
5	Bacteria	PVC group	Candidatus Omnitrophica	unclassified Candidatus Omnitrophica	Candidatus Omnitrophica bacterium CG1_02_49_10		
5	Bacteria	Nitrospirae	unclassified Nitrospirae	Nitrospirae bacterium RBG_13_43_8			
5	Bacteria	Nitrospirae	unclassified Nitrospirae	Nitrospirae bacterium GWC2_56_14			
5	Bacteria	Nitrospirae	Nitrospira	unclassified Nitrospira	Nitrospira bacterium SG8_35_4		
5	Bacteria	Nitrospinae/Tectomicrobia group	Nitrospinae	Nitrospinia	Nitrospinales	Nitrospinaceae	Nitrospina
5	Bacteria	FCB group	candidate division Zixibacteria	candidate division Zixibacteria bacterium RBG_16_48_11			
5	Bacteria	FCB group	Gemmatimonadetes	unclassified Gemmatimonadetes	Gemmatimonadetes bacterium RBG_16_66_8		
5	Bacteria	FCB group	Gemmatimonadetes	unclassified Gemmatimonadetes	Gemmatimonadetes bacterium 13_2_20CM_2_69_23		
5	Bacteria	FCB group	Candidatus Aegiribacteria	Candidatus Aegiribacteria bacterium MLS_C			
5	Bacteria	FCB group	Bacteroidetes/Chlorobi group	Chlorobi	unclassified Chlorobi	Chlorobi bacterium NICIL-2	
5	Bacteria	FCB group	Bacteroidetes/Chlorobi group	Chlorobi	Chlorobia	Chlorobiales	Chlorobiaceae
5	Bacteria	FCB group	Bacteroidetes/Chlorobi group	Chlorobi	Chlorobia	Chlorobiales	Chlorobiaceae
5	Bacteria	FCB group	Bacteroidetes/Chlorobi group	Bacteroidetes	unclassified Bacteroidetes	unclassified Bacteroidetes (miscellaneous)	Bacteroidetes bacterium UKL13-3
5	Bacteria	FCB group	Bacteroidetes/Chlorobi group	Bacteroidetes	unclassified Bacteroidetes	unclassified Bacteroidetes (miscellaneous)	Bacteroidetes bacterium RBG_13_43_22
5	Bacteria	FCB group	Bacteroidetes/Chlorobi group	Bacteroidetes	unclassified Bacteroidetes	unclassified Bacteroidetes (miscellaneous)	Bacteroidetes bacterium MedPE-SWsnd-G1
5	Bacteria	FCB group	Bacteroidetes/Chlorobi group	Bacteroidetes	unclassified Bacteroidetes	unclassified Bacteroidetes (miscellaneous)	Bacteroidetes bacterium 37-13
5	Bacteria	FCB group	Bacteroidetes/Chlorobi group	Bacteroidetes	Sphingobacteriia	Sphingobacteriales	Sphingobacteriaceae
5	Bacteria	FCB group	Bacteroidetes/Chlorobi group	Bacteroidetes	Sphingobacteriia	Sphingobacteriales	Sphingobacteriaceae
5	Bacteria	FCB group	Bacteroidetes/Chlorobi group	Bacteroidetes	Sphingobacteriia	Sphingobacteriales	Sphingobacteriaceae
5	Bacteria	FCB group	Bacteroidetes/Chlorobi group	Bacteroidetes	Sphingobacteriia	Sphingobacteriales	Sphingobacteriaceae
5	Bacteria	FCB group	Bacteroidetes/Chlorobi group	Bacteroidetes	Sphingobacteriia	Sphingobacteriales	Sphingobacteriaceae
5	Bacteria	FCB group	Bacteroidetes/Chlorobi group	Bacteroidetes	Sphingobacteriia	Sphingobacteriales	Sphingobacteriaceae
5	Bacteria	FCB group	Bacteroidetes/Chlorobi group	Bacteroidetes	Sphingobacteriia	Sphingobacteriales	Sphingobacteriaceae
5	Bacteria	FCB group	Bacteroidetes/Chlorobi group	Bacteroidetes	Sphingobacteriia	Sphingobacteriales	Sphingobacteriaceae
5	Bacteria	FCB group	Bacteroidetes/Chlorobi group	Bacteroidetes	Sphingobacteriia	Sphingobacteriales	Sphingobacteriaceae
5	Bacteria	FCB group	Bacteroidetes/Chlorobi group	Bacteroidetes	Sphingobacteriia	Sphingobacteriales	Sphingobacteriaceae
5	Bacteria	FCB group	Bacteroidetes/Chlorobi group	Bacteroidetes	Sphingobacteriia	Sphingobacteriales	Sphingobacteriaceae
5	Bacteria	FCB group	Bacteroidetes/Chlorobi group	Bacteroidetes	Saprospiria	Saprospirales	Saprospiraceae
5	Bacteria	FCB group	Bacteroidetes/Chlorobi group	Bacteroidetes	Flavobacteriia	unclassified Flavobacteriia	Flavobacteriia bacterium 40-80
5	Bacteria	FCB group	Bacteroidetes/Chlorobi group	Bacteroidetes	Flavobacteriia	Flavobacteriales	Flavobacteriaceae
5	Bacteria	FCB group	Bacteroidetes/Chlorobi group	Bacteroidetes	Flavobacteriia	Flavobacteriales	Flavobacteriaceae
5	Bacteria	FCB group	Bacteroidetes/Chlorobi group	Bacteroidetes	Flavobacteriia	Flavobacteriales	Flavobacteriaceae
5	Bacteria	FCB group	Bacteroidetes/Chlorobi group	Bacteroidetes	Flavobacteriia	Flavobacteriales	Flavobacteriaceae
5	Bacteria	FCB group	Bacteroidetes/Chlorobi group	Bacteroidetes	Flavobacteriia	Flavobacteriales	Flavobacteriaceae
5	Bacteria	FCB group	Bacteroidetes/Chlorobi group	Bacteroidetes	Flavobacteriia	Flavobacteriales	Flavobacteriaceae
5	Bacteria	FCB group	Bacteroidetes/Chlorobi group	Bacteroidetes	Flavobacteriia	Flavobacteriales	Flavobacteriaceae
5	Bacteria	FCB group	Bacteroidetes/Chlorobi group	Bacteroidetes	Flavobacteriia	Flavobacteriales	Flavobacteriaceae
5	Bacteria	FCB group	Bacteroidetes/Chlorobi group	Bacteroidetes	Flavobacteriia	Flavobacteriales	Flavobacteriaceae
5	Bacteria	FCB group	Bacteroidetes/Chlorobi group	Bacteroidetes	Flavobacteriia	Flavobacteriales	Flavobacteriaceae
5	Bacteria	FCB group	Bacteroidetes/Chlorobi group	Bacteroidetes	Flavobacteriia	Flavobacteriales	Flavobacteriaceae
5	Bacteria	FCB group	Bacteroidetes/Chlorobi group	Bacteroidetes	Flavobacteriia	Flavobacteriales	Flavobacteriaceae
5	Bacteria	FCB group	Bacteroidetes/Chlorobi group	Bacteroidetes	Flavobacteriia	Flavobacteriales	Cryomorphaceae
5	Bacteria	FCB group	Bacteroidetes/Chlorobi group	Bacteroidetes	Flavobacteriia	Flavobacteriales	Crocinitomicaceae
5	Bacteria	FCB group	Bacteroidetes/Chlorobi group	Bacteroidetes	Cytophagia	Cytophagales	unclassified Cytophagales
5	Bacteria	FCB group	Bacteroidetes/Chlorobi group	Bacteroidetes	Cytophagia	Cytophagales	Persicobacteraceae
5	Bacteria	FCB group	Bacteroidetes/Chlorobi group	Bacteroidetes	Cytophagia	Cytophagales	Hymenobacteraceae
5	Bacteria	FCB group	Bacteroidetes/Chlorobi group	Bacteroidetes	Cytophagia	Cytophagales	Hymenobacteraceae
5	Bacteria	FCB group	Bacteroidetes/Chlorobi group	Bacteroidetes	Cytophagia	Cytophagales	Hymenobacteraceae
5	Bacteria	FCB group	Bacteroidetes/Chlorobi group	Bacteroidetes	Cytophagia	Cytophagales	Hymenobacteraceae
5	Bacteria	FCB group	Bacteroidetes/Chlorobi group	Bacteroidetes	Cytophagia	Cytophagales	Hymenobacteraceae
5	Bacteria	FCB group	Bacteroidetes/Chlorobi group	Bacteroidetes	Cytophagia	Cytophagales	Hymenobacteraceae
5	Bacteria	FCB group	Bacteroidetes/Chlorobi group	Bacteroidetes	Cytophagia	Cytophagales	Cytophagaceae
5	Bacteria	FCB group	Bacteroidetes/Chlorobi group	Bacteroidetes	Cytophagia	Cytophagales	Cytophagaceae
5	Bacteria	FCB group	Bacteroidetes/Chlorobi group	Bacteroidetes	Cytophagia	Cytophagales	Cytophagaceae
5	Bacteria	FCB group	Bacteroidetes/Chlorobi group	Bacteroidetes	Cytophagia	Cytophagales	Cytophagaceae
5	Bacteria	FCB group	Bacteroidetes/Chlorobi group	Bacteroidetes	Cytophagia	Cytophagales	Cytophagaceae
5	Bacteria	FCB group	Bacteroidetes/Chlorobi group	Bacteroidetes	Cytophagia	Cytophagales	Cytophagaceae
5	Bacteria	FCB group	Bacteroidetes/Chlorobi group	Bacteroidetes	Cytophagia	Cytophagales	Cyclobacteriaceae
5	Bacteria	FCB group	Bacteroidetes/Chlorobi group	Bacteroidetes	Cytophagia	Cytophagales	Cyclobacteriaceae
5	Bacteria	FCB group	Bacteroidetes/Chlorobi group	Bacteroidetes	Cytophagia	Cytophagales	Cyclobacteriaceae
5	Bacteria	FCB group	Bacteroidetes/Chlorobi group	Bacteroidetes	Cytophagia	Cytophagales	Cyclobacteriaceae
5	Bacteria	FCB group	Bacteroidetes/Chlorobi group	Bacteroidetes	Cytophagia	Cytophagales	Cyclobacteriaceae
5	Bacteria	FCB group	Bacteroidetes/Chlorobi group	Bacteroidetes	Cytophagia	Cytophagales	
5	Bacteria	FCB group	Bacteroidetes/Chlorobi group	Bacteroidetes	Chitinophagia	Chitinophagales	Chitinophagaceae
5	Bacteria	FCB group	Bacteroidetes/Chlorobi group	Bacteroidetes	Chitinophagia	Chitinophagales	Chitinophagaceae
5	Bacteria	FCB group	Bacteroidetes/Chlorobi group	Bacteroidetes	Chitinophagia	Chitinophagales	Chitinophagaceae
5	Bacteria	FCB group	Bacteroidetes/Chlorobi group	Bacteroidetes	Chitinophagia	Chitinophagales	Chitinophagaceae
5	Bacteria	FCB group	Bacteroidetes/Chlorobi group	Bacteroidetes	Chitinophagia	Chitinophagales	Chitinophagaceae
5	Bacteria	FCB group	Bacteroidetes/Chlorobi group	Bacteroidetes	Chitinophagia	Chitinophagales	Chitinophagaceae
5	Bacteria	FCB group	Bacteroidetes/Chlorobi group	Bacteroidetes	Chitinophagia	Chitinophagales	Chitinophagaceae
5	Bacteria	FCB group	Bacteroidetes/Chlorobi group	Bacteroidetes	Bacteroidia	Bacteroidales	unclassified Bacteroidales
5	Bacteria	FCB group	Bacteroidetes/Chlorobi group	Bacteroidetes	Bacteroidia	Bacteroidales	Rikenellaceae
5	Bacteria	FCB group	Bacteroidetes/Chlorobi group	Bacteroidetes	Bacteroidia	Bacteroidales	Prevotellaceae
5	Bacteria	FCB group	Bacteroidetes/Chlorobi group	Bacteroidetes	Bacteroidia	Bacteroidales	Prevotellaceae
5	Bacteria	FCB group	Bacteroidetes/Chlorobi group	Bacteroidetes	Bacteroidia	Bacteroidales	Prevotellaceae
5	Bacteria	FCB group	Bacteroidetes/Chlorobi group	Bacteroidetes	Bacteroidia	Bacteroidales	Prevotellaceae
5	Bacteria	FCB group	Bacteroidetes/Chlorobi group	Bacteroidetes	Bacteroidia	Bacteroidales	Prevotellaceae
5	Bacteria	FCB group	Bacteroidetes/Chlorobi group	Bacteroidetes	Bacteroidia	Bacteroidales	Prevotellaceae
5	Bacteria	FCB group	Bacteroidetes/Chlorobi group	Bacteroidetes	Bacteroidia	Bacteroidales	Porphyromonadaceae
5	Bacteria	FCB group	Bacteroidetes/Chlorobi group	Bacteroidetes	Bacteroidia	Bacteroidales	Porphyromonadaceae
5	Bacteria	FCB group	Bacteroidetes/Chlorobi group	Bacteroidetes	Bacteroidia	Bacteroidales	Porphyromonadaceae
5	Bacteria	FCB group	Bacteroidetes/Chlorobi group	Bacteroidetes	Bacteroidia	Bacteroidales	Porphyromonadaceae

5	Bacteria	FCB group	Bacteroidetes/Chlorobi group	Bacteroidetes	Bacteroidia	Bacteroidales	Porphyromonadaceae
5	Bacteria	FCB group	Bacteroidetes/Chlorobi group	Bacteroidetes	Bacteroidia	Bacteroidales	Bacteroidaceae
5	Bacteria	FCB group	Bacteroidetes/Chlorobi group	Bacteroidetes	Bacteroidia	Bacteroidales	Bacteroidaceae
5	Bacteria	FCB group	Bacteroidetes/Chlorobi group	Bacteroidetes	Bacteroidia	Bacteroidales	Bacteroidaceae
5	Bacteria	FCB group	Bacteroidetes/Chlorobi group	Bacteroidetes	Bacteroidia	Bacteroidales	Bacteroidaceae
5	Bacteria	FCB group	Bacteroidetes/Chlorobi group	Bacteroidetes	Bacteroidetes Order II. Incertae sedis	Rhodothermaceae	Rhodothermus
5	Bacteria	FCB group	Bacteroidetes/Chlorobi group	Bacteroidetes	Bacteroidetes Order II. Incertae sedis	Rhodothermaceae	Rhodothermus
5	Bacteria	FCB group	Bacteroidetes/Chlorobi group	Bacteroidetes	Bacteroidetes Order II. Incertae sedis	Rhodothermaceae	Rhodothermus
5	Bacteria	FCB group	Bacteroidetes/Chlorobi group				
5	Bacteria	Elusimicrobia	unclassified Elusimicrobia	Elusimicrobia bacterium RIFCSPLOWO2_01_FULL_59_12			
5	Bacteria	Elusimicrobia	unclassified Elusimicrobia	Elusimicrobia bacterium CG1_02_37_114			
5	Bacteria	Balneolaeota	Balneolia	Balneolales	Balneolaceae	Gracilimonas	Gracilimonas tropica
5	Bacteria	Balneolaeota	Balneolia	Balneolales	Balneolaceae	Balneola	Balneola sp. EhC07
5	Bacteria	Aquificae	Aquificae	Desulfurobacteriales	Desulfurobacteriaceae	Desulfurobacterium	Desulfurobacterium sp. TC5-1
5	Bacteria	Aquificae	Aquificae	Aquificales	Hydrogenothermaceae	Persephonella	Persephonella marina
5	Bacteria	Acidobacteria	unclassified Acidobacteria	unclassified Acidobacteria (miscellaneous)	Acidobacteria bacterium 13_1_40CM_4_69_4		
5	Bacteria	Acidobacteria	unclassified Acidobacteria	unclassified Acidobacteria (miscellaneous)	Acidobacteria bacterium 13_1_20CM_58_21		
5	Bacteria	Acidobacteria	unclassified Acidobacteria	Acidobacteria subdivision 23	Thermoanaerobaculum	Thermoanaerobaculum aquaticum	
5	Bacteria	Acidobacteria	Acidobacteriia	Acidobacteriales	Acidobacteriaceae	unclassified Acidobacteriaceae	Acidobacteriaceae bacterium TAA166
5	Bacteria	Acidobacteria	Acidobacteriia	Acidobacteriales	Acidobacteriaceae	Granulicella	Granulicella pectinivorans
5	Bacteria	Acidobacteria	Acidobacteriia	Acidobacteriales	Acidobacteriaceae	Bryocella	Bryocella elongata
5	Bacteria	Acidobacteria	Acidobacteriia	Acidobacteriales			
5	Archaea	Asgard group	Candidatus Lokiarchaeota	Lokiarchaeum	Lokiarchaeum sp. GC14_75		
4	Eukaryota	Viridiplantae	Chlorophyta	prasinophytes	Mamiellophyceae	Mamiellales	Mamiellaceae
4	Eukaryota	Viridiplantae	Chlorophyta	Chlorophyceae	Sphaeropleales	Sphaeropleaceae	Atractomorpha
4	Eukaryota	Viridiplantae	Chlorophyta	Chlorophyceae	Sphaeropleales	Selenastraceae	Monoraphidium
4	Eukaryota	Stramenopiles	Bacillariophyta	Fragilariophyceae	Fragilariophycidae	Fragilariales	Fragilariaceae
4	Eukaryota	Stramenopiles	Bacillariophyta	Coscinodiscophyceae	Thalassiosirophycidae	Thalassiosirales	Thalassiosiraceae
4	Eukaryota	Opisthokonta	Fungi	Neocallimastigomycota	Neocallimastigomycetes	Neocallimastigales	Neocallimastigaceae
4	Eukaryota	Opisthokonta	Fungi	Mucoromycota	Mucoromycotina	Mucorales	Phycomycetaceae
4	Eukaryota	Opisthokonta	Fungi	Dikarya	Ascomycota	saccharomyceta	Saccharomycotina
4	Eukaryota	Opisthokonta	Fungi	Dikarya	Ascomycota	saccharomyceta	Pezizomycotina
4	Eukaryota	Opisthokonta	Fungi	Dikarya	Ascomycota	saccharomyceta	Pezizomycotina
4	Eukaryota	Opisthokonta	Fungi	Dikarya	Ascomycota	saccharomyceta	Pezizomycotina
4	Eukaryota	Opisthokonta	Fungi	Chytridiomycota	Chytridiomycetes	Rhizophydiales	Rhizophydiales incertae sedis
4	Eukaryota	Opisthokonta	Fungi	Chytridiomycota	Chytridiomycetes	Rhizophydiales	Rhizophydiales incertae sedis
4	Eukaryota	Malawimonadidae	Malawimonas	Malawimonas californiana			
4	Eukaryota	Amoebozoa	Discosea	Longamoebia	Centramoebida	Acanthamoebidae	Acanthamoeba
4	Eukaryota	Alveolata	Ciliophora	Intramacronucleata	Spirotrichea	Stichotrichia	Sporadotrichida
4	Eukaryota	Alveolata	Chromerida	Vitrella	Vitrella brassicaformis	Vitrella brassicaformis CCMP3155	
4	Eukaryota	Alveolata	Apicomplexa	Aconoidasida	Haemosporida	Plasmodiidae	Plasmodium
4	Bacteria	unclassified Bacteria	unclassified Bacteria (miscellaneous)	bacterium SIT5			
4	Bacteria	unclassified Bacteria	unclassified Bacteria (miscellaneous)	bacterium SCN 62-11			
4	Bacteria	unclassified Bacteria	unclassified Bacteria (miscellaneous)	bacterium CG2_30_54_10			
4	Bacteria	unclassified Bacteria	Bacteria candidate phyla	candidate division WOR-3	candidate division WOR_3 bacterium SM23_60		
4	Bacteria	unclassified Bacteria	Bacteria candidate phyla	candidate division TA06	candidate division TA06 bacterium DG_26		
4	Bacteria	unclassified Bacteria	Bacteria candidate phyla	candidate division TA06			
4	Bacteria	unclassified Bacteria	Bacteria candidate phyla	Patescibacteria group	Parcubacteria group	Candidatus Yanofskybacteria	Candidatus Yanofskybacteria bacterium RIFCSPLOWO2_01_FULL_49_25
4	Bacteria	unclassified Bacteria	Bacteria candidate phyla	Patescibacteria group	Parcubacteria group	Candidatus Nomurabacteria	
4	Bacteria	unclassified Bacteria	Bacteria candidate phyla	Patescibacteria group	Parcubacteria group	Candidatus Falkowbacteria	
4	Bacteria	unclassified Bacteria	Bacteria candidate phyla	Patescibacteria group	Microgenomates group	Candidatus Levybacteria	
4	Bacteria	unclassified Bacteria	Bacteria candidate phyla	Patescibacteria group	Microgenomates group	Candidatus Gottesmanbacteria	
4	Bacteria	unclassified Bacteria	Bacteria candidate phyla	Candidatus Rokubacteria	Candidatus Rokubacteria bacterium GWA2_70_23		
4	Bacteria	unclassified Bacteria	Bacteria candidate phyla	Candidatus Rokubacteria	Candidatus Rokubacteria bacterium 13_1_40CM_2_68_8		
4	Bacteria	unclassified Bacteria	Bacteria candidate phyla	Candidatus Peregrinibacteria	Candidatus Peribacteria	unclassified Candidatus Peribacteria	
4	Bacteria	unclassified Bacteria	Bacteria candidate phyla	Candidatus Margulisbacteria	Candidatus Margulisbacteria bacterium GWF2_35_9		
4	Bacteria	unclassified Bacteria	Bacteria candidate phyla	Candidatus Firestonebacteria	Candidatus Firestonebacteria bacterium RIFOXYA2_FULL_40_8		
4	Bacteria	unclassified Bacteria	Bacteria candidate phyla	Candidatus Dependentiae	unclassified Candidatus Dependentiae	candidate division TM6 bacterium GW2011_GWF2_37_49	
4	Bacteria	unclassified Bacteria	Bacteria candidate phyla	Candidatus Dadabacteria	Candidatus Dadabacteria bacterium CSP1-2		
4	Bacteria	unclassified Bacteria	Bacteria candidate phyla	Candidatus Atribacteria	unclassified Atribacteria	Candidatus Atribacteria bacterium CG2_30_33_13	
4	Bacteria	unclassified Bacteria	Bacteria candidate phyla	Candidatus Aminicenantes	unclassified Aminicenantes	Candidatus Aminicenantes bacterium RBG_13_62_12	
4	Bacteria	Thermotogae	Thermotogae	Thermotogales	Thermotogaceae	Thermotoga	
4	Bacteria	Thermotogae	Thermotogae	Petrotogales	Petrotogaceae	Geotoga	Geotoga petraea
4	Bacteria	Thermodesulfobacteria	Thermodesulfobacteria	Thermodesulfobacteriales	Thermodesulfobacteriaceae	Thermosulfurimonas	Thermosulfurimonas dismutans
4	Bacteria	Terrabacteria group	Firmicutes	unclassified Firmicutes sensu stricto	unclassified Firmicutes sensu stricto (miscellaneous)	Firmicutes bacterium ML8_F2	
4	Bacteria	Terrabacteria group	Firmicutes	unclassified Firmicutes sensu stricto	unclassified Firmicutes sensu stricto (miscellaneous)	Firmicutes bacterium ASF500	
4	Bacteria	Terrabacteria group	Firmicutes	unclassified Firmicutes sensu stricto	unclassified Firmicutes sensu stricto (miscellaneous)		
4	Bacteria	Terrabacteria group	Firmicutes	environmental samples	Firmicutes bacterium CAG:83		
4	Bacteria	Terrabacteria group	Firmicutes	environmental samples	Firmicutes bacterium CAG:124		
4	Bacteria	Terrabacteria group	Firmicutes	environmental samples	Firmicutes bacterium CAG:114		
4	Bacteria	Terrabacteria group	Firmicutes	environmental samples	Firmicutes bacterium CAG:103		
4	Bacteria	Terrabacteria group	Firmicutes	Tissierellia	unclassified Tissierellia	Tepidimicrobium	Tepidimicrobium xylanilyticum
4	Bacteria	Terrabacteria group	Firmicutes	Tissierellia	Tissierellales	Tissierellaceae	Tissierella
4	Bacteria	Terrabacteria group	Firmicutes	Tissierellia	Tissierellales	Sporanaerobacter	Sporanaerobacter acetigenes
4	Bacteria	Terrabacteria group	Firmicutes	Tissierellia	Tissierellales	Peptoniphilaceae	Peptoniphilus
4	Bacteria	Terrabacteria group	Firmicutes	Tissierellia	Tissierellales	Peptoniphilaceae	Anaerosphaera
4	Bacteria	Terrabacteria group	Firmicutes	Tissierellia	Tissierellales	Peptoniphilaceae	Anaerococcus
4	Bacteria	Terrabacteria group	Firmicutes	Tissierellia	Tissierellales	Peptoniphilaceae	Anaerococcus
4	Bacteria	Terrabacteria group	Firmicutes	Negativicutes	Selenomonadales	Sporomusaceae	Thermosinus
4	Bacteria	Terrabacteria group	Firmicutes	Negativicutes	Selenomonadales	Selenomonadaceae	Selenomonas
4	Bacteria	Terrabacteria group	Firmicutes	Negativicutes	Acidaminococcales	Acidaminococcaceae	Succiniclasticum
4	Bacteria	Terrabacteria group	Firmicutes	Erysipelotrichia	Erysipelotrichales	Erysipelotrichaceae	Turicibacter
4	Bacteria	Terrabacteria group	Firmicutes	Erysipelotrichia	Erysipelotrichales	Erysipelotrichaceae	Traorella
4	Bacteria	Terrabacteria group	Firmicutes	Clostridia	Thermoanaerobacterales	Thermoanaerobacterales Family III. Incertae Sedis	Thermosediminibacter
4	Bacteria	Terrabacteria group	Firmicutes	Clostridia	Thermoanaerobacterales	Thermoanaerobacteraceae	Thermacetogenium
4	Bacteria	Terrabacteria group	Firmicutes	Clostridia	Thermoanaerobacterales	Thermoanaerobacteraceae	Carboxydothermus
4	Bacteria	Terrabacteria group	Firmicutes	Clostridia	Thermoanaerobacterales	Thermoanaerobacteraceae	Carboxydothermus
4	Bacteria	Terrabacteria group	Firmicutes	Clostridia	Halanaerobiales	Halobacteroidaceae	Orenia
4	Bacteria	Terrabacteria group	Firmicutes	Clostridia	Halanaerobiales	Halanaerobiaceae	Halarsenatibacter
4	Bacteria	Terrabacteria group	Firmicutes	Clostridia	Halanaerobiales	Halanaerobiaceae	Halanaerobium
4	Bacteria	Terrabacteria group	Firmicutes	Clostridia	Clostridiales	unclassified Clostridiales	unclassified Clostridiales (miscellaneous)
4	Bacteria	Terrabacteria group	Firmicutes	Clostridia	Clostridiales	unclassified Clostridiales	unclassified Clostridiales (miscellaneous)
4	Bacteria	Terrabacteria group	Firmicutes	Clostridia	Clostridiales	unclassified Clostridiales	unclassified Clostridiales (miscellaneous)
4	Bacteria	Terrabacteria group	Firmicutes	Clostridia	Clostridiales	unclassified Clostridiales	unclassified Clostridiales (miscellaneous)
4	Bacteria	Terrabacteria group	Firmicutes	Clostridia	Clostridiales	unclassified Clostridiales	unclassified Clostridiales (miscellaneous)
4	Bacteria	Terrabacteria group	Firmicutes	Clostridia	Clostridiales	unclassified Clostridiales	Intestinimonas
4	Bacteria	Terrabacteria group	Firmicutes	Clostridia	Clostridiales	Syntrophomonadaceae	Syntrophomonas
4	Bacteria	Terrabacteria group	Firmicutes	Clostridia	Clostridiales	Syntrophomonadaceae	Dethiobacter
4	Bacteria	Terrabacteria group	Firmicutes	Clostridia	Clostridiales	Ruminococcaceae	Subdoligranulum
4	Bacteria	Terrabacteria group	Firmicutes	Clostridia	Clostridiales	Ruminococcaceae	Ruminiclostridium
4	Bacteria	Terrabacteria group	Firmicutes	Clostridia	Clostridiales	Ruminococcaceae	Ruminiclostridium
4	Bacteria	Terrabacteria group	Firmicutes	Clostridia	Clostridiales	Ruminococcaceae	Massilimaliae
4	Bacteria	Terrabacteria group	Firmicutes	Clostridia	Clostridiales	Ruminococcaceae	Anaerotruncus
4	Bacteria	Terrabacteria group	Firmicutes	Clostridia	Clostridiales	Ruminococcaceae	Anaerotruncus
4	Bacteria	Terrabacteria group	Firmicutes	Clostridia	Clostridiales	Ruminococcaceae	Anaeromassilibacillus
4	Bacteria	Terrabacteria group	Firmicutes	Clostridia	Clostridiales	Peptostreptococcaceae	unclassified Peptostreptococcaceae
4	Bacteria	Terrabacteria group	Firmicutes	Clostridia	Clostridiales	Peptostreptococcaceae	unclassified Peptostreptococcaceae
4	Bacteria	Terrabacteria group	Firmicutes	Clostridia	Clostridiales	Peptostreptococcaceae	Tepidibacter
4	Bacteria	Terrabacteria group	Firmicutes	Clostridia	Clostridiales	Peptococcaceae	Thermincola
4	Bacteria	Terrabacteria group	Firmicutes	Clostridia	Clostridiales	Peptococcaceae	Syntrophobotulus
4	Bacteria	Terrabacteria group	Firmicutes	Clostridia	Clostridiales	Peptococcaceae	Desulfurispora
4	Bacteria	Terrabacteria group	Firmicutes	Clostridia	Clostridiales	Peptococcaceae	Desulfosporosinus
4	Bacteria	Terrabacteria group	Firmicutes	Clostridia	Clostridiales	Peptococcaceae	Desulfosporosinus
4	Bacteria	Terrabacteria group	Firmicutes	Clostridia	Clostridiales	Peptococcaceae	Desulfitibacter
4	Bacteria	Terrabacteria group	Firmicutes	Clostridia	Clostridiales	Peptococcaceae	Dehalobacter
4	Bacteria	Terrabacteria group	Firmicutes	Clostridia	Clostridiales	Peptococcaceae	Dehalobacter
4	Bacteria	Terrabacteria group	Firmicutes	Clostridia	Clostridiales	Lachnospiraceae	unclassified Lachnospiraceae
4	Bacteria	Terrabacteria group	Firmicutes	Clostridia	Clostridiales	Lachnospiraceae	unclassified Lachnospiraceae
4	Bacteria	Terrabacteria group	Firmicutes	Clostridia	Clostridiales	Lachnospiraceae	unclassified Lachnospiraceae
4	Bacteria	Terrabacteria group	Firmicutes	Clostridia	Clostridiales	Lachnospiraceae	unclassified Lachnospiraceae
4	Bacteria	Terrabacteria group	Firmicutes	Clostridia	Clostridiales	Lachnospiraceae	unclassified Lachnospiraceae
4	Bacteria	Terrabacteria group	Firmicutes	Clostridia	Clostridiales	Lachnospiraceae	unclassified Lachnospiraceae
4	Bacteria	Terrabacteria group	Firmicutes	Clostridia	Clostridiales	Lachnospiraceae	Oribacterium
4	Bacteria	Terrabacteria group	Firmicutes	Clostridia	Clostridiales	Lachnospiraceae	Lachnoclostridium
4	Bacteria	Terrabacteria group	Firmicutes	Clostridia	Clostridiales	Lachnospiraceae	Lachnoclostridium
4	Bacteria	Terrabacteria group	Firmicutes	Clostridia	Clostridiales	Lachnospiraceae	Lachnoclostridium
4	Bacteria	Terrabacteria group	Firmicutes	Clostridia	Clostridiales	Lachnospiraceae	Hespellia
4	Bacteria	Terrabacteria group	Firmicutes	Clostridia	Clostridiales	Lachnospiraceae	Butyrivibrio
4	Bacteria	Terrabacteria group	Firmicutes	Clostridia	Clostridiales	Heliobacteriaceae	Heliobacterium
4	Bacteria	Terrabacteria group	Firmicutes	Clostridia	Clostridiales	Eubacteriaceae	Eubacterium
4	Bacteria	Terrabacteria group	Firmicutes	Clostridia	Clostridiales	Eubacteriaceae	Eubacterium
4	Bacteria	Terrabacteria group	Firmicutes	Clostridia	Clostridiales	Eubacteriaceae	Eubacterium
4	Bacteria	Terrabacteria group	Firmicutes	Clostridia	Clostridiales	Eubacteriaceae	Eubacterium
4	Bacteria	Terrabacteria group	Firmicutes	Clostridia	Clostridiales	Clostridiales incertae sedis	Clostridiales Family XIII. Incertae Sedis
4	Bacteria	Terrabacteria group	Firmicutes	Clostridia	Clostridiales	Clostridiales incertae sedis	Clostridiales Family XIII. Incertae Sedis
4	Bacteria	Terrabacteria group	Firmicutes	Clostridia	Clostridiales	Clostridiaceae	Proteiniclasticum
4	Bacteria	Terrabacteria group	Firmicutes	Clostridia	Clostridiales	Clostridiaceae	Clostridium
4	Bacteria	Terrabacteria group	Firmicutes	Clostridia	Clostridiales	Clostridiaceae	Clostridium
4	Bacteria	Terrabacteria group	Firmicutes	Clostridia	Clostridiales	Clostridiaceae	Clostridium
4	Bacteria	Terrabacteria group	Firmicutes	Clostridia	Clostridiales	Clostridiaceae	Clostridium
4	Bacteria	Terrabacteria group	Firmicutes	Clostridia	Clostridiales	Clostridiaceae	Clostridium
4	Bacteria	Terrabacteria group	Firmicutes	Clostridia	Clostridiales	Clostridiaceae	Clostridium
4	Bacteria	Terrabacteria group	Firmicutes	Clostridia	Clostridiales	Clostridiaceae	Clostridium
4	Bacteria	Terrabacteria group	Firmicutes	Clostridia	Clostridiales	Clostridiaceae	Clostridium
4	Bacteria	Terrabacteria group	Firmicutes	Clostridia	Clostridiales	Clostridiaceae	Clostridium
4	Bacteria	Terrabacteria group	Firmicutes	Clostridia	Clostridiales	Clostridiaceae	Clostridium
4	Bacteria	Terrabacteria group	Firmicutes	Clostridia	Clostridiales	Clostridiaceae	Clostridium
4	Bacteria	Terrabacteria group	Firmicutes	Clostridia	Clostridiales	Clostridiaceae	Clostridium
4	Bacteria	Terrabacteria group	Firmicutes	Clostridia	Clostridiales	Clostridiaceae	Clostridium
4	Bacteria	Terrabacteria group	Firmicutes	Clostridia	Clostridiales	Clostridiaceae	Clostridium
4	Bacteria	Terrabacteria group	Firmicutes	Clostridia	Clostridiales	Clostridiaceae	Caloranaerobacter
4	Bacteria	Terrabacteria group	Firmicutes	Clostridia	Clostridiales	Clostridiaceae	Butyricicoccus
4	Bacteria	Terrabacteria group	Firmicutes	Clostridia	Clostridiales	Clostridiaceae	Butyricicoccus
4	Bacteria	Terrabacteria group	Firmicutes	Clostridia	Clostridiales	Clostridiaceae	Alkaliphilus
4	Bacteria	Terrabacteria group	Firmicutes	Bacilli	Lactobacillales	Lactobacillaceae	Sharpea
4	Bacteria	Terrabacteria group	Firmicutes	Bacilli	Lactobacillales	Lactobacillaceae	Lactobacillus
4	Bacteria	Terrabacteria group	Firmicutes	Bacilli	Lactobacillales	Enterococcaceae	Enterococcus
4	Bacteria	Terrabacteria group	Firmicutes	Bacilli	Lactobacillales	Carnobacteriaceae	Carnobacterium
4	Bacteria	Terrabacteria group	Firmicutes	Bacilli	Bacillales	unclassified Bacillales	Acidibacillus
4	Bacteria	Terrabacteria group	Firmicutes	Bacilli	Bacillales	Thermoactinomycetaceae	Risungbinella
4	Bacteria	Terrabacteria group	Firmicutes	Bacilli	Bacillales	Thermoactinomycetaceae	Melghirimyces
4	Bacteria	Terrabacteria group	Firmicutes	Bacilli	Bacillales	Thermoactinomycetaceae	Marininema
4	Bacteria	Terrabacteria group	Firmicutes	Bacilli	Bacillales	Thermoactinomycetaceae	Lihuaxuella
4	Bacteria	Terrabacteria group	Firmicutes	Bacilli	Bacillales	Planococcaceae	Jeotgalibacillus
4	Bacteria	Terrabacteria group	Firmicutes	Bacilli	Bacillales	Planococcaceae	
4	Bacteria	Terrabacteria group	Firmicutes	Bacilli	Bacillales	Paenibacillaceae	Paenibacillus
4	Bacteria	Terrabacteria group	Firmicutes	Bacilli	Bacillales	Paenibacillaceae	Paenibacillus
4	Bacteria	Terrabacteria group	Firmicutes	Bacilli	Bacillales	Paenibacillaceae	Paenibacillus
4	Bacteria	Terrabacteria group	Firmicutes	Bacilli	Bacillales	Paenibacillaceae	Paenibacillus
4	Bacteria	Terrabacteria group	Firmicutes	Bacilli	Bacillales	Paenibacillaceae	Paenibacillus
4	Bacteria	Terrabacteria group	Firmicutes	Bacilli	Bacillales	Paenibacillaceae	Brevibacillus
4	Bacteria	Terrabacteria group	Firmicutes	Bacilli	Bacillales	Paenibacillaceae	Aneurinibacillus group
4	Bacteria	Terrabacteria group	Firmicutes	Bacilli	Bacillales	Listeriaceae	Listeria
4	Bacteria	Terrabacteria group	Firmicutes	Bacilli	Bacillales	Bacillaceae	Pontibacillus
4	Bacteria	Terrabacteria group	Firmicutes	Bacilli	Bacillales	Bacillaceae	Lysinibacillus
4	Bacteria	Terrabacteria group	Firmicutes	Bacilli	Bacillales	Bacillaceae	Lentibacillus
4	Bacteria	Terrabacteria group	Firmicutes	Bacilli	Bacillales	Bacillaceae	Halalkalibacillus
4	Bacteria	Terrabacteria group	Firmicutes	Bacilli	Bacillales	Bacillaceae	Bacillus
4	Bacteria	Terrabacteria group	Firmicutes	Bacilli	Bacillales	Bacillaceae	Bacillus
4	Bacteria	Terrabacteria group	Firmicutes	Bacilli	Bacillales	Bacillaceae	Bacillus
4	Bacteria	Terrabacteria group	Firmicutes	Bacilli	Bacillales	Bacillaceae	Bacillus
4	Bacteria	Terrabacteria group	Deinococcus-Thermus	Deinococci	Thermales	Thermaceae	Meiothermus
4	Bacteria	Terrabacteria group	Deinococcus-Thermus	Deinococci	Deinococcales	Deinococcaceae	Deinococcus
4	Bacteria	Terrabacteria group	Deinococcus-Thermus	Deinococci	Deinococcales	Deinococcaceae	Deinococcus
4	Bacteria	Terrabacteria group	Deinococcus-Thermus	Deinococci	Deinococcales	Deinococcaceae	Deinococcus
4	Bacteria	Terrabacteria group	Deinococcus-Thermus	Deinococci	Deinococcales	Deinococcaceae	Deinococcus
4	Bacteria	Terrabacteria group	Deinococcus-Thermus	Deinococci	Deinococcales	Deinococcaceae	Deinococcus
4	Bacteria	Terrabacteria group	Cyanobacteria/Melainabacteria group	Cyanobacteria	Synechococcales	Synechococcaceae	Synechococcus
4	Bacteria	Terrabacteria group	Cyanobacteria/Melainabacteria group	Cyanobacteria	Synechococcales	Synechococcaceae	Cyanobium
4	Bacteria	Terrabacteria group	Cyanobacteria/Melainabacteria group	Cyanobacteria	Synechococcales	Synechococcaceae	Cyanobium
4	Bacteria	Terrabacteria group	Cyanobacteria/Melainabacteria group	Cyanobacteria	Synechococcales	Prochloraceae	Prochlorococcus
4	Bacteria	Terrabacteria group	Cyanobacteria/Melainabacteria group	Cyanobacteria	Synechococcales	Leptolyngbyaceae	Leptolyngbya
4	Bacteria	Terrabacteria group	Cyanobacteria/Melainabacteria group	Cyanobacteria	Synechococcales	Leptolyngbyaceae	Leptolyngbya
4	Bacteria	Terrabacteria group	Cyanobacteria/Melainabacteria group	Cyanobacteria	Synechococcales	Leptolyngbyaceae	Leptolyngbya
4	Bacteria	Terrabacteria group	Cyanobacteria/Melainabacteria group	Cyanobacteria	Synechococcales	Leptolyngbyaceae	Leptolyngbya
4	Bacteria	Terrabacteria group	Cyanobacteria/Melainabacteria group	Cyanobacteria	Synechococcales	Chamaesiphonaceae	Chamaesiphon
4	Bacteria	Terrabacteria group	Cyanobacteria/Melainabacteria group	Cyanobacteria	Oscillatoriophycideae	Oscillatoriales	unclassified Oscillatoriales
4	Bacteria	Terrabacteria group	Cyanobacteria/Melainabacteria group	Cyanobacteria	Oscillatoriophycideae	Oscillatoriales	Microcoleaceae
4	Bacteria	Terrabacteria group	Cyanobacteria/Melainabacteria group	Cyanobacteria	Oscillatoriophycideae	Oscillatoriales	Desertifilaceae
4	Bacteria	Terrabacteria group	Cyanobacteria/Melainabacteria group	Cyanobacteria	Oscillatoriophycideae	Oscillatoriales	Coleofasciculaceae
4	Bacteria	Terrabacteria group	Cyanobacteria/Melainabacteria group	Cyanobacteria	Oscillatoriophycideae	Chroococcales	Microcystaceae
4	Bacteria	Terrabacteria group	Cyanobacteria/Melainabacteria group	Cyanobacteria	Nostocales	Rivulariaceae	Calothrix
4	Bacteria	Terrabacteria group	Cyanobacteria/Melainabacteria group	Cyanobacteria	Nostocales	Hapalosiphonaceae	Mastigocoleus
4	Bacteria	Terrabacteria group	Cyanobacteria/Melainabacteria group	Cyanobacteria	Nostocales	Chlorogloeopsidaceae	Chlorogloeopsis
4	Bacteria	Terrabacteria group	Chloroflexi	unclassified Chloroflexi	unclassified Chloroflexi (miscellaneous)	Chloroflexi bacterium RBG_16_57_11	
4	Bacteria	Terrabacteria group	Chloroflexi	Thermomicrobia	unclassified Thermomicrobia	Thermorudis	Thermorudis peleae
4	Bacteria	Terrabacteria group	Chloroflexi	Dehalococcoidia	unclassified Dehalococcoidia	Dehalococcoidia bacterium CG2_30_46_9	
4	Bacteria	Terrabacteria group	Chloroflexi	Dehalococcoidia	Dehalococcoidales	Dehalococcoidaceae	Dehalococcoides
4	Bacteria	Terrabacteria group	Chloroflexi	Chloroflexia	Chloroflexales	Roseiflexineae	Roseiflexaceae
4	Bacteria	Terrabacteria group	Chloroflexi	Anaerolineae	unclassified Anaerolineae	Anaerolineae bacterium CG2_30_64_16	
4	Bacteria	Terrabacteria group	Chloroflexi	Anaerolineae	Anaerolineales	Anaerolineaceae	unclassified Anaerolineaceae
4	Bacteria	Terrabacteria group	Chloroflexi	Anaerolineae	Anaerolineales	Anaerolineaceae	Anaerolinea
4	Bacteria	Terrabacteria group	Chloroflexi	Anaerolineae	Anaerolineales	Anaerolineaceae	Anaerolinea
4	Bacteria	Terrabacteria group	Actinobacteria	unclassified Actinobacteria	Actinobacteria bacterium RBG_16_64_13		
4	Bacteria	Terrabacteria group	Actinobacteria	unclassified Actinobacteria	Actinobacteria bacterium 13_2_20CM_68_14		
4	Bacteria	Terrabacteria group	Actinobacteria	Thermoleophilia	Solirubrobacterales	Patulibacteraceae	Patulibacter
4	Bacteria	Terrabacteria group	Actinobacteria	Rubrobacteria	Rubrobacterales	Rubrobacteraceae	Rubrobacter
4	Bacteria	Terrabacteria group	Actinobacteria	Coriobacteriia	Coriobacteriales	Coriobacteriaceae	Collinsella
4	Bacteria	Terrabacteria group	Actinobacteria	Actinobacteria	unclassified Actinobacteria (class)	unclassified Actinobacteria (class) (miscellaneous)	actinobacterium acAMD-5
4	Bacteria	Terrabacteria group	Actinobacteria	Actinobacteria	Streptosporangiales	Thermomonosporaceae	Actinomadura
4	Bacteria	Terrabacteria group	Actinobacteria	Actinobacteria	Streptosporangiales	Streptosporangiaceae	Streptosporangium
4	Bacteria	Terrabacteria group	Actinobacteria	Actinobacteria	Streptosporangiales	Streptosporangiaceae	Microbispora
4	Bacteria	Terrabacteria group	Actinobacteria	Actinobacteria	Streptosporangiales	Streptosporangiaceae	Microbispora
4	Bacteria	Terrabacteria group	Actinobacteria	Actinobacteria	Streptosporangiales	Nocardiopsaceae	Nocardiopsis
4	Bacteria	Terrabacteria group	Actinobacteria	Actinobacteria	Streptosporangiales	Nocardiopsaceae	Nocardiopsis
4	Bacteria	Terrabacteria group	Actinobacteria	Actinobacteria	Streptomycetales	Streptomycetaceae	Streptomyces
4	Bacteria	Terrabacteria group	Actinobacteria	Actinobacteria	Streptomycetales	Streptomycetaceae	Streptomyces
4	Bacteria	Terrabacteria group	Actinobacteria	Actinobacteria	Streptomycetales	Streptomycetaceae	Streptomyces
4	Bacteria	Terrabacteria group	Actinobacteria	Actinobacteria	Streptomycetales	Streptomycetaceae	Streptomyces
4	Bacteria	Terrabacteria group	Actinobacteria	Actinobacteria	Streptomycetales	Streptomycetaceae	
4	Bacteria	Terrabacteria group	Actinobacteria	Actinobacteria	Pseudonocardiales	Pseudonocardiaceae	Yuhushiella
4	Bacteria	Terrabacteria group	Actinobacteria	Actinobacteria	Pseudonocardiales	Pseudonocardiaceae	Saccharothrix
4	Bacteria	Terrabacteria group	Actinobacteria	Actinobacteria	Pseudonocardiales	Pseudonocardiaceae	Saccharothrix
4	Bacteria	Terrabacteria group	Actinobacteria	Actinobacteria	Pseudonocardiales	Pseudonocardiaceae	Saccharopolyspora
4	Bacteria	Terrabacteria group	Actinobacteria	Actinobacteria	Pseudonocardiales	Pseudonocardiaceae	Pseudonocardia
4	Bacteria	Terrabacteria group	Actinobacteria	Actinobacteria	Pseudonocardiales	Pseudonocardiaceae	Pseudonocardia
4	Bacteria	Terrabacteria group	Actinobacteria	Actinobacteria	Pseudonocardiales	Pseudonocardiaceae	Pseudonocardia
4	Bacteria	Terrabacteria group	Actinobacteria	Actinobacteria	Pseudonocardiales	Pseudonocardiaceae	Amycolatopsis
4	Bacteria	Terrabacteria group	Actinobacteria	Actinobacteria	Pseudonocardiales	Pseudonocardiaceae	Amycolatopsis
4	Bacteria	Terrabacteria group	Actinobacteria	Actinobacteria	Pseudonocardiales	Pseudonocardiaceae	Allokutzneria
4	Bacteria	Terrabacteria group	Actinobacteria	Actinobacteria	Pseudonocardiales	Pseudonocardiaceae	Actinokineospora
4	Bacteria	Terrabacteria group	Actinobacteria	Actinobacteria	Propionibacteriales	Propionibacteriaceae	Raineyella
4	Bacteria	Terrabacteria group	Actinobacteria	Actinobacteria	Propionibacteriales	Nocardioidaceae	Nocardioides
4	Bacteria	Terrabacteria group	Actinobacteria	Actinobacteria	Propionibacteriales	Nocardioidaceae	Nocardioides
4	Bacteria	Terrabacteria group	Actinobacteria	Actinobacteria	Propionibacteriales	Nocardioidaceae	Nocardioides
4	Bacteria	Terrabacteria group	Actinobacteria	Actinobacteria	Propionibacteriales	Nocardioidaceae	Nocardioides
4	Bacteria	Terrabacteria group	Actinobacteria	Actinobacteria	Propionibacteriales	Nocardioidaceae	Mumia
4	Bacteria	Terrabacteria group	Actinobacteria	Actinobacteria	Propionibacteriales	Nocardioidaceae	Marmoricola
4	Bacteria	Terrabacteria group	Actinobacteria	Actinobacteria	Nakamurellales	Nakamurellaceae	Nakamurella
4	Bacteria	Terrabacteria group	Actinobacteria	Actinobacteria	Micromonosporales	Micromonosporaceae	Salinispora
4	Bacteria	Terrabacteria group	Actinobacteria	Actinobacteria	Micromonosporales	Micromonosporaceae	Actinoplanes
4	Bacteria	Terrabacteria group	Actinobacteria	Actinobacteria	Micromonosporales	Micromonosporaceae	Actinoplanes
4	Bacteria	Terrabacteria group	Actinobacteria	Actinobacteria	Micromonosporales	Micromonosporaceae	
4	Bacteria	Terrabacteria group	Actinobacteria	Actinobacteria	Micrococcales	unclassified Micrococcales	unclassified Micrococcales (miscellaneous)
4	Bacteria	Terrabacteria group	Actinobacteria	Actinobacteria	Micrococcales	Micrococcaceae	Arthrobacter
4	Bacteria	Terrabacteria group	Actinobacteria	Actinobacteria	Micrococcales	Microbacteriaceae	Microbacterium
4	Bacteria	Terrabacteria group	Actinobacteria	Actinobacteria	Micrococcales	Microbacteriaceae	Leucobacter
4	Bacteria	Terrabacteria group	Actinobacteria	Actinobacteria	Micrococcales	Microbacteriaceae	Curtobacterium
4	Bacteria	Terrabacteria group	Actinobacteria	Actinobacteria	Micrococcales	Microbacteriaceae	Agromyces
4	Bacteria	Terrabacteria group	Actinobacteria	Actinobacteria	Micrococcales	Microbacteriaceae	Agrococcus
4	Bacteria	Terrabacteria group	Actinobacteria	Actinobacteria	Micrococcales	Intrasporangiaceae	Tetrasphaera
4	Bacteria	Terrabacteria group	Actinobacteria	Actinobacteria	Micrococcales	Intrasporangiaceae	Tetrasphaera
4	Bacteria	Terrabacteria group	Actinobacteria	Actinobacteria	Micrococcales	Intrasporangiaceae	Terrabacter
4	Bacteria	Terrabacteria group	Actinobacteria	Actinobacteria	Micrococcales	Intrasporangiaceae	Phycicoccus
4	Bacteria	Terrabacteria group	Actinobacteria	Actinobacteria	Micrococcales	Intrasporangiaceae	Phycicoccus
4	Bacteria	Terrabacteria group	Actinobacteria	Actinobacteria	Micrococcales	Intrasporangiaceae	Intrasporangium
4	Bacteria	Terrabacteria group	Actinobacteria	Actinobacteria	Micrococcales	Dermatophilaceae	Piscicoccus
4	Bacteria	Terrabacteria group	Actinobacteria	Actinobacteria	Micrococcales	Cellulomonadaceae	Cellulomonas
4	Bacteria	Terrabacteria group	Actinobacteria	Actinobacteria	Micrococcales	Cellulomonadaceae	Cellulomonas
4	Bacteria	Terrabacteria group	Actinobacteria	Actinobacteria	Kineosporiales	Kineosporiaceae	Quadrisphaera
4	Bacteria	Terrabacteria group	Actinobacteria	Actinobacteria	Jiangellales	Jiangellaceae	Jiangella
4	Bacteria	Terrabacteria group	Actinobacteria	Actinobacteria	Geodermatophilales	Geodermatophilaceae	unclassified Geodermatophilaceae
4	Bacteria	Terrabacteria group	Actinobacteria	Actinobacteria	Geodermatophilales	Geodermatophilaceae	Modestobacter
4	Bacteria	Terrabacteria group	Actinobacteria	Actinobacteria	Geodermatophilales	Geodermatophilaceae	Geodermatophilus
4	Bacteria	Terrabacteria group	Actinobacteria	Actinobacteria	Geodermatophilales	Geodermatophilaceae	Blastococcus
4	Bacteria	Terrabacteria group	Actinobacteria	Actinobacteria	Geodermatophilales	Geodermatophilaceae	Blastococcus
4	Bacteria	Terrabacteria group	Actinobacteria	Actinobacteria	Corynebacteriales	Nocardiaceae	Rhodococcus
4	Bacteria	Terrabacteria group	Actinobacteria	Actinobacteria	Corynebacteriales	Nocardiaceae	Nocardia
4	Bacteria	Terrabacteria group	Actinobacteria	Actinobacteria	Corynebacteriales	Nocardiaceae	Nocardia
4	Bacteria	Terrabacteria group	Actinobacteria	Actinobacteria	Corynebacteriales	Nocardiaceae	Nocardia
4	Bacteria	Terrabacteria group	Actinobacteria	Actinobacteria	Corynebacteriales	Mycobacteriaceae	Mycobacterium
4	Bacteria	Terrabacteria group	Actinobacteria	Actinobacteria	Corynebacteriales	Mycobacteriaceae	Mycobacterium
4	Bacteria	Terrabacteria group	Actinobacteria	Actinobacteria	Corynebacteriales	Mycobacteriaceae	Mycobacterium
4	Bacteria	Terrabacteria group	Actinobacteria	Actinobacteria	Corynebacteriales	Mycobacteriaceae	Mycobacterium
4	Bacteria	Terrabacteria group	Actinobacteria	Actinobacteria	Corynebacteriales	Mycobacteriaceae	Mycobacterium
4	Bacteria	Terrabacteria group	Actinobacteria	Actinobacteria	Corynebacteriales	Mycobacteriaceae	Mycobacterium
4	Bacteria	Terrabacteria group	Actinobacteria	Actinobacteria	Corynebacteriales	Dietziaceae	Dietzia
4	Bacteria	Terrabacteria group	Actinobacteria	Actinobacteria	Corynebacteriales	Corynebacteriaceae	
4	Bacteria	Terrabacteria group	Actinobacteria	Actinobacteria	Catenulisporales	Catenulisporaceae	Catenulispora
4	Bacteria	Terrabacteria group	Actinobacteria	Actinobacteria	Bifidobacteriales	Bifidobacteriaceae	Bifidobacterium
4	Bacteria	Terrabacteria group	Actinobacteria	Actinobacteria	Actinomycetales	Actinomycetaceae	Actinomyces
4	Bacteria	Terrabacteria group	Actinobacteria	Acidimicrobiia	Acidimicrobiales	Acidimicrobiaceae	Ferrimicrobium
4	Bacteria	Synergistetes	Synergistia	Synergistales	unclassified Synergistales	Synergistales bacterium 54_24	
4	Bacteria	Synergistetes	Synergistia	Synergistales	Synergistaceae	Thermanaerovibrio	
4	Bacteria	Synergistetes	Synergistia	Synergistales	Synergistaceae	Pyramidobacter	Pyramidobacter piscolens
4	Bacteria	Synergistetes	Synergistia	Synergistales			
4	Bacteria	Spirochaetes	Spirochaetia	Spirochaetales	Spirochaetaceae	Treponema	Treponema saccharophilum
4	Bacteria	Spirochaetes	Spirochaetia	Spirochaetales	Spirochaetaceae	Treponema	Treponema pedis
4	Bacteria	Spirochaetes	Spirochaetia	Spirochaetales	Spirochaetaceae	Treponema	Treponema maltophilum
4	Bacteria	Spirochaetes	Spirochaetia	Spirochaetales	Spirochaetaceae	Spirochaeta	
4	Bacteria	Spirochaetes	Spirochaetia	Spirochaetales	Spirochaetaceae	Sediminispirochaeta	Sediminispirochaeta smaragdinae
4	Bacteria	Proteobacteria	unclassified Proteobacteria	unclassified Proteobacteria (miscellaneous)	Proteobacteria bacterium Nonnen-W8red		
4	Bacteria	Proteobacteria	delta/epsilon subdivisions	Epsilonproteobacteria	Nautiliales	Nautiliaceae	Lebetimonas
4	Bacteria	Proteobacteria	delta/epsilon subdivisions	Epsilonproteobacteria	Campylobacterales	Helicobacteraceae	unclassified Helicobacteraceae
4	Bacteria	Proteobacteria	delta/epsilon subdivisions	Epsilonproteobacteria	Campylobacterales	Helicobacteraceae	Sulfurimonas
4	Bacteria	Proteobacteria	delta/epsilon subdivisions	Epsilonproteobacteria	Campylobacterales	Helicobacteraceae	Sulfuricurvum
4	Bacteria	Proteobacteria	delta/epsilon subdivisions	Epsilonproteobacteria	Campylobacterales	Helicobacteraceae	Helicobacter
4	Bacteria	Proteobacteria	delta/epsilon subdivisions	Epsilonproteobacteria	Campylobacterales	Helicobacteraceae	Helicobacter
4	Bacteria	Proteobacteria	delta/epsilon subdivisions	Epsilonproteobacteria	Campylobacterales	Campylobacteraceae	Sulfurospirillum
4	Bacteria	Proteobacteria	delta/epsilon subdivisions	Deltaproteobacteria	unclassified Deltaproteobacteria	unclassified Deltaproteobacteria (miscellaneous)	Deltaproteobacteria bacterium RIFCSPLOWO2_12_FULL_60_16
4	Bacteria	Proteobacteria	delta/epsilon subdivisions	Deltaproteobacteria	unclassified Deltaproteobacteria	unclassified Deltaproteobacteria (miscellaneous)	Deltaproteobacteria bacterium RIFCSPLOWO2_02_FULL_50_16
4	Bacteria	Proteobacteria	delta/epsilon subdivisions	Deltaproteobacteria	unclassified Deltaproteobacteria	unclassified Deltaproteobacteria (miscellaneous)	Deltaproteobacteria bacterium RBG_16_47_11
4	Bacteria	Proteobacteria	delta/epsilon subdivisions	Deltaproteobacteria	unclassified Deltaproteobacteria	unclassified Deltaproteobacteria (miscellaneous)	Deltaproteobacteria bacterium GWC2_65_14
4	Bacteria	Proteobacteria	delta/epsilon subdivisions	Deltaproteobacteria	unclassified Deltaproteobacteria	unclassified Deltaproteobacteria (miscellaneous)	Deltaproteobacteria bacterium GWB2_55_19
4	Bacteria	Proteobacteria	delta/epsilon subdivisions	Deltaproteobacteria	Syntrophobacterales	unclassified Syntrophobacterales	Syntrophobacterales bacterium RIFOXYC2_FULL_60_23
4	Bacteria	Proteobacteria	delta/epsilon subdivisions	Deltaproteobacteria	Syntrophobacterales	unclassified Syntrophobacterales	Syntrophobacterales bacterium RBG_19FT_COMBO_59_10
4	Bacteria	Proteobacteria	delta/epsilon subdivisions	Deltaproteobacteria	Syntrophobacterales	Syntrophaceae	unclassified Syntrophaceae
4	Bacteria	Proteobacteria	delta/epsilon subdivisions	Deltaproteobacteria	Syntrophobacterales	Syntrophaceae	Smithella
4	Bacteria	Proteobacteria	delta/epsilon subdivisions	Deltaproteobacteria	Myxococcales	unclassified Myxococcales	unclassified Myxococcales (miscellaneous)
4	Bacteria	Proteobacteria	delta/epsilon subdivisions	Deltaproteobacteria	Myxococcales	Cystobacterineae	Anaeromyxobacteraceae
4	Bacteria	Proteobacteria	delta/epsilon subdivisions	Deltaproteobacteria	Desulfuromonadales	unclassified Desulfuromonadales	Desulfuromonadales bacterium GWC2_61_20
4	Bacteria	Proteobacteria	delta/epsilon subdivisions	Deltaproteobacteria	Desulfuromonadales	unclassified Desulfuromonadales	Desulfuromonadales bacterium C00003096
4	Bacteria	Proteobacteria	delta/epsilon subdivisions	Deltaproteobacteria	Desulfuromonadales	Geobacteraceae	unclassified Geobacteraceae
4	Bacteria	Proteobacteria	delta/epsilon subdivisions	Deltaproteobacteria	Desulfuromonadales	Desulfuromonadaceae	Pelobacter
4	Bacteria	Proteobacteria	delta/epsilon subdivisions	Deltaproteobacteria	Desulfuromonadales	Desulfuromonadaceae	Malonomonas
4	Bacteria	Proteobacteria	delta/epsilon subdivisions	Deltaproteobacteria	Desulfovibrionales	Desulfovibrionaceae	Desulfovibrio
4	Bacteria	Proteobacteria	delta/epsilon subdivisions	Deltaproteobacteria	Desulfovibrionales	Desulfovibrionaceae	Desulfovibrio
4	Bacteria	Proteobacteria	delta/epsilon subdivisions	Deltaproteobacteria	Desulfovibrionales	Desulfovibrionaceae	Desulfovibrio
4	Bacteria	Proteobacteria	delta/epsilon subdivisions	Deltaproteobacteria	Desulfovibrionales	Desulfovibrionaceae	Desulfovibrio
4	Bacteria	Proteobacteria	delta/epsilon subdivisions	Deltaproteobacteria	Desulfobacterales	unclassified Desulfobacterales	Desulfobacterales bacterium GWB2_56_26
4	Bacteria	Proteobacteria	delta/epsilon subdivisions	Deltaproteobacteria	Desulfobacterales	Desulfobacteraceae	unclassified Desulfobacteraceae
4	Bacteria	Proteobacteria	delta/epsilon subdivisions	Deltaproteobacteria	Desulfobacterales	Desulfobacteraceae	Desulfobacula
4	Bacteria	Proteobacteria	delta/epsilon subdivisions	Deltaproteobacteria	Desulfobacterales	Desulfobacteraceae	Candidatus Magnetoglobus
4	Bacteria	Proteobacteria	delta/epsilon subdivisions	Deltaproteobacteria	Bdellovibrionales	unclassified Bdellovibrionales	Bdellovibrionales bacterium RIFOXYD1_FULL_53_11
4	Bacteria	Proteobacteria	delta/epsilon subdivisions	Deltaproteobacteria	Bdellovibrionales	Bacteriovoracaceae	Bacteriovorax
4	Bacteria	Proteobacteria	Zetaproteobacteria	unclassified Zetaproteobacteria	Zetaproteobacteria bacterium CG1_02_55_237		
4	Bacteria	Proteobacteria	Zetaproteobacteria	unclassified Zetaproteobacteria	Zetaproteobacteria bacterium CG1_02_49_23		
4	Bacteria	Proteobacteria	Zetaproteobacteria	Mariprofundales	Mariprofundaceae	Mariprofundus	Mariprofundus micogutta
4	Bacteria	Proteobacteria	Gammaproteobacteria	unclassified Gammaproteobacteria	unclassified Gammaproteobacteria (miscellaneous)	gamma proteobacterium IMCC2047	
4	Bacteria	Proteobacteria	Gammaproteobacteria	unclassified Gammaproteobacteria	unclassified Gammaproteobacteria (miscellaneous)	Gammaproteobacteria bacterium SCGC AG-212-F23	
4	Bacteria	Proteobacteria	Gammaproteobacteria	unclassified Gammaproteobacteria	unclassified Gammaproteobacteria (miscellaneous)	Gammaproteobacteria bacterium RIFCSPLOWO2_12_FULL_52_10	
4	Bacteria	Proteobacteria	Gammaproteobacteria	unclassified Gammaproteobacteria	unclassified Gammaproteobacteria (miscellaneous)	Gammaproteobacteria bacterium RIFCSPHIGHO2_12_FULL_43_28	
4	Bacteria	Proteobacteria	Gammaproteobacteria	unclassified Gammaproteobacteria	unclassified Gammaproteobacteria (miscellaneous)	Gammaproteobacteria bacterium RIFCSPHIGHO2_12_FULL_42_10	
4	Bacteria	Proteobacteria	Gammaproteobacteria	unclassified Gammaproteobacteria	unclassified Gammaproteobacteria (miscellaneous)	Gammaproteobacteria bacterium RIFCSPHIGHO2_12_FULL_37_14	
4	Bacteria	Proteobacteria	Gammaproteobacteria	unclassified Gammaproteobacteria	unclassified Gammaproteobacteria (miscellaneous)	Gammaproteobacteria bacterium GWE2_37_16	
4	Bacteria	Proteobacteria	Gammaproteobacteria	unclassified Gammaproteobacteria	sulfur-oxidizing symbionts	Solemya velesiana gill symbiont	
4	Bacteria	Proteobacteria	Gammaproteobacteria	unclassified Gammaproteobacteria	OMG group	OM182 clade	OM182 bacterium BACL3 MAG-120619-bin3
4	Bacteria	Proteobacteria	Gammaproteobacteria	Xanthomonadales	Xanthomonadaceae	Xanthomonas	Xanthomonas massiliensis
4	Bacteria	Proteobacteria	Gammaproteobacteria	Xanthomonadales	Xanthomonadaceae	Xanthomonas	Xanthomonas citri group
4	Bacteria	Proteobacteria	Gammaproteobacteria	Xanthomonadales	Xanthomonadaceae	Xanthomonas	Xanthomonas bromi
4	Bacteria	Proteobacteria	Gammaproteobacteria	Xanthomonadales	Xanthomonadaceae	Stenotrophomonas	Stenotrophomonas sp. BIIR7
4	Bacteria	Proteobacteria	Gammaproteobacteria	Xanthomonadales	Xanthomonadaceae	Stenotrophomonas	Stenotrophomonas humi
4	Bacteria	Proteobacteria	Gammaproteobacteria	Xanthomonadales	Xanthomonadaceae	Stenotrophomonas	Stenotrophomonas chelatiphaga
4	Bacteria	Proteobacteria	Gammaproteobacteria	Xanthomonadales	Rhodanobacteraceae	Frateuria	Frateuria sp. Soil773
4	Bacteria	Proteobacteria	Gammaproteobacteria	Xanthomonadales	Rhodanobacteraceae	Frateuria	Frateuria aurantia
4	Bacteria	Proteobacteria	Gammaproteobacteria	Xanthomonadales	Rhodanobacteraceae	Dyella	Dyella japonica
4	Bacteria	Proteobacteria	Gammaproteobacteria	Vibrionales	Vibrionaceae	Vibrio	Vibrio vulnificus
4	Bacteria	Proteobacteria	Gammaproteobacteria	Vibrionales	Vibrionaceae	Vibrio	Vibrio quintilis
4	Bacteria	Proteobacteria	Gammaproteobacteria	Vibrionales	Vibrionaceae	Vibrio	Vibrio harveyi group
4	Bacteria	Proteobacteria	Gammaproteobacteria	Vibrionales	Vibrionaceae	Vibrio	Vibrio cidicii
4	Bacteria	Proteobacteria	Gammaproteobacteria	Vibrionales	Vibrionaceae	Photobacterium	Photobacterium sp. JCM 19050
4	Bacteria	Proteobacteria	Gammaproteobacteria	Vibrionales	Vibrionaceae	Grimontia	Grimontia hollisae
4	Bacteria	Proteobacteria	Gammaproteobacteria	Vibrionales	Vibrionaceae	Grimontia	
4	Bacteria	Proteobacteria	Gammaproteobacteria	Vibrionales	Vibrionaceae	Enterovibrio	Enterovibrio norvegicus
4	Bacteria	Proteobacteria	Gammaproteobacteria	Thiotrichales	Thiotrichaceae	Beggiatoa	
4	Bacteria	Proteobacteria	Gammaproteobacteria	Thiotrichales	Piscirickettsiaceae	Cycloclasticus	Cycloclasticus sp. symbiont of Bathymodiolus heckerae
4	Bacteria	Proteobacteria	Gammaproteobacteria	Salinisphaerales	Salinisphaeraceae	Salinisphaera	Salinisphaera shabanensis
4	Bacteria	Proteobacteria	Gammaproteobacteria	Pseudomonadales	Pseudomonadaceae	Pseudomonas	Pseudomonas vancouverensis
4	Bacteria	Proteobacteria	Gammaproteobacteria	Pseudomonadales	Pseudomonadaceae	Pseudomonas	Pseudomonas sp. TCU-HL1
4	Bacteria	Proteobacteria	Gammaproteobacteria	Pseudomonadales	Pseudomonadaceae	Pseudomonas	Pseudomonas sp. P1.31
4	Bacteria	Proteobacteria	Gammaproteobacteria	Pseudomonadales	Pseudomonadaceae	Pseudomonas	Pseudomonas sp. NFACC07-1
4	Bacteria	Proteobacteria	Gammaproteobacteria	Pseudomonadales	Pseudomonadaceae	Pseudomonas	Pseudomonas sp. MF4836
4	Bacteria	Proteobacteria	Gammaproteobacteria	Pseudomonadales	Pseudomonadaceae	Pseudomonas	Pseudomonas sp. BAY1663
4	Bacteria	Proteobacteria	Gammaproteobacteria	Pseudomonadales	Pseudomonadaceae	Pseudomonas	Pseudomonas sp. ATCC PTA-122608
4	Bacteria	Proteobacteria	Gammaproteobacteria	Pseudomonadales	Pseudomonadaceae	Pseudomonas	Pseudomonas helleri
4	Bacteria	Proteobacteria	Gammaproteobacteria	Pseudomonadales	Pseudomonadaceae	Pseudomonas	Pseudomonas benzenivorans
4	Bacteria	Proteobacteria	Gammaproteobacteria	Pseudomonadales	Pseudomonadaceae	Pseudomonas	Pseudomonas azotifigens
4	Bacteria	Proteobacteria	Gammaproteobacteria	Pseudomonadales	Pseudomonadaceae	Pseudomonas	Pseudomonas alkylphenolica
4	Bacteria	Proteobacteria	Gammaproteobacteria	Pseudomonadales	Pseudomonadaceae	Pseudomonas	Pseudomonas alcaliphila
4	Bacteria	Proteobacteria	Gammaproteobacteria	Pseudomonadales	Pseudomonadaceae	Pseudomonas	Pseudomonas agarici
4	Bacteria	Proteobacteria	Gammaproteobacteria	Pseudomonadales	Pseudomonadaceae	Pseudomonas	Pseudomonas aeruginosa group
4	Bacteria	Proteobacteria	Gammaproteobacteria	Pseudomonadales	Moraxellaceae	Psychrobacter	Psychrobacter sp. PRwf-1
4	Bacteria	Proteobacteria	Gammaproteobacteria	Pseudomonadales	Moraxellaceae	Moraxella	Moraxella macacae
4	Bacteria	Proteobacteria	Gammaproteobacteria	Pseudomonadales	Moraxellaceae	Moraxella	Moraxella bovis
4	Bacteria	Proteobacteria	Gammaproteobacteria	Pseudomonadales	Moraxellaceae	Alkanindiges	Alkanindiges sp. H1
4	Bacteria	Proteobacteria	Gammaproteobacteria	Pseudomonadales	Moraxellaceae	Acinetobacter	Acinetobacter seohaensis
4	Bacteria	Proteobacteria	Gammaproteobacteria	Pseudomonadales	Moraxellaceae		
4	Bacteria	Proteobacteria	Gammaproteobacteria	Pasteurellales	Pasteurellaceae	unclassified Pasteurellaceae	[Haemophilus] felis
4	Bacteria	Proteobacteria	Gammaproteobacteria	Pasteurellales	Pasteurellaceae	Pasteurella	Pasteurella multocida
4	Bacteria	Proteobacteria	Gammaproteobacteria	Pasteurellales	Pasteurellaceae	Gallibacterium	
4	Bacteria	Proteobacteria	Gammaproteobacteria	Orbales	Orbaceae	Frischella	Frischella perrara
4	Bacteria	Proteobacteria	Gammaproteobacteria	Oceanospirillales	Oceanospirillaceae	Oceanospirillum	Oceanospirillum maris
4	Bacteria	Proteobacteria	Gammaproteobacteria	Oceanospirillales	Oceanospirillaceae	Nitrincola	Nitrincola lacisaponensis
4	Bacteria	Proteobacteria	Gammaproteobacteria	Oceanospirillales	Oceanospirillaceae	Neptunomonas	Neptunomonas qingdaonensis
4	Bacteria	Proteobacteria	Gammaproteobacteria	Oceanospirillales	Oceanospirillaceae	Neptunomonas	Neptunomonas phycophila
4	Bacteria	Proteobacteria	Gammaproteobacteria	Oceanospirillales	Oceanospirillaceae	Neptunomonas	Neptunomonas antarctica
4	Bacteria	Proteobacteria	Gammaproteobacteria	Oceanospirillales	Oceanospirillaceae	Bermanella	Bermanella marisrubri
4	Bacteria	Proteobacteria	Gammaproteobacteria	Oceanospirillales	Oceanospirillaceae		
4	Bacteria	Proteobacteria	Gammaproteobacteria	Oceanospirillales	Halomonadaceae	unclassified Halomonadaceae	Halomonadaceae bacterium T82-2
4	Bacteria	Proteobacteria	Gammaproteobacteria	Oceanospirillales	Halomonadaceae	Halomonas	Halomonas sp. Marseille-P2426
4	Bacteria	Proteobacteria	Gammaproteobacteria	Oceanospirillales	Halomonadaceae	Halomonas	Halomonas muralis
4	Bacteria	Proteobacteria	Gammaproteobacteria	Oceanospirillales	Halomonadaceae	Halomonas	Halomonas daqingensis
4	Bacteria	Proteobacteria	Gammaproteobacteria	Oceanospirillales	Hahellaceae	Endozoicomonas	Endozoicomonas elysicola
4	Bacteria	Proteobacteria	Gammaproteobacteria	Oceanospirillales	Alcanivoracaceae	Alcanivorax	Alcanivorax sp. DG881
4	Bacteria	Proteobacteria	Gammaproteobacteria	Oceanospirillales	Alcanivoracaceae	Alcanivorax	Alcanivorax dieselolei
4	Bacteria	Proteobacteria	Gammaproteobacteria	Nevskiales	Sinobacteraceae	Fontimonas	Fontimonas thermophila
4	Bacteria	Proteobacteria	Gammaproteobacteria	Methylococcales	Methylococcaceae	Methylomonas	Methylomonas sp. MK1
4	Bacteria	Proteobacteria	Gammaproteobacteria	Methylococcales	Methylococcaceae	Methylobacter	Methylobacter whittenburyi
4	Bacteria	Proteobacteria	Gammaproteobacteria	Legionellales	Legionellaceae	Legionella	Legionella sp. 40-6
4	Bacteria	Proteobacteria	Gammaproteobacteria	Legionellales	Legionellaceae	Legionella	Legionella jamestowniensis
4	Bacteria	Proteobacteria	Gammaproteobacteria	Legionellales	Legionellaceae	Legionella	Legionella fallonii
4	Bacteria	Proteobacteria	Gammaproteobacteria	Legionellales	Legionellaceae	Legionella	Legionella erythra
4	Bacteria	Proteobacteria	Gammaproteobacteria	Legionellales	Coxiellaceae	Candidatus Berkiella	Candidatus Berkiella cookevillensis
4	Bacteria	Proteobacteria	Gammaproteobacteria	Enterobacterales	unclassified Enterobacterales	Plesiomonas	
4	Bacteria	Proteobacteria	Gammaproteobacteria	Enterobacterales	Yersiniaceae	Yersinia	Yersinia pseudotuberculosis complex
4	Bacteria	Proteobacteria	Gammaproteobacteria	Enterobacterales	Yersiniaceae	Serratia	Serratia plymuthica
4	Bacteria	Proteobacteria	Gammaproteobacteria	Enterobacterales	Pectobacteriaceae	Pectobacterium	
4	Bacteria	Proteobacteria	Gammaproteobacteria	Enterobacterales	Pectobacteriaceae	Lonsdalea	Lonsdalea quercina
4	Bacteria	Proteobacteria	Gammaproteobacteria	Enterobacterales	Morganellaceae	Xenorhabdus	Xenorhabdus innexi
4	Bacteria	Proteobacteria	Gammaproteobacteria	Enterobacterales	Morganellaceae	Providencia	Providencia rettgeri
4	Bacteria	Proteobacteria	Gammaproteobacteria	Enterobacterales	Morganellaceae	Photorhabdus	Photorhabdus luminescens
4	Bacteria	Proteobacteria	Gammaproteobacteria	Enterobacterales	Morganellaceae	Photorhabdus	
4	Bacteria	Proteobacteria	Gammaproteobacteria	Enterobacterales	Morganellaceae	Morganella	Morganella psychrotolerans
4	Bacteria	Proteobacteria	Gammaproteobacteria	Enterobacterales	Morganellaceae	Morganella	Morganella morganii
4	Bacteria	Proteobacteria	Gammaproteobacteria	Enterobacterales	Morganellaceae	Moellerella	Moellerella wisconsensis
4	Bacteria	Proteobacteria	Gammaproteobacteria	Enterobacterales	Hafniaceae	Edwardsiella	
4	Bacteria	Proteobacteria	Gammaproteobacteria	Enterobacterales	Erwiniaceae	Pantoea	Pantoea sp. A4
4	Bacteria	Proteobacteria	Gammaproteobacteria	Enterobacterales	Erwiniaceae	Erwinia	Erwinia teleogrylli
4	Bacteria	Proteobacteria	Gammaproteobacteria	Enterobacterales	Enterobacteriaceae	unclassified Enterobacteriaceae	ant, tsetse, mealybug, aphid, etc. endosymbionts
4	Bacteria	Proteobacteria	Gammaproteobacteria	Enterobacterales	Enterobacteriaceae	Kluyvera	Kluyvera intermedia
4	Bacteria	Proteobacteria	Gammaproteobacteria	Enterobacterales	Enterobacteriaceae	Enterobacter	Enterobacter cloacae complex
4	Bacteria	Proteobacteria	Gammaproteobacteria	Enterobacterales	Enterobacteriaceae	Buttiauxella	Buttiauxella ferragutiae
4	Bacteria	Proteobacteria	Gammaproteobacteria	Chromatiales	Halothiobacillaceae	Halothiobacillus	
4	Bacteria	Proteobacteria	Gammaproteobacteria	Chromatiales	Ectothiorhodospiraceae	Thiorhodospira	Thiorhodospira sibirica
4	Bacteria	Proteobacteria	Gammaproteobacteria	Chromatiales	Chromatiaceae	Thiorhodococcus	
4	Bacteria	Proteobacteria	Gammaproteobacteria	Cellvibrionales	Porticoccaceae	unclassified Porticoccaceae	SAR92 clade
4	Bacteria	Proteobacteria	Gammaproteobacteria	Cellvibrionales	Porticoccaceae	unclassified Porticoccaceae	SAR92 clade
4	Bacteria	Proteobacteria	Gammaproteobacteria	Cellvibrionales	Cellvibrionaceae	Teredinibacter	Teredinibacter turnerae
4	Bacteria	Proteobacteria	Gammaproteobacteria	Cellvibrionales	Cellvibrionaceae	Gilvimarinus	Gilvimarinus polysaccharolyticus
4	Bacteria	Proteobacteria	Gammaproteobacteria	Cellvibrionales			
4	Bacteria	Proteobacteria	Gammaproteobacteria	Cardiobacteriales	Cardiobacteriaceae	Suttonella	Suttonella ornithocola
4	Bacteria	Proteobacteria	Gammaproteobacteria	Alteromonadales	Shewanellaceae	Shewanella	Shewanella sp. Sh95
4	Bacteria	Proteobacteria	Gammaproteobacteria	Alteromonadales	Shewanellaceae	Shewanella	Shewanella denitrificans
4	Bacteria	Proteobacteria	Gammaproteobacteria	Alteromonadales	Shewanellaceae	Shewanella	Shewanella amazonensis
4	Bacteria	Proteobacteria	Gammaproteobacteria	Alteromonadales	Pseudoalteromonadaceae	Pseudoalteromonas	Pseudoalteromonas phenolica
4	Bacteria	Proteobacteria	Gammaproteobacteria	Alteromonadales	Moritellaceae	Moritella	Moritella viscosa
4	Bacteria	Proteobacteria	Gammaproteobacteria	Alteromonadales	Ferrimonadaceae	Ferrimonas	Ferrimonas marina
4	Bacteria	Proteobacteria	Gammaproteobacteria	Alteromonadales	Colwelliaceae	Thalassomonas	Thalassomonas viridans
4	Bacteria	Proteobacteria	Gammaproteobacteria	Alteromonadales	Alteromonadaceae	unclassified Alteromonadaceae	Alteromonadaceae bacterium Bs31
4	Bacteria	Proteobacteria	Gammaproteobacteria	Alteromonadales	Alteromonadaceae	Paraglaciecola	Paraglaciecola sp. S66
4	Bacteria	Proteobacteria	Gammaproteobacteria	Alteromonadales	Alteromonadaceae	Paraglaciecola	Paraglaciecola arctica
4	Bacteria	Proteobacteria	Gammaproteobacteria	Alteromonadales	Alteromonadaceae	Paraglaciecola	Paraglaciecola agarilytica
4	Bacteria	Proteobacteria	Gammaproteobacteria	Alteromonadales	Alteromonadaceae	Marinobacter	Marinobacter zhejiangensis
4	Bacteria	Proteobacteria	Gammaproteobacteria	Alteromonadales	Alteromonadaceae	Marinobacter	Marinobacter sp. HL-58
4	Bacteria	Proteobacteria	Gammaproteobacteria	Alteromonadales	Alteromonadaceae	Marinobacter	Marinobacter excellens
4	Bacteria	Proteobacteria	Gammaproteobacteria	Alteromonadales	Alteromonadaceae	Glaciecola	Glaciecola sp. HTCC2999
4	Bacteria	Proteobacteria	Gammaproteobacteria	Alteromonadales	Alteromonadaceae	Glaciecola	Glaciecola nitratireducens
4	Bacteria	Proteobacteria	Gammaproteobacteria	Alteromonadales	Alteromonadaceae	Alteromonas	Alteromonas lipolytica
4	Bacteria	Proteobacteria	Gammaproteobacteria	Alteromonadales	Alteromonadaceae	Alteromonas	Alteromonas confluentis
4	Bacteria	Proteobacteria	Gammaproteobacteria	Alteromonadales	Alteromonadaceae	Alishewanella	
4	Bacteria	Proteobacteria	Gammaproteobacteria	Aeromonadales	Succinivibrionaceae	Anaerobiospirillum	Anaerobiospirillum succiniciproducens
4	Bacteria	Proteobacteria	Gammaproteobacteria	Aeromonadales	Aeromonadaceae	Aeromonas	Aeromonas caviae
4	Bacteria	Proteobacteria	Gammaproteobacteria	Aeromonadales	Aeromonadaceae	Aeromonas	Aeromonas bivalvium
4	Bacteria	Proteobacteria	Gammaproteobacteria	Aeromonadales	Aeromonadaceae	Aeromonas	Aeromonas australiensis
4	Bacteria	Proteobacteria	Gammaproteobacteria	Aeromonadales	Aeromonadaceae	Aeromonas	Aeromonas aquatica

4	Bacteria	Proteobacteria	Candidatus Muproteobacteria	Candidatus Muproteobacteria bacterium RIFCSPHIGHO2_01_FULL_61_200			
4	Bacteria	Proteobacteria	Candidatus Muproteobacteria				
4	Bacteria	Proteobacteria	Candidatus Lambdaproteobacteria				
4	Bacteria	Proteobacteria	Betaproteobacteria	unclassified Betaproteobacteria	unclassified Betaproteobacteria [illegible]	[illegible]	[illegible]
4	Bacteria	Proteobacteria	Betaproteobacteria	unclassified Betaproteobacteria	unclassified Betaproteobacteria [illegible]	[illegible]	[illegible]
4	Bacteria	Proteobacteria	Betaproteobacteria	unclassified Betaproteobacteria	unclassified Betaproteobacteria [illegible]	[illegible]	[illegible]
4	Bacteria	Proteobacteria	Betaproteobacteria	Rhodocyclales	Rhodocyclaceae	[illegible]	[illegible]
4	Bacteria	Proteobacteria	Betaproteobacteria	Rhodocyclales	Rhodocyclaceae	[illegible]	[illegible]
4	Bacteria	Proteobacteria	Betaproteobacteria	Rhodocyclales	Rhodocyclaceae	[illegible]	[illegible]
4	Bacteria	Proteobacteria	Betaproteobacteria	Nitrosomonadales	unclassified Nitrosomonadales	[illegible]	[illegible]
4	Bacteria	Proteobacteria	Betaproteobacteria	Nitrosomonadales	Nitrosomonadaceae	[illegible]	[illegible]
4	Bacteria	Proteobacteria	Betaproteobacteria	Nitrosomonadales	Nitrosomonadaceae	[illegible]	[illegible]
4	Bacteria	Proteobacteria	Betaproteobacteria	Nitrosomonadales	Nitrosomonadaceae	[illegible]	[illegible]
4	Bacteria	Proteobacteria	Betaproteobacteria	Nitrosomonadales	Nitrosomonadaceae	[illegible]	[illegible]
4	Bacteria	Proteobacteria	Betaproteobacteria	Nitrosomonadales	Nitrosomonadaceae	[illegible]	[illegible]
4	Bacteria	Proteobacteria	Betaproteobacteria	Nitrosomonadales	Nitrosomonadaceae	[illegible]	[illegible]
4	Bacteria	Proteobacteria	Betaproteobacteria	Neisseriales	Neisseriaceae	[illegible]	[illegible]
4	Bacteria	Proteobacteria	Betaproteobacteria	Neisseriales	Neisseriaceae	[illegible]	[illegible]
4	Bacteria	Proteobacteria	Betaproteobacteria	Neisseriales	Neisseriaceae	[illegible]	[illegible]
4	Bacteria	Proteobacteria	Betaproteobacteria	Neisseriales	Neisseriaceae	[illegible]	[illegible]
4	Bacteria	Proteobacteria	Betaproteobacteria	Neisseriales	Neisseriaceae	[illegible]	[illegible]
4	Bacteria	Proteobacteria	Betaproteobacteria	Neisseriales	Chromobacteriaceae	[illegible]	[illegible]
4	Bacteria	Proteobacteria	Betaproteobacteria	Methylophilales	Methylophilaceae	[illegible]	[illegible]
4	Bacteria	Proteobacteria	Betaproteobacteria	Methylophilales	Methylophilaceae	[illegible]	[illegible]
4	Bacteria	Proteobacteria	Betaproteobacteria	Methylophilales	Methylophilaceae	[illegible]	[illegible]
4	Bacteria	Proteobacteria	Betaproteobacteria	Methylophilales	Methylophilaceae	[illegible]	[illegible]
4	Bacteria	Proteobacteria	Betaproteobacteria	Hydrogenophilales	unclassified Hydrogenophilales	[illegible]	[illegible]
4	Bacteria	Proteobacteria	Betaproteobacteria	Hydrogenophilales	Hydrogenophilaceae	[illegible]	[illegible]
4	Bacteria	Proteobacteria	Betaproteobacteria	Gallionellales	Gallionellaceae	[illegible]	[illegible]
4	Bacteria	Proteobacteria	Betaproteobacteria	Ferrovales	Ferrovaceae	[illegible]	[illegible]
4	Bacteria	Proteobacteria	Betaproteobacteria	Burkholderiales	unclassified Burkholderiales	[illegible]	[illegible]
4	Bacteria	Proteobacteria	Betaproteobacteria	Burkholderiales	unclassified Burkholderiales	[illegible]	[illegible]
4	Bacteria	Proteobacteria	Betaproteobacteria	Burkholderiales	unclassified Burkholderiales	[illegible]	[illegible]
4	Bacteria	Proteobacteria	Betaproteobacteria	Burkholderiales	unclassified Burkholderiales	[illegible]	[illegible]
4	Bacteria	Proteobacteria	Betaproteobacteria	Burkholderiales	unclassified Burkholderiales	[illegible]	[illegible]
4	Bacteria	Proteobacteria	Betaproteobacteria	Burkholderiales	unclassified Burkholderiales	[illegible]	[illegible]
4	Bacteria	Proteobacteria	Betaproteobacteria	Burkholderiales	Oxalobacteraceae	[illegible]	[illegible]
4	Bacteria	Proteobacteria	Betaproteobacteria	Burkholderiales	Oxalobacteraceae	[illegible]	[illegible]
4	Bacteria	Proteobacteria	Betaproteobacteria	Burkholderiales	Oxalobacteraceae	[illegible]	[illegible]
4	Bacteria	Proteobacteria	Betaproteobacteria	Burkholderiales	Oxalobacteraceae	[illegible]	[illegible]
4	Bacteria	Proteobacteria	Betaproteobacteria	Burkholderiales	Oxalobacteraceae	[illegible]	[illegible]
4	Bacteria	Proteobacteria	Betaproteobacteria	Burkholderiales	Oxalobacteraceae	[illegible]	[illegible]
4	Bacteria	Proteobacteria	Betaproteobacteria	Burkholderiales	Comamonadaceae	[illegible]	[illegible]
[illegible]	[illegible]	[illegible]	[illegible]	[illegible]	[illegible]	[illegible]	[illegible]

3	Bacteria	Terrabacteria group	Firmicutes	Clostridia	Clostridiales	Peptococcaceae	Desulfitobacterium
3	Bacteria	Terrabacteria group	Firmicutes	Clostridia	Clostridiales	Oscillospiraceae	Oscillibacter
3	Bacteria	Terrabacteria group	Firmicutes	Clostridia	Clostridiales	Oscillospiraceae	Oscillibacter
3	Bacteria	Terrabacteria group	Firmicutes	Clostridia	Clostridiales	Oscillospiraceae	Oscillibacter
3	Bacteria	Terrabacteria group	Firmicutes	Clostridia	Clostridiales	Lachnospiraceae	unclassified Lachnospiraceae
3	Bacteria	Terrabacteria group	Firmicutes	Clostridia	Clostridiales	Lachnospiraceae	unclassified Lachnospiraceae
3	Bacteria	Terrabacteria group	Firmicutes	Clostridia	Clostridiales	Lachnospiraceae	unclassified Lachnospiraceae
3	Bacteria	Terrabacteria group	Firmicutes	Clostridia	Clostridiales	Lachnospiraceae	unclassified Lachnospiraceae
3	Bacteria	Terrabacteria group	Firmicutes	Clostridia	Clostridiales	Lachnospiraceae	unclassified Lachnospiraceae
3	Bacteria	Terrabacteria group	Firmicutes	Clostridia	Clostridiales	Lachnospiraceae	unclassified Lachnospiraceae
3	Bacteria	Terrabacteria group	Firmicutes	Clostridia	Clostridiales	Lachnospiraceae	unclassified Lachnospiraceae
3	Bacteria	Terrabacteria group	Firmicutes	Clostridia	Clostridiales	Lachnospiraceae	Tyzzerella
3	Bacteria	Terrabacteria group	Firmicutes	Clostridia	Clostridiales	Lachnospiraceae	Shuttleworthia
3	Bacteria	Terrabacteria group	Firmicutes	Clostridia	Clostridiales	Lachnospiraceae	Roseburia
3	Bacteria	Terrabacteria group	Firmicutes	Clostridia	Clostridiales	Lachnospiraceae	Roseburia
3	Bacteria	Terrabacteria group	Firmicutes	Clostridia	Clostridiales	Lachnospiraceae	Pseudobutyrivibrio
3	Bacteria	Terrabacteria group	Firmicutes	Clostridia	Clostridiales	Lachnospiraceae	Pseudobutyrivibrio
3	Bacteria	Terrabacteria group	Firmicutes	Clostridia	Clostridiales	Lachnospiraceae	Lachnoclostridium
3	Bacteria	Terrabacteria group	Firmicutes	Clostridia	Clostridiales	Lachnospiraceae	Lachnoclostridium
3	Bacteria	Terrabacteria group	Firmicutes	Clostridia	Clostridiales	Lachnospiraceae	Lachnobacterium
3	Bacteria	Terrabacteria group	Firmicutes	Clostridia	Clostridiales	Lachnospiraceae	Johnsonella
3	Bacteria	Terrabacteria group	Firmicutes	Clostridia	Clostridiales	Lachnospiraceae	Dorea
3	Bacteria	Terrabacteria group	Firmicutes	Clostridia	Clostridiales	Lachnospiraceae	Coprococcus
3	Bacteria	Terrabacteria group	Firmicutes	Clostridia	Clostridiales	Lachnospiraceae	Blautia
3	Bacteria	Terrabacteria group	Firmicutes	Clostridia	Clostridiales	Lachnospiraceae	Anaerostipes
3	Bacteria	Terrabacteria group	Firmicutes	Clostridia	Clostridiales	Eubacteriaceae	Pseudoramibacter
3	Bacteria	Terrabacteria group	Firmicutes	Clostridia	Clostridiales	Eubacteriaceae	Eubacterium
3	Bacteria	Terrabacteria group	Firmicutes	Clostridia	Clostridiales	Eubacteriaceae	Eubacterium
3	Bacteria	Terrabacteria group	Firmicutes	Clostridia	Clostridiales	Eubacteriaceae	Acetobacterium
3	Bacteria	Terrabacteria group	Firmicutes	Clostridia	Clostridiales	Eubacteriaceae	Acetobacterium
3	Bacteria	Terrabacteria group	Firmicutes	Clostridia	Clostridiales	Eubacteriaceae	Acetobacterium
3	Bacteria	Terrabacteria group	Firmicutes	Clostridia	Clostridiales	Clostridiales incertae sedis	Clostridiales Family XVII. Incertae Sedis
3	Bacteria	Terrabacteria group	Firmicutes	Clostridia	Clostridiales	Clostridiales incertae sedis	Clostridiales Family XIII. Incertae Sedis
3	Bacteria	Terrabacteria group	Firmicutes	Clostridia	Clostridiales	Clostridiaceae	Thermobrachium
3	Bacteria	Terrabacteria group	Firmicutes	Clostridia	Clostridiales	Clostridiaceae	Paramaledivibacter
3	Bacteria	Terrabacteria group	Firmicutes	Clostridia	Clostridiales	Clostridiaceae	Geosporobacter
3	Bacteria	Terrabacteria group	Firmicutes	Clostridia	Clostridiales	Clostridiaceae	Clostridium
3	Bacteria	Terrabacteria group	Firmicutes	Clostridia	Clostridiales	Clostridiaceae	Clostridium
3	Bacteria	Terrabacteria group	Firmicutes	Clostridia	Clostridiales	Clostridiaceae	Clostridium
3	Bacteria	Terrabacteria group	Firmicutes	Clostridia	Clostridiales	Clostridiaceae	Clostridium
3	Bacteria	Terrabacteria group	Firmicutes	Clostridia	Clostridiales	Clostridiaceae	Clostridium
3	Bacteria	Terrabacteria group	Firmicutes	Clostridia	Clostridiales	Clostridiaceae	Clostridium
3	Bacteria	Terrabacteria group	Firmicutes	Clostridia	Clostridiales	Clostridiaceae	Clostridium
3	Bacteria	Terrabacteria group	Firmicutes	Clostridia	Clostridiales	Clostridiaceae	Clostridium
3	Bacteria	Terrabacteria group	Firmicutes	Clostridia	Clostridiales	Clostridiaceae	Clostridium
3	Bacteria	Terrabacteria group	Firmicutes	Clostridia	Clostridiales	Clostridiaceae	Clostridium
3	Bacteria	Terrabacteria group	Firmicutes	Clostridia	Clostridiales	Clostridiaceae	Clostridium
3	Bacteria	Terrabacteria group	Firmicutes	Clostridia	Clostridiales	Clostridiaceae	Clostridium
3	Bacteria	Terrabacteria group	Firmicutes	Clostridia	Clostridiales	Clostridiaceae	Clostridium
3	Bacteria	Terrabacteria group	Firmicutes	Clostridia	Clostridiales	Clostridiaceae	Clostridium
3	Bacteria	Terrabacteria group	Firmicutes	Clostridia	Clostridiales	Clostridiaceae	Clostridium
3	Bacteria	Terrabacteria group	Firmicutes	Clostridia	Clostridiales	Clostridiaceae	Clostridium
3	Bacteria	Terrabacteria group	Firmicutes	Clostridia	Clostridiales	Clostridiaceae	Clostridium
3	Bacteria	Terrabacteria group	Firmicutes	Clostridia	Clostridiales	Clostridiaceae	Clostridium
3	Bacteria	Terrabacteria group	Firmicutes	Clostridia	Clostridiales	Clostridiaceae	Candidatus Arthromitus
3	Bacteria	Terrabacteria group	Firmicutes	Clostridia	Clostridiales	Clostridiaceae	Caloranaerobacter
3	Bacteria	Terrabacteria group	Firmicutes	Clostridia	Clostridiales	Clostridiaceae	Caloramator
3	Bacteria	Terrabacteria group	Firmicutes	Clostridia	Clostridiales	Clostridiaceae	Butyricicoccus
3	Bacteria	Terrabacteria group	Firmicutes	Clostridia	Clostridiales	Clostridiaceae	Anaerosalibacter
3	Bacteria	Terrabacteria group	Firmicutes	Clostridia	Clostridiales	Clostridiaceae	Anaerosalibacter
3	Bacteria	Terrabacteria group	Firmicutes	Clostridia	Clostridiales	Clostridiaceae	Alkaliphilus
3	Bacteria	Terrabacteria group	Firmicutes	Bacilli	Lactobacillales	Streptococcaceae	Streptococcus
3	Bacteria	Terrabacteria group	Firmicutes	Bacilli	Lactobacillales	Streptococcaceae	Streptococcus
3	Bacteria	Terrabacteria group	Firmicutes	Bacilli	Lactobacillales	Lactobacillaceae	Pediococcus
3	Bacteria	Terrabacteria group	Firmicutes	Bacilli	Lactobacillales	Enterococcaceae	Vagococcus
3	Bacteria	Terrabacteria group	Firmicutes	Bacilli	Lactobacillales	Enterococcaceae	Tetragenococcus
3	Bacteria	Terrabacteria group	Firmicutes	Bacilli	Lactobacillales	Enterococcaceae	Enterococcus
3	Bacteria	Terrabacteria group	Firmicutes	Bacilli	Lactobacillales	Enterococcaceae	Enterococcus
3	Bacteria	Terrabacteria group	Firmicutes	Bacilli	Lactobacillales	Enterococcaceae	Enterococcus
3	Bacteria	Terrabacteria group	Firmicutes	Bacilli	Lactobacillales	Enterococcaceae	Enterococcus
3	Bacteria	Terrabacteria group	Firmicutes	Bacilli	Lactobacillales	Carnobacteriaceae	Trichococcus
3	Bacteria	Terrabacteria group	Firmicutes	Bacilli	Lactobacillales	Carnobacteriaceae	Isobaculum
3	Bacteria	Terrabacteria group	Firmicutes	Bacilli	Lactobacillales	Carnobacteriaceae	Carnobacterium
3	Bacteria	Terrabacteria group	Firmicutes	Bacilli	Lactobacillales	Aerococcaceae	Facklamia
3	Bacteria	Terrabacteria group	Firmicutes	Bacilli	Bacillales	unclassified Bacillales	Geomicrobium
3	Bacteria	Terrabacteria group	Firmicutes	Bacilli	Bacillales	unclassified Bacillales	Desulfuribacillus
3	Bacteria	Terrabacteria group	Firmicutes	Bacilli	Bacillales	Thermoactinomycetaceae	Planifilum
3	Bacteria	Terrabacteria group	Firmicutes	Bacilli	Bacillales	Thermoactinomycetaceae	Marininema
3	Bacteria	Terrabacteria group	Firmicutes	Bacilli	Bacillales	Thermoactinomycetaceae	
3	Bacteria	Terrabacteria group	Firmicutes	Bacilli	Bacillales	Staphylococcaceae	Staphylococcus
3	Bacteria	Terrabacteria group	Firmicutes	Bacilli	Bacillales	Sporolactobacillaceae	Sporolactobacillus
3	Bacteria	Terrabacteria group	Firmicutes	Bacilli	Bacillales	Planococcaceae	Sporosarcina
3	Bacteria	Terrabacteria group	Firmicutes	Bacilli	Bacillales	Planococcaceae	Caryophanon
3	Bacteria	Terrabacteria group	Firmicutes	Bacilli	Bacillales	Paenibacillaceae	Paenibacillus
3	Bacteria	Terrabacteria group	Firmicutes	Bacilli	Bacillales	Paenibacillaceae	Paenibacillus
3	Bacteria	Terrabacteria group	Firmicutes	Bacilli	Bacillales	Paenibacillaceae	Paenibacillus
3	Bacteria	Terrabacteria group	Firmicutes	Bacilli	Bacillales	Paenibacillaceae	Paenibacillus
3	Bacteria	Terrabacteria group	Firmicutes	Bacilli	Bacillales	Paenibacillaceae	Paenibacillus
3	Bacteria	Terrabacteria group	Firmicutes	Bacilli	Bacillales	Paenibacillaceae	Paenibacillus
3	Bacteria	Terrabacteria group	Firmicutes	Bacilli	Bacillales	Paenibacillaceae	Paenibacillus
3	Bacteria	Terrabacteria group	Firmicutes	Bacilli	Bacillales	Paenibacillaceae	Paenibacillus
3	Bacteria	Terrabacteria group	Firmicutes	Bacilli	Bacillales	Paenibacillaceae	Paenibacillus
3	Bacteria	Terrabacteria group	Firmicutes	Bacilli	Bacillales	Paenibacillaceae	Paenibacillus
3	Bacteria	Terrabacteria group	Firmicutes	Bacilli	Bacillales	Paenibacillaceae	Paenibacillus
3	Bacteria	Terrabacteria group	Firmicutes	Bacilli	Bacillales	Paenibacillaceae	Cohnella
3	Bacteria	Terrabacteria group	Firmicutes	Bacilli	Bacillales	Bacillaceae	Virgibacillus
3	Bacteria	Terrabacteria group	Firmicutes	Bacilli	Bacillales	Bacillaceae	Pontibacillus
3	Bacteria	Terrabacteria group	Firmicutes	Bacilli	Bacillales	Bacillaceae	Pontibacillus
3	Bacteria	Terrabacteria group	Firmicutes	Bacilli	Bacillales	Bacillaceae	Oceanobacillus
3	Bacteria	Terrabacteria group	Firmicutes	Bacilli	Bacillales	Bacillaceae	Lysinibacillus
3	Bacteria	Terrabacteria group	Firmicutes	Bacilli	Bacillales	Bacillaceae	Lysinibacillus
3	Bacteria	Terrabacteria group	Firmicutes	Bacilli	Bacillales	Bacillaceae	Lysinibacillus
3	Bacteria	Terrabacteria group	Firmicutes	Bacilli	Bacillales	Bacillaceae	Lysinibacillus
3	Bacteria	Terrabacteria group	Firmicutes	Bacilli	Bacillales	Bacillaceae	Fictibacillus
3	Bacteria	Terrabacteria group	Firmicutes	Bacilli	Bacillales	Bacillaceae	Bacillus
3	Bacteria	Terrabacteria group	Firmicutes	Bacilli	Bacillales	Bacillaceae	Bacillus
3	Bacteria	Terrabacteria group	Firmicutes	Bacilli	Bacillales	Bacillaceae	Bacillus
3	Bacteria	Terrabacteria group	Firmicutes	Bacilli	Bacillales	Bacillaceae	Bacillus
3	Bacteria	Terrabacteria group	Firmicutes	Bacilli	Bacillales	Bacillaceae	Bacillus
3	Bacteria	Terrabacteria group	Firmicutes	Bacilli	Bacillales	Bacillaceae	Bacillus
3	Bacteria	Terrabacteria group	Firmicutes	Bacilli	Bacillales	Bacillaceae	Bacillus
3	Bacteria	Terrabacteria group	Firmicutes	Bacilli	Bacillales	Bacillaceae	Bacillus
3	Bacteria	Terrabacteria group	Firmicutes	Bacilli	Bacillales	Bacillaceae	Bacillus
3	Bacteria	Terrabacteria group	Firmicutes	Bacilli	Bacillales	Bacillaceae	Bacillus
3	Bacteria	Terrabacteria group	Firmicutes	Bacilli	Bacillales	Bacillaceae	Bacillus
3	Bacteria	Terrabacteria group	Firmicutes	Bacilli	Bacillales	Bacillaceae	Bacillus
3	Bacteria	Terrabacteria group	Firmicutes	Bacilli	Bacillales	Bacillaceae	Bacillus
3	Bacteria	Terrabacteria group	Firmicutes	Bacilli	Bacillales	Bacillaceae	Bacillus
3	Bacteria	Terrabacteria group	Firmicutes	Bacilli	Bacillales	Bacillaceae	Bacillus
3	Bacteria	Terrabacteria group	Firmicutes	Bacilli	Bacillales	Bacillaceae	Amphibacillus
3	Bacteria	Terrabacteria group	Firmicutes	Bacilli	Bacillales	Bacillaceae	Aeribacillus
3	Bacteria	Terrabacteria group	Firmicutes	Bacilli	Bacillales	Alicyclobacillaceae	Alicyclobacillus
3	Bacteria	Terrabacteria group	Firmicutes	Bacilli	Bacillales	Alicyclobacillaceae	Alicyclobacillus
3	Bacteria	Terrabacteria group	Firmicutes	Bacilli	Bacillales	Alicyclobacillaceae	Alicyclobacillus
3	Bacteria	Terrabacteria group	Deinococcus-Thermus	Deinococci	Thermales	Thermaceae	Thermus
3	Bacteria	Terrabacteria group	Deinococcus-Thermus	Deinococci	Thermales	Thermaceae	Oceanithermus
3	Bacteria	Terrabacteria group	Deinococcus-Thermus	Deinococci	Thermales	Thermaceae	Meiothermus
3	Bacteria	Terrabacteria group	Deinococcus-Thermus	Deinococci	Deinococcales	Deinococcaceae	Deinococcus
3	Bacteria	Terrabacteria group	Deinococcus-Thermus	Deinococci	Deinococcales	Deinococcaceae	Deinococcus
3	Bacteria	Terrabacteria group	Deinococcus-Thermus	Deinococci	Deinococcales	Deinococcaceae	Deinococcus
3	Bacteria	Terrabacteria group	Deinococcus-Thermus	Deinococci	Deinococcales	Deinococcaceae	Deinococcus
3	Bacteria	Terrabacteria group	Cyanobacteria/Melainabacteria group	Cyanobacteria	Synechococcales	Synechococcaceae	Synechococcus
3	Bacteria	Terrabacteria group	Cyanobacteria/Melainabacteria group	Cyanobacteria	Synechococcales	Synechococcaceae	Synechococcus
3	Bacteria	Terrabacteria group	Cyanobacteria/Melainabacteria group	Cyanobacteria	Synechococcales	Synechococcaceae	Synechococcus
3	Bacteria	Terrabacteria group	Cyanobacteria/Melainabacteria group	Cyanobacteria	Synechococcales	Synechococcaceae	Synechococcus
3	Bacteria	Terrabacteria group	Cyanobacteria/Melainabacteria group	Cyanobacteria	Synechococcales	Pseudanabaenaceae	Pseudanabaena
3	Bacteria	Terrabacteria group	Cyanobacteria/Melainabacteria group	Cyanobacteria	Synechococcales	Pseudanabaenaceae	Prochlorothrix
3	Bacteria	Terrabacteria group	Cyanobacteria/Melainabacteria group	Cyanobacteria	Synechococcales	Leptolyngbyaceae	Leptolyngbya
3	Bacteria	Terrabacteria group	Cyanobacteria/Melainabacteria group	Cyanobacteria	Synechococcales	Leptolyngbyaceae	Leptolyngbya
3	Bacteria	Terrabacteria group	Cyanobacteria/Melainabacteria group	Cyanobacteria	Synechococcales	Leptolyngbyaceae	Halomicronema
3	Bacteria	Terrabacteria group	Cyanobacteria/Melainabacteria group	Cyanobacteria	Pleurocapsales	Hyellaceae	Pleurocapsa
3	Bacteria	Terrabacteria group	Cyanobacteria/Melainabacteria group	Cyanobacteria	Pleurocapsales	Dermocarpellaceae	Stanieria
3	Bacteria	Terrabacteria group	Cyanobacteria/Melainabacteria group	Cyanobacteria	Pleurocapsales		
3	Bacteria	Terrabacteria group	Cyanobacteria/Melainabacteria group	Cyanobacteria	Oscillatoriophycideae	Oscillatoriales	Oscillatoriaceae
3	Bacteria	Terrabacteria group	Cyanobacteria/Melainabacteria group	Cyanobacteria	Oscillatoriophycideae	Oscillatoriales	Oscillatoriaceae
3	Bacteria	Terrabacteria group	Cyanobacteria/Melainabacteria group	Cyanobacteria	Oscillatoriophycideae	Oscillatoriales	Oscillatoriaceae
3	Bacteria	Terrabacteria group	Cyanobacteria/Melainabacteria group	Cyanobacteria	Oscillatoriophycideae	Oscillatoriales	Cyanothecaceae
3	Bacteria	Terrabacteria group	Cyanobacteria/Melainabacteria group	Cyanobacteria	Oscillatoriophycideae	Oscillatoriales	Cyanothecaceae
3	Bacteria	Terrabacteria group	Cyanobacteria/Melainabacteria group	Cyanobacteria	Oscillatoriophycideae	Oscillatoriales	Cyanothecaceae
3	Bacteria	Terrabacteria group	Cyanobacteria/Melainabacteria group	Cyanobacteria	Oscillatoriophycideae	Oscillatoriales	Coleofasciculaceae
3	Bacteria	Terrabacteria group	Cyanobacteria/Melainabacteria group	Cyanobacteria	Oscillatoriophycideae	Chroococcales	Aphanothecaceae
3	Bacteria	Terrabacteria group	Cyanobacteria/Melainabacteria group	Cyanobacteria	Nostocales	Tolypothrichaceae	Tolypothrix
3	Bacteria	Terrabacteria group	Cyanobacteria/Melainabacteria group	Cyanobacteria	Nostocales	Tolypothrichaceae	Tolypothrix
3	Bacteria	Terrabacteria group	Cyanobacteria/Melainabacteria group	Cyanobacteria	Nostocales	Scytonemataceae	Scytonema
3	Bacteria	Terrabacteria group	Cyanobacteria/Melainabacteria group	Cyanobacteria	Nostocales	Rivulariaceae	Calothrix
3	Bacteria	Terrabacteria group	Cyanobacteria/Melainabacteria group	Cyanobacteria	Nostocales	Rivulariaceae	Calothrix
3	Bacteria	Terrabacteria group	Cyanobacteria/Melainabacteria group	Cyanobacteria	Nostocales	Rivulariaceae	Calothrix
3	Bacteria	Terrabacteria group	Cyanobacteria/Melainabacteria group	Cyanobacteria	Nostocales	Rivulariaceae	Calothrix
3	Bacteria	Terrabacteria group	Cyanobacteria/Melainabacteria group	Cyanobacteria	Nostocales	Nostocaceae	Nostoc
3	Bacteria	Terrabacteria group	Cyanobacteria/Melainabacteria group	Cyanobacteria	Nostocales	Nostocaceae	Anabaena
3	Bacteria	Terrabacteria group	Cyanobacteria/Melainabacteria group	Cyanobacteria	Nostocales	Hapalosiphonaceae	Hapalosiphon
3	Bacteria	Terrabacteria group	Cyanobacteria/Melainabacteria group	Cyanobacteria	Nostocales	Hapalosiphonaceae	Fischerella
3	Bacteria	Terrabacteria group	Cyanobacteria/Melainabacteria group	Cyanobacteria	Nostocales	Aphanizomenonaceae	Nodularia
3	Bacteria	Terrabacteria group	Cyanobacteria/Melainabacteria group	Cyanobacteria	Gloeobacteria	Gloeobacterales	Gloeobacteraceae
3	Bacteria	Terrabacteria group	Cyanobacteria/Melainabacteria group	Cyanobacteria	Gloeobacteria	Gloeobacterales	Gloeobacteraceae
3	Bacteria	Terrabacteria group	Cyanobacteria/Melainabacteria group	Cyanobacteria	Chroococcidiopsidales	Chroococcidiopsidaceae	Chroococcidiopsis
3	Bacteria	Terrabacteria group	Chloroflexi	unclassified Chloroflexi	unclassified Chloroflexi (miscellaneous)	Chloroflexi bacterium RBG_19FT_COMBO_50_10	
3	Bacteria	Terrabacteria group	Chloroflexi	unclassified Chloroflexi	unclassified Chloroflexi (miscellaneous)	Chloroflexi bacterium RBG_16_64_32	
3	Bacteria	Terrabacteria group	Chloroflexi	unclassified Chloroflexi	unclassified Chloroflexi (miscellaneous)	Chloroflexi bacterium RBG_16_63_12	
3	Bacteria	Terrabacteria group	Chloroflexi	unclassified Chloroflexi	unclassified Chloroflexi (miscellaneous)	Chloroflexi bacterium RBG_16_60_22	
3	Bacteria	Terrabacteria group	Chloroflexi	unclassified Chloroflexi	unclassified Chloroflexi (miscellaneous)	Chloroflexi bacterium RBG_16_57_9	
3	Bacteria	Terrabacteria group	Chloroflexi	unclassified Chloroflexi	unclassified Chloroflexi (miscellaneous)	Chloroflexi bacterium RBG_16_50_9	
3	Bacteria	Terrabacteria group	Chloroflexi	unclassified Chloroflexi	unclassified Chloroflexi (miscellaneous)	Chloroflexi bacterium RBG_16_48_8	
3	Bacteria	Terrabacteria group	Chloroflexi	unclassified Chloroflexi	unclassified Chloroflexi (miscellaneous)	Chloroflexi bacterium RBG_13_51_36	
3	Bacteria	Terrabacteria group	Chloroflexi	unclassified Chloroflexi	unclassified Chloroflexi (miscellaneous)	Chloroflexi bacterium RBG_13_51_18	
3	Bacteria	Terrabacteria group	Chloroflexi	unclassified Chloroflexi	unclassified Chloroflexi (miscellaneous)	Chloroflexi bacterium RBG_13_50_10	
3	Bacteria	Terrabacteria group	Chloroflexi	unclassified Chloroflexi	unclassified Chloroflexi (miscellaneous)	Chloroflexi bacterium 44-23	
3	Bacteria	Terrabacteria group	Chloroflexi	Ktedonobacteria	Ktedonobacterales	Ktedonobacteraceae	Ktedonobacter
3	Bacteria	Terrabacteria group	Chloroflexi	Dehalococcoidia	unclassified Dehalococcoidia	Dehalococcoidia bacterium SM23_28_1	
3	Bacteria	Terrabacteria group	Chloroflexi	Anaerolineae	unclassified Anaerolineae	Anaerolineae bacterium UTCFX1	
3	Bacteria	Terrabacteria group	Chloroflexi	Anaerolineae	unclassified Anaerolineae	Anaerolineae bacterium CG1_02_58_13	
3	Bacteria	Terrabacteria group	Chloroflexi	Anaerolineae	Anaerolineales	Anaerolineaceae	Anaerolinea
3	Bacteria	Terrabacteria group	Armatimonadetes	unclassified Armatimonadetes	Armatimonadetes bacterium RBG_19FT_COMBO_69_19		
3	Bacteria	Terrabacteria group	Armatimonadetes	unclassified Armatimonadetes	Armatimonadetes bacterium RBG_16_58_9		
3	Bacteria	Terrabacteria group	Armatimonadetes	unclassified Armatimonadetes	Armatimonadetes bacterium 13_1_40CM_3_65_7		
3	Bacteria	Terrabacteria group	Armatimonadetes	unclassified Armatimonadetes			
3	Bacteria	Terrabacteria group	Actinobacteria	unclassified Actinobacteria	Actinobacteria bacterium RBG_19FT_COMBO_70_19		
3	Bacteria	Terrabacteria group	Actinobacteria	unclassified Actinobacteria	Actinobacteria bacterium RBG_16_67_10		
3	Bacteria	Terrabacteria group	Actinobacteria	unclassified Actinobacteria	Actinobacteria bacterium RBG_13_63_9		
3	Bacteria	Terrabacteria group	Actinobacteria	unclassified Actinobacteria	Actinobacteria bacterium GWC2_53_9		
3	Bacteria	Terrabacteria group	Actinobacteria	unclassified Actinobacteria	Actinobacteria bacterium 13_2_20CM_2_71_6		
3	Bacteria	Terrabacteria group	Actinobacteria	Thermoleophilia	Solirubrobacterales	Patulibacteraceae	Patulibacter
3	Bacteria	Terrabacteria group	Actinobacteria	Thermoleophilia	Solirubrobacterales		
3	Bacteria	Terrabacteria group	Actinobacteria	Coriobacteriia	Eggerthellales	Eggerthellaceae	Enterorhabdus
3	Bacteria	Terrabacteria group	Actinobacteria	Coriobacteriia	Eggerthellales	Eggerthellaceae	
3	Bacteria	Terrabacteria group	Actinobacteria	Coriobacteriia	Coriobacteriales	Coriobacteriaceae	unclassified Coriobacteriaceae
3	Bacteria	Terrabacteria group	Actinobacteria	Coriobacteriia	Coriobacteriales	Coriobacteriaceae	unclassified Coriobacteriaceae
3	Bacteria	Terrabacteria group	Actinobacteria	Coriobacteriia	Coriobacteriales	Coriobacteriaceae	Collinsella
3	Bacteria	Terrabacteria group	Actinobacteria	Coriobacteriia	Coriobacteriales	Coriobacteriaceae	Collinsella
3	Bacteria	Terrabacteria group	Actinobacteria	Coriobacteriia	Coriobacteriales	Coriobacteriaceae	
3	Bacteria	Terrabacteria group	Actinobacteria	Coriobacteriia	Coriobacteriales	Atopobiaceae	Olsenella
3	Bacteria	Terrabacteria group	Actinobacteria	Coriobacteriia	Coriobacteriales	Atopobiaceae	Atopobium
3	Bacteria	Terrabacteria group	Actinobacteria	Coriobacteriia	Coriobacteriales	Atopobiaceae	Atopobium
3	Bacteria	Terrabacteria group	Actinobacteria	Actinobacteria	unclassified Actinobacteria (class)	unclassified Actinobacteria (class) (miscellaneous)	actinobacterium LLX17
3	Bacteria	Terrabacteria group	Actinobacteria	Actinobacteria	unclassified Actinobacteria (class)	unclassified Actinobacteria (class) (miscellaneous)	Actinobacteria bacterium 69-20
3	Bacteria	Terrabacteria group	Actinobacteria	Actinobacteria	unclassified Actinobacteria (class)	marine Actinobacteria clade	environmental samples
3	Bacteria	Terrabacteria group	Actinobacteria	Actinobacteria	Streptosporangiales	Thermomonosporaceae	Actinomadura
3	Bacteria	Terrabacteria group	Actinobacteria	Actinobacteria	Streptosporangiales	Thermomonosporaceae	Actinomadura
3	Bacteria	Terrabacteria group	Actinobacteria	Actinobacteria	Streptosporangiales	Thermomonosporaceae	Actinomadura
3	Bacteria	Terrabacteria group	Actinobacteria	Actinobacteria	Streptosporangiales	Thermomonosporaceae	Actinomadura
3	Bacteria	Terrabacteria group	Actinobacteria	Actinobacteria	Streptosporangiales	Thermomonosporaceae	Actinomadura
3	Bacteria	Terrabacteria group	Actinobacteria	Actinobacteria	Streptosporangiales	Thermomonosporaceae	Actinomadura
3	Bacteria	Terrabacteria group	Actinobacteria	Actinobacteria	Streptosporangiales	Streptosporangiaceae	Planomonospora
3	Bacteria	Terrabacteria group	Actinobacteria	Actinobacteria	Streptosporangiales	Streptosporangiaceae	Nonomuraea
3	Bacteria	Terrabacteria group	Actinobacteria	Actinobacteria	Streptosporangiales	Streptosporangiaceae	Nonomuraea
3	Bacteria	Terrabacteria group	Actinobacteria	Actinobacteria	Streptosporangiales	Streptosporangiaceae	Herbidospora
3	Bacteria	Terrabacteria group	Actinobacteria	Actinobacteria	Streptosporangiales	Nocardiopsaceae	Nocardiopsis
3	Bacteria	Terrabacteria group	Actinobacteria	Actinobacteria	Streptosporangiales	Nocardiopsaceae	Marinactinospora
3	Bacteria	Terrabacteria group	Actinobacteria	Actinobacteria	Streptosporangiales	Nocardiopsaceae	
3	Bacteria	Terrabacteria group	Actinobacteria	Actinobacteria	Streptomycetales	Streptomycetaceae	Streptomyces
3	Bacteria	Terrabacteria group	Actinobacteria	Actinobacteria	Streptomycetales	Streptomycetaceae	Streptomyces
3	Bacteria	Terrabacteria group	Actinobacteria	Actinobacteria	Streptomycetales	Streptomycetaceae	Streptomyces
3	Bacteria	Terrabacteria group	Actinobacteria	Actinobacteria	Streptomycetales	Streptomycetaceae	Streptomyces
3	Bacteria	Terrabacteria group	Actinobacteria	Actinobacteria	Streptomycetales	Streptomycetaceae	Streptomyces
3	Bacteria	Terrabacteria group	Actinobacteria	Actinobacteria	Streptomycetales	Streptomycetaceae	Streptomyces
3	Bacteria	Terrabacteria group	Actinobacteria	Actinobacteria	Streptomycetales	Streptomycetaceae	Streptomyces
3	Bacteria	Terrabacteria group	Actinobacteria	Actinobacteria	Streptomycetales	Streptomycetaceae	Streptomyces
3	Bacteria	Terrabacteria group	Actinobacteria	Actinobacteria	Streptomycetales	Streptomycetaceae	Streptomyces
3	Bacteria	Terrabacteria group	Actinobacteria	Actinobacteria	Streptomycetales	Streptomycetaceae	Streptomyces
3	Bacteria	Terrabacteria group	Actinobacteria	Actinobacteria	Streptomycetales	Streptomycetaceae	Streptomyces
3	Bacteria	Terrabacteria group	Actinobacteria	Actinobacteria	Streptomycetales	Streptomycetaceae	Streptomyces
3	Bacteria	Terrabacteria group	Actinobacteria	Actinobacteria	Streptomycetales	Streptomycetaceae	Streptacidiphilus
3	Bacteria	Terrabacteria group	Actinobacteria	Actinobacteria	Streptomycetales	Streptomycetaceae	Kitasatospora
3	Bacteria	Terrabacteria group	Actinobacteria	Actinobacteria	Pseudonocardiales	Pseudonocardiaceae	Streptoalloteichus
3	Bacteria	Terrabacteria group	Actinobacteria	Actinobacteria	Pseudonocardiales	Pseudonocardiaceae	Sciscionella
3	Bacteria	Terrabacteria group	Actinobacteria	Actinobacteria	Pseudonocardiales	Pseudonocardiaceae	Saccharomonospora
3	Bacteria	Terrabacteria group	Actinobacteria	Actinobacteria	Pseudonocardiales	Pseudonocardiaceae	Saccharomonospora
3	Bacteria	Terrabacteria group	Actinobacteria	Actinobacteria	Pseudonocardiales	Pseudonocardiaceae	Pseudonocardia
3	Bacteria	Terrabacteria group	Actinobacteria	Actinobacteria	Pseudonocardiales	Pseudonocardiaceae	Pseudonocardia
3	Bacteria	Terrabacteria group	Actinobacteria	Actinobacteria	Pseudonocardiales	Pseudonocardiaceae	Lechevalieria
3	Bacteria	Terrabacteria group	Actinobacteria	Actinobacteria	Pseudonocardiales	Pseudonocardiaceae	Kutzneria
3	Bacteria	Terrabacteria group	Actinobacteria	Actinobacteria	Pseudonocardiales	Pseudonocardiaceae	Kutzneria
3	Bacteria	Terrabacteria group	Actinobacteria	Actinobacteria	Pseudonocardiales	Pseudonocardiaceae	Amycolatopsis
3	Bacteria	Terrabacteria group	Actinobacteria	Actinobacteria	Pseudonocardiales	Pseudonocardiaceae	Amycolatopsis
3	Bacteria	Terrabacteria group	Actinobacteria	Actinobacteria	Pseudonocardiales	Pseudonocardiaceae	Amycolatopsis
3	Bacteria	Terrabacteria group	Actinobacteria	Actinobacteria	Pseudonocardiales	Pseudonocardiaceae	Amycolatopsis
3	Bacteria	Terrabacteria group	Actinobacteria	Actinobacteria	Pseudonocardiales	Pseudonocardiaceae	Alloactinosynnema
3	Bacteria	Terrabacteria group	Actinobacteria	Actinobacteria	Pseudonocardiales	Pseudonocardiaceae	Actinosynnema
3	Bacteria	Terrabacteria group	Actinobacteria	Actinobacteria	Pseudonocardiales	Pseudonocardiaceae	Actinophytocola
3	Bacteria	Terrabacteria group	Actinobacteria	Actinobacteria	Pseudonocardiales	Pseudonocardiaceae	Actinoalloteichus
3	Bacteria	Terrabacteria group	Actinobacteria	Actinobacteria	Propionibacteriales	Propionibacteriaceae	Tessaracoccus
3	Bacteria	Terrabacteria group	Actinobacteria	Actinobacteria	Propionibacteriales	Propionibacteriaceae	Propionimicrobium
3	Bacteria	Terrabacteria group	Actinobacteria	Actinobacteria	Propionibacteriales	Propionibacteriaceae	Propionibacterium
3	Bacteria	Terrabacteria group	Actinobacteria	Actinobacteria	Propionibacteriales	Propionibacteriaceae	Microlunatus
3	Bacteria	Terrabacteria group	Actinobacteria	Actinobacteria	Propionibacteriales	Propionibacteriaceae	Acidipropionibacterium
3	Bacteria	Terrabacteria group	Actinobacteria	Actinobacteria	Propionibacteriales	Nocardioidaceae	Nocardioides
3	Bacteria	Terrabacteria group	Actinobacteria	Actinobacteria	Propionibacteriales	Nocardioidaceae	Nocardioides
3	Bacteria	Terrabacteria group	Actinobacteria	Actinobacteria	Propionibacteriales	Nocardioidaceae	Aeromicrobium
3	Bacteria	Terrabacteria group	Actinobacteria	Actinobacteria	Micromonosporales	Micromonosporaceae	Salinispora
3	Bacteria	Terrabacteria group	Actinobacteria	Actinobacteria	Micromonosporales	Micromonosporaceae	Micromonospora
3	Bacteria	Terrabacteria group	Actinobacteria	Actinobacteria	Micromonosporales	Micromonosporaceae	Micromonospora
3	Bacteria	Terrabacteria group	Actinobacteria	Actinobacteria	Micromonosporales	Micromonosporaceae	Dactylosporangium
3	Bacteria	Terrabacteria group	Actinobacteria	Actinobacteria	Micromonosporales	Micromonosporaceae	Actinoplanes
3	Bacteria	Terrabacteria group	Actinobacteria	Actinobacteria	Micromonosporales	Micromonosporaceae	Actinoplanes
3	Bacteria	Terrabacteria group	Actinobacteria	Actinobacteria	Micromonosporales	Micromonosporaceae	Actinoplanes
3	Bacteria	Terrabacteria group	Actinobacteria	Actinobacteria	Micromonosporales	Micromonosporaceae	Actinoplanes
3	Bacteria	Terrabacteria group	Actinobacteria	Actinobacteria	Micromonosporales	Micromonosporaceae	Actinoplanes
3	Bacteria	Terrabacteria group	Actinobacteria	Actinobacteria	Micrococcales	Sanguibacteraceae	Sanguibacter
3	Bacteria	Terrabacteria group	Actinobacteria	Actinobacteria	Micrococcales	Sanguibacteraceae	Sanguibacter
3	Bacteria	Terrabacteria group	Actinobacteria	Actinobacteria	Micrococcales	Ruaniaceae	Haloactinobacterium
3	Bacteria	Terrabacteria group	Actinobacteria	Actinobacteria	Micrococcales	Micrococcaceae	Rothia
3	Bacteria	Terrabacteria group	Actinobacteria	Actinobacteria	Micrococcales	Micrococcaceae	Kocuria
3	Bacteria	Terrabacteria group	Actinobacteria	Actinobacteria	Micrococcales	Micrococcaceae	Kocuria
3	Bacteria	Terrabacteria group	Actinobacteria	Actinobacteria	Micrococcales	Micrococcaceae	Kocuria
3	Bacteria	Terrabacteria group	Actinobacteria	Actinobacteria	Micrococcales	Micrococcaceae	Arthrobacter
3	Bacteria	Terrabacteria group	Actinobacteria	Actinobacteria	Micrococcales	Micrococcaceae	Arthrobacter
3	Bacteria	Terrabacteria group	Actinobacteria	Actinobacteria	Micrococcales	Microbacteriaceae	Mycetocola
3	Bacteria	Terrabacteria group	Actinobacteria	Actinobacteria	Micrococcales	Microbacteriaceae	Microbacterium
3	Bacteria	Terrabacteria group	Actinobacteria	Actinobacteria	Micrococcales	Microbacteriaceae	Microbacterium
3	Bacteria	Terrabacteria group	Actinobacteria	Actinobacteria	Micrococcales	Microbacteriaceae	Microbacterium
3	Bacteria	Terrabacteria group	Actinobacteria	Actinobacteria	Micrococcales	Microbacteriaceae	Microbacterium
3	Bacteria	Terrabacteria group	Actinobacteria	Actinobacteria	Micrococcales	Microbacteriaceae	Microbacterium
3	Bacteria	Terrabacteria group	Actinobacteria	Actinobacteria	Micrococcales	Microbacteriaceae	Leifsonia
3	Bacteria	Terrabacteria group	Actinobacteria	Actinobacteria	Micrococcales	Microbacteriaceae	Gulosibacter
3	Bacteria	Terrabacteria group	Actinobacteria	Actinobacteria	Micrococcales	Microbacteriaceae	Agromyces
3	Bacteria	Terrabacteria group	Actinobacteria	Actinobacteria	Micrococcales	Microbacteriaceae	Agrococcus
3	Bacteria	Terrabacteria group	Actinobacteria	Actinobacteria	Micrococcales	Microbacteriaceae	Agrococcus
3	Bacteria	Terrabacteria group	Actinobacteria	Actinobacteria	Micrococcales	Jonesiaceae	Jonesia
3	Bacteria	Terrabacteria group	Actinobacteria	Actinobacteria	Micrococcales	Intrasporangiaceae	Tetrasphaera
3	Bacteria	Terrabacteria group	Actinobacteria	Actinobacteria	Micrococcales	Intrasporangiaceae	Serinicoccus
3	Bacteria	Terrabacteria group	Actinobacteria	Actinobacteria	Micrococcales	Intrasporangiaceae	Janibacter
3	Bacteria	Terrabacteria group	Actinobacteria	Actinobacteria	Micrococcales	Intrasporangiaceae	Intrasporangium
3	Bacteria	Terrabacteria group	Actinobacteria	Actinobacteria	Micrococcales	Intrasporangiaceae	Humibacillus
3	Bacteria	Terrabacteria group	Actinobacteria	Actinobacteria	Micrococcales	Dermatophilaceae	Mobilicoccus
3	Bacteria	Terrabacteria group	Actinobacteria	Actinobacteria	Micrococcales	Dermatophilaceae	Kineosphaera
3	Bacteria	Terrabacteria group	Actinobacteria	Actinobacteria	Micrococcales	Dermacoccaceae	Luteipulveratus
3	Bacteria	Terrabacteria group	Actinobacteria	Actinobacteria	Micrococcales	Dermacoccaceae	Luteipulveratus
3	Bacteria	Terrabacteria group	Actinobacteria	Actinobacteria	Micrococcales	Dermabacteraceae	Devriesea
3	Bacteria	Terrabacteria group	Actinobacteria	Actinobacteria	Micrococcales	Demequinaceae	Demequina
3	Bacteria	Terrabacteria group	Actinobacteria	Actinobacteria	Micrococcales	Demequinaceae	Demequina
3	Bacteria	Terrabacteria group	Actinobacteria	Actinobacteria	Micrococcales	Demequinaceae	
3	Bacteria	Terrabacteria group	Actinobacteria	Actinobacteria	Micrococcales	Cellulomonadaceae	Cellulomonas
3	Bacteria	Terrabacteria group	Actinobacteria	Actinobacteria	Micrococcales	Cellulomonadaceae	Cellulomonas
3	Bacteria	Terrabacteria group	Actinobacteria	Actinobacteria	Micrococcales	Cellulomonadaceae	Cellulomonas
3	Bacteria	Terrabacteria group	Actinobacteria	Actinobacteria	Micrococcales	Cellulomonadaceae	Actinotalea
3	Bacteria	Terrabacteria group	Actinobacteria	Actinobacteria	Micrococcales	Bogoriellaceae	Georgenia
3	Bacteria	Terrabacteria group	Actinobacteria	Actinobacteria	Micrococcales	Beutenbergiaceae	Beutenbergia
3	Bacteria	Terrabacteria group	Actinobacteria	Actinobacteria	Kineosporiales	Kineosporiaceae	Kineococcus
3	Bacteria	Terrabacteria group	Actinobacteria	Actinobacteria	Glycomycetales	Glycomycetaceae	Glycomyces
3	Bacteria	Terrabacteria group	Actinobacteria	Actinobacteria	Geodermatophilales	Geodermatophilaceae	unclassified Geodermatophilaceae
3	Bacteria	Terrabacteria group	Actinobacteria	Actinobacteria	Geodermatophilales	Geodermatophilaceae	Geodermatophilus
3	Bacteria	Terrabacteria group	Actinobacteria	Actinobacteria	Geodermatophilales	Geodermatophilaceae	Geodermatophilus
3	Bacteria	Terrabacteria group	Actinobacteria	Actinobacteria	Geodermatophilales	Geodermatophilaceae	Blastococcus
3	Bacteria	Terrabacteria group	Actinobacteria	Actinobacteria	Geodermatophilales	Geodermatophilaceae	
3	Bacteria	Terrabacteria group	Actinobacteria	Actinobacteria	Frankiales	Frankiaceae	Frankia
3	Bacteria	Terrabacteria group	Actinobacteria	Actinobacteria	Frankiales	Frankiaceae	Frankia
3	Bacteria	Terrabacteria group	Actinobacteria	Actinobacteria	Frankiales	Frankiaceae	Frankia
3	Bacteria	Terrabacteria group	Actinobacteria	Actinobacteria	Frankiales	Cryptosporangiaceae	Cryptosporangium
3	Bacteria	Terrabacteria group	Actinobacteria	Actinobacteria	Corynebacteriales	Williamsiaceae	Williamsia
3	Bacteria	Terrabacteria group	Actinobacteria	Actinobacteria	Corynebacteriales	Nocardiaceae	Rhodococcus
3	Bacteria	Terrabacteria group	Actinobacteria	Actinobacteria	Corynebacteriales	Nocardiaceae	Nocardia
3	Bacteria	Terrabacteria group	Actinobacteria	Actinobacteria	Corynebacteriales	Nocardiaceae	Nocardia
3	Bacteria	Terrabacteria group	Actinobacteria	Actinobacteria	Corynebacteriales	Nocardiaceae	Nocardia
3	Bacteria	Terrabacteria group	Actinobacteria	Actinobacteria	Corynebacteriales	Nocardiaceae	Nocardia
3	Bacteria	Terrabacteria group	Actinobacteria	Actinobacteria	Corynebacteriales	Nocardiaceae	Nocardia
3	Bacteria	Terrabacteria group	Actinobacteria	Actinobacteria	Corynebacteriales	Nocardiaceae	Nocardia
3	Bacteria	Terrabacteria group	Actinobacteria	Actinobacteria	Corynebacteriales	Nocardiaceae	Nocardia
3	Bacteria	Terrabacteria group	Actinobacteria	Actinobacteria	Corynebacteriales	Mycobacteriaceae	Mycobacterium
3	Bacteria	Terrabacteria group	Actinobacteria	Actinobacteria	Corynebacteriales	Mycobacteriaceae	Mycobacterium
3	Bacteria	Terrabacteria group	Actinobacteria	Actinobacteria	Corynebacteriales	Mycobacteriaceae	Mycobacterium
3	Bacteria	Terrabacteria group	Actinobacteria	Actinobacteria	Corynebacteriales	Mycobacteriaceae	Mycobacterium
3	Bacteria	Terrabacteria group	Actinobacteria	Actinobacteria	Corynebacteriales	Mycobacteriaceae	Mycobacterium
3	Bacteria	Terrabacteria group	Actinobacteria	Actinobacteria	Corynebacteriales	Mycobacteriaceae	Mycobacterium
3	Bacteria	Terrabacteria group	Actinobacteria	Actinobacteria	Corynebacteriales	Mycobacteriaceae	Mycobacterium
3	Bacteria	Terrabacteria group	Actinobacteria	Actinobacteria	Corynebacteriales	Mycobacteriaceae	Mycobacterium
3	Bacteria	Terrabacteria group	Actinobacteria	Actinobacteria	Corynebacteriales	Mycobacteriaceae	Mycobacterium
3	Bacteria	Terrabacteria group	Actinobacteria	Actinobacteria	Corynebacteriales	Mycobacteriaceae	Mycobacterium
3	Bacteria	Terrabacteria group	Actinobacteria	Actinobacteria	Corynebacteriales	Mycobacteriaceae	Mycobacterium
3	Bacteria	Terrabacteria group	Actinobacteria	Actinobacteria	Corynebacteriales	Mycobacteriaceae	Mycobacterium
3	Bacteria	Terrabacteria group	Actinobacteria	Actinobacteria	Corynebacteriales	Mycobacteriaceae	Mycobacterium
3	Bacteria	Terrabacteria group	Actinobacteria	Actinobacteria	Corynebacteriales	Mycobacteriaceae	Mycobacterium
3	Bacteria	Terrabacteria group	Actinobacteria	Actinobacteria	Corynebacteriales	Mycobacteriaceae	Mycobacterium
3	Bacteria	Terrabacteria group	Actinobacteria	Actinobacteria	Corynebacteriales	Dietziaceae	Dietzia
3	Bacteria	Terrabacteria group	Actinobacteria	Actinobacteria	Corynebacteriales	Dietziaceae	Dietzia
3	Bacteria	Terrabacteria group	Actinobacteria	Actinobacteria	Corynebacteriales	Corynebacteriaceae	Corynebacterium
3	Bacteria	Terrabacteria group	Actinobacteria	Actinobacteria	Corynebacteriales	Corynebacteriaceae	Corynebacterium
3	Bacteria	Terrabacteria group	Actinobacteria	Actinobacteria	Catenulisporales	Actinospicaceae	Actinospica
3	Bacteria	Terrabacteria group	Actinobacteria	Actinobacteria	Bifidobacteriales	Bifidobacteriaceae	Gardnerella
3	Bacteria	Terrabacteria group	Actinobacteria	Actinobacteria	Actinomycetales	Actinomycetaceae	Actinomyces
3	Bacteria	Terrabacteria group	Actinobacteria	Acidimicrobiia	Acidimicrobiales	Microthrixaceae	Candidatus Microthrix
3	Bacteria	Synergistetes	Synergistia	Synergistales	Synergistaceae	Pyramidobacter	
3	Bacteria	Spirochaetes	unclassified Spirochaetes	Spirochaetes bacterium RBG_16_67_19			
3	Bacteria	Spirochaetes	unclassified Spirochaetes	Spirochaetes bacterium GWF1_49_6			
3	Bacteria	Spirochaetes	Spirochaetia	Spirochaetales	Spirochaetaceae	Treponema	Treponema vincentii
3	Bacteria	Spirochaetes	Spirochaetia	Spirochaetales	Spirochaetaceae	Treponema	Treponema porcinum
3	Bacteria	Spirochaetes	Spirochaetia	Leptospirales	Leptospiraceae	Leptospira	Leptospira wolbachii
3	Bacteria	Spirochaetes	Spirochaetia	Leptospirales	Leptospiraceae	Leptospira	Leptospira biflexa
3	Bacteria	Spirochaetes	Spirochaetia	Brachyspirales	Brachyspiraceae	Brachyspira	environmental samples
3	Bacteria	Spirochaetes	Spirochaetia	Brachyspirales	Brachyspiraceae	Brachyspira	Brachyspira pilosicoli
3	Bacteria	Proteobacteria	unclassified Proteobacteria	unclassified Proteobacteria (miscellaneous)	Proteobacteria bacterium ST_bin15		
3	Bacteria	Proteobacteria	delta/epsilon subdivisions	Epsilonproteobacteria	unclassified Epsilonproteobacteria	Sulfurovum	Sulfurovum sp. NBC37-1
3	Bacteria	Proteobacteria	delta/epsilon subdivisions	Epsilonproteobacteria	unclassified Epsilonproteobacteria	Sulfurovum	Sulfurovum sp. FS08-3
3	Bacteria	Proteobacteria	delta/epsilon subdivisions	Epsilonproteobacteria	unclassified Epsilonproteobacteria	Sulfurovum	Sulfurovum riftiae
3	Bacteria	Proteobacteria	delta/epsilon subdivisions	Epsilonproteobacteria	unclassified Epsilonproteobacteria	Nitratiruptor	Nitratiruptor tergarcus
3	Bacteria	Proteobacteria	delta/epsilon subdivisions	Epsilonproteobacteria	unclassified Epsilonproteobacteria	Nitratiruptor	Nitratiruptor sp. SB155-2
3	Bacteria	Proteobacteria	delta/epsilon subdivisions	Epsilonproteobacteria	Nautiliales	Nautiliaceae	Nautilia
3	Bacteria	Proteobacteria	delta/epsilon subdivisions	Epsilonproteobacteria	Campylobacterales	Helicobacteraceae	Sulfurimonas
3	Bacteria	Proteobacteria	delta/epsilon subdivisions	Epsilonproteobacteria	Campylobacterales	Helicobacteraceae	Helicobacter
3	Bacteria	Proteobacteria	delta/epsilon subdivisions	Epsilonproteobacteria	Campylobacterales	Helicobacteraceae	Helicobacter
3	Bacteria	Proteobacteria	delta/epsilon subdivisions	Epsilonproteobacteria	Campylobacterales	Helicobacteraceae	Helicobacter
3	Bacteria	Proteobacteria	delta/epsilon subdivisions	Epsilonproteobacteria	Campylobacterales	Helicobacteraceae	Helicobacter
3	Bacteria	Proteobacteria	delta/epsilon subdivisions	Epsilonproteobacteria	Campylobacterales	Helicobacteraceae	Helicobacter
3	Bacteria	Proteobacteria	delta/epsilon subdivisions	Epsilonproteobacteria	Campylobacterales	Campylobacteraceae	Sulfurospirillum
3	Bacteria	Proteobacteria	delta/epsilon subdivisions	Epsilonproteobacteria	Campylobacterales	Campylobacteraceae	Arcobacter
3	Bacteria	Proteobacteria	delta/epsilon subdivisions	Epsilonproteobacteria			
3	Bacteria	Proteobacteria	delta/epsilon subdivisions	Deltaproteobacteria	unclassified Deltaproteobacteria	unclassified Deltaproteobacteria (miscellaneous)	delta proteobacterium PSCGC 5342
3	Bacteria	Proteobacteria	delta/epsilon subdivisions	Deltaproteobacteria	unclassified Deltaproteobacteria	unclassified Deltaproteobacteria (miscellaneous)	Deltaproteobacteria bacterium RIFOXYB2_FULL_66_7
3	Bacteria	Proteobacteria	delta/epsilon subdivisions	Deltaproteobacteria	unclassified Deltaproteobacteria	unclassified Deltaproteobacteria (miscellaneous)	Deltaproteobacteria bacterium RIFCSPHIGHO2_12_FULL_43_9
3	Bacteria	Proteobacteria	delta/epsilon subdivisions	Deltaproteobacteria	unclassified Deltaproteobacteria	unclassified Deltaproteobacteria (miscellaneous)	Deltaproteobacteria bacterium RBG_19FT_COMBO_52_11
3	Bacteria	Proteobacteria	delta/epsilon subdivisions	Deltaproteobacteria	unclassified Deltaproteobacteria	unclassified Deltaproteobacteria (miscellaneous)	Deltaproteobacteria bacterium RBG_13_58_19
3	Bacteria	Proteobacteria	delta/epsilon subdivisions	Deltaproteobacteria	unclassified Deltaproteobacteria	unclassified Deltaproteobacteria (miscellaneous)	Deltaproteobacteria bacterium RBG_13_52_11b
3	Bacteria	Proteobacteria	delta/epsilon subdivisions	Deltaproteobacteria	unclassified Deltaproteobacteria	unclassified Deltaproteobacteria (miscellaneous)	Deltaproteobacteria bacterium RBG_13_47_9
3	Bacteria	Proteobacteria	delta/epsilon subdivisions	Deltaproteobacteria	unclassified Deltaproteobacteria	unclassified Deltaproteobacteria (miscellaneous)	Deltaproteobacteria bacterium GWC2_42_11
3	Bacteria	Proteobacteria	delta/epsilon subdivisions	Deltaproteobacteria	environmental samples	uncultured delta proteobacterium HF0070_10I02	
3	Bacteria	Proteobacteria	delta/epsilon subdivisions	Deltaproteobacteria	Syntrophobacterales	Syntrophobacteraceae	Syntrophobacter
3	Bacteria	Proteobacteria	delta/epsilon subdivisions	Deltaproteobacteria	Syntrophobacterales	Syntrophobacteraceae	Desulfacinum
3	Bacteria	Proteobacteria	delta/epsilon subdivisions	Deltaproteobacteria	Syntrophobacterales	Syntrophaceae	Syntrophus
3	Bacteria	Proteobacteria	delta/epsilon subdivisions	Deltaproteobacteria	Syntrophobacterales	Syntrophaceae	Desulfobacca
3	Bacteria	Proteobacteria	delta/epsilon subdivisions	Deltaproteobacteria	Syntrophobacterales	Syntrophaceae	
3	Bacteria	Proteobacteria	delta/epsilon subdivisions	Deltaproteobacteria	Myxococcales	Sorangiineae	Polyangiaceae
3	Bacteria	Proteobacteria	delta/epsilon subdivisions	Deltaproteobacteria	Myxococcales	Sorangiineae	Polyangiaceae
3	Bacteria	Proteobacteria	delta/epsilon subdivisions	Deltaproteobacteria	Myxococcales	Cystobacterineae	Myxococcaceae
3	Bacteria	Proteobacteria	delta/epsilon subdivisions	Deltaproteobacteria	Myxococcales	Cystobacterineae	Archangiaceae
3	Bacteria	Proteobacteria	delta/epsilon subdivisions	Deltaproteobacteria	Myxococcales	Cystobacterineae	Archangiaceae
3	Bacteria	Proteobacteria	delta/epsilon subdivisions	Deltaproteobacteria	Desulfuromonadales	unclassified Desulfuromonadales	Desulfuromonadales bacterium C00003107
3	Bacteria	Proteobacteria	delta/epsilon subdivisions	Deltaproteobacteria	Desulfuromonadales	unclassified Desulfuromonadales	Desulfuromonadales bacterium C00003068
3	Bacteria	Proteobacteria	delta/epsilon subdivisions	Deltaproteobacteria	Desulfuromonadales	Geobacteraceae	unclassified Geobacteraceae
3	Bacteria	Proteobacteria	delta/epsilon subdivisions	Deltaproteobacteria	Desulfuromonadales	Geobacteraceae	Geobacter
3	Bacteria	Proteobacteria	delta/epsilon subdivisions	Deltaproteobacteria	Desulfuromonadales	Desulfuromonadaceae	Pelobacter
3	Bacteria	Proteobacteria	delta/epsilon subdivisions	Deltaproteobacteria	Desulfuromonadales	Desulfuromonadaceae	Pelobacter
3	Bacteria	Proteobacteria	delta/epsilon subdivisions	Deltaproteobacteria	Desulfuromonadales	Desulfuromonadaceae	
3	Bacteria	Proteobacteria	delta/epsilon subdivisions	Deltaproteobacteria	Desulfurellales	Desulfurellaceae	Desulfurella
3	Bacteria	Proteobacteria	delta/epsilon subdivisions	Deltaproteobacteria	Desulfovibrionales	Desulfovibrionaceae	Bilophila
3	Bacteria	Proteobacteria	delta/epsilon subdivisions	Deltaproteobacteria	Desulfobacterales	unclassified Desulfobacterales	Desulfobacterales bacterium SG8_35
3	Bacteria	Proteobacteria	delta/epsilon subdivisions	Deltaproteobacteria	Desulfobacterales	unclassified Desulfobacterales	Desulfobacterales bacterium C00003106
3	Bacteria	Proteobacteria	delta/epsilon subdivisions	Deltaproteobacteria	Desulfobacterales	Desulfobulbaceae	unclassified Desulfobulbaceae
3	Bacteria	Proteobacteria	delta/epsilon subdivisions	Deltaproteobacteria	Desulfobacterales	Desulfobacteraceae	Desulfotignum
3	Bacteria	Proteobacteria	delta/epsilon subdivisions	Deltaproteobacteria	Desulfobacterales	Desulfobacteraceae	Desulfobacula
3	Bacteria	Proteobacteria	delta/epsilon subdivisions	Deltaproteobacteria	Desulfobacterales	Desulfobacteraceae	Desulfobacter
3	Bacteria	Proteobacteria	delta/epsilon subdivisions	Deltaproteobacteria	Desulfobacterales	Desulfobacteraceae	Desulfatibacillum
3	Bacteria	Proteobacteria	delta/epsilon subdivisions	Deltaproteobacteria	Candidatus Desulfofervidaceae	Candidatus Desulfofervidus	Candidatus Desulfofervidus auxilii
3	Bacteria	Proteobacteria	delta/epsilon subdivisions	Deltaproteobacteria	Bdellovibrionales	Bacteriovoracaceae	Bacteriovorax
3	Bacteria	Proteobacteria	Zetaproteobacteria	unclassified Zetaproteobacteria	Zetaproteobacteria bacterium TAG-1		
3	Bacteria	Proteobacteria	Gammaproteobacteria	unclassified Gammaproteobacteria	unclassified Gammaproteobacteria (miscellaneous)	methanotrophic bacterial endosymbiont of Bathymodiolus sp.	
3	Bacteria	Proteobacteria	Gammaproteobacteria	unclassified Gammaproteobacteria	unclassified Gammaproteobacteria (miscellaneous)	gamma proteobacterium L18	
3	Bacteria	Proteobacteria	Gammaproteobacteria	unclassified Gammaproteobacteria	unclassified Gammaproteobacteria (miscellaneous)	Gammaproteobacteria bacterium RIFOXYD12_FULL_61_37	
3	Bacteria	Proteobacteria	Gammaproteobacteria	unclassified Gammaproteobacteria	unclassified Gammaproteobacteria (miscellaneous)	Gammaproteobacteria bacterium RIFCSPHIGHO2_12_FULL_38_14	
3	Bacteria	Proteobacteria	Gammaproteobacteria	unclassified Gammaproteobacteria	unclassified Gammaproteobacteria (miscellaneous)	Gammaproteobacteria bacterium RIFCSPHIGHO2_12_FULL_36_30	
3	Bacteria	Proteobacteria	Gammaproteobacteria	unclassified Gammaproteobacteria	sulfur-oxidizing symbionts	endosymbiont of Ridgeia piscesae	
3	Bacteria	Proteobacteria	Gammaproteobacteria	unclassified Gammaproteobacteria	sulfur-oxidizing symbionts	Solemya elarraichensis gill symbiont	
3	Bacteria	Proteobacteria	Gammaproteobacteria	unclassified Gammaproteobacteria	sulfur-oxidizing symbionts	Candidatus Thiodiazotropha	
3	Bacteria	Proteobacteria	Gammaproteobacteria	unclassified Gammaproteobacteria	sulfur-oxidizing symbionts	Bathymodiolus azoricus thioautotrophic gill symbiont	
3	Bacteria	Proteobacteria	Gammaproteobacteria	unclassified Gammaproteobacteria	Wohlfahrtiimonas	Wohlfahrtiimonas chitiniclastica	
3	Bacteria	Proteobacteria	Gammaproteobacteria	unclassified Gammaproteobacteria	Wohlfahrtiimonas		
3	Bacteria	Proteobacteria	Gammaproteobacteria	unclassified Gammaproteobacteria	SAR86 cluster	SAR86 cluster bacterium SAR86A	
3	Bacteria	Proteobacteria	Gammaproteobacteria	unclassified Gammaproteobacteria	Gallaecimonas		
3	Bacteria	Proteobacteria	Gammaproteobacteria	unclassified Gammaproteobacteria	Competibacteraceae	Candidatus Contendobacter	Candidatus Contendobacter odensis
3	Bacteria	Proteobacteria	Gammaproteobacteria	unclassified Gammaproteobacteria	Competibacteraceae	Candidatus Competibacter	Candidatus Competibacter denitrificans
3	Bacteria	Proteobacteria	Gammaproteobacteria	unclassified Gammaproteobacteria			
3	Bacteria	Proteobacteria	Gammaproteobacteria	environmental samples	uncultured gamma proteobacterium		
3	Bacteria	Proteobacteria	Gammaproteobacteria	Xanthomonadales	unclassified Xanthomonadales	Xanthomonadales bacterium 66-474	
3	Bacteria	Proteobacteria	Gammaproteobacteria	Xanthomonadales	Xanthomonadaceae	Xanthomonas	Xanthomonas sp. WHRI 8848
3	Bacteria	Proteobacteria	Gammaproteobacteria	Xanthomonadales	Xanthomonadaceae	Xanthomonas	Xanthomonas sacchari
3	Bacteria	Proteobacteria	Gammaproteobacteria	Xanthomonadales	Xanthomonadaceae	Xanthomonas	Xanthomonas citri group
3	Bacteria	Proteobacteria	Gammaproteobacteria	Xanthomonadales	Xanthomonadaceae	Xanthomonas	Xanthomonas citri group
3	Bacteria	Proteobacteria	Gammaproteobacteria	Xanthomonadales	Xanthomonadaceae	Xanthomonas	Xanthomonas albilineans
3	Bacteria	Proteobacteria	Gammaproteobacteria	Xanthomonadales	Xanthomonadaceae	Stenotrophomonas	Stenotrophomonas terrae
3	Bacteria	Proteobacteria	Gammaproteobacteria	Xanthomonadales	Xanthomonadaceae	Stenotrophomonas	Stenotrophomonas daejeonensis
3	Bacteria	Proteobacteria	Gammaproteobacteria	Xanthomonadales	Xanthomonadaceae	Lysobacter	Lysobacter sp. yr284
3	Bacteria	Proteobacteria	Gammaproteobacteria	Xanthomonadales	Xanthomonadaceae	Lysobacter	Lysobacter sp. Root690
3	Bacteria	Proteobacteria	Gammaproteobacteria	Xanthomonadales	Rhodanobacteraceae	Rhodanobacter	Rhodanobacter thiooxydans
3	Bacteria	Proteobacteria	Gammaproteobacteria	Xanthomonadales	Rhodanobacteraceae	Rhodanobacter	Rhodanobacter sp. C03
3	Bacteria	Proteobacteria	Gammaproteobacteria	Xanthomonadales	Rhodanobacteraceae	Rhodanobacter	Rhodanobacter sp. B04
3	Bacteria	Proteobacteria	Gammaproteobacteria	Xanthomonadales	Rhodanobacteraceae	Rhodanobacter	Rhodanobacter denitrificans
3	Bacteria	Proteobacteria	Gammaproteobacteria	Xanthomonadales	Rhodanobacteraceae	Luteibacter	Luteibacter sp. UNCMF366Tsu5.1
3	Bacteria	Proteobacteria	Gammaproteobacteria	Vibrionales	Vibrionaceae	Vibrio	Vibrio tritonius
3	Bacteria	Proteobacteria	Gammaproteobacteria	Vibrionales	Vibrionaceae	Vibrio	Vibrio splendidus
3	Bacteria	Proteobacteria	Gammaproteobacteria	Vibrionales	Vibrionaceae	Vibrio	Vibrio pacinii
3	Bacteria	Proteobacteria	Gammaproteobacteria	Vibrionales	Vibrionaceae	Vibrio	Vibrio harveyi group
3	Bacteria	Proteobacteria	Gammaproteobacteria	Vibrionales	Vibrionaceae	Vibrio	Vibrio furnissii
3	Bacteria	Proteobacteria	Gammaproteobacteria	Vibrionales	Vibrionaceae	Vibrio	Vibrio cincinnatiensis
3	Bacteria	Proteobacteria	Gammaproteobacteria	Vibrionales	Vibrionaceae	Photobacterium	Photobacterium sp. 13-12
3	Bacteria	Proteobacteria	Gammaproteobacteria	Vibrionales	Vibrionaceae	Photobacterium	Photobacterium aquae
3	Bacteria	Proteobacteria	Gammaproteobacteria	Vibrionales	Vibrionaceae	Enterovibrio	Enterovibrio nigricans
3	Bacteria	Proteobacteria	Gammaproteobacteria	Vibrionales	Vibrionaceae	Aliivibrio	Aliivibrio fischeri
3	Bacteria	Proteobacteria	Gammaproteobacteria	Thiotrichales	Thiotrichaceae	Candidatus Marithrix	Candidatus Marithrix sp. Canyon 246
3	Bacteria	Proteobacteria	Gammaproteobacteria	Thiotrichales	Thiotrichaceae	Achromatium	Candidatus Achromatium palustre
3	Bacteria	Proteobacteria	Gammaproteobacteria	Thiotrichales	Thiotrichaceae	Achromatium	Achromatium sp. WMS2
3	Bacteria	Proteobacteria	Gammaproteobacteria	Thiotrichales	Thiotrichaceae	Achromatium	
3	Bacteria	Proteobacteria	Gammaproteobacteria	Thiotrichales	Piscirickettsiaceae	Thiomicrospira	
3	Bacteria	Proteobacteria	Gammaproteobacteria	Thiotrichales	Piscirickettsiaceae	Thioalkalimicrobium	Thioalkalimicrobium cyclicum
3	Bacteria	Proteobacteria	Gammaproteobacteria	Thiotrichales	Piscirickettsiaceae	Methylophaga	Methylophaga nitratireducenticrescens
3	Bacteria	Proteobacteria	Gammaproteobacteria	Thiotrichales	Piscirickettsiaceae	Methylophaga	Methylophaga muralis
3	Bacteria	Proteobacteria	Gammaproteobacteria	Thiotrichales	Francisellaceae	Francisella	Francisella sp. TX077310
3	Bacteria	Proteobacteria	Gammaproteobacteria	Thiotrichales	Francisellaceae	Francisella	

3	Bacteria	Proteobacteria	Gammaproteobacteria	Pseudomonadales	Pseudomonadaceae	Pseudomonas	Pseudomonas toyotomiensis
3	Bacteria	Proteobacteria	Gammaproteobacteria	Pseudomonadales	Pseudomonadaceae	Pseudomonas	Pseudomonas syringae group
3	Bacteria	Proteobacteria	Gammaproteobacteria	Pseudomonadales	Pseudomonadaceae	Pseudomonas	Pseudomonas stutzeri group
3	Bacteria	Proteobacteria	Gammaproteobacteria	Pseudomonadales	Pseudomonadaceae	Pseudomonas	Pseudomonas stutzeri group
3	Bacteria	Proteobacteria	Gammaproteobacteria	Pseudomonadales	Pseudomonadaceae	Pseudomonas	Pseudomonas sp. VI4.1
3	Bacteria	Proteobacteria	Gammaproteobacteria	Pseudomonadales	Pseudomonadaceae	Pseudomonas	Pseudomonas sp. UW4
3	Bacteria	Proteobacteria	Gammaproteobacteria	Pseudomonadales	Pseudomonadaceae	Pseudomonas	Pseudomonas sp. TTU2014-080ASC
3	Bacteria	Proteobacteria	Gammaproteobacteria	Pseudomonadales	Pseudomonadaceae	Pseudomonas	Pseudomonas sp. R37(2017)
3	Bacteria	Proteobacteria	Gammaproteobacteria	Pseudomonadales	Pseudomonadaceae	Pseudomonas	Pseudomonas sp. PI1
3	Bacteria	Proteobacteria	Gammaproteobacteria	Pseudomonadales	Pseudomonadaceae	Pseudomonas	Pseudomonas sp. Leaf127
3	Bacteria	Proteobacteria	Gammaproteobacteria	Pseudomonadales	Pseudomonadaceae	Pseudomonas	Pseudomonas sp. GM21
3	Bacteria	Proteobacteria	Gammaproteobacteria	Pseudomonadales	Pseudomonadaceae	Pseudomonas	Pseudomonas sp. ES3-33
3	Bacteria	Proteobacteria	Gammaproteobacteria	Pseudomonadales	Pseudomonadaceae	Pseudomonas	Pseudomonas sp. CHM02
3	Bacteria	Proteobacteria	Gammaproteobacteria	Pseudomonadales	Pseudomonadaceae	Pseudomonas	Pseudomonas sp. Ant30-3
3	Bacteria	Proteobacteria	Gammaproteobacteria	Pseudomonadales	Pseudomonadaceae	Pseudomonas	Pseudomonas sagittaria
3	Bacteria	Proteobacteria	Gammaproteobacteria	Pseudomonadales	Pseudomonadaceae	Pseudomonas	Pseudomonas sabulinigri
3	Bacteria	Proteobacteria	Gammaproteobacteria	Pseudomonadales	Pseudomonadaceae	Pseudomonas	Pseudomonas putida group
3	Bacteria	Proteobacteria	Gammaproteobacteria	Pseudomonadales	Pseudomonadaceae	Pseudomonas	Pseudomonas punonensis
3	Bacteria	Proteobacteria	Gammaproteobacteria	Pseudomonadales	Pseudomonadaceae	Pseudomonas	Pseudomonas peli
3	Bacteria	Proteobacteria	Gammaproteobacteria	Pseudomonadales	Pseudomonadaceae	Pseudomonas	Pseudomonas parafulva
3	Bacteria	Proteobacteria	Gammaproteobacteria	Pseudomonadales	Pseudomonadaceae	Pseudomonas	Pseudomonas otitidis
3	Bacteria	Proteobacteria	Gammaproteobacteria	Pseudomonadales	Pseudomonadaceae	Pseudomonas	Pseudomonas linyingensis
3	Bacteria	Proteobacteria	Gammaproteobacteria	Pseudomonadales	Pseudomonadaceae	Pseudomonas	Pseudomonas japonica
3	Bacteria	Proteobacteria	Gammaproteobacteria	Pseudomonadales	Pseudomonadaceae	Pseudomonas	Pseudomonas guangdongensis
3	Bacteria	Proteobacteria	Gammaproteobacteria	Pseudomonadales	Pseudomonadaceae	Pseudomonas	Pseudomonas gingeri
3	Bacteria	Proteobacteria	Gammaproteobacteria	Pseudomonadales	Pseudomonadaceae	Pseudomonas	Pseudomonas fluorescens group
3	Bacteria	Proteobacteria	Gammaproteobacteria	Pseudomonadales	Pseudomonadaceae	Pseudomonas	Pseudomonas fluorescens group
3	Bacteria	Proteobacteria	Gammaproteobacteria	Pseudomonadales	Pseudomonadaceae	Pseudomonas	Pseudomonas delhiensis
3	Bacteria	Proteobacteria	Gammaproteobacteria	Pseudomonadales	Pseudomonadaceae	Pseudomonas	Pseudomonas cremoricolorata
3	Bacteria	Proteobacteria	Gammaproteobacteria	Pseudomonadales	Pseudomonadaceae	Pseudomonas	Pseudomonas borbori
3	Bacteria	Proteobacteria	Gammaproteobacteria	Pseudomonadales	Pseudomonadaceae	Pseudomonas	Pseudomonas aeruginosa group
3	Bacteria	Proteobacteria	Gammaproteobacteria	Pseudomonadales	Pseudomonadaceae	Pseudomonas	Pseudomonas aeruginosa group
3	Bacteria	Proteobacteria	Gammaproteobacteria	Pseudomonadales	Pseudomonadaceae	Pseudomonas	Pseudomonas aeruginosa group
3	Bacteria	Proteobacteria	Gammaproteobacteria	Pseudomonadales	Moraxellaceae	Moraxella	Moraxella oblonga
3	Bacteria	Proteobacteria	Gammaproteobacteria	Pseudomonadales	Moraxellaceae	Acinetobacter	Acinetobacter soli
3	Bacteria	Proteobacteria	Gammaproteobacteria	Pseudomonadales	Moraxellaceae	Acinetobacter	Acinetobacter nectaris
3	Bacteria	Proteobacteria	Gammaproteobacteria	Pseudomonadales	Moraxellaceae	Acinetobacter	Acinetobacter genomosp. 33YU
3	Bacteria	Proteobacteria	Gammaproteobacteria	Pseudomonadales	Moraxellaceae	Acinetobacter	Acinetobacter calcoaceticus/baumannii complex
3	Bacteria	Proteobacteria	Gammaproteobacteria	Pseudomonadales	Moraxellaceae	Acinetobacter	Acinetobacter brisouii
3	Bacteria	Proteobacteria	Gammaproteobacteria	Pseudomonadales	Moraxellaceae	Acinetobacter	Acinetobacter boissieri
3	Bacteria	Proteobacteria	Gammaproteobacteria	Pasteurellales	Pasteurellaceae	unclassified Pasteurellaceae	unclassified Pasteurellaceae (miscellaneous)
3	Bacteria	Proteobacteria	Gammaproteobacteria	Pasteurellales	Pasteurellaceae	Mannheimia	Mannheimia varigena
3	Bacteria	Proteobacteria	Gammaproteobacteria	Pasteurellales	Pasteurellaceae	Haemophilus	Haemophilus influenzae
3	Bacteria	Proteobacteria	Gammaproteobacteria	Pasteurellales	Pasteurellaceae	Avibacterium	Avibacterium paragallinarum
3	Bacteria	Proteobacteria	Gammaproteobacteria	Orbales	Orbaceae	Gilliamella	
3	Bacteria	Proteobacteria	Gammaproteobacteria	Oceanospirillales	unclassified Oceanospirillales	unclassified Oceanospirillales (miscellaneous)	Oceanospirillales bacterium LUC14_002_19_P2
3	Bacteria	Proteobacteria	Gammaproteobacteria	Oceanospirillales	Saccharospirillaceae	Reinekea	Reinekea blandensis
3	Bacteria	Proteobacteria	Gammaproteobacteria	Oceanospirillales	Oceanospirillaceae	Thalassolituus	Thalassolituus sp. HI0120
3	Bacteria	Proteobacteria	Gammaproteobacteria	Oceanospirillales	Oceanospirillaceae	Pseudospirillum	Pseudospirillum japonicum
3	Bacteria	Proteobacteria	Gammaproteobacteria	Oceanospirillales	Oceanospirillaceae	Oceanospirillum	Oceanospirillum multiglobuliferum
3	Bacteria	Proteobacteria	Gammaproteobacteria	Oceanospirillales	Oceanospirillaceae	Neptuniibacter	Neptuniibacter caesariensis
3	Bacteria	Proteobacteria	Gammaproteobacteria	Oceanospirillales	Oceanospirillaceae	Neptuniibacter	
3	Bacteria	Proteobacteria	Gammaproteobacteria	Oceanospirillales	Oceanospirillaceae	Marinospirillum	Marinospirillum alkaliphilum
3	Bacteria	Proteobacteria	Gammaproteobacteria	Oceanospirillales	Oceanospirillaceae	Marinomonas	Marinomonas ushuaiensis
3	Bacteria	Proteobacteria	Gammaproteobacteria	Oceanospirillales	Oceanospirillaceae	Marinomonas	Marinomonas profundimaris
3	Bacteria	Proteobacteria	Gammaproteobacteria	Oceanospirillales	Halomonadaceae	Salinicola	
3	Bacteria	Proteobacteria	Gammaproteobacteria	Oceanospirillales	Halomonadaceae	Kushneria	Kushneria aurantia
3	Bacteria	Proteobacteria	Gammaproteobacteria	Oceanospirillales	Halomonadaceae	Halomonas	Halomonas zincidurans
3	Bacteria	Proteobacteria	Gammaproteobacteria	Oceanospirillales	Halomonadaceae	Halomonas	Halomonas sp. S2151
3	Bacteria	Proteobacteria	Gammaproteobacteria	Oceanospirillales	Halomonadaceae	Halomonas	Halomonas sp. KO116
3	Bacteria	Proteobacteria	Gammaproteobacteria	Oceanospirillales	Halomonadaceae	Halomonas	Halomonas sp. J8380
3	Bacteria	Proteobacteria	Gammaproteobacteria	Oceanospirillales	Halomonadaceae	Halomonas	Halomonas sp. 1513
3	Bacteria	Proteobacteria	Gammaproteobacteria	Oceanospirillales	Halomonadaceae	Halomonas	Halomonas lutea
3	Bacteria	Proteobacteria	Gammaproteobacteria	Oceanospirillales	Halomonadaceae	Halomonas	Halomonas korlensis
3	Bacteria	Proteobacteria	Gammaproteobacteria	Oceanospirillales	Halomonadaceae	Halomonas	Halomonas daqiaonensis
3	Bacteria	Proteobacteria	Gammaproteobacteria	Oceanospirillales	Halomonadaceae	Cobetia	Cobetia crustatorum
3	Bacteria	Proteobacteria	Gammaproteobacteria	Oceanospirillales	Halomonadaceae	Chromohalobacter	Chromohalobacter salexigens
3	Bacteria	Proteobacteria	Gammaproteobacteria	Oceanospirillales	Halomonadaceae	Chromohalobacter	
3	Bacteria	Proteobacteria	Gammaproteobacteria	Oceanospirillales	Halomonadaceae	Aidingimonas	Aidingimonas halophila
3	Bacteria	Proteobacteria	Gammaproteobacteria	Oceanospirillales	Hahellaceae	Endozoicomonas	Endozoicomonas sp. (ex Bugula neritina AB1)
3	Bacteria	Proteobacteria	Gammaproteobacteria	Oceanospirillales	Hahellaceae	Endozoicomonas	Endozoicomonas atrinae
3	Bacteria	Proteobacteria	Gammaproteobacteria	Oceanospirillales	Alcanivoracaceae	Alcanivorax	Alcanivorax hongdengensis
3	Bacteria	Proteobacteria	Gammaproteobacteria	Nevskiales	Sinobacteraceae	Solimonas	Solimonas variicoloris
3	Bacteria	Proteobacteria	Gammaproteobacteria	Nevskiales	Sinobacteraceae		
3	Bacteria	Proteobacteria	Gammaproteobacteria	Methylococcales	Methylococcaceae	Methylomonas	Methylomonas sp. LW13
3	Bacteria	Proteobacteria	Gammaproteobacteria	Methylococcales	Methylococcaceae	Methylomicrobium	Methylomicrobium agile
3	Bacteria	Proteobacteria	Gammaproteobacteria	Legionellales	unclassified Legionellales	Legionellales bacterium RIFCSPHIGHO2_12_FULL_37_14	
3	Bacteria	Proteobacteria	Gammaproteobacteria	Legionellales	Legionellaceae	Legionella	Legionella oakridgensis
3	Bacteria	Proteobacteria	Gammaproteobacteria	Legionellales	Legionellaceae	Legionella	Legionella feeleii
3	Bacteria	Proteobacteria	Gammaproteobacteria	Legionellales	Legionellaceae	Legionella	Legionella fairfieldensis
3	Bacteria	Proteobacteria	Gammaproteobacteria	Legionellales	Coxiellaceae	Coxiella	Coxiella sp. RIFCSPHIGHO2_12_FULL_44_14
3	Bacteria	Proteobacteria	Gammaproteobacteria	Enterobacterales	Yersiniaceae	Yersinia	Yersinia frederiksenii
3	Bacteria	Proteobacteria	Gammaproteobacteria	Enterobacterales	Pectobacteriaceae	Brenneria	Brenneria sp. EniD312
3	Bacteria	Proteobacteria	Gammaproteobacteria	Enterobacterales	Morganellaceae	Photorhabdus	Photorhabdus temperata
3	Bacteria	Proteobacteria	Gammaproteobacteria	Enterobacterales	Morganellaceae	Photorhabdus	Photorhabdus asymbiotica
3	Bacteria	Proteobacteria	Gammaproteobacteria	Enterobacterales	Morganellaceae		
3	Bacteria	Proteobacteria	Gammaproteobacteria	Enterobacterales	Erwiniaceae	Pantoea	Pantoea ananatis
3	Bacteria	Proteobacteria	Gammaproteobacteria	Enterobacterales	Erwiniaceae	Pantoea	Pantoea agglomerans group
3	Bacteria	Proteobacteria	Gammaproteobacteria	Enterobacterales	Erwiniaceae	Erwinia	Erwinia persicina
3	Bacteria	Proteobacteria	Gammaproteobacteria	Enterobacterales	Erwiniaceae	Buchnera	Buchnera aphidicola
3	Bacteria	Proteobacteria	Gammaproteobacteria	Enterobacterales	Erwiniaceae		
3	Bacteria	Proteobacteria	Gammaproteobacteria	Enterobacterales	Enterobacteriaceae	unclassified Enterobacteriaceae	unclassified Enterobacteriaceae (miscellaneous)
3	Bacteria	Proteobacteria	Gammaproteobacteria	Enterobacterales	Enterobacteriaceae	Trabulsiella	Trabulsiella odontotermitis
3	Bacteria	Proteobacteria	Gammaproteobacteria	Enterobacterales	Enterobacteriaceae	Salmonella	Salmonella enterica
3	Bacteria	Proteobacteria	Gammaproteobacteria	Enterobacterales	Enterobacteriaceae	Enterobacter	Enterobacter cloacae complex
3	Bacteria	Proteobacteria	Gammaproteobacteria	Enterobacterales	Enterobacteriaceae	Enterobacter	Enterobacter cloacae complex
3	Bacteria	Proteobacteria	Gammaproteobacteria	Enterobacterales	Enterobacteriaceae	Enterobacter	Enterobacter cloacae complex
3	Bacteria	Proteobacteria	Gammaproteobacteria	Enterobacterales	Enterobacteriaceae	Enterobacter	
3	Bacteria	Proteobacteria	Gammaproteobacteria	Enterobacterales	Enterobacteriaceae	Cronobacter	
3	Bacteria	Proteobacteria	Gammaproteobacteria	Enterobacterales	Enterobacteriaceae	Citrobacter	Citrobacter freundii complex
3	Bacteria	Proteobacteria	Gammaproteobacteria	Enterobacterales	Enterobacteriaceae	Citrobacter	
3	Bacteria	Proteobacteria	Gammaproteobacteria	Enterobacterales	Enterobacteriaceae	Candidatus Ishikawaella	Candidatus Ishikawaella capsulata
3	Bacteria	Proteobacteria	Gammaproteobacteria	Chromatiales	Ectothiorhodospiraceae	Thiorhodospira	Thiorhodospira sibirica
3	Bacteria	Proteobacteria	Gammaproteobacteria	Chromatiales	Ectothiorhodospiraceae	Thioalkalivibrio	Thioalkalivibrio sp. ALSr1
3	Bacteria	Proteobacteria	Gammaproteobacteria	Chromatiales	Ectothiorhodospiraceae	Thioalkalivibrio	Thioalkalivibrio nitratireducens
3	Bacteria	Proteobacteria	Gammaproteobacteria	Chromatiales	Ectothiorhodospiraceae	Halorhodospira	Halorhodospira halophila
3	Bacteria	Proteobacteria	Gammaproteobacteria	Chromatiales	Ectothiorhodospiraceae	Halorhodospira	Halorhodospira halochloris
3	Bacteria	Proteobacteria	Gammaproteobacteria	Chromatiales	Chromatiaceae	Rheinheimera	Rheinheimera sp. KL1
3	Bacteria	Proteobacteria	Gammaproteobacteria	Chromatiales	Chromatiaceae	Rheinheimera	Rheinheimera salexigens
3	Bacteria	Proteobacteria	Gammaproteobacteria	Chromatiales	Chromatiaceae	Rheinheimera	Rheinheimera perlucida
3	Bacteria	Proteobacteria	Gammaproteobacteria	Chromatiales	Chromatiaceae	Nitrosococcus	Nitrosococcus watsonii
3	Bacteria	Proteobacteria	Gammaproteobacteria	Chromatiales	Chromatiaceae	Marichromatium	Marichromatium gracile
3	Bacteria	Proteobacteria	Gammaproteobacteria	Chromatiales	Chromatiaceae	Arsukibacterium	Arsukibacterium ikkense
3	Bacteria	Proteobacteria	Gammaproteobacteria	Cellvibrionales	Microbulbiferaceae	Microbulbifer	Microbulbifer variabilis
3	Bacteria	Proteobacteria	Gammaproteobacteria	Cellvibrionales	Microbulbiferaceae	Microbulbifer	Microbulbifer thermotolerans
3	Bacteria	Proteobacteria	Gammaproteobacteria	Cellvibrionales	Microbulbiferaceae	Microbulbifer	Microbulbifer sp. HZ11
3	Bacteria	Proteobacteria	Gammaproteobacteria	Cellvibrionales	Microbulbiferaceae	Microbulbifer	Microbulbifer marinus
[illegible]	[illegible]	[illegible]	[illegible]	[illegible]	[illegible]	[illegible]	[illegible]

3	Bacteria	FCB group	Bacteroidetes/Chlorobi group	Bacteroidetes	Cytophagia	Cytophagales	Hymenobacteraceae
3	Bacteria	FCB group	Bacteroidetes/Chlorobi group	Bacteroidetes	Cytophagia	Cytophagales	Hymenobacteraceae
3	Bacteria	FCB group	Bacteroidetes/Chlorobi group	Bacteroidetes	Cytophagia	Cytophagales	Hymenobacteraceae
3	Bacteria	FCB group	Bacteroidetes/Chlorobi group	Bacteroidetes	Cytophagia	Cytophagales	Hymenobacteraceae
3	Bacteria	FCB group	Bacteroidetes/Chlorobi group	Bacteroidetes	Cytophagia	Cytophagales	Hymenobacteraceae
3	Bacteria	FCB group	Bacteroidetes/Chlorobi group	Bacteroidetes	Cytophagia	Cytophagales	Hymenobacteraceae
3	Bacteria	FCB group	Bacteroidetes/Chlorobi group	Bacteroidetes	Cytophagia	Cytophagales	Hymenobacteraceae
3	Bacteria	FCB group	Bacteroidetes/Chlorobi group	Bacteroidetes	Cytophagia	Cytophagales	Hymenobacteraceae
3	Bacteria	FCB group	Bacteroidetes/Chlorobi group	Bacteroidetes	Cytophagia	Cytophagales	Hymenobacteraceae
3	Bacteria	FCB group	Bacteroidetes/Chlorobi group	Bacteroidetes	Cytophagia	Cytophagales	Hymenobacteraceae
3	Bacteria	FCB group	Bacteroidetes/Chlorobi group	Bacteroidetes	Cytophagia	Cytophagales	Hymenobacteraceae
3	Bacteria	FCB group	Bacteroidetes/Chlorobi group	Bacteroidetes	Cytophagia	Cytophagales	Hymenobacteraceae
3	Bacteria	FCB group	Bacteroidetes/Chlorobi group	Bacteroidetes	Cytophagia	Cytophagales	Hymenobacteraceae
3	Bacteria	FCB group	Bacteroidetes/Chlorobi group	Bacteroidetes	Cytophagia	Cytophagales	Flammeovirgaceae
3	Bacteria	FCB group	Bacteroidetes/Chlorobi group	Bacteroidetes	Cytophagia	Cytophagales	Flammeovirgaceae
3	Bacteria	FCB group	Bacteroidetes/Chlorobi group	Bacteroidetes	Cytophagia	Cytophagales	Flammeovirgaceae
3	Bacteria	FCB group	Bacteroidetes/Chlorobi group	Bacteroidetes	Cytophagia	Cytophagales	Flammeovirgaceae
3	Bacteria	FCB group	Bacteroidetes/Chlorobi group	Bacteroidetes	Cytophagia	Cytophagales	Cytophagaceae
3	Bacteria	FCB group	Bacteroidetes/Chlorobi group	Bacteroidetes	Cytophagia	Cytophagales	Cytophagaceae
3	Bacteria	FCB group	Bacteroidetes/Chlorobi group	Bacteroidetes	Cytophagia	Cytophagales	Cyclobacteriaceae
3	Bacteria	FCB group	Bacteroidetes/Chlorobi group	Bacteroidetes	Cytophagia	Cytophagales	Cyclobacteriaceae
3	Bacteria	FCB group	Bacteroidetes/Chlorobi group	Bacteroidetes	Cytophagia	Cytophagales	Cyclobacteriaceae
3	Bacteria	FCB group	Bacteroidetes/Chlorobi group	Bacteroidetes	Cytophagia	Cytophagales	Cyclobacteriaceae
3	Bacteria	FCB group	Bacteroidetes/Chlorobi group	Bacteroidetes	Cytophagia	Cytophagales	Cyclobacteriaceae
3	Bacteria	FCB group	Bacteroidetes/Chlorobi group	Bacteroidetes	Cytophagia	Cytophagales	Cyclobacteriaceae
3	Bacteria	FCB group	Bacteroidetes/Chlorobi group	Bacteroidetes	Cytophagia	Cytophagales	Cyclobacteriaceae
3	Bacteria	FCB group	Bacteroidetes/Chlorobi group	Bacteroidetes	Cytophagia	Cytophagales	Bernardetiaceae
3	Bacteria	FCB group	Bacteroidetes/Chlorobi group	Bacteroidetes	Chitinophagia	Chitinophagales	Chitinophagaceae
3	Bacteria	FCB group	Bacteroidetes/Chlorobi group	Bacteroidetes	Chitinophagia	Chitinophagales	Chitinophagaceae
3	Bacteria	FCB group	Bacteroidetes/Chlorobi group	Bacteroidetes	Chitinophagia	Chitinophagales	Chitinophagaceae
3	Bacteria	FCB group	Bacteroidetes/Chlorobi group	Bacteroidetes	Chitinophagia	Chitinophagales	Chitinophagaceae
3	Bacteria	FCB group	Bacteroidetes/Chlorobi group	Bacteroidetes	Bacteroidia	Marinilabiliales	
3	Bacteria	FCB group	Bacteroidetes/Chlorobi group	Bacteroidetes	Bacteroidia	Bacteroidales	unclassified Bacteroidales
3	Bacteria	FCB group	Bacteroidetes/Chlorobi group	Bacteroidetes	Bacteroidia	Bacteroidales	Rikenellaceae
3	Bacteria	FCB group	Bacteroidetes/Chlorobi group	Bacteroidetes	Bacteroidia	Bacteroidales	Rikenellaceae
3	Bacteria	FCB group	Bacteroidetes/Chlorobi group	Bacteroidetes	Bacteroidia	Bacteroidales	Rikenellaceae
3	Bacteria	FCB group	Bacteroidetes/Chlorobi group	Bacteroidetes	Bacteroidia	Bacteroidales	Prevotellaceae
3	Bacteria	FCB group	Bacteroidetes/Chlorobi group	Bacteroidetes	Bacteroidia	Bacteroidales	Prevotellaceae
3	Bacteria	FCB group	Bacteroidetes/Chlorobi group	Bacteroidetes	Bacteroidia	Bacteroidales	Prevotellaceae
3	Bacteria	FCB group	Bacteroidetes/Chlorobi group	Bacteroidetes	Bacteroidia	Bacteroidales	Prevotellaceae
3	Bacteria	FCB group	Bacteroidetes/Chlorobi group	Bacteroidetes	Bacteroidia	Bacteroidales	Prevotellaceae
3	Bacteria	FCB group	Bacteroidetes/Chlorobi group	Bacteroidetes	Bacteroidia	Bacteroidales	Prevotellaceae
3	Bacteria	FCB group	Bacteroidetes/Chlorobi group	Bacteroidetes	Bacteroidia	Bacteroidales	Prevotellaceae
3	Bacteria	FCB group	Bacteroidetes/Chlorobi group	Bacteroidetes	Bacteroidia	Bacteroidales	Prevotellaceae
3	Bacteria	FCB group	Bacteroidetes/Chlorobi group	Bacteroidetes	Bacteroidia	Bacteroidales	Prevotellaceae
3	Bacteria	FCB group	Bacteroidetes/Chlorobi group	Bacteroidetes	Bacteroidia	Bacteroidales	Prevotellaceae
3	Bacteria	FCB group	Bacteroidetes/Chlorobi group	Bacteroidetes	Bacteroidia	Bacteroidales	Prevotellaceae
3	Bacteria	FCB group	Bacteroidetes/Chlorobi group	Bacteroidetes	Bacteroidia	Bacteroidales	Prevotellaceae
3	Bacteria	FCB group	Bacteroidetes/Chlorobi group	Bacteroidetes	Bacteroidia	Bacteroidales	Prevotellaceae
[illegible]	[illegible]	[illegible]	[illegible]	[illegible]	[illegible]	[illegible]	[illegible]

2	Bacteria	Terrabacteria group	Firmicutes	Bacilli	Lactobacillales	Enterococcaceae	Enterococcus
2	Bacteria	Terrabacteria group	Firmicutes	Bacilli	Lactobacillales	Enterococcaceae	Enterococcus
2	Bacteria	Terrabacteria group	Firmicutes	Bacilli	Lactobacillales	Enterococcaceae	Enterococcus
2	Bacteria	Terrabacteria group	Firmicutes	Bacilli	Lactobacillales	Enterococcaceae	Enterococcus
2	Bacteria	Terrabacteria group	Firmicutes	Bacilli	Lactobacillales	Carnobacteriaceae	Marinilactibacillus
2	Bacteria	Terrabacteria group	Firmicutes	Bacilli	Lactobacillales	Carnobacteriaceae	Jeotgalibaca
2	Bacteria	Terrabacteria group	Firmicutes	Bacilli	Lactobacillales	Carnobacteriaceae	Granulicatella
2	Bacteria	Terrabacteria group	Firmicutes	Bacilli	Lactobacillales	Carnobacteriaceae	Granulicatella
2	Bacteria	Terrabacteria group	Firmicutes	Bacilli	Lactobacillales	Carnobacteriaceae	Alkalibacterium
2	Bacteria	Terrabacteria group	Firmicutes	Bacilli	Lactobacillales	Aerococcaceae	Eremococcus
2	Bacteria	Terrabacteria group	Firmicutes	Bacilli	Lactobacillales	Aerococcaceae	Dolosicoccus
2	Bacteria	Terrabacteria group	Firmicutes	Bacilli	Lactobacillales	Aerococcaceae	Aerococcus
2	Bacteria	Terrabacteria group	Firmicutes	Bacilli	Bacillales	Thermoactinomycetaceae	Thermoactinomyces
2	Bacteria	Terrabacteria group	Firmicutes	Bacilli	Bacillales	Thermoactinomycetaceae	Thermoactinomyces
2	Bacteria	Terrabacteria group	Firmicutes	Bacilli	Bacillales	Thermoactinomycetaceae	Shimazuella
2	Bacteria	Terrabacteria group	Firmicutes	Bacilli	Bacillales	Staphylococcaceae	unclassified Staphylococcaceae
2	Bacteria	Terrabacteria group	Firmicutes	Bacilli	Bacillales	Staphylococcaceae	Staphylococcus
2	Bacteria	Terrabacteria group	Firmicutes	Bacilli	Bacillales	Staphylococcaceae	Salinicoccus
2	Bacteria	Terrabacteria group	Firmicutes	Bacilli	Bacillales	Staphylococcaceae	Salinicoccus
2	Bacteria	Terrabacteria group	Firmicutes	Bacilli	Bacillales	Sporolactobacillaceae	Sporolactobacillus
2	Bacteria	Terrabacteria group	Firmicutes	Bacilli	Bacillales	Sporolactobacillaceae	Sporolactobacillus
2	Bacteria	Terrabacteria group	Firmicutes	Bacilli	Bacillales	Planococcaceae	Sporosarcina
2	Bacteria	Terrabacteria group	Firmicutes	Bacilli	Bacillales	Planococcaceae	Solibacillus
2	Bacteria	Terrabacteria group	Firmicutes	Bacilli	Bacillales	Planococcaceae	Planococcus
2	Bacteria	Terrabacteria group	Firmicutes	Bacilli	Bacillales	Planococcaceae	Kurthia
2	Bacteria	Terrabacteria group	Firmicutes	Bacilli	Bacillales	Planococcaceae	Jeotgalibacillus
2	Bacteria	Terrabacteria group	Firmicutes	Bacilli	Bacillales	Paenibacillaceae	unclassified Paenibacillaceae
2	Bacteria	Terrabacteria group	Firmicutes	Bacilli	Bacillales	Paenibacillaceae	Thermobacillus
2	Bacteria	Terrabacteria group	Firmicutes	Bacilli	Bacillales	Paenibacillaceae	Paenibacillus
2	Bacteria	Terrabacteria group	Firmicutes	Bacilli	Bacillales	Paenibacillaceae	Paenibacillus
2	Bacteria	Terrabacteria group	Firmicutes	Bacilli	Bacillales	Paenibacillaceae	Paenibacillus
2	Bacteria	Terrabacteria group	Firmicutes	Bacilli	Bacillales	Paenibacillaceae	Paenibacillus
2	Bacteria	Terrabacteria group	Firmicutes	Bacilli	Bacillales	Paenibacillaceae	Paenibacillus
2	Bacteria	Terrabacteria group	Firmicutes	Bacilli	Bacillales	Paenibacillaceae	Paenibacillus
2	Bacteria	Terrabacteria group	Firmicutes	Bacilli	Bacillales	Paenibacillaceae	Paenibacillus
2	Bacteria	Terrabacteria group	Firmicutes	Bacilli	Bacillales	Paenibacillaceae	Paenibacillus
2	Bacteria	Terrabacteria group	Firmicutes	Bacilli	Bacillales	Paenibacillaceae	Paenibacillus
2	Bacteria	Terrabacteria group	Firmicutes	Bacilli	Bacillales	Paenibacillaceae	Paenibacillus
2	Bacteria	Terrabacteria group	Firmicutes	Bacilli	Bacillales	Paenibacillaceae	Paenibacillus
2	Bacteria	Terrabacteria group	Firmicutes	Bacilli	Bacillales	Paenibacillaceae	Paenibacillus
2	Bacteria	Terrabacteria group	Firmicutes	Bacilli	Bacillales	Paenibacillaceae	Paenibacillus
2	Bacteria	Terrabacteria group	Firmicutes	Bacilli	Bacillales	Paenibacillaceae	Paenibacillus
2	Bacteria	Terrabacteria group	Firmicutes	Bacilli	Bacillales	Paenibacillaceae	Paenibacillus
2	Bacteria	Terrabacteria group	Firmicutes	Bacilli	Bacillales	Paenibacillaceae	Paenibacillus
[illegible]	[illegible]	[illegible]	[illegible]	[illegible]	[illegible]	[illegible]	[illegible]

2	Bacteria	Synergistetes	Synergistia	Synergistales	unclassified Synergistales	Synergistales bacterium 54_9	
2	Bacteria	Synergistetes	Synergistia	Synergistales	unclassified Synergistales	Synergistales bacterium 53_16	
2	Bacteria	Synergistetes	Synergistia	Synergistales	Synergistaceae	Pyramidobacter	Pyramidobacter sp. C12-8
2	Bacteria	Synergistetes	Synergistia	Synergistales	Synergistaceae	Cloacibacillus	Cloacibacillus evryensis
2	Bacteria	Synergistetes	Synergistia	Synergistales	Synergistaceae	Acetomicrobium	Acetomicrobium thermoterrenum
2	Bacteria	Synergistetes	Synergistia	Synergistales	Synergistaceae	Acetomicrobium	Acetomicrobium flavidum
2	Bacteria	Spirochaetes	Spirochaetia	Spirochaetales	Spirochaetaceae	unclassified Spirochaetaceae	Spirochaetaceae bacterium 4572_7
2	Bacteria	Spirochaetes	Spirochaetia	Spirochaetales	Spirochaetaceae	Treponema	Treponema succinifaciens
2	Bacteria	Spirochaetes	Spirochaetia	Spirochaetales	Spirochaetaceae	Treponema	Treponema sp. CETP13
2	Bacteria	Spirochaetes	Spirochaetia	Spirochaetales	Spirochaetaceae	Treponema	Treponema putidum
2	Bacteria	Spirochaetes	Spirochaetia	Spirochaetales	Spirochaetaceae	Treponema	Treponema phagedenis
2	Bacteria	Spirochaetes	Spirochaetia	Spirochaetales	Spirochaetaceae	Treponema	Treponema medium
2	Bacteria	Spirochaetes	Spirochaetia	Spirochaetales	Spirochaetaceae	Treponema	Treponema denticola
2	Bacteria	Spirochaetes	Spirochaetia	Spirochaetales	Spirochaetaceae	Treponema	Treponema caldarium
2	Bacteria	Spirochaetes	Spirochaetia	Spirochaetales	Spirochaetaceae	Treponema	Treponema bryantii
2	Bacteria	Spirochaetes	Spirochaetia	Spirochaetales	Spirochaetaceae	Spirochaeta	Spirochaeta americana
2	Bacteria	Spirochaetes	Spirochaetia	Spirochaetales	Borreliaceae	Borreliella	
2	Bacteria	Spirochaetes	Spirochaetia	Spirochaetales	Borreliaceae	Borrelia	
2	Bacteria	Spirochaetes	Spirochaetia	Leptospirales	Leptospiraceae	Leptospira	Leptospira weilii
2	Bacteria	Spirochaetes	Spirochaetia	Leptospirales	Leptospiraceae	Leptospira	Leptospira santarosai
2	Bacteria	Spirochaetes	Spirochaetia	Leptospirales	Leptospiraceae	Leptospira	Leptospira licerasiae
2	Bacteria	Spirochaetes	Spirochaetia	Leptospirales	Leptospiraceae	Leptospira	Leptospira kmetyi
2	Bacteria	Spirochaetes	Spirochaetia	Leptospirales	Leptospiraceae	Leptospira	Leptospira interrogans
2	Bacteria	Spirochaetes	Spirochaetia	Leptospirales	Leptospiraceae	Leptospira	Leptospira alexanderi
2	Bacteria	Spirochaetes	Spirochaetia	Brevinematales	Brevinemataceae	Brevinema	Brevinema andersonii
2	Bacteria	Spirochaetes	Spirochaetia	Brachyspirales	Brachyspiraceae	Brachyspira	Brachyspira hyodysenteriae
2	Bacteria	Spirochaetes					
2	Bacteria	Proteobacteria	unclassified Proteobacteria	unclassified Proteobacteria (miscellaneous)	Proteobacteria bacterium ST_bin11		
2	Bacteria	Proteobacteria	delta/epsilon subdivisions	Epsilonproteobacteria	unclassified Epsilonproteobacteria	unclassified Epsilonproteobacteria (miscellaneous)	Epsilonproteobacteria bacterium LFT 1.7
2	Bacteria	Proteobacteria	delta/epsilon subdivisions	Epsilonproteobacteria	unclassified Epsilonproteobacteria	Sulfurovum	environmental samples
2	Bacteria	Proteobacteria	delta/epsilon subdivisions	Epsilonproteobacteria	unclassified Epsilonproteobacteria	Sulfurovum	Sulfurovum sp. AS07-7
2	Bacteria	Proteobacteria	delta/epsilon subdivisions	Epsilonproteobacteria	unclassified Epsilonproteobacteria	Sulfurovum	Sulfurovum sp. AR
2	Bacteria	Proteobacteria	delta/epsilon subdivisions	Epsilonproteobacteria	Campylobacterales	unclassified Campylobacterales	Nitratifractor
2	Bacteria	Proteobacteria	delta/epsilon subdivisions	Epsilonproteobacteria	Campylobacterales	Helicobacteraceae	Wolinella
2	Bacteria	Proteobacteria	delta/epsilon subdivisions	Epsilonproteobacteria	Campylobacterales	Helicobacteraceae	Helicobacter
2	Bacteria	Proteobacteria	delta/epsilon subdivisions	Epsilonproteobacteria	Campylobacterales	Helicobacteraceae	Helicobacter
2	Bacteria	Proteobacteria	delta/epsilon subdivisions	Epsilonproteobacteria	Campylobacterales	Campylobacteraceae	Sulfurospirillum
2	Bacteria	Proteobacteria	delta/epsilon subdivisions	Epsilonproteobacteria	Campylobacterales	Campylobacteraceae	Sulfurospirillum
2	Bacteria	Proteobacteria	delta/epsilon subdivisions	Epsilonproteobacteria	Campylobacterales	Campylobacteraceae	Campylobacter
2	Bacteria	Proteobacteria	delta/epsilon subdivisions	Epsilonproteobacteria	Campylobacterales	Campylobacteraceae	Campylobacter
2	Bacteria	Proteobacteria	delta/epsilon subdivisions	Epsilonproteobacteria	Campylobacterales	Campylobacteraceae	Campylobacter
2	Bacteria	Proteobacteria	delta/epsilon subdivisions	Epsilonproteobacteria	Campylobacterales	Campylobacteraceae	Campylobacter
2	Bacteria	Proteobacteria	delta/epsilon subdivisions	Epsilonproteobacteria	Campylobacterales	Campylobacteraceae	Campylobacter
2	Bacteria	Proteobacteria	delta/epsilon subdivisions	Epsilonproteobacteria	Campylobacterales	Campylobacteraceae	Campylobacter
2	Bacteria	Proteobacteria	delta/epsilon subdivisions	Epsilonproteobacteria	Campylobacterales	Campylobacteraceae	Campylobacter
2	Bacteria	Proteobacteria	delta/epsilon subdivisions	Epsilonproteobacteria	Campylobacterales	Campylobacteraceae	Campylobacter
2	Bacteria	Proteobacteria	delta/epsilon subdivisions	Epsilonproteobacteria	Campylobacterales	Campylobacteraceae	Arcobacter
2	Bacteria	Proteobacteria	delta/epsilon subdivisions	Epsilonproteobacteria	Campylobacterales	Campylobacteraceae	Arcobacter
2	Bacteria	Proteobacteria	delta/epsilon subdivisions	Epsilonproteobacteria	Campylobacterales	Campylobacteraceae	Arcobacter
2	Bacteria	Proteobacteria	delta/epsilon subdivisions	Epsilonproteobacteria	Campylobacterales		
2	Bacteria	Proteobacteria	delta/epsilon subdivisions	Deltaproteobacteria	unclassified Deltaproteobacteria	unclassified Deltaproteobacteria (miscellaneous)	Deltaproteobacteria bacterium RIFOXYB12_FULL_58_9
2	Bacteria	Proteobacteria	delta/epsilon subdivisions	Deltaproteobacteria	unclassified Deltaproteobacteria	unclassified Deltaproteobacteria (miscellaneous)	Deltaproteobacteria bacterium RIFCSPLOWO2_12_55_13
2	Bacteria	Proteobacteria	delta/epsilon subdivisions	Deltaproteobacteria	unclassified Deltaproteobacteria	unclassified Deltaproteobacteria (miscellaneous)	Deltaproteobacteria bacterium RIFCSPLOWO2_02_FULL_47_10
2	Bacteria	Proteobacteria	delta/epsilon subdivisions	Deltaproteobacteria	unclassified Deltaproteobacteria	unclassified Deltaproteobacteria (miscellaneous)	Deltaproteobacteria bacterium RIFCSPLOWO2_02_FULL_46_8
2	Bacteria	Proteobacteria	delta/epsilon subdivisions	Deltaproteobacteria	unclassified Deltaproteobacteria	unclassified Deltaproteobacteria (miscellaneous)	Deltaproteobacteria bacterium RIFCSPLOWO2_02_56_12
2	Bacteria	Proteobacteria	delta/epsilon subdivisions	Deltaproteobacteria	unclassified Deltaproteobacteria	unclassified Deltaproteobacteria (miscellaneous)	Deltaproteobacteria bacterium RIFCSPHIGHO2_02_FULL_40_11
2	Bacteria	Proteobacteria	delta/epsilon subdivisions	Deltaproteobacteria	unclassified Deltaproteobacteria	unclassified Deltaproteobacteria (miscellaneous)	Deltaproteobacteria bacterium RBG_19FT_COMBO_46_9
2	Bacteria	Proteobacteria	delta/epsilon subdivisions	Deltaproteobacteria	unclassified Deltaproteobacteria	unclassified Deltaproteobacteria (miscellaneous)	Deltaproteobacteria bacterium RBG_16_64_85
2	Bacteria	Proteobacteria	delta/epsilon subdivisions	Deltaproteobacteria	unclassified Deltaproteobacteria	unclassified Deltaproteobacteria (miscellaneous)	Deltaproteobacteria bacterium RBG_16_54_18
2	Bacteria	Proteobacteria	delta/epsilon subdivisions	Deltaproteobacteria	unclassified Deltaproteobacteria	unclassified Deltaproteobacteria (miscellaneous)	Deltaproteobacteria bacterium RBG_16_50_11
2	Bacteria	Proteobacteria	delta/epsilon subdivisions	Deltaproteobacteria	unclassified Deltaproteobacteria	unclassified Deltaproteobacteria (miscellaneous)	Deltaproteobacteria bacterium RBG_16_42_7
2	Bacteria	Proteobacteria	delta/epsilon subdivisions	Deltaproteobacteria	unclassified Deltaproteobacteria	unclassified Deltaproteobacteria (miscellaneous)	Deltaproteobacteria bacterium RBG_13_52_11
2	Bacteria	Proteobacteria	delta/epsilon subdivisions	Deltaproteobacteria	unclassified Deltaproteobacteria	unclassified Deltaproteobacteria (miscellaneous)	Deltaproteobacteria bacterium GWA2_47_9
2	Bacteria	Proteobacteria	delta/epsilon subdivisions	Deltaproteobacteria	unclassified Deltaproteobacteria	unclassified Deltaproteobacteria (miscellaneous)	Deltaproteobacteria bacterium CG2_30_66_27
2	Bacteria	Proteobacteria	delta/epsilon subdivisions	Deltaproteobacteria	unclassified Deltaproteobacteria	unclassified Deltaproteobacteria (miscellaneous)	Deltaproteobacteria bacterium CG2_30_43_15
2	Bacteria	Proteobacteria	delta/epsilon subdivisions	Deltaproteobacteria	unclassified Deltaproteobacteria	unclassified Deltaproteobacteria (miscellaneous)	Deltaproteobacteria bacterium 13_1_40CM_4_54_4
2	Bacteria	Proteobacteria	delta/epsilon subdivisions	Deltaproteobacteria	unclassified Deltaproteobacteria	Candidatus Babela	Candidatus Babela massiliensis
2	Bacteria	Proteobacteria	delta/epsilon subdivisions	Deltaproteobacteria	Syntrophobacterales	unclassified Syntrophobacterales	Syntrophobacterales bacterium GWC2_56_13
2	Bacteria	Proteobacteria	delta/epsilon subdivisions	Deltaproteobacteria	Syntrophobacterales	Syntrophaceae	Syntrophus
2	Bacteria	Proteobacteria	delta/epsilon subdivisions	Deltaproteobacteria	Myxococcales	Nannocystineae	Kofleriaceae
2	Bacteria	Proteobacteria	delta/epsilon subdivisions	Deltaproteobacteria	Myxococcales	Cystobacterineae	Myxococcaceae
2	Bacteria	Proteobacteria	delta/epsilon subdivisions	Deltaproteobacteria	Myxococcales	Cystobacterineae	Myxococcaceae
2	Bacteria	Proteobacteria	delta/epsilon subdivisions	Deltaproteobacteria	Myxococcales	Cystobacterineae	Archangiaceae
2	Bacteria	Proteobacteria	delta/epsilon subdivisions	Deltaproteobacteria	Myxococcales	Cystobacterineae	Anaeromyxobacteraceae
2	Bacteria	Proteobacteria	delta/epsilon subdivisions	Deltaproteobacteria	Desulfuromonadales	unclassified Desulfuromonadales	Desulfuromonadales bacterium GWD2_61_12
2	Bacteria	Proteobacteria	delta/epsilon subdivisions	Deltaproteobacteria	Desulfuromonadales	Geobacteraceae	unclassified Geobacteraceae
2	Bacteria	Proteobacteria	delta/epsilon subdivisions	Deltaproteobacteria	Desulfuromonadales	Geobacteraceae	Geobacter
2	Bacteria	Proteobacteria	delta/epsilon subdivisions	Deltaproteobacteria	Desulfuromonadales	Desulfuromonadaceae	Pelobacter
2	Bacteria	Proteobacteria	delta/epsilon subdivisions	Deltaproteobacteria	Desulfurellales	Desulfurellaceae	Hippea
2	Bacteria	Proteobacteria	delta/epsilon subdivisions	Deltaproteobacteria	Desulfurellales	Desulfurellaceae	Hippea
2	Bacteria	Proteobacteria	delta/epsilon subdivisions	Deltaproteobacteria	Desulfovibrionales	Desulfovibrionaceae	Desulfovibrio
2	Bacteria	Proteobacteria	delta/epsilon subdivisions	Deltaproteobacteria	Desulfovibrionales	Desulfovibrionaceae	Desulfovibrio
2	Bacteria	Proteobacteria	delta/epsilon subdivisions	Deltaproteobacteria	Desulfovibrionales	Desulfovibrionaceae	Desulfovibrio
2	Bacteria	Proteobacteria	delta/epsilon subdivisions	Deltaproteobacteria	Desulfovibrionales	Desulfomicrobiaceae	Desulfomicrobium
2	Bacteria	Proteobacteria	delta/epsilon subdivisions	Deltaproteobacteria	Desulfovibrionales	Desulfomicrobiaceae	Desulfomicrobium
2	Bacteria	Proteobacteria	delta/epsilon subdivisions	Deltaproteobacteria	Desulfovibrionales	Desulfohalobiaceae	
2	Bacteria	Proteobacteria	delta/epsilon subdivisions	Deltaproteobacteria	Desulfobacterales	unclassified Desulfobacterales	Desulfobacterales bacterium S7086C20
2	Bacteria	Proteobacteria	delta/epsilon subdivisions	Deltaproteobacteria	Desulfobacterales	unclassified Desulfobacterales	Desulfobacterales bacterium S5133MH4
2	Bacteria	Proteobacteria	delta/epsilon subdivisions	Deltaproteobacteria	Desulfobacterales	unclassified Desulfobacterales	Desulfobacterales bacterium PC51MH44
2	Bacteria	Proteobacteria	delta/epsilon subdivisions	Deltaproteobacteria	Desulfobacterales	Desulfobulbaceae	Desulfotalea
2	Bacteria	Proteobacteria	delta/epsilon subdivisions	Deltaproteobacteria	Desulfobacterales	Desulfobacteraceae	unclassified Desulfobacteraceae
2	Bacteria	Proteobacteria	delta/epsilon subdivisions	Deltaproteobacteria	Desulfobacterales	Desulfobacteraceae	unclassified Desulfobacteraceae
2	Bacteria	Proteobacteria	delta/epsilon subdivisions	Deltaproteobacteria	Desulfobacterales	Desulfobacteraceae	unclassified Desulfobacteraceae
2	Bacteria	Proteobacteria	delta/epsilon subdivisions	Deltaproteobacteria	Desulfobacterales	Desulfobacteraceae	Desulfotignum
2	Bacteria	Proteobacteria	delta/epsilon subdivisions	Deltaproteobacteria	Desulfobacterales	Desulfobacteraceae	Desulfococcus
2	Bacteria	Proteobacteria	delta/epsilon subdivisions	Deltaproteobacteria	Bdellovibrionales	unclassified Bdellovibrionales	Bdellovibrionales bacterium RIFOXYD1_FULL_44_7
2	Bacteria	Proteobacteria	delta/epsilon subdivisions	Deltaproteobacteria	Bdellovibrionales	unclassified Bdellovibrionales	Bdellovibrionales bacterium RBG_16_40_8
2	Bacteria	Proteobacteria	Zetaproteobacteria	unclassified Zetaproteobacteria	Zetaproteobacteria bacterium CG1_02_53_45		
2	Bacteria	Proteobacteria	Gammaproteobacteria	unclassified Gammaproteobacteria	unclassified Gammaproteobacteria (miscellaneous)	gamma proteobacterium HTCC5015	
2	Bacteria	Proteobacteria	Gammaproteobacteria	unclassified Gammaproteobacteria	unclassified Gammaproteobacteria (miscellaneous)	Gammaproteobacteria bacterium SG8_15	
2	Bacteria	Proteobacteria	Gammaproteobacteria	unclassified Gammaproteobacteria	unclassified Gammaproteobacteria (miscellaneous)	Gammaproteobacteria bacterium RIFCSPHIGHO2_12_FULL_40_19	
2	Bacteria	Proteobacteria	Gammaproteobacteria	unclassified Gammaproteobacteria	unclassified Gammaproteobacteria (miscellaneous)	Gammaproteobacteria bacterium RIFCSPHIGHO2_12_FULL_35_23	
2	Bacteria	Proteobacteria	Gammaproteobacteria	unclassified Gammaproteobacteria	unclassified Gammaproteobacteria (miscellaneous)	Gammaproteobacteria bacterium RBG_16_66_13	
2	Bacteria	Proteobacteria	Gammaproteobacteria	unclassified Gammaproteobacteria	unclassified Gammaproteobacteria (miscellaneous)	Gammaproteobacteria bacterium RBG_16_37_9	
2	Bacteria	Proteobacteria	Gammaproteobacteria	unclassified Gammaproteobacteria	Sedimenticola		
2	Bacteria	Proteobacteria	Gammaproteobacteria	unclassified Gammaproteobacteria	SAR86 cluster	SAR86 cluster bacterium SAR86E	
2	Bacteria	Proteobacteria	Gammaproteobacteria	unclassified Gammaproteobacteria	SAR86 cluster	SAR86 cluster bacterium SAR86B	
2	Bacteria	Proteobacteria	Gammaproteobacteria	unclassified Gammaproteobacteria	Gallaecimonas	Gallaecimonas xiamenensis	
2	Bacteria	Proteobacteria	Gammaproteobacteria	unclassified Gammaproteobacteria	Competibacteraceae		
2	Bacteria	Proteobacteria	Gammaproteobacteria	Xanthomonadales	unclassified Xanthomonadales	Xanthomonadales bacterium RIFOXYD1_FULL_69_11	
2	Bacteria	Proteobacteria	Gammaproteobacteria	Xanthomonadales	unclassified Xanthomonadales	Xanthomonadales bacterium RIFOXYA1_FULL_68_6	
2	Bacteria	Proteobacteria	Gammaproteobacteria	Xanthomonadales	Xanthomonadaceae	Xanthomonas	Xanthomonas sp. SHU308
2	Bacteria	Proteobacteria	Gammaproteobacteria	Xanthomonadales	Xanthomonadaceae	Xanthomonas	Xanthomonas fragariae
2	Bacteria	Proteobacteria	Gammaproteobacteria	Xanthomonadales	Xanthomonadaceae	Xanthomonas	Xanthomonas cannabis
2	Bacteria	Proteobacteria	Gammaproteobacteria	Xanthomonadales	Xanthomonadaceae	Stenotrophomonas	Stenotrophomonas sp. HMSC10F06
2	Bacteria	Proteobacteria	Gammaproteobacteria	Xanthomonadales	Xanthomonadaceae	Lysobacter	Lysobacter sp. cf310
2	Bacteria	Proteobacteria	Gammaproteobacteria	Xanthomonadales	Xanthomonadaceae	Lysobacter	Lysobacter sp. Root604
2	Bacteria	Proteobacteria	Gammaproteobacteria	Xanthomonadales	Rhodanobacteraceae	Rhodanobacter	Rhodanobacter spathiphylli
2	Bacteria	Proteobacteria	Gammaproteobacteria	Xanthomonadales	Rhodanobacteraceae	Rhodanobacter	Rhodanobacter sp. Soil772
2	Bacteria	Proteobacteria	Gammaproteobacteria	Xanthomonadales	Rhodanobacteraceae	Rhodanobacter	Rhodanobacter sp. SCN 67-45
2	Bacteria	Proteobacteria	Gammaproteobacteria	Xanthomonadales	Rhodanobacteraceae	Rhodanobacter	Rhodanobacter sp. Root627
2	Bacteria	Proteobacteria	Gammaproteobacteria	Xanthomonadales	Rhodanobacteraceae	Rhodanobacter	Rhodanobacter sp. OR87
2	Bacteria	Proteobacteria	Gammaproteobacteria	Xanthomonadales	Rhodanobacteraceae	Rhodanobacter	Rhodanobacter fulvus
2	Bacteria	Proteobacteria	Gammaproteobacteria	Xanthomonadales	Rhodanobacteraceae	Luteibacter	Luteibacter sp. UNCMF331Sha3.1
2	Bacteria	Proteobacteria	Gammaproteobacteria	Xanthomonadales	Rhodanobacteraceae	Luteibacter	Luteibacter rhizovicinus
2	Bacteria	Proteobacteria	Gammaproteobacteria	Xanthomonadales	Rhodanobacteraceae	Dyella	Dyella ginsengisoli
2	Bacteria	Proteobacteria	Gammaproteobacteria	Vibrionales	Vibrionaceae	Vibrio	Vibrio tasmaniensis
2	Bacteria	Proteobacteria	Gammaproteobacteria	Vibrionales	Vibrionaceae	Vibrio	Vibrio sp. C7
2	Bacteria	Proteobacteria	Gammaproteobacteria	Vibrionales	Vibrionaceae	Vibrio	Vibrio ruber
2	Bacteria	Proteobacteria	Gammaproteobacteria	Vibrionales	Vibrionaceae	Vibrio	Vibrio oreintalis group
2	Bacteria	Proteobacteria	Gammaproteobacteria	Vibrionales	Vibrionaceae	Vibrio	Vibrio mediterranei
2	Bacteria	Proteobacteria	Gammaproteobacteria	Vibrionales	Vibrionaceae	Vibrio	Vibrio maritimus
2	Bacteria	Proteobacteria	Gammaproteobacteria	Vibrionales	Vibrionaceae	Vibrio	Vibrio genomosp. F6
2	Bacteria	Proteobacteria	Gammaproteobacteria	Vibrionales	Vibrionaceae	Vibrio	Vibrio barjaei
2	Bacteria	Proteobacteria	Gammaproteobacteria	Vibrionales	Vibrionaceae	Vibrio	Vibrio aerogenes
2	Bacteria	Proteobacteria	Gammaproteobacteria	Vibrionales	Vibrionaceae	Photobacterium	Photobacterium sp. SKA34
2	Bacteria	Proteobacteria	Gammaproteobacteria	Vibrionales	Vibrionaceae	Photobacterium	Photobacterium sanctipauli
2	Bacteria	Proteobacteria	Gammaproteobacteria	Vibrionales	Vibrionaceae	Photobacterium	Photobacterium galatheae
2	Bacteria	Proteobacteria	Gammaproteobacteria	Vibrionales	Vibrionaceae	Photobacterium	Photobacterium gaetbulicola
2	Bacteria	Proteobacteria	Gammaproteobacteria	Vibrionales	Vibrionaceae	Photobacterium	Photobacterium damselae
2	Bacteria	Proteobacteria	Gammaproteobacteria	Vibrionales	Vibrionaceae	Grimontia	Grimontia marina
2	Bacteria	Proteobacteria	Gammaproteobacteria	Vibrionales	Vibrionaceae	Enterovibrio	Enterovibrio coralii
2	Bacteria	Proteobacteria	Gammaproteobacteria	Vibrionales	Vibrionaceae	Enterovibrio	Enterovibrio calviensis
2	Bacteria	Proteobacteria	Gammaproteobacteria	Vibrionales	Vibrionaceae	Candidatus Photodesmus	Candidatus Photodesmus katoptron
2	Bacteria	Proteobacteria	Gammaproteobacteria	Vibrionales	Vibrionaceae	Aliivibrio	Aliivibrio wodanis
2	Bacteria	Proteobacteria	Gammaproteobacteria	Thiotrichales	unclassified Thiotrichales	Fangia	Fangia hongkongensis
2	Bacteria	Proteobacteria	Gammaproteobacteria	Thiotrichales	Thiotrichaceae	Achromatium	Achromatium sp. WMS3
2	Bacteria	Proteobacteria	Gammaproteobacteria	Thiotrichales	Thiotrichaceae	Achromatium	Achromatium sp. WMS1
2	Bacteria	Proteobacteria	Gammaproteobacteria	Thiotrichales	Piscirickettsiaceae	Thiomicrospira	Thiomicrospira sp. Milos-T1
2	Bacteria	Proteobacteria	Gammaproteobacteria	Thiotrichales	Piscirickettsiaceae	Thiomicrospira	Thiomicrospira sp. CG2_30_44_34
2	Bacteria	Proteobacteria	Gammaproteobacteria	Thiotrichales	Piscirickettsiaceae	Thiomicrospira	Thiomicrospira pelophila
2	Bacteria	Proteobacteria	Gammaproteobacteria	Thiotrichales	Piscirickettsiaceae	Thioalkalimicrobium	
2	Bacteria	Proteobacteria	Gammaproteobacteria	Thiotrichales	Piscirickettsiaceae	Methylophaga	Methylophaga thiooxydans
2	Bacteria	Proteobacteria	Gammaproteobacteria	Thiotrichales	Piscirickettsiaceae		
2	Bacteria	Proteobacteria	Gammaproteobacteria	Thiotrichales	Francisellaceae	Francisella	Francisella philomiragia
2	Bacteria	Proteobacteria	Gammaproteobacteria	Pseudomonadales	Pseudomonadaceae	Pseudomonas	Pseudomonas xinjiangensis
2	Bacteria	Proteobacteria	Gammaproteobacteria	Pseudomonadales	Pseudomonadaceae	Pseudomonas	Pseudomonas vranovensis
2	Bacteria	Proteobacteria	Gammaproteobacteria	Pseudomonadales	Pseudomonadaceae	Pseudomonas	Pseudomonas syringae group
2	Bacteria	Proteobacteria	Gammaproteobacteria	Pseudomonadales	Pseudomonadaceae	Pseudomonas	Pseudomonas syringae group
2	Bacteria	Proteobacteria	Gammaproteobacteria	Pseudomonadales	Pseudomonadaceae	Pseudomonas	Pseudomonas sp. ok266
2	Bacteria	Proteobacteria	Gammaproteobacteria	Pseudomonadales	Pseudomonadaceae	Pseudomonas	Pseudomonas sp. W15Feb9B
2	Bacteria	Proteobacteria	Gammaproteobacteria	Pseudomonadales	Pseudomonadaceae	Pseudomonas	Pseudomonas sp. URMO17WK12:I12
2	Bacteria	Proteobacteria	Gammaproteobacteria	Pseudomonadales	Pseudomonadaceae	Pseudomonas	Pseudomonas sp. URHB0015
2	Bacteria	Proteobacteria	Gammaproteobacteria	Pseudomonadales	Pseudomonadaceae	Pseudomonas	Pseudomonas sp. T
2	Bacteria	Proteobacteria	Gammaproteobacteria	Pseudomonadales	Pseudomonadaceae	Pseudomonas	Pseudomonas sp. StFLB209
2	Bacteria	Proteobacteria	Gammaproteobacteria	Pseudomonadales	Pseudomonadaceae	Pseudomonas	Pseudomonas sp. S9
2	Bacteria	Proteobacteria	Gammaproteobacteria	Pseudomonadales	Pseudomonadaceae	Pseudomonas	Pseudomonas sp. Root401
2	Bacteria	Proteobacteria	Gammaproteobacteria	Pseudomonadales	Pseudomonadaceae	Pseudomonas	Pseudomonas sp. RIT288
2	Bacteria	Proteobacteria	Gammaproteobacteria	Pseudomonadales	Pseudomonadaceae	Pseudomonas	Pseudomonas sp. RIT-PI-q
2	Bacteria	Proteobacteria	Gammaproteobacteria	Pseudomonadales	Pseudomonadaceae	Pseudomonas	Pseudomonas sp. PH1b
2	Bacteria	Proteobacteria	Gammaproteobacteria	Pseudomonadales	Pseudomonadaceae	Pseudomonas	Pseudomonas sp. P818
2	Bacteria	Proteobacteria	Gammaproteobacteria	Pseudomonadales	Pseudomonadaceae	Pseudomonas	Pseudomonas sp. NFACC13-1
2	Bacteria	Proteobacteria	Gammaproteobacteria	Pseudomonadales	Pseudomonadaceae	Pseudomonas	Pseudomonas sp. NBRC 111131
2	Bacteria	Proteobacteria	Gammaproteobacteria	Pseudomonadales	Pseudomonadaceae	Pseudomonas	Pseudomonas sp. Leaf58
2	Bacteria	Proteobacteria	Gammaproteobacteria	Pseudomonadales	Pseudomonadaceae	Pseudomonas	Pseudomonas sp. Leaf48
2	Bacteria	Proteobacteria	Gammaproteobacteria	Pseudomonadales	Pseudomonadaceae	Pseudomonas	Pseudomonas sp. LAB-08
2	Bacteria	Proteobacteria	Gammaproteobacteria	Pseudomonadales	Pseudomonadaceae	Pseudomonas	Pseudomonas sp. GM80
2	Bacteria	Proteobacteria	Gammaproteobacteria	Pseudomonadales	Pseudomonadaceae	Pseudomonas	Pseudomonas sp. GM102
2	Bacteria	Proteobacteria	Gammaproteobacteria	Pseudomonadales	Pseudomonadaceae	Pseudomonas	Pseudomonas sp. GEV388
2	Bacteria	Proteobacteria	Gammaproteobacteria	Pseudomonadales	Pseudomonadaceae	Pseudomonas	Pseudomonas sp. ENNP23
2	Bacteria	Proteobacteria	Gammaproteobacteria	Pseudomonadales	Pseudomonadaceae	Pseudomonas	Pseudomonas sp. CF161
2	Bacteria	Proteobacteria	Gammaproteobacteria	Pseudomonadales	Pseudomonadaceae	Pseudomonas	Pseudomonas sp. 250J
2	Bacteria	Proteobacteria	Gammaproteobacteria	Pseudomonadales	Pseudomonadaceae	Pseudomonas	Pseudomonas sp. 21
2	Bacteria	Proteobacteria	Gammaproteobacteria	Pseudomonadales	Pseudomonadaceae	Pseudomonas	Pseudomonas sp. 10-1B
2	Bacteria	Proteobacteria	Gammaproteobacteria	Pseudomonadales	Pseudomonadaceae	Pseudomonas	Pseudomonas saudiphocaensis
2	Bacteria	Proteobacteria	Gammaproteobacteria	Pseudomonadales	Pseudomonadaceae	Pseudomonas	Pseudomonas saudimassiliensis
2	Bacteria	Proteobacteria	Gammaproteobacteria	Pseudomonadales	Pseudomonadaceae	Pseudomonas	Pseudomonas salomonii
2	Bacteria	Proteobacteria	Gammaproteobacteria	Pseudomonadales	Pseudomonadaceae	Pseudomonas	Pseudomonas reinekei
2	Bacteria	Proteobacteria	Gammaproteobacteria	Pseudomonadales	Pseudomonadaceae	Pseudomonas	Pseudomonas putida group
2	Bacteria	Proteobacteria	Gammaproteobacteria	Pseudomonadales	Pseudomonadaceae	Pseudomonas	Pseudomonas putida group
2	Bacteria	Proteobacteria	Gammaproteobacteria	Pseudomonadales	Pseudomonadaceae	Pseudomonas	Pseudomonas psychrophila
2	Bacteria	Proteobacteria	Gammaproteobacteria	Pseudomonadales	Pseudomonadaceae	Pseudomonas	Pseudomonas litoralis
2	Bacteria	Proteobacteria	Gammaproteobacteria	Pseudomonadales	Pseudomonadaceae	Pseudomonas	Pseudomonas kuykendallii
2	Bacteria	Proteobacteria	Gammaproteobacteria	Pseudomonadales	Pseudomonadaceae	Pseudomonas	Pseudomonas koreensis
2	Bacteria	Proteobacteria	Gammaproteobacteria	Pseudomonadales	Pseudomonadaceae	Pseudomonas	Pseudomonas fluorescens group
2	Bacteria	Proteobacteria	Gammaproteobacteria	Pseudomonadales	Pseudomonadaceae	Pseudomonas	Pseudomonas endophytica
2	Bacteria	Proteobacteria	Gammaproteobacteria	Pseudomonadales	Pseudomonadaceae	Pseudomonas	Pseudomonas deceptionensis
2	Bacteria	Proteobacteria	Gammaproteobacteria	Pseudomonadales	Pseudomonadaceae	Pseudomonas	Pseudomonas composti
2	Bacteria	Proteobacteria	Gammaproteobacteria	Pseudomonadales	Pseudomonadaceae	Pseudomonas	Pseudomonas brassicacearum
2	Bacteria	Proteobacteria	Gammaproteobacteria	Pseudomonadales	Pseudomonadaceae	Pseudomonas	Pseudomonas aeruginosa group
2	Bacteria	Proteobacteria	Gammaproteobacteria	Pseudomonadales	Pseudomonadaceae		
2	Bacteria	Proteobacteria	Gammaproteobacteria	Pseudomonadales	Moraxellaceae	Psychrobacter	Psychrobacter urativorans
2	Bacteria	Proteobacteria	Gammaproteobacteria	Pseudomonadales	Moraxellaceae	Psychrobacter	Psychrobacter sp. JCM 18901
2	Bacteria	Proteobacteria	Gammaproteobacteria	Pseudomonadales	Moraxellaceae	Psychrobacter	Psychrobacter sp. AntiMn-1
2	Bacteria	Proteobacteria	Gammaproteobacteria	Pseudomonadales	Moraxellaceae	Psychrobacter	Psychrobacter lutiphocae
2	Bacteria	Proteobacteria	Gammaproteobacteria	Pseudomonadales	Moraxellaceae	Perlucidibaca	
2	Bacteria	Proteobacteria	Gammaproteobacteria	Pseudomonadales	Moraxellaceae	Acinetobacter	Acinetobacter venetianus
2	Bacteria	Proteobacteria	Gammaproteobacteria	Pseudomonadales	Moraxellaceae	Acinetobacter	Acinetobacter sp. MN12
2	Bacteria	Proteobacteria	Gammaproteobacteria	Pseudomonadales	Moraxellaceae	Acinetobacter	Acinetobacter sp. CAG:196_36_41
2	Bacteria	Proteobacteria	Gammaproteobacteria	Pseudomonadales	Moraxellaceae	Acinetobacter	Acinetobacter rudis
2	Bacteria	Proteobacteria	Gammaproteobacteria	Pseudomonadales	Moraxellaceae	Acinetobacter	Acinetobacter radioresistens
2	Bacteria	Proteobacteria	Gammaproteobacteria	Pseudomonadales	Moraxellaceae	Acinetobacter	Acinetobacter qingfengensis
2	Bacteria	Proteobacteria	Gammaproteobacteria	Pseudomonadales	Moraxellaceae	Acinetobacter	Acinetobacter harbinensis
2	Bacteria	Proteobacteria	Gammaproteobacteria	Pseudomonadales	Moraxellaceae	Acinetobacter	Acinetobacter gerneri
2	Bacteria	Proteobacteria	Gammaproteobacteria	Pasteurellales	Pasteurellaceae	Rodentibacter	Rodentibacter ratti
2	Bacteria	Proteobacteria	Gammaproteobacteria	Pasteurellales	Pasteurellaceae	Rodentibacter	Rodentibacter pneumotropicus
2	Bacteria	Proteobacteria	Gammaproteobacteria	Pasteurellales	Pasteurellaceae	Pasteurella	Pasteurella dagmatis
2	Bacteria	Proteobacteria	Gammaproteobacteria	Pasteurellales	Pasteurellaceae	Mannheimia	
2	Bacteria	Proteobacteria	Gammaproteobacteria	Pasteurellales	Pasteurellaceae	Haemophilus	[Haemophilus] parasuis
2	Bacteria	Proteobacteria	Gammaproteobacteria	Pasteurellales	Pasteurellaceae	Haemophilus	[Haemophilus] parasuis
2	Bacteria	Proteobacteria	Gammaproteobacteria	Pasteurellales	Pasteurellaceae	Haemophilus	Haemophilus pittmaniae
2	Bacteria	Proteobacteria	Gammaproteobacteria	Pasteurellales	Pasteurellaceae	Haemophilus	Haemophilus paracuniculus
2	Bacteria	Proteobacteria	Gammaproteobacteria	Pasteurellales	Pasteurellaceae	Gallibacterium	Gallibacterium genomosp. 3
2	Bacteria	Proteobacteria	Gammaproteobacteria	Pasteurellales	Pasteurellaceae	Chelonobacter	Chelonobacter oris
2	Bacteria	Proteobacteria	Gammaproteobacteria	Pasteurellales	Pasteurellaceae	Aggregatibacter	Aggregatibacter actinomycetemcomitans
2	Bacteria	Proteobacteria	Gammaproteobacteria	Pasteurellales	Pasteurellaceae	Actinobacillus	Actinobacillus succinogenes
2	Bacteria	Proteobacteria	Gammaproteobacteria	Orbales	Orbaceae	Gilliamella	Gilliamella apicola
2	Bacteria	Proteobacteria	Gammaproteobacteria	Orbales	Orbaceae	Candidatus Schmidhempelia	Candidatus Schmidhempelia bombi
2	Bacteria	Proteobacteria	Gammaproteobacteria	Oceanospirillales	Saccharospirillaceae	Reinekea	Reinekea blandensis
2	Bacteria	Proteobacteria	Gammaproteobacteria	Oceanospirillales	Oleiphilaceae	Oleiphilus	Oleiphilus sp. HI0132
2	Bacteria	Proteobacteria	Gammaproteobacteria	Oceanospirillales	Oceanospirillaceae	Oleispira	Oleispira antarctica
2	Bacteria	Proteobacteria	Gammaproteobacteria	Oceanospirillales	Oceanospirillaceae	Oleibacter	Oleibacter marinus
2	Bacteria	Proteobacteria	Gammaproteobacteria	Oceanospirillales	Oceanospirillaceae	Nitrincola	Nitrincola sp. A-D6
2	Bacteria	Proteobacteria	Gammaproteobacteria	Oceanospirillales	Oceanospirillaceae	Nitrincola	
2	Bacteria	Proteobacteria	Gammaproteobacteria	Oceanospirillales	Oceanospirillaceae	Neptuniibacter	Neptuniibacter marinus
2	Bacteria	Proteobacteria	Gammaproteobacteria	Oceanospirillales	Oceanospirillaceae	Marinomonas	Marinomonas sp. TW1
2	Bacteria	Proteobacteria	Gammaproteobacteria	Oceanospirillales	Oceanospirillaceae	Marinomonas	Marinomonas polaris
2	Bacteria	Proteobacteria	Gammaproteobacteria	Oceanospirillales	Oceanospirillaceae	Marinomonas	Marinomonas fungiae
2	Bacteria	Proteobacteria	Gammaproteobacteria	Oceanospirillales	Oceanospirillaceae	Marinomonas	Marinomonas atlantica
2	Bacteria	Proteobacteria	Gammaproteobacteria	Oceanospirillales	Oceanospirillaceae	Marinobacterium	Marinobacterium rhizophilum
2	Bacteria	Proteobacteria	Gammaproteobacteria	Oceanospirillales	Kangiellaceae	Kangiella	Kangiella koreensis
2	Bacteria	Proteobacteria	Gammaproteobacteria	Oceanospirillales	Kangiellaceae	Kangiella	
2	Bacteria	Proteobacteria	Gammaproteobacteria	Oceanospirillales	Halomonadaceae	Zymobacter group	whitefly endosymbionts
2	Bacteria	Proteobacteria	Gammaproteobacteria	Oceanospirillales	Halomonadaceae	Zymobacter group	Zymobacter
2	Bacteria	Proteobacteria	Gammaproteobacteria	Oceanospirillales	Halomonadaceae	Salinicola	Salinicola sp. MIT1003
2	Bacteria	Proteobacteria	Gammaproteobacteria	Oceanospirillales	Halomonadaceae	Kushneria	
2	Bacteria	Proteobacteria	Gammaproteobacteria	Oceanospirillales	Halomonadaceae	Halomonas	Halomonas zhanjiangensis
2	Bacteria	Proteobacteria	Gammaproteobacteria	Oceanospirillales	Halomonadaceae	Halomonas	Halomonas titanicae
2	Bacteria	Proteobacteria	Gammaproteobacteria	Oceanospirillales	Halomonadaceae	Halomonas	Halomonas sp. QHL1
2	Bacteria	Proteobacteria	Gammaproteobacteria	Oceanospirillales	Halomonadaceae	Halomonas	Halomonas sp. HL-93
2	Bacteria	Proteobacteria	Gammaproteobacteria	Oceanospirillales	Halomonadaceae	Halomonas	Halomonas sp. HG01
2	Bacteria	Proteobacteria	Gammaproteobacteria	Oceanospirillales	Halomonadaceae	Halomonas	Halomonas sp. BC1
2	Bacteria	Proteobacteria	Gammaproteobacteria	Oceanospirillales	Halomonadaceae	Halomonas	Halomonas smyrnensis
2	Bacteria	Proteobacteria	Gammaproteobacteria	Oceanospirillales	Halomonadaceae	Halomonas	Halomonas shengliensis
2	Bacteria	Proteobacteria	Gammaproteobacteria	Oceanospirillales	Halomonadaceae	Halomonas	Halomonas salina
2	Bacteria	Proteobacteria	Gammaproteobacteria	Oceanospirillales	Halomonadaceae	Halomonas	Halomonas pantelleriensis
2	Bacteria	Proteobacteria	Gammaproteobacteria	Oceanospirillales	Halomonadaceae	Halomonas	Halomonas halodenitrificans
2	Bacteria	Proteobacteria	Gammaproteobacteria	Oceanospirillales	Halomonadaceae	Halomonas	Halomonas elongata
2	Bacteria	Proteobacteria	Gammaproteobacteria	Oceanospirillales	Halomonadaceae	Halomonas	Halomonas arcis
2	Bacteria	Proteobacteria	Gammaproteobacteria	Oceanospirillales	Halomonadaceae	Halomonas	Halomonas anticariensis
2	Bacteria	Proteobacteria	Gammaproteobacteria	Oceanospirillales	Halomonadaceae	Chromohalobacter	Chromohalobacter japonicus
2	Bacteria	Proteobacteria	Gammaproteobacteria	Oceanospirillales	Halomonadaceae	Carnimonas	Carnimonas nigrificans
2	Bacteria	Proteobacteria	Gammaproteobacteria	Oceanospirillales	Halomonadaceae		
2	Bacteria	Proteobacteria	Gammaproteobacteria	Oceanospirillales	Hahellaceae	Endozoicomonas	Endozoicomonas montiporae
2	Bacteria	Proteobacteria	Gammaproteobacteria	Oceanospirillales	Alcanivoracaceae	Alcanivorax	Alcanivorax sp. Nap_24
2	Bacteria	Proteobacteria	Gammaproteobacteria	Oceanospirillales	Alcanivoracaceae	Alcanivorax	Alcanivorax nanhaiticus
2	Bacteria	Proteobacteria	Gammaproteobacteria	Nevskiales	Sinobacteraceae	Solimonas	Solimonas flava
2	Bacteria	Proteobacteria	Gammaproteobacteria	Methylococcales	Methylococcaceae	Methylosarcina	Methylosarcina lacus
2	Bacteria	Proteobacteria	Gammaproteobacteria	Methylococcales	Methylococcaceae	Methylomonas	Methylomonas sp. DH-1
2	Bacteria	Proteobacteria	Gammaproteobacteria	Methylococcales	Methylococcaceae	Methylomicrobium	Methylomicrobium buryatense
2	Bacteria	Proteobacteria	Gammaproteobacteria	Methylococcales	Methylococcaceae	Methylomicrobium	Methylomicrobium alcaliphilum
2	Bacteria	Proteobacteria	Gammaproteobacteria	Methylococcales	Methylococcaceae	Methylobacter	Methylobacter sp. BBA5.1
2	Bacteria	Proteobacteria	Gammaproteobacteria	Methylococcales			
2	Bacteria	Proteobacteria	Gammaproteobacteria	Legionellales	unclassified Legionellales	Legionellales bacterium RIFCSPHIGHO2_12_FULL_35_11	
2	Bacteria	Proteobacteria	Gammaproteobacteria	Legionellales	Legionellaceae	Tatlockia	Legionella maceachernii
2	Bacteria	Proteobacteria	Gammaproteobacteria	Legionellales	Legionellaceae	Legionella	Legionella tunisiensis
2	Bacteria	Proteobacteria	Gammaproteobacteria	Legionellales	Legionellaceae	Legionella	Legionella saoudiensis
2	Bacteria	Proteobacteria	Gammaproteobacteria	Legionellales	Legionellaceae	Legionella	Legionella sainthelensi
2	Bacteria	Proteobacteria	Gammaproteobacteria	Legionellales	Legionellaceae	Legionella	Legionella nautarum
2	Bacteria	Proteobacteria	Gammaproteobacteria	Legionellales	Legionellaceae	Legionella	Legionella moravica
2	Bacteria	Proteobacteria	Gammaproteobacteria	Legionellales	Legionellaceae	Legionella	Legionella longbeachae
2	Bacteria	Proteobacteria	Gammaproteobacteria	Legionellales	Legionellaceae	Legionella	Legionella israelensis
2	Bacteria	Proteobacteria	Gammaproteobacteria	Legionellales	Legionellaceae	Legionella	Legionella hackeliae
2	Bacteria	Proteobacteria	Gammaproteobacteria	Legionellales	Legionellaceae	Legionella	Legionella drozanskii
2	Bacteria	Proteobacteria	Gammaproteobacteria	Legionellales	Legionellaceae	Legionella	Legionella brunensis
2	Bacteria	Proteobacteria	Gammaproteobacteria	Legionellales	Legionellaceae	Fluoribacter	Fluoribacter dumoffii
2	Bacteria	Proteobacteria	Gammaproteobacteria	Legionellales	Coxiellaceae	Diplorickettsia	Diplorickettsia massiliensis
2	Bacteria	Proteobacteria	Gammaproteobacteria	Legionellales	Coxiellaceae	Candidatus Berkiella	Candidatus Berkiella aquae
2	Bacteria	Proteobacteria	Gammaproteobacteria	Enterobacterales	Yersiniaceae	Yersinia	Yersinia kristensenii
2	Bacteria	Proteobacteria	Gammaproteobacteria	Enterobacterales	Yersiniaceae	Serratia	Serratia symbiotica
2	Bacteria	Proteobacteria	Gammaproteobacteria	Enterobacterales	Yersiniaceae	Serratia	Serratia sp. M24T3
2	Bacteria	Proteobacteria	Gammaproteobacteria	Enterobacterales	Yersiniaceae	Serratia	Serratia sp. Ag2
2	Bacteria	Proteobacteria	Gammaproteobacteria	Enterobacterales	Pectobacteriaceae	Sodalis	Candidatus Sodalis sp. SoCistrobi
2	Bacteria	Proteobacteria	Gammaproteobacteria	Enterobacterales	Pectobacteriaceae	Dickeya	Dickeya zeae
2	Bacteria	Proteobacteria	Gammaproteobacteria	Enterobacterales	Pectobacteriaceae		
2	Bacteria	Proteobacteria	Gammaproteobacteria	Enterobacterales	Morganellaceae	Xenorhabdus	Xenorhabdus vietnamensis
2	Bacteria	Proteobacteria	Gammaproteobacteria	Enterobacterales	Morganellaceae	Xenorhabdus	Xenorhabdus nematophila
2	Bacteria	Proteobacteria	Gammaproteobacteria	Enterobacterales	Morganellaceae	Xenorhabdus	Xenorhabdus hominickii
2	Bacteria	Proteobacteria	Gammaproteobacteria	Enterobacterales	Morganellaceae	Providencia	Providencia stuartii
2	Bacteria	Proteobacteria	Gammaproteobacteria	Enterobacterales	Morganellaceae	Providencia	Providencia sneebia
2	Bacteria	Proteobacteria	Gammaproteobacteria	Enterobacterales	Morganellaceae	Providencia	Providencia alcalifaciens
2	Bacteria	Proteobacteria	Gammaproteobacteria	Enterobacterales	Morganellaceae	Providencia	
2	Bacteria	Proteobacteria	Gammaproteobacteria	Enterobacterales	Morganellaceae	Proteus	Proteus mirabilis
2	Bacteria	Proteobacteria	Gammaproteobacteria	Enterobacterales	Morganellaceae	Arsenophonus	Arsenophonus endosymbiont of Nilaparvata lugens
2	Bacteria	Proteobacteria	Gammaproteobacteria	Enterobacterales	Hafniaceae	Obesumbacterium	Obesumbacterium proteus
2	Bacteria	Proteobacteria	Gammaproteobacteria	Enterobacterales	Hafniaceae	Hafnia	Hafnia alvei
2	Bacteria	Proteobacteria	Gammaproteobacteria	Enterobacterales	Hafniaceae	Edwardsiella	Edwardsiella tarda
2	Bacteria	Proteobacteria	Gammaproteobacteria	Enterobacterales	Hafniaceae	Edwardsiella	Edwardsiella ictaluri
2	Bacteria	Proteobacteria	Gammaproteobacteria	Enterobacterales	Hafniaceae		
2	Bacteria	Proteobacteria	Gammaproteobacteria	Enterobacterales	Erwiniaceae	Pantoea	Pantoea vagans
2	Bacteria	Proteobacteria	Gammaproteobacteria	Enterobacterales	Erwiniaceae	Pantoea	Pantoea sp. OV426
2	Bacteria	Proteobacteria	Gammaproteobacteria	Enterobacterales	Erwiniaceae	Pantoea	Pantoea sp. IMH
2	Bacteria	Proteobacteria	Gammaproteobacteria	Enterobacterales	Erwiniaceae	Pantoea	Pantoea sp. At-9b
2	Bacteria	Proteobacteria	Gammaproteobacteria	Enterobacterales	Erwiniaceae	Pantoea	Pantoea rwandensis
2	Bacteria	Proteobacteria	Gammaproteobacteria	Enterobacterales	Erwiniaceae	Pantoea	Pantoea cypripedii
2	Bacteria	Proteobacteria	Gammaproteobacteria	Enterobacterales	Erwiniaceae	Erwinia	Erwinia tracheiphila
2	Bacteria	Proteobacteria	Gammaproteobacteria	Enterobacterales	Erwiniaceae	Erwinia	Erwinia sp. ErVv1
2	Bacteria	Proteobacteria	Gammaproteobacteria	Enterobacterales	Erwiniaceae	Erwinia	Erwinia gerundensis
2	Bacteria	Proteobacteria	Gammaproteobacteria	Enterobacterales	Enterobacteriaceae	unclassified Enterobacteriaceae	unclassified Enterobacteriaceae (miscellaneous)
2	Bacteria	Proteobacteria	Gammaproteobacteria	Enterobacterales	Enterobacteriaceae	unclassified Enterobacteriaceae	ant, tsetse, mealybug, aphid, etc. endosymbionts
2	Bacteria	Proteobacteria	Gammaproteobacteria	Enterobacterales	Enterobacteriaceae	Yokenella	Yokenella regensburgei
2	Bacteria	Proteobacteria	Gammaproteobacteria	Enterobacterales	Enterobacteriaceae	Shimwellia	Shimwellia blattae
2	Bacteria	Proteobacteria	Gammaproteobacteria	Enterobacterales	Enterobacteriaceae	Shigella	Shigella sonnei
2	Bacteria	Proteobacteria	Gammaproteobacteria	Enterobacterales	Enterobacteriaceae	Mangrovibacter	
2	Bacteria	Proteobacteria	Gammaproteobacteria	Enterobacterales	Enterobacteriaceae	Lelliottia	Lelliottia amnigena
2	Bacteria	Proteobacteria	Gammaproteobacteria	Enterobacterales	Enterobacteriaceae	Kosakonia	Kosakonia sacchari
2	Bacteria	Proteobacteria	Gammaproteobacteria	Enterobacterales	Enterobacteriaceae	Kosakonia	Kosakonia arachidis
2	Bacteria	Proteobacteria	Gammaproteobacteria	Enterobacterales	Enterobacteriaceae	Klebsiella	Klebsiella sp. RIT-PI-d
2	Bacteria	Proteobacteria	Gammaproteobacteria	Enterobacterales	Enterobacteriaceae	Klebsiella	Klebsiella oxytoca
2	Bacteria	Proteobacteria	Gammaproteobacteria	Enterobacterales	Enterobacteriaceae	Klebsiella	Klebsiella aerogenes
2	Bacteria	Proteobacteria	Gammaproteobacteria	Enterobacterales	Enterobacteriaceae	Izhakiella	Izhakiella sp. D4N98
2	Bacteria	Proteobacteria	Gammaproteobacteria	Enterobacterales	Enterobacteriaceae	Izhakiella	Izhakiella capsodis
2	Bacteria	Proteobacteria	Gammaproteobacteria	Enterobacterales	Enterobacteriaceae	Franconibacter	
2	Bacteria	Proteobacteria	Gammaproteobacteria	Enterobacterales	Enterobacteriaceae	Escherichia	Escherichia vulneris
2	Bacteria	Proteobacteria	Gammaproteobacteria	Enterobacterales	Enterobacteriaceae	Escherichia	Escherichia coli
2	Bacteria	Proteobacteria	Gammaproteobacteria	Enterobacterales	Enterobacteriaceae	Enterobacter	Enterobacter sp. kpr-6
2	Bacteria	Proteobacteria	Gammaproteobacteria	Enterobacterales	Enterobacteriaceae	Enterobacter	Enterobacter sp. Bisph1
2	Bacteria	Proteobacteria	Gammaproteobacteria	Enterobacterales	Enterobacteriaceae	Enterobacter	Enterobacter massiliensis
2	Bacteria	Proteobacteria	Gammaproteobacteria	Enterobacterales	Enterobacteriaceae	Enterobacter	Enterobacter cloacae complex
2	Bacteria	Proteobacteria	Gammaproteobacteria	Enterobacterales	Enterobacteriaceae	Enterobacter	Enterobacter cloacae complex
2	Bacteria	Proteobacteria	Gammaproteobacteria	Enterobacterales	Enterobacteriaceae	Enterobacter	Enterobacter cloacae complex
2	Bacteria	Proteobacteria	Gammaproteobacteria	Enterobacterales	Enterobacteriaceae	Buttiauxella	Buttiauxella gaviniae
2	Bacteria	Proteobacteria	Gammaproteobacteria	Chromatiales	environmental samples	uncultured Chromatiales bacterium HF0200_41F04	
2	Bacteria	Proteobacteria	Gammaproteobacteria	Chromatiales	Ectothiorhodospiraceae	Thioalkalivibrio	Thioalkalivibrio sp. ALMg11
2	Bacteria	Proteobacteria	Gammaproteobacteria	Chromatiales	Ectothiorhodospiraceae	Thioalkalivibrio	Thioalkalivibrio sp. ALM2T
2	Bacteria	Proteobacteria	Gammaproteobacteria	Chromatiales	Ectothiorhodospiraceae	Thioalkalivibrio	Thioalkalivibrio sp. ALJT
2	Bacteria	Proteobacteria	Gammaproteobacteria	Chromatiales	Ectothiorhodospiraceae	Thioalkalivibrio	Thioalkalivibrio sp. ALJ24
2	Bacteria	Proteobacteria	Gammaproteobacteria	Chromatiales	Ectothiorhodospiraceae	Thioalkalivibrio	Thioalkalivibrio sp. ALJ16
2	Bacteria	Proteobacteria	Gammaproteobacteria	Chromatiales	Ectothiorhodospiraceae	Thioalkalivibrio	Thioalkalivibrio sp. AKL11
2	Bacteria	Proteobacteria	Gammaproteobacteria	Chromatiales	Ectothiorhodospiraceae	Ectothiorhodospira	Ectothiorhodospira sp. BSL-9
2	Bacteria	Proteobacteria	Gammaproteobacteria	Chromatiales	Ectothiorhodospiraceae	Ectothiorhodospira	Ectothiorhodospira marina
2	Bacteria	Proteobacteria	Gammaproteobacteria	Chromatiales	Ectothiorhodospiraceae	Ectothiorhodospira	
2	Bacteria	Proteobacteria	Gammaproteobacteria	Chromatiales	Ectothiorhodospiraceae	Acidihalobacter	
2	Bacteria	Proteobacteria	Gammaproteobacteria	Chromatiales	Chromatiaceae	Thiocystis	Thiocystis violascens
2	Bacteria	Proteobacteria	Gammaproteobacteria	Chromatiales	Chromatiaceae	Thiocapsa	Thiocapsa marina
2	Bacteria	Proteobacteria	Gammaproteobacteria	Chromatiales	Chromatiaceae	Rheinheimera	Rheinheimera sp. F8
2	Bacteria	Proteobacteria	Gammaproteobacteria	Chromatiales	Chromatiaceae	Rheinheimera	Rheinheimera sp. EpRS3
2	Bacteria	Proteobacteria	Gammaproteobacteria	Chromatiales	Chromatiaceae	Rheinheimera	Rheinheimera pacifica
2	Bacteria	Proteobacteria	Gammaproteobacteria	Chromatiales	Chromatiaceae	Rheinheimera	Rheinheimera mesophila
2	Bacteria	Proteobacteria	Gammaproteobacteria	Chromatiales	Chromatiaceae	Rheinheimera	Rheinheimera baltica
2	Bacteria	Proteobacteria	Gammaproteobacteria	Chromatiales	Chromatiaceae	Arsukibacterium	Arsukibacterium sp. MJ3
2	Bacteria	Proteobacteria	Gammaproteobacteria	Cellvibrionales	Spongiibacteraceae	unclassified Spongiibacteraceae	gamma proteobacterium BDW918
2	Bacteria	Proteobacteria	Gammaproteobacteria	Cellvibrionales	Halieaceae		
2	Bacteria	Proteobacteria	Gammaproteobacteria	Cellvibrionales	Cellvibrionaceae	Marinimicrobium	
2	Bacteria	Proteobacteria	Gammaproteobacteria	Cardiobacteriales	Cardiobacteriaceae	Rappaport	Rappaport israeli
2	Bacteria	Proteobacteria	Gammaproteobacteria	Alteromonadales	Shewanellaceae	Shewanella	Shewanella sp. UCD-KL12
2	Bacteria	Proteobacteria	Gammaproteobacteria	Alteromonadales	Shewanellaceae	Shewanella	Shewanella marina
2	Bacteria	Proteobacteria	Gammaproteobacteria	Alteromonadales	Shewanellaceae	Shewanella	Shewanella frigidimarina
2	Bacteria	Proteobacteria	Gammaproteobacteria	Alteromonadales	Shewanellaceae	Shewanella	Shewanella fidelis
2	Bacteria	Proteobacteria	Gammaproteobacteria	Alteromonadales	Shewanellaceae	Shewanella	Shewanella decolorationis
2	Bacteria	Proteobacteria	Gammaproteobacteria	Alteromonadales	Psychromonadaceae	Psychromonas	Psychromonas sp. SP041
2	Bacteria	Proteobacteria	Gammaproteobacteria	Alteromonadales	Psychromonadaceae	Psychromonas	Psychromonas ingrahamii
2	Bacteria	Proteobacteria	Gammaproteobacteria	Alteromonadales	Psychromonadaceae	Psychromonas	Psychromonas arctica
2	Bacteria	Proteobacteria	Gammaproteobacteria	Alteromonadales	Psychromonadaceae	Psychromonas	Psychromonas aquimarina
2	Bacteria	Proteobacteria	Gammaproteobacteria	Alteromonadales	Pseudoalteromonadaceae	Pseudoalteromonas	Pseudoalteromonas ruthenica
2	Bacteria	Proteobacteria	Gammaproteobacteria	Alteromonadales	Pseudoalteromonadaceae	Pseudoalteromonas	Pseudoalteromonas rubra
2	Bacteria	Proteobacteria	Gammaproteobacteria	Alteromonadales	Pseudoalteromonadaceae	Pseudoalteromonas	Pseudoalteromonas prydzensis
2	Bacteria	Proteobacteria	Gammaproteobacteria	Alteromonadales	Moritellaceae	Moritella	Moritella marina
2	Bacteria	Proteobacteria	Gammaproteobacteria	Alteromonadales	Moritellaceae	Moritella	Moritella dasanensis
2	Bacteria	Proteobacteria	Gammaproteobacteria	Alteromonadales	Idiomarinaceae	Idiomarina	Idiomarina sp. 5.13
2	Bacteria	Proteobacteria	Gammaproteobacteria	Alteromonadales	Idiomarinaceae	Idiomarina	Idiomarina salinarum
2	Bacteria	Proteobacteria	Gammaproteobacteria	Alteromonadales	Idiomarinaceae	Idiomarina	Idiomarina maritima
2	Bacteria	Proteobacteria	Gammaproteobacteria	Alteromonadales	Ferrimonadaceae	Ferrimonas	
2	Bacteria	Proteobacteria	Gammaproteobacteria	Alteromonadales	Colwelliaceae	Colwellia	Colwellia sp. QMS0
2	Bacteria	Proteobacteria	Gammaproteobacteria	Alteromonadales	Colwelliaceae	Colwellia	Colwellia piezophila
2	Bacteria	Proteobacteria	Gammaproteobacteria	Alteromonadales	Colwelliaceae	Colwellia	Colwellia mytili
2	Bacteria	Proteobacteria	Gammaproteobacteria	Alteromonadales	Colwelliaceae	Colwellia	Colwellia chukchiensis
2	Bacteria	Proteobacteria	Gammaproteobacteria	Alteromonadales	Alteromonadaceae	Marinobacter	Marinobacter sp. BSs20148
2	Bacteria	Proteobacteria	Gammaproteobacteria	Alteromonadales	Alteromonadaceae	Marinobacter	Marinobacter similis
2	Bacteria	Proteobacteria	Gammaproteobacteria	Alteromonadales	Alteromonadaceae	Marinobacter	Marinobacter psychrophilus
2	Bacteria	Proteobacteria	Gammaproteobacteria	Alteromonadales	Alteromonadaceae	Marinobacter	Marinobacter nitratireducens
2	Bacteria	Proteobacteria	Gammaproteobacteria	Alteromonadales	Alteromonadaceae	Marinobacter	Marinobacter antarcticus
2	Bacteria	Proteobacteria	Gammaproteobacteria	Alteromonadales	Alteromonadaceae	Glaciecola	Glaciecola pallidula
2	Bacteria	Proteobacteria	Gammaproteobacteria	Alteromonadales	Alteromonadaceae	Alteromonas	Alteromonas sp. ALT199
2	Bacteria	Proteobacteria	Gammaproteobacteria	Alteromonadales	Alteromonadaceae	Alteromonas	Alteromonas naphthalenivorans
2	Bacteria	Proteobacteria	Gammaproteobacteria	Alteromonadales	Alteromonadaceae	Alteromonas	Alteromonas australica
2	Bacteria	Proteobacteria	Gammaproteobacteria	Alteromonadales	Alteromonadaceae	Agarivorans	Agarivorans albus
2	Bacteria	Proteobacteria	Gammaproteobacteria	Aeromonadales	Succinivibrionaceae	unclassified Succinivibrionaceae	Succinivibrionaceae bacterium WG-1
2	Bacteria	Proteobacteria	Gammaproteobacteria	Aeromonadales	Aeromonadaceae	Aeromonas	Aeromonas sp. DNP9
2	Bacteria	Proteobacteria	Gammaproteobacteria	Aeromonadales	Aeromonadaceae	Aeromonas	Aeromonas sp. 159
2	Bacteria	Proteobacteria	Gammaproteobacteria	Aeromonadales	Aeromonadaceae	Aeromonas	Aeromonas sobria
2	Bacteria	Proteobacteria	Gammaproteobacteria	Aeromonadales	Aeromonadaceae	Aeromonas	Aeromonas simiae
2	Bacteria	Proteobacteria	Gammaproteobacteria	Aeromonadales	Aeromonadaceae	Aeromonas	Aeromonas sanarellii
2	Bacteria	Proteobacteria	Gammaproteobacteria	Aeromonadales	Aeromonadaceae	Aeromonas	Aeromonas popoffii
2	Bacteria	Proteobacteria	Gammaproteobacteria	Aeromonadales	Aeromonadaceae	Aeromonas	Aeromonas fluvialis
2	Bacteria	Proteobacteria	Candidatus Muproteobacteria	Candidatus Muproteobacteria bacterium RBG_16_64_10			
2	Bacteria	Proteobacteria	Betaproteobacteria	unclassified Betaproteobacteria	unclassified Betaproteobacteria (miscellaneous)	Betaproteobacteria bacterium RIFCSPLOWO2_02_FULL_62_79	
2	Bacteria	Proteobacteria	Betaproteobacteria	unclassified Betaproteobacteria	Kinetoplastibacterium	Candidatus Kinetoplastibacterium galatii	
2	Bacteria	Proteobacteria	Betaproteobacteria	unclassified Betaproteobacteria	Kinetoplastibacterium	Candidatus Kinetoplastibacterium desouzaii	
2	Bacteria	Proteobacteria	Betaproteobacteria	unclassified Betaproteobacteria	Kinetoplastibacterium	Candidatus Kinetoplastibacterium crithidii	
2	Bacteria	Proteobacteria	Betaproteobacteria	unclassified Betaproteobacteria			
2	Bacteria	Proteobacteria	Betaproteobacteria	environmental samples	uncultured beta proteobacterium		
2	Bacteria	Proteobacteria	Betaproteobacteria	Nitrosomonadales	Nitrosomonadaceae	environmental samples	uncultured beta proteobacterium HF0130_04F21
2	Bacteria	Proteobacteria	Betaproteobacteria	Neisseriales	Neisseriaceae	Snodgrassella	
2	Bacteria	Proteobacteria	Betaproteobacteria	Neisseriales	Neisseriaceae	Neisseria	Neisseria sicca
2	Bacteria	Proteobacteria	Betaproteobacteria	Neisseriales	Neisseriaceae	Neisseria	Neisseria gonorrhoeae
2	Bacteria	Proteobacteria	Betaproteobacteria	Neisseriales	Neisseriaceae	Neisseria	Neisseria bacilliformis
2	Bacteria	Proteobacteria	Betaproteobacteria	Neisseriales	Neisseriaceae	Kingella	Kingella oralis
2	Bacteria	Proteobacteria	Betaproteobacteria	Neisseriales	Neisseriaceae	Conchiformibius	Conchiformibius steedae
2	Bacteria	Proteobacteria	Betaproteobacteria	Neisseriales	Neisseriaceae	Alysiella	Alysiella crassa
2	Bacteria	Proteobacteria	Betaproteobacteria	Neisseriales	Chromobacteriaceae	Pseudogulbenkiania	Pseudogulbenkiania ferrooxidans
2	Bacteria	Proteobacteria	Betaproteobacteria	Neisseriales	Chromobacteriaceae	Gulbenkiania	Gulbenkiania mobilis
2	Bacteria	Proteobacteria	Betaproteobacteria	Neisseriales	Chromobacteriaceae	Chromobacterium group	Chromobacterium
2	Bacteria	Proteobacteria	Betaproteobacteria	Neisseriales	Chromobacteriaceae	Chromobacterium group	Chromobacterium
2	Bacteria	Proteobacteria	Betaproteobacteria	Neisseriales	Chromobacteriaceae	Aquitalea	Aquitalea pelogenes
2	Bacteria	Proteobacteria	Betaproteobacteria	Methylophilales	Methylophilaceae	Methylophilus	Methylophilus sp. Leaf414
2	Bacteria	Proteobacteria	Betaproteobacteria	Hydrogenophilales	Hydrogenophilaceae	Thiobacillus	Thiobacillus sp. SCN 64-35
2	Bacteria	Proteobacteria	Betaproteobacteria	Gallionellales	unclassified Gallionellales	Gallionellales bacterium RIFCSPLOWO2_12_FULL_59_22	
2	Bacteria	Proteobacteria	Betaproteobacteria	Gallionellales	unclassified Gallionellales	Gallionellales bacterium RIFCSPLOWO2_02_FULL_57_47	
2	Bacteria	Proteobacteria	Betaproteobacteria	Gallionellales	unclassified Gallionellales	Gallionellales bacterium RIFCSPLOWO2_02_60_31	
2	Bacteria	Proteobacteria	Betaproteobacteria	Gallionellales	unclassified Gallionellales	Gallionellales bacterium RIFCSPLOWO2_02_58_13	
2	Bacteria	Proteobacteria	Betaproteobacteria	Gallionellales	unclassified Gallionellales	Gallionellales bacterium RBG_16_56_9	
2	Bacteria	Proteobacteria	Betaproteobacteria	Gallionellales	unclassified Gallionellales	Gallionellales bacterium GWE2_58_10	
2	Bacteria	Proteobacteria	Betaproteobacteria	Gallionellales	unclassified Gallionellales	Gallionellales bacterium GWA2_59_43	
2	Bacteria	Proteobacteria	Betaproteobacteria	Gallionellales	Gallionellaceae	unclassified Gallionellaceae	
2	Bacteria	Proteobacteria	Betaproteobacteria	Burkholderiales	unclassified Burkholderiales	unclassified Burkholderiales (miscellaneous)	Burkholderiales bacterium RIFCSPLOWO2_12_FULL_67_210
2	Bacteria	Proteobacteria	Betaproteobacteria	Burkholderiales	unclassified Burkholderiales	unclassified Burkholderiales (miscellaneous)	Burkholderiales bacterium GWE1_65_30
2	Bacteria	Proteobacteria	Betaproteobacteria	Burkholderiales	unclassified Burkholderiales	unclassified Burkholderiales (miscellaneous)	Burkholderiales bacterium 68-20
2	Bacteria	Proteobacteria	Betaproteobacteria	Burkholderiales	unclassified Burkholderiales	Burkholderiales Genera incertae sedis	Methylibium
2	Bacteria	Proteobacteria	Betaproteobacteria	Burkholderiales	Sutterellaceae	Sutterella	environmental samples
2	Bacteria	Proteobacteria	Betaproteobacteria	Burkholderiales	Oxalobacteraceae	Massilia	Massilia sp. LC238
2	Bacteria	Proteobacteria	Betaproteobacteria	Burkholderiales	Oxalobacteraceae	Janthinobacterium	Janthinobacterium sp. TND4EL3
2	Bacteria	Proteobacteria	Betaproteobacteria	Burkholderiales	Oxalobacteraceae	Janthinobacterium	Janthinobacterium sp. HH104
2	Bacteria	Proteobacteria	Betaproteobacteria	Burkholderiales	Oxalobacteraceae	Janthinobacterium	Janthinobacterium sp. 1_2014MBL_MicDiv
2	Bacteria	Proteobacteria	Betaproteobacteria	Burkholderiales	Oxalobacteraceae	Herbaspirillum	Herbaspirillum sp. B39
2	Bacteria	Proteobacteria	Betaproteobacteria	Burkholderiales	Oxalobacteraceae	Duganella	Duganella zoogloeoides
2	Bacteria	Proteobacteria	Betaproteobacteria	Burkholderiales	Oxalobacteraceae	Duganella	Duganella sp. Root198D2
2	Bacteria	Proteobacteria	Betaproteobacteria	Burkholderiales	Oxalobacteraceae	Duganella	Duganella sp. HH105
2	Bacteria	Proteobacteria	Betaproteobacteria	Burkholderiales	Comamonadaceae	unclassified Comamonadaceae	Comamonadaceae bacterium CCH12-A10
2	Bacteria	Proteobacteria	Betaproteobacteria	Burkholderiales	Comamonadaceae	Verminephrobacter	
2	Bacteria	Proteobacteria	Betaproteobacteria	Burkholderiales	Comamonadaceae	Variovorax	Variovorax sp. YR634
2	Bacteria	Proteobacteria	Betaproteobacteria	Burkholderiales	Comamonadaceae	Variovorax	Variovorax sp. HH01
2	Bacteria	Proteobacteria	Betaproteobacteria	Burkholderiales	Comamonadaceae	Rhodoferax	Rhodoferax antarcticus
2	Bacteria	Proteobacteria	Betaproteobacteria	Burkholderiales	Comamonadaceae	Polaromonas	Polaromonas naphthalenivorans
2	Bacteria	Proteobacteria	Betaproteobacteria	Burkholderiales	Comamonadaceae	Hydrogenophaga	Hydrogenophaga sp. SCN 70-13
2	Bacteria	Proteobacteria	Betaproteobacteria	Burkholderiales	Comamonadaceae	Hydrogenophaga	Hydrogenophaga sp. 70-12
2	Bacteria	Proteobacteria	Betaproteobacteria	Burkholderiales	Comamonadaceae	Acidovorax	Acidovorax sp. SCN 65-108
2	Bacteria	Proteobacteria	Betaproteobacteria	Burkholderiales	Comamonadaceae	Acidovorax	Acidovorax sp. Leaf84
2	Bacteria	Proteobacteria	Betaproteobacteria	Burkholderiales	Burkholderiaceae	Ralstonia	unclassified Ralstonia
2	Bacteria	Proteobacteria	Betaproteobacteria	Burkholderiales	Burkholderiaceae	Ralstonia	Ralstonia syzygii
2	Bacteria	Proteobacteria	Betaproteobacteria	Burkholderiales	Burkholderiaceae	Ralstonia	Ralstonia sp. UNC404CL21Col
2	Bacteria	Proteobacteria	Betaproteobacteria	Burkholderiales	Burkholderiaceae	Ralstonia	Ralstonia sp. A12
2	Bacteria	Proteobacteria	Betaproteobacteria	Burkholderiales	Burkholderiaceae	Paraburkholderia	Paraburkholderia rhizoxinica
2	Bacteria	Proteobacteria	Betaproteobacteria	Burkholderiales	Burkholderiaceae	Paraburkholderia	Paraburkholderia caledonica
2	Bacteria	Proteobacteria	Betaproteobacteria	Burkholderiales	Burkholderiaceae	Paraburkholderia	Paraburkholderia bryophila

2	Bacteria	Proteobacteria	Betaproteobacteria	Burkholderiales	Burkholderiaceae	Paraburkholderia	Paraburkholderia aspalathi
2	Bacteria	Proteobacteria	Betaproteobacteria	Burkholderiales	Burkholderiaceae	Pandoraea	Pandoraea sp. ISTKB
2	Bacteria	Proteobacteria	Betaproteobacteria	Burkholderiales	Burkholderiaceae	Pandoraea	Pandoraea pulmonicola
2	Bacteria	Proteobacteria	Betaproteobacteria	Burkholderiales	Burkholderiaceae	Pandoraea	Pandoraea oxalativorans
2	Bacteria	Proteobacteria	Betaproteobacteria	Burkholderiales	Burkholderiaceae	Pandoraea	Pandoraea apista
2	Bacteria	Proteobacteria	Betaproteobacteria	Burkholderiales	Burkholderiaceae	Chitinimonas	
2	Bacteria	Proteobacteria	Betaproteobacteria	Burkholderiales	Burkholderiaceae	Caballeronia	Caballeronia terrestris
2	Bacteria	Proteobacteria	Betaproteobacteria	Burkholderiales	Burkholderiaceae	Caballeronia	Caballeronia megalochromosomata
2	Bacteria	Proteobacteria	Betaproteobacteria	Burkholderiales	Burkholderiaceae	Burkholderia	pseudomallei group
2	Bacteria	Proteobacteria	Betaproteobacteria	Burkholderiales	Burkholderiaceae	Burkholderia	pseudomallei group
2	Bacteria	Proteobacteria	Betaproteobacteria	Burkholderiales	Burkholderiaceae	Burkholderia	pseudomallei group
2	Bacteria	Proteobacteria	Betaproteobacteria	Burkholderiales	Burkholderiaceae	Burkholderia	Candidatus Burkholderia pumila
2	Bacteria	Proteobacteria	Betaproteobacteria	Burkholderiales	Burkholderiaceae	Burkholderia	Burkholderia sp. b13
2	Bacteria	Proteobacteria	Betaproteobacteria	Burkholderiales	Burkholderiaceae	Burkholderia	Burkholderia sp. WSM2232
2	Bacteria	Proteobacteria	Betaproteobacteria	Burkholderiales	Burkholderiaceae	Burkholderia	Burkholderia sp. WSM2230
2	Bacteria	Proteobacteria	Betaproteobacteria	Burkholderiales	Burkholderiaceae	Burkholderia	Burkholderia sp. RPE67
2	Bacteria	Proteobacteria	Betaproteobacteria	Burkholderiales	Burkholderiaceae	Burkholderia	Burkholderia sp. PAMC 26561
2	Bacteria	Proteobacteria	Betaproteobacteria	Burkholderiales	Burkholderiaceae	Burkholderia	Burkholderia sp. K24
2	Bacteria	Proteobacteria	Betaproteobacteria	Burkholderiales	Burkholderiaceae	Burkholderia	Burkholderia sp. CCGE1003
2	Bacteria	Proteobacteria	Betaproteobacteria	Burkholderiales	Burkholderiaceae	Burkholderia	Burkholderia sp. CCGE1001
2	Bacteria	Proteobacteria	Betaproteobacteria	Burkholderiales	Burkholderiaceae	Burkholderia	Burkholderia sp. A9
2	Bacteria	Proteobacteria	Betaproteobacteria	Burkholderiales	Burkholderiaceae	Burkholderia	Burkholderia glebae
2	Bacteria	Proteobacteria	Betaproteobacteria	Burkholderiales	Burkholderiaceae	Burkholderia	Burkholderia cepacia complex
2	Bacteria	Proteobacteria	Betaproteobacteria	Burkholderiales	Burkholderiaceae	Burkholderia	Burkholderia cepacia complex
2	Bacteria	Proteobacteria	Betaproteobacteria	Burkholderiales	Burkholderiaceae	Burkholderia	Burkholderia cepacia complex
2	Bacteria	Proteobacteria	Betaproteobacteria	Burkholderiales	Burkholderiaceae	Burkholderia	Burkholderia cepacia complex
2	Bacteria	Proteobacteria	Betaproteobacteria	Burkholderiales	Burkholderiaceae	Burkholderia	Burkholderia cepacia complex
2	Bacteria	Proteobacteria	Betaproteobacteria	Burkholderiales	Alcaligenaceae	Oligella	Oligella urethralis
2	Bacteria	Proteobacteria	Betaproteobacteria	Burkholderiales	Alcaligenaceae	Oligella	Oligella ureolytica
2	Bacteria	Proteobacteria	Betaproteobacteria	Burkholderiales	Alcaligenaceae	Oligella	Oligella sp. HMSC09E12
2	Bacteria	Proteobacteria	Betaproteobacteria	Burkholderiales	Alcaligenaceae	Bordetella	Bordetella sp. SCN 67-23
2	Bacteria	Proteobacteria	Betaproteobacteria	Burkholderiales	Alcaligenaceae	Bordetella	Bordetella parapertussis
2	Bacteria	Proteobacteria	Betaproteobacteria	Burkholderiales	Alcaligenaceae	Bordetella	Bordetella holmesii
2	Bacteria	Proteobacteria	Betaproteobacteria	Burkholderiales	Alcaligenaceae	Achromobacter	Achromobacter spanius
2	Bacteria	Proteobacteria	Betaproteobacteria	Burkholderiales	Alcaligenaceae	Achromobacter	Achromobacter sp. ATCC8750
2	Bacteria	Proteobacteria	Betaproteobacteria	Burkholderiales	Alcaligenaceae	Achromobacter	Achromobacter sp. ATCC31444
2	Bacteria	Proteobacteria	Betaproteobacteria	Burkholderiales	Alcaligenaceae	Achromobacter	Achromobacter ruhlandii
2	Bacteria	Proteobacteria	Betaproteobacteria	Burkholderiales	Alcaligenaceae	Achromobacter	Achromobacter arsenitoxydans
2	Bacteria	Proteobacteria	Alphaproteobacteria	unclassified Alphaproteobacteria	unclassified Alphaproteobacteria (miscellaneous)	alpha proteobacterium L41A	
2	Bacteria	Proteobacteria	Alphaproteobacteria	unclassified Alphaproteobacteria	unclassified Alphaproteobacteria (miscellaneous)	Alphaproteobacteria bacterium 43-37	
2	Bacteria	Proteobacteria	Alphaproteobacteria	unclassified Alphaproteobacteria	unclassified Alphaproteobacteria (miscellaneous)	Alphaproteobacteria bacterium 13_1_20CM_4_65_11	
2	Bacteria	Proteobacteria	Alphaproteobacteria	environmental samples	uncultured alpha proteobacterium HF0130_06E21		
2	Bacteria	Proteobacteria	Alphaproteobacteria	environmental samples	uncultured alpha proteobacterium 01-003886		
2	Bacteria	Proteobacteria	Alphaproteobacteria	Sphingomonadales	Sphingomonadaceae	Sphingomonas	Sphingomonas sp. STIS6.2
2	Bacteria	Proteobacteria	Alphaproteobacteria	Sphingomonadales	Sphingomonadaceae	Sphingomonas	Sphingomonas sp. NS2
2	Bacteria	Proteobacteria	Alphaproteobacteria	Sphingomonadales	Sphingomonadaceae	Sphingomonas	Sphingomonas sp. Leaf62
2	Bacteria	Proteobacteria	Alphaproteobacteria	Sphingomonadales	Sphingomonadaceae	Sphingomonas	Sphingomonas sp. Leaf34
2	Bacteria	Proteobacteria	Alphaproteobacteria	Sphingomonadales	Sphingomonadaceae	Sphingomonas	Sphingomonas sp. Leaf30
2	Bacteria	Proteobacteria	Alphaproteobacteria	Sphingomonadales	Sphingomonadaceae	Sphingomonas	Sphingomonas sp. Leaf257
2	Bacteria	Proteobacteria	Alphaproteobacteria	Sphingomonadales	Sphingomonadaceae	Sphingomonas	Sphingomonas sp. FUKUSWIS1
2	Bacteria	Proteobacteria	Alphaproteobacteria	Sphingomonadales	Sphingomonadaceae	Sphingomonas	Sphingomonas sp. CCH9-E2
2	Bacteria	Proteobacteria	Alphaproteobacteria	Sphingomonadales	Sphingomonadaceae	Sphingomonas	Sphingomonas sp. CCH5-D11
2	Bacteria	Proteobacteria	Alphaproteobacteria	Sphingomonadales	Sphingomonadaceae	Sphingomonas	Sphingomonas parapaucimobilis
2	Bacteria	Proteobacteria	Alphaproteobacteria	Sphingomonadales	Sphingomonadaceae	Sphingobium	Sphingobium ummariense
2	Bacteria	Proteobacteria	Alphaproteobacteria	Sphingomonadales	Sphingomonadaceae	Sphingobium	Sphingobium sp. YBL2
2	Bacteria	Proteobacteria	Alphaproteobacteria	Sphingomonadales	Sphingomonadaceae	Sphingobium	Sphingobium quisquiliarum
2	Bacteria	Proteobacteria	Alphaproteobacteria	Sphingomonadales	Sphingomonadaceae	Sphingobium	Sphingobium herbicidovorans
2	Bacteria	Proteobacteria	Alphaproteobacteria	Sphingomonadales	Sphingomonadaceae	Novosphingobium	Novosphingobium aromaticivorans
2	Bacteria	Proteobacteria	Alphaproteobacteria	Sphingomonadales	Sphingomonadaceae	Citromicrobium	Citromicrobium sp. WPS32
2	Bacteria	Proteobacteria	Alphaproteobacteria	Sphingomonadales	Erythrobacteraceae	Erythrobacter	Erythrobacter sp. HI0059
2	Bacteria	Proteobacteria	Alphaproteobacteria	Rickettsiales	Rickettsiaceae	unclassified Rickettsiaceae	Rickettsiaceae bacterium 4572_127
2	Bacteria	Proteobacteria	Alphaproteobacteria	Rickettsiales	Rickettsiaceae	Rickettsieae	Rickettsia
2	Bacteria	Proteobacteria	Alphaproteobacteria	Rickettsiales	Rickettsiaceae	Rickettsieae	Rickettsia
2	Bacteria	Proteobacteria	Alphaproteobacteria	Rickettsiales	Rickettsiaceae	Rickettsieae	Orientia
2	Bacteria	Proteobacteria	Alphaproteobacteria	Rickettsiales	Anaplasmataceae	Wolbachieae	Wolbachia
2	Bacteria	Proteobacteria	Alphaproteobacteria	Rickettsiales	Anaplasmataceae	Wolbachieae	Wolbachia
2	Bacteria	Proteobacteria	Alphaproteobacteria	Rhodospirillales	unclassified Rhodospirillales	unclassified Rhodospirillales (miscellaneous)	Rhodospirillales bacterium SCN 65-16
2	Bacteria	Proteobacteria	Alphaproteobacteria	Rhodospirillales	environmental samples	uncultured Rhodospirillales bacterium HF4000_24M03	
2	Bacteria	Proteobacteria	Alphaproteobacteria	Rhodospirillales	Rhodospirillaceae	Tistlia	Tistlia consotensis
2	Bacteria	Proteobacteria	Alphaproteobacteria	Rhodospirillales	Rhodospirillaceae	Thalassospira	Thalassospira sp. MCCC 1A01428
2	Bacteria	Proteobacteria	Alphaproteobacteria	Rhodospirillales	Rhodospirillaceae	Thalassospira	Thalassospira alkalitolerans
2	Bacteria	Proteobacteria	Alphaproteobacteria	Rhodospirillales	Rhodospirillaceae	Azospirillum	Azospirillum sp. 51_20
2	Bacteria	Proteobacteria	Alphaproteobacteria	Rhodospirillales	Acetobacteraceae	Saccharibacter	Saccharibacter floricola
2	Bacteria	Proteobacteria	Alphaproteobacteria	Rhodospirillales	Acetobacteraceae	Neokomagataea	Neokomagataea thailandica
2	Bacteria	Proteobacteria	Alphaproteobacteria	Rhodospirillales	Acetobacteraceae	Neoasaia	Neoasaia chiangmaiensis
2	Bacteria	Proteobacteria	Alphaproteobacteria	Rhodospirillales	Acetobacteraceae	Komagataeibacter	Komagataeibacter xylinus
2	Bacteria	Proteobacteria	Alphaproteobacteria	Rhodospirillales	Acetobacteraceae	Komagataeibacter	Komagataeibacter kakiaceti
2	Bacteria	Proteobacteria	Alphaproteobacteria	Rhodospirillales	Acetobacteraceae	Komagataeibacter	Komagataeibacter intermedius
[illegible]	[illegible]	[illegible]	[illegible]	[illegible]	[illegible]	[illegible]	[illegible]

2	Bacteria	FCB group	Bacteroidetes/Chlorobi group	Bacteroidetes	Bacteroidia	Bacteroidales	Bacteroidaceae
2	Bacteria	FCB group	Bacteroidetes/Chlorobi group	Bacteroidetes	Bacteroidia	Bacteroidales	Bacteroidaceae
2	Bacteria	FCB group	Bacteroidetes/Chlorobi group	Bacteroidetes	Bacteroidia	Bacteroidales	Bacteroidaceae
2	Bacteria	FCB group	Bacteroidetes/Chlorobi group	Bacteroidetes	Bacteroidia	Bacteroidales	Bacteroidaceae
2	Bacteria	FCB group	Bacteroidetes/Chlorobi group	Bacteroidetes	Bacteroidia	Bacteroidales	Bacteroidaceae
2	Bacteria	FCB group	Bacteroidetes/Chlorobi group	Bacteroidetes	Bacteroidia	Bacteroidales	Bacteroidaceae
2	Bacteria	FCB group	Bacteroidetes/Chlorobi group	Bacteroidetes	Bacteroidia	Bacteroidales	Bacteroidaceae
2	Bacteria	FCB group	Bacteroidetes/Chlorobi group	Bacteroidetes	Bacteroidia	Bacteroidales	Bacteroidaceae
2	Bacteria	FCB group	Bacteroidetes/Chlorobi group	Bacteroidetes	Bacteroidia	Bacteroidales	Bacteroidaceae
2	Bacteria	FCB group	Bacteroidetes/Chlorobi group	Bacteroidetes	Bacteroidia	Bacteroidales	Bacteroidaceae
2	Bacteria	FCB group	Bacteroidetes/Chlorobi group	Bacteroidetes	Bacteroidia	Bacteroidales	Bacteroidaceae
2	Bacteria	FCB group	Bacteroidetes/Chlorobi group	Bacteroidetes	Bacteroidia	Bacteroidales	Bacteroidaceae
2	Bacteria	FCB group	Bacteroidetes/Chlorobi group	Bacteroidetes	Bacteroidia	Bacteroidales	Bacteroidaceae
2	Bacteria	FCB group	Bacteroidetes/Chlorobi group	Bacteroidetes	Bacteroidia	Bacteroidales	Bacteroidaceae
2	Bacteria	FCB group	Bacteroidetes/Chlorobi group	Bacteroidetes	Bacteroidia	Bacteroidales	Bacteroidaceae
2	Bacteria	FCB group	Bacteroidetes/Chlorobi group	Bacteroidetes	Bacteroidia	Bacteroidales	Bacteroidaceae
2	Bacteria	FCB group	Bacteroidetes/Chlorobi group	Bacteroidetes	Bacteroidetes Order II. Incertae sedis	Rhodothermaceae	Salinibacter
2	Bacteria	FCB group	Bacteroidetes/Chlorobi group	Bacteroidetes	Bacteroidetes Order II. Incertae sedis	Rhodothermaceae	
2	Bacteria	Elusimicrobia	unclassified Elusimicrobia	Elusimicrobia bacterium RIFOXYB2_FULL_62_6			
2	Bacteria	Elusimicrobia	unclassified Elusimicrobia	Elusimicrobia bacterium RIFOXYA2_FULL_39_19			
2	Bacteria	Elusimicrobia	unclassified Elusimicrobia	Elusimicrobia bacterium RIFCSPLOWO2_12_FULL_59_9			
2	Bacteria	Elusimicrobia	unclassified Elusimicrobia	Elusimicrobia bacterium RIFCSPHIGHO2_02_FULL_57_9			
2	Bacteria	Elusimicrobia	unclassified Elusimicrobia	Elusimicrobia bacterium GWD2_63_28			
2	Bacteria	Elusimicrobia					
2	Bacteria	Dictyoglomi	Dictyoglomia	Dictyoglomales	Dictyoglomaceae	Dictyoglomus	Dictyoglomus thermophilum
2	Bacteria	Balneolaeota	Balneolia	Balneolales	Balneolaceae	Balneola	Balneola vulgaris
2	Bacteria	Balneolaeota	Balneolia	Balneolales	Balneolaceae		
2	Bacteria	Aquificae	Aquificae	Aquificales	Aquificaceae	Thermocrinis	
2	Bacteria	Aquificae	Aquificae	Aquificales	Aquificaceae	Aquifex	Aquifex aeolicus
2	Bacteria	Acidobacteria	unclassified Acidobacteria	unclassified Acidobacteria (miscellaneous)	Acidobacteria bacterium RIFCSPLOWO2_02_FULL_68_18		
2	Bacteria	Acidobacteria	unclassified Acidobacteria	unclassified Acidobacteria (miscellaneous)	Acidobacteria bacterium RIFCSPLOWO2_02_FULL_67_36		
2	Bacteria	Acidobacteria	unclassified Acidobacteria	unclassified Acidobacteria (miscellaneous)	Acidobacteria bacterium 13_2_20CM_57_17		
2	Bacteria	Acidobacteria	unclassified Acidobacteria	unclassified Acidobacteria (miscellaneous)	Acidobacteria bacterium 13_1_40CM_3_56_11		
2	Bacteria	Acidobacteria	unclassified Acidobacteria	unclassified Acidobacteria (miscellaneous)	Acidobacteria bacterium 13_1_40CM_2_68_5		
2	Bacteria	Acidobacteria	unclassified Acidobacteria	unclassified Acidobacteria (miscellaneous)	Acidobacteria bacterium 13_1_40CM_2_68_10		
2	Bacteria	Acidobacteria	unclassified Acidobacteria	unclassified Acidobacteria (miscellaneous)	Acidobacteria bacterium 13_1_40CM_2_64_6		
2	Bacteria	Acidobacteria	unclassified Acidobacteria	unclassified Acidobacteria (miscellaneous)	Acidobacteria bacterium 13_1_40CM_2_56_5		
2	Bacteria	Acidobacteria	unclassified Acidobacteria	unclassified Acidobacteria (miscellaneous)	Acidobacteria bacterium 13_1_20CM_2_68_7		
2	Bacteria	Acidobacteria	unclassified Acidobacteria	unclassified Acidobacteria (miscellaneous)	Acidobacteria bacterium 13_1_20CM_2_68_14		
2	Bacteria	Acidobacteria	unclassified Acidobacteria	unclassified Acidobacteria (miscellaneous)	Acidobacteria bacterium 13_1_20CM_2_60_10		
2	Bacteria	Acidobacteria	unclassified Acidobacteria				
2	Bacteria	Acidobacteria	Acidobacteriia	Acidobacteriales	Acidobacteriaceae	Acidobacterium	Acidobacterium ailaaui
2	Archaea	unclassified Archaea	unclassified Archaea (miscellaneous)	archaeon GW2011_AR11			
2	Archaea	TACK group	Crenarchaeota	Thermoprotei	Sulfolobales	Sulfolobaceae	Sulfolobus
2	Archaea	TACK group	Crenarchaeota	Thermoprotei	Sulfolobales	Sulfolobaceae	Acidianus
2	Archaea	TACK group	Candidatus Bathyarchaeota	unclassified Candidatus Bathyarchaeota	Candidatus Bathyarchaeota archaeon BA1		
2	Archaea	TACK group	Candidatus Bathyarchaeota	unclassified Candidatus Bathyarchaeota	Candidatus Bathyarchaeota archaeon B63		
2	Archaea	Euryarchaeota	Thermoplasmata	Thermoplasmatales	unclassified Thermoplasmatales	unclassified Thermoplasmatales (miscellaneous)	Thermoplasmatales archaeon SG8-52-4
2	Archaea	Euryarchaeota	Thermoplasmata	Thermoplasmatales	unclassified Thermoplasmatales	unclassified Thermoplasmatales (miscellaneous)	Thermoplasmatales archaeon SG8-52-2
2	Archaea	Euryarchaeota	Thermoplasmata	Methanomassiliicoccales	Methanomassiliicoccaceae	Candidatus Methanomethylophilus	Candidatus Methanomethylophilus sp. 1R26
2	Archaea	Euryarchaeota	Thermococci	Thermococcales	Thermococcaceae	Thermococcus	Thermococcus litoralis
2	Archaea	Euryarchaeota	Thermococci	Thermococcales	Thermococcaceae	Thermococcus	
2	Archaea	Euryarchaeota	Methanomicrobia	Methanosarcinales	Methanosarcinaceae	Methanosarcina	Methanosarcina sp. Ant1
2	[illegible]	[illegible]	[illegible]	[illegible]	[illegible]	[illegible]	[illegible]
[illegible]	[illegible]	[illegible]	[illegible]	[illegible]	[illegible]	[illegible]	[illegible]

1	Bacteria	Thermotogae	Thermotogae	Thermotogales	Thermotogaceae	Thermotoga	Thermotoga sp. Mc24
1	Bacteria	Thermotogae	Thermotogae	Thermotogales	Thermotogaceae	Thermotoga	Thermotoga sp. 50_64
1	Bacteria	Thermotogae	Thermotogae	Thermotogales	Thermotogaceae	Thermotoga	Thermotoga sp. 4484_232
1	Bacteria	Thermotogae	Thermotogae	Thermotogales	Thermotogaceae	Thermotoga	Thermotoga profunda
1	Bacteria	Thermotogae	Thermotogae	Thermotogales	Thermotogaceae	Thermotoga	Thermotoga caldifontis
1	Bacteria	Thermotogae	Thermotogae	Thermotogales	Thermotogaceae	Pseudothermotoga	Pseudothermotoga hypogea
1	Bacteria	Thermotogae	Thermotogae	Thermotogales	Thermotogaceae	Pseudothermotoga	
1	Bacteria	Thermotogae	Thermotogae	Thermotogales	Fervidobacteriaceae	Thermosipho	Thermosipho melanesiensis
1	Bacteria	Thermotogae	Thermotogae	Petrotogales	Petrotogaceae	Petrotoga	Petrotoga mobilis
1	Bacteria	Thermotogae	Thermotogae	Petrotogales	Petrotogaceae	Marinitoga	Marinitoga hydrogenitolerans
1	Bacteria	Thermotogae	Thermotogae				
1	Bacteria	Thermodesulfobacteria	Thermodesulfobacteria	Thermodesulfobacteriales	Thermodesulfobacteriaceae	Thermodesulfobacterium	Thermodesulfobacterium geofontis
1	Bacteria	Thermodesulfobacteria	Thermodesulfobacteria	Thermodesulfobacteriales	Thermodesulfobacteriaceae	Thermodesulfobacterium	Thermodesulfobacterium commune
1	Bacteria	Thermodesulfobacteria	Thermodesulfobacteria	Thermodesulfobacteriales	Thermodesulfobacteriaceae	Thermodesulfobacterium	
1	Bacteria	Thermodesulfobacteria	Thermodesulfobacteria	Thermodesulfobacteriales	Thermodesulfobacteriaceae	Thermodesulfatator	Thermodesulfatator autotrophicus
1	Bacteria	Terrabacteria group	Tenericutes	unclassified Tenericutes	Tenericutes bacterium 4572_104		
1	Bacteria	Terrabacteria group	Tenericutes	unclassified Tenericutes			
1	Bacteria	Terrabacteria group	Tenericutes	Mollicutes	Mycoplasmatales	Mycoplasmataceae	Ureaplasma
1	Bacteria	Terrabacteria group	Tenericutes	Mollicutes	Mycoplasmatales	Mycoplasmataceae	Ureaplasma
1	Bacteria	Terrabacteria group	Tenericutes	Mollicutes	Mycoplasmatales	Mycoplasmataceae	Mycoplasma
1	Bacteria	Terrabacteria group	Tenericutes	Mollicutes	Mycoplasmatales	Mycoplasmataceae	Mycoplasma
1	Bacteria	Terrabacteria group	Tenericutes	Mollicutes	Mycoplasmatales	Mycoplasmataceae	Mycoplasma
1	Bacteria	Terrabacteria group	Tenericutes	Mollicutes	Mycoplasmatales	Mycoplasmataceae	Mycoplasma
1	Bacteria	Terrabacteria group	Tenericutes	Mollicutes	Mycoplasmatales	Mycoplasmataceae	Mycoplasma
1	Bacteria	Terrabacteria group	Tenericutes	Mollicutes	Mycoplasmatales	Mycoplasmataceae	Mycoplasma
1	Bacteria	Terrabacteria group	Tenericutes	Mollicutes	Mycoplasmatales	Mycoplasmataceae	Mycoplasma
1	Bacteria	Terrabacteria group	Tenericutes	Mollicutes	Mycoplasmatales	Mycoplasmataceae	Mycoplasma
1	Bacteria	Terrabacteria group	Tenericutes	Mollicutes	Mycoplasmatales	Mycoplasmataceae	Mycoplasma
1	Bacteria	Terrabacteria group	Tenericutes	Mollicutes	Mycoplasmatales	Mycoplasmataceae	Mycoplasma
1	Bacteria	Terrabacteria group	Tenericutes	Mollicutes	Entomoplasmatales	Spiroplasmataceae	Spiroplasma
1	Bacteria	Terrabacteria group	Tenericutes	Mollicutes	Entomoplasmatales	Entomoplasmataceae	Entomoplasma
1	Bacteria	Terrabacteria group	Tenericutes	Mollicutes	Acholeplasmatales	Acholeplasmataceae	Candidatus Phytoplasma
1	Bacteria	Terrabacteria group	Tenericutes	Mollicutes	Acholeplasmatales	Acholeplasmataceae	Candidatus Phytoplasma
1	Bacteria	Terrabacteria group	Tenericutes	Mollicutes	Acholeplasmatales	Acholeplasmataceae	Candidatus Phytoplasma
1	Bacteria	Terrabacteria group	Tenericutes	Mollicutes	Acholeplasmatales	Acholeplasmataceae	Acholeplasma
1	Bacteria	Terrabacteria group	Tenericutes	Mollicutes	Acholeplasmatales	Acholeplasmataceae	Acholeplasma
1	Bacteria	Terrabacteria group	Tenericutes	Mollicutes	Acholeplasmatales	Acholeplasmataceae	Acholeplasma
1	Bacteria	Terrabacteria group	Firmicutes	unclassified Firmicutes sensu stricto	unclassified Firmicutes sensu stricto (miscellaneous)	Firmicutes bacterium M10-2	
1	Bacteria	Terrabacteria group	Firmicutes	unclassified Firmicutes sensu stricto	unclassified Firmicutes sensu stricto (miscellaneous)	Firmicutes bacterium CAG:552_39_19	
1	Bacteria	Terrabacteria group	Firmicutes	unclassified Firmicutes sensu stricto	unclassified Firmicutes sensu stricto (miscellaneous)	Firmicutes bacterium CAG:176_59_8	
1	Bacteria	Terrabacteria group	Firmicutes	unclassified Firmicutes sensu stricto	unclassified Firmicutes sensu stricto (miscellaneous)	Firmicutes bacterium CAG:129_59_24	
1	Bacteria	Terrabacteria group	Firmicutes	unclassified Firmicutes sensu stricto	Ndongobacter	Ndongobacter massiliensis	
1	Bacteria	Terrabacteria group	Firmicutes	environmental samples	uncultured firmicutes bacterium contig_61		
1	Bacteria	Terrabacteria group	Firmicutes	environmental samples	Firmicutes bacterium CAG:822		
1	Bacteria	Terrabacteria group	Firmicutes	environmental samples	Firmicutes bacterium CAG:582		
[illegible]	[illegible]	[illegible]	[illegible]	[illegible]	[illegible]	[illegible]	[illegible]

[illegible]	[illegible]	[illegible]	[illegible]	[illegible]	[illegible]	[illegible]	[illegible]

[illegible]	[illegible]	[illegible]	[illegible]	[illegible]	[illegible]	[illegible]	[illegible]

1	Bacteria	Terrabacteria group	Actinobacteria	Actinobacteria	Corynebacteriales	Mycobacteriaceae	Mycobacterium
1	Bacteria	Terrabacteria group	Actinobacteria	Actinobacteria	Corynebacteriales	Mycobacteriaceae	Mycobacterium
1	Bacteria	Terrabacteria group	Actinobacteria	Actinobacteria	Corynebacteriales	Mycobacteriaceae	Mycobacterium
1	Bacteria	Terrabacteria group	Actinobacteria	Actinobacteria	Corynebacteriales	Mycobacteriaceae	Mycobacterium
1	Bacteria	Terrabacteria group	Actinobacteria	Actinobacteria	Corynebacteriales	Mycobacteriaceae	Hoyosella
1	Bacteria	Terrabacteria group	Actinobacteria	Actinobacteria	Corynebacteriales	Mycobacteriaceae	Hoyosella
1	Bacteria	Terrabacteria group	Actinobacteria	Actinobacteria	Corynebacteriales	Gordoniaceae	Skermania
1	Bacteria	Terrabacteria group	Actinobacteria	Actinobacteria	Corynebacteriales	Gordoniaceae	Gordonia
1	Bacteria	Terrabacteria group	Actinobacteria	Actinobacteria	Corynebacteriales	Gordoniaceae	Gordonia
1	Bacteria	Terrabacteria group	Actinobacteria	Actinobacteria	Corynebacteriales	Gordoniaceae	Gordonia
1	Bacteria	Terrabacteria group	Actinobacteria	Actinobacteria	Corynebacteriales	Gordoniaceae	Gordonia
1	Bacteria	Terrabacteria group	Actinobacteria	Actinobacteria	Corynebacteriales	Gordoniaceae	Gordonia
1	Bacteria	Terrabacteria group	Actinobacteria	Actinobacteria	Corynebacteriales	Gordoniaceae	Gordonia
1	Bacteria	Terrabacteria group	Actinobacteria	Actinobacteria	Corynebacteriales	Dietziaceae	Dietzia
1	Bacteria	Terrabacteria group	Actinobacteria	Actinobacteria	Corynebacteriales	Corynebacteriaceae	unclassified Corynebacteriaceae
1	Bacteria	Terrabacteria group	Actinobacteria	Actinobacteria	Corynebacteriales	Corynebacteriaceae	Turicella
1	Bacteria	Terrabacteria group	Actinobacteria	Actinobacteria	Corynebacteriales	Corynebacteriaceae	Corynebacterium
1	Bacteria	Terrabacteria group	Actinobacteria	Actinobacteria	Corynebacteriales	Corynebacteriaceae	Corynebacterium
1	Bacteria	Terrabacteria group	Actinobacteria	Actinobacteria	Corynebacteriales	Corynebacteriaceae	Corynebacterium
1	Bacteria	Terrabacteria group	Actinobacteria	Actinobacteria	Corynebacteriales	Corynebacteriaceae	Corynebacterium
1	Bacteria	Terrabacteria group	Actinobacteria	Actinobacteria	Corynebacteriales	Corynebacteriaceae	Corynebacterium
1	Bacteria	Terrabacteria group	Actinobacteria	Actinobacteria	Corynebacteriales	Corynebacteriaceae	Corynebacterium
1	Bacteria	Terrabacteria group	Actinobacteria	Actinobacteria	Corynebacteriales	Corynebacteriaceae	Corynebacterium
1	Bacteria	Terrabacteria group	Actinobacteria	Actinobacteria	Corynebacteriales	Corynebacteriaceae	Corynebacterium
1	Bacteria	Terrabacteria group	Actinobacteria	Actinobacteria	Corynebacteriales	Corynebacteriaceae	Corynebacterium
1	Bacteria	Terrabacteria group	Actinobacteria	Actinobacteria	Corynebacteriales	Corynebacteriaceae	Corynebacterium
1	Bacteria	Terrabacteria group	Actinobacteria	Actinobacteria	Corynebacteriales	Corynebacteriaceae	Corynebacterium
1	Bacteria	Terrabacteria group	Actinobacteria	Actinobacteria	Corynebacteriales	Corynebacteriaceae	Corynebacterium
1	Bacteria	Terrabacteria group	Actinobacteria	Actinobacteria	Corynebacteriales	Corynebacteriaceae	Corynebacterium
1	Bacteria	Terrabacteria group	Actinobacteria	Actinobacteria	Corynebacteriales	Corynebacteriaceae	Corynebacterium
1	Bacteria	Terrabacteria group	Actinobacteria	Actinobacteria	Corynebacteriales	Corynebacteriaceae	Corynebacterium
1	Bacteria	Terrabacteria group	Actinobacteria	Actinobacteria	Corynebacteriales	Corynebacteriaceae	Corynebacterium
1	Bacteria	Terrabacteria group	Actinobacteria	Actinobacteria	Corynebacteriales	Corynebacteriaceae	Corynebacterium
1	Bacteria	Terrabacteria group	Actinobacteria	Actinobacteria	Corynebacteriales	Corynebacteriaceae	Corynebacterium
1	Bacteria	Terrabacteria group	Actinobacteria	Actinobacteria	Corynebacteriales	Corynebacteriaceae	Corynebacterium
1	Bacteria	Terrabacteria group	Actinobacteria	Actinobacteria	Corynebacteriales	Corynebacteriaceae	Corynebacterium
1	Bacteria	Terrabacteria group	Actinobacteria	Actinobacteria	Corynebacteriales	Corynebacteriaceae	Corynebacterium
1	Bacteria	Terrabacteria group	Actinobacteria	Actinobacteria	Corynebacteriales	Corynebacteriaceae	Corynebacterium
1	Bacteria	Terrabacteria group	Actinobacteria	Actinobacteria	Corynebacteriales	Corynebacteriaceae	Corynebacterium
1	Bacteria	Terrabacteria group	Actinobacteria	Actinobacteria	Corynebacteriales	Corynebacteriaceae	Corynebacterium
1	Bacteria	Terrabacteria group	Actinobacteria	Actinobacteria	Catenulisporales	Catenulisporaceae	Catenulispora
1	Bacteria	Terrabacteria group	Actinobacteria	Actinobacteria	Bifidobacteriales	Bifidobacteriaceae	Gardnerella
1	Bacteria	Terrabacteria group	Actinobacteria	Actinobacteria	Bifidobacteriales	Bifidobacteriaceae	Bifidobacterium
1	Bacteria	Terrabacteria group	Actinobacteria	Actinobacteria	Bifidobacteriales	Bifidobacteriaceae	Bifidobacterium
1	Bacteria	Terrabacteria group	Actinobacteria	Actinobacteria	Bifidobacteriales	Bifidobacteriaceae	Bifidobacterium
1	Bacteria	Terrabacteria group	Actinobacteria	Actinobacteria	Bifidobacteriales	Bifidobacteriaceae	Bifidobacterium
1	Bacteria	Terrabacteria group	Actinobacteria	Actinobacteria	Bifidobacteriales	Bifidobacteriaceae	Bifidobacterium
1	Bacteria	Terrabacteria group	Actinobacteria	Actinobacteria	Bifidobacteriales	Bifidobacteriaceae	Bifidobacterium
1	Bacteria	Terrabacteria group	Actinobacteria	Actinobacteria	Bifidobacteriales	Bifidobacteriaceae	Bifidobacterium
1	Bacteria	Terrabacteria group	Actinobacteria	Actinobacteria	Bifidobacteriales	Bifidobacteriaceae	Bifidobacterium
1	Bacteria	Terrabacteria group	Actinobacteria	Actinobacteria	Bifidobacteriales	Bifidobacteriaceae	Bifidobacterium
1	Bacteria	Terrabacteria group	Actinobacteria	Actinobacteria	Actinopolysporales	Actinopolysporaceae	Actinopolyspora
1	Bacteria	Terrabacteria group	Actinobacteria	Actinobacteria	Actinopolysporales	Actinopolysporaceae	Actinopolyspora
1	Bacteria	Terrabacteria group	Actinobacteria	Actinobacteria	Actinopolysporales	Actinopolysporaceae	Actinopolyspora
1	Bacteria	Terrabacteria group	Actinobacteria	Actinobacteria	Actinomycetales	unclassified Actinomycetales	unclassified Actinomycetales (miscellaneous)
1	Bacteria	Terrabacteria group	Actinobacteria	Actinobacteria	Actinomycetales	Actinomycetaceae	Trueperella
1	Bacteria	Terrabacteria group	Actinobacteria	Actinobacteria	Actinomycetales	Actinomycetaceae	Arcanobacterium
1	Bacteria	Terrabacteria group	Actinobacteria	Actinobacteria	Actinomycetales	Actinomycetaceae	Actinomyces
1	Bacteria	Terrabacteria group	Actinobacteria	Actinobacteria	Actinomycetales	Actinomycetaceae	Actinomyces
1	Bacteria	Terrabacteria group	Actinobacteria	Actinobacteria	Actinomycetales	Actinomycetaceae	Actinomyces
1	Bacteria	Terrabacteria group	Actinobacteria	Actinobacteria	Actinomycetales	Actinomycetaceae	Actinomyces
1	Bacteria	Terrabacteria group	Actinobacteria	Actinobacteria	Actinomycetales	Actinomycetaceae	Actinomyces
1	Bacteria	Terrabacteria group	Actinobacteria	Actinobacteria	Actinomycetales	Actinomycetaceae	Actinomyces
1	Bacteria	Terrabacteria group	Actinobacteria	Actinobacteria	Actinomycetales	Actinomycetaceae	Actinomyces
1	Bacteria	Terrabacteria group	Actinobacteria	Actinobacteria	Actinomycetales	Actinomycetaceae	Actinobaculum
1	Bacteria	Terrabacteria group	Actinobacteria	Actinobacteria	Actinomycetales	Actinomycetaceae	Actinobaculum
1	Bacteria	Terrabacteria group	Actinobacteria	Actinobacteria	Actinobacteria incertae sedis	Thermobispora	Thermobispora bispora
1	Bacteria	Terrabacteria group	Actinobacteria	Acidimicrobiia	unclassified Acidimicrobiia	Acidimicrobiia bacterium BACL6 MAG-120910-bin40	
1	Bacteria	Synergistetes	Synergistia	Synergistales	unclassified Synergistales	Synergistales bacterium 57_84	
1	Bacteria	Synergistetes	Synergistia	Synergistales	Synergistaceae	Dethiosulfovibrio	
1	Bacteria	Synergistetes	Synergistia	Synergistales	Synergistaceae	Cloacibacillus	
1	Bacteria	Synergistetes	Synergistia	Synergistales	Synergistaceae	Acetomicrobium	Acetomicrobium hydrogeniformans
1	Bacteria	Spirochaetes	unclassified Spirochaetes	Spirochaetes bacterium GWB1_66_5			
1	Bacteria	Spirochaetes	Spirochaetia	Spirochaetales	Spirochaetaceae	Treponema	Treponema sp. RIFOXYC1_FULL_61_9
1	Bacteria	Spirochaetes	Spirochaetia	Spirochaetales	Spirochaetaceae	Treponema	Treponema sp. GWC1_61_84
1	Bacteria	Spirochaetes	Spirochaetia	Spirochaetales	Spirochaetaceae	Treponema	Treponema sp. GWA1_62_8
1	Bacteria	Spirochaetes	Spirochaetia	Spirochaetales	Spirochaetaceae	Treponema	Treponema sp. C6A8
1	Bacteria	Spirochaetes	Spirochaetia	Spirochaetales	Spirochaetaceae	Treponema	Treponema endosymbiont of Eucomonympha sp.
1	Bacteria	Spirochaetes	Spirochaetia	Spirochaetales	Spirochaetaceae	Treponema	Treponema berlinense
1	Bacteria	Spirochaetes	Spirochaetia	Spirochaetales	Spirochaetaceae	Candidatus Marispirochaeta	
1	Bacteria	Spirochaetes	Spirochaetia	Spirochaetales	Borreliaceae	Borreliella	Borreliella burgdorferi
1	Bacteria	Spirochaetes	Spirochaetia	Leptospirales	Leptospiraceae	Turneriella	Turneriella parva
1	Bacteria	Spirochaetes	Spirochaetia	Leptospirales	Leptospiraceae	Leptospira	Leptospira wolffii
1	Bacteria	Spirochaetes	Spirochaetia	Leptospirales	Leptospiraceae	Leptospira	Leptospira terpstrae
1	Bacteria	Spirochaetes	Spirochaetia	Leptospirales	Leptospiraceae	Leptospira	Leptospira sp. GIMC2001:Bairam-Ali
1	Bacteria	Spirochaetes	Spirochaetia	Leptospirales	Leptospiraceae	Leptospira	Leptospira santarosai
1	Bacteria	Spirochaetes	Spirochaetia	Leptospirales	Leptospiraceae	Leptospira	Leptospira fainei
1	Bacteria	Spirochaetes	Spirochaetia	Leptospirales	Leptospiraceae	Leptospira	Leptospira broomii
1	Bacteria	Spirochaetes	Spirochaetia	Leptospirales	Leptospiraceae	Leptospira	Leptospira alstonii
1	Bacteria	Spirochaetes	Spirochaetia	Brachyspirales	Brachyspiraceae	Brachyspira	environmental samples
1	Bacteria	Spirochaetes	Spirochaetia	Brachyspirales	Brachyspiraceae	Brachyspira	Brachyspira suanatina
1	Bacteria	Spirochaetes	Spirochaetia	Brachyspirales	Brachyspiraceae	Brachyspira	Brachyspira murdochii
1	Bacteria	Proteobacteria	environmental samples	uncultured proteobacterium DelRiverFos13D03			
1	Bacteria	Proteobacteria	environmental samples	uncultured proteobacterium DelRiverFos06H03			
1	Bacteria	Proteobacteria	environmental samples	uncultured marine proteobacterium			
1	Bacteria	Proteobacteria	delta/epsilon subdivisions	Epsilonproteobacteria	unclassified Epsilonproteobacteria	Sulfurovum	Sulfurovum sp. FS06-10
1	Bacteria	Proteobacteria	delta/epsilon subdivisions	Epsilonproteobacteria	unclassified Epsilonproteobacteria	Sulfurovum	Sulfurovum lithotrophicum
1	Bacteria	Proteobacteria	delta/epsilon subdivisions	Epsilonproteobacteria	unclassified Epsilonproteobacteria	Nitratiruptor	
1	Bacteria	Proteobacteria	delta/epsilon subdivisions	Epsilonproteobacteria	Nautiliales	Nautiliaceae	Caminibacter
1	Bacteria	Proteobacteria	delta/epsilon subdivisions	Epsilonproteobacteria	Nautiliales	Nautiliaceae	Caminibacter
1	Bacteria	Proteobacteria	delta/epsilon subdivisions	Epsilonproteobacteria	Campylobacterales	Hydrogenimonaceae	Hydrogenimonas
1	Bacteria	Proteobacteria	delta/epsilon subdivisions	Epsilonproteobacteria	Campylobacterales	Helicobacteraceae	unclassified Helicobacteraceae
1	Bacteria	Proteobacteria	delta/epsilon subdivisions	Epsilonproteobacteria	Campylobacterales	Helicobacteraceae	Sulfurimonas
1	Bacteria	Proteobacteria	delta/epsilon subdivisions	Epsilonproteobacteria	Campylobacterales	Helicobacteraceae	Sulfurimonas
1	Bacteria	Proteobacteria	delta/epsilon subdivisions	Epsilonproteobacteria	Campylobacterales	Helicobacteraceae	Sulfuricurvum
1	Bacteria	Proteobacteria	delta/epsilon subdivisions	Epsilonproteobacteria	Campylobacterales	Helicobacteraceae	Sulfuricurvum
1	Bacteria	Proteobacteria	delta/epsilon subdivisions	Epsilonproteobacteria	Campylobacterales	Helicobacteraceae	Sulfuricurvum
1	Bacteria	Proteobacteria	delta/epsilon subdivisions	Epsilonproteobacteria	Campylobacterales	Helicobacteraceae	Helicobacter
1	Bacteria	Proteobacteria	delta/epsilon subdivisions	Epsilonproteobacteria	Campylobacterales	Helicobacteraceae	Helicobacter
1	Bacteria	Proteobacteria	delta/epsilon subdivisions	Epsilonproteobacteria	Campylobacterales	Helicobacteraceae	Helicobacter
1	Bacteria	Proteobacteria	delta/epsilon subdivisions	Epsilonproteobacteria	Campylobacterales	Helicobacteraceae	Helicobacter
1	Bacteria	Proteobacteria	delta/epsilon subdivisions	Epsilonproteobacteria	Campylobacterales	Helicobacteraceae	Helicobacter
1	Bacteria	Proteobacteria	delta/epsilon subdivisions	Epsilonproteobacteria	Campylobacterales	Helicobacteraceae	Helicobacter
1	Bacteria	Proteobacteria	delta/epsilon subdivisions	Epsilonproteobacteria	Campylobacterales	Helicobacteraceae	Helicobacter
1	Bacteria	Proteobacteria	delta/epsilon subdivisions	Epsilonproteobacteria	Campylobacterales	Helicobacteraceae	Helicobacter
1	Bacteria	Proteobacteria	delta/epsilon subdivisions	Epsilonproteobacteria	Campylobacterales	Helicobacteraceae	Helicobacter
1	Bacteria	Proteobacteria	delta/epsilon subdivisions	Epsilonproteobacteria	Campylobacterales	Helicobacteraceae	Helicobacter
1	Bacteria	Proteobacteria	delta/epsilon subdivisions	Epsilonproteobacteria	Campylobacterales	Helicobacteraceae	Helicobacter
1	Bacteria	Proteobacteria	delta/epsilon subdivisions	Epsilonproteobacteria	Campylobacterales	Helicobacteraceae	Helicobacter
1	Bacteria	Proteobacteria	delta/epsilon subdivisions	Epsilonproteobacteria	Campylobacterales	Helicobacteraceae	Helicobacter
1	Bacteria	Proteobacteria	delta/epsilon subdivisions	Epsilonproteobacteria	Campylobacterales	Helicobacteraceae	Helicobacter
1	Bacteria	Proteobacteria	delta/epsilon subdivisions	Epsilonproteobacteria	Campylobacterales	Helicobacteraceae	Helicobacter
1	Bacteria	Proteobacteria	delta/epsilon subdivisions	Epsilonproteobacteria	Campylobacterales	Helicobacteraceae	Helicobacter
1	Bacteria	Proteobacteria	delta/epsilon subdivisions	Epsilonproteobacteria	Campylobacterales	Helicobacteraceae	
1	Bacteria	Proteobacteria	delta/epsilon subdivisions	Epsilonproteobacteria	Campylobacterales	Campylobacteraceae	unclassified Campylobacteraceae
1	Bacteria	Proteobacteria	delta/epsilon subdivisions	Epsilonproteobacteria	Campylobacterales	Campylobacteraceae	Sulfurospirillum
1	Bacteria	Proteobacteria	delta/epsilon subdivisions	Epsilonproteobacteria	Campylobacterales	Campylobacteraceae	Sulfurospirillum
1	Bacteria	Proteobacteria	delta/epsilon subdivisions	Epsilonproteobacteria	Campylobacterales	Campylobacteraceae	Sulfurospirillum
1	Bacteria	Proteobacteria	delta/epsilon subdivisions	Epsilonproteobacteria	Campylobacterales	Campylobacteraceae	Sulfurospirillum
1	Bacteria	Proteobacteria	delta/epsilon subdivisions	Epsilonproteobacteria	Campylobacterales	Campylobacteraceae	Campylobacter
1	Bacteria	Proteobacteria	delta/epsilon subdivisions	Epsilonproteobacteria	Campylobacterales	Campylobacteraceae	Campylobacter
1	Bacteria	Proteobacteria	delta/epsilon subdivisions	Epsilonproteobacteria	Campylobacterales	Campylobacteraceae	Campylobacter
1	Bacteria	Proteobacteria	delta/epsilon subdivisions	Epsilonproteobacteria	Campylobacterales	Campylobacteraceae	Campylobacter
1	Bacteria	Proteobacteria	delta/epsilon subdivisions	Epsilonproteobacteria	Campylobacterales	Campylobacteraceae	Campylobacter
1	Bacteria	Proteobacteria	delta/epsilon subdivisions	Epsilonproteobacteria	Campylobacterales	Campylobacteraceae	Campylobacter
1	Bacteria	Proteobacteria	delta/epsilon subdivisions	Epsilonproteobacteria	Campylobacterales	Campylobacteraceae	Campylobacter
1	Bacteria	Proteobacteria	delta/epsilon subdivisions	Epsilonproteobacteria	Campylobacterales	Campylobacteraceae	Arcobacter
1	Bacteria	Proteobacteria	delta/epsilon subdivisions	Epsilonproteobacteria	Campylobacterales	Campylobacteraceae	Arcobacter
1	Bacteria	Proteobacteria	delta/epsilon subdivisions	Epsilonproteobacteria	Campylobacterales	Campylobacteraceae	Arcobacter
1	Bacteria	Proteobacteria	delta/epsilon subdivisions	Epsilonproteobacteria	Campylobacterales	Campylobacteraceae	Arcobacter
1	Bacteria	Proteobacteria	delta/epsilon subdivisions	Epsilonproteobacteria	Campylobacterales	Campylobacteraceae	Arcobacter
1	Bacteria	Proteobacteria	delta/epsilon subdivisions	Epsilonproteobacteria	Campylobacterales	Campylobacteraceae	
1	Bacteria	Proteobacteria	delta/epsilon subdivisions	Deltaproteobacteria	unclassified Deltaproteobacteria	unclassified Deltaproteobacteria (miscellaneous)	delta proteobacterium PSCGC 5296
1	Bacteria	Proteobacteria	delta/epsilon subdivisions	Deltaproteobacteria	unclassified Deltaproteobacteria	unclassified Deltaproteobacteria (miscellaneous)	Deltaproteobacteria bacterium RIFOXYA12_FULL_58_15
1	Bacteria	Proteobacteria	delta/epsilon subdivisions	Deltaproteobacteria	unclassified Deltaproteobacteria	unclassified Deltaproteobacteria (miscellaneous)	Deltaproteobacteria bacterium RIFCSPLOWO2_02_FULL_44_10
1	Bacteria	Proteobacteria	delta/epsilon subdivisions	Deltaproteobacteria	unclassified Deltaproteobacteria	unclassified Deltaproteobacteria (miscellaneous)	Deltaproteobacteria bacterium RIFCSPHIGHO2_02_FULL_60_17
1	Bacteria	Proteobacteria	delta/epsilon subdivisions	Deltaproteobacteria	unclassified Deltaproteobacteria	unclassified Deltaproteobacteria (miscellaneous)	Deltaproteobacteria bacterium RIFCSPHIGHO2_02_FULL_44_16
1	Bacteria	Proteobacteria	delta/epsilon subdivisions	Deltaproteobacteria	unclassified Deltaproteobacteria	unclassified Deltaproteobacteria (miscellaneous)	Deltaproteobacteria bacterium RBG_19FT_COMBO_58_16
1	Bacteria	Proteobacteria	delta/epsilon subdivisions	Deltaproteobacteria	unclassified Deltaproteobacteria	unclassified Deltaproteobacteria (miscellaneous)	Deltaproteobacteria bacterium RBG_19FT_COMBO_56_10
1	Bacteria	Proteobacteria	delta/epsilon subdivisions	Deltaproteobacteria	unclassified Deltaproteobacteria	unclassified Deltaproteobacteria (miscellaneous)	Deltaproteobacteria bacterium RBG_19FT_COMBO_46_12
1	Bacteria	Proteobacteria	delta/epsilon subdivisions	Deltaproteobacteria	unclassified Deltaproteobacteria	unclassified Deltaproteobacteria (miscellaneous)	Deltaproteobacteria bacterium RBG_19FT_COMBO_43_11
1	Bacteria	Proteobacteria	delta/epsilon subdivisions	Deltaproteobacteria	unclassified Deltaproteobacteria	unclassified Deltaproteobacteria (miscellaneous)	Deltaproteobacteria bacterium RBG_16_58_17
1	Bacteria	Proteobacteria	delta/epsilon subdivisions	Deltaproteobacteria	unclassified Deltaproteobacteria	unclassified Deltaproteobacteria (miscellaneous)	Deltaproteobacteria bacterium RBG_16_55_12
1	Bacteria	Proteobacteria	delta/epsilon subdivisions	Deltaproteobacteria	unclassified Deltaproteobacteria	unclassified Deltaproteobacteria (miscellaneous)	Deltaproteobacteria bacterium RBG_16_54_11
1	Bacteria	Proteobacteria	delta/epsilon subdivisions	Deltaproteobacteria	unclassified Deltaproteobacteria	unclassified Deltaproteobacteria (miscellaneous)	Deltaproteobacteria bacterium GWA2_57_13
1	Bacteria	Proteobacteria	delta/epsilon subdivisions	Deltaproteobacteria	unclassified Deltaproteobacteria	unclassified Deltaproteobacteria (miscellaneous)	Deltaproteobacteria bacterium DG_8
1	Bacteria	Proteobacteria	delta/epsilon subdivisions	Deltaproteobacteria	unclassified Deltaproteobacteria	unclassified Deltaproteobacteria (miscellaneous)	Deltaproteobacteria bacterium CG1_02_45_11
1	Bacteria	Proteobacteria	delta/epsilon subdivisions	Deltaproteobacteria	unclassified Deltaproteobacteria	unclassified Deltaproteobacteria (miscellaneous)	Deltaproteobacteria bacterium 13_1_40CM_3_71_4
1	Bacteria	Proteobacteria	delta/epsilon subdivisions	Deltaproteobacteria	unclassified Deltaproteobacteria	unclassified Deltaproteobacteria (miscellaneous)	Deltaproteobacteria bacterium 13_1_40CM_3_69_14
1	Bacteria	Proteobacteria	delta/epsilon subdivisions	Deltaproteobacteria	unclassified Deltaproteobacteria	unclassified Deltaproteobacteria (miscellaneous)	Deltaproteobacteria bacterium 13_1_20CM_2_69_21
1	Bacteria	Proteobacteria	delta/epsilon subdivisions	Deltaproteobacteria	environmental samples	uncultured delta proteobacterium	
1	Bacteria	Proteobacteria	delta/epsilon subdivisions	Deltaproteobacteria	Syntrophobacterales	Syntrophobacteraceae	
1	Bacteria	Proteobacteria	delta/epsilon subdivisions	Deltaproteobacteria	Syntrophobacterales	Syntrophaceae	Smithella
1	Bacteria	Proteobacteria	delta/epsilon subdivisions	Deltaproteobacteria	Syntrophobacterales	Syntrophaceae	Smithella
1	Bacteria	Proteobacteria	delta/epsilon subdivisions	Deltaproteobacteria	Syntrophobacterales	Syntrophaceae	Smithella
1	Bacteria	Proteobacteria	delta/epsilon subdivisions	Deltaproteobacteria	Syntrophobacterales	Syntrophaceae	Desulfobacca
1	Bacteria	Proteobacteria	delta/epsilon subdivisions	Deltaproteobacteria	Myxococcales	unclassified Myxococcales	unclassified Myxococcales (miscellaneous)
1	Bacteria	Proteobacteria	delta/epsilon subdivisions	Deltaproteobacteria	Myxococcales	environmental samples	uncultured myxobacterium HF0200_08J13
1	Bacteria	Proteobacteria	delta/epsilon subdivisions	Deltaproteobacteria	Myxococcales	environmental samples	uncultured myxobacterium HF0200_01L06
1	Bacteria	Proteobacteria	delta/epsilon subdivisions	Deltaproteobacteria	Myxococcales	Cystobacterineae	Archangiaceae
1	Bacteria	Proteobacteria	delta/epsilon subdivisions	Deltaproteobacteria	Myxococcales	Cystobacterineae	Archangiaceae
1	Bacteria	Proteobacteria	delta/epsilon subdivisions	Deltaproteobacteria	Myxococcales	Cystobacterineae	Archangiaceae
1	Bacteria	Proteobacteria	delta/epsilon subdivisions	Deltaproteobacteria	Desulfuromonadales	unclassified Desulfuromonadales	Desulfuromonadales bacterium C00003094
1	Bacteria	Proteobacteria	delta/epsilon subdivisions	Deltaproteobacteria	Desulfuromonadales	Geobacteraceae	Geobacter
1	Bacteria	Proteobacteria	delta/epsilon subdivisions	Deltaproteobacteria	Desulfuromonadales	Geobacteraceae	Geobacter
1	Bacteria	Proteobacteria	delta/epsilon subdivisions	Deltaproteobacteria	Desulfuromonadales	Geobacteraceae	Geobacter
1	Bacteria	Proteobacteria	delta/epsilon subdivisions	Deltaproteobacteria	Desulfuromonadales	Desulfuromonadaceae	unclassified Desulfuromonadaceae
1	Bacteria	Proteobacteria	delta/epsilon subdivisions	Deltaproteobacteria	Desulfurellales	Desulfurellaceae	Hippea
1	Bacteria	Proteobacteria	delta/epsilon subdivisions	Deltaproteobacteria	Desulfurellales	Desulfurellaceae	Hippea
1	Bacteria	Proteobacteria	delta/epsilon subdivisions	Deltaproteobacteria	Desulfurellales	Desulfurellaceae	Desulfurella
1	Bacteria	Proteobacteria	delta/epsilon subdivisions	Deltaproteobacteria	Desulfovibrionales	Desulfovibrionaceae	Halodesulfovibrio
1	Bacteria	Proteobacteria	delta/epsilon subdivisions	Deltaproteobacteria	Desulfovibrionales	Desulfovibrionaceae	Desulfovibrio
1	Bacteria	Proteobacteria	delta/epsilon subdivisions	Deltaproteobacteria	Desulfovibrionales	Desulfohalobiaceae	Desulfonatronovibrio
1	Bacteria	Proteobacteria	delta/epsilon subdivisions	Deltaproteobacteria	Desulfobacterales	unclassified Desulfobacterales	Desulfobacterales bacterium RIFOXYA12_FULL_46_15
1	Bacteria	Proteobacteria	delta/epsilon subdivisions	Deltaproteobacteria	Desulfobacterales	Desulfobulbaceae	unclassified Desulfobulbaceae
1	Bacteria	Proteobacteria	delta/epsilon subdivisions	Deltaproteobacteria	Desulfobacterales	Desulfobulbaceae	Desulfopila
1	Bacteria	Proteobacteria	delta/epsilon subdivisions	Deltaproteobacteria	Desulfobacterales	Desulfobulbaceae	Desulfobulbus
1	Bacteria	Proteobacteria	delta/epsilon subdivisions	Deltaproteobacteria	Desulfobacterales	Desulfobulbaceae	
1	Bacteria	Proteobacteria	delta/epsilon subdivisions	Deltaproteobacteria	Desulfobacterales	Desulfobacteraceae	unclassified Desulfobacteraceae
1	Bacteria	Proteobacteria	delta/epsilon subdivisions	Deltaproteobacteria	Desulfobacterales	Desulfobacteraceae	Desulfobacula
1	Bacteria	Proteobacteria	delta/epsilon subdivisions	Deltaproteobacteria	Desulfobacterales	Desulfobacteraceae	Desulfobacula
1	Bacteria	Proteobacteria	delta/epsilon subdivisions	Deltaproteobacteria	Desulfobacterales	Desulfobacteraceae	Desulfobacter
1	Bacteria	Proteobacteria	delta/epsilon subdivisions	Deltaproteobacteria	Desulfarculales	Desulfarculaceae	
1	Bacteria	Proteobacteria	delta/epsilon subdivisions	Deltaproteobacteria	Bdellovibrionales	unclassified Bdellovibrionales	Bdellovibrionales bacterium RIFOXYD1_FULL_55_31
1	Bacteria	Proteobacteria	delta/epsilon subdivisions	Deltaproteobacteria	Bdellovibrionales	unclassified Bdellovibrionales	Bdellovibrionales bacterium RIFOXYB2_FULL_36_6
1	Bacteria	Proteobacteria	delta/epsilon subdivisions	Deltaproteobacteria	Bdellovibrionales	unclassified Bdellovibrionales	Bdellovibrionales bacterium RIFCSPHIGHO2_01_FULL_40_29
1	Bacteria	Proteobacteria	delta/epsilon subdivisions	Deltaproteobacteria	Bdellovibrionales	unclassified Bdellovibrionales	Bdellovibrionales bacterium GWB1_55_8
1	Bacteria	Proteobacteria	delta/epsilon subdivisions	Deltaproteobacteria	Bdellovibrionales	unclassified Bdellovibrionales	Bdellovibrionales bacterium GWA2_49_15
1	Bacteria	Proteobacteria	delta/epsilon subdivisions	Deltaproteobacteria	Bdellovibrionales	unclassified Bdellovibrionales	Bdellovibrionales bacterium GWA1_52_35
1	Bacteria	Proteobacteria	delta/epsilon subdivisions	Deltaproteobacteria	Bdellovibrionales	Bdellovibrionaceae	Bdellovibrio
1	Bacteria	Proteobacteria	delta/epsilon subdivisions	Deltaproteobacteria	Bdellovibrionales	Bacteriovoracaceae	Bacteriovorax
1	Bacteria	Proteobacteria	delta/epsilon subdivisions	Deltaproteobacteria	Bdellovibrionales	Bacteriovoracaceae	Bacteriovorax
1	Bacteria	Proteobacteria	delta/epsilon subdivisions				
1	Bacteria	Proteobacteria	Zetaproteobacteria	unclassified Zetaproteobacteria			
1	Bacteria	Proteobacteria	Zetaproteobacteria	Mariprofundales	Mariprofundaceae	Mariprofundus	
1	Bacteria	Proteobacteria	Zetaproteobacteria				
1	Bacteria	Proteobacteria	Gammaproteobacteria	unclassified Gammaproteobacteria	unclassified Gammaproteobacteria (miscellaneous)	Gammaproteobacteria bacterium RIFOXYB2_FULL_38_6	
1	Bacteria	Proteobacteria	Gammaproteobacteria	unclassified Gammaproteobacteria	unclassified Gammaproteobacteria (miscellaneous)	Gammaproteobacteria bacterium RIFOXYA12_FULL_61_12	
1	Bacteria	Proteobacteria	Gammaproteobacteria	unclassified Gammaproteobacteria	unclassified Gammaproteobacteria (miscellaneous)	Gammaproteobacteria bacterium RIFCSPHIGHO2_12_FULL_45_9	
1	Bacteria	Proteobacteria	Gammaproteobacteria	unclassified Gammaproteobacteria	unclassified Gammaproteobacteria (miscellaneous)	Gammaproteobacteria bacterium RIFCSPHIGHO2_12_FULL_45_12	
1	Bacteria	Proteobacteria	Gammaproteobacteria	unclassified Gammaproteobacteria	unclassified Gammaproteobacteria (miscellaneous)	Gammaproteobacteria bacterium RIFCSPHIGHO2_12_FULL_39_24	
1	Bacteria	Proteobacteria	Gammaproteobacteria	unclassified Gammaproteobacteria	unclassified Gammaproteobacteria (miscellaneous)	Gammaproteobacteria bacterium RIFCSPHIGHO2_12_FULL_38_11	
1	Bacteria	Proteobacteria	Gammaproteobacteria	unclassified Gammaproteobacteria	unclassified Gammaproteobacteria (miscellaneous)	Gammaproteobacteria bacterium RIFCSPHIGHO2_12_FULL_37_34	
1	Bacteria	Proteobacteria	Gammaproteobacteria	unclassified Gammaproteobacteria	unclassified Gammaproteobacteria (miscellaneous)	Gammaproteobacteria bacterium RIFCSPHIGHO2_02_FULL_42_13	
1	Bacteria	Proteobacteria	Gammaproteobacteria	unclassified Gammaproteobacteria	unclassified Gammaproteobacteria (miscellaneous)	Gammaproteobacteria bacterium GWF2_41_13	
1	Bacteria	Proteobacteria	Gammaproteobacteria	unclassified Gammaproteobacteria	unclassified Gammaproteobacteria (miscellaneous)	Gammaproteobacteria bacterium GWE2_42_36	
1	Bacteria	Proteobacteria	Gammaproteobacteria	unclassified Gammaproteobacteria	unclassified Gammaproteobacteria (miscellaneous)	Gammaproteobacteria bacterium 2W06	
1	Bacteria	Proteobacteria	Gammaproteobacteria	unclassified Gammaproteobacteria	sulfur-oxidizing symbionts	endosymbiont of Riftia pachyptila	
1	Bacteria	Proteobacteria	Gammaproteobacteria	unclassified Gammaproteobacteria	sulfur-oxidizing symbionts	Solemya pervernicosa gill symbiont	
1	Bacteria	Proteobacteria	Gammaproteobacteria	unclassified Gammaproteobacteria	sulfur-oxidizing symbionts	Bathymodiolus septemdierum thioautotrophic gill symbiont	
1	Bacteria	Proteobacteria	Gammaproteobacteria	unclassified Gammaproteobacteria	SAR86 cluster	SAR86 cluster bacterium BACL1 MAG-120920-bin57	
1	Bacteria	Proteobacteria	Gammaproteobacteria	unclassified Gammaproteobacteria	Candidatus Thioglobus	Candidatus Thioglobus autotrophicus	
1	Bacteria	Proteobacteria	Gammaproteobacteria	unclassified Gammaproteobacteria	Candidatus Thioglobus		
1	Bacteria	Proteobacteria	Gammaproteobacteria	environmental samples	uncultured gamma proteobacterium Rifle_16ft_4_minimus_1061		
1	Bacteria	Proteobacteria	Gammaproteobacteria	environmental samples	uncultured gamma proteobacterium HF0130_25M15		
1	Bacteria	Proteobacteria	Gammaproteobacteria	environmental samples	uncultured gamma proteobacterium HF0010_05D02		
1	Bacteria	Proteobacteria	Gammaproteobacteria	Xanthomonadales	unclassified Xanthomonadales	Xanthomonadales bacterium RIFOXYA1_FULL_69_10	
1	Bacteria	Proteobacteria	Gammaproteobacteria	Xanthomonadales	unclassified Xanthomonadales	Xanthomonadales bacterium GWF1_69_6	
1	Bacteria	Proteobacteria	Gammaproteobacteria	Xanthomonadales	Xanthomonadaceae	unclassified Xanthomonadaceae	unclassified Xanthomonadaceae (miscellaneous)
1	Bacteria	Proteobacteria	Gammaproteobacteria	Xanthomonadales	Xanthomonadaceae	Xylella	
1	Bacteria	Proteobacteria	Gammaproteobacteria	Xanthomonadales	Xanthomonadaceae	Xanthomonas	Xanthomonas vesicatoria
1	Bacteria	Proteobacteria	Gammaproteobacteria	Xanthomonadales	Xanthomonadaceae	Xanthomonas	Xanthomonas translucens
1	Bacteria	Proteobacteria	Gammaproteobacteria	Xanthomonadales	Xanthomonadaceae	Xanthomonas	Xanthomonas sp. NCPPB1128
1	Bacteria	Proteobacteria	Gammaproteobacteria	Xanthomonadales	Xanthomonadaceae	Xanthomonas	Xanthomonas sp. MUS 060
1	Bacteria	Proteobacteria	Gammaproteobacteria	Xanthomonadales	Xanthomonadaceae	Xanthomonas	Xanthomonas sp. Leaf148
1	Bacteria	Proteobacteria	Gammaproteobacteria	Xanthomonadales	Xanthomonadaceae	Xanthomonas	Xanthomonas sp. Leaf131
1	Bacteria	Proteobacteria	Gammaproteobacteria	Xanthomonadales	Xanthomonadaceae	Xanthomonas	Xanthomonas pisi
1	Bacteria	Proteobacteria	Gammaproteobacteria	Xanthomonadales	Xanthomonadaceae	Xanthomonas	Xanthomonas perforans
1	Bacteria	Proteobacteria	Gammaproteobacteria	Xanthomonadales	Xanthomonadaceae	Xanthomonas	Xanthomonas oryzae
1	Bacteria	Proteobacteria	Gammaproteobacteria	Xanthomonadales	Xanthomonadaceae	Xanthomonas	Xanthomonas oryzae
1	Bacteria	Proteobacteria	Gammaproteobacteria	Xanthomonadales	Xanthomonadaceae	Xanthomonas	Xanthomonas maliensis
1	Bacteria	Proteobacteria	Gammaproteobacteria	Xanthomonadales	Xanthomonadaceae	Xanthomonas	Xanthomonas hyacinthi
1	Bacteria	Proteobacteria	Gammaproteobacteria	Xanthomonadales	Xanthomonadaceae	Xanthomonas	Xanthomonas hortorum
1	Bacteria	Proteobacteria	Gammaproteobacteria	Xanthomonadales	Xanthomonadaceae	Xanthomonas	Xanthomonas gardneri
1	Bacteria	Proteobacteria	Gammaproteobacteria	Xanthomonadales	Xanthomonadaceae	Xanthomonas	Xanthomonas citri group
1	Bacteria	Proteobacteria	Gammaproteobacteria	Xanthomonadales	Xanthomonadaceae	Xanthomonas	Xanthomonas cassavae
1	Bacteria	Proteobacteria	Gammaproteobacteria	Xanthomonadales	Xanthomonadaceae	Xanthomonas	Xanthomonas axonopodis
1	Bacteria	Proteobacteria	Gammaproteobacteria	Xanthomonadales	Xanthomonadaceae	Xanthomonas	Xanthomonas arboricola
1	Bacteria	Proteobacteria	Gammaproteobacteria	Xanthomonadales	Xanthomonadaceae	Stenotrophomonas	Stenotrophomonas sp. TD3
1	Bacteria	Proteobacteria	Gammaproteobacteria	Xanthomonadales	Xanthomonadaceae	Stenotrophomonas	Stenotrophomonas sp. RIT309
1	Bacteria	Proteobacteria	Gammaproteobacteria	Xanthomonadales	Xanthomonadaceae	Stenotrophomonas	Stenotrophomonas sp. Leaf70
1	Bacteria	Proteobacteria	Gammaproteobacteria	Xanthomonadales	Xanthomonadaceae	Stenotrophomonas	Stenotrophomonas nitritireducens
1	Bacteria	Proteobacteria	Gammaproteobacteria	Xanthomonadales	Xanthomonadaceae	Pseudoxanthomonas	Pseudoxanthomonas sp. YR558
1	Bacteria	Proteobacteria	Gammaproteobacteria	Xanthomonadales	Xanthomonadaceae	Pseudoxanthomonas	Pseudoxanthomonas sp. J35
1	Bacteria	Proteobacteria	Gammaproteobacteria	Xanthomonadales	Xanthomonadaceae	Lysobacter	Lysobacter sp. Root667
1	Bacteria	Proteobacteria	Gammaproteobacteria	Xanthomonadales	Xanthomonadaceae	Arenimonas	Arenimonas malthae
1	Bacteria	Proteobacteria	Gammaproteobacteria	Xanthomonadales	Rhodanobacteraceae	Rhodanobacter	Rhodanobacter spathiphylli
1	Bacteria	Proteobacteria	Gammaproteobacteria	Xanthomonadales	Rhodanobacteraceae	Rhodanobacter	Rhodanobacter sp. SCN 68-63
1	Bacteria	Proteobacteria	Gammaproteobacteria	Xanthomonadales	Rhodanobacteraceae	Rhodanobacter	Rhodanobacter sp. Root561
1	Bacteria	Proteobacteria	Gammaproteobacteria	Xanthomonadales	Rhodanobacteraceae	Rhodanobacter	Rhodanobacter sp. Root179
1	Bacteria	Proteobacteria	Gammaproteobacteria	Xanthomonadales	Rhodanobacteraceae	Rhodanobacter	Rhodanobacter sp. OR444
1	Bacteria	Proteobacteria	Gammaproteobacteria	Xanthomonadales	Rhodanobacteraceae	Rhodanobacter	Rhodanobacter sp. FW510-R12
1	Bacteria	Proteobacteria	Gammaproteobacteria	Xanthomonadales	Rhodanobacteraceae	Rhodanobacter	Rhodanobacter sp. C05
1	Bacteria	Proteobacteria	Gammaproteobacteria	Xanthomonadales	Rhodanobacteraceae	Rhodanobacter	Rhodanobacter sp. 67-28
1	Bacteria	Proteobacteria	Gammaproteobacteria	Xanthomonadales	Rhodanobacteraceae	Rhodanobacter	Rhodanobacter sp. 115
1	Bacteria	Proteobacteria	Gammaproteobacteria	Xanthomonadales	Rhodanobacteraceae	Luteibacter	Luteibacter sp. 9143
1	Bacteria	Proteobacteria	Gammaproteobacteria	Xanthomonadales	Rhodanobacteraceae	Luteibacter	Luteibacter sp. 9133
1	Bacteria	Proteobacteria	Gammaproteobacteria	Xanthomonadales	Rhodanobacteraceae	Dyella	Dyella soli
1	Bacteria	Proteobacteria	Gammaproteobacteria	Vibrionales	Vibrionaceae	Vibrio	Vibrio xiamenensis
1	Bacteria	Proteobacteria	Gammaproteobacteria	Vibrionales	Vibrionaceae	Vibrio	Vibrio variabilis
1	Bacteria	Proteobacteria	Gammaproteobacteria	Vibrionales	Vibrionaceae	Vibrio	Vibrio toranzoniae
1	Bacteria	Proteobacteria	Gammaproteobacteria	Vibrionales	Vibrionaceae	Vibrio	Vibrio sp. qd031
1	Bacteria	Proteobacteria	Gammaproteobacteria	Vibrionales	Vibrionaceae	Vibrio	Vibrio sp. VT2(2010)
1	Bacteria	Proteobacteria	Gammaproteobacteria	Vibrionales	Vibrionaceae	Vibrio	Vibrio sp. MEBiC08052
1	Bacteria	Proteobacteria	Gammaproteobacteria	Vibrionales	Vibrionaceae	Vibrio	Vibrio sp. J2-12
1	Bacteria	Proteobacteria	Gammaproteobacteria	Vibrionales	Vibrionaceae	Vibrio	Vibrio sp. HENC-03
1	Bacteria	Proteobacteria	Gammaproteobacteria	Vibrionales	Vibrionaceae	Vibrio	Vibrio sp. ECSMB14106
1	Bacteria	Proteobacteria	Gammaproteobacteria	Vibrionales	Vibrionaceae	Vibrio	Vibrio sp. CECT 9027
1	Bacteria	Proteobacteria	Gammaproteobacteria	Vibrionales	Vibrionaceae	Vibrio	Vibrio sp. 10N.261.45.E1
1	Bacteria	Proteobacteria	Gammaproteobacteria	Vibrionales	Vibrionaceae	Vibrio	Vibrio sonorensis
1	Bacteria	Proteobacteria	Gammaproteobacteria	Vibrionales	Vibrionaceae	Vibrio	Vibrio scophthalmi
1	Bacteria	Proteobacteria	Gammaproteobacteria	Vibrionales	Vibrionaceae	Vibrio	Vibrio proteolyticus
1	Bacteria	Proteobacteria	Gammaproteobacteria	Vibrionales	Vibrionaceae	Vibrio	Vibrio ponticus
1	Bacteria	Proteobacteria	Gammaproteobacteria	Vibrionales	Vibrionaceae	Vibrio	Vibrio panuliri
1	Bacteria	Proteobacteria	Gammaproteobacteria	Vibrionales	Vibrionaceae	Vibrio	Vibrio oreintalis group
1	Bacteria	Proteobacteria	Gammaproteobacteria	Vibrionales	Vibrionaceae	Vibrio	Vibrio oreintalis group
1	Bacteria	Proteobacteria	Gammaproteobacteria	Vibrionales	Vibrionaceae	Vibrio	Vibrio oreintalis group
1	Bacteria	Proteobacteria	Gammaproteobacteria	Vibrionales	Vibrionaceae	Vibrio	Vibrio oreintalis group
1	Bacteria	Proteobacteria	Gammaproteobacteria	Vibrionales	Vibrionaceae	Vibrio	Vibrio nereis
1	Bacteria	Proteobacteria	Gammaproteobacteria	Vibrionales	Vibrionaceae	Vibrio	Vibrio neptunius
1	Bacteria	Proteobacteria	Gammaproteobacteria	Vibrionales	Vibrionaceae	Vibrio	Vibrio navarrensis
1	Bacteria	Proteobacteria	Gammaproteobacteria	Vibrionales	Vibrionaceae	Vibrio	Vibrio mimicus
1	Bacteria	Proteobacteria	Gammaproteobacteria	Vibrionales	Vibrionaceae	Vibrio	Vibrio mimicus
1	Bacteria	Proteobacteria	Gammaproteobacteria	Vibrionales	Vibrionaceae	Vibrio	Vibrio litoralis
1	Bacteria	Proteobacteria	Gammaproteobacteria	Vibrionales	Vibrionaceae	Vibrio	Vibrio harveyi group
1	Bacteria	Proteobacteria	Gammaproteobacteria	Vibrionales	Vibrionaceae	Vibrio	Vibrio harveyi group
1	Bacteria	Proteobacteria	Gammaproteobacteria	Vibrionales	Vibrionaceae	Vibrio	Vibrio harveyi group
1	Bacteria	Proteobacteria	Gammaproteobacteria	Vibrionales	Vibrionaceae	Vibrio	Vibrio harveyi group
1	Bacteria	Proteobacteria	Gammaproteobacteria	Vibrionales	Vibrionaceae	Vibrio	Vibrio harveyi group
1	Bacteria	Proteobacteria	Gammaproteobacteria	Vibrionales	Vibrionaceae	Vibrio	Vibrio harveyi group
1	Bacteria	Proteobacteria	Gammaproteobacteria	Vibrionales	Vibrionaceae	Vibrio	Vibrio harveyi group
1	Bacteria	Proteobacteria	Gammaproteobacteria	Vibrionales	Vibrionaceae	Vibrio	Vibrio gazogenes
1	Bacteria	Proteobacteria	Gammaproteobacteria	Vibrionales	Vibrionaceae	Vibrio	Vibrio gazogenes
1	Bacteria	Proteobacteria	Gammaproteobacteria	Vibrionales	Vibrionaceae	Vibrio	Vibrio galatheae
1	Bacteria	Proteobacteria	Gammaproteobacteria	Vibrionales	Vibrionaceae	Vibrio	Vibrio fortis
1	Bacteria	Proteobacteria	Gammaproteobacteria	Vibrionales	Vibrionaceae	Vibrio	Vibrio ezurae
1	Bacteria	Proteobacteria	Gammaproteobacteria	Vibrionales	Vibrionaceae	Vibrio	Vibrio diazotrophicus
1	Bacteria	Proteobacteria	Gammaproteobacteria	Vibrionales	Vibrionaceae	Salinivibrio	Salinivibrio sp. IB563
1	Bacteria	Proteobacteria	Gammaproteobacteria	Vibrionales	Vibrionaceae	Salinivibrio	Salinivibrio sp. IB560
1	Bacteria	Proteobacteria	Gammaproteobacteria	Vibrionales	Vibrionaceae	Salinivibrio	Salinivibrio sp. ES.052
1	Bacteria	Proteobacteria	Gammaproteobacteria	Vibrionales	Vibrionaceae	Photobacterium	Photobacterium sp. J15
1	Bacteria	Proteobacteria	Gammaproteobacteria	Vibrionales	Vibrionaceae	Photobacterium	Photobacterium iliopiscarium
1	Bacteria	Proteobacteria	Gammaproteobacteria	Vibrionales	Vibrionaceae	Photobacterium	Photobacterium halotolerans
1	Bacteria	Proteobacteria	Gammaproteobacteria	Vibrionales	Vibrionaceae	Photobacterium	Photobacterium aphoticum
1	Bacteria	Proteobacteria	Gammaproteobacteria	Vibrionales	Vibrionaceae	Photobacterium	Photobacterium angustum
1	Bacteria	Proteobacteria	Gammaproteobacteria	Vibrionales	Vibrionaceae	Enterovibrio	
1	Bacteria	Proteobacteria	Gammaproteobacteria	Vibrionales			
1	Bacteria	Proteobacteria	Gammaproteobacteria	Thiotrichales	Thiotrichaceae	Thiothrix	Thiothrix nivea
1	Bacteria	Proteobacteria	Gammaproteobacteria	Thiotrichales	Thiotrichaceae		
1	Bacteria	Proteobacteria	Gammaproteobacteria	Thiotrichales	Piscirickettsiaceae	unclassified Piscirickettsiaceae	Piscirickettsiaceae bacterium NZ-RLO
1	Bacteria	Proteobacteria	Gammaproteobacteria	Thiotrichales	Piscirickettsiaceae	Thiomicrospira	Thiomicrospira sp. WB1
1	Bacteria	Proteobacteria	Gammaproteobacteria	Thiotrichales	Piscirickettsiaceae	Thiomicrospira	Thiomicrospira sp. Milos-T2
1	Bacteria	Proteobacteria	Gammaproteobacteria	Thiotrichales	Piscirickettsiaceae	Thiomicrospira	Thiomicrospira halophila
1	Bacteria	Proteobacteria	Gammaproteobacteria	Thiotrichales	Piscirickettsiaceae	Thiomicrospira	Thiomicrospira chilensis
1	Bacteria	Proteobacteria	Gammaproteobacteria	Thiotrichales	Piscirickettsiaceae	Thioalkalimicrobium	Thioalkalimicrobium sp. ALE5
1	Bacteria	Proteobacteria	Gammaproteobacteria	Thiotrichales	Piscirickettsiaceae	Thioalkalimicrobium	Thioalkalimicrobium aerophilum
1	Bacteria	Proteobacteria	Gammaproteobacteria	Thiotrichales	Piscirickettsiaceae	Piscirickettsia	Piscirickettsia salmonis
1	Bacteria	Proteobacteria	Gammaproteobacteria	Thiotrichales	Piscirickettsiaceae	Piscirickettsia	Piscirickettsia litoralis
1	Bacteria	Proteobacteria	Gammaproteobacteria	Thiotrichales	Piscirickettsiaceae	Methylophaga	Methylophaga sulfidovorans
1	Bacteria	Proteobacteria	Gammaproteobacteria	Thiotrichales	Piscirickettsiaceae	Hydrogenovibrio	Hydrogenovibrio marinus
1	Bacteria	Proteobacteria	Gammaproteobacteria	Thiotrichales	Piscirickettsiaceae	Cycloclasticus	Cycloclasticus sp. Phe_18
1	Bacteria	Proteobacteria	Gammaproteobacteria	Thiotrichales	Francisellaceae	Francisella	Francisella tularensis
1	Bacteria	Proteobacteria	Gammaproteobacteria	Thiotrichales	Francisellaceae	Francisella	Francisella sp. MA067296
1	Bacteria	Proteobacteria	Gammaproteobacteria	Thiotrichales	Francisellaceae	Francisella	Francisella sp. CA97-1460
1	Bacteria	Proteobacteria	Gammaproteobacteria	Salinisphaerales	Salinisphaeraceae	Salinisphaera	Salinisphaera shabanensis
1	Bacteria	Proteobacteria	Gammaproteobacteria	Pseudomonadales	unclassified Pseudomonadales	unclassified Pseudomonadales (miscellaneous)	Pseudomonadales bacterium RIFCSPHIGHO2_02_FULL_60_43
1	Bacteria	Proteobacteria	Gammaproteobacteria	Pseudomonadales	unclassified Pseudomonadales	unclassified Pseudomonadales (miscellaneous)	Pseudomonadales bacterium GWC1_66_9
1	Bacteria	Proteobacteria	Gammaproteobacteria	Pseudomonadales	Pseudomonadaceae	Pseudomonas	Pseudomonas yamanorum
1	Bacteria	Proteobacteria	Gammaproteobacteria	Pseudomonadales	Pseudomonadaceae	Pseudomonas	Pseudomonas weihenstephanensis
1	Bacteria	Proteobacteria	Gammaproteobacteria	Pseudomonadales	Pseudomonadaceae	Pseudomonas	Pseudomonas versuta
1	Bacteria	Proteobacteria	Gammaproteobacteria	Pseudomonadales	Pseudomonadaceae	Pseudomonas	Pseudomonas thivervalensis
1	Bacteria	Proteobacteria	Gammaproteobacteria	Pseudomonadales	Pseudomonadaceae	Pseudomonas	Pseudomonas taiwanensis
1	Bacteria	Proteobacteria	Gammaproteobacteria	Pseudomonadales	Pseudomonadaceae	Pseudomonas	Pseudomonas syringae group
1	Bacteria	Proteobacteria	Gammaproteobacteria	Pseudomonadales	Pseudomonadaceae	Pseudomonas	Pseudomonas syringae group
1	Bacteria	Proteobacteria	Gammaproteobacteria	Pseudomonadales	Pseudomonadaceae	Pseudomonas	Pseudomonas syringae group
1	Bacteria	Proteobacteria	Gammaproteobacteria	Pseudomonadales	Pseudomonadaceae	Pseudomonas	Pseudomonas syringae group
1	Bacteria	Proteobacteria	Gammaproteobacteria	Pseudomonadales	Pseudomonadaceae	Pseudomonas	Pseudomonas syringae group
1	Bacteria	Proteobacteria	Gammaproteobacteria	Pseudomonadales	Pseudomonadaceae	Pseudomonas	Pseudomonas syringae group
1	Bacteria	Proteobacteria	Gammaproteobacteria	Pseudomonadales	Pseudomonadaceae	Pseudomonas	Pseudomonas syringae group
1	Bacteria	Proteobacteria	Gammaproteobacteria	Pseudomonadales	Pseudomonadaceae	Pseudomonas	Pseudomonas syringae group
1	Bacteria	Proteobacteria	Gammaproteobacteria	Pseudomonadales	Pseudomonadaceae	Pseudomonas	Pseudomonas stutzeri group
1	Bacteria	Proteobacteria	Gammaproteobacteria	Pseudomonadales	Pseudomonadaceae	Pseudomonas	Pseudomonas sp. bs2935
1	Bacteria	Proteobacteria	Gammaproteobacteria	Pseudomonadales	Pseudomonadaceae	Pseudomonas	Pseudomonas sp. Z003-0.4C(8344-21)
1	Bacteria	Proteobacteria	Gammaproteobacteria	Pseudomonadales	Pseudomonadaceae	Pseudomonas	Pseudomonas sp. WCHP16
1	Bacteria	Proteobacteria	Gammaproteobacteria	Pseudomonadales	Pseudomonadaceae	Pseudomonas	Pseudomonas sp. URIL14HWK12:I6
1	Bacteria	Proteobacteria	Gammaproteobacteria	Pseudomonadales	Pseudomonadaceae	Pseudomonas	Pseudomonas sp. TTU2014-105ASC
1	Bacteria	Proteobacteria	Gammaproteobacteria	Pseudomonadales	Pseudomonadaceae	Pseudomonas	Pseudomonas sp. St29
1	Bacteria	Proteobacteria	Gammaproteobacteria	Pseudomonadales	Pseudomonadaceae	Pseudomonas	Pseudomonas sp. SHC52
1	Bacteria	Proteobacteria	Gammaproteobacteria	Pseudomonadales	Pseudomonadaceae	Pseudomonas	Pseudomonas sp. Root562
1	Bacteria	Proteobacteria	Gammaproteobacteria	Pseudomonadales	Pseudomonadaceae	Pseudomonas	Pseudomonas sp. P482
1	Bacteria	Proteobacteria	Gammaproteobacteria	Pseudomonadales	Pseudomonadaceae	Pseudomonas	Pseudomonas sp. NFR09
1	Bacteria	Proteobacteria	Gammaproteobacteria	Pseudomonadales	Pseudomonadaceae	Pseudomonas	Pseudomonas sp. NFPP33
1	Bacteria	Proteobacteria	Gammaproteobacteria	Pseudomonadales	Pseudomonadaceae	Pseudomonas	Pseudomonas sp. NFPP07
1	Bacteria	Proteobacteria	Gammaproteobacteria	Pseudomonadales	Pseudomonadaceae	Pseudomonas	Pseudomonas sp. NFACC42-2
1	Bacteria	Proteobacteria	Gammaproteobacteria	Pseudomonadales	Pseudomonadaceae	Pseudomonas	Pseudomonas sp. NFACC39-1
1	Bacteria	Proteobacteria	Gammaproteobacteria	Pseudomonadales	Pseudomonadaceae	Pseudomonas	Pseudomonas sp. NBRC 111136
1	Bacteria	Proteobacteria	Gammaproteobacteria	Pseudomonadales	Pseudomonadaceae	Pseudomonas	Pseudomonas sp. NBRC 111134
1	Bacteria	Proteobacteria	Gammaproteobacteria	Pseudomonadales	Pseudomonadaceae	Pseudomonas	Pseudomonas sp. NBRC 111127
1	Bacteria	Proteobacteria	Gammaproteobacteria	Pseudomonadales	Pseudomonadaceae	Pseudomonas	Pseudomonas sp. MRSN12121
1	Bacteria	Proteobacteria	Gammaproteobacteria	Pseudomonadales	Pseudomonadaceae	Pseudomonas	Pseudomonas sp. MIACH
1	Bacteria	Proteobacteria	Gammaproteobacteria	Pseudomonadales	Pseudomonadaceae	Pseudomonas	Pseudomonas sp. M1
1	Bacteria	Proteobacteria	Gammaproteobacteria	Pseudomonadales	Pseudomonadaceae	Pseudomonas	Pseudomonas sp. Leaf15
1	Bacteria	Proteobacteria	Gammaproteobacteria	Pseudomonadales	Pseudomonadaceae	Pseudomonas	Pseudomonas sp. LPH1
1	Bacteria	Proteobacteria	Gammaproteobacteria	Pseudomonadales	Pseudomonadaceae	Pseudomonas	Pseudomonas sp. KK4
1	Bacteria	Proteobacteria	Gammaproteobacteria	Pseudomonadales	Pseudomonadaceae	Pseudomonas	Pseudomonas sp. J237
1	Bacteria	Proteobacteria	Gammaproteobacteria	Pseudomonadales	Pseudomonadaceae	Pseudomonas	Pseudomonas sp. In5
1	Bacteria	Proteobacteria	Gammaproteobacteria	Pseudomonadales	Pseudomonadaceae	Pseudomonas	Pseudomonas sp. H2
1	Bacteria	Proteobacteria	Gammaproteobacteria	Pseudomonadales	Pseudomonadaceae	Pseudomonas	Pseudomonas sp. H1h
1	Bacteria	Proteobacteria	Gammaproteobacteria	Pseudomonadales	Pseudomonadaceae	Pseudomonas	Pseudomonas sp. GTC 16482
1	Bacteria	Proteobacteria	Gammaproteobacteria	Pseudomonadales	Pseudomonadaceae	Pseudomonas	Pseudomonas sp. GM84
1	Bacteria	Proteobacteria	Gammaproteobacteria	Pseudomonadales	Pseudomonadaceae	Pseudomonas	Pseudomonas sp. GM74
1	Bacteria	Proteobacteria	Gammaproteobacteria	Pseudomonadales	Pseudomonadaceae	Pseudomonas	Pseudomonas sp. GM41(2012)
1	Bacteria	Proteobacteria	Gammaproteobacteria	Pseudomonadales	Pseudomonadaceae	Pseudomonas	Pseudomonas sp. GM33
1	Bacteria	Proteobacteria	Gammaproteobacteria	Pseudomonadales	Pseudomonadaceae	Pseudomonas	Pseudomonas sp. GLE121
1	Bacteria	Proteobacteria	Gammaproteobacteria	Pseudomonadales	Pseudomonadaceae	Pseudomonas	Pseudomonas sp. FeS53a
1	Bacteria	Proteobacteria	Gammaproteobacteria	Pseudomonadales	Pseudomonadaceae	Pseudomonas	Pseudomonas sp. FGI182
1	Bacteria	Proteobacteria	Gammaproteobacteria	Pseudomonadales	Pseudomonadaceae	Pseudomonas	Pseudomonas sp. EpS/L25
1	Bacteria	Proteobacteria	Gammaproteobacteria	Pseudomonadales	Pseudomonadaceae	Pseudomonas	Pseudomonas sp. DSM 28140
1	Bacteria	Proteobacteria	Gammaproteobacteria	Pseudomonadales	Pseudomonadaceae	Pseudomonas	Pseudomonas sp. Chol1
1	Bacteria	Proteobacteria	Gammaproteobacteria	Pseudomonadales	Pseudomonadaceae	Pseudomonas	Pseudomonas sp. CO183
1	Bacteria	Proteobacteria	Gammaproteobacteria	Pseudomonadales	Pseudomonadaceae	Pseudomonas	Pseudomonas sp. CMR5c
1	Bacteria	Proteobacteria	Gammaproteobacteria	Pseudomonadales	Pseudomonadaceae	Pseudomonas	Pseudomonas sp. CF864
1	Bacteria	Proteobacteria	Gammaproteobacteria	Pseudomonadales	Pseudomonadaceae	Pseudomonas	Pseudomonas sp. CF149
1	Bacteria	Proteobacteria	Gammaproteobacteria	Pseudomonadales	Pseudomonadaceae	Pseudomonas	Pseudomonas sp. CBZ-4
1	Bacteria	Proteobacteria	Gammaproteobacteria	Pseudomonadales	Pseudomonadaceae	Pseudomonas	Pseudomonas sp. B28(2017)
1	Bacteria	Proteobacteria	Gammaproteobacteria	Pseudomonadales	Pseudomonadaceae	Pseudomonas	Pseudomonas sp. B10
1	Bacteria	Proteobacteria	Gammaproteobacteria	Pseudomonadales	Pseudomonadaceae	Pseudomonas	Pseudomonas sp. A3(2016)
1	Bacteria	Proteobacteria	Gammaproteobacteria	Pseudomonadales	Pseudomonadaceae	Pseudomonas	Pseudomonas sp. 63_8
1	Bacteria	Proteobacteria	Gammaproteobacteria	Pseudomonadales	Pseudomonadaceae	Pseudomonas	Pseudomonas sp. 44 R 15
1	Bacteria	Proteobacteria	Gammaproteobacteria	Pseudomonadales	Pseudomonadaceae	Pseudomonas	Pseudomonas sp. 22 E 5
1	Bacteria	Proteobacteria	Gammaproteobacteria	Pseudomonadales	Pseudomonadaceae	Pseudomonas	Pseudomonas sp. 11/12A
1	Bacteria	Proteobacteria	Gammaproteobacteria	Pseudomonadales	Pseudomonadaceae	Pseudomonas	Pseudomonas sp. 108238
1	Bacteria	Proteobacteria	Gammaproteobacteria	Pseudomonadales	Pseudomonadaceae	Pseudomonas	Pseudomonas rhizosphaerae
1	Bacteria	Proteobacteria	Gammaproteobacteria	Pseudomonadales	Pseudomonadaceae	Pseudomonas	Pseudomonas putida group
1	Bacteria	Proteobacteria	Gammaproteobacteria	Pseudomonadales	Pseudomonadaceae	Pseudomonas	Pseudomonas putida group
1	Bacteria	Proteobacteria	Gammaproteobacteria	Pseudomonadales	Pseudomonadaceae	Pseudomonas	Pseudomonas putida group
1	Bacteria	Proteobacteria	Gammaproteobacteria	Pseudomonadales	Pseudomonadaceae	Pseudomonas	Pseudomonas pachastrellae
1	Bacteria	Proteobacteria	Gammaproteobacteria	Pseudomonadales	Pseudomonadaceae	Pseudomonas	Pseudomonas moraviensis
1	Bacteria	Proteobacteria	Gammaproteobacteria	Pseudomonadales	Pseudomonadaceae	Pseudomonas	Pseudomonas marincola
1	Bacteria	Proteobacteria	Gammaproteobacteria	Pseudomonadales	Pseudomonadaceae	Pseudomonas	Pseudomonas kunmingensis
1	Bacteria	Proteobacteria	Gammaproteobacteria	Pseudomonadales	Pseudomonadaceae	Pseudomonas	Pseudomonas jessenii
1	Bacteria	Proteobacteria	Gammaproteobacteria	Pseudomonadales	Pseudomonadaceae	Pseudomonas	Pseudomonas frederiksbergensis
1	Bacteria	Proteobacteria	Gammaproteobacteria	Pseudomonadales	Pseudomonadaceae	Pseudomonas	Pseudomonas fluorescens group
1	Bacteria	Proteobacteria	Gammaproteobacteria	Pseudomonadales	Pseudomonadaceae	Pseudomonas	Pseudomonas fluorescens group
1	Bacteria	Proteobacteria	Gammaproteobacteria	Pseudomonadales	Pseudomonadaceae	Pseudomonas	Pseudomonas fluorescens group
1	Bacteria	Proteobacteria	Gammaproteobacteria	Pseudomonadales	Pseudomonadaceae	Pseudomonas	Pseudomonas fluorescens group
1	Bacteria	Proteobacteria	Gammaproteobacteria	Pseudomonadales	Pseudomonadaceae	Pseudomonas	Pseudomonas fluorescens group
1	Bacteria	Proteobacteria	Gammaproteobacteria	Pseudomonadales	Pseudomonadaceae	Pseudomonas	Pseudomonas fluorescens group
1	Bacteria	Proteobacteria	Gammaproteobacteria	Pseudomonadales	Pseudomonadaceae	Pseudomonas	Pseudomonas fluorescens group
1	Bacteria	Proteobacteria	Gammaproteobacteria	Pseudomonadales	Pseudomonadaceae	Pseudomonas	Pseudomonas fluorescens group
1	Bacteria	Proteobacteria	Gammaproteobacteria	Pseudomonadales	Pseudomonadaceae	Pseudomonas	Pseudomonas fluorescens group
1	Bacteria	Proteobacteria	Gammaproteobacteria	Pseudomonadales	Pseudomonadaceae	Pseudomonas	Pseudomonas entomophila
1	Bacteria	Proteobacteria	Gammaproteobacteria	Pseudomonadales	Pseudomonadaceae	Pseudomonas	Pseudomonas capeferrum
1	Bacteria	Proteobacteria	Gammaproteobacteria	Pseudomonadales	Pseudomonadaceae	Pseudomonas	Pseudomonas batumici
1	Bacteria	Proteobacteria	Gammaproteobacteria	Pseudomonadales	Pseudomonadaceae	Pseudomonas	Pseudomonas aeruginosa group
1	Bacteria	Proteobacteria	Gammaproteobacteria	Pseudomonadales	Pseudomonadaceae	Pseudomonas	Pseudomonas aeruginosa group
1	Bacteria	Proteobacteria	Gammaproteobacteria	Pseudomonadales	Pseudomonadaceae	Pseudomonas	Pseudomonas abietaniphila
1	Bacteria	Proteobacteria	Gammaproteobacteria	Pseudomonadales	Pseudomonadaceae	Azotobacter group	Azotobacter
1	Bacteria	Proteobacteria	Gammaproteobacteria	Pseudomonadales	Moraxellaceae	Psychrobacter	Psychrobacter sp. P11G5
1	Bacteria	Proteobacteria	Gammaproteobacteria	Pseudomonadales	Moraxellaceae	Psychrobacter	Psychrobacter sp. P11F6
1	Bacteria	Proteobacteria	Gammaproteobacteria	Pseudomonadales	Moraxellaceae	Psychrobacter	Psychrobacter sp. JCM 18902
1	Bacteria	Proteobacteria	Gammaproteobacteria	Pseudomonadales	Moraxellaceae	Psychrobacter	Psychrobacter sp. G
1	Bacteria	Proteobacteria	Gammaproteobacteria	Pseudomonadales	Moraxellaceae	Psychrobacter	Psychrobacter sp. DAB_AL43B
1	Bacteria	Proteobacteria	Gammaproteobacteria	Pseudomonadales	Moraxellaceae	Psychrobacter	Psychrobacter phenylpyruvicus
1	Bacteria	Proteobacteria	Gammaproteobacteria	Pseudomonadales	Moraxellaceae	Psychrobacter	Psychrobacter arcticus
1	Bacteria	Proteobacteria	Gammaproteobacteria	Pseudomonadales	Moraxellaceae	Moraxella	Moraxella sp. CK-1
1	Bacteria	Proteobacteria	Gammaproteobacteria	Pseudomonadales	Moraxellaceae	Moraxella	Moraxella porci
1	Bacteria	Proteobacteria	Gammaproteobacteria	Pseudomonadales	Moraxellaceae	Moraxella	Moraxella ovis
1	Bacteria	Proteobacteria	Gammaproteobacteria	Pseudomonadales	Moraxellaceae	Moraxella	Moraxella nonliquefaciens
1	Bacteria	Proteobacteria	Gammaproteobacteria	Pseudomonadales	Moraxellaceae	Moraxella	Moraxella cuniculi
1	Bacteria	Proteobacteria	Gammaproteobacteria	Pseudomonadales	Moraxellaceae	Moraxella	Moraxella caviae
1	Bacteria	Proteobacteria	Gammaproteobacteria	Pseudomonadales	Moraxellaceae	Moraxella	Moraxella catarrhalis
1	Bacteria	Proteobacteria	Gammaproteobacteria	Pseudomonadales	Moraxellaceae	Moraxella	Moraxella bovoculi
1	Bacteria	Proteobacteria	Gammaproteobacteria	Pseudomonadales	Moraxellaceae	Moraxella	Moraxella atlantae
1	Bacteria	Proteobacteria	Gammaproteobacteria	Pseudomonadales	Moraxellaceae	Acinetobacter	Acinetobacter towneri
1	Bacteria	Proteobacteria	Gammaproteobacteria	Pseudomonadales	Moraxellaceae	Acinetobacter	Acinetobacter towneri
1	Bacteria	Proteobacteria	Gammaproteobacteria	Pseudomonadales	Moraxellaceae	Acinetobacter	Acinetobacter tjernbergiae
1	Bacteria	Proteobacteria	Gammaproteobacteria	Pseudomonadales	Moraxellaceae	Acinetobacter	Acinetobacter tandoii
1	Bacteria	Proteobacteria	Gammaproteobacteria	Pseudomonadales	Moraxellaceae	Acinetobacter	Acinetobacter sp. yr461
1	Bacteria	Proteobacteria	Gammaproteobacteria	Pseudomonadales	Moraxellaceae	Acinetobacter	Acinetobacter sp. WCHA60
1	Bacteria	Proteobacteria	Gammaproteobacteria	Pseudomonadales	Moraxellaceae	Acinetobacter	Acinetobacter sp. V2
1	Bacteria	Proteobacteria	Gammaproteobacteria	Pseudomonadales	Moraxellaceae	Acinetobacter	Acinetobacter sp. TGL-Y2
1	Bacteria	Proteobacteria	Gammaproteobacteria	Pseudomonadales	Moraxellaceae	Acinetobacter	Acinetobacter sp. SFC
1	Bacteria	Proteobacteria	Gammaproteobacteria	Pseudomonadales	Moraxellaceae	Acinetobacter	Acinetobacter sp. SFA
1	Bacteria	Proteobacteria	Gammaproteobacteria	Pseudomonadales	Moraxellaceae	Acinetobacter	Acinetobacter sp. Root1280
1	Bacteria	Proteobacteria	Gammaproteobacteria	Pseudomonadales	Moraxellaceae	Acinetobacter	Acinetobacter sp. NIPH 298
1	Bacteria	Proteobacteria	Gammaproteobacteria	Pseudomonadales	Moraxellaceae	Acinetobacter	Acinetobacter sp. NIPH 2168
1	Bacteria	Proteobacteria	Gammaproteobacteria	Pseudomonadales	Moraxellaceae	Acinetobacter	Acinetobacter sp. DSM 11652
1	Bacteria	Proteobacteria	Gammaproteobacteria	Pseudomonadales	Moraxellaceae	Acinetobacter	Acinetobacter sp. COS3
1	Bacteria	Proteobacteria	Gammaproteobacteria	Pseudomonadales	Moraxellaceae	Acinetobacter	Acinetobacter sp. ANC 4149
1	Bacteria	Proteobacteria	Gammaproteobacteria	Pseudomonadales	Moraxellaceae	Acinetobacter	Acinetobacter sp. ANC 3789
1	Bacteria	Proteobacteria	Gammaproteobacteria	Pseudomonadales	Moraxellaceae	Acinetobacter	Acinetobacter schindleri
1	Bacteria	Proteobacteria	Gammaproteobacteria	Pseudomonadales	Moraxellaceae	Acinetobacter	Acinetobacter parvus
1	Bacteria	Proteobacteria	Gammaproteobacteria	Pseudomonadales	Moraxellaceae	Acinetobacter	Acinetobacter kyonggiensis
1	Bacteria	Proteobacteria	Gammaproteobacteria	Pseudomonadales	Moraxellaceae	Acinetobacter	Acinetobacter junii

1	Bacteria	Proteobacteria	Gammaproteobacteria	Pseudomonadales	Moraxellaceae	Acinetobacter	Acinetobacter johnsonii
1	Bacteria	Proteobacteria	Gammaproteobacteria	Pseudomonadales	Moraxellaceae	Acinetobacter	Acinetobacter gyllenbergii
1	Bacteria	Proteobacteria	Gammaproteobacteria	Pseudomonadales	Moraxellaceae	Acinetobacter	Acinetobacter calcoaceticus/baumannii complex
1	Bacteria	Proteobacteria	Gammaproteobacteria	Pseudomonadales	Moraxellaceae	Acinetobacter	Acinetobacter bohemicus
1	Bacteria	Proteobacteria	Gammaproteobacteria	Pseudomonadales	Moraxellaceae	Acinetobacter	Acinetobacter bereziniae
1	Bacteria	Proteobacteria	Gammaproteobacteria	Pasteurellales	Pasteurellaceae	Rodentibacter	Rodentibacter sp. Ppn85
1	Bacteria	Proteobacteria	Gammaproteobacteria	Pasteurellales	Pasteurellaceae	Rodentibacter	Rodentibacter heidelbergensis
1	Bacteria	Proteobacteria	Gammaproteobacteria	Pasteurellales	Pasteurellaceae	Pasteurella	Pasteurella testudinis
1	Bacteria	Proteobacteria	Gammaproteobacteria	Pasteurellales	Pasteurellaceae	Pasteurella	Pasteurella skyensis
1	Bacteria	Proteobacteria	Gammaproteobacteria	Pasteurellales	Pasteurellaceae	Pasteurella	Pasteurella multocida
1	Bacteria	Proteobacteria	Gammaproteobacteria	Pasteurellales	Pasteurellaceae	Pasteurella	Pasteurella bettyae
1	Bacteria	Proteobacteria	Gammaproteobacteria	Pasteurellales	Pasteurellaceae	Muribacter	Muribacter muris
1	Bacteria	Proteobacteria	Gammaproteobacteria	Pasteurellales	Pasteurellaceae	Mannheimia	Mannheimia varigena
1	Bacteria	Proteobacteria	Gammaproteobacteria	Pasteurellales	Pasteurellaceae	Mannheimia	Mannheimia sp. USDA-ARS-USMARC-1261
1	Bacteria	Proteobacteria	Gammaproteobacteria	Pasteurellales	Pasteurellaceae	Mannheimia	Mannheimia haemolytica
1	Bacteria	Proteobacteria	Gammaproteobacteria	Pasteurellales	Pasteurellaceae	Haemophilus	Haemophilus paraphrohaemolyticus
1	Bacteria	Proteobacteria	Gammaproteobacteria	Pasteurellales	Pasteurellaceae	Haemophilus	Haemophilus parahaemolyticus
1	Bacteria	Proteobacteria	Gammaproteobacteria	Pasteurellales	Pasteurellaceae	Haemophilus	Haemophilus haemolyticus
1	Bacteria	Proteobacteria	Gammaproteobacteria	Pasteurellales	Pasteurellaceae	Haemophilus	Haemophilus aegyptius
1	Bacteria	Proteobacteria	Gammaproteobacteria	Pasteurellales	Pasteurellaceae	Gallibacterium	Gallibacterium salpingitidis
1	Bacteria	Proteobacteria	Gammaproteobacteria	Pasteurellales	Pasteurellaceae	Gallibacterium	Gallibacterium genomosp. 1
1	Bacteria	Proteobacteria	Gammaproteobacteria	Pasteurellales	Pasteurellaceae	Bibersteinia	Bibersteinia trehalosi
1	Bacteria	Proteobacteria	Gammaproteobacteria	Pasteurellales	Pasteurellaceae	Aggregatibacter	Aggregatibacter segnis
1	Bacteria	Proteobacteria	Gammaproteobacteria	Pasteurellales	Pasteurellaceae	Aggregatibacter	Aggregatibacter aphrophilus
1	Bacteria	Proteobacteria	Gammaproteobacteria	Pasteurellales	Pasteurellaceae	Aggregatibacter	
1	Bacteria	Proteobacteria	Gammaproteobacteria	Pasteurellales	Pasteurellaceae	Actinobacillus	Actinobacillus ureae
1	Bacteria	Proteobacteria	Gammaproteobacteria	Pasteurellales	Pasteurellaceae	Actinobacillus	Actinobacillus pleuropneumoniae
1	Bacteria	Proteobacteria	Gammaproteobacteria	Pasteurellales	Pasteurellaceae	Actinobacillus	Actinobacillus minor
1	Bacteria	Proteobacteria	Gammaproteobacteria	Orbales	Orbaceae	Gilliamella	Gilliamella mensalis
1	Bacteria	Proteobacteria	Gammaproteobacteria	Orbales	Orbaceae	Gilliamella	Gilliamella bombicola
1	Bacteria	Proteobacteria	Gammaproteobacteria	Orbales	Orbaceae	Gilliamella	Gilliamella apicola
1	Bacteria	Proteobacteria	Gammaproteobacteria	Oceanospirillales	unclassified Oceanospirillales	Terasakiispira	Terasakiispira papahanaumokuakeensis
1	Bacteria	Proteobacteria	Gammaproteobacteria	Oceanospirillales	environmental samples	uncultured Oceanospirillales bacterium HF4000_21D01	
1	Bacteria	Proteobacteria	Gammaproteobacteria	Oceanospirillales	Saccharospirillaceae	Gynuella	Gynuella sunshinyii
1	Bacteria	Proteobacteria	Gammaproteobacteria	Oceanospirillales	Oleiphilaceae	Oleiphilus	Oleiphilus sp. HI0125
1	Bacteria	Proteobacteria	Gammaproteobacteria	Oceanospirillales	Oleiphilaceae	Oleiphilus	Oleiphilus sp. HI0118
1	Bacteria	Proteobacteria	Gammaproteobacteria	Oceanospirillales	Oceanospirillaceae	unclassified Oceanospirillaceae	Osedax symbiont Rs1
1	Bacteria	Proteobacteria	Gammaproteobacteria	Oceanospirillales	Oceanospirillaceae	Oceanospirillum	
1	Bacteria	Proteobacteria	Gammaproteobacteria	Oceanospirillales	Oceanospirillaceae	Neptunomonas	
1	Bacteria	Proteobacteria	Gammaproteobacteria	Oceanospirillales	Oceanospirillaceae	Neptuniibacter	Neptuniibacter pectenicola
1	Bacteria	Proteobacteria	Gammaproteobacteria	Oceanospirillales	Oceanospirillaceae	Marinomonas	Marinomonas spartinae
1	Bacteria	Proteobacteria	Gammaproteobacteria	Oceanospirillales	Oceanospirillaceae	Marinomonas	Marinomonas sp. S3726
1	Bacteria	Proteobacteria	Gammaproteobacteria	Oceanospirillales	Oceanospirillaceae	Marinomonas	Marinomonas sp. MWYL1
1	Bacteria	Proteobacteria	Gammaproteobacteria	Oceanospirillales	Oceanospirillaceae	Marinomonas	Marinomonas sp. MED121
1	Bacteria	Proteobacteria	Gammaproteobacteria	Oceanospirillales	Oceanospirillaceae	Marinomonas	Marinomonas posidonica
1	Bacteria	Proteobacteria	Gammaproteobacteria	Oceanospirillales	Oceanospirillaceae	Marinomonas	Marinomonas gallaica
1	Bacteria	Proteobacteria	Gammaproteobacteria	Oceanospirillales	Oceanospirillaceae	Marinomonas	Marinomonas aquimarina
1	Bacteria	Proteobacteria	Gammaproteobacteria	Oceanospirillales	Kangiellaceae	Kangiella	Kangiella geojedonensis
1	Bacteria	Proteobacteria	Gammaproteobacteria	Oceanospirillales	Halomonadaceae	Salinicola	Salinicola socius
1	Bacteria	Proteobacteria	Gammaproteobacteria	Oceanospirillales	Halomonadaceae	Kushneria	Kushneria avicenniae
1	Bacteria	Proteobacteria	Gammaproteobacteria	Oceanospirillales	Halomonadaceae	Halomonas	Halomonas xianhensis
1	Bacteria	Proteobacteria	Gammaproteobacteria	Oceanospirillales	Halomonadaceae	Halomonas	Halomonas taeanensis
1	Bacteria	Proteobacteria	Gammaproteobacteria	Oceanospirillales	Halomonadaceae	Halomonas	Halomonas subterranea
1	Bacteria	Proteobacteria	Gammaproteobacteria	Oceanospirillales	Halomonadaceae	Halomonas	Halomonas sp. TD01
1	Bacteria	Proteobacteria	Gammaproteobacteria	Oceanospirillales	Halomonadaceae	Halomonas	Halomonas sp. PBN3
1	Bacteria	Proteobacteria	Gammaproteobacteria	Oceanospirillales	Halomonadaceae	Halomonas	Halomonas sp. HL-48
1	Bacteria	Proteobacteria	Gammaproteobacteria	Oceanospirillales	Halomonadaceae	Halomonas	Halomonas sp. GT
1	Bacteria	Proteobacteria	Gammaproteobacteria	Oceanospirillales	Halomonadaceae	Halomonas	Halomonas sp. GFAJ-1
1	Bacteria	Proteobacteria	Gammaproteobacteria	Oceanospirillales	Halomonadaceae	Halomonas	Halomonas sp. ALS9
1	Bacteria	Proteobacteria	Gammaproteobacteria	Oceanospirillales	Halomonadaceae	Halomonas	Halomonas sp. 54_146
1	Bacteria	Proteobacteria	Gammaproteobacteria	Oceanospirillales	Halomonadaceae	Halomonas	Halomonas sp. 'Soap Lake #7'
1	Bacteria	Proteobacteria	Gammaproteobacteria	Oceanospirillales	Halomonadaceae	Halomonas	Halomonas meridiana
1	Bacteria	Proteobacteria	Gammaproteobacteria	Oceanospirillales	Halomonadaceae	Halomonas	Halomonas hydrothermalis
1	Bacteria	Proteobacteria	Gammaproteobacteria	Oceanospirillales	Halomonadaceae	Halomonas	Halomonas elongata
1	Bacteria	Proteobacteria	Gammaproteobacteria	Oceanospirillales	Halomonadaceae	Halomonas	Halomonas chromatireducens
1	Bacteria	Proteobacteria	Gammaproteobacteria	Oceanospirillales	Halomonadaceae	Halomonas	Halomonas campaniensis
1	Bacteria	Proteobacteria	Gammaproteobacteria	Oceanospirillales	Hahellaceae	Hahella	
1	Bacteria	Proteobacteria	Gammaproteobacteria	Oceanospirillales	Alcanivoracaceae	Alcanivorax	Alcanivorax xenomutans
1	Bacteria	Proteobacteria	Gammaproteobacteria	Oceanospirillales	Alcanivoracaceae	Alcanivorax	Alcanivorax sp. KX64203
1	Bacteria	Proteobacteria	Gammaproteobacteria	Oceanospirillales	Alcanivoracaceae	Alcanivorax	Alcanivorax sp. DSM 26293
1	Bacteria	Proteobacteria	Gammaproteobacteria	Oceanospirillales	Alcanivoracaceae	Alcanivorax	Alcanivorax sp. CP2C
1	Bacteria	Proteobacteria	Gammaproteobacteria	Oceanospirillales	Alcanivoracaceae	Alcanivorax	Alcanivorax jadensis
1	Bacteria	Proteobacteria	Gammaproteobacteria	Oceanospirillales	Alcanivoracaceae	Alcanivorax	Alcanivorax borkumensis
1	Bacteria	Proteobacteria	Gammaproteobacteria	Nevskiales	Sinobacteraceae	Hydrocarboniphaga	
1	Bacteria	Proteobacteria	Gammaproteobacteria	Methylococcales	Methylococcaceae	Methylomonas	Methylomonas sp. LW13
1	Bacteria	Proteobacteria	Gammaproteobacteria	Methylococcales	Methylococcaceae	Methylomonas	Methylomonas sp. 11b
1	Bacteria	Proteobacteria	Gammaproteobacteria	Methylococcales	Methylococcaceae	Methyloglobulus	Methyloglobulus morosus
1	Bacteria	Proteobacteria	Gammaproteobacteria	Methylococcales	Methylococcaceae	Methylobacter	Methylobacter marinus
1	Bacteria	Proteobacteria	Gammaproteobacteria	Legionellales	Legionellaceae	Tatlockia	Tatlockia micdadei
1	Bacteria	Proteobacteria	Gammaproteobacteria	Legionellales	Legionellaceae	Tatlockia	
1	Bacteria	Proteobacteria	Gammaproteobacteria	Legionellales	Legionellaceae	Legionella	Legionella worsleiensis
1	Bacteria	Proteobacteria	Gammaproteobacteria	Legionellales	Legionellaceae	Legionella	Legionella wadsworthii
1	Bacteria	Proteobacteria	Gammaproteobacteria	Legionellales	Legionellaceae	Legionella	Legionella steigerwaltii
1	Bacteria	Proteobacteria	Gammaproteobacteria	Legionellales	Legionellaceae	Legionella	Legionella steelei
1	Bacteria	Proteobacteria	Gammaproteobacteria	Legionellales	Legionellaceae	Legionella	Legionella spiritensis
1	Bacteria	Proteobacteria	Gammaproteobacteria	Legionellales	Legionellaceae	Legionella	Legionella rubrilucens
1	Bacteria	Proteobacteria	Gammaproteobacteria	Legionellales	Legionellaceae	Legionella	Legionella quateirensis
1	Bacteria	Proteobacteria	Gammaproteobacteria	Legionellales	Legionellaceae	Legionella	Legionella lansingensis
1	Bacteria	Proteobacteria	Gammaproteobacteria	Legionellales	Legionellaceae	Legionella	Legionella cincinnatiensis
1	Bacteria	Proteobacteria	Gammaproteobacteria	Legionellales	Legionellaceae	Legionella	Legionella cherrii
1	Bacteria	Proteobacteria	Gammaproteobacteria	Legionellales	Legionellaceae	Legionella	Legionella birminghamensis
1	Bacteria	Proteobacteria	Gammaproteobacteria	Legionellales	Legionellaceae	Legionella	Legionella anisa
1	Bacteria	Proteobacteria	Gammaproteobacteria	Legionellales	Legionellaceae	Fluoribacter	Fluoribacter gormanii
1	Bacteria	Proteobacteria	Gammaproteobacteria	Legionellales	Legionellaceae	Fluoribacter	Fluoribacter bozemanae
1	Bacteria	Proteobacteria	Gammaproteobacteria	Legionellales	Coxiellaceae	Rickettsiella	Rickettsiella grylli
1	Bacteria	Proteobacteria	Gammaproteobacteria	Legionellales	Coxiellaceae	Coxiella	Coxiella sp. RIFCSPHIGHO2_12_FULL_42_15
1	Bacteria	Proteobacteria	Gammaproteobacteria	Enterobacterales	unclassified Enterobacterales	Plesiomonas	Plesiomonas shigelloides
1	Bacteria	Proteobacteria	Gammaproteobacteria	Enterobacterales	Yersiniaceae	Yersinia	Yersinia pseudotuberculosis complex
1	Bacteria	Proteobacteria	Gammaproteobacteria	Enterobacterales	Yersiniaceae	Yersinia	Yersinia pseudotuberculosis complex
1	Bacteria	Proteobacteria	Gammaproteobacteria	Enterobacterales	Yersiniaceae	Yersinia	Yersinia pseudotuberculosis complex
1	Bacteria	Proteobacteria	Gammaproteobacteria	Enterobacterales	Yersiniaceae	Yersinia	Yersinia pekkanenii
1	Bacteria	Proteobacteria	Gammaproteobacteria	Enterobacterales	Yersiniaceae	Serratia	Serratia sp. Leaf50
1	Bacteria	Proteobacteria	Gammaproteobacteria	Enterobacterales	Yersiniaceae	Serratia	Serratia sp. FS14
1	Bacteria	Proteobacteria	Gammaproteobacteria	Enterobacterales	Yersiniaceae	Serratia	Serratia sp. C-1
1	Bacteria	Proteobacteria	Gammaproteobacteria	Enterobacterales	Yersiniaceae	Serratia	Serratia sp. 14-2641
1	Bacteria	Proteobacteria	Gammaproteobacteria	Enterobacterales	Yersiniaceae	Serratia	Serratia rubidaea
1	Bacteria	Proteobacteria	Gammaproteobacteria	Enterobacterales	Yersiniaceae	Serratia	Serratia proteamaculans
1	Bacteria	Proteobacteria	Gammaproteobacteria	Enterobacterales	Yersiniaceae	Serratia	Serratia liquefaciens
1	Bacteria	Proteobacteria	Gammaproteobacteria	Enterobacterales	Yersiniaceae	Serratia	Serratia fonticola
1	Bacteria	Proteobacteria	Gammaproteobacteria	Enterobacterales	Yersiniaceae	Serratia	Serratia ficaria
1	Bacteria	Proteobacteria	Gammaproteobacteria	Enterobacterales	Yersiniaceae	Rouxiella	Rouxiella badensis
1	Bacteria	Proteobacteria	Gammaproteobacteria	Enterobacterales	Yersiniaceae	Rahnella	Rahnella sp. Y9602
1	Bacteria	Proteobacteria	Gammaproteobacteria	Enterobacterales	Yersiniaceae	Ewingella	Ewingella americana
1	Bacteria	Proteobacteria	Gammaproteobacteria	Enterobacterales	Pectobacteriaceae	Sodalis	Sodalis sp. TME1
1	Bacteria	Proteobacteria	Gammaproteobacteria	Enterobacterales	Pectobacteriaceae	Pectobacterium	Pectobacterium betavasculorum
1	Bacteria	Proteobacteria	Gammaproteobacteria	Enterobacterales	Pectobacteriaceae	Lonsdalea	Lonsdalea quercina
1	Bacteria	Proteobacteria	Gammaproteobacteria	Enterobacterales	Pectobacteriaceae	Lonsdalea	Lonsdalea quercina
1	Bacteria	Proteobacteria	Gammaproteobacteria	Enterobacterales	Pectobacteriaceae	Dickeya	Dickeya dadantii
1	Bacteria	Proteobacteria	Gammaproteobacteria	Enterobacterales	Morganellaceae	Xenorhabdus	Xenorhabdus szentirmaii
1	Bacteria	Proteobacteria	Gammaproteobacteria	Enterobacterales	Morganellaceae	Xenorhabdus	Xenorhabdus poinarii
1	Bacteria	Proteobacteria	Gammaproteobacteria	Enterobacterales	Morganellaceae	Xenorhabdus	Xenorhabdus koppenhoeferi
1	Bacteria	Proteobacteria	Gammaproteobacteria	Enterobacterales	Morganellaceae	Xenorhabdus	Xenorhabdus japonica
1	Bacteria	Proteobacteria	Gammaproteobacteria	Enterobacterales	Morganellaceae	Xenorhabdus	Xenorhabdus bovienii
1	Bacteria	Proteobacteria	Gammaproteobacteria	Enterobacterales	Morganellaceae	Xenorhabdus	Xenorhabdus beddingii
1	Bacteria	Proteobacteria	Gammaproteobacteria	Enterobacterales	Morganellaceae	Providencia	Providencia rustigianii
1	Bacteria	Proteobacteria	Gammaproteobacteria	Enterobacterales	Morganellaceae	Providencia	Providencia heimbachae
1	Bacteria	Proteobacteria	Gammaproteobacteria	Enterobacterales	Morganellaceae	Proteus	Proteus hauseri
1	Bacteria	Proteobacteria	Gammaproteobacteria	Enterobacterales	Morganellaceae	Proteus	
1	Bacteria	Proteobacteria	Gammaproteobacteria	Enterobacterales	Morganellaceae	Photorhabdus	Photorhabdus heterorhabditis
1	Bacteria	Proteobacteria	Gammaproteobacteria	Enterobacterales	Morganellaceae	Cosenzaea	Cosenzaea myxofaciens
1	Bacteria	Proteobacteria	Gammaproteobacteria	Enterobacterales	Morganellaceae	Arsenophonus	
1	Bacteria	Proteobacteria	Gammaproteobacteria	Enterobacterales	Hafniaceae	Hafnia	Hafnia paralvei
1	Bacteria	Proteobacteria	Gammaproteobacteria	Enterobacterales	Erwiniaceae	Tatumella	Tatumella saanichensis
1	Bacteria	Proteobacteria	Gammaproteobacteria	Enterobacterales	Erwiniaceae	Tatumella	
1	Bacteria	Proteobacteria	Gammaproteobacteria	Enterobacterales	Erwiniaceae	Pantoea	Pantoea sp. MBLJ3
1	Bacteria	Proteobacteria	Gammaproteobacteria	Enterobacterales	Erwiniaceae	Pantoea	Pantoea sp. AS-PWVM4
1	Bacteria	Proteobacteria	Gammaproteobacteria	Enterobacterales	Erwiniaceae	Pantoea	Pantoea sp. 1.19
1	Bacteria	Proteobacteria	Gammaproteobacteria	Enterobacterales	Erwiniaceae	Pantoea	Pantoea sesami
1	Bacteria	Proteobacteria	Gammaproteobacteria	Enterobacterales	Erwiniaceae	Pantoea	Pantoea dispersa
1	Bacteria	Proteobacteria	Gammaproteobacteria	Enterobacterales	Erwiniaceae	Erwinia	Erwinia toletana
1	Bacteria	Proteobacteria	Gammaproteobacteria	Enterobacterales	Erwiniaceae	Erwinia	Erwinia sp. Leaf53
1	Bacteria	Proteobacteria	Gammaproteobacteria	Enterobacterales	Erwiniaceae	Erwinia	Erwinia piriflorinigrans
1	Bacteria	Proteobacteria	Gammaproteobacteria	Enterobacterales	Erwiniaceae	Erwinia	Erwinia iniecta
1	Bacteria	Proteobacteria	Gammaproteobacteria	Enterobacterales	Erwiniaceae	Erwinia	Candidatus Erwinia sp. ErCipseudotaxifoliae
1	Bacteria	Proteobacteria	Gammaproteobacteria	Enterobacterales	Enterobacteriaceae	unclassified Enterobacteriaceae	unclassified Enterobacteriaceae (miscellaneous)
1	Bacteria	Proteobacteria	Gammaproteobacteria	Enterobacterales	Enterobacteriaceae	unclassified Enterobacteriaceae	unclassified Enterobacteriaceae (miscellaneous)
1	Bacteria	Proteobacteria	Gammaproteobacteria	Enterobacterales	Enterobacteriaceae	unclassified Enterobacteriaceae	ant, tsetse, mealybug, aphid, etc. endosymbionts
1	Bacteria	Proteobacteria	Gammaproteobacteria	Enterobacterales	Enterobacteriaceae	unclassified Enterobacteriaceae	ant, tsetse, mealybug, aphid, etc. endosymbionts
1	Bacteria	Proteobacteria	Gammaproteobacteria	Enterobacterales	Enterobacteriaceae	unclassified Enterobacteriaceae	ant, tsetse, mealybug, aphid, etc. endosymbionts
1	Bacteria	Proteobacteria	Gammaproteobacteria	Enterobacterales	Enterobacteriaceae	unclassified Enterobacteriaceae	ant, tsetse, mealybug, aphid, etc. endosymbionts
1	Bacteria	Proteobacteria	Gammaproteobacteria	Enterobacterales	Enterobacteriaceae	unclassified Enterobacteriaceae	ant, tsetse, mealybug, aphid, etc. endosymbionts
1	Bacteria	Proteobacteria	Gammaproteobacteria	Enterobacterales	Enterobacteriaceae	unclassified Enterobacteriaceae	ant, tsetse, mealybug, aphid, etc. endosymbionts
1	Bacteria	Proteobacteria	Gammaproteobacteria	Enterobacterales	Enterobacteriaceae	unclassified Enterobacteriaceae	ant, tsetse, mealybug, aphid, etc. endosymbionts
1	Bacteria	Proteobacteria	Gammaproteobacteria	Enterobacterales	Enterobacteriaceae	unclassified Enterobacteriaceae	ant, tsetse, mealybug, aphid, etc. endosymbionts
1	Bacteria	Proteobacteria	Gammaproteobacteria	Enterobacterales	Enterobacteriaceae	Trabulsiella	
1	Bacteria	Proteobacteria	Gammaproteobacteria	Enterobacterales	Enterobacteriaceae	Shigella	Shigella flexneri
1	Bacteria	Proteobacteria	Gammaproteobacteria	Enterobacterales	Enterobacteriaceae	Shigella	Shigella boydii
1	Bacteria	Proteobacteria	Gammaproteobacteria	Enterobacterales	Enterobacteriaceae	Shigella	
1	Bacteria	Proteobacteria	Gammaproteobacteria	Enterobacterales	Enterobacteriaceae	Salmonella	Salmonella bongori
1	Bacteria	Proteobacteria	Gammaproteobacteria	Enterobacterales	Enterobacteriaceae	Salmonella	
1	Bacteria	Proteobacteria	Gammaproteobacteria	Enterobacterales	Enterobacteriaceae	Pluralibacter	Pluralibacter gergoviae
1	Bacteria	Proteobacteria	Gammaproteobacteria	Enterobacterales	Enterobacteriaceae	Leclercia	Leclercia adecarboxylata
1	Bacteria	Proteobacteria	Gammaproteobacteria	Enterobacterales	Enterobacteriaceae	Kosakonia	Kosakonia radicincitans
1	Bacteria	Proteobacteria	Gammaproteobacteria	Enterobacterales	Enterobacteriaceae	Kosakonia	Kosakonia cowanii
1	Bacteria	Proteobacteria	Gammaproteobacteria	Enterobacterales	Enterobacteriaceae	Kluyvera	Kluyvera georgiana
1	Bacteria	Proteobacteria	Gammaproteobacteria	Enterobacterales	Enterobacteriaceae	Kluyvera	Kluyvera ascorbata
1	Bacteria	Proteobacteria	Gammaproteobacteria	Enterobacterales	Enterobacteriaceae	Kluyvera	
1	Bacteria	Proteobacteria	Gammaproteobacteria	Enterobacterales	Enterobacteriaceae	Klebsiella	Klebsiella michiganensis
1	Bacteria	Proteobacteria	Gammaproteobacteria	Enterobacterales	Enterobacteriaceae	Franconibacter	Franconibacter pulveris
1	Bacteria	Proteobacteria	Gammaproteobacteria	Enterobacterales	Enterobacteriaceae	Escherichia	Escherichia fergusonii
1	Bacteria	Proteobacteria	Gammaproteobacteria	Enterobacterales	Enterobacteriaceae	Escherichia	Escherichia coli
1	Bacteria	Proteobacteria	Gammaproteobacteria	Enterobacterales	Enterobacteriaceae	Escherichia	Escherichia coli
1	Bacteria	Proteobacteria	Gammaproteobacteria	Enterobacterales	Enterobacteriaceae	Escherichia	Escherichia coli
1	Bacteria	Proteobacteria	Gammaproteobacteria	Enterobacterales	Enterobacteriaceae	Enterobacter	Enterobacter timonensis
1	Bacteria	Proteobacteria	Gammaproteobacteria	Enterobacterales	Enterobacteriaceae	Enterobacter	Enterobacter sp. Sa187
1	Bacteria	Proteobacteria	Gammaproteobacteria	Enterobacterales	Enterobacteriaceae	Enterobacter	Enterobacter cloacae complex
1	Bacteria	Proteobacteria	Gammaproteobacteria	Enterobacterales	Enterobacteriaceae	Citrobacter	Citrobacter freundii complex
1	Bacteria	Proteobacteria	Gammaproteobacteria	Enterobacterales	Enterobacteriaceae	Citrobacter	Citrobacter freundii complex
1	Bacteria	Proteobacteria	Gammaproteobacteria	Enterobacterales	Enterobacteriaceae	Citrobacter	Citrobacter freundii complex
1	Bacteria	Proteobacteria	Gammaproteobacteria	Enterobacterales	Enterobacteriaceae	Citrobacter	Citrobacter europaeus
1	Bacteria	Proteobacteria	Gammaproteobacteria	Enterobacterales	Enterobacteriaceae	Cedecea	Cedecea neteri
1	Bacteria	Proteobacteria	Gammaproteobacteria	Enterobacterales	Enterobacteriaceae	Cedecea	Cedecea davisae
1	Bacteria	Proteobacteria	Gammaproteobacteria	Enterobacterales	Enterobacteriaceae	Buttiauxella	Buttiauxella ferragutiae
1	Bacteria	Proteobacteria	Gammaproteobacteria	Enterobacterales	Enterobacteriaceae	Buttiauxella	Buttiauxella brennerae
1	Bacteria	Proteobacteria	Gammaproteobacteria	Chromatiales	Ectothiorhodospiraceae	Thioalkalivibrio	Thioalkalivibrio sp. ALJ7
1	Bacteria	Proteobacteria	Gammaproteobacteria	Chromatiales	Ectothiorhodospiraceae	Thioalkalivibrio	Thioalkalivibrio sp. ALE20
1	Bacteria	Proteobacteria	Gammaproteobacteria	Chromatiales	Ectothiorhodospiraceae	Thioalkalivibrio	Thioalkalivibrio sp. AKL17
1	Bacteria	Proteobacteria	Gammaproteobacteria	Chromatiales	Ectothiorhodospiraceae	Halorhodospira	environmental samples
1	Bacteria	Proteobacteria	Gammaproteobacteria	Chromatiales	Ectothiorhodospiraceae	Ectothiorhodospira	Ectothiorhodospira sp. PHS-1
1	Bacteria	Proteobacteria	Gammaproteobacteria	Chromatiales	Ectothiorhodospiraceae	Ectothiorhodospira	Ectothiorhodospira magna
1	Bacteria	Proteobacteria	Gammaproteobacteria	Chromatiales	Ectothiorhodospiraceae	Ectothiorhodospira	Ectothiorhodospira haloalkaliphila
1	Bacteria	Proteobacteria	Gammaproteobacteria	Chromatiales	Chromatiaceae	Thioflavicoccus	Thioflavicoccus mobilis
1	Bacteria	Proteobacteria	Gammaproteobacteria	Chromatiales	Chromatiaceae	Rheinheimera	Rheinheimera sp. A13L
1	Bacteria	Proteobacteria	Gammaproteobacteria	Chromatiales	Chromatiaceae	Rheinheimera	Rheinheimera nanhaiensis
1	Bacteria	Proteobacteria	Gammaproteobacteria	Chromatiales	Chromatiaceae	Nitrosococcus	
1	Bacteria	Proteobacteria	Gammaproteobacteria	Chromatiales	Chromatiaceae	Marichromatium	Marichromatium purpuratum
1	Bacteria	Proteobacteria	Gammaproteobacteria	Chromatiales	Chromatiaceae	Allochromatium	
1	Bacteria	Proteobacteria	Gammaproteobacteria	Cellvibrionales	Spongiibacteraceae	Spongiibacter	
1	Bacteria	Proteobacteria	Gammaproteobacteria	Cellvibrionales	Porticoccaceae	unclassified Porticoccaceae	SAR92 clade
1	Bacteria	Proteobacteria	Gammaproteobacteria	Cellvibrionales	Porticoccaceae	unclassified Porticoccaceae	SAR92 clade
1	Bacteria	Proteobacteria	Gammaproteobacteria	Cellvibrionales	Porticoccaceae	unclassified Porticoccaceae	
1	Bacteria	Proteobacteria	Gammaproteobacteria	Cellvibrionales	Microbulbiferaceae	Microbulbifer	Microbulbifer yueqingensis
1	Bacteria	Proteobacteria	Gammaproteobacteria	Cellvibrionales	Halieaceae	Halioglobus	Halioglobus sp. HI00S01
1	Bacteria	Proteobacteria	Gammaproteobacteria	Cellvibrionales	Halieaceae	Congregibacter	Congregibacter litoralis
1	Bacteria	Proteobacteria	Gammaproteobacteria	Cellvibrionales	Cellvibrionaceae	Teredinibacter	Teredinibacter sp. 991H.S.0a.06
1	Bacteria	Proteobacteria	Gammaproteobacteria	Cellvibrionales	Cellvibrionaceae	Teredinibacter	
1	Bacteria	Proteobacteria	Gammaproteobacteria	Cellvibrionales	Cellvibrionaceae	Saccharophagus	environmental samples
1	Bacteria	Proteobacteria	Gammaproteobacteria	Cellvibrionales	Cellvibrionaceae		
1	Bacteria	Proteobacteria	Gammaproteobacteria	Cardiobacteriales	Cardiobacteriaceae	Cardiobacterium	Cardiobacterium valvarum
1	Bacteria	Proteobacteria	Gammaproteobacteria	Cardiobacteriales	Cardiobacteriaceae	Cardiobacterium	
1	Bacteria	Proteobacteria	Gammaproteobacteria	Alteromonadales	Shewanellaceae	Shewanella	Shewanella waksmanii
1	Bacteria	Proteobacteria	Gammaproteobacteria	Alteromonadales	Shewanellaceae	Shewanella	Shewanella violacea
1	Bacteria	Proteobacteria	Gammaproteobacteria	Alteromonadales	Shewanellaceae	Shewanella	Shewanella sp. cp20
1	Bacteria	Proteobacteria	Gammaproteobacteria	Alteromonadales	Shewanellaceae	Shewanella	Shewanella sp. SACH
1	Bacteria	Proteobacteria	Gammaproteobacteria	Alteromonadales	Shewanellaceae	Shewanella	Shewanella sp. POL2
1	Bacteria	Proteobacteria	Gammaproteobacteria	Alteromonadales	Shewanellaceae	Shewanella	Shewanella sp. P1-14-1
1	Bacteria	Proteobacteria	Gammaproteobacteria	Alteromonadales	Shewanellaceae	Shewanella	Shewanella sp. ANA-3
1	Bacteria	Proteobacteria	Gammaproteobacteria	Alteromonadales	Shewanellaceae	Shewanella	Shewanella psychrophila
1	Bacteria	Proteobacteria	Gammaproteobacteria	Alteromonadales	Shewanellaceae	Shewanella	Shewanella piezotolerans
1	Bacteria	Proteobacteria	Gammaproteobacteria	Alteromonadales	Shewanellaceae	Shewanella	Shewanella mangrovi
1	Bacteria	Proteobacteria	Gammaproteobacteria	Alteromonadales	Shewanellaceae	Shewanella	Shewanella loihica
1	Bacteria	Proteobacteria	Gammaproteobacteria	Alteromonadales	Shewanellaceae	Shewanella	Shewanella algae
1	Bacteria	Proteobacteria	Gammaproteobacteria	Alteromonadales	Psychromonadaceae	Psychromonas	Psychromonas sp. PRT-SC03
1	Bacteria	Proteobacteria	Gammaproteobacteria	Alteromonadales	Psychromonadaceae	Psychromonas	Psychromonas sp. CNPT3
1	Bacteria	Proteobacteria	Gammaproteobacteria	Alteromonadales	Psychromonadaceae	Psychromonas	Psychromonas ossibalaenae
1	Bacteria	Proteobacteria	Gammaproteobacteria	Alteromonadales	Psychromonadaceae	Psychromonas	Psychromonas hadalis
1	Bacteria	Proteobacteria	Gammaproteobacteria	Alteromonadales	Pseudoalteromonadaceae	Pseudoalteromonas	Pseudoalteromonas tunicata
1	Bacteria	Proteobacteria	Gammaproteobacteria	Alteromonadales	Pseudoalteromonadaceae	Pseudoalteromonas	Pseudoalteromonas tetraodonis
1	Bacteria	Proteobacteria	Gammaproteobacteria	Alteromonadales	Pseudoalteromonadaceae	Pseudoalteromonas	Pseudoalteromonas sp. P1-9
1	Bacteria	Proteobacteria	Gammaproteobacteria	Alteromonadales	Pseudoalteromonadaceae	Pseudoalteromonas	Pseudoalteromonas sp. P1-26
1	Bacteria	Proteobacteria	Gammaproteobacteria	Alteromonadales	Pseudoalteromonadaceae	Pseudoalteromonas	Pseudoalteromonas sp. H105
1	Bacteria	Proteobacteria	Gammaproteobacteria	Alteromonadales	Pseudoalteromonadaceae	Pseudoalteromonas	Pseudoalteromonas sp. EB27
1	Bacteria	Proteobacteria	Gammaproteobacteria	Alteromonadales	Pseudoalteromonadaceae	Pseudoalteromonas	Pseudoalteromonas sp. BSi20495
1	Bacteria	Proteobacteria	Gammaproteobacteria	Alteromonadales	Pseudoalteromonadaceae	Pseudoalteromonas	Pseudoalteromonas sp. BSi20439
1	Bacteria	Proteobacteria	Gammaproteobacteria	Alteromonadales	Pseudoalteromonadaceae	Pseudoalteromonas	Pseudoalteromonas piscicida
1	Bacteria	Proteobacteria	Gammaproteobacteria	Alteromonadales	Pseudoalteromonadaceae	Pseudoalteromonas	Pseudoalteromonas luteoviolacea
1	Bacteria	Proteobacteria	Gammaproteobacteria	Alteromonadales	Pseudoalteromonadaceae	Pseudoalteromonas	Pseudoalteromonas gelatinilytica
1	Bacteria	Proteobacteria	Gammaproteobacteria	Alteromonadales	Pseudoalteromonadaceae	Pseudoalteromonas	Pseudoalteromonas flavipulchra
1	Bacteria	Proteobacteria	Gammaproteobacteria	Alteromonadales	Pseudoalteromonadaceae	Pseudoalteromonas	Pseudoalteromonas byunsanensis
1	Bacteria	Proteobacteria	Gammaproteobacteria	Alteromonadales	Moritellaceae	Moritella	Moritella sp. JT01
1	Bacteria	Proteobacteria	Gammaproteobacteria	Alteromonadales	Idiomarinaceae	Idiomarina	Idiomarina siamensis
1	Bacteria	Proteobacteria	Gammaproteobacteria	Alteromonadales	Idiomarinaceae	Idiomarina	Idiomarina sp. MD25a
1	Bacteria	Proteobacteria	Gammaproteobacteria	Alteromonadales	Idiomarinaceae	Idiomarina	Idiomarina sp. 28-8
1	Bacteria	Proteobacteria	Gammaproteobacteria	Alteromonadales	Idiomarinaceae	Idiomarina	Idiomarina abyssalis
1	Bacteria	Proteobacteria	Gammaproteobacteria	Alteromonadales	Ferrimonadaceae	Ferrimonas	Ferrimonas sediminum
1	Bacteria	Proteobacteria	Gammaproteobacteria	Alteromonadales	Colwelliaceae	Thalassotalea	
1	Bacteria	Proteobacteria	Gammaproteobacteria	Alteromonadales	Colwelliaceae	Colwellia	Colwellia sp. UCD-KL20
1	Bacteria	Proteobacteria	Gammaproteobacteria	Alteromonadales	Colwelliaceae	Colwellia	Colwellia sp. TT2012
1	Bacteria	Proteobacteria	Gammaproteobacteria	Alteromonadales	Colwelliaceae	Colwellia	Colwellia sp. NB097-1
1	Bacteria	Proteobacteria	Gammaproteobacteria	Alteromonadales	Colwelliaceae	Colwellia	Colwellia sp. MT41
1	Bacteria	Proteobacteria	Gammaproteobacteria	Alteromonadales	Colwelliaceae	Colwellia	Colwellia sediminilitoris
1	Bacteria	Proteobacteria	Gammaproteobacteria	Alteromonadales	Alteromonadaceae	Paraglaciecola	Paraglaciecola mesophila
1	Bacteria	Proteobacteria	Gammaproteobacteria	Alteromonadales	Alteromonadaceae	Paraglaciecola	Paraglaciecola chathamensis
1	Bacteria	Proteobacteria	Gammaproteobacteria	Alteromonadales	Alteromonadaceae	Paraglaciecola	
1	Bacteria	Proteobacteria	Gammaproteobacteria	Alteromonadales	Alteromonadaceae	Marinobacter	Marinobacter subterrani
1	Bacteria	Proteobacteria	Gammaproteobacteria	Alteromonadales	Alteromonadaceae	Marinobacter	Marinobacter sp. LQ44
1	Bacteria	Proteobacteria	Gammaproteobacteria	Alteromonadales	Alteromonadaceae	Marinobacter	Marinobacter sp. EN3
1	Bacteria	Proteobacteria	Gammaproteobacteria	Alteromonadales	Alteromonadaceae	Marinobacter	Marinobacter salarius
1	Bacteria	Proteobacteria	Gammaproteobacteria	Alteromonadales	Alteromonadaceae	Marinobacter	Marinobacter pelagius
1	Bacteria	Proteobacteria	Gammaproteobacteria	Alteromonadales	Alteromonadaceae	Marinobacter	Marinobacter lutaoensis
1	Bacteria	Proteobacteria	Gammaproteobacteria	Alteromonadales	Alteromonadaceae	Marinobacter	Marinobacter lipolyticus
1	Bacteria	Proteobacteria	Gammaproteobacteria	Alteromonadales	Alteromonadaceae	Marinobacter	Marinobacter hydrocarbonoclasticus
1	Bacteria	Proteobacteria	Gammaproteobacteria	Alteromonadales	Alteromonadaceae	Marinobacter	Marinobacter daepoensis
1	Bacteria	Proteobacteria	Gammaproteobacteria	Alteromonadales	Alteromonadaceae	Marinobacter	Marinobacter adhaerens
1	Bacteria	Proteobacteria	Gammaproteobacteria	Alteromonadales	Alteromonadaceae	Glaciecola	
1	Bacteria	Proteobacteria	Gammaproteobacteria	Alteromonadales	Alteromonadaceae	Catenovulum	Catenovulum maritimum
1	Bacteria	Proteobacteria	Gammaproteobacteria	Alteromonadales	Alteromonadaceae	Alteromonas	Alteromonas sp. RW2A1
1	Bacteria	Proteobacteria	Gammaproteobacteria	Alteromonadales	Alteromonadaceae	Alteromonas	Alteromonas sp. Nap_26
1	Bacteria	Proteobacteria	Gammaproteobacteria	Alteromonadales	Alteromonadaceae	Alishewanella	Alishewanella jeotgali
1	Bacteria	Proteobacteria	Gammaproteobacteria	Alteromonadales	Alteromonadaceae	Alishewanella	Alishewanella agri
1	Bacteria	Proteobacteria	Gammaproteobacteria	Alteromonadales	Alteromonadaceae	Alishewanella	Alishewanella aestuarii
1	Bacteria	Proteobacteria	Gammaproteobacteria	Alteromonadales	Alteromonadaceae	Aliagarivorans	Aliagarivorans marinus
1	Bacteria	Proteobacteria	Gammaproteobacteria	Alteromonadales	Alteromonadaceae	Aliagarivorans	
1	Bacteria	Proteobacteria	Gammaproteobacteria	Alteromonadales	Alteromonadaceae	Agarivorans	Agarivorans gilvus
1	Bacteria	Proteobacteria	Gammaproteobacteria	Alteromonadales	Alteromonadaceae	Aestuariibacter	
1	Bacteria	Proteobacteria	Gammaproteobacteria	Aeromonadales	Succinivibrionaceae	Succinatimonas	environmental samples
1	Bacteria	Proteobacteria	Gammaproteobacteria	Aeromonadales	Succinivibrionaceae	Ruminobacter	Ruminobacter sp. RM87
1	Bacteria	Proteobacteria	Gammaproteobacteria	Aeromonadales	Succinivibrionaceae		
1	Bacteria	Proteobacteria	Gammaproteobacteria	Aeromonadales	Aeromonadaceae	Tolumonas	Tolumonas lignilytica
1	Bacteria	Proteobacteria	Gammaproteobacteria	Aeromonadales	Aeromonadaceae	Oceanimonas	
1	Bacteria	Proteobacteria	Gammaproteobacteria	Aeromonadales	Aeromonadaceae	Aeromonas	Aeromonas veronii
1	Bacteria	Proteobacteria	Gammaproteobacteria	Aeromonadales	Aeromonadaceae	Aeromonas	Aeromonas tecta
1	Bacteria	Proteobacteria	Gammaproteobacteria	Aeromonadales	Aeromonadaceae	Aeromonas	Aeromonas sp. ZOR0002
1	Bacteria	Proteobacteria	Gammaproteobacteria	Aeromonadales	Aeromonadaceae	Aeromonas	Aeromonas sp. SCS5
1	Bacteria	Proteobacteria	Gammaproteobacteria	Aeromonadales	Aeromonadaceae	Aeromonas	Aeromonas sp. L_1B5_3
1	Bacteria	Proteobacteria	Gammaproteobacteria	Aeromonadales	Aeromonadaceae	Aeromonas	Aeromonas schubertii
1	Bacteria	Proteobacteria	Gammaproteobacteria	Aeromonadales	Aeromonadaceae	Aeromonas	Aeromonas piscicola
1	Bacteria	Proteobacteria	Gammaproteobacteria	Aeromonadales	Aeromonadaceae	Aeromonas	Aeromonas media
1	Bacteria	Proteobacteria	Gammaproteobacteria	Aeromonadales	Aeromonadaceae	Aeromonas	Aeromonas jandaei
1	Bacteria	Proteobacteria	Gammaproteobacteria	Aeromonadales	Aeromonadaceae	Aeromonas	Aeromonas hydrophila
1	Bacteria	Proteobacteria	Gammaproteobacteria	Aeromonadales	Aeromonadaceae	Aeromonas	Aeromonas enteropelogenes
1	Bacteria	Proteobacteria	Gammaproteobacteria	Aeromonadales	Aeromonadaceae	Aeromonas	Aeromonas diversa
1	Bacteria	Proteobacteria	Gammaproteobacteria	Aeromonadales	Aeromonadaceae		
1	Bacteria	Proteobacteria	Candidatus Muproteobacteria	Candidatus Muproteobacteria bacterium RIFCSPHIGHO2_02_FULL_65_16			
1	Bacteria	Proteobacteria	Candidatus Muproteobacteria	Candidatus Muproteobacteria bacterium RIFCSPHIGHO2_01_FULL_65_16			
1	Bacteria	Proteobacteria	Candidatus Muproteobacteria	Candidatus Muproteobacteria bacterium RIFCSPHIGHO2_01_60_12			
1	Bacteria	Proteobacteria	Betaproteobacteria	unclassified Betaproteobacteria	unclassified Betaproteobacteria (miscellaneous)	beta proteobacterium FWI2	
1	Bacteria	Proteobacteria	Betaproteobacteria	unclassified Betaproteobacteria	unclassified Betaproteobacteria (miscellaneous)	beta proteobacterium AAP65	
1	Bacteria	Proteobacteria	Betaproteobacteria	unclassified Betaproteobacteria	unclassified Betaproteobacteria (miscellaneous)	Betaproteobacteria bacterium RIFCSPLOWO2_12_FULL_65_110	
1	Bacteria	Proteobacteria	Betaproteobacteria	unclassified Betaproteobacteria	unclassified Betaproteobacteria (miscellaneous)	Betaproteobacteria bacterium RIFCSPHIGHO2_12_FULL_69_13	
1	Bacteria	Proteobacteria	Betaproteobacteria	unclassified Betaproteobacteria	unclassified Betaproteobacteria (miscellaneous)	Betaproteobacteria bacterium RBG_16_64_9	
1	Bacteria	Proteobacteria	Betaproteobacteria	unclassified Betaproteobacteria	Kinetoplastibacterium	Kinetoplastibacterium blastocrithidii	
1	Bacteria	Proteobacteria	Betaproteobacteria	unclassified Betaproteobacteria	Kinetoplastibacterium	Candidatus Kinetoplastibacterium oncopeltii	
1	Bacteria	Proteobacteria	Betaproteobacteria	Rhodocyclales	Rhodocyclaceae	Thauera	Thauera humireducens
1	Bacteria	Proteobacteria	Betaproteobacteria	Rhodocyclales	Rhodocyclaceae	Thauera	Thauera aminoaromatica
1	Bacteria	Proteobacteria	Betaproteobacteria	Rhodocyclales	Rhodocyclaceae	Dechloromonas	Dechloromonas aromatica
1	Bacteria	Proteobacteria	Betaproteobacteria	Rhodocyclales	Rhodocyclaceae	Azospira	Azospira oryzae
1	Bacteria	Proteobacteria	Betaproteobacteria	Rhodocyclales	Rhodocyclaceae	Azoarcus	Azoarcus olearius
1	Bacteria	Proteobacteria	Betaproteobacteria	Nitrosomonadales	Nitrosomonadaceae	Nitrosospira	Nitrosospira sp. Nsp18
1	Bacteria	Proteobacteria	Betaproteobacteria	Neisseriales	Neisseriaceae	Vitreoscilla	
1	Bacteria	Proteobacteria	Betaproteobacteria	Neisseriales	Neisseriaceae	Snodgrassella	Snodgrassella alvi
1	Bacteria	Proteobacteria	Betaproteobacteria	Neisseriales	Neisseriaceae	Neisseria	Neisseria zoodegmatis
1	Bacteria	Proteobacteria	Betaproteobacteria	Neisseriales	Neisseriaceae	Neisseria	Neisseria sp. oral taxon 014
1	Bacteria	Proteobacteria	Betaproteobacteria	Neisseriales	Neisseriaceae	Neisseria	Neisseria sp. HMSC055F11
1	Bacteria	Proteobacteria	Betaproteobacteria	Neisseriales	Neisseriaceae	Neisseria	Neisseria shayeganii
1	Bacteria	Proteobacteria	Betaproteobacteria	Neisseriales	Neisseriaceae	Neisseria	Neisseria meningitidis
1	Bacteria	Proteobacteria	Betaproteobacteria	Neisseriales	Neisseriaceae	Neisseria	Neisseria lactamica
1	Bacteria	Proteobacteria	Betaproteobacteria	Neisseriales	Neisseriaceae	Neisseria	Neisseria flavescens
1	Bacteria	Proteobacteria	Betaproteobacteria	Neisseriales	Neisseriaceae	Neisseria	Neisseria cinerea
1	Bacteria	Proteobacteria	Betaproteobacteria	Neisseriales	Neisseriaceae	Neisseria	Neisseria canis
1	Bacteria	Proteobacteria	Betaproteobacteria	Neisseriales	Neisseriaceae	Neisseria	Neisseria animaloris
1	Bacteria	Proteobacteria	Betaproteobacteria	Neisseriales	Neisseriaceae	Eikenella	Eikenella sp. NML130454
1	Bacteria	Proteobacteria	Betaproteobacteria	Neisseriales	Neisseriaceae	Eikenella	Eikenella sp. NML02-A-017
1	Bacteria	Proteobacteria	Betaproteobacteria	Neisseriales	Neisseriaceae	Conchiformibius	
1	Bacteria	Proteobacteria	Betaproteobacteria	Neisseriales	Chromobacteriaceae	Chromobacterium group	Chromobacterium
1	Bacteria	Proteobacteria	Betaproteobacteria	Neisseriales	Chromobacteriaceae	Chromobacterium group	Chromobacterium
1	Bacteria	Proteobacteria	Betaproteobacteria	Methylophilales	unclassified Methylophilales	OM43 clade	beta proteobacterium KB13
1	Bacteria	Proteobacteria	Betaproteobacteria	Methylophilales	unclassified Methylophilales	OM43 clade	Methylophilales bacterium HTCC2181
1	Bacteria	Proteobacteria	Betaproteobacteria	Methylophilales	unclassified Methylophilales	Methylophilales bacterium BACL14 MAG-120910-bin43	
1	Bacteria	Proteobacteria	Betaproteobacteria	Methylophilales	Methylophilaceae	Methylotenera	Methylotenera versatilis
1	Bacteria	Proteobacteria	Betaproteobacteria	Hydrogenophilales	Hydrogenophilaceae	Thiobacillus	Thiobacillus sp. SCN 64-317
1	Bacteria	Proteobacteria	Betaproteobacteria	Hydrogenophilales	Hydrogenophilaceae	Hydrogenophilus	Hydrogenophilus thermoluteolus
1	Bacteria	Proteobacteria	Betaproteobacteria	Gallionellales	Gallionellaceae	unclassified Gallionellaceae	Gallionellaceae bacterium CG1_02_60_948
1	Bacteria	Proteobacteria	Betaproteobacteria	Gallionellales	Gallionellaceae	Sideroxydans	Sideroxydans sp. RIFOXYB12_FULL_59_6
1	Bacteria	Proteobacteria	Betaproteobacteria	Gallionellales	Gallionellaceae	Sideroxydans	Sideroxydans lithotrophicus
1	Bacteria	Proteobacteria	Betaproteobacteria	Gallionellales	Gallionellaceae	Sideroxydans	
1	Bacteria	Proteobacteria	Betaproteobacteria	Ferrovales	Ferrovaceae	Ferrovum	Ferrovum sp. Z-31
1	Bacteria	Proteobacteria	Betaproteobacteria	Burkholderiales	unclassified Burkholderiales	unclassified Burkholderiales (miscellaneous)	Burkholderiales bacterium RIFOXYD2_FULL_59_8
1	Bacteria	Proteobacteria	Betaproteobacteria	Burkholderiales	unclassified Burkholderiales	unclassified Burkholderiales (miscellaneous)	Burkholderiales bacterium 66-26
1	Bacteria	Proteobacteria	Betaproteobacteria	Burkholderiales	unclassified Burkholderiales	unclassified Burkholderiales (miscellaneous)	Burkholderiales bacterium 64-34
1	Bacteria	Proteobacteria	Betaproteobacteria	Burkholderiales	unclassified Burkholderiales	Burkholderiales Genera incertae sedis	Thiomonas
1	Bacteria	Proteobacteria	Betaproteobacteria	Burkholderiales	unclassified Burkholderiales	Burkholderiales Genera incertae sedis	Thiomonas
1	Bacteria	Proteobacteria	Betaproteobacteria	Burkholderiales	unclassified Burkholderiales	Burkholderiales Genera incertae sedis	Thiomonas
1	Bacteria	Proteobacteria	Betaproteobacteria	Burkholderiales	unclassified Burkholderiales	Burkholderiales Genera incertae sedis	Rubrivivax
1	Bacteria	Proteobacteria	Betaproteobacteria	Burkholderiales	unclassified Burkholderiales	Burkholderiales Genera incertae sedis	Rubrivivax
1	Bacteria	Proteobacteria	Betaproteobacteria	Burkholderiales	unclassified Burkholderiales	Burkholderiales Genera incertae sedis	Methylibium
1	Bacteria	Proteobacteria	Betaproteobacteria	Burkholderiales	unclassified Burkholderiales	Burkholderiales Genera incertae sedis	Methylibium
1	Bacteria	Proteobacteria	Betaproteobacteria	Burkholderiales	unclassified Burkholderiales	Burkholderiales Genera incertae sedis	Leptothrix
1	Bacteria	Proteobacteria	Betaproteobacteria	Burkholderiales	unclassified Burkholderiales	Burkholderiales Genera incertae sedis	Ideonella
1	Bacteria	Proteobacteria	Betaproteobacteria	Burkholderiales	Sutterellaceae	Sutterella	environmental samples
1	Bacteria	Proteobacteria	Betaproteobacteria	Burkholderiales	Sutterellaceae	Sutterella	Sutterella sp. 54_7
1	Bacteria	Proteobacteria	Betaproteobacteria	Burkholderiales	Sutterellaceae	Sutterella	Sutterella parvirubra
1	Bacteria	Proteobacteria	Betaproteobacteria	Burkholderiales	Sutterellaceae	Parasutterella	
1	Bacteria	Proteobacteria	Betaproteobacteria	Burkholderiales	Sutterellaceae	Duodenibacillus	Duodenibacillus massiliensis
1	Bacteria	Proteobacteria	Betaproteobacteria	Burkholderiales	Oxalobacteraceae	Massilia	Massilia timonae
1	Bacteria	Proteobacteria	Betaproteobacteria	Burkholderiales	Oxalobacteraceae	Janthinobacterium	Janthinobacterium sp. RA13
1	Bacteria	Proteobacteria	Betaproteobacteria	Burkholderiales	Oxalobacteraceae	Janthinobacterium	Janthinobacterium sp. OK676
1	Bacteria	Proteobacteria	Betaproteobacteria	Burkholderiales	Oxalobacteraceae	Janthinobacterium	Janthinobacterium agaricidamnosum
1	Bacteria	Proteobacteria	Betaproteobacteria	Burkholderiales	Oxalobacteraceae	Herbaspirillum	Herbaspirillum sp. B65
1	Bacteria	Proteobacteria	Betaproteobacteria	Burkholderiales	Oxalobacteraceae	Herbaspirillum	Herbaspirillum sp. B501
1	Bacteria	Proteobacteria	Betaproteobacteria	Burkholderiales	Oxalobacteraceae	Herbaspirillum	Herbaspirillum seropedicae
1	Bacteria	Proteobacteria	Betaproteobacteria	Burkholderiales	Oxalobacteraceae	Duganella	Duganella sp. Root336D2
1	Bacteria	Proteobacteria	Betaproteobacteria	Burkholderiales	Oxalobacteraceae	Candidatus Zinderia	Candidatus Zinderia insecticola
1	Bacteria	Proteobacteria	Betaproteobacteria	Burkholderiales	Comamonadaceae	Variovorax	Variovorax sp. JS669
1	Bacteria	Proteobacteria	Betaproteobacteria	Burkholderiales	Comamonadaceae	Rhodoferax	Rhodoferax ferrireducens
1	Bacteria	Proteobacteria	Betaproteobacteria	Burkholderiales	Comamonadaceae	Ramlibacter	Ramlibacter tataouinensis
1	Bacteria	Proteobacteria	Betaproteobacteria	Burkholderiales	Comamonadaceae	Lampropedia	
1	Bacteria	Proteobacteria	Betaproteobacteria	Burkholderiales	Comamonadaceae	Delftia	environmental samples
1	Bacteria	Proteobacteria	Betaproteobacteria	Burkholderiales	Comamonadaceae	Delftia	Delftia sp. JD2
1	Bacteria	Proteobacteria	Betaproteobacteria	Burkholderiales	Comamonadaceae	Delftia	Delftia sp. GW456-R20
1	Bacteria	Proteobacteria	Betaproteobacteria	Burkholderiales	Comamonadaceae	Delftia	Delftia sp. 67-8
1	Bacteria	Proteobacteria	Betaproteobacteria	Burkholderiales	Comamonadaceae	Comamonas	Comamonas testosteroni
1	Bacteria	Proteobacteria	Betaproteobacteria	Burkholderiales	Comamonadaceae	Comamonas	Comamonas sp. SFCD1
1	Bacteria	Proteobacteria	Betaproteobacteria	Burkholderiales	Comamonadaceae	Comamonas	Comamonas sp. SCN 67-35
1	Bacteria	Proteobacteria	Betaproteobacteria	Burkholderiales	Comamonadaceae	Alicycliphilus	Alicycliphilus denitrificans
1	Bacteria	Proteobacteria	Betaproteobacteria	Burkholderiales	Comamonadaceae	Acidovorax	Acidovorax sp. Root275
1	Bacteria	Proteobacteria	Betaproteobacteria	Burkholderiales	Comamonadaceae	Acidovorax	Acidovorax sp. Root267
1	Bacteria	Proteobacteria	Betaproteobacteria	Burkholderiales	Comamonadaceae	Acidovorax	Acidovorax sp. 65-7
1	Bacteria	Proteobacteria	Betaproteobacteria	Burkholderiales	Comamonadaceae	Acidovorax	Acidovorax radicis
1	Bacteria	Proteobacteria	Betaproteobacteria	Burkholderiales	Comamonadaceae	Acidovorax	Acidovorax ebreus
1	Bacteria	Proteobacteria	Betaproteobacteria	Burkholderiales	Burkholderiaceae	unclassified Burkholderiaceae	bacterium endosymbiont of Mortierella elongata FMR23-6
1	Bacteria	Proteobacteria	Betaproteobacteria	Burkholderiales	Burkholderiaceae	Ralstonia	Ralstonia sp. NT80
1	Bacteria	Proteobacteria	Betaproteobacteria	Burkholderiales	Burkholderiaceae	Ralstonia	Ralstonia sp. NFACC01
1	Bacteria	Proteobacteria	Betaproteobacteria	Burkholderiales	Burkholderiaceae	Ralstonia	Ralstonia solanacearum
1	Bacteria	Proteobacteria	Betaproteobacteria	Burkholderiales	Burkholderiaceae	Ralstonia	Ralstonia pickettii
1	Bacteria	Proteobacteria	Betaproteobacteria	Burkholderiales	Burkholderiaceae	Polynucleobacter	Polynucleobacter sp. QLW-P1DATA-2
1	Bacteria	Proteobacteria	Betaproteobacteria	Burkholderiales	Burkholderiaceae	Polynucleobacter	Polynucleobacter necessarius
1	Bacteria	Proteobacteria	Betaproteobacteria	Burkholderiales	Burkholderiaceae	Paraburkholderia	Paraburkholderia xenovorans
1	Bacteria	Proteobacteria	Betaproteobacteria	Burkholderiales	Burkholderiaceae	Paraburkholderia	Paraburkholderia terrae
1	Bacteria	Proteobacteria	Betaproteobacteria	Burkholderiales	Burkholderiaceae	Paraburkholderia	Paraburkholderia terrae
1	Bacteria	Proteobacteria	Betaproteobacteria	Burkholderiales	Burkholderiaceae	Paraburkholderia	Paraburkholderia sartisoli
1	Bacteria	Proteobacteria	Betaproteobacteria	Burkholderiales	Burkholderiaceae	Paraburkholderia	Paraburkholderia phymatum
1	Bacteria	Proteobacteria	Betaproteobacteria	Burkholderiales	Burkholderiaceae	Paraburkholderia	Candidatus Paraburkholderia schumannianae
1	Bacteria	Proteobacteria	Betaproteobacteria	Burkholderiales	Burkholderiaceae	Pandoraea	Pandoraea sp. SD6-2
1	Bacteria	Proteobacteria	Betaproteobacteria	Burkholderiales	Burkholderiaceae	Lautropia	
1	Bacteria	Proteobacteria	Betaproteobacteria	Burkholderiales	Burkholderiaceae	Cupriavidus	Cupriavidus sp. WS
1	Bacteria	Proteobacteria	Betaproteobacteria	Burkholderiales	Burkholderiaceae	Cupriavidus	Cupriavidus sp. USMAA1020
1	Bacteria	Proteobacteria	Betaproteobacteria	Burkholderiales	Burkholderiaceae	Cupriavidus	Cupriavidus oxalaticus
1	Bacteria	Proteobacteria	Betaproteobacteria	Burkholderiales	Burkholderiaceae	Cupriavidus	Cupriavidus necator
1	Bacteria	Proteobacteria	Betaproteobacteria	Burkholderiales	Burkholderiaceae	Cupriavidus	Cupriavidus metallidurans
1	Bacteria	Proteobacteria	Betaproteobacteria	Burkholderiales	Burkholderiaceae	Cupriavidus	Cupriavidus alkaliphilus
1	Bacteria	Proteobacteria	Betaproteobacteria	Burkholderiales	Burkholderiaceae	Caballeronia	Caballeronia zhejiangensis
1	Bacteria	Proteobacteria	Betaproteobacteria	Burkholderiales	Burkholderiaceae	Caballeronia	Caballeronia telluris
1	Bacteria	Proteobacteria	Betaproteobacteria	Burkholderiales	Burkholderiaceae	Caballeronia	Caballeronia jiangsuensis
1	Bacteria	Proteobacteria	Betaproteobacteria	Burkholderiales	Burkholderiaceae	Caballeronia	Caballeronia cordobensis
1	Bacteria	Proteobacteria	Betaproteobacteria	Burkholderiales	Burkholderiaceae	Burkholderia	pseudomallei group
1	Bacteria	Proteobacteria	Betaproteobacteria	Burkholderiales	Burkholderiaceae	Burkholderia	pseudomallei group
1	Bacteria	Proteobacteria	Betaproteobacteria	Burkholderiales	Burkholderiaceae	Burkholderia	pseudomallei group
1	Bacteria	Proteobacteria	Betaproteobacteria	Burkholderiales	Burkholderiaceae	Burkholderia	pseudomallei group
1	Bacteria	Proteobacteria	Betaproteobacteria	Burkholderiales	Burkholderiaceae	Burkholderia	pseudomallei group
1	Bacteria	Proteobacteria	Betaproteobacteria	Burkholderiales	Burkholderiaceae	Burkholderia	pseudomallei group
1	Bacteria	Proteobacteria	Betaproteobacteria	Burkholderiales	Burkholderiaceae	Burkholderia	pseudomallei group
1	Bacteria	Proteobacteria	Betaproteobacteria	Burkholderiales	Burkholderiaceae	Burkholderia	[Pseudomonas] mesoacidophila
1	Bacteria	Proteobacteria	Betaproteobacteria	Burkholderiales	Burkholderiaceae	Burkholderia	Candidatus Burkholderia crenata
1	Bacteria	Proteobacteria	Betaproteobacteria	Burkholderiales	Burkholderiaceae	Burkholderia	Burkholderia sp. b14
1	Bacteria	Proteobacteria	Betaproteobacteria	Burkholderiales	Burkholderiaceae	Burkholderia	Burkholderia sp. WP40
1	Bacteria	Proteobacteria	Betaproteobacteria	Burkholderiales	Burkholderiaceae	Burkholderia	Burkholderia sp. ST111
1	Bacteria	Proteobacteria	Betaproteobacteria	Burkholderiales	Burkholderiaceae	Burkholderia	Burkholderia sp. PAMC 28687
1	Bacteria	Proteobacteria	Betaproteobacteria	Burkholderiales	Burkholderiaceae	Burkholderia	Burkholderia sp. HMSC10F09
1	Bacteria	Proteobacteria	Betaproteobacteria	Burkholderiales	Burkholderiaceae	Burkholderia	Burkholderia sp. HB1
1	Bacteria	Proteobacteria	Betaproteobacteria	Burkholderiales	Burkholderiaceae	Burkholderia	Burkholderia sp. DNA89
1	Bacteria	Proteobacteria	Betaproteobacteria	Burkholderiales	Burkholderiaceae	Burkholderia	Burkholderia sp. Bp7605
1	Bacteria	Proteobacteria	Betaproteobacteria	Burkholderiales	Burkholderiaceae	Burkholderia	Burkholderia glumae
1	Bacteria	Proteobacteria	Betaproteobacteria	Burkholderiales	Burkholderiaceae	Burkholderia	Burkholderia fortuita
1	Bacteria	Proteobacteria	Betaproteobacteria	Burkholderiales	Burkholderiaceae	Burkholderia	Burkholderia cepacia complex
1	Bacteria	Proteobacteria	Betaproteobacteria	Burkholderiales	Burkholderiaceae	Burkholderia	Burkholderia cepacia complex
1	Bacteria	Proteobacteria	Betaproteobacteria	Burkholderiales	Burkholderiaceae	Burkholderia	Burkholderia cepacia complex
1	Bacteria	Proteobacteria	Betaproteobacteria	Burkholderiales	Burkholderiaceae	Burkholderia	Burkholderia cepacia complex
1	Bacteria	Proteobacteria	Betaproteobacteria	Burkholderiales	Burkholderiaceae	Burkholderia	Burkholderia cepacia complex
1	Bacteria	Proteobacteria	Betaproteobacteria	Burkholderiales	Burkholderiaceae	Burkholderia	Burkholderia cepacia complex
1	Bacteria	Proteobacteria	Betaproteobacteria	Burkholderiales	Burkholderiaceae	Burkholderia	Burkholderia cepacia complex
1	Bacteria	Proteobacteria	Betaproteobacteria	Burkholderiales	Burkholderiaceae	Burkholderia	Burkholderia cepacia complex
1	Bacteria	Proteobacteria	Betaproteobacteria	Burkholderiales	Burkholderiaceae	Burkholderia	Burkholderia cepacia complex
1	Bacteria	Proteobacteria	Betaproteobacteria	Burkholderiales	Burkholderiaceae	Burkholderia	Burkholderia cepacia complex
1	Bacteria	Proteobacteria	Betaproteobacteria	Burkholderiales	Alcaligenaceae	Pelistega	Pelistega indica
1	Bacteria	Proteobacteria	Betaproteobacteria	Burkholderiales	Alcaligenaceae	Castellaniella	Castellaniella defragrans
1	Bacteria	Proteobacteria	Betaproteobacteria	Burkholderiales	Alcaligenaceae	Castellaniella	
1	Bacteria	Proteobacteria	Betaproteobacteria	Burkholderiales	Alcaligenaceae	Bordetella	Bordetella sp. SCN 68-11
1	Bacteria	Proteobacteria	Betaproteobacteria	Burkholderiales	Alcaligenaceae	Bordetella	Bordetella hinzii
1	Bacteria	Proteobacteria	Betaproteobacteria	Burkholderiales	Alcaligenaceae	Advenella	Advenella kashmirensis
1	Bacteria	Proteobacteria	Betaproteobacteria	Burkholderiales	Alcaligenaceae	Achromobacter	Achromobacter sp. Root565
1	Bacteria	Proteobacteria	Betaproteobacteria	Burkholderiales	Alcaligenaceae	Achromobacter	Achromobacter sp. MFA1 R4
1	Bacteria	Proteobacteria	Betaproteobacteria	Burkholderiales	Alcaligenaceae	Achromobacter	Achromobacter sp. HMSC15D03
1	Bacteria	Proteobacteria	Betaproteobacteria	Burkholderiales	Alcaligenaceae	Achromobacter	Achromobacter sp. 2789STDY5608633
1	Bacteria	Proteobacteria	Betaproteobacteria	Burkholderiales	Alcaligenaceae	Achromobacter	Achromobacter sp. 2789STDY5608623
1	Bacteria	Proteobacteria	Betaproteobacteria	Burkholderiales	Alcaligenaceae	Achromobacter	Achromobacter sp. 2789STDY5608621
1	Bacteria	Proteobacteria	Betaproteobacteria	Burkholderiales	Alcaligenaceae	Achromobacter	Achromobacter insuavis
1	Bacteria	Proteobacteria	Alphaproteobacteria	unclassified Alphaproteobacteria	unclassified Alphaproteobacteria (miscellaneous)	alpha proteobacterium LLX12A	
1	Bacteria	Proteobacteria	Alphaproteobacteria	unclassified Alphaproteobacteria	unclassified Alphaproteobacteria (miscellaneous)	Alphaproteobacteria bacterium RIFCSPHIGHO2_12_FULL_45_9	
1	Bacteria	Proteobacteria	Alphaproteobacteria	environmental samples	uncultured alpha proteobacterium HF0130_20P23		
1	Bacteria	Proteobacteria	Alphaproteobacteria	environmental samples	uncultured alpha proteobacterium HF0070_14E07		
1	Bacteria	Proteobacteria	Alphaproteobacteria	environmental samples	uncultured alpha proteobacterium EB080_L58F04		
1	Bacteria	Proteobacteria	Alphaproteobacteria	Sphingomonadales	environmental samples	uncultured Sphingomonadales bacterium HF0500_24B12	
1	Bacteria	Proteobacteria	Alphaproteobacteria	Sphingomonadales	Sphingomonadaceae	Zymomonas	Zymomonas mobilis
1	Bacteria	Proteobacteria	Alphaproteobacteria	Sphingomonadales	Sphingomonadaceae	Sphingopyxis	Sphingopyxis sp. MB-E
1	Bacteria	Proteobacteria	Alphaproteobacteria	Sphingomonadales	Sphingomonadaceae	Sphingopyxis	Sphingopyxis alaskensis
1	Bacteria	Proteobacteria	Alphaproteobacteria	Sphingomonadales	Sphingomonadaceae	Sphingomonas	Sphingomonas sp. Leaf38
1	Bacteria	Proteobacteria	Alphaproteobacteria	Sphingomonadales	Sphingomonadaceae	Sphingomonas	Sphingomonas sp. Leaf242
1	Bacteria	Proteobacteria	Alphaproteobacteria	Sphingomonadales	Sphingomonadaceae	Sphingomonas	Sphingomonas sp. Leaf23
1	Bacteria	Proteobacteria	Alphaproteobacteria	Sphingomonadales	Sphingomonadaceae	Sphingomonas	Sphingomonas sp. Leaf22
1	Bacteria	Proteobacteria	Alphaproteobacteria	Sphingomonadales	Sphingomonadaceae	Sphingomonas	Sphingomonas sp. Ant20
1	Bacteria	Proteobacteria	Alphaproteobacteria	Sphingomonadales	Sphingomonadaceae	Sphingomonas	Sphingomonas sp. A1
1	Bacteria	Proteobacteria	Alphaproteobacteria	Sphingomonadales	Sphingomonadaceae	Sphingomonas	Sphingomonas sp. 133
1	Bacteria	Proteobacteria	Alphaproteobacteria	Sphingomonadales	Sphingomonadaceae	Sphingomonas	Sphingomonas sp. 056
1	Bacteria	Proteobacteria	Alphaproteobacteria	Sphingomonadales	Sphingomonadaceae	Sphingomonas	Sphingomonas changbaiensis

[illegible]	[illegible]	[illegible]	[illegible]	[illegible]	[illegible]	[illegible]

1	Bacteria	FCB group	Bacteroidetes/Chlorobi group	Bacteroidetes	Flavobacteriia	Flavobacteriales	Flavobacteriaceae
1	Bacteria	FCB group	Bacteroidetes/Chlorobi group	Bacteroidetes	Flavobacteriia	Flavobacteriales	Flavobacteriaceae
1	Bacteria	FCB group	Bacteroidetes/Chlorobi group	Bacteroidetes	Flavobacteriia	Flavobacteriales	Flavobacteriaceae
1	Bacteria	FCB group	Bacteroidetes/Chlorobi group	Bacteroidetes	Flavobacteriia	Flavobacteriales	Flavobacteriaceae
1	Bacteria	FCB group	Bacteroidetes/Chlorobi group	Bacteroidetes	Flavobacteriia	Flavobacteriales	Flavobacteriaceae
1	Bacteria	FCB group	Bacteroidetes/Chlorobi group	Bacteroidetes	Flavobacteriia	Flavobacteriales	Flavobacteriaceae
1	Bacteria	FCB group	Bacteroidetes/Chlorobi group	Bacteroidetes	Flavobacteriia	Flavobacteriales	Flavobacteriaceae
1	Bacteria	FCB group	Bacteroidetes/Chlorobi group	Bacteroidetes	Flavobacteriia	Flavobacteriales	Flavobacteriaceae
1	Bacteria	FCB group	Bacteroidetes/Chlorobi group	Bacteroidetes	Flavobacteriia	Flavobacteriales	Flavobacteriaceae
1	Bacteria	FCB group	Bacteroidetes/Chlorobi group	Bacteroidetes	Flavobacteriia	Flavobacteriales	Flavobacteriaceae
1	Bacteria	FCB group	Bacteroidetes/Chlorobi group	Bacteroidetes	Flavobacteriia	Flavobacteriales	Flavobacteriaceae
1	Bacteria	FCB group	Bacteroidetes/Chlorobi group	Bacteroidetes	Flavobacteriia	Flavobacteriales	Flavobacteriaceae
1	Bacteria	FCB group	Bacteroidetes/Chlorobi group	Bacteroidetes	Flavobacteriia	Flavobacteriales	Flavobacteriaceae
1	Bacteria	FCB group	Bacteroidetes/Chlorobi group	Bacteroidetes	Flavobacteriia	Flavobacteriales	Flavobacteriaceae
1	Bacteria	FCB group	Bacteroidetes/Chlorobi group	Bacteroidetes	Flavobacteriia	Flavobacteriales	Flavobacteriaceae
1	Bacteria	FCB group	Bacteroidetes/Chlorobi group	Bacteroidetes	Flavobacteriia	Flavobacteriales	Flavobacteriaceae
1	Bacteria	FCB group	Bacteroidetes/Chlorobi group	Bacteroidetes	Flavobacteriia	Flavobacteriales	Flavobacteriaceae
1	Bacteria	FCB group	Bacteroidetes/Chlorobi group	Bacteroidetes	Flavobacteriia	Flavobacteriales	Flavobacteriaceae
1	Bacteria	FCB group	Bacteroidetes/Chlorobi group	Bacteroidetes	Flavobacteriia	Flavobacteriales	Flavobacteriaceae
1	Bacteria	FCB group	Bacteroidetes/Chlorobi group	Bacteroidetes	Flavobacteriia	Flavobacteriales	Flavobacteriaceae
1	Bacteria	FCB group	Bacteroidetes/Chlorobi group	Bacteroidetes	Flavobacteriia	Flavobacteriales	Flavobacteriaceae
1	Bacteria	FCB group	Bacteroidetes/Chlorobi group	Bacteroidetes	Flavobacteriia	Flavobacteriales	Flavobacteriaceae
1	Bacteria	FCB group	Bacteroidetes/Chlorobi group	Bacteroidetes	Flavobacteriia	Flavobacteriales	Flavobacteriaceae
1	Bacteria	FCB group	Bacteroidetes/Chlorobi group	Bacteroidetes	Flavobacteriia	Flavobacteriales	Flavobacteriaceae
1	Bacteria	FCB group	Bacteroidetes/Chlorobi group	Bacteroidetes	Flavobacteriia	Flavobacteriales	Flavobacteriaceae
1	Bacteria	FCB group	Bacteroidetes/Chlorobi group	Bacteroidetes	Flavobacteriia	Flavobacteriales	Flavobacteriaceae
1	Bacteria	FCB group	Bacteroidetes/Chlorobi group	Bacteroidetes	Flavobacteriia	Flavobacteriales	Flavobacteriaceae
1	Bacteria	FCB group	Bacteroidetes/Chlorobi group	Bacteroidetes	Flavobacteriia	Flavobacteriales	Flavobacteriaceae
1	Bacteria	FCB group	Bacteroidetes/Chlorobi group	Bacteroidetes	Flavobacteriia	Flavobacteriales	Flavobacteriaceae
1	Bacteria	FCB group	Bacteroidetes/Chlorobi group	Bacteroidetes	Flavobacteriia	Flavobacteriales	Flavobacteriaceae
1	Bacteria	FCB group	Bacteroidetes/Chlorobi group	Bacteroidetes	Flavobacteriia	Flavobacteriales	Flavobacteriaceae
1	Bacteria	FCB group	Bacteroidetes/Chlorobi group	Bacteroidetes	Flavobacteriia	Flavobacteriales	Flavobacteriaceae
1	Bacteria	FCB group	Bacteroidetes/Chlorobi group	Bacteroidetes	Flavobacteriia	Flavobacteriales	Flavobacteriaceae
1	Bacteria	FCB group	Bacteroidetes/Chlorobi group	Bacteroidetes	Flavobacteriia	Flavobacteriales	Flavobacteriaceae
1	Bacteria	FCB group	Bacteroidetes/Chlorobi group	Bacteroidetes	Flavobacteriia	Flavobacteriales	Flavobacteriaceae
1	Bacteria	FCB group	Bacteroidetes/Chlorobi group	Bacteroidetes	Flavobacteriia	Flavobacteriales	Flavobacteriaceae
1	Bacteria	FCB group	Bacteroidetes/Chlorobi group	Bacteroidetes	Flavobacteriia	Flavobacteriales	Flavobacteriaceae
1	Bacteria	FCB group	Bacteroidetes/Chlorobi group	Bacteroidetes	Flavobacteriia	Flavobacteriales	Flavobacteriaceae
1	Bacteria	FCB group	Bacteroidetes/Chlorobi group	Bacteroidetes	Flavobacteriia	Flavobacteriales	Flavobacteriaceae
1	Bacteria	FCB group	Bacteroidetes/Chlorobi group	Bacteroidetes	Flavobacteriia	Flavobacteriales	Flavobacteriaceae
1	Bacteria	FCB group	Bacteroidetes/Chlorobi group	Bacteroidetes	Flavobacteriia	Flavobacteriales	Flavobacteriaceae
1	Bacteria	FCB group	Bacteroidetes/Chlorobi group	Bacteroidetes	Flavobacteriia	Flavobacteriales	Flavobacteriaceae
1	Bacteria	FCB group	Bacteroidetes/Chlorobi group	Bacteroidetes	Flavobacteriia	Flavobacteriales	Flavobacteriaceae
1	Bacteria	FCB group	Bacteroidetes/Chlorobi group	Bacteroidetes	Flavobacteriia	Flavobacteriales	Flavobacteriaceae
1	Bacteria	FCB group	Bacteroidetes/Chlorobi group	Bacteroidetes	Flavobacteriia	Flavobacteriales	Flavobacteriaceae
1	Bacteria	FCB group	Bacteroidetes/Chlorobi group	Bacteroidetes	Flavobacteriia	Flavobacteriales	Flavobacteriaceae
1	Bacteria	FCB group	Bacteroidetes/Chlorobi group	Bacteroidetes	Flavobacteriia	Flavobacteriales	Flavobacteriaceae
1	Bacteria	FCB group	Bacteroidetes/Chlorobi group	Bacteroidetes	Flavobacteriia	Flavobacteriales	Flavobacteriaceae
1	Bacteria	FCB group	Bacteroidetes/Chlorobi group	Bacteroidetes	Flavobacteriia	Flavobacteriales	Flavobacteriaceae
1	Bacteria	FCB group	Bacteroidetes/Chlorobi group	Bacteroidetes	Flavobacteriia	Flavobacteriales	Flavobacteriaceae
1	Bacteria	FCB group	Bacteroidetes/Chlorobi group	Bacteroidetes	Flavobacteriia	Flavobacteriales	Flavobacteriaceae
1	Bacteria	FCB group	Bacteroidetes/Chlorobi group	Bacteroidetes	Flavobacteriia	Flavobacteriales	Flavobacteriaceae
1	Bacteria	FCB group	Bacteroidetes/Chlorobi group	Bacteroidetes	Flavobacteriia	Flavobacteriales	Flavobacteriaceae
1	Bacteria	FCB group	Bacteroidetes/Chlorobi group	Bacteroidetes	Flavobacteriia	Flavobacteriales	Flavobacteriaceae
1	Bacteria	FCB group	Bacteroidetes/Chlorobi group	Bacteroidetes	Flavobacteriia	Flavobacteriales	Flavobacteriaceae
1	Bacteria	FCB group	Bacteroidetes/Chlorobi group	Bacteroidetes	Flavobacteriia	Flavobacteriales	Flavobacteriaceae
1	Bacteria	FCB group	Bacteroidetes/Chlorobi group	Bacteroidetes	Flavobacteriia	Flavobacteriales	Flavobacteriaceae
1	Bacteria	FCB group	Bacteroidetes/Chlorobi group	Bacteroidetes	Flavobacteriia	Flavobacteriales	Flavobacteriaceae
1	Bacteria	FCB group	Bacteroidetes/Chlorobi group	Bacteroidetes	Flavobacteriia	Flavobacteriales	Flavobacteriaceae
1	Bacteria	FCB group	Bacteroidetes/Chlorobi group	Bacteroidetes	Flavobacteriia	Flavobacteriales	Flavobacteriaceae
1	Bacteria	FCB group	Bacteroidetes/Chlorobi group	Bacteroidetes	Flavobacteriia	Flavobacteriales	Flavobacteriaceae
1	Bacteria	FCB group	Bacteroidetes/Chlorobi group	Bacteroidetes	Flavobacteriia	Flavobacteriales	Flavobacteriaceae
1	Bacteria	FCB group	Bacteroidetes/Chlorobi group	Bacteroidetes	Flavobacteriia	Flavobacteriales	Flavobacteriaceae
1	Bacteria	FCB group	Bacteroidetes/Chlorobi group	Bacteroidetes	Flavobacteriia	Flavobacteriales	Flavobacteriaceae
1	Bacteria	FCB group	Bacteroidetes/Chlorobi group	Bacteroidetes	Flavobacteriia	Flavobacteriales	Flavobacteriaceae
1	Bacteria	FCB group	Bacteroidetes/Chlorobi group	Bacteroidetes	Flavobacteriia	Flavobacteriales	Flavobacteriaceae
1	Bacteria	FCB group	Bacteroidetes/Chlorobi group	Bacteroidetes	Flavobacteriia	Flavobacteriales	Flavobacteriaceae
1	Bacteria	FCB group	Bacteroidetes/Chlorobi group	Bacteroidetes	Flavobacteriia	Flavobacteriales	Flavobacteriaceae
1	Bacteria	FCB group	Bacteroidetes/Chlorobi group	Bacteroidetes	Flavobacteriia	Flavobacteriales	Flavobacteriaceae
1	Bacteria	FCB group	Bacteroidetes/Chlorobi group	Bacteroidetes	Flavobacteriia	Flavobacteriales	Flavobacteriaceae
1	Bacteria	FCB group	Bacteroidetes/Chlorobi group	Bacteroidetes	Flavobacteriia	Flavobacteriales	Flavobacteriaceae
1	Bacteria	FCB group	Bacteroidetes/Chlorobi group	Bacteroidetes	Flavobacteriia	Flavobacteriales	Flavobacteriaceae
1	Bacteria	FCB group	Bacteroidetes/Chlorobi group	Bacteroidetes	Flavobacteriia	Flavobacteriales	Flavobacteriaceae
1	Bacteria	FCB group	Bacteroidetes/Chlorobi group	Bacteroidetes	Flavobacteriia	Flavobacteriales	Flavobacteriaceae
1	Bacteria	FCB group	Bacteroidetes/Chlorobi group	Bacteroidetes	Flavobacteriia	Flavobacteriales	Flavobacteriaceae
1	Bacteria	FCB group	Bacteroidetes/Chlorobi group	Bacteroidetes	Flavobacteriia	Flavobacteriales	Flavobacteriaceae
1	Bacteria	FCB group	Bacteroidetes/Chlorobi group	Bacteroidetes	Flavobacteriia	Flavobacteriales	Flavobacteriaceae
1	Bacteria	FCB group	Bacteroidetes/Chlorobi group	Bacteroidetes	Flavobacteriia	Flavobacteriales	Flavobacteriaceae
1	Bacteria	FCB group	Bacteroidetes/Chlorobi group	Bacteroidetes	Flavobacteriia	Flavobacteriales	Flavobacteriaceae
1	Bacteria	FCB group	Bacteroidetes/Chlorobi group	Bacteroidetes	Flavobacteriia	Flavobacteriales	Flavobacteriaceae
1	Bacteria	FCB group	Bacteroidetes/Chlorobi group	Bacteroidetes	Flavobacteriia	Flavobacteriales	Flavobacteriaceae
1	Bacteria	FCB group	Bacteroidetes/Chlorobi group	Bacteroidetes	Flavobacteriia	Flavobacteriales	Flavobacteriaceae
1	Bacteria	FCB group	Bacteroidetes/Chlorobi group	Bacteroidetes	Flavobacteriia	Flavobacteriales	Flavobacteriaceae
1	Bacteria	FCB group	Bacteroidetes/Chlorobi group	Bacteroidetes	Flavobacteriia	Flavobacteriales	Flavobacteriaceae
1	Bacteria	FCB group	Bacteroidetes/Chlorobi group	Bacteroidetes	Flavobacteriia	Flavobacteriales	Flavobacteriaceae
1	Bacteria	FCB group	Bacteroidetes/Chlorobi group	Bacteroidetes	Flavobacteriia	Flavobacteriales	Flavobacteriaceae
1	Bacteria	FCB group	Bacteroidetes/Chlorobi group	Bacteroidetes	Flavobacteriia	Flavobacteriales	Flavobacteriaceae
1	Bacteria	FCB group	Bacteroidetes/Chlorobi group	Bacteroidetes	Flavobacteriia	Flavobacteriales	Flavobacteriaceae
1	Bacteria	FCB group	Bacteroidetes/Chlorobi group	Bacteroidetes	Flavobacteriia	Flavobacteriales	Flavobacteriaceae
1	Bacteria	FCB group	Bacteroidetes/Chlorobi group	Bacteroidetes	Flavobacteriia	Flavobacteriales	Flavobacteriaceae
1	Bacteria	FCB group	Bacteroidetes/Chlorobi group	Bacteroidetes	Flavobacteriia	Flavobacteriales	Flavobacteriaceae
1	Bacteria	FCB group	Bacteroidetes/Chlorobi group	Bacteroidetes	Flavobacteriia	Flavobacteriales	Flavobacteriaceae
1	Bacteria	FCB group	Bacteroidetes/Chlorobi group	Bacteroidetes	Flavobacteriia	Flavobacteriales	Flavobacteriaceae
1	Bacteria	FCB group	Bacteroidetes/Chlorobi group	Bacteroidetes	Flavobacteriia	Flavobacteriales	Flavobacteriaceae
1	Bacteria	FCB group	Bacteroidetes/Chlorobi group	Bacteroidetes	Flavobacteriia	Flavobacteriales	Flavobacteriaceae
1	Bacteria	FCB group	Bacteroidetes/Chlorobi group	Bacteroidetes	Flavobacteriia	Flavobacteriales	Flavobacteriaceae
1	Bacteria	FCB group	Bacteroidetes/Chlorobi group	Bacteroidetes	Flavobacteriia	Flavobacteriales	Flavobacteriaceae
1	Bacteria	FCB group	Bacteroidetes/Chlorobi group	Bacteroidetes	Flavobacteriia	Flavobacteriales	Flavobacteriaceae
1	Bacteria	FCB group	Bacteroidetes/Chlorobi group	Bacteroidetes	Flavobacteriia	Flavobacteriales	Cryomorphaceae
1	Bacteria	FCB group	Bacteroidetes/Chlorobi group	Bacteroidetes	Flavobacteriia	Flavobacteriales	Cryomorphaceae
1	Bacteria	FCB group	Bacteroidetes/Chlorobi group	Bacteroidetes	Flavobacteriia	Flavobacteriales	Cryomorphaceae
1	Bacteria	FCB group	Bacteroidetes/Chlorobi group	Bacteroidetes	Flavobacteriia	Flavobacteriales	Cryomorphaceae
1	Bacteria	FCB group	Bacteroidetes/Chlorobi group	Bacteroidetes	Flavobacteriia	Flavobacteriales	Crocinitomicaceae
1	Bacteria	FCB group	Bacteroidetes/Chlorobi group	Bacteroidetes	Flavobacteriia	Flavobacteriales	Blattabacteriaceae
1	Bacteria	FCB group	Bacteroidetes/Chlorobi group	Bacteroidetes	Flavobacteriia	Flavobacteriales	Blattabacteriaceae
1	Bacteria	FCB group	Bacteroidetes/Chlorobi group	Bacteroidetes	Flavobacteriia	Flavobacteriales	Blattabacteriaceae
1	Bacteria	FCB group	Bacteroidetes/Chlorobi group	Bacteroidetes	Cytophagia	Cytophagales	Persicobacteraceae
1	Bacteria	FCB group	Bacteroidetes/Chlorobi group	Bacteroidetes	Cytophagia	Cytophagales	Hymenobacteraceae
1	Bacteria	FCB group	Bacteroidetes/Chlorobi group	Bacteroidetes	Cytophagia	Cytophagales	Hymenobacteraceae
1	Bacteria	FCB group	Bacteroidetes/Chlorobi group	Bacteroidetes	Cytophagia	Cytophagales	Hymenobacteraceae
1	Bacteria	FCB group	Bacteroidetes/Chlorobi group	Bacteroidetes	Cytophagia	Cytophagales	Hymenobacteraceae
1	Bacteria	FCB group	Bacteroidetes/Chlorobi group	Bacteroidetes	Cytophagia	Cytophagales	Hymenobacteraceae
1	Bacteria	FCB group	Bacteroidetes/Chlorobi group	Bacteroidetes	Cytophagia	Cytophagales	Hymenobacteraceae
1	Bacteria	FCB group	Bacteroidetes/Chlorobi group	Bacteroidetes	Cytophagia	Cytophagales	Hymenobacteraceae
1	Bacteria	FCB group	Bacteroidetes/Chlorobi group	Bacteroidetes	Cytophagia	Cytophagales	Flammeovirgaceae
1	Bacteria	FCB group	Bacteroidetes/Chlorobi group	Bacteroidetes	Cytophagia	Cytophagales	Flammeovirgaceae
1	Bacteria	FCB group	Bacteroidetes/Chlorobi group	Bacteroidetes	Cytophagia	Cytophagales	Flammeovirgaceae
1	Bacteria	FCB group	Bacteroidetes/Chlorobi group	Bacteroidetes	Cytophagia	Cytophagales	Flammeovirgaceae
1	Bacteria	FCB group	Bacteroidetes/Chlorobi group	Bacteroidetes	Cytophagia	Cytophagales	Flammeovirgaceae
1	Bacteria	FCB group	Bacteroidetes/Chlorobi group	Bacteroidetes	Cytophagia	Cytophagales	Cytophagaceae
1	Bacteria	FCB group	Bacteroidetes/Chlorobi group	Bacteroidetes	Cytophagia	Cytophagales	Cytophagaceae
1	Bacteria	FCB group	Bacteroidetes/Chlorobi group	Bacteroidetes	Cytophagia	Cytophagales	Cytophagaceae
1	Bacteria	FCB group	Bacteroidetes/Chlorobi group	Bacteroidetes	Cytophagia	Cytophagales	Cytophagaceae
1	Bacteria	FCB group	Bacteroidetes/Chlorobi group	Bacteroidetes	Cytophagia	Cytophagales	Cytophagaceae
1	Bacteria	FCB group	Bacteroidetes/Chlorobi group	Bacteroidetes	Cytophagia	Cytophagales	Cytophagaceae
1	Bacteria	FCB group	Bacteroidetes/Chlorobi group	Bacteroidetes	Cytophagia	Cytophagales	Cyclobacteriaceae
1	Bacteria	FCB group	Bacteroidetes/Chlorobi group	Bacteroidetes	Cytophagia	Cytophagales	Cyclobacteriaceae
1	Bacteria	FCB group	Bacteroidetes/Chlorobi group	Bacteroidetes	Cytophagia	Cytophagales	Cyclobacteriaceae
1	Bacteria	FCB group	Bacteroidetes/Chlorobi group	Bacteroidetes	Cytophagia	Cytophagales	Cyclobacteriaceae
1	Bacteria	FCB group	Bacteroidetes/Chlorobi group	Bacteroidetes	Cytophagia	Cytophagales	Amoebophilaceae
1	Bacteria	FCB group	Bacteroidetes/Chlorobi group	Bacteroidetes	Cytophagia	Cytophagales	Amoebophilaceae
1	Bacteria	FCB group	Bacteroidetes/Chlorobi group	Bacteroidetes	Chitinophagia	Chitinophagales	Chitinophagaceae
1	Bacteria	FCB group	Bacteroidetes/Chlorobi group	Bacteroidetes	Chitinophagia	Chitinophagales	Chitinophagaceae
1	Bacteria	FCB group	Bacteroidetes/Chlorobi group	Bacteroidetes	Chitinophagia	Chitinophagales	Chitinophagaceae
1	Bacteria	FCB group	Bacteroidetes/Chlorobi group	Bacteroidetes	Chitinophagia	Chitinophagales	Chitinophagaceae
1	Bacteria	FCB group	Bacteroidetes/Chlorobi group	Bacteroidetes	Chitinophagia	Chitinophagales	Chitinophagaceae
1	Bacteria	FCB group	Bacteroidetes/Chlorobi group	Bacteroidetes	Chitinophagia	Chitinophagales	Chitinophagaceae
1	Bacteria	FCB group	Bacteroidetes/Chlorobi group	Bacteroidetes	Chitinophagia	Chitinophagales	Chitinophagaceae
1	Bacteria	FCB group	Bacteroidetes/Chlorobi group	Bacteroidetes	Bacteroidia	Marinilabiliales	Prolixibacteraceae
1	Bacteria	FCB group	Bacteroidetes/Chlorobi group	Bacteroidetes	Bacteroidia	Marinilabiliales	Marinilabiliaceae
1	Bacteria	FCB group	Bacteroidetes/Chlorobi group	Bacteroidetes	Bacteroidia	Bacteroidales	unclassified Bacteroidales
1	Bacteria	FCB group	Bacteroidetes/Chlorobi group	Bacteroidetes	Bacteroidia	Bacteroidales	unclassified Bacteroidales
1	Bacteria	FCB group	Bacteroidetes/Chlorobi group	Bacteroidetes	Bacteroidia	Bacteroidales	Rikenellaceae
1	Bacteria	FCB group	Bacteroidetes/Chlorobi group	Bacteroidetes	Bacteroidia	Bacteroidales	Rikenellaceae
1	Bacteria	FCB group	Bacteroidetes/Chlorobi group	Bacteroidetes	Bacteroidia	Bacteroidales	Rikenellaceae
1	Bacteria	FCB group	Bacteroidetes/Chlorobi group	Bacteroidetes	Bacteroidia	Bacteroidales	Prevotellaceae
1	Bacteria	FCB group	Bacteroidetes/Chlorobi group	Bacteroidetes	Bacteroidia	Bacteroidales	Prevotellaceae
1	Bacteria	FCB group	Bacteroidetes/Chlorobi group	Bacteroidetes	Bacteroidia	Bacteroidales	Prevotellaceae
1	Bacteria	FCB group	Bacteroidetes/Chlorobi group	Bacteroidetes	Bacteroidia	Bacteroidales	Prevotellaceae
1	Bacteria	FCB group	Bacteroidetes/Chlorobi group	Bacteroidetes	Bacteroidia	Bacteroidales	Prevotellaceae
1	Bacteria	FCB group	Bacteroidetes/Chlorobi group	Bacteroidetes	Bacteroidia	Bacteroidales	Prevotellaceae
1	Bacteria	FCB group	Bacteroidetes/Chlorobi group	Bacteroidetes	Bacteroidia	Bacteroidales	Prevotellaceae
1	Bacteria	FCB group	Bacteroidetes/Chlorobi group	Bacteroidetes	Bacteroidia	Bacteroidales	Prevotellaceae
1	Bacteria	FCB group	Bacteroidetes/Chlorobi group	Bacteroidetes	Bacteroidia	Bacteroidales	Prevotellaceae
1	Bacteria	FCB group	Bacteroidetes/Chlorobi group	Bacteroidetes	Bacteroidia	Bacteroidales	Prevotellaceae
1	Bacteria	FCB group	Bacteroidetes/Chlorobi group	Bacteroidetes	Bacteroidia	Bacteroidales	Prevotellaceae
1	Bacteria	FCB group	Bacteroidetes/Chlorobi group	Bacteroidetes	Bacteroidia	Bacteroidales	Prevotellaceae
1	Bacteria	FCB group	Bacteroidetes/Chlorobi group	Bacteroidetes	Bacteroidia	Bacteroidales	Prevotellaceae
1	Bacteria	FCB group	Bacteroidetes/Chlorobi group	Bacteroidetes	Bacteroidia	Bacteroidales	Prevotellaceae
1	Bacteria	FCB group	Bacteroidetes/Chlorobi group	Bacteroidetes	Bacteroidia	Bacteroidales	Prevotellaceae
1	Bacteria	FCB group	Bacteroidetes/Chlorobi group	Bacteroidetes	Bacteroidia	Bacteroidales	Prevotellaceae
1	Bacteria	FCB group	Bacteroidetes/Chlorobi group	Bacteroidetes	Bacteroidia	Bacteroidales	Prevotellaceae
1	Bacteria	FCB group	Bacteroidetes/Chlorobi group	Bacteroidetes	Bacteroidia	Bacteroidales	Prevotellaceae
1	Bacteria	FCB group	Bacteroidetes/Chlorobi group	Bacteroidetes	Bacteroidia	Bacteroidales	Prevotellaceae
1	Bacteria	FCB group	Bacteroidetes/Chlorobi group	Bacteroidetes	Bacteroidia	Bacteroidales	Prevotellaceae
1	Bacteria	FCB group	Bacteroidetes/Chlorobi group	Bacteroidetes	Bacteroidia	Bacteroidales	Prevotellaceae
1	Bacteria	FCB group	Bacteroidetes/Chlorobi group	Bacteroidetes	Bacteroidia	Bacteroidales	Prevotellaceae
1	Bacteria	FCB group	Bacteroidetes/Chlorobi group	Bacteroidetes	Bacteroidia	Bacteroidales	Prevotellaceae
1	Bacteria	FCB group	Bacteroidetes/Chlorobi group	Bacteroidetes	Bacteroidia	Bacteroidales	Prevotellaceae
1	Bacteria	FCB group	Bacteroidetes/Chlorobi group	Bacteroidetes	Bacteroidia	Bacteroidales	Prevotellaceae
1	Bacteria	FCB group	Bacteroidetes/Chlorobi group	Bacteroidetes	Bacteroidia	Bacteroidales	Prevotellaceae
1	Bacteria	FCB group	Bacteroidetes/Chlorobi group	Bacteroidetes	Bacteroidia	Bacteroidales	Prevotellaceae
1	Bacteria	FCB group	Bacteroidetes/Chlorobi group	Bacteroidetes	Bacteroidia	Bacteroidales	Prevotellaceae
1	Bacteria	FCB group	Bacteroidetes/Chlorobi group	Bacteroidetes	Bacteroidia	Bacteroidales	Prevotellaceae
1	Bacteria	FCB group	Bacteroidetes/Chlorobi group	Bacteroidetes	Bacteroidia	Bacteroidales	Prevotellaceae
1	Bacteria	FCB group	Bacteroidetes/Chlorobi group	Bacteroidetes	Bacteroidia	Bacteroidales	Prevotellaceae
1	Bacteria	FCB group	Bacteroidetes/Chlorobi group	Bacteroidetes	Bacteroidia	Bacteroidales	Prevotellaceae
1	Bacteria	FCB group	Bacteroidetes/Chlorobi group	Bacteroidetes	Bacteroidia	Bacteroidales	Porphyromonadaceae
1	Bacteria	FCB group	Bacteroidetes/Chlorobi group	Bacteroidetes	Bacteroidia	Bacteroidales	Porphyromonadaceae
1	Bacteria	FCB group	Bacteroidetes/Chlorobi group	Bacteroidetes	Bacteroidia	Bacteroidales	Porphyromonadaceae
1	Bacteria	FCB group	Bacteroidetes/Chlorobi group	Bacteroidetes	Bacteroidia	Bacteroidales	Porphyromonadaceae
1	Bacteria	FCB group	Bacteroidetes/Chlorobi group	Bacteroidetes	Bacteroidia	Bacteroidales	Porphyromonadaceae
1	Bacteria	FCB group	Bacteroidetes/Chlorobi group	Bacteroidetes	Bacteroidia	Bacteroidales	Porphyromonadaceae
1	Bacteria	FCB group	Bacteroidetes/Chlorobi group	Bacteroidetes	Bacteroidia	Bacteroidales	Porphyromonadaceae
1	Bacteria	FCB group	Bacteroidetes/Chlorobi group	Bacteroidetes	Bacteroidia	Bacteroidales	Porphyromonadaceae
1	Bacteria	FCB group	Bacteroidetes/Chlorobi group	Bacteroidetes	Bacteroidia	Bacteroidales	Porphyromonadaceae
1	Bacteria	FCB group	Bacteroidetes/Chlorobi group	Bacteroidetes	Bacteroidia	Bacteroidales	Porphyromonadaceae
1	Bacteria	FCB group	Bacteroidetes/Chlorobi group	Bacteroidetes	Bacteroidia	Bacteroidales	Porphyromonadaceae
1	Bacteria	FCB group	Bacteroidetes/Chlorobi group	Bacteroidetes	Bacteroidia	Bacteroidales	Porphyromonadaceae
1	Bacteria	FCB group	Bacteroidetes/Chlorobi group	Bacteroidetes	Bacteroidia	Bacteroidales	Porphyromonadaceae
1	Bacteria	FCB group	Bacteroidetes/Chlorobi group	Bacteroidetes	Bacteroidia	Bacteroidales	Porphyromonadaceae
1	Bacteria	FCB group	Bacteroidetes/Chlorobi group	Bacteroidetes	Bacteroidia	Bacteroidales	Porphyromonadaceae
1	Bacteria	FCB group	Bacteroidetes/Chlorobi group	Bacteroidetes	Bacteroidia	Bacteroidales	Odoribacteraceae
1	Bacteria	FCB group	Bacteroidetes/Chlorobi group	Bacteroidetes	Bacteroidia	Bacteroidales	Bacteroidaceae
1	Bacteria	FCB group	Bacteroidetes/Chlorobi group	Bacteroidetes	Bacteroidia	Bacteroidales	Bacteroidaceae
1	Bacteria	FCB group	Bacteroidetes/Chlorobi group	Bacteroidetes	Bacteroidia	Bacteroidales	Bacteroidaceae
1	Bacteria	FCB group	Bacteroidetes/Chlorobi group	Bacteroidetes	Bacteroidia	Bacteroidales	Bacteroidaceae
1	Bacteria	FCB group	Bacteroidetes/Chlorobi group	Bacteroidetes	Bacteroidia	Bacteroidales	Bacteroidaceae
1	Bacteria	FCB group	Bacteroidetes/Chlorobi group	Bacteroidetes	Bacteroidia	Bacteroidales	Bacteroidaceae
1	Bacteria	FCB group	Bacteroidetes/Chlorobi group	Bacteroidetes	Bacteroidia	Bacteroidales	Bacteroidaceae
1	Bacteria	FCB group	Bacteroidetes/Chlorobi group	Bacteroidetes	Bacteroidia	Bacteroidales	Bacteroidaceae
1	Bacteria	FCB group	Bacteroidetes/Chlorobi group	Bacteroidetes	Bacteroidia	Bacteroidales	Bacteroidaceae
1	Bacteria	FCB group	Bacteroidetes/Chlorobi group	Bacteroidetes	Bacteroidia	Bacteroidales	Bacteroidaceae
1	Bacteria	FCB group	Bacteroidetes/Chlorobi group	Bacteroidetes	Bacteroidia	Bacteroidales	Bacteroidaceae
1	Bacteria	FCB group	Bacteroidetes/Chlorobi group	Bacteroidetes	Bacteroidia	Bacteroidales	Bacteroidaceae
1	Bacteria	FCB group	Bacteroidetes/Chlorobi group	Bacteroidetes	Bacteroidia	Bacteroidales	Bacteroidaceae
1	Bacteria	FCB group	Bacteroidetes/Chlorobi group	Bacteroidetes	Bacteroidia	Bacteroidales	Bacteroidaceae
1	Bacteria	FCB group	Bacteroidetes/Chlorobi group	Bacteroidetes	Bacteroidia	Bacteroidales	Bacteroidaceae
1	Bacteria	FCB group	Bacteroidetes/Chlorobi group	Bacteroidetes	Bacteroidia	Bacteroidales	Bacteroidaceae
1	Bacteria	FCB group	Bacteroidetes/Chlorobi group	Bacteroidetes	Bacteroidia	Bacteroidales	Bacteroidaceae
1	Bacteria	FCB group	Bacteroidetes/Chlorobi group	Bacteroidetes	Bacteroidia	Bacteroidales	Bacteroidaceae
1	Bacteria	FCB group	Bacteroidetes/Chlorobi group	Bacteroidetes	Bacteroidia	Bacteroidales	Bacteroidaceae
1	Bacteria	FCB group	Bacteroidetes/Chlorobi group	Bacteroidetes	Bacteroidia	Bacteroidales	Bacteroidaceae
1	Bacteria	FCB group	Bacteroidetes/Chlorobi group	Bacteroidetes	Bacteroidia	Bacteroidales	Bacteroidaceae
1	Bacteria	FCB group	Bacteroidetes/Chlorobi group	Bacteroidetes	Bacteroidia	Bacteroidales	Bacteroidaceae
1	Bacteria	FCB group	Bacteroidetes/Chlorobi group	Bacteroidetes	Bacteroidia	Bacteroidales	Bacteroidaceae
1	Bacteria	FCB group	Bacteroidetes/Chlorobi group	Bacteroidetes	Bacteroidia	Bacteroidales	Bacteroidaceae
1	Bacteria	FCB group	Bacteroidetes/Chlorobi group	Bacteroidetes	Bacteroidia	Bacteroidales	Bacteroidaceae
1	Bacteria	Elusimicrobia	unclassified Elusimicrobia	Elusimicrobia bacterium RIFOXYD2_FULL_34_30			
1	Bacteria	Elusimicrobia	unclassified Elusimicrobia	Elusimicrobia bacterium RIFOXYA2_FULL_58_8			
1	Bacteria	Elusimicrobia	unclassified Elusimicrobia	Elusimicrobia bacterium RIFOXYA12_FULL_57_11			
1	Bacteria	Elusimicrobia	unclassified Elusimicrobia	Elusimicrobia bacterium RIFCSPLOWO2_01_FULL_64_13			
1	Bacteria	Elusimicrobia	unclassified Elusimicrobia	Elusimicrobia bacterium RIFCSPLOWO2_01_FULL_60_11			
1	Bacteria	Elusimicrobia	unclassified Elusimicrobia	Elusimicrobia bacterium GWC2_61_19			
1	Bacteria	Elusimicrobia	Endomicrobia	Endomicrobiales	Endomicrobiaceae	Endomicrobium	Candidatus Endomicrobium trichonymphae
1	Bacteria	Dictyoglomi	Dictyoglomia	Dictyoglomales	Dictyoglomaceae	Dictyoglomus	environmental samples
1	Bacteria	Dictyoglomi	Dictyoglomia	Dictyoglomales	Dictyoglomaceae	Dictyoglomus	Dictyoglomus turgidum
1	Bacteria	Deferribacteres	Deferribacteres	Deferribacterales	Deferribacteraceae	Mucispirillum	Mucispirillum schaedleri
1	Bacteria	Calditrichaeota	Calditrichae	Calditrichales	Calditrichaceae	Caldithrix	Caldithrix abyssi
1	Bacteria	Caldiserica	Caldisericia	Caldisericales	Caldisericaceae	Caldisericum	Caldisericum sp. CG2_30_36_11
1	Bacteria	Aquificae	Aquificae	Aquificales	Hydrogenothermaceae	Sulfurihydrogenibium	Sulfurihydrogenibium yellowstonense
1	Bacteria	Aquificae	Aquificae	Aquificales	Hydrogenothermaceae	Persephonella	Persephonella sp. KM09-Lau-8
1	Bacteria	Aquificae	Aquificae	Aquificales	Aquificaceae	Thermocrinis	Thermocrinis sp. GBS
1	Bacteria	Aquificae	Aquificae	Aquificales	Aquificaceae	Thermocrinis	Thermocrinis minervae
1	Bacteria	Aquificae	Aquificae	Aquificales	Aquificaceae	Thermocrinis	Thermocrinis albus
1	Bacteria	Aquificae	Aquificae	Aquificales	Aquificaceae	Hydrogenobacter	Hydrogenobacter thermophilus
1	Bacteria	Acidobacteria	unclassified Acidobacteria	unclassified Acidobacteria (miscellaneous)	Acidobacteria bacterium SCGC AG-212-P17		
1	Bacteria	Acidobacteria	unclassified Acidobacteria	unclassified Acidobacteria (miscellaneous)	Acidobacteria bacterium RIFCSPHIGHO2_12_FULL_67_30		
1	Bacteria	Acidobacteria	unclassified Acidobacteria	unclassified Acidobacteria (miscellaneous)	Acidobacteria bacterium RBG_16_64_8		
1	Bacteria	Acidobacteria	unclassified Acidobacteria	unclassified Acidobacteria (miscellaneous)	Acidobacteria bacterium 13_1_40CM_4_61_5		
1	Bacteria	Acidobacteria	unclassified Acidobacteria	unclassified Acidobacteria (miscellaneous)	Acidobacteria bacterium 13_1_40CM_2_56_11		
1	Bacteria	Acidobacteria	unclassified Acidobacteria	unclassified Acidobacteria (miscellaneous)	Acidobacteria bacterium 13_1_20CM_3_58_11		
1	Bacteria	Acidobacteria	environmental samples	uncultured Acidobacteria bacterium HF4000_26D02			
1	Bacteria	Acidobacteria	Solibacteres	Solibacterales	Solibacteraceae	Candidatus Solibacter	Candidatus Solibacter usitatus
1	Bacteria	Acidobacteria	Solibacteres	Solibacterales			
1	Bacteria	Acidobacteria	Holophagae	Holophagales	Holophagaceae		
1	Bacteria	Acidobacteria	Acidobacteriia	Acidobacteriales	unclassified Acidobacteriales	Acidobacteriales bacterium 13_1_40CM_3_55_5	
1	Bacteria	Acidobacteria	Acidobacteriia	Acidobacteriales	environmental samples	uncultured Acidobacteriales bacterium HF0200_23L05	
1	Bacteria	Acidobacteria	Acidobacteriia	Acidobacteriales	Acidobacteriaceae	unclassified Acidobacteriaceae	
1	Bacteria	Acidobacteria	Acidobacteriia	Acidobacteriales	Acidobacteriaceae	Terriglobus	Terriglobus sp. TAA 43
1	Bacteria	Acidobacteria	Acidobacteriia	Acidobacteriales	Acidobacteriaceae	Terriglobus	Terriglobus sp. 03SUJ4
1	Bacteria	Acidobacteria	Acidobacteriia	Acidobacteriales	Acidobacteriaceae	Acidobacterium	
1	Archaea	unclassified Archaea	unclassified Archaea (miscellaneous)	archaeon RBG_16_50_20			
1	Archaea	unclassified Archaea	unclassified Archaea (miscellaneous)	archaeon GW2011_AR10			
1	Archaea	unclassified Archaea	unclassified Archaea (miscellaneous)				
1	Archaea	environmental samples	uncultured archaeon MedDCM-OCT-S02-C115				
1	Archaea	environmental samples	uncultured archaeon GZfos26G2				
1	Archaea	TACK group	Thaumarchaeota	unclassified Thaumarchaeota	Marine Group I		
1	Archaea	TACK group	Thaumarchaeota	unclassified Thaumarchaeota	Candidatus Nitrosotenuis	Candidatus Nitrosotenuis cloacae	
1	Archaea	TACK group	Thaumarchaeota	unclassified Thaumarchaeota	Candidatus Caldiarchaeum	Candidatus Caldiarchaeum subterraneum	
1	Archaea	TACK group	Thaumarchaeota	environmental samples	uncultured marine thaumarchaeote KM3_72_F07		
1	Archaea	TACK group	Thaumarchaeota	Nitrososphaeria	Nitrososphaerales	Nitrososphaeraceae	Nitrososphaera
1	Archaea	TACK group	Thaumarchaeota	Nitrososphaeria	Nitrososphaerales	Nitrososphaeraceae	Nitrososphaera
1	Archaea	TACK group	Thaumarchaeota	Nitrososphaeria	Nitrososphaerales	Nitrososphaeraceae	Nitrososphaera
1	Archaea	TACK group	Thaumarchaeota	Nitrosopumilales	Nitrosopumilaceae	Nitrosopumilus	Candidatus Nitrosopumilus salaria
1	Archaea	TACK group	Thaumarchaeota	Nitrosopumilales	Nitrosopumilaceae	Nitrosopumilus	Candidatus Nitrosopumilus piranensis
1	Archaea	TACK group	Thaumarchaeota	Nitrosopumilales	Nitrosopumilaceae	Candidatus Nitrosoarchaeum	Candidatus Nitrosoarchaeum limnia
1	Archaea	TACK group	Thaumarchaeota				
1	Archaea	TACK group	Crenarchaeota	Thermoprotei	Thermoproteales	Thermoproteaceae	Vulcanisaeta
1	Archaea	TACK group	Crenarchaeota	Thermoprotei	Thermoproteales	Thermoproteaceae	Thermoproteus
1	Archaea	TACK group	Crenarchaeota	Thermoprotei	Thermoproteales	Thermoproteaceae	Thermoproteus
1	Archaea	TACK group	Crenarchaeota	Thermoprotei	Thermoproteales	Thermoproteaceae	Caldivirga
1	Archaea	TACK group	Crenarchaeota	Thermoprotei	Thermoproteales	Thermofilaceae	Thermofilum
1	Archaea	TACK group	Crenarchaeota	Thermoprotei	Thermoproteales	Thermofilaceae	Thermofilum
1	Archaea	TACK group	Crenarchaeota	Thermoprotei	Sulfolobales	Sulfolobaceae	Sulfolobus
1	Archaea	TACK group	Crenarchaeota	Thermoprotei	Sulfolobales	Sulfolobaceae	Acidianus
1	Archaea	TACK group	Crenarchaeota	Thermoprotei	Desulfurococcales	Desulfurococcaceae	Ignicoccus
1	Archaea	TACK group	Crenarchaeota	Thermoprotei	Desulfurococcales	Desulfurococcaceae	Aeropyrum
1	Archaea	TACK group	Candidatus Korarchaeota	Candidatus Korarchaeum	Candidatus Korarchaeum cryptofilum		
1	Archaea	TACK group	Candidatus Bathyarchaeota	unclassified Candidatus Bathyarchaeota	miscellaneous Crenarchaeota group archaeon SMTZ1-55		
1	Archaea	TACK group	Candidatus Bathyarchaeota	unclassified Candidatus Bathyarchaeota	miscellaneous Crenarchaeota group archaeon SMTZ-80		
1	Archaea	TACK group	Candidatus Bathyarchaeota	unclassified Candidatus Bathyarchaeota	Candidatus Bathyarchaeota archaeon RBG_13_52_12		
1	Archaea	TACK group	Candidatus Bathyarchaeota	unclassified Candidatus Bathyarchaeota	Candidatus Bathyarchaeota archaeon B25		
1	Archaea	TACK group	Candidatus Bathyarchaeota	unclassified Candidatus Bathyarchaeota			
1	Archaea	Euryarchaeota	unclassified Euryarchaeota	unclassified Euryarchaeota (miscellaneous)	Euryarchaeota archaeon SM23-78		
1	Archaea	Euryarchaeota	unclassified Euryarchaeota	unclassified Euryarchaeota (miscellaneous)	Euryarchaeota archaeon RBG_19FT_COMBO_56_21		
1	Archaea	Euryarchaeota	unclassified Euryarchaeota	unclassified Euryarchaeota (miscellaneous)	Euryarchaeota archaeon RBG_16_68_12		
1	Archaea	Euryarchaeota	unclassified Euryarchaeota	unclassified Euryarchaeota (miscellaneous)	Euryarchaeota archaeon RBG_16_62_10		
1	Archaea	Euryarchaeota	unclassified Euryarchaeota	unclassified Euryarchaeota (miscellaneous)	Euryarchaeota archaeon RBG_13_31_8		
1	Archaea	Euryarchaeota	unclassified Euryarchaeota	unclassified Euryarchaeota (miscellaneous)			
1	Archaea	Euryarchaeota	unclassified Euryarchaeota	candidate division MSBL1	candidate divison MSBL1 archaeon SCGC-AAA259E19		
1	Archaea	Euryarchaeota	unclassified Euryarchaeota	Marine Group III	Marine Group III euryarchaeote CG-Epi2		
1	Archaea	Euryarchaeota	unclassified Euryarchaeota	Marine Group II	environmental samples	uncultured marine group II euryarchaeote	
1	Archaea	Euryarchaeota	unclassified Euryarchaeota	DHVE2 group	Aciduliprofundum	Aciduliprofundum boonei	
1	Archaea	Euryarchaeota	unclassified Euryarchaeota	Candidatus Altiarchaeales	unclassified Candidatus Altiarchaeales	Candidatus Altiarchaeales archaeon WOR_SM1_79	
1	Archaea	Euryarchaeota	unclassified Euryarchaeota	Candidatus Altiarchaeales	Candidatus Altiarchaeum	Candidatus Altiarchaeum sp. CG2_30_32_3053	
1	Archaea	Euryarchaeota	environmental samples	uncultured marine group II/III euryarchaeote KM3_77_D02			
1	Archaea	Euryarchaeota	environmental samples	uncultured marine group II/III euryarchaeote KM3_57_F04			
1	Archaea	Euryarchaeota	environmental samples	uncultured marine group II/III euryarchaeote AD1000_29_E08			
1	Archaea	Euryarchaeota	environmental samples				
1	Archaea	Euryarchaeota	Thermoplasmata	unclassified Thermoplasmata	methanogenic archaeon ISO4-H5		
1	Archaea	Euryarchaeota	Thermoplasmata	Thermoplasmatales	unclassified Thermoplasmatales	unclassified Thermoplasmatales (miscellaneous)	Thermoplasmatales archaeon SM1-50
1	Archaea	Euryarchaeota	Thermoplasmata	Thermoplasmatales	unclassified Thermoplasmatales	unclassified Thermoplasmatales (miscellaneous)	Thermoplasmatales archaeon SG8-52-3
1	Archaea	Euryarchaeota	Thermoplasmata	Thermoplasmatales	unclassified Thermoplasmatales	unclassified Thermoplasmatales (miscellaneous)	Thermoplasmatales archaeon SCGC AB-540-F20
1	Archaea	Euryarchaeota	Thermoplasmata	Thermoplasmatales	unclassified Thermoplasmatales	unclassified Thermoplasmatales (miscellaneous)	Thermoplasmatales archaeon BRNA1
1	Archaea	Euryarchaeota	Thermoplasmata	Methanomassiliicoccales	unclassified Methanomassiliicoccales	Methanomassiliicoccales archaeon RumEn M1	
1	Archaea	Euryarchaeota	Thermoplasmata	Methanomassiliicoccales	Methanomassiliicoccaceae	Methanomassiliicoccus	Candidatus Methanomassiliicoccus intestinalis
1	Archaea	Euryarchaeota	Thermoplasmata	Methanomassiliicoccales	Methanomassiliicoccaceae	Candidatus Methanomethylophilus	Candidatus Methanomethylophilus alvus
1	Archaea	Euryarchaeota	Thermococci	Thermococcales	Thermococcaceae	Thermococcus	Thermococcus thioreducens
1	Archaea	Euryarchaeota	Thermococci	Thermococcales	Thermococcaceae	Pyrococcus	Pyrococcus yayanosii
1	Archaea	Euryarchaeota	Thermococci	Thermococcales	Thermococcaceae	Palaeococcus	Palaeococcus pacificus
1	Archaea	Euryarchaeota	Theionarchaea	unclassified Theionarchaea	Theionarchaea archaeon DG-70		
1	Archaea	Euryarchaeota	Methanomicrobia	Methanosarcinales	unclassified Methanosarcinales	ANME-2 cluster	Candidatus Syntrophoarchaeum
1	Archaea	Euryarchaeota	Methanomicrobia	Methanosarcinales	Methanosarcinaceae	Methanosarcina	Methanosarcina vacuolata
1	Archaea	Euryarchaeota	Methanomicrobia	Methanosarcinales	Methanosarcinaceae	Methanosarcina	Methanosarcina sp. MTP4
1	Archaea	Euryarchaeota	Methanomicrobia	Methanosarcinales	Methanosarcinaceae	Methanosarcina	Methanosarcina sp. 2.H.A.1B.4
1	Archaea	Euryarchaeota	Methanomicrobia	Methanosarcinales	Methanosarcinaceae	Methanosarcina	Methanosarcina flavescens
1	Archaea	Euryarchaeota	Methanomicrobia	Methanosarcinales	Methanosarcinaceae	Methanolobus	Methanolobus psychrophilus
1	Archaea	Euryarchaeota	Methanomicrobia	Methanosarcinales	Methanosarcinaceae	Methanolobus	Methanolobus profundi
1	Archaea	Euryarchaeota	Methanomicrobia	Methanosarcinales	Methanosarcinaceae	Methanolobus	
1	Archaea	Euryarchaeota	Methanomicrobia	Methanosarcinales	Methanosarcinaceae	Methanohalophilus	Methanohalophilus sp. 2-GBenrich
1	Archaea	Euryarchaeota	Methanomicrobia	Methanosarcinales	Methanosarcinaceae	Methanococcoides	Methanococcoides burtonii
1	Archaea	Euryarchaeota	Methanomicrobia	Methanosarcinales	Methanosarcinaceae	Methanococcoides	
1	Archaea	Euryarchaeota	Methanomicrobia	Methanosarcinales	Methanosarcinaceae		
1	Archaea	Euryarchaeota	Methanomicrobia	Methanosarcinales	Methanosaetaceae	Methanosaeta	Methanosaeta sp. SDB
1	Archaea	Euryarchaeota	Methanomicrobia	Methanosarcinales	Methanosaetaceae	Methanosaeta	
1	Archaea	Euryarchaeota	Methanomicrobia	Methanosarcinales	Candidatus Methanoperedenaceae	Candidatus Methanoperedens	Candidatus Methanoperedens sp. BLZ1
1	Archaea	Euryarchaeota	Methanomicrobia	Methanomicrobiales	unclassified Methanomicrobiales	unclassified Methanomicrobiales (miscellaneous)	Methanomicrobiales archaeon 53_19
1	Archaea	Euryarchaeota	Methanomicrobia	Methanomicrobiales	Methanoregulaceae	Methanoregula	Methanoregula boonei
1	Archaea	Euryarchaeota	Methanomicrobia	Methanomicrobiales	Methanoregulaceae	Methanolinea	Methanolinea sp. SDB
1	Archaea	Euryarchaeota	Methanomicrobia	Methanomicrobiales	Methanomicrobiaceae	Methanoplanus	Methanoplanus limicola
1	Archaea	Euryarchaeota	Methanomicrobia	Methanomicrobiales	Methanomicrobiaceae	Methanogenium	Methanogenium cariaci
1	Archaea	Euryarchaeota	Methanomicrobia	Methanomicrobiales	Methanomicrobiaceae	Methanoculleus	Methanoculleus thermophilus
1	Archaea	Euryarchaeota	Methanomicrobia	Methanomicrobiales	Methanomicrobiaceae	Methanoculleus	Methanoculleus sp. MH98A
1	Archaea	Euryarchaeota	Methanomicrobia	Methanomicrobiales	Methanocorpusculaceae	Methanocorpusculum	Methanocorpusculum labreanum
1	Archaea	Euryarchaeota	Methanomicrobia				
1	Archaea	Euryarchaeota	Methanococci	Methanococcales	Methanococcaceae	Methanococcus	Methanococcus voltae
1	Archaea	Euryarchaeota	Methanococci	Methanococcales	Methanococcaceae	Methanococcus	Methanococcus aeolicus
1	Archaea	Euryarchaeota	Methanococci	Methanococcales	Methanocaldococcaceae		
1	Archaea	Euryarchaeota	Methanobacteria	Methanobacteriales	Methanobacteriaceae	unclassified Methanobacteriaceae	Methanobacteriaceae archaeon 41_258
1	Archaea	Euryarchaeota	Methanobacteria	Methanobacteriales	Methanobacteriaceae	Methanobrevibacter	Methanobrevibacter sp. YE315
1	Archaea	Euryarchaeota	Methanobacteria	Methanobacteriales	Methanobacteriaceae	Methanobrevibacter	Methanobrevibacter millerae
1	Archaea	Euryarchaeota	Methanobacteria	Methanobacteriales	Methanobacteriaceae	Methanobrevibacter	Methanobrevibacter cuticularis
1	Archaea	Euryarchaeota	Methanobacteria	Methanobacteriales	Methanobacteriaceae	Methanobacterium	Methanobacterium sp. SMA-27
1	Archaea	Euryarchaeota	Methanobacteria	Methanobacteriales	Methanobacteriaceae	Methanobacterium	Methanobacterium paludis
1	Archaea	Euryarchaeota	Methanobacteria	Methanobacteriales	Methanobacteriaceae	Methanobacterium	Methanobacterium formicicum
1	Archaea	Euryarchaeota	Methanobacteria	Methanobacteriales	Methanobacteriaceae	Methanobacterium	Methanobacterium congolense
1	Archaea	Euryarchaeota	Halobacteria	Natrialbales	Natrialbaceae	Salinarchaeum	Salinarchaeum sp. Harcht-Bsk1
1	Archaea	Euryarchaeota	Halobacteria	Natrialbales	Natrialbaceae	Natronorubrum	Natronorubrum texcoconense
1	Archaea	Euryarchaeota	Halobacteria	Natrialbales	Natrialbaceae	Natronobacterium	Natronobacterium gregoryi
1	Archaea	Euryarchaeota	Halobacteria	Natrialbales	Natrialbaceae	Natrinema	Natrinema salaciae
1	Archaea	Euryarchaeota	Halobacteria	Natrialbales	Natrialbaceae	Natrialba	Natrialba magadii
1	Archaea	Euryarchaeota	Halobacteria	Natrialbales	Natrialbaceae	Natrialba	Natrialba chahannaoensis
1	Archaea	Euryarchaeota	Halobacteria	Natrialbales	Natrialbaceae	Natrialba	Natrialba asiatica
1	Archaea	Euryarchaeota	Halobacteria	Natrialbales	Natrialbaceae	Halopiger	Halopiger xanaduensis
1	Archaea	Euryarchaeota	Halobacteria	Natrialbales	Natrialbaceae	Halopiger	Halopiger salifodinae
1	Archaea	Euryarchaeota	Halobacteria	Natrialbales	Natrialbaceae		
1	Archaea	Euryarchaeota	Halobacteria	Haloferacales	Halorubraceae	Halorubrum	
1	Archaea	Euryarchaeota	Halobacteria	Haloferacales	Halorubraceae	Halolamina	Halolamina sediminis
1	Archaea	Euryarchaeota	Halobacteria	Haloferacales	Halorubraceae	Halobaculum	Halobaculum gomorrense
1	Archaea	Euryarchaeota	Halobacteria	Haloferacales	Haloferacaceae	Halogranum	
1	Archaea	Euryarchaeota	Halobacteria	Haloferacales	Haloferacaceae	Haloferax	Haloferax sp. SB29
1	Archaea	Euryarchaeota	Halobacteria	Haloferacales	Haloferacaceae	Haloferax	Haloferax larsenii
1	Archaea	Euryarchaeota	Halobacteria	Halobacteriales	Halococcaceae	Halococcus	Halococcus sediminicola
1	Archaea	Euryarchaeota	Halobacteria	Halobacteriales	Halococcaceae	Halococcus	Halococcus morrhuae
1	Archaea	Euryarchaeota	Halobacteria	Halobacteriales	Halococcaceae	Halococcus	Halococcus hamelinensis
1	Archaea	Euryarchaeota	Halobacteria	Halobacteriales	Halococcaceae	Halococcus	
1	Archaea	Euryarchaeota	Halobacteria	Halobacteriales	Halobacteriaceae	Halarchaeum	Halarchaeum acidiphilum
1	Archaea	Euryarchaeota	Halobacteria	Halobacteriales	Halobacteriaceae	Halalkalicoccus	Halalkalicoccus jeotgali
1	Archaea	Euryarchaeota	Halobacteria	Halobacteriales	Halobacteriaceae	Haladaptatus	Haladaptatus litoreus
1	Archaea	Euryarchaeota	Halobacteria	Halobacteriales	Halobacteriaceae	Haladaptatus	Haladaptatus cibarius
1	Archaea	Euryarchaeota	Halobacteria	Halobacteriales	Halobacteriaceae	Haladaptatus	
1	Archaea	Euryarchaeota	Halobacteria	Halobacteriales	Haloarculaceae	Halosimplex	Halosimplex carlsbadense
1	Archaea	Euryarchaeota	Halobacteria	Halobacteriales	Haloarculaceae	Halorientalis	Halorientalis regularis
1	Archaea	Euryarchaeota	Halobacteria	Halobacteriales	Haloarculaceae	Halapricum	Halapricum salinum
1	Archaea	Euryarchaeota	Hadesarchaea	unclassified Hadesarchaea	Hadesarchaea archaeon YNP_45		
1	Archaea	Euryarchaeota	Hadesarchaea	unclassified Hadesarchaea	Hadesarchaea archaeon DG-33		
1	Archaea	Euryarchaeota	Archaeoglobi	Archaeoglobales	Archaeoglobaceae	Geoglobus	Geoglobus ahangari
1	Archaea	Euryarchaeota	Archaeoglobi	Archaeoglobales	Archaeoglobaceae	Archaeoglobus	Archaeoglobus sulfaticallidus
1	Archaea	Euryarchaeota	Archaeoglobi	Archaeoglobales	Archaeoglobaceae	Archaeoglobus	Archaeoglobus fulgidus
1	Archaea	DPANN group	Candidatus Woesearchaeota	Candidatus Woesearchaeota archaeon CG1_02_57_44			
1	Archaea	DPANN group	Candidatus Woesearchaeota	Candidatus Woesearchaeota archaeon CG1_02_33_12			
1	Archaea	DPANN group	Candidatus Micrarchaeota	unclassified Candidatus Micrarchaeota	Candidatus Micrarchaeota archaeon CG1_02_51_15		
1	Archaea	DPANN group	Candidatus Micrarchaeota	Candidatus Micrarchaeum	Candidatus Micrarchaeum sp. AZ1		
1	Archaea	Asgard group	Candidatus Thorarchaeota	Candidatus Thorarchaeota archaeon SMTZ1-83			
1	Archaea	Asgard group	Candidatus Thorarchaeota	Candidatus Thorarchaeota archaeon SMTZ1-45			
1	Archaea	Asgard group	Candidatus Thorarchaeota				
1	Archaea	Asgard group	Candidatus Heimdallarchaeota	Candidatus Heimdallarchaeota archaeon LC_3			
1	unclassified bacterial viruses	Shewanella phage SppYZU01					
1	unclassified bacterial viruses	Alteromonas phage PB15					
1	ssDNA viruses	Microviridae	Bullavirinae	Phix174microvirus	unclassified Phix174microvirus	Enterobacteria phage phiX174 sensu lato	Enterobacteria phage ID45
1	ssDNA viruses	Microviridae	Bullavirinae	Phix174microvirus			
1	environmental samples	uncultured marine virus					
1	environmental samples	uncultured Mediterranean phage					
1	dsDNA viruses, no RNA stage	unclassified dsDNA viruses	Pandoravirus dulcis				
1	dsDNA viruses, no RNA stage	Polydnaviridae	Bracovirus	unclassified Bracovirus	Cotesia vestalis bracovirus		
1	dsDNA viruses, no RNA stage	Iridoviridae	Iridovirus	unclassified Iridovirus			
1	dsDNA viruses, no RNA stage	Caudovirales	Siphoviridae	unclassified Siphoviridae	Rhizobium phage 16-3		
1	dsDNA viruses, no RNA stage	Caudovirales	Siphoviridae	unclassified Siphoviridae	Geobacillus virus E3		
1	dsDNA viruses, no RNA stage	Caudovirales	Siphoviridae	unclassified Siphoviridae			
1	dsDNA viruses, no RNA stage	Caudovirales	Siphoviridae	Xp10virus	Xanthomonas phage phiL7		
1	dsDNA viruses, no RNA stage	Caudovirales	Siphoviridae	Np1virus	Pseudomonas virus PaMx25	Pseudomonas phage PaMx25	
1	dsDNA viruses, no RNA stage	Caudovirales	Siphoviridae	Np1virus			
1	dsDNA viruses, no RNA stage	Caudovirales	Siphoviridae	Ab18virus	Pseudomonas virus Ab18	Pseudomonas phage vB_PaeS_PAO1_Ab18	
1	dsDNA viruses, no RNA stage	Caudovirales	Siphoviridae				
1	dsDNA viruses, no RNA stage	Caudovirales	Podoviridae	unclassified Podoviridae	Ralstonia phage RSK1		
1	dsDNA viruses, no RNA stage	Caudovirales	Podoviridae	unclassified Podoviridae	EBPR podovirus 2		
1	dsDNA viruses, no RNA stage	Caudovirales	Podoviridae	unclassified Podoviridae	Caulobacter phage Percy		
1	dsDNA viruses, no RNA stage	Caudovirales	Myoviridae	unclassified Myoviridae	Acinetobacter phage AM24		
1	dsDNA viruses, no RNA stage	Caudovirales	Myoviridae	Marthavirus	Arthrobacter virus Jawnski	Arthrobacter phage Jawnski	
1	dsDNA viruses, no RNA stage	Caudovirales					
1	Retro-transcribing viruses	Retroviridae	Orthoretrovirinae	Gammaretrovirus	Mammalian gammaretrovirus group	Replication defective viruses	Hardy-Zuckerman feline sarcoma virus
1	Retro-transcribing viruses	Retroviridae	Orthoretrovirinae	Alpharetrovirus	unclassified Alpharetrovirus	Avian musculoaponeurotic fibrosarcoma virus AS42	
146910							
